# Genomic analysis of ionome-related QTLs in *Arabidopsis thaliana*

**DOI:** 10.1038/s41598-021-98592-7

**Published:** 2021-09-28

**Authors:** Nikwan Shariatipour, Bahram Heidari, Samathmika Ravi, Piergiorgio Stevanato

**Affiliations:** 1 Department of Plant Production and Genetics, School of Agriculture, Shiraz University, 7144165186 Shiraz, Iran; 2 Department of Agronomy, Animals, Natural Resources and Environment‐ DAFNAE, University of Padova, Legnaro, Padova Italy

**Keywords:** Plant biotechnology, Plant breeding, Plant genetics, Biomimetics, Functional genomics

## Abstract

Ionome contributes to maintain cell integrity and acts as cofactors for catalyzing regulatory pathways. Identifying ionome contributing genomic regions provides a practical framework to dissect the genetic architecture of ionomic traits for use in biofortification. Meta-QTL (MQTL) analysis is a robust method to discover stable genomic regions for traits regardless of the genetic background. This study used information of 483 QTLs for ionomic traits identified from 12 populations for MQTL analysis in *Arabidopsis thaliana*. The selected QTLs were projected onto the newly constructed genetic consensus map and 33 MQTLs distributed on *A. thaliana* chromosomes were identified. The average confidence interval (CI) of the drafted MQTLs was 1.30 cM, reduced eight folds from a mean CI of 10.88 cM for the original QTLs. Four MQTLs were considered as stable MQTLs over different genetic backgrounds and environments. In parallel to the gene density over the *A. thaliana* genome, the genomic distribution of MQTLs over the genetic and physical maps indicated the highest density at non- and sub-telomeric chromosomal regions, respectively. Several candidate genes identified in the MQTLs intervals were associated with ion transportation, tolerance, and homeostasis. The genomic context of the identified MQTLs suggested nine chromosomal regions for Zn, Mn, and Fe control. The QTLs for potassium (K) and phosphorus (P) were the most frequently co-located with Zn (78.3%), Mn (76.2%), and Fe (88.2% and 70.6%) QTLs. The current MQTL analysis demonstrates that meta-QTL analysis is cheaper than, and as informative as genome-wide association study (GWAS) in refining the known QTLs.

## Introduction

Nutrient elements contribute to the plant’s tolerance to abiotic stresses and detoxification of reactive oxygen species (ROS) by altering metabolic pathways involved in gene expression; biosynthesis of proteins, carbohydrates, and lipids; and production of phytohormones that protect plants from ROS-induced injury^1–3^. In crops, essential nutrient concentration affects the quality and quantity of crop production^1^ and a nutrient imbalance result in various disorders in the world’s population especially for plant-based dietary societies. Among nutrient elements, the essential trace elements such as zinc (Zn), iron (Fe) and manganese (Mn) play crucial role in human health, immune system and life longevity^4,5^. Trace elements are involved in psychomotor development, maintenance of physical activity, and resistance to infection^5,6^. The deficiency of trace elements causes several health problems including retarded growth, skeletal abnormalities, increased abortion risk, anemia, diarrhea, impaired mental development, and learning capacity^7,8^. An inadequate intake of essential nutrients like iron and zinc which is known as “hidden hunger” affects around two billion people globally^9^. Today, agriculture is undergoing a shift from producing more quantity of food crops to producing higher quality such as nutrient-rich food crops in sufficient quantities.

All mineral nutrient, trace elements (including both essential and non-essential elements, metals and non-metals), and the inorganic component of cellular and organismal systems constitutes the “Ionome”^10^. It is an important determinant of the physiological state of an organism. For example, iron deficiency in a plant can be identified by looking at a number of other elements, rather than iron itself. The ionomics is a powerful approach for analysis of dynamic network of elements that are controlled by the genome of the plant in response to the environmental conditions^11^. Understanding the ionome function and dynamics is also vital to uncover how living organisms work, especially for plants that depend on external sources for the uptake of almost all elements except oxygen and carbon^10,11^. The complex nature of the molecular mechanism of ionomic variation requires detailed information illustrated at the OMICs (biological sciences ends with omics such as genomics, transcriptomics, proteomics, or metabolomics) levels and highlights critical concepts for assessment of the ionome^12,13^. Thus, ionomics should depict the functional status of a complex biological organism in both a quantitative and qualitative pattern of elements in various components of the organism^10^.

The ionomics method refers to quantitative analysis of ionomic traits and their changes in living organisms in response to developmental state, physiological stimuli and genetic modifications. In addition to ionomics, the application of practical genetic analysis such as the genetic mapping approach leads to an understanding of the genetic architecture of ionomic traits. The use of molecularly well-characterized and diverse germplasms of model species like *Arabidopsis thaliana* expands the knowledge about ionomic variation and their associated genomic regions. Various QTLs have been identified using linkage mapping approaches for numerous important ionomic traits in plants^14–19^. Traditional QTL mapping has been successful in identifying genomic regions controlling the variation of ionomic traits. In traditional QTL mapping models, the genetic effects of QTL identified in an individual population may not be validated in other genetic backgrounds or environments and more often, only a limited number of traits can be tested in a single study^20^. However, meta- quantitative trait loci (Meta-QTL or MQTL) overcomes the limitation posed by traditional QTL analysis^21,22^. In the meta-QTL model, QTL data from independent studies and populations are used to detect consensus QTL regions known as Meta-QTL^23^. Further, the information on QTLs derived from various population structure, origin and sizes can be used to refine the known QTLs regardless of the genetic backgrounds, marker density and phenotypic variations^21,24,25^. In this way, a meta-QTL analysis helps to narrow down the confidence interval (CI) of the initial QTLs identified in independent populations and lays the foundation for a better understanding of traits underlying a QTL region than what is possible in independent QTL mapping studies^26^. Moreover, the MQTL analysis helps to validate the genetic association of loci identified by genome-wide association study (GWAS) approach^27–29^.

Despite the wide use of meta-QTL analysis in plant species^30–39^, little is known about ionomic meta- QTL analysis in *A. thaliana*. This study aims to perform a meta-QTL analysis to uncover genomic control of ionome related traits in *A. thaliana*. Due to the importance of essential elements in biofortification studies, the emphasis of the current study is placed on the assessment of genomic regions controlling trace elements, especially Zn, Fe, and Mn. In addition, the ability of MQTL analysis to validate the loci associated with micronutrients identified by GWAS^40^ was assessed in our study.

## Materials and methods

### Development of QTL database

**Figure 1:**
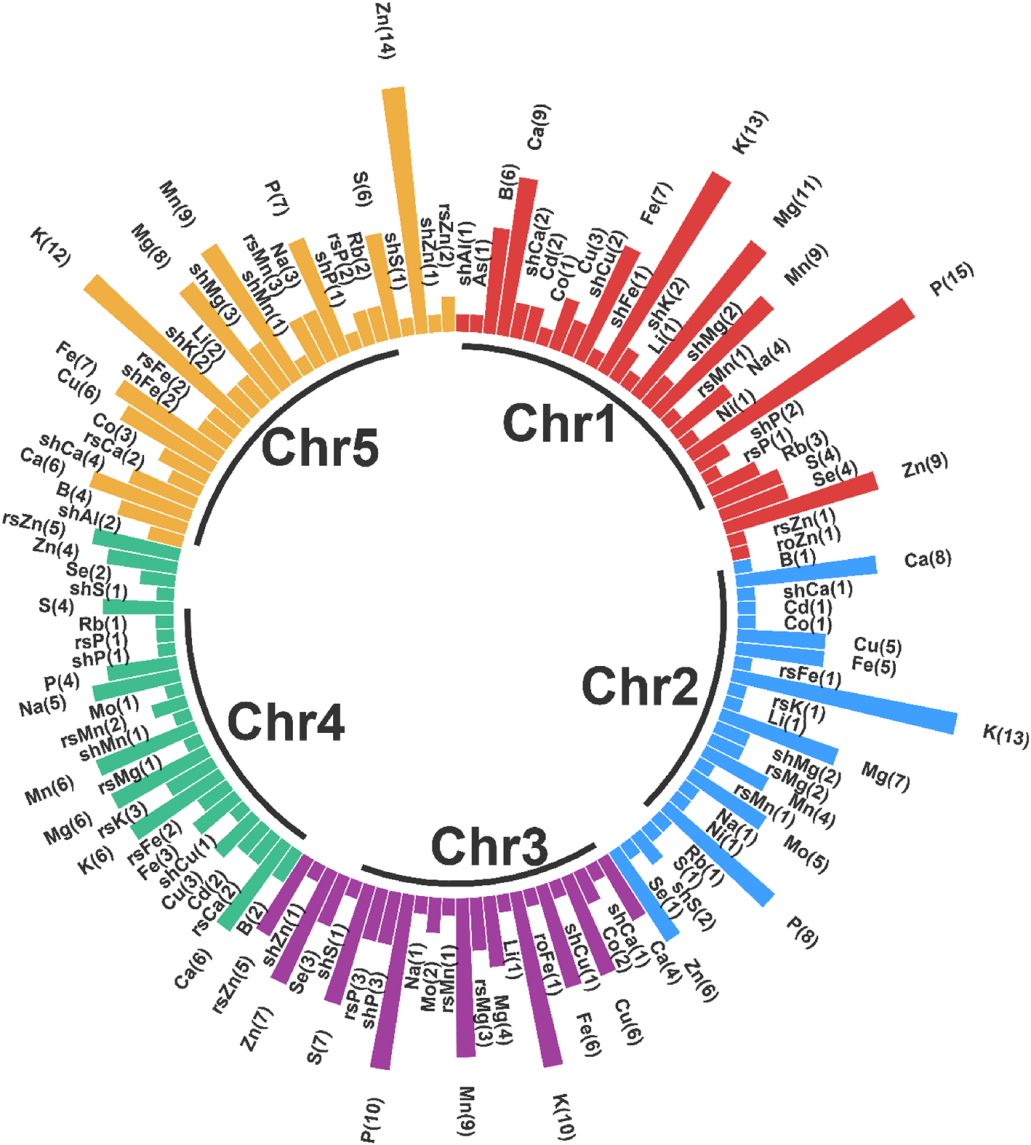
Distribution of QTL associated with different ionomic traits across the *A. thaliana* chromosomes. The numbers inside parentheses represent the number of QTL in each trait.

A database containing information on 483 QTLs for 38 different traits (Fig. 1) derived from 12 segregating populations was developed. The number of QTL per study ranged from 5 to 83 QTLs that were used to find the most stable genomic regions controlling ionomic quantitative traits in *A. thaliana* (Table 1). Except for those lacking proper genetic map and QTL-related information, QTL mapping studies including important quantitative traits with different markers in *A. thaliana* were used for the identification of MQTLs. The information of populations used in the meta-QTL analysis is summarized in Table 1. The number of QTLs for each ionomic trait and their distribution on *A. thaliana* chromosomes are presented in Fig. 1 and Supplementary Figure S1. The assessed ionomic QTLs were distributed across different chromosomes (χ_(4)_^2^ = 17.40, P = 0.0016) with chromosome 1 showing the highest number of QTLs (119 QTLs), followed by chromosome 5 (117 QTLs). Chromosomes 4 and 2 had the lowest number of QTLs (Fig. 1). The chromosomes 1 and 5 with 9 and 7 QTLs possessed the highest QTL numbers for Mn and Fe, respectively (Fig. 1). The highest number of QTLs for Zn were located on chromosome 5 with 14 QTLs. Details regarding the position, the proportion of phenotypic variance (% Expl.), and the log of odds ratios (LOD score) for QTLs were used for the analysis of meta-QTLs.

**Table 1:** Summary of the selected QTLs used in meta-QTL analysis for ionomic profile in *Arabidopsis thaliana.*

Study	Population number	Parents	Population type	Population size	Number of markers	Number of trait QTLs	Genome length (cM)	Trait	References
1	4	Bay × Shadahara	RIL	165	69	49	464.5	B, Ca, Cd, Co, Cu, Fe, K, Li, Mg, Mn, Mo, Na, P, Se, Zn	^41^
		Bay × Shadahara	RIL	411	69	83	464.5	As, B, Ca, Cd, Co, Cu, Fe, K, Li, Mg, Mn, Mo, Na, Ni, P, Rb, S, Se, Zn	
		Ler × Cvi	RIL	151	93	23	547.4	B, Ca, Cd, Co, Cu, Fe, K, Mg, Mn, Mo, Na, P, Se, Zn	
		Ler × Cvi	RIL	161	93	42	542.7	B, Ca, Cd, Co, Cu, Fe, K, Li, Mg, Mn, Mo, Na, P, Zn	
2	1	Ler × An-1	RIL	120	64	37	435.1	rsCa, rsFe, rsK, rsMg, rsMn, rsP, rsZn	^42^
3	3	Ler × Kond	RIL	120	75	32	401.1	Ca, Fe, K, Mg, Mn, Zn, roFe, roZn, rsFe, rsMn, rsZn^a^	^43^
		Ler × An-1	RIL	120	64	40	435.1	Ca, Fe, K, Mg, Mn, P, Zn rsMn, rsZn^a^	
		Ler × Eri	RIL	110	115	5	398.1	K, Mn	
4	1	Ler × Cvi	RIL	162	117	45	512.0	shAl, shCa, shCu, shFe, shK, shMg, shMn, shP, shS, shZn^a^	^16^
5	1	Ler × Cvi	RIL	158	288	28	486.6	Ca, Fe, K, Mg, Mn, P, Zn	^14^
6	2	Ler × Cvi	RIL	305	164	49	499.6	Ca, Cu, Fe, K, Mg, Mn, P, S, Zn	^44^
		Col × Ler	RIL	197	210	50	642.9	Ca, Cu, Fe, K, Mg, Mn, P, S, Zn	

RIL, As, B, Ca, Cd, Co, Cu, Fe, K, Li, Mg, Mn, Mo, Na, Ni, P, Rb, S, Se and Zn stands for recombinant inbred line, arsenic, boron, calcium, cadmium, cobalt, copper, iron, potassium, lithium, magnesium, manganese, molybdenum, sodium, nickel, phosphorus, rubidium, sulfur, selenium and zinc in caryopsis, respectively.

^a^rs, ro and sh represent rosette, root and shoot. rsZn and shFe stand for rosette Zn and shoot Fe, respectively.

### Constructing consensus genetic map and QTL projection

A consensus genetic map was constructed by integrating 12 map files. The constructed map file for each population included information about the type of cross, population size, map function, map units and the position of DNA markers used in different linkage groups. For map comparisons, the genome lengths from the individual and consensus genetic maps were calculated according to Hubert and Hedgecock^45^. First, the average spacing (χ) between markers was calculated by dividing the total length of all linkage groups by the number of intervals (number of markers minus the number of linkage groups). Then, the genome lengths for individual and consensus linkage maps were calculated by adding 2χ to the length of each linkage group to account for terminal chromosome regions^46^. After the development of the final consensus genetic map, the individual QTLs obtained from each population were projected onto the consensus genetic map in BioMercator v 4.2^24,25^.

### Meta-QTL analysis

**Figure 2:**
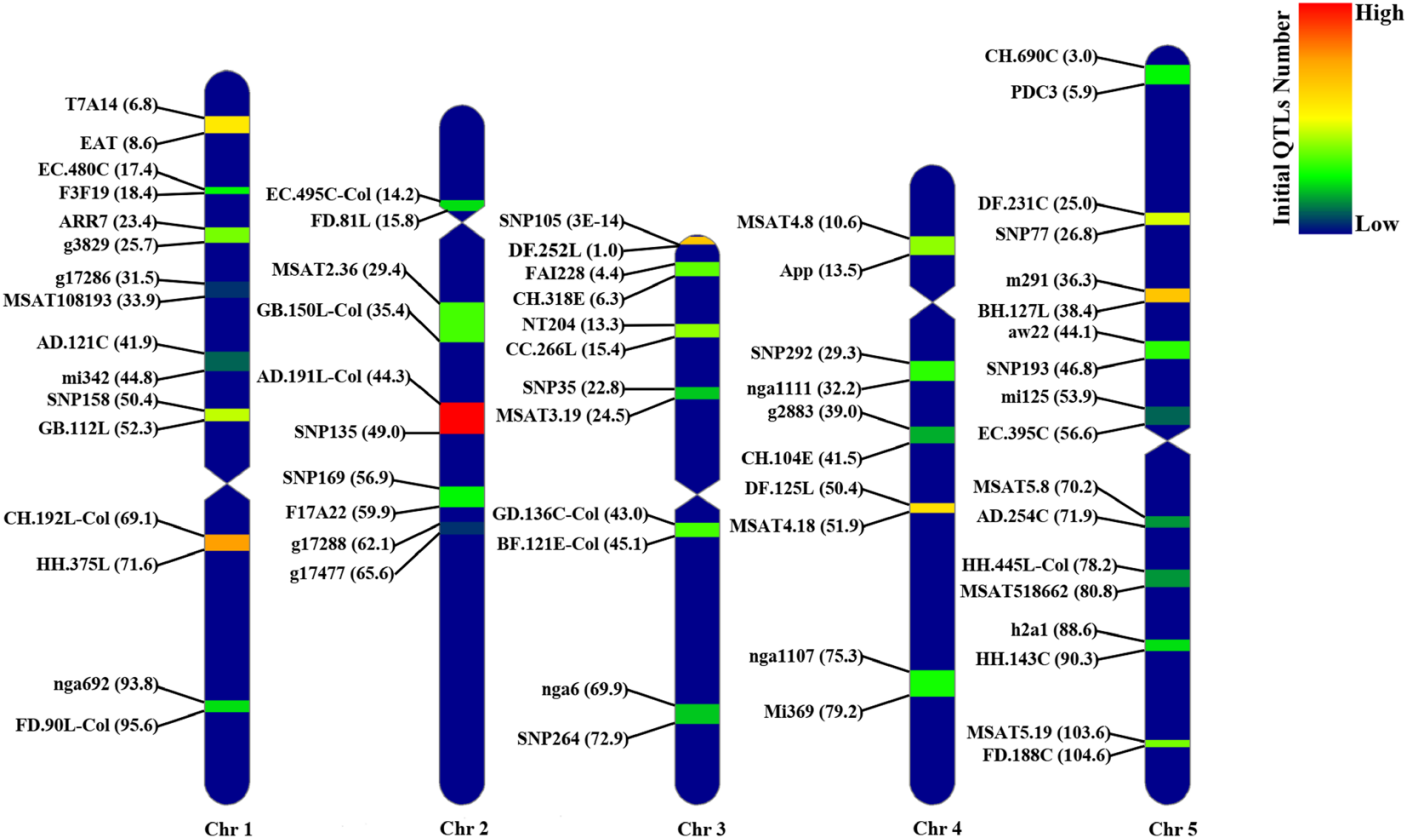
Position of identified MQTLs on *Arabidopsis* genome associated with ionomic quantitative traits with 95% confidence interval. Each color in different linkage groups indicates the number of initial QTLs involved in each MQTL. The flanking markers for each MQTL are presented on the left side of linkage groups (cM).

The BioMercator v4.2 was^24,25^ used to perform the meta-QTL analysis (available at https://urgi.versailles.inra.fr/Tools/BioMercator-V4). The Veyrieras et al.^22^ approach was used for meta-QTL analysis where the number of QTLs in each chromosome was higher than 10 and the best QTL model was selected based on Akaike information criterion (AIC)^22^, corrected AIC criterion (AICc)^22^, AIC 3 candidate (AIC3)^22^, Bayesian information criterion (BIC)^22^, and Approximate Weight of Evidence (AWE)^22^. The best QTL model shows lower values in at least three of these five criterions. Consensus QTLs from the optimum model was regarded as meta-QTLs (MQTLs). The MQTL position and distribution in each linkage group were presented as a heatmap using the *RIdeogram* R package^47^ (Fig. 2).

**Figure 3:**
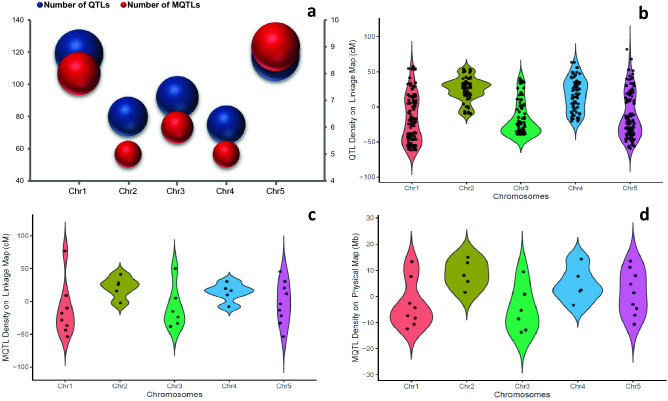
Number and distribution of QTLs and MQTLs over *Arabidopsis* genome. (**a**) comparison between QTLs and MQTLs number on five chromosomes of *Arabidopsis thaliana*; (**b**) Distribution of assessed QTLs for ionomic traits over genetic consensus map; (**c**) Projection of the detected MQTLs over *Arabidopsis* genome based on genetic map; (**d**) Distribution of MQTLs for ionomic quantitative traits on physical map of *Arabidopsis thaliana*. The projection of QTLs and MQTLs on genetic map were illustrated as number per 50 cM distance. For physical map 10 Mb was considered. The distance was started from the centromeric region of each chromosome where it was considered as position 0 cM or Mb. Dots represent the location of QTLs and MQTLs.

**Figure 4:**
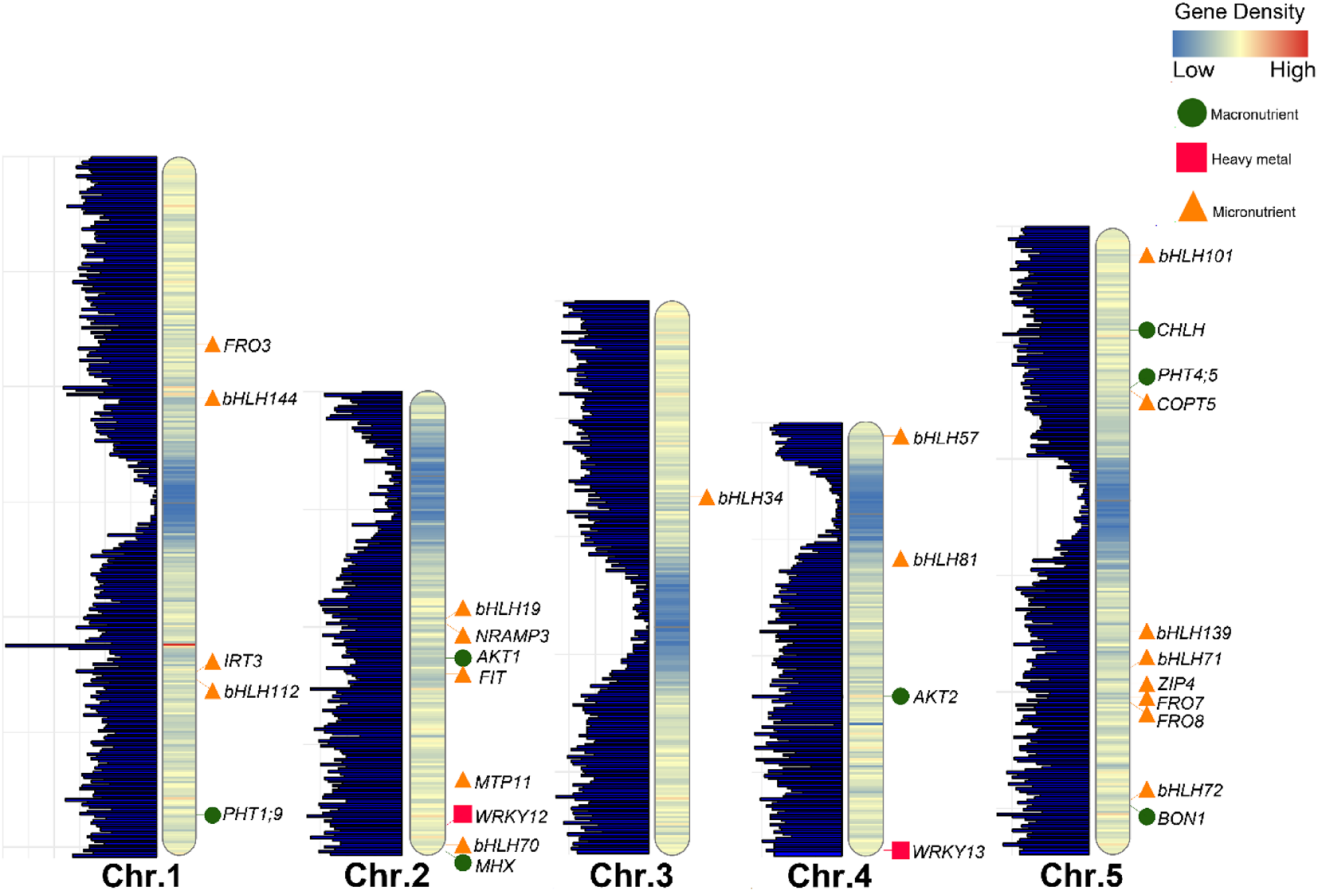
The gene density of *Arabidopsis* *thaliana* chromosomes based on heat map and bar plot illustrations. The position of proved genes (detected in MQTLs interval) for different ionomic traits are presented in the right side of chromosomes.

The variation of QTLs of traits over genetic consensus map and the MQTLs density in both genetic and physical maps regions were estimated following the study by Martinez et al.^32^. The QTL and MQTL densities on the genetic map were evaluated by counting the number of QTLs and MQTLs in every 50 cM interval across the *A. thaliana* genome, starting from the centromeric region towards the ends of the chromosome (Fig. 3). The same procedure was followed for estimating MQTLs density in 10 Mb intervals across the *A. thaliana* physical map. The centromere position for genetic and physical maps of *A. thaliana* was retrieved from The *Arabidopsis* Information Resource (TAIR) database [https://www.arabidopsis.org/]. The distribution of QTLs and MQTLs was also compared in perspective with the gene density calculated with a bin size of in 0.1 Mb distance in each chromosome using the gff file of TAIR10. The projection of gene densities in comparison with the MQTLs was generated using the *RIdeogram* R package^47^ (Fig. 4).

The genetic loci highly associated with grain Zn, Fe, and Mn (as the most targeted mineral elements in biofortification studies) in the *A. thaliana* GWAS study was retrieved and also compared with the MQTLs and the results of GWAS based on SNP markers^40^. The genomic positions of MQTLs and associated loci reported in the *A. thaliana* GWAS study^40^ were compared based on the *A. thaliana* genome.

### Functional candidate genes (CGs) in MQTLs interval

To uncover functional CGs controlling the ionomic traits (especially for grain Zn and Fe content), the sequences of the flanking markers for each MQTL were used to identify the corresponding genomic interval on the *A. thaliana* genome. The sequences of flanking markers were retrieved from the TAIR10 genome and BLAST was used to identify the precise genomic location. For those flanking markers lacking a definite position on the *A. thaliana* genome, the closest marker on the consensus map was selected to identify the MQTL position on the genome. The corresponding gene annotation was retrieved from the gff file containing gene coordinates for TAIR10 in addition to the Uniprot ID corresponding to the *A. thaliana* reference genome (TAIR10).

### Trait analysis within MQTL

To identify ionomic traits within MQTL regions, the MQTLs result was converted into binary scores (0 or 1) based on the absence/presence of an individual trait QTL within an MQTL region. Hence, the number of times a trait was present within an MQTL, the number of times more than one QTL for a trait was present within MQTL (implying confirmation of the QTL), and the number of times traits co-localized within an MQTL were calculated. A chi-squared test with a degree of freedom (DF) = 1 was performed to determine traits showing significant co-localization with grain Zn, Fe, and Mn contents beyond what would be expected for a random distribution of QTL within MQTL throughout the genome. The expected number of each Zn, Fe, and Mn MQTL co-localized with each tested ionomic trait was calculated using Eq. (1) that was 0.7, 0.52, and 0.64 for Zn, Fe, and Mn, respectively.

1 $${\text{MQTL }}_{{\text{P}}} = { }\frac{{{\text{Number}}\,{\text{of}}\,{\text{MQTLs}}\,{\text{containg}}\,{\text{target}}\,{\text{traits}}\,{\text{ QTL}}\left( {\text{s}} \right){ }}}{{{\text{All }}\,{\text{detected}}\,{\text{MQTLs}}}}$$

Traits within MQTL regions were further analyzed using IBM SPSS Statistics v.24.

## Results

### Genetic consensus map and meta-QTL analysis

The original linkage map consisted of five linkage groups with an average of 118 DNA markers and covered 485.8 cM of the *A. thaliana* genome (Table 2). The constructed consensus genetic map contained 725 markers that covered 514.01 cM of the *A. thaliana* genome (Table 2).

**Table 2:** The information on independent and consensus linkage maps used in meta-analysis in *A. thaliana.*

Linkage group (chromosome)	Independent linkage map		Consensus linkage map	
	Genome length (cM)	Number of marker	Genome length (cM)	Number of marker
Chr. 1	120.8	29	110.76	175
Chr. 2	80.5	19	105.64	112
Chr. 3	95.2	24	86.32	146
Chr. 4	87.4	19	96.72	122
Chr. 5	101.9	27	114.57	170
Average	97.2	24	102.8	145
Total	485.8	118	514.01	725

All the 483 initial QTLs were successfully projected onto the consensus genetic map and used for meta-QTL analysis. The meta-QTL analysis confined these QTLs into 33 MQTLs with QTLs originating from at least two populations for all the ionomic traits. The distribution of MQTLs for each ionomic trait on each chromosome is presented in Table 3, Fig. 2, and Supplementary Figure S1. Of these MQTLs, 22 MQTLs (60.67%) were obtained from at least six independent populations (Table 3; Supplementary Figure S1).

**Table 3:** Description of the detected meta-quantitative trait loci (MQTL) for ionome traits in *Arabidopsis thaliana.*

Meta-QTL	Chr	Flanking markers	Position on the consensus map (cM)	CI (cM)	Genomic position on the *Arabidopsis* genome (Mb)	No. of initial QTLs	No. of population	Trait	No. of genes laying identified in the MQTL interval
MQTL-1/Chr1	1	T7A14 ‒ EAT	7.91	0.71	2.66–2.77	24	9	shAl, B, shCa, Fe, shFe, K, shK, Mg, shMg, Mn, P, shP, S, Zn, rsZn	29
MQTL-2/Chr1	1	EC.480C ‒ F3F19	17.86	0.29	4.31–4.60	11	7	B, Ca, Cd, Cu, Mn, Na, P, shP, Se, Zn	91
MQTL-3/Chr1	1	ARR7 ‒ g3829	24.63	1.3	6.58–7.09	16	8	B, Ca, Cd, Fe, K, Mg, Mn, rsMn, Na, S	170
MQTL-4/Chr1	1	g17286 ‒ MSAT108193	33.02	1.91	7.30–8.19	2	2	Mg, Na	258
MQTL-5/Chr1	1	AD.121C ‒ mi342	43.33	1.85	10.16–11.41	5	5	K, Fe, Mn, P	335
MQTL-6/Chr1	1	SNP158 ‒ GB.112L	51.24	1.08	12.18–12.73	19	6	Ca, Co, Fe, K, Mg, P, Rb, Se, Zn	128
MQTL-7/Chr1	1	CH.192L-Col ‒ HH.375L	70.62	1.94	22.00–23.49	32	9	As, B, Ca, Cu, shCu, Fe, K, Mg, Ni, P, rsP, Rb, S, Se, Zn, roZn	459
MQTL-8/Chr1	1	nga692 ‒ FD.90L-Col	94.16	0.11	28.01–28.84	10	7	Mn, Li, P, S, Zn	285
MQTL-1/Chr2	2	EC.495C-Col ‒ FD.81L	15.06	1.46	4.96–5.30	10	5	Li, Ca, Cd, Co, Cu, K, Mg, P, Se	20
MQTL-2/Chr2	2	MSAT2.36 ‒ GB.150L-Col	33.36	3.57	8.69–9.98	14	9	B, Ca, shCa, K, rsK, rsMg, shMg, P, Zn	432
MQTL-3/Chr2	2	AD.191L-Col ‒ SNP135	45.9	2.31	11.22–12.11	42	10	Ca, Cu, Fe, rsFe, K, Mg, rsMg, Mn, rsMn, Mo, Na, P, shS, Zn	237
MQTL-4/Chr2	2	SNP169 ‒ F17A22	58.42	2.61	17.64–19.56	11	7	Ca, Cu, K, Mg, Mo, Rb, Zn	693
MQTL-5/Chr2	2	g17288 ‒ g17477	64.45	0.45	16.29–16.78	3	3	Cu, K, Ni	160
MQTL-1/Chr3	3	SNP105 ‒ DF.252L	0.44	0.63	0.05–0.18	28	7	Ca, Fe, K, Li, Mg, rsMg, Mn, P, rsP, shP, S, Zn, rsZn	61
MQTL-2/Chr3	3	FAI228 ‒ CH.318E	5.16	1.32	0.79–1.08	15	7	Ca, K, roFe, rsMg, Mn, Mo, Na, P, rsP, Se, Zn, rsZn	99
MQTL-3/Chr3	3	NT204 ‒ CC.266L	14.78	1.21	4.95–5.57	17	8	Ca, shCa, Co, Cu, shCu, Fe, K, Mn, P, Se, Zn, shZn	220
MQTL-4/Chr3	3	SNP35 ‒ MSAT3.19	23.55	1.51	8.17–8.81	9	5	Co, Cu, Mg, rsMg, Mn, rsP, Zn, rsZn	175
MQTL-5/Chr3	3	GD.136C-Col ‒ BF.121E-Col	43.91	0.87	14.53–14.72	14	7	Cu, Fe, Mg, Mn, rsMn, P, S, Zn, rsZn	9
MQTL-6/Chr3	3	nga6 ‒ SNP264	70.42	0.38	23.04–23.39	9	6	Cu, K, P, shP, S, shS, Zn	131
MQTL-1/Chr4	4	MSAT4.8 ‒ app	12.41	1.04	0.41–0.90	17	4	Ca, Fe, rsFe, K, rsK, Mg, Mn, Na, P, rsP, Rb, S, rsZn	154
MQTL-2/Chr4	4	SNP292 ‒ nga1111	30.53	2.29	5.87–6.09	13	5	B, Ca, rsCa, Fe, rsFe, K, rsK, Na, Se, Zn, rsZn	774
MQTL-3/Chr4	4	g2883 ‒ CH.104E	40.23	1.35	6.25–6.62	8	3	Ca, K, Mg, Mn, rsMn, P, shS, Zn	97
MQTL-4/Chr4	4	DF.125L ‒ MSAT4.18	51.1	1.08	11.52–11.97	25	10	Ca, rsCa, Cd, Cu, K, rsK, Mg, rsMg, Mn, rsMn, Mo, Na, P, S, Se, Zn, rsZn	150
MQTL-5/Chr4	4	nga1107 ‒ mi369	75.49	0.1	18.10–18.54	12	6	B, Ca, Cu, shCu, Mg, Mn, shMn, Na, shP, Zn	161
MQTL-1/Chr5	5	CH.690C ‒ PDC3	5.4	0.96	0.77–1.32	11	4	shAl, B, Ca, shCa, Fe, K, Mn, shMn	170
MQTL-2/Chr5	5	DF.231C ‒ SNP77	26.02	1.29	3.46–5.64	20	7	B, Ca, rsCa, Co, Fe, K, shK, Mg, Mn, rsMn, Na, P, rsP, shP, S, rsZn	692
MQTL-3/Chr5	5	m291 ‒ BH.127L	37.21	1.29	6.68–7.49	28	9	shAl, rsCa, shCa, Co, Cu, Fe, shFe, K, Mg, shMg, Mn, Na, P, S, Zn, shZn	252
MQTL-4/Chr5	5	aw22 ‒ SNP193	45.47	1.96	8.50–8.79	13	6	Ca, K, rsFe, shFe, Li, Mg, Mn, P, Zn	69
MQTL-5/Chr5	5	mi125 ‒ EC.395C	55.24	2.37	12.91–13.28	5	4	Fe, rsMn, Rb, S, shS,	21
MQTL-6/Chr5	5	MSAT5.8 ‒ AD.254C	70.77	0.91	15.46–17.35	7	4	Cu, Fe, K, Mg, Mn	585
MQTL-7/Chr5	5	HH.445L-Col ‒ MSAT518662	79.5	0.98	18.66–20.66	7	6	Fe, K, shMg, P, S, Zn	608
MQTL-8/Chr5	5	h2a1 ‒ HH.143C	89.58	1.51	22.39–23.30	10	6	B, Cu, K, Mn, Na, P, S, Zn	283
MQTL-9/Chr5	5	MSAT5.19 ‒ FD.188C	104.22	0.1	24.53–25.92	16	7	Ca, Co, K, Li, Mg, rsMn, Rb, rsFe, rsP, S, Zn	468

Chr., chromosome.

Chromosome 5 harbored the largest number (9 MQTLs) and chromosomes 2 and 4 had the lowest number of MQTLs (Table 3, Fig. 2). The MQTLs with the highest initial QTLs could be considered as the more stable regions which control ionomic traits independent of genetic background and environment. Among the identified MQTLs in our study, MQTL-3/Chr2 had the highest number of initial QTLs (42 QTLs) derived from 10 studies followed by MQTL-7/Chr1, MQTL-1/Chr3, and MQTL-3/Chr5 with 32, 28 and 28 initial QTLs derived from nine, seven, and nine independent population studies, respectively (Table 3). The results showed that the “CH.192L-Col”, “HH.357L”, “AD.191L-Col”, “SNP135”, “SNP105”, “DF.252L”, “m291” and “BH.127L” markers were linked to these stable MQTLs.

The current meta-QTL analysis reduced the average confidence interval (CI) up to eight folds with an average of 1.30 cM in MQTLs in comparison to the mean CI of the original QTLs (10.88 cM). Among the detected MQTLs, the CI of 12 MQTLs (36.4%) was reduced to < 1 cM (Table 3).

### Gene density and distribution of MQTLs and QTLs on chromosomes

The distribution patterns of QTLs on the consensus genetic map and MQTLs on the genetic and physical maps were investigated and compared with gene density (Figs. 3, 4). The overview of the distribution of the assessed QTLs for different ionomic traits exhibited that the non-telomeric regions possessed majority of QTLs and MQTLs in their intervals. Chromosomes 2, and 4 harbored relatively all of the QTLs on their non-telomeric regions (Figs. 2, 3b). A large number of QTLs were projected over the sub-telomeric region of chromosomes 1, 3, and 5 (Figs. 2, 3b). Similarly, the same pattern was observed for MQTLs distribution over the genetic consensus map. Chromosomes 2 and 4 possessed the majority of MQTLs in their non-telomeric intervals and chromosomes 1, 3, and 5 harbored several MQTLs in their sub-telomeric regions (Figs. 2, 3c). In contrast, the result of MQTLs distribution over *A. thaliana* physical map indicated a relatively high number of MQTLs in sub-telomeric regions (Fig. 3d).

The lowest number of QTLs and MQTLs were detected at the centromeric intervals for the ionomic traits (Figs. 2, 3). The MQTL-1/Chr2, MQTL-5/Chr3, and MQTL-5/Chr5 were located near the centromeric region of chromosomes 2, 3, and 5 of the physical and genetic consensus maps, respectively (Figs. 2, 3c,d). Furthermore, there was a significant correlation between the number of QTLs and MQTLs over the *A. thaliana* chromosomes (r = 0.97, P = 0.007) (Fig. 3a). The gene density over *A. thaliana* chromosomes showed a similar pattern with QTLs and MQTLs distributions (Fig. 4).

### Functional candidate genes within MQTL intervals

The number of identified candidate genes in the detected MQTL intervals are reported in Table 3. All the annotated genes located in each MQTL interval and the functions of the potential candidate are presented in Supplementary Table S1. The highest number of candidate genes in the identified meta-QTLs was observed in MQTL-2/Chr4 (774 genes) followed by MQTL-4/Chr2 and MQTL-2/Chr5 with 693 and 692 gene number, respectively (Table 3). The lowest number of candidate genes belongs to MQTL-5/Chr3 with nine genes.

Several well-known candidate genes including bHLH-encoding genes family (e.g. *bHLH34* (*At3g23210*)), *WRKY* (e.g. *WRKY12* (*At2g44745*), *WRKY13* (*At4g39410*)), *MHX* (*At2g47600*), *NRAMP3* (*At2g23150*), *ZIP4* (*At5g48390*), *MTP11* (*At2g39450*), *IRT3* (*At1g60960*), and *FIT* (*At2g28160*) were identified in the MQTLs regions of our study. The CGs have shown diverse functions for micronutrients transportation and homeostasis (Supplementary Table S1).

### Trait analysis within MQTLs

The results of trait –MQTL analysis showed the unequal presence of individual QTLs across the confined MQTLs. Individual QTLs for potassium (K) were present in 26 of the 33 MQTL regions (78.8%), the most for any ionomic trait, followed by Zn (23 MQTL), P (22 MQTL), Mn (21 MQTL), Mg (20 MQTL) and Ca (20 MQTL). The Fe was presented in 17 MQTL regions. The meta-analysis was able to confirm at least one QTL for all assessed ionomic traits in the detected MQTLs intervals (Table 4).

**Table 4:** Co-localization frequency analysis of the QTLs for ionomic traits, and Fe, Zn and Mn contents in grain in the detected MQTL regions in the *Arabidopsis thaliana* genome.

Trait	Number of MQTL^a^	Co-localization frequency with grain Zn	χ^2^	P value	Co-localization frequency with grain Fe	χ^2^	P value	Co-localization frequency with grain Mn	χ^2^	P value
shAl	3	0.087	0.257	0.612^n.s^	0.176	1.329	0.249^n.s^	0.143	0.608	0.436^n.s^
As	1	0.043	0.129	0.720^n.s^	0.059	0.443	0.506^n.s^	0.000	–	–
B	10	0.304	0.900	0.343^n.s^	0.353	2.658	0.103^n.s^	0.333	1.418	0.234^n.s^
Ca	20	0.652	1.929	0.165^n.s^	0.588	4.431	0.035*	0.619	2.633	0.105^n.s^
rsCa	4	0.130	0.386	0.535^n.s^	0.176	1.329	0.249^n.s^	0.143	0.608	0.436^n.s^
shCa	5	0.174	0.514	0.473^n.s^	0.235	1.772	0.183^n.s^	0.190	0.810	0.368^n.s^
Cd	4	0.087	0.257	0.612^n.s^	0.059	0.443	0.506^n.s^	0.143	0.608	0.436^n.s^
Co	7	0.217	0.643	0.423^n.s^	0.235	1.772	0.183^n.s^	0.190	0.810	0.368^n.s^
Cu	15	0.522	1.543	0.214^n.s^	0.353	2.658	0.103^n.s^	0.476	2.025	0.155^n.s^
shCu	3	0.130	0.386	0.535^n.s^	0.118	0.886	0.347^n.s^	0.095	0.405	0.525^n.s^
Fe	17	0.435	1.286	0.257^n.s^	–	–	–	0.571	2.430	0.119^n.s^
roFe	1	0.043	0.129	0.720^n.s^	0.000	–	–	0.048	0.203	0.653^n.s^
rsFe	5	0.174	0.514	0.473^n.s^	0.176	1.329	0.249^n.s^	0.143	0.608	0.436^n.s^
shFe	3	0.130	0.386	0.535^n.s^	0.118	0.886	0.347^n.s^	0.143	0.608	0.436^n.s^
K	26	0.783	2.314	0.128^n.s^	0.882	6.646	0.010*	0.762	3.240	0.072^n.s^
rsK	4	0.130	0.386	0.535^n.s^	0.118	0.886	0.347^n.s^	0.095	0.405	0.525^n.s^
shK	2	0.043	0.129	0.720^n.s^	0.118	0.886	0.347^n.s^	0.095	0.405	0.525^n.s^
Li	5	0.174	0.514	0.473^n.s^	0.059	0.443	0.506^n.s^	0.143	0.608	0.436^n.s^
Mg	20	0.609	1.800	0.180^n.s^	0.647	4.874	0.027*	0.667	2.835	0.092^n.s^
rsMg	6	0.261	0.771	0.380^n.s^	0.118	0.886	0.347^n.s^	0.238	1.013	0.314^n.s^
shMg	4	0.174	0.514	0.473^n.s^	0.176	1.329	0.249^n.s^	0.095	0.405	0.525^n.s^
Mn	21	0.652	1.929	0.165^n.s^	0.706	5.317	0.021*	–	–	–
rsMn	8	0.217	0.643	0.423^n.s^	0.294	2.215	0.137^n.s^	0.286	1.215	0.270^n.s^
shMn	2	0.043	0.129	0.720^n.s^	0.059	0.443	0.506^n.s^	0.095	0.405	0.525^n.s^
Mo	4	0.174	0.514	0.473^n.s^	0.059	0.443	0.506^n.s^	0.143	0.608	0.436^n.s^
Na	12	0.348	1.029	0.310^n.s^	0.353	2.658	0.103^n.s^	0.476	2.025	0.155^n.s^
Ni	2	0.043	0.129	0.720^n.s^	0.059	0.443	0.506^n.s^	0.000	–	–
P	22	0.783	2.314	0.128^n.s^	0.706	5.317	0.021*	0.762	3.240	0.072^n.s^
rsP	7	0.217	0.643	0.423^n.s^	0.235	1.772	0.183^n.s^	0.238	1.013	0.314^n.s^
shP	6	0.217	0.643	0.423^n.s^	0.176	1.329	0.249^n.s^	0.238	1.013	0.314^n.s^
Rb	6	0.174	0.514	0.473^n.s^	0.235	1.772	0.183^n.s^	0.048	0.203	0.653^n.s^
S	15	0.478	1.414	0.234^n.s^	0.588	4.431	0.035*	0.476	2.025	0.155^n.s^
shS	4	0.130	0.386	0.535^n.s^	0.118	0.886	0.347^n.s^	0.095	0.405	0.525^n.s^
Se	8	0.304	0.900	0.343^n.s^	0.235	1.772	0.183^n.s^	0.190	0.810	0.368^n.s^
Zn	23	–	–	–	0.588	4.431	0.035*	0.714	3.038	0.081^n.s^
roZn	1	0.043	0.129	0.720^n.s^	0.059	0.443	0.506^n.s^	0.000	–	–
rsZn	9	0.304	0.900	0.343^n.s^	0.353	2.658	0.103^n.s^	0.381	1.620	0.203^n.s^
shZn	2	0.087	0.257	0.612^n.s^	0.118	0.886	0.347^n.s^	0.095	0.405	0.525^n.s^

^n.s^Non-significant.

*Significant at the 0.05 probability level.

^a^Number of discovered MQTLs containing each ionomic trait.

The association between the individual ionomic QTLs and target QTLs (i.e. Zn, Fe, and Mn) was assessed by analyzing the co-localization frequency. There was 65.2% and 43.5% co-localization frequency between QTLs for Fe and Mn with Zn QTLs, 58.8% and 70.6% co-localization between Zn and Mn QTLs with Fe QTLs. The Zn and Fe QTLs represent 71.4% and 57.1% co-localization with Mn QTLs, respectively (Table 4). Among the identified MQTLs, the MQTL-1/Chr1, MQTL-3/Chr2, MQTL-1/Chr3, MQTL-3/Chr3, MQTL-5/Chr3, and MQTL-3/Chr5 harbored all Zn, Fe, and Mn QTLs. Besides, the highest QTLs number for Zn (5 QTL) was located on MQTL-3/Chr5 whilst the MQTL-3/Chr2 includes the highest QTLs number for Fe (5) and Mn (4) QTLs. Association between ionomic QTLs with Zn and Mn QTLs did not differ from the expectations. However, the association between ionomic (especially Mn and Zn QTLs) and Fe QTLs was statistically significant suggesting their association was more frequent than expected (Table 4).

### Comparison MQTLs and GWAS results

Location of the identified MQTLs was compared with the SNPs linked to Zn, Fe, and Mn traits in a GWAS study for *A. thaliana*^43^. This comparison led to the identification of nine highly associated loci for Zn, Fe, and Mn elements that illustrated more reliable MQTLs. In fact, two, three, and four highly associated loci from the GWAS study were co-located with our identified MQTLs for Zn, Fe, and Mn, respectively (Table 5). These loci were distributed on chromosomes 3 and 4. In addition, 104 common genes between GWAS study and the current MQTL analysis were located in the MQTL-3/Chr1 (11 genes), MQTL-3/Chr3 (16 genes), MQTL-4/Chr3 (10 genes), MQTL-5/Chr3 (2 genes), MQTL-2/Chr4 (14 genes), MQTL-3/Chr4 (19 genes), MQTL-4/CHr4 (9 genes), MQTL-2/Chr5 (12 genes) and MQTL-7/Chr5 (11 genes) intervals. Among the detected common genes between GWAS study and the current MQTL analysis, the well-known *At4g08170* gene annotated as *ITPK3* was located in the MQTL-2/Chr4 interval (Supplementary Table S2).

**Table 5:** Meta-QTLs (MQTLs) collinear with the highly associated SNPs for micronutrients in *Arabidopsis thaliana* GWAS study.

Trait	*A. thaliana* MQTL	Chr. no	Genomic position (Mb)	Genomic position of highly associated SNPs (Mb)	*A. thaliana* GWAS
Zn	MQTL-3/Chr3	3	4.95–5.57	5.325	^40^
	MQTL-5/Chr3	3	14.53–14.72	12.43	
Fe	MQTL-3/Chr3	3	4.95–5.57	5.290	
				5.300	
	MQTL-5/Chr3	3	14.53–14.72	14.59	
Mn	MQTL-4/Chr3	3	8.17–8.81	8.478	
				8.479	
	MQTL-3/Chr4	4	6.25–6.62	6.473	
				6.553	

## Discussion

The construction of a new consensus genetic map is a prerequisite for the projection of QTLs in the QTL database and running meta-QTL analysis. The results of the current MQTL analysis suggested that the MQTL-3/Chr2, MQTL-7/Chr1, MQTL-1/Chr3, and MQTL-3/Chr5 with the highest number of initial QTLs identified in independent populations were the most viable, stable, and robust QTLs under different experimental conditions. Identification of meta-QTLs significantly increases the power and precision of our ability to map important traits that will provide greater precision for future fine mapping and marker development^48^. In the current MQTL analysis, the average confidence interval (CI) for QTLs of ionomic traits reduced up to eight folds compared to the mean CI of the original QTLs. The ability of the MQTL analysis methods to reduce the QTL confidence interval by taking advantage of pooling QTLs helps to increase the resolution of selected candidate genes^22^ and consequently increases the precision of detecting functional candidate genes. Two complementary approaches for genetic mapping, linkage mapping (QTL mapping) and association mapping (LD mapping) have led to the successful dissection of complex traits in many plant species by detecting QTLs and identifying marker-trait associations^49^. Often, the genetic and environmental background, number of traits, phenotypic variation, marker density, and map regulation are the most limiting factors in linkage mapping approaches. However, the meta-QTL analysis helps to identify the most stable QTLs regardless of limitations in traditional QTL mapping studies^49^. The LD mapping uncovers genomic regions underlying quantitative traits with higher accuracy compared to the linkage mapping that is particularly efficient in species with low linkage disequilibrium (LD)^50,51^. Besides, the population structure of the studied panel in LD mapping can lead to false-positive association discovery^52,53^. Despite the limitations of QTL- and LD-mapping methods, the statistical power to detect QTLs through the use of these approaches is particularly affected by the sample size^54^. To overcome these limitations, meta- analysis has been used to combine results from multiple studies and increase the power of genetic mapping^49^. The QTL meta-analysis method is a horizontal way to integrate information for the same trait from different experiments and populations. Thus, a meta-analysis allows for comparative genomics and is a valuable tool to complement information obtained from vertical integration^55^.

Our meta-QTL analysis over the *A. thaliana* chromosomes revealed that chromosomes 4 and 2 possessed a low number of QTLs and MQTLs, which could be attributed to fewer markers on these chromosomes. This result was in line with the results of the Serin et al.^56^ study. Our results revealed a similar pattern for the distribution of QTLs and MQTLs over the *A. thaliana* chromosomes based on the genetic map used. Besides, the projection of QTLs and MQTLs across *A. thaliana* genome was in accordance with the constructed gene density map. It has been shown that QTLs result from the genetic segregation of sequence polymorphisms at functional elements such as regulatory sequences upstream of genes and/or coding sequences^57,58^. Therefore, it is expected that QTL density is related to the gene density, polymorphism rate at functional sites in genic regions and the frequency of recombination in the genome^32^. These outcomes showed for the first time that majority of QTLs and MQTLs controlling ionomic traits are located in the non-telomeric regions of the *A. thaliana* genetic map. However, higher QTL density at the sub-telomeric regions was apparent when physical distances were considered for meta-QTL analysis (Figs. 2, 3)^32^. Particularly, the distribution of MQTLs was higher in the gene dense regions located in non-telomeric and sub-telomeric regions in the *A. thaliana* genome. This result was in accordance with the result of the Martinez et al.^32^ study showing QTLs appeared to more densely map on non-telomeric regions of genetic maps and sub-telomeric regions of *A. thaliana* physical map.

Identifying genes for ionomic traits control is a fundamental step for better understanding of ionome transportation, accumulation and mineral element changes in plants. Results of our meta-QTL analysis for ionomic traits showed that several important genes identified in the MQTLs were associated with micronutrient homeostasis. A better understanding of responsible genes involved in micronutrient homeostasis in plants is among the most important proceedings for breeding programs to combat “micronutrient malnutrition”^6^. A useful gene can be used to target multiple crops through genomic engineering approaches. The *bHLH*-encoding genes family including *bHLH34* (8283003–8285081 bp) was identified in the MQTL-4/Chr3 of our meta-QTL study. Several members of bHLH-encoding genes are associated with the Fe homeostasis control^59^. The *bHLH34* belonging to the clade IVc involved in *A. thaliana* Fe deficiency response^59^. The MQTL-2/Chr2 contains the *NRAMP3* (9856149–9858778 bp) gene that is associated with iron deficiency in both roots and aerial parts of *A. thaliana* and localize to the vacuolar membrane, indicating its contribution to intracellular metal homeostasis^60–62^. The MQTL-3/Chr2 harbored another important gene, *FIT* (12004658–12006276 bp), which plays a predominant role in the regulation of iron mobilization^63–66^. The role of *FIT* in Fe acquisition had been documented^67^. The *ZIP4* (19612020–19615383 bp) and *IRT3* (22445299–22447299 bp) genes that located in the MQTL-7/Chr5 and MQTL-7/Chr1 intervals of our study are involved in Zn, Fe, Mn, and Cd transport^68^. Further, ZIP proteins that express in shoot tissues contribute to Zn uptake from the soil translocation of Zn throughout the plant^69,70^. In *A. thaliana*, the *ZIP4* is highly expressed under zinc deficiency conditions^68,71^. The expression of *AtIRT3* can respond to Zn^72^, which implies a possible role for *IRT3* in Zn transport and homeostasis^73^. Overexpressing of *AtIRT3* in *A. thaliana* increases accumulation of Zn in the shoot and Fe in the root of transgenic lines suggesting the role of *IRT3* as a Zn and Fe‐uptake transporter^73^. Detection of the *MTP11* (16471560–16474060 bp) gene in the MQTL-5/Chr2 intervals in the current meta-analysis provides an evidence for Mn^2+^-specific transport activity of *AtMTP11* that implicate the pre-vacuolar compartments in both Mn^2+^ tolerance and Mn^2+^ homeostasis mechanisms^74^. Our meta-analysis confirmed the presence of *WRKY12* (18447203–18449093 bp) and *WRKY13* (18332606–18334893 bp) genes located in the MQTL-4/Chr2 and MQTL-5/Chr4 intervals, respectively. The *AtWRKY12*^75^ gene regulates cadmium (Cd) accumulation and *AtWRKY13*^76^ enhances tolerance to Cd. *MHX* (19524117–19527580 bp) located in the identified MQTL-4/Chr2 was another identified gene associated with concentration and transportation of Mg^14,77^. Electrophysiological analysis and overexpression studies have shown that the *A. thaliana MHX* gene encodes Mg^2+^/H^+^ exchanger, which exchange protons with Mg^2+^, Zn^2+^, Cd^2+^, and possibly Fe^2+^ ions across the vacuolar membrane^78,79^.

The results of our meta-QTL analysis suggested that QTLs for K, Zn, P, Mn, Mg, and Ca were the most frequent distributed QTLs in the detected MQTLs regions that could be due to high heritability and the role of these ions in physiological pathways^80–83^. Our MQTL-analysis revealed co-localization between QTLs for Zn, Mn, and Fe ions. The co-localization of QTLs may reflect the correlations observed between the ionomic traits. Based on *A. thaliana* QTL analysis, different ionomic traits demonstrated significant correlation which most robust was the correlations between Zn, Mn, and Fe traits^16,42^. The result of co-localization frequency of ionomic QTLs on detected MQTLs regions in this study revealed the possible signs of MQTLs interaction, which impacts the association of ionomic traits at the genomic level. Co-localized QTLs or pleiotropic genes are of great significance for breeders and geneticists, for assessing the effects of selective genetic improvement programs, or for understanding pathways of genetic activity^84^. For biofortification, especially with Zn and Fe, large-effect and consistent QTLs are of particular importance and merit further examination^85^. Furthermore, the co-localization of QTLs of different traits in the same chromosomal regions suggests the existence of physiological and/or genetic relationships between traits^86^. Thus, the co-localized QTLs are important for breeding programs, QTL pyramiding, and simultaneous improvement of several ionomic traits.

Association analysis helps to unravel the genetic control of quantitative complex traits and validation of their corresponding genes in independent backgrounds^87,88^. In our study, four MQTLs (MQTL-3/Chr3, MQTL-4/Chr3, MQTL-3/Chr5, and MQTL-3/Chr4) were collinear with nine highly associated SNPs responsible for Zn, Fe, and Mn quantitative traits. Further, the *ITPK3* (5163220–5167192 bp) gene, which involved in micronutrients chelating and accumulation, was among the identified functional candidate genes detected through the current MQTL analyses and the GWAS study^47,89^. The ability of the MQTL analysis method to validate identified loci/QTLs using GWAS have been previously documented^27–29^. Results of our meta-QTL analysis represent the coherence and consistency of MQTL analyses and GWAS for the identification of genomic regions corresponding to the studied ionomic traits.

## Conclusions

To our knowledge, this is the first MQTL analysis report that identified several major genomic regions associated with ionomic traits in *A. thaliana*. This analysis defines a genome-wide landscape on the most stable genomic regions (MQTL-3/Chr2, MQTL-7/Chr1, MQTL-1/Chr3, and MQTL-3/Chr5) along with reliable genetic markers (CH.192L-Col, HH.357L, AD.191L-Col, SNP135, SNP105, DF.252L, m291, and BH.127L) that provide a robust tool for breeding ionomic traits through marker-assisted selection. Measuring trace elements in large sample size population is costly for breeders and identifying linked DNA markers accelerates pre-breeding and variety screening for biofortification programs and improving quality traits in plants. Results of our study suggested that the genomic positions of QTLs and MQTLs were mainly located in non-telomeric regions based on the newly constructed genetic consensus map, whilst the physical map revealed the projection of higher MQTLs in sub-telomeric regions. This was in line with the results of gene density in sub-telomeric and sub-centromeric regions of the *Arabidopsis* chromosomes. The results of the analysis for co-localization frequency revealed co-map QTLs controlling ionomic traits suggesting the existence of physiological and/or genetic relationships between these traits which lead to possible improvement of multiple ionome traits simultaneously. Further, the current study demonstrates that meta-QTL analysis method is cheaper than, and as informative as GWAS and then is powerful enough to provide greater resolution for future fine mapping without GWAS. Overall, the identified candidate genes at the detected MQTLs regions will provide a better understanding of ionomic variation in the *A. thaliana* genome. Besides their function can be generalized to other plant species which provides the raw material for breeders in various breeding programs such as biofortification.

## Supplementary Information

Supplementary Information.

## Data Availability

The datasets generated during and/or analyzed during the current study are available from the corresponding author on reasonable request.
