# Supplementary material for: Genomic analysis of ionome-related QTLs in Arabidopsis thaliana
Source: Sci Rep. 2021 Sep 28;11:19194. doi: 10.1038/s41598-021-98592-7 (PMC8479127; doi:10.1038/s41598-021-98592-7)
Supplement: Supplementary file 1 — Supplementary Information. [file 41598_2021_98592_MOESM1_ESM.pdf]

Genomic analysis of ionome-related QTLs in *Arabidopsis thaliana*

Nikwan Shariatipour[1], Bahram Heidari[*1], Samathmika Ravi[2], Piergiorgio Stevanato[2]

[1] Department of Plant Production and Genetics, School of Agriculture, Shiraz University, Shiraz, 7144165186, Iran

[2] Department of Agronomy, Animals, Natural Resources and Environment- DAFNAE, University of Padova, Legnaro (Padova), Italy

ORCID

Nikwan Shariatipour – http://orcid.org/0000-0003-4174-4375.

Bahram Heidari – https://orcid.org/0000-0002-5856-4592.

Samathmika Ravi – https://orcid.org/0000-0002-6259-492X

Piergiorgio Stevanato – https://orcid.org/0000-0002-6577-6956.

***Corresponding Author:** Bahram Heidari.

Email: bheidari@shirazu.ac.ir.

Phone: +987136138176.

Fax: +987132276907.

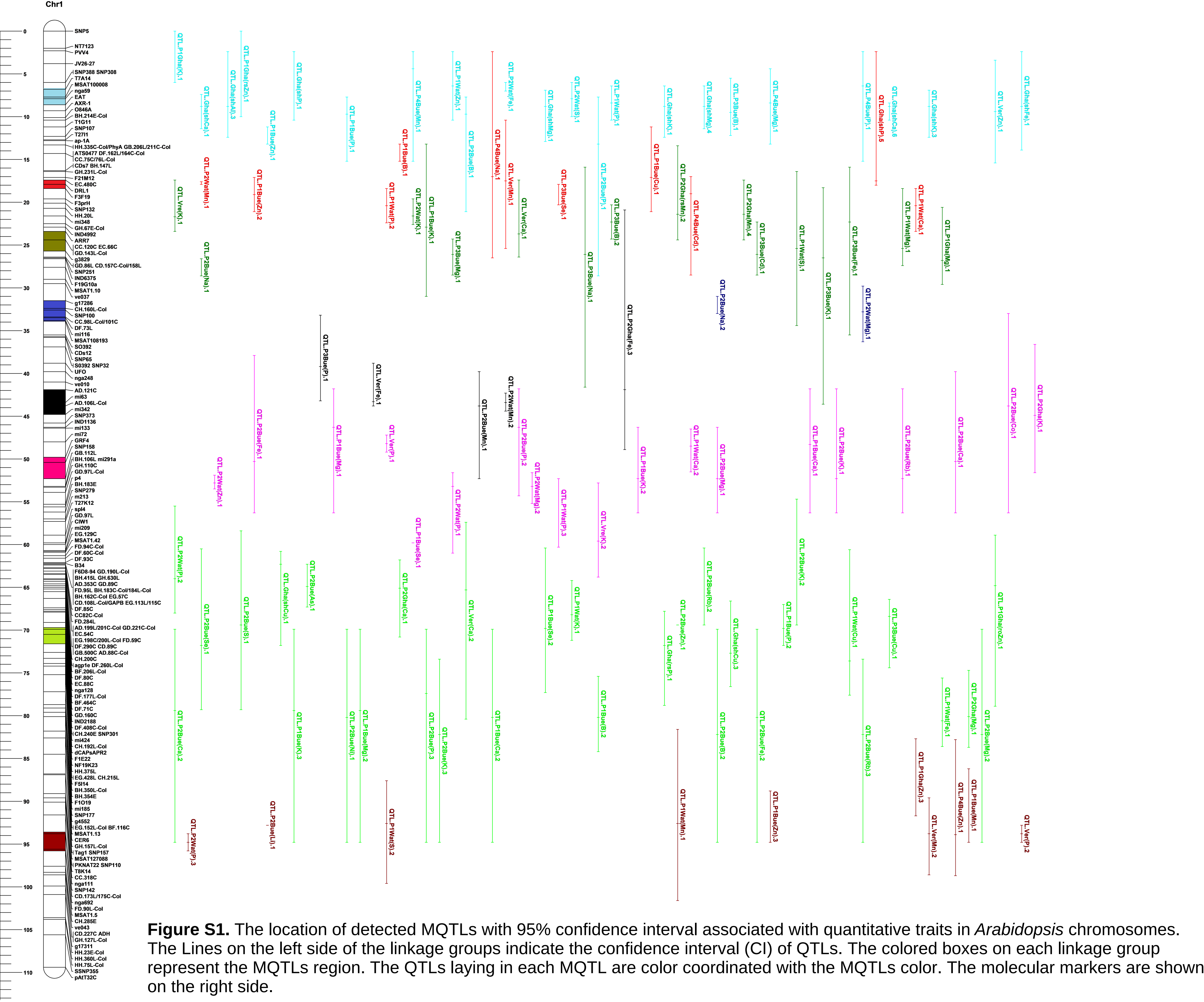

Figure S1. The location of detected MQTLs with 95% confidence interval associated with quantitative traits in *Arabidopsis* chromosomes. The Lines on the left side of the linkage groups indicate the confidence interval (CI) of QTLs. The colored boxes on each linkage group represent the MQTLs region. The QTLs laying in each MQTL are color coordinated with the MQTLs color. The molecular markers are shown on the right side.

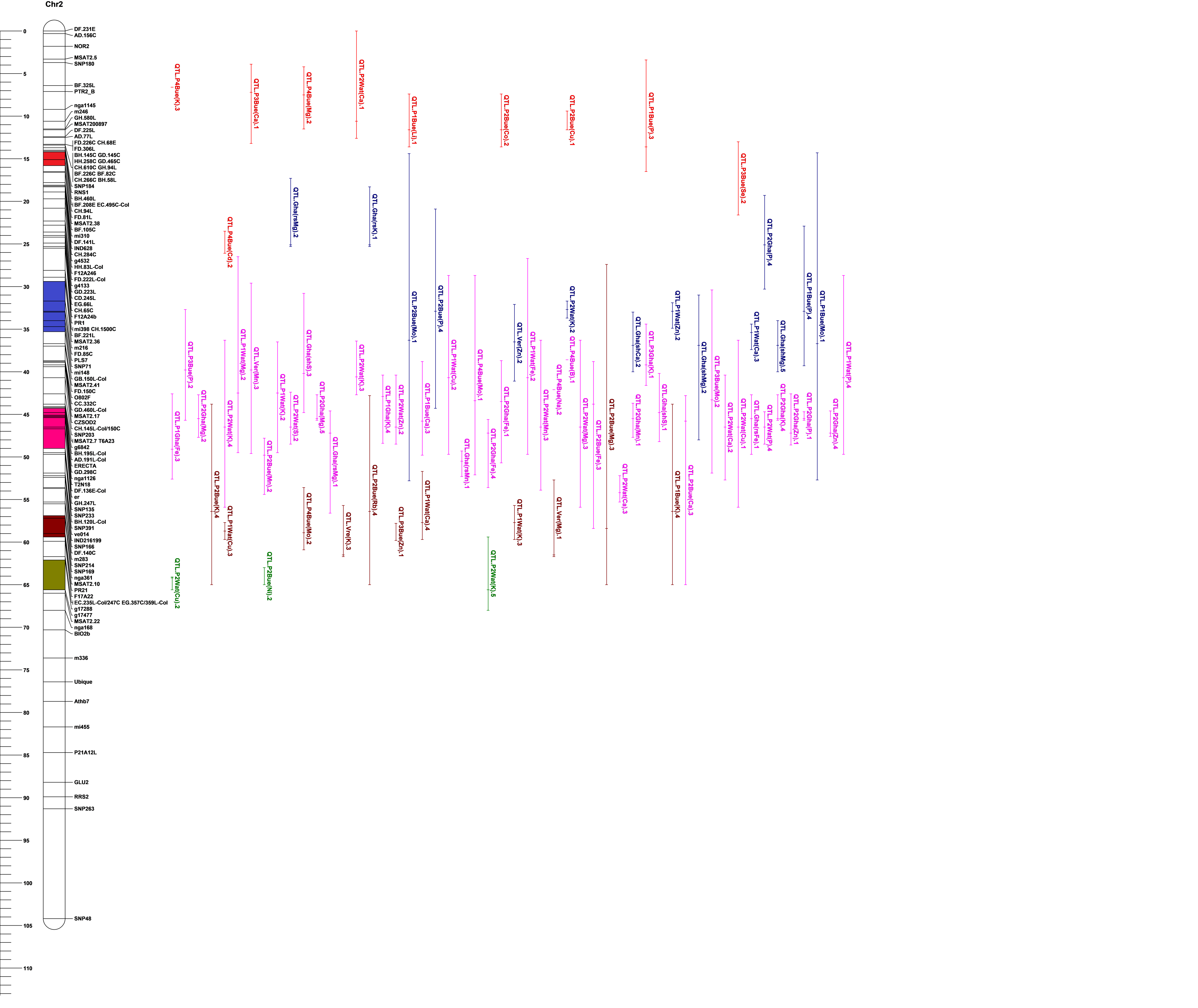

Chr2
0
5
10
15
20
25
30
35
40
45
50
55
60
65
70
75
80
85
90
95
100
105
110
DF.231E
AD.156C
NOR2
MSAT2.5
SNP180
BF.325L
PTR2_B
nga1145
m246
GH.580L
MSAT200897
DF.225L
AD.77L
FD.226C CH.68E
FD.306L
BH.145C GD.145C
HH.258C GD.465C
CH.610C GH.94L
BF.226C BF.82C
CH.266C BH.58L
SNP184
RNS1
BH.460L
BF.208E EC.495C-Col
CH.94L
FD.81L
MSAT2.38
BF.105C
mi310
DF.141L
IND628
CH.284C
g4532
HH.83L-Col
F12A246
FD.222L-Col
g4133
GD.223L
CD.245L
EG.66L
CH.65C
F12A24b
PR1
mi398 CH.1500C
BF.221L
MSAT2.36
m216
FD.85C
PLS7
SNP71
mi148
GB.150L-Col
MSAT2.41
FD.150C
O802F
CC.332C
GD.460L-Col
MSAT2.17
CZSOD2
CH.145L-Col/150C
SNP203
MSAT2.7 T6A23
g6842
BH.195L-Col
AD.191L-Col
ERECTA
GD.298C
nga1126
T2N18
DF.136E-Col
er
GH.247L
SNP135
SNP233
BH.120L-Col
SNP391
ve014
IND216199
SNP166
DF.140C
m283
SNP214
SNP169
nga361
MSAT2.10
PR21
F17A22
EC.235L-Col/247C EG.357C/359L-Col
g17288
g17477
MSAT2.22
nga168
BIO2b
m336
Ubique
Athb7
mi455
P21A12L
GLU2
RRS2
SNP263
SNP48
QTL.P4Bue(K).3
QTL.P3Bue(Ca).1
QTL.P4Bue(Mg).2
QTL.P2Wat(Ca).1
QTL.P1Bue(Li).1
QTL.P2Bue(Co).2
QTL.P2Bue(Cu).1
QTL.P1Bue(P).3
QTL.P3Bue(Se).2
QTL.Gha(rsMg).2
QTL.Gha(rsK).1
QTL.P4Bue(Cd).2
QTL.P2Bue(Mo).1
QTL.P2Bue(P).4
QTL.P2Gha(P).4
QTL.P1Bue(P).4
QTL.P1Bue(Mo).1
QTL.P2Wat(K).2
QTL.P1Wat(Zn).2
QTL.Gha(shCa).2
QTL.P3Gha(K).1
QTL.P1Wat(Ca).3
QTL.Gha(shMg).5
QTL.Gha(shMg).2
QTL.P3Bue(P).2
QTL.P1Gha(Fe).3
QTL.P2Gha(Mg).2
QTL.P1Wat(Mg).2
QTL.Ver(Mn).3
QTL.P2Wat(K).4
QTL.P1Wat(K).2
QTL.P2Wat(S).2
QTL.Gha(shS).3
QTL.P2Gha(Mg).5
QTL.P2Bue(Mn).2
QTL.Gha(rsMg).1
QTL.P2Wat(K).3
QTL.P1Gha(K).4
QTL.P2Wat(Zn).2
QTL.P1Bue(Ca).3
QTL.P1Wat(Cu).2
QTL.P4Bue(Mo).1
QTL.Gha(rsMn).1
QTL.P2Gha(Fe).1
QTL.P2Gha(Fe).4
QTL.Ver(Zn).2
QTL.P1Wat(Fe).2
QTL.P2Wat(Mn).3
QTL.P4Bue(Na).2
QTL.P4Bue(B).1
QTL.P2Wat(Mg).3
QTL.P2Bue(Fe).3
QTL.P2Bue(Mg).3
QTL.P2Gha(Mn).1
QTL.Gha(shS).1
QTL.P2Wat(Ca).3
QTL.P3Bue(Mo).2
QTL.P2Wat(Ca).2
QTL.P2Wat(Cu).1
QTL.Gha(rsFe).1
QTL.P2Wat(P).4
QTL.P2Gha(K).4
QTL.P2Gha(Zn).1
QTL.P3Gha(P).1
QTL.P2Gha(Zn).4
QTL.P1Wat(P).4
QTL.P2Bue(K).4
QTL.P1Wat(Cu).3
QTL.P4Bue(Mo).2
QTL.Vre(K).3
QTL.P2Bue(Rb).4
QTL.P3Bue(Zn).1
QTL.P1Wat(Ca).4
QTL.P1Wat(K).3
QTL.Ver(Mg).1
QTL.P1Bue(K).4
QTL.P2Bue(Ca).3
QTL.P2Wat(Cu).2
QTL.P2Bue(Ni).2
QTL.P2Wat(K).5

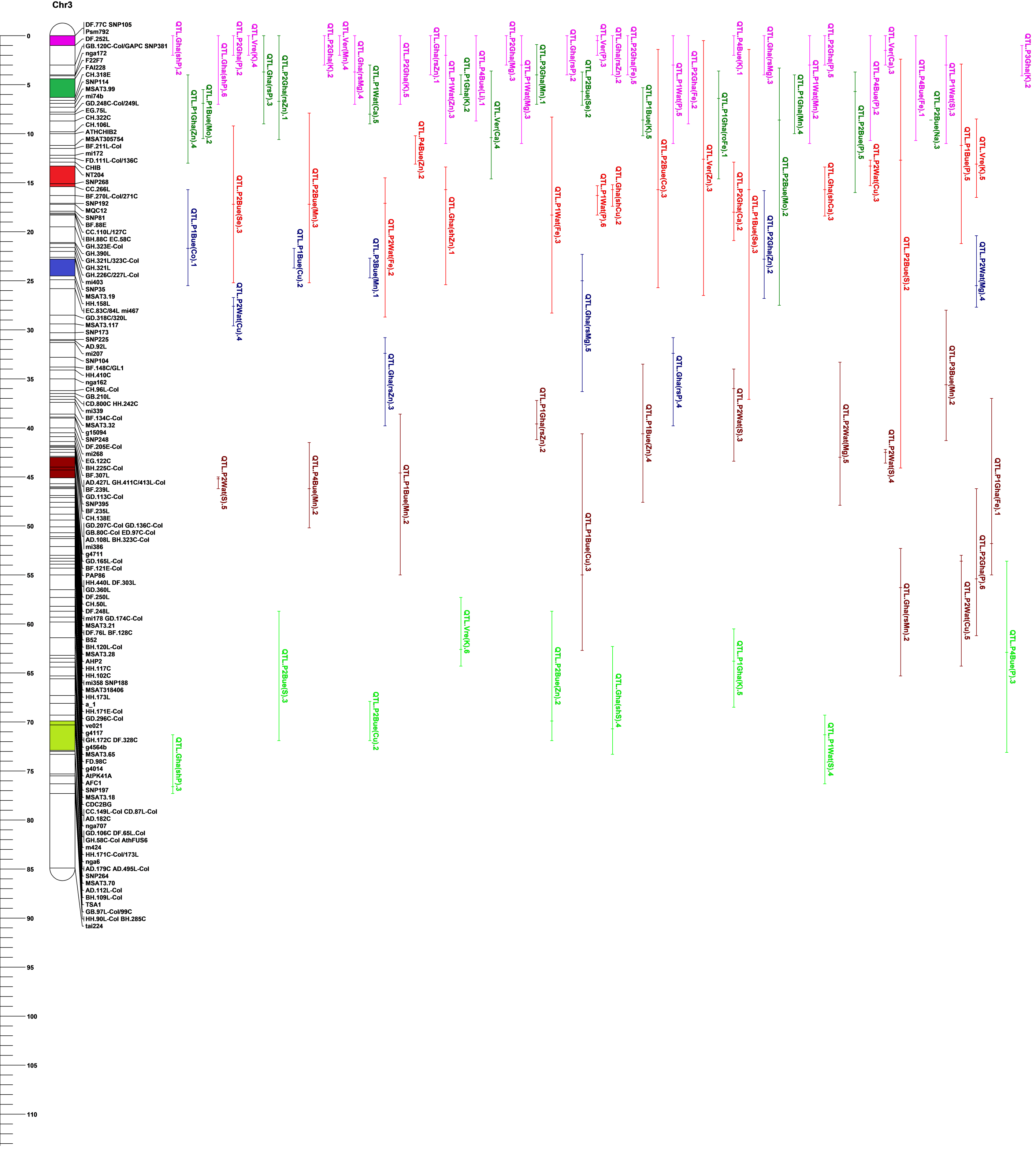

Chr3
DF.77C SNP105
Psm792
DF.252L
GB.120C-Col/GAPC SNP381
nga172
F22F7
FAI228
CH.318E
SNP114
MSAT3.99
mi74b
GD.248C-Col/249L
EG.75L
CH.322C
CH.106L
ATHCHIB2
MSAT305754
BF.211L-Col
mi172
FD.111L-Col/136C
CHIB
NT204
SNP268
CC.266L
BF.270L-Col/271C
SNP192
MQC12
SNP81
BF.88E
CC.110L/127C
BH.88C EC.58C
GH.323E-Col
GH.390L
GH.321L/323C-Col
GH.321L
GH.226C/227L-Col
mi403
SNP35
MSAT3.19
HH.158L
EC.83C/84L mi467
GD.318C/320L
MSAT3.117
SNP173
SNP225
AD.92L
mi207
SNP104
BF.148C/GL1
HH.410C
nga162
CH.96L-Col
GB.210L
CD.800C HH.242C
mi339
BF.134C-Col
MSAT3.32
g15094
SNP248
DF.205E-Col
mi268
EG.122C
BH.225C-Col
BF.307L
AD.427L GH.411C/413L-Col
BF.239L
GD.113C-Col
SNP395
BF.235L
CH.138E
GD.207C-Col GD.136C-Col
GB.80C-Col ED.97C-Col
AD.108L BH.323C-Col
mi386
g4711
GD.165L-Col
BF.121E-Col
PAP86
HH.440L DF.303L
GD.360L
DF.250L
CH.50L
DF.248L
mi178 GD.174C-Col
MSAT3.21
DF.76L BF.128C
B52
BH.120L-Col
MSAT3.28
AHP2
HH.117C
HH.102C
mi358 SNP188
MSAT318406
HH.173L
a_1
HH.171E-Col
GD.296C-Col
ve021
g4117
GH.172C DF.328C
g4564b
MSAT3.65
FD.98C
g4014
AtPK41A
AFC1
SNP197
MSAT3.18
CDC2BG
CC.149L-Col CD.87L-Col
AD.182C
nga707
GD.106C DF.65L.Col
GH.58C-Col AthFUS6
m424
HH.171C-Col/173L
nga6
AD.179C AD.495L-Col
SNP264
MSAT3.70
AD.112L-Col
BH.109L-Col
TSA1
GB.97L-Col/99C
HH.90L-Col BH.285C
tai224
0
5
10
15
20
25
30
35
40
45
50
55
60
65
70
75
80
85
90
95
100
105
110

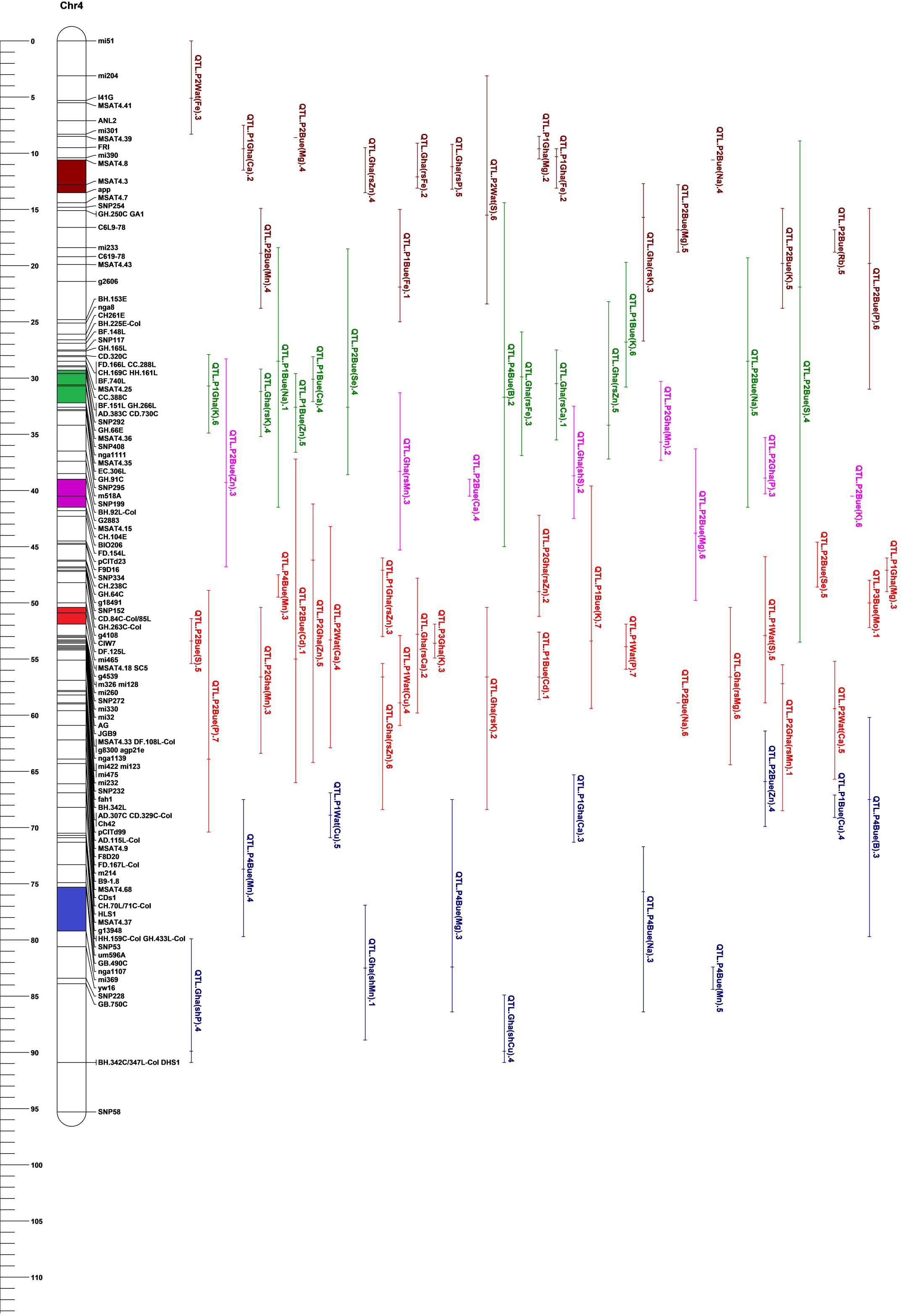
Chr4
0
5
10
15
20
25
30
35
40
45
50
55
60
65
70
75
80
85
90
95
100
105
110
mi51
mi204
I41G
MSAT4.41
ANL2
mi301
MSAT4.39
FRI
mi390
MSAT4.8
MSAT4.3
app
MSAT4.7
SNP254
GH.250C GA1
C6L9-78
mi233
C619-78
MSAT4.43
g2606
BH.153E
nga8
CH261E
BH.225E-Col
BF.148L
SNP117
GH.165L
CD.320C
FD.166L CC.288L
CH.169C HH.161L
BF.740L
MSAT4.25
CC.388C
BF.151L GH.266L
AD.383C CD.730C
SNP292
GH.66E
MSAT4.36
SNP408
nga1111
MSAT4.35
EC.306L
GH.91C
SNP295
m518A
SNP199
BH.92L-Col
G2883
MSAT4.15
CH.104E
BIO206
FD.154L
pCITd23
F9D16
SNP334
CH.238C
GH.64C
g18491
SNP152
CD.84C-Col/85L
GH.263C-Col
g4108
CIW7
DF.125L
mi465
MSAT4.18 SC5
g4539
m326 mi128
mi260
SNP272
mi330
mi32
AG
JGB9
MSAT4.33 DF.108L-Col
g8300 agp21e
nga1139
mi422 mi123
mi475
mi232
SNP232
fah1
BH.342L
AD.307C CD.329C-Col
Ch42
pCITd99
AD.115L-Col
MSAT4.9
F8D20
FD.167L-Col
m214
B9-1.8
MSAT4.68
CDs1
CH.70L/71C-Col
HLS1
MSAT4.37
g13948
HH.159C-Col GH.433L-Col
SNP53
um596A
GB.490C
nga1107
mi369
yw16
SNP228
GB.750C
BH.342C/347L-Col DHS1
SNP58
QTL.P2Wat(Fe).3
QTL.P1Gha(Ca).2
QTL.P2Bue(Mg).4
QTL.Gha(rsZn).4
QTL.Gha(rsFe).2
QTL.Gha(rsP).5
QTL.P2Wat(S).6
QTL.P1Gha(Mg).2
QTL.P1Gha(Fe).2
QTL.P2Bue(Na).4
QTL.P2Bue(Mg).5
QTL.Gha(rsK).3
QTL.P2Bue(K).5
QTL.P2Bue(Rb).5
QTL.P2Bue(P).6
QTL.P2Bue(Mn).4
QTL.P1Bue(Fe).1
QTL.P1Bue(Na).1
QTL.P2Bue(Se).4
QTL.P1Gha(K).6
QTL.Gha(rsK).4
QTL.P1Bue(Ca).4
QTL.P1Bue(Zn).5
QTL.P4Bue(B).2
QTL.Gha(rsFe).3
QTL.Gha(rsCa).1
QTL.Gha(rsZn).5
QTL.P1Bue(K).6
QTL.P2Bue(Na).5
QTL.P2Bue(S).4
QTL.P2Bue(Zn).3
QTL.Gha(rsMn).3
QTL.P2Bue(Ca).4
QTL.Gha(shS).2
QTL.P2Gha(Mn).2
QTL.P2Gha(P).3
QTL.P2Bue(K).6
QTL.P2Bue(Mg).6
QTL.P4Bue(Mn).3
QTL.P2Bue(Cd).1
QTL.P2Gha(Zn).5
QTL.P2Wat(Ca).4
QTL.P1Gha(rsZn).3
QTL.Gha(rsCa).2
QTL.P3Gha(K).3
QTL.P1Wat(Cu).4
QTL.P2Gha(rsZn).2
QTL.P1Bue(K).7
QTL.P1Wat(P).7
QTL.P2Bue(Se).5
QTL.P1Gha(Mg).3
QTL.P3Bue(Mo).1
QTL.P1Wat(S).5
QTL.P2Bue(S).5
QTL.P2Bue(P).7
QTL.P2Gha(Mn).3
QTL.Gha(rsZn).6
QTL.Gha(rsK).2
QTL.P1Bue(Cd).1
QTL.P2Bue(Na).6
QTL.Gha(rsMg).6
QTL.P2Gha(rsMn).1
QTL.P2Wat(Ca).5
QTL.P2Bue(Zn).4
QTL.P1Bue(Cu).4
QTL.P4Bue(B).3
QTL.P1Wat(Cu).5
QTL.P1Gha(Ca).3
QTL.P4Bue(Mn).4
QTL.P4Bue(Mg).3
QTL.P4Bue(Na).3
QTL.Gha(shMn).1
QTL.P4Bue(Mn).5
QTL.Gha(shP).4
QTL.Gha(shCu).4

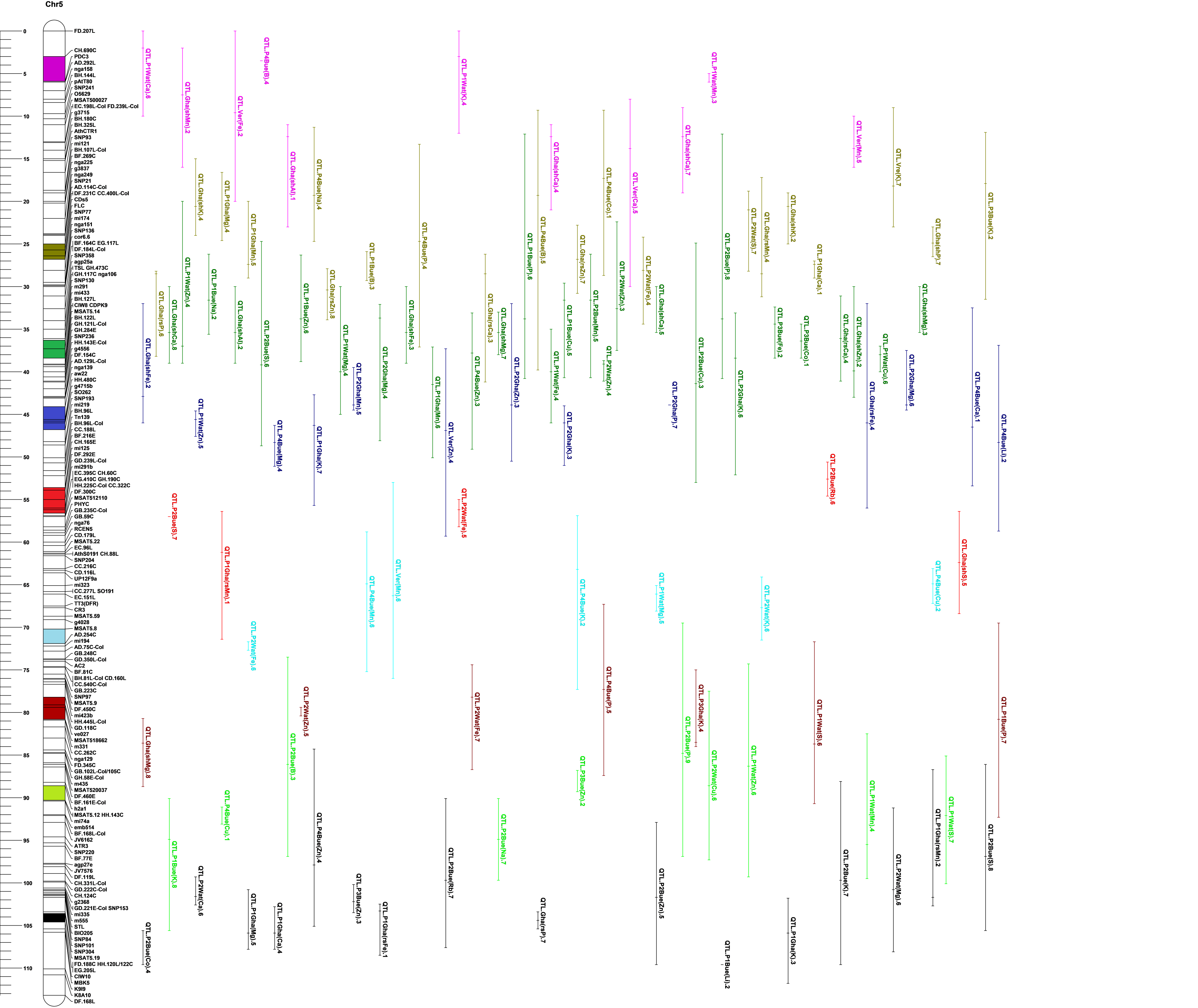
Chr5
0
5
10
15
20
25
30
35
40
45
50
55
60
65
70
75
80
85
90
95
100
105
110
FD.207L
CH.690C
PDC3
AD.292L
nga158
BH.144L
pAtT80
SNP241
O5629
MSAT500027
EC.198L-Col FD.239L-Col
g3715
BH.180C
BH.325L
AthCTR1
SNP93
mi121
BH.107L-Col
BF.269C
nga225
g3837
nga249
SNP21
AD.114C-Col
DF.231C CC.400L-Col
CDs5
FLC
SNP77
mi174
nga151
SNP136
cor6.6
BF.164C EG.117L
DF.184L-Col
SNP358
agp25a
TSL GH.473C
GH.117C nga106
SNP130
m291
mi433
BH.127L
CIW8 CDPK9
MSAT5.14
BH.122L
GH.121L-Col
GH.284E
SNP236
HH.143E-Col
g4556
DF.154C
AD.129L-Col
nga139
aw22
HH.480C
g4715b
SO262
SNP193
mi219
BH.96L
Tn139
BH.96L-Col
CC.188L
BF.216E
CH.165E
mi125
DF.292E
GD.239L-Col
mi291b
EC.395C CH.60C
EG.410C GH.190C
HH.225C-Col CC.322C
DF.300C
MSAT512110
PHYC
GB.235C-Col
GB.59C
nga76
RCEN5
CD.179L
MSAT5.22
EC.96L
AthS0191 CH.88L
SNP204
CC.216C
CD.116L
UP12F9a
mi323
CC.277L SO191
EC.151L
TT3(DFR)
CR3
MSAT5.59
g4028
MSAT5.8
AD.254C
mi194
AD.75C-Col
GB.248C
GD.350L-Col
AC2
BF.81C
BH.81L-Col CD.160L
CC.540C-Col
GB.223C
SNP97
MSAT5.9
DF.450C
mi423b
HH.445L-Col
GD.118C
ve027
MSAT518662
m331
CC.262C
nga129
FD.345C
GB.102L-Col/105C
GH.58E-Col
m435
MSAT520037
DF.460E
BF.161E-Col
h2a1
MSAT5.12 HH.143C
mi74a
emb514
BF.168L-Col
JV6162
ATR3
SNP220
BF.77E
agp27e
JV7576
DF.119L
CH.331L-Col
GD.222C-Col
CH.124C
g2368
GD.221E-Col SNP153
mi335
m555
STL
BIO205
SNP84
SNP101
SNP304
MSAT5.19
FD.188C HH.120L/122C
EG.205L
CIW10
MBK5
K9I9
K8A10
DF.168L
QTL.P1Wat(Ca).6
QTL.Gha(shMn).2
QTL.Ver(Fe).2
QTL.P4Bue(B).4
QTL.Gha(shAl).1
QTL.P4Bue(Na).4
QTL.P4Bue(P).4
QTL.P1Wat(K).4
QTL.P1Bue(P).6
QTL.P4Bue(B).6
QTL.Gha(shCa).4
QTL.P4Bue(Co).1
QTL.Ver(Ca).5
QTL.Gha(shCa).7
QTL.P1Wat(Mn).3
QTL.P2Bue(P).8
QTL.P2Wat(S).7
QTL.Gha(rsMn).4
QTL.Gha(shK).2
QTL.Ver(Mn).5
QTL.Vre(K).7
QTL.P3Bue(K).2
QTL.Gha(shK).4
QTL.P1Gha(Mg).4
QTL.P1Gha(Mn).5
QTL.P1Wat(Zn).4
QTL.P1Bue(Na).2
QTL.Gha(shAl).2
QTL.P2Bue(S).6
QTL.P1Bue(Zn).6
QTL.Gha(rsZn).8
QTL.P1Bue(B).3
QTL.P1Wat(Mg).4
QTL.P2Gha(Mg).4
QTL.Gha(shFe).3
QTL.P1Gha(Mn).6
QTL.P4Bue(Zn).3
QTL.Gha(rsCa).3
QTL.Gha(shMg).7
QTL.P2Gha(Zn).3
QTL.P1Wat(Fe).4
QTL.P1Bue(Cu).5
QTL.Gha(rsZn).7
QTL.P2Bue(Mn).5
QTL.P2Wat(Zn).3
QTL.P2Wat(Zn).4
QTL.P2Wat(Fe).4
QTL.Gha(shCa).5
QTL.P2Bue(Cu).3
QTL.P2Gha(K).6
QTL.P3Bue(Fe).2
QTL.P3Bue(Co).1
QTL.P1Gha(Ca).1
QTL.Gha(rsCa).4
QTL.Gha(shZn).2
QTL.P1Wat(Cu).6
QTL.Gha(shP).7
QTL.Gha(shMg).3
QTL.Gha(rsP).6
QTL.Gha(shCa).8
QTL.Gha(shFe).2
QTL.P1Wat(Zn).5
QTL.P4Bue(Mg).4
QTL.P1Gha(K).7
QTL.P2Gha(Mn).5
QTL.Ver(Zn).4
QTL.P2Gha(K).3
QTL.P2Gha(P).7
QTL.Gha(rsFe).4
QTL.P2Gha(Mg).6
QTL.P4Bue(Ca).1
QTL.P4Bue(Li).2
QTL.P2Bue(Rb).6
QTL.P2Bue(S).7
QTL.P1Gha(rsMn).1
QTL.P2Wat(Fe).5
QTL.Ver(Mn).6
QTL.P4Bue(Mn).6
QTL.P2Wat(Fe).6
QTL.P4Bue(K).2
QTL.P1Wat(Mg).5
QTL.P2Wat(K).6
QTL.P4Bue(Cu).2
QTL.Gha(shS).5
QTL.P2Wat(Zn).5
QTL.P2Bue(B).3
QTL.P2Wat(Fe).7
QTL.P4Bue(P).5
QTL.P3Gha(K).4
QTL.P2Bue(P).9
QTL.P1Wat(S).6
QTL.P1Bue(P).7
QTL.Gha(shMg).8
QTL.P4Bue(Cu).1
QTL.P1Bue(K).8
QTL.P4Bue(Zn).4
QTL.P2Bue(Na).7
QTL.P3Bue(Zn).2
QTL.P2Wat(Cu).6
QTL.P1Wat(Zn).6
QTL.P1Wat(Mn).4
QTL.P1Wat(S).7
QTL.P2Wat(Ca).6
QTL.P1Gha(Mg).5
QTL.P1Gha(Ca).4
QTL.P3Bue(Zn).3
QTL.P1Gha(rsFe).1
QTL.P2Bue(Rb).7
QTL.Gha(rsP).7
QTL.P2Bue(Zn).5
QTL.P1Bue(Li).2
QTL.P1Gha(K).3
QTL.P2Bue(K).7
QTL.P2Wat(Mg).6
QTL.P1Gha(rsMn).2
QTL.P2Bue(S).8
QTL.P2Bue(Co).4

Table S1. The list of identified candidate genes (CGs) on detected MQTLs in the Arabidopsis genome

MQTLs	Gene stable ID	Gene name	Gene description	Gene start (bp)	Gene end (bp)	Gene % GC content
MQTL-1/Chr1	AT1G08440	ALMT2	Aluminum-activated malate transporter 2 [Source:UniProtKB/Swiss-Prot;Acc:Q9SJE8]	2662967	2665584	36.36
	AT1G08450	CRT3	Calreticulin-3 [Source:UniProtKB/Swiss-Prot;Acc:O04153]	2667798	2671957	35.7
	AT1G08460	HDA8	Histone deacetylase 8 [Source:UniProtKB/Swiss-Prot;Acc:Q94EJ2]	2672198	2674721	42.27
	AT1G08465	YAB2	Putative axial regulator YABBY 2 [Source:UniProtKB/Swiss-Prot;Acc:Q9XFB0]	2675813	2679824	31.85
	AT1G08470	SSL3	SSL3 [Source:UniProtKB/TrEMBL;Acc:A0A178WK70]	2682058	2684171	39.97
	AT1G08480	SDH6	Succinate dehydrogenase subunit 6, mitochondrial [Source:UniProtKB/Swiss-Prot;Acc:Q941A6]	2684176	2685702	39.95
	AT1G08490	NFS2	SUFS [Source:UniProtKB/TrEMBL;Acc:A0A178WGK3]	2685659	2688658	39.77
	AT1G08500	ENODL18	Early nodulin-like protein 18 [Source:UniProtKB/TrEMBL;Acc:O82083]	2688880	2690250	35.96
	AT1G08510	FATB	Palmitoyl-acyl carrier protein thioesterase, chloroplastic [Source:UniProtKB/Swiss-Prot;Acc:Q9SJE2]	2691062	2694547	37.84
	AT1G08520	CHLD	Mg-protoporphyrin IX chelatase [Source:UniProtKB/TrEMBL;Acc:A0A178W355]	2696381	2701022	39.6
	AT1G08530		Chitinase-like protein [Source:UniProtKB/TrEMBL;Acc:Q0WNC2]	2700981	2703162	37.53
	AT1G08540	SIGB	RNA polymerase sigma factor sigB [Source:UniProtKB/Swiss-Prot;Acc:O22056]	2703295	2706860	39.15
	AT1G08550	VDE1	NPQ1 [Source:UniProtKB/TrEMBL;Acc:A0A384K8V4]	2706868	2709988	39.03
	AT1G08560	KN	SYP111 [Source:UniProtKB/TrEMBL;Acc:A0A178WAC8]	2709525	2711000	40.85
	AT1G08570	ACHT4	Thioredoxin-like 1-1, chloroplastic [Source:UniProtKB/Swiss-Prot;Acc:O64654]	2712694	2714833	38.74
	AT1G08580		At1g08580 [Source:UniProtKB/TrEMBL;Acc:Q9FRS7]	2714994	2716532	37.04
	AT1G08590	PXL1	Leucine-rich repeat receptor-like protein kinase PXL1 [Source:UniProtKB/Swiss-Prot;Acc:Q9FRS6]	2718538	2722280	42.19
	AT1G08592		Potential natural antisense gene, locus overlaps with AT1G08590 [Source:TAIR;Acc:AT1G08592]	2718616	2721910	43.55
	AT1G08600	ATRX	CHR20 [Source:UniProtKB/TrEMBL;Acc:A0A178VZQ9]	2723677	2733613	37.77
	AT1G08610		Pentatricopeptide repeat-containing protein At1g08610 [Source:UniProtKB/Swiss-Prot;Acc:Q9FRS4]	2733596	2735819	41.46
	AT1G04657			2736598	2736830	33.91
	AT1G08620	PKDM7D	Transcription factor jumonji (jmj) family protein / zinc finger (C5HC2 type) family protein [Source:TAIR;Acc:AT1G08620]	2736656	2743881	37.95
	AT1G08630	THA1	Probable low-specificity L-threonine aldolase 1 [Source:UniProtKB/Swiss-Prot;Acc:Q8RXU4]	2743761	2747967	34.75
	AT1G08640	CJD1	Chloroplast J-like domain 1 [Source:UniProtKB/TrEMBL;Acc:Q93WG3]	2748361	2751620	37.98
	AT1G08645			2751813	2752595	39.46
	AT1G08650	PPCK1	Phosphoenolpyruvate carboxylase kinase 1 [Source:UniProtKB/Swiss-Prot;Acc:Q9SPK4]	2752124	2753809	41.58
	AT1G08660	SIA1	Sialyltransferase-like protein 1 [Source:UniProtKB/Swiss-Prot;Acc:Q8VZJ0]	2756904	2759889	39.45
	AT1G08670		ENTH/VHS family protein [Source:UniProtKB/TrEMBL;Acc:Q9FRR8]	2760183	2761599	35.36
	AT1G08680	ZIGA4	ARF GAP-like zinc finger-containing protein ZIGA4 [Source:UniProtKB/Swiss-Prot;Acc:F4HXP0]	2762584	2768967	37.37
MQTL-2/Chr1	AT1G12650		At1g12650/T12C24_1 [Source:UniProtKB/TrEMBL;Acc:Q8W1E4]	4305791	4308007	35.86
	AT1G12660		Predicted to encode a PR (pathogenesis-related) protein. Belongs to the plant thionin (PR-13) family with the following memb	4308943	4309749	34.08
	AT1G12663		Thionin-like protein 2 [Source:UniProtKB/TrEMBL;Acc:A8MRP4]	4310969	4311519	37.39
	AT1G12665		Thionin-like protein [Source:UniProtKB/TrEMBL;Acc:A8MSF3]	4312331	4312935	34.55
	AT1G12672		unknown protein; LOCATED IN: endomembrane system; BEST Arabidopsis thaliana protein match is: unknown protein (TA	4318309	4319090	34.14
	AT1G12680	PEPKR2	Serine/threonine-protein kinase PEPKR2 [Source:UniProtKB/Swiss-Prot;Acc:Q8W490]	4319877	4323265	39.89
	AT1G12700		ATP binding;nucleic acid binding;helicases [Source:TAIR;Acc:AT1G12700]	4323533	4326377	38.21
	AT1G12710	AtPP2-A12	phloem protein 2-A12 [Source:TAIR;Acc:AT1G12710]	4326739	4328616	38.55
	AT1G12730		GPI transamidase subunit PIG-U [Source:UniProtKB/TrEMBL;Acc:Q94K70]	4334499	4337957	37.35
	AT1G04967			4338942	4339323	36.65
	AT1G12740	CYP87A2	Cytochrome P450, family 87, subfamily A, polypeptide 2 [Source:UniProtKB/TrEMBL;Acc:Q9LN73]	4342209	4344875	36.67
	AT1G12750	RBL6	RHOMBOID-like protein 6, mitochondrial [Source:UniProtKB/Swiss-Prot;Acc:Q8VZ48]	4344943	4348390	38.25
	AT1G12760		E3 ubiquitin-protein ligase At1g12760 [Source:UniProtKB/Swiss-Prot;Acc:Q9LN71]	4348498	4350993	40.22
	AT1G12770	RH47	DEAD-box ATP-dependent RNA helicase 47, mitochondrial [Source:UniProtKB/Swiss-Prot;Acc:Q8W4E1]	4351019	4353797	42.32
	AT1G12775		Pentatricopeptide repeat-containing protein At1g12775, mitochondrial [Source:UniProtKB/Swiss-Prot;Acc:Q9LPX2]	4353842	4355929	40.52
	AT1G12780	UGE1	Bifunctional UDP-glucose 4-epimerase and UDP-xylose 4-epimerase 1 [Source:UniProtKB/Swiss-Prot;Acc:Q42605]	4355926	4358894	36.11
	AT1G12790	PTD	Protein PARTING DANCERS [Source:UniProtKB/Swiss-Prot;Acc:F4IDW9]	4358895	4361371	35.61
	AT1G12800		Nucleic acid-binding, OB-fold-like protein [Source:UniProtKB/TrEMBL;Acc:Q94AJ9]	4361551	4365414	38.61
	AT1G12805		Nucleotide binding protein [Source:UniProtKB/TrEMBL;Acc:Q3EDD5]	4366577	4367136	37.5
	AT1G12810		Proline-rich family protein [Source:UniProtKB/TrEMBL;Acc:F4IDX2]	4367256	4368822	38.67
	AT1G04973			4367768	4368493	35.4
	AT1G12820	AFB3	Protein AUXIN SIGNALING F-BOX 3 [Source:UniProtKB/Swiss-Prot;Acc:Q9LPW7]	4368760	4371298	42.89
	AT1G04977			4368980	4369617	47.65
	AT1G12840	VHA-C	V-type proton ATPase subunit C [Source:UniProtKB/Swiss-Prot;Acc:Q9SDS7]	4374205	4378514	38.77
	AT1G12830		F13K23.8 protein [Source:UniProtKB/TrEMBL;Acc:Q9LPW6]	4374249	4375247	41.34
	AT1G12845		At1g12845 [Source:UniProtKB/TrEMBL;Acc:Q0IGM1]	4378832	4379607	38.53
	AT1G12850		At1g12850/F13K23_8 [Source:UniProtKB/TrEMBL;Acc:Q94BZ6]	4379694	4381764	42.68
	AT1G12855		Putative F-box protein At1g12855 [Source:UniProtKB/Swiss-Prot;Acc:Q9LPW4]	4382160	4383673	39.83
	AT1G12860	SCRM2	Transcription factor SCREAM2 [Source:UniProtKB/Swiss-Prot;Acc:Q9LPW3]	4384304	4386651	39.27
	AT1G12870		Putative F-box/kelch-repeat protein At1g12870 [Source:UniProtKB/Swiss-Prot;Acc:Q9LPW2]	4387129	4389110	37.03
	AT1G12880	NUDT12	NUDT12 [Source:UniProtKB/TrEMBL;Acc:A0A178WEE5]	4389678	4391344	33.53
	AT1G04983			4391438	4391675	33.19
	AT1G12890	ERF088	Ethylene-responsive transcription factor ERF088 [Source:UniProtKB/Swiss-Prot;Acc:Q3E703]	4391671	4392393	39.83
	AT1G12900	GAPA-2	glyceraldehyde 3-phosphate dehydrogenase A subunit 2 [Source:TAIR;Acc:AT1G12900]	4392438	4394454	41.3
	AT1G12910	LWD1	WD repeat-containing protein LWD1 [Source:UniProtKB/Swiss-Prot;Acc:Q9LPV9]	4394897	4396291	43.08
	AT1G12920	ERF1-2	Eukaryotic peptide chain release factor subunit 1-2 [Source:UniProtKB/Swiss-Prot;Acc:Q9LPV8]	4396348	4398193	40.74
	AT1G12930		ARM repeat superfamily protein [Source:UniProtKB/TrEMBL;Acc:F4HNZ9]	4398322	4405669	37.14
	AT1G12940	NRT2.5	High affinity nitrate transporter 2.5 [Source:UniProtKB/Swiss-Prot;Acc:Q9LPV5]	4416315	4418590	42.66
	AT1G12950	DTX31	Protein DETOXIFICATION [Source:UniProtKB/TrEMBL;Acc:A0A178WCP4]	4419714	4422684	38.07
	AT1G12960	RPL27AA	Putative 60S ribosomal protein L27a-1 [Source:UniProtKB/Swiss-Prot;Acc:Q9LPV3]	4422707	4423155	41.87
	AT1G12970	PIRL3	Plant intracellular Ras-group-related LRR protein 3 [Source:UniProtKB/Swiss-Prot;Acc:Q8W4Q3]	4423540	4425936	40.55
	AT1G12980	ESR1	Ethylene-responsive transcription factor ESR1 [Source:UniProtKB/Swiss-Prot;Acc:Q9SAD4]	4429718	4430965	44.31
	AT1G12990		Beta-1,4-N-acetylglucosaminyltransferase family protein [Source:UniProtKB/TrEMBL;Acc:F4HP06]	4433605	4436102	39.55
	AT1G13000		Transmembrane protein, putative (DUF707) [Source:UniProtKB/TrEMBL;Acc:Q8VYF6]	4436101	4439639	37.07
	AT1G13010		pre-tRNA [Source:TAIR;Acc:AT1G13010]	4440214	4440286	53.42
	AT1G13020	EIF4B2	eIF4B2 [Source:UniProtKB/TrEMBL;Acc:A0A178W345]	4440404	4443954	42.38
	AT1G13030	COIL	Coilin [Source:UniProtKB/Swiss-Prot;Acc:Q8RWK8]	4444043	4447412	39.14
	AT1G13040		Pentatricopeptide repeat-containing protein At1g13040, mitochondrial [Source:UniProtKB/Swiss-Prot;Acc:Q9SAD9]	4447549	4450001	40.97
	AT1G13050		F3F19.7 [Source:UniProtKB/TrEMBL;Acc:Q9SAE0]	4449713	4451742	40.99
	AT1G13060	PBE1	20S proteasome beta subunit E1 [Source:UniProtKB/TrEMBL;Acc:F4HP14]	4452190	4454900	37.81
	AT1G04987			4457271	4457513	35.8
	AT1G13080	CYP71B2	Cytochrome P450 71B2 [Source:UniProtKB/Swiss-Prot;Acc:O65788]	4459002	4460996	38.4
	AT1G13090	CYP71B28	Cytochrome P450 71B28 [Source:UniProtKB/Swiss-Prot;Acc:Q9SAE3]	4461738	4463469	42.32
	AT1G13100	CYP71B29	Cytochrome P450 71B29 [Source:UniProtKB/Swiss-Prot;Acc:Q9SAE4]	4463859	4465725	40.49
	AT1G13110	CYP71B7	CYP71B7 [Source:UniProtKB/TrEMBL;Acc:A0A178WGC6]	4467094	4469033	40.26
	AT1G13120	GLE1	Protein GLE1 [Source:UniProtKB/Swiss-Prot;Acc:Q0WPZ7]	4469144	4473319	37.36
	AT1G13130		At1g13130 [Source:UniProtKB/TrEMBL;Acc:Q66GP7]	4474684	4478005	36.63
	AT1G13140	CYP86C3	At1g13140 [Source:UniProtKB/TrEMBL;Acc:Q500V6]	4478384	4480327	41.15
	AT1G13143			4479025	4479709	42.63
	AT1G13150	CYP86C4	Cytochrome P450, family 86, subfamily C, polypeptide 4 [Source:UniProtKB/TrEMBL;Acc:Q9SAE8]	4481875	4483751	40.81
	AT1G13160		ARM repeat superfamily protein [Source:UniProtKB/TrEMBL;Acc:Q0WVH7]	4484584	4488577	38.86
	AT1G13170	ORP1D	OSBP(Oxysterol binding protein)-related protein 1D [Source:UniProtKB/TrEMBL;Acc:F4HP28]	4488533	4492667	39.88
	AT1G13180	ARP3	Actin-related protein 3 [Source:UniProtKB/Swiss-Prot;Acc:Q9SAF1]	4494924	4498469	37.48
	AT1G13190		F3F19.21 protein [Source:UniProtKB/TrEMBL;Acc:Q9SAF2]	4498764	4501624	40.48
	AT1G13195		At1g13195 [Source:UniProtKB/TrEMBL;Acc:Q9SAF3]	4501581	4503676	35.31
	AT1G13200		Putative F-box/kelch-repeat protein At1g13200 [Source:UniProtKB/Swiss-Prot;Acc:Q9SAF4]	4507221	4508528	42.35
	AT1G13210	ALA11	Probable phospholipid-transporting ATPase 11 [Source:UniProtKB/Swiss-Prot;Acc:Q9SAF5]	4508906	4514010	39.22

	AT1G13220	LINC2	nuclear matrix constituent protein-related [Source:TAIR;Acc:AT1G13220]	4515464	4520306	39.11
	AT1G13230	PII-2	Piriformospora indica-insensitive protein 2 [Source:UniProtKB/Swiss-Prot;Acc:Q5PP26]	4520559	4522644	37.68
	AT1G13240		pre-tRNA [Source:TAIR;Acc:AT1G13240]	4522709	4522782	55.41
	AT1G04993			4525271	4525360	26.67
	AT1G13245	RTFL17	At1g13245 [Source:UniProtKB/TrEMBL;Acc:Q9SAF8]	4525414	4526204	34.51
	AT1G04997			4526960	4527364	33.33
	AT1G13250	GATL3	Probable galacturonosyltransferase-like 3 [Source:UniProtKB/Swiss-Prot;Acc:Q0V7R1]	4528485	4530222	44.88
	AT1G05003			4532193	4532450	34.88
	AT1G13260	RAV1	AP2/ERF and B3 domain-containing transcription factor RAV1 [Source:UniProtKB/Swiss-Prot;Acc:Q9ZWM9]	4542168	4543970	39.16
	AT1G13270	MAP1B	Methionine aminopeptidase 1B, chloroplastic [Source:UniProtKB/Swiss-Prot;Acc:Q9FV52]	4544889	4547373	37.38
	AT1G13280	AOC4	Allene oxide cyclase 4, chloroplastic [Source:UniProtKB/Swiss-Prot;Acc:Q93ZC5]	4547388	4548894	40.08
	AT1G13290	WIP6	Zinc finger protein WIP6 [Source:UniProtKB/Swiss-Prot;Acc:Q9FX68]	4550153	4551620	41.28
	AT1G13300	HRS1	Transcription factor HRS1 [Source:UniProtKB/Swiss-Prot;Acc:Q9FX67]	4556878	4558793	36.8
	AT1G13310		Endosomal targeting BRO1-like domain-containing protein [Source:UniProtKB/TrEMBL;Acc:Q9FX66]	4561028	4563160	35.91
	AT1G13320	PP2AA3	Serine/threonine-protein phosphatase 2A 65 kDa regulatory subunit A gamma isoform [Source:UniProtKB/Swiss-Prot;Acc:Q	4563428	4567849	38.29
	AT1G13330	HOP2	Homologous-pairing protein 2 homolog [Source:UniProtKB/Swiss-Prot;Acc:Q9FX64]	4567935	4570598	36.37
	AT1G13340		Regulator of Vps4 activity in the MVB pathway protein [Source:UniProtKB/TrEMBL;Acc:Q9FX63]	4569448	4571229	40.52
	AT1G13350		Protein kinase superfamily protein [Source:TAIR;Acc:AT1G13350]	4572304	4576711	38.88
	AT1G13360		T6J4.11 protein [Source:UniProtKB/TrEMBL;Acc:Q9FX61]	4576873	4578369	39.41
	AT1G05007			4580080	4580364	33.68
	AT1G13370		Histone H3-like 1 [Source:UniProtKB/Swiss-Prot;Acc:Q9FX60]	4587780	4588661	37.98
	AT1G13380		Expressed protein [Source:UniProtKB/TrEMBL;Acc:Q9FX59]	4588994	4590501	37.27
	AT1G13390		At1g13390 [Source:UniProtKB/TrEMBL;Acc:Q9FX58]	4592304	4594468	34.18
	AT1G13400	JGL	Zinc finger protein JAGGED-like [Source:UniProtKB/Swiss-Prot;Acc:Q6S592]	4597567	4599141	36
MQTL-3/Chr1	AT1G19050	ARR7	At1g19050 [Source:UniProtKB/TrEMBL;Acc:Q2HIJ6]	6577833	6579314	35.83
	AT1G19060		UPF0725 protein At1g19060 [Source:UniProtKB/Swiss-Prot;Acc:Q9LMC2]	6582066	6583601	40.1
	AT1G19080	TTN10	At1g19080 [Source:UniProtKB/TrEMBL;Acc:Q6NNH6]	6584241	6586517	36.89
	AT1G19070		F-box protein At1g19070 [Source:UniProtKB/Swiss-Prot;Acc:Q3E7S2]	6584454	6584705	40.08
	AT1G19086		F14D16.23 [Source:UniProtKB/TrEMBL;Acc:Q9LMC0]	6588542	6589338	37.39
	AT1G19090	RKF2	receptor-like serine/threonine kinase 2 [Source:TAIR;Acc:AT1G19090]	6589835	6592762	39.69
	AT1G19100	MORC6	Protein MICRORCHIDIA 6 [Source:UniProtKB/Swiss-Prot;Acc:Q56Y74]	6594883	6601355	37.51
	AT1G19110		Inter-alpha-trypsin inhibitor heavy chain-like protein [Source:UniProtKB/TrEMBL;Acc:Q8L798]	6602105	6606028	39.4
	AT1G19115		unknown protein; FUNCTIONS IN: molecular_function unknown; INVOLVED IN: biological_process unknown; LOCATEI	6606237	6607943	29.53
	AT1G19120	LSM1A	LSM1A [Source:UniProtKB/TrEMBL;Acc:A0A178W3J1]	6608115	6609369	38.25
	AT1G05463			6608326	6608519	35.57
	AT1G19130		RmlC-like jelly roll fold protein [Source:UniProtKB/TrEMBL;Acc:Q8GYZ3]	6609325	6610613	40.81
	AT1G05467			6610071	6610443	38.34
	AT1G19140		FUNCTIONS IN: molecular_function unknown; INVOLVED IN: ubiquinone biosynthetic process; LOCATED IN: mitochon	6610652	6612609	40.91
	AT1G19150	LHCA6	Photosystem I chlorophyll a/b-binding protein 6, chloroplastic [Source:UniProtKB/Swiss-Prot;Acc:Q8LCQ4]	6612630	6613972	42.29
	AT1G19160		Putative F-box protein At1g19160 [Source:UniProtKB/Swiss-Prot;Acc:Q9LMB0]	6614087	6615214	41.93
	AT1G19170		Pectin lyase-like superfamily protein [Source:UniProtKB/TrEMBL;Acc:F4IE17]	6616602	6619006	42.08
	AT1G05473			6620854	6621071	38.99
	AT1G19180	TIFY10A	TIFY10A [Source:UniProtKB/TrEMBL;Acc:A0A178W7G1]	6621777	6623668	37.21
	AT1G19190	CXE1	Probable carboxylesterase 1 [Source:UniProtKB/Swiss-Prot;Acc:Q9LMA7]	6623823	6624977	43.46
	AT1G19200	FLZ12	Protein of unknown function (DUF581) [Source:TAIR;Acc:AT1G19200]	6625012	6626217	38.72
	AT1G19210	ERF017	Ethylene-responsive transcription factor ERF017 [Source:UniProtKB/Swiss-Prot;Acc:Q84QC2]	6626794	6627688	41.79
	AT1G19220	ARF19	Auxin response factor 19 [Source:UniProtKB/Swiss-Prot;Acc:Q8RYC8]	6627683	6633133	39.81
	AT1G05477			6635120	6635338	43.38
	AT1G05483			6642907	6643372	35.62
	AT1G05487			6642987	6643519	36.21
	AT1G19230	RBOHE	Respiratory burst oxidase homolog protein E [Source:UniProtKB/Swiss-Prot;Acc:O81211]	6643942	6649375	39.23
	AT1G19240		T29M8.11 [Source:UniProtKB/TrEMBL;Acc:Q9LMA2]	6649243	6650446	39.2
	AT1G19250	FMO1	Probable flavin-containing monooxygenase 1 [Source:UniProtKB/Swiss-Prot;Acc:Q9LMA1]	6650476	6653195	37.43
	AT1G19260		TTF-type zinc finger protein with HAT dimerization domain-containing protein [Source:UniProtKB/TrEMBL;Acc:F4IE28]	6657260	6659569	36.75
	AT1G19270	DA1	DA1 [Source:UniProtKB/TrEMBL;Acc:A0A178WC71]	6662417	6666032	37.47
	AT1G19290		Putative pentatricopeptide repeat-containing protein At1g19290 [Source:UniProtKB/Swiss-Prot;Acc:Q9LN69]	6666074	6669576	40.45
	AT1G05493			6669408	6669795	40.46
	AT1G05497			6670303	6670780	31.59
	AT1G19300	GATL1	Probable galacturonosyltransferase-like 1 [Source:UniProtKB/Swiss-Prot;Acc:Q9LN68]	6671095	6672777	43.2
	AT1G19310		At1g19310/F18O14_14 [Source:UniProtKB/TrEMBL;Acc:Q9LN67]	6676210	6677680	39.77
	AT1G19320		At1g19320 [Source:UniProtKB/TrEMBL;Acc:Q9LN66]	6679281	6680252	43.42
	AT1G19330		unknown protein; BEST Arabidopsis thaliana protein match is: unknown protein (TAIR:AT1G75060.1); Ha. [Source:TAIR;/	6680423	6683559	34.65
	AT1G19340		Methyltransferase-like protein 2 [Source:UniProtKB/Swiss-Prot;Acc:Q8LFA9]	6684467	6687116	38.11
	AT1G19350	BES1	Brassinosteroid signaling positive regulator (BZR1) family protein [Source:UniProtKB/TrEMBL;Acc:F4HP45]	6688463	6691216	40.85
	AT1G19360	RRA3	Arabinosyltransferase RRA3 [Source:UniProtKB/Swiss-Prot;Acc:Q9LN62]	6690399	6692566	40.54
	AT1G19370		F18O14.9 [Source:UniProtKB/TrEMBL;Acc:Q9LN61]	6692724	6695055	41.17
	AT1G19371	MIR169H	MIR169H; miRNA [Source:TAIR;Acc:AT1G19371]	6695420	6695609	39.47
	AT1G19373		snoRNA [Source:TAIR;Acc:AT1G19373]	6697178	6697253	36.84
	AT1G19376		snoRNA [Source:TAIR;Acc:AT1G19376]	6697438	6697524	42.53
	AT1G19380		At1g19380 [Source:UniProtKB/TrEMBL;Acc:Q9LN60]	6697874	6698858	38.78
	AT1G19390	WAKL11	Putative wall-associated receptor kinase-like 11 [Source:UniProtKB/Swiss-Prot;Acc:Q9LN59]	6700756	6703368	40.6
	AT1G19394		unknown protein; Ha. [Source:TAIR;Acc:AT1G19394]	6709773	6710670	38.08
	AT1G19396		unknown protein. [Source:TAIR;Acc:AT1G19396]	6709778	6711788	38.99
	AT1G19397		unknown protein; Ha. [Source:TAIR;Acc:AT1G19397]	6710851	6711787	38.21
	AT1G19400		Erythronate-4-phosphate dehydrogenase family protein [Source:UniProtKB/TrEMBL;Acc:Q8VYC6]	6712022	6714483	37.65
	AT1G19410		FBD / Leucine Rich Repeat domains containing protein [Source:UniProtKB/TrEMBL;Acc:F4HP61]	6714492	6716439	36.55
	AT1G19430		Probable methyltransferase PMT28 [Source:UniProtKB/Swiss-Prot;Acc:Q9LN50]	6724413	6728075	38.63
	AT1G19440	KCS4	3-ketoacyl-CoA synthase 4 [Source:UniProtKB/Swiss-Prot;Acc:Q9LN49]	6728747	6730931	41.65
	AT1G19450		Sugar transporter ERD6-like 4 [Source:UniProtKB/Swiss-Prot;Acc:Q93YP9]	6731411	6735024	34.92
	AT1G19460		Kelch repeat-containing protein At1g19460 [Source:UniProtKB/Swiss-Prot;Acc:Q3ED93]	6738484	6739734	43.65
	AT1G19464	MIR864A	MIR864a; miRNA [Source:TAIR;Acc:AT1G19464]	6740500	6740591	29.35
	AT1G19470		Kelch repeat-containing protein At1g19470 [Source:UniProtKB/Swiss-Prot;Acc:P0C2F7]	6741296	6742534	44.71
	AT1G19480		At1g19480 [Source:UniProtKB/TrEMBL;Acc:Q0V7V4]	6744353	6746635	42.4
	AT1G19485		Transducin/WD40 repeat-like superfamily protein [Source:UniProtKB/TrEMBL;Acc:F4HP69]	6746778	6751609	39.26
	AT1G19490		Basic-leucine zipper (BZIP) transcription factor family protein [Source:UniProtKB/TrEMBL;Acc:Q8L5Y2]	6751544	6754073	39.49
	AT1G19500		Putative uncharacterized protein [Source:UniProtKB/TrEMBL;Acc:Q5Q0G4]	6754665	6755684	35.2
	AT1G19510	RL5	Protein RADIALIS-like 5 [Source:UniProtKB/Swiss-Prot;Acc:Q8GW75]	6756237	6757420	32.01
	AT1G19520	NFD5	pentatricopeptide (PPR) repeat-containing protein [Source:TAIR;Acc:AT1G19520]	6759859	6762847	40.72
	AT1G19530		DNA polymerase epsilon catalytic subunit A [Source:UniProtKB/TrEMBL;Acc:Q93WK6]	6763765	6765049	33.93
	AT1G19540		At1g19540 [Source:UniProtKB/TrEMBL;Acc:Q29PX7]	6765653	6768290	39.23
	AT1G19550		F18O14.31 [Source:UniProtKB/TrEMBL;Acc:Q9LN39]	6767451	6768158	43.79
	AT1G19565		UPF0725 protein At1g19565 [Source:UniProtKB/Swiss-Prot;Acc:P0C8P5]	6772084	6773131	43.32
	AT1G19570	DHAR1	Glutathione S-transferase DHAR1, mitochondrial [Source:UniProtKB/Swiss-Prot;Acc:Q9FWR4]	6773302	6774523	42.06
	AT1G19580	GAMMACA1	GAMMA CA1 [Source:UniProtKB/TrEMBL;Acc:A0A178WHZ1]	6774683	6777568	38.84
	AT1G19600		At1g19600 [Source:UniProtKB/TrEMBL;Acc:Q9LN35]	6778887	6781222	41.05
	AT1G19610	PDF1.4	Defensin-like protein 19 [Source:UniProtKB/Swiss-Prot;Acc:P82787]	6781483	6782216	37.87
	AT1G19630	CYP722A1	Cytochrome P450, family 722, subfamily A, polypeptide 1 [Source:UniProtKB/TrEMBL;Acc:F4HP86]	6783803	6788175	37.27
	AT1G19620		unknown protein; Ha. [Source:TAIR;Acc:AT1G19620]	6784020	6785075	48.3

	AT1G19640	JMT	Jasmonate O-methyltransferase [Source:UniProtKB/Swiss-Prot;Acc:Q9AR07]	6788885	6791919	35.82
	AT1G19650	SFH4	Phosphatidylinositol/phosphatidylcholine transfer protein SFH4 [Source:UniProtKB/Swiss-Prot;Acc:F4HP88]	6796137	6799764	37.93
	AT1G19660	BBD2	Bifunctional nuclease 2 [Source:UniProtKB/Swiss-Prot;Acc:Q93VH2]	6799969	6802906	37.78
	AT1G19670	CLH1	Chlorophyllase-1 [Source:UniProtKB/Swiss-Prot;Acc:O22527]	6803486	6805092	40.7
	AT1G19680		At1g19680 [Source:UniProtKB/TrEMBL;Acc:Q9FXG6]	6805430	6807862	42.25
	AT1G19690		At1g19690 [Source:UniProtKB/TrEMBL;Acc:Q147R9]	6807715	6809642	39.99
	AT1G19700	BLH10	BEL1-like homeodomain protein 10 [Source:UniProtKB/Swiss-Prot;Acc:Q9FXG8]	6809585	6813063	37.19
	AT1G05507			6813647	6814005	31.48
	AT1G19710		At1g19710 [Source:UniProtKB/TrEMBL;Acc:Q67Z55]	6814704	6816876	39.3
	AT1G19715	JAL3	Jacalin-related lectin 3 [Source:UniProtKB/Swiss-Prot;Acc:F4HQX1]	6816702	6819542	39.99
	AT1G19720	DYW7	Pentatricopeptide repeat-containing protein At1g19720 [Source:UniProtKB/Swiss-Prot;Acc:Q9FXH1]	6819564	6822711	39.64
	AT1G19730	TRX4	Thioredoxin H4 [Source:UniProtKB/Swiss-Prot;Acc:Q39239]	6822847	6824071	36.82
	AT1G19740		ATP-dependent protease La (LON) domain protein [Source:UniProtKB/TrEMBL;Acc:Q9FXH3]	6824246	6825459	43.74
	AT1G19750		Transducin/WD40 repeat-like superfamily protein [Source:UniProtKB/TrEMBL;Acc:F4HQX6]	6826830	6830330	38.96
	AT1G19770	PUP14	PUP14 [Source:UniProtKB/TrEMBL;Acc:A0A178WHV5]	6832325	6833837	41.77
	AT1G19780	CNGC8	Putative cyclic nucleotide-gated ion channel 8 [Source:UniProtKB/Swiss-Prot;Acc:Q9FXH6]	6833669	6836633	38.72
	AT1G19790	SRS7	Protein SHI RELATED SEQUENCE 7 [Source:UniProtKB/Swiss-Prot;Acc:Q9FXH7]	6838120	6840313	39.2
	AT1G19800	TGD1	Protein TRIGALACTOSYLDIACYLGLYCEROL 1, chloroplastic [Source:UniProtKB/Swiss-Prot;Acc:Q8L4R0]	6846338	6848128	39.53
	AT1G19830		At1g19830 [Source:UniProtKB/TrEMBL;Acc:Q9FXI0]	6852116	6852945	37.71
	AT1G19835	FPP4	Filament-like plant protein 4 [Source:UniProtKB/Swiss-Prot;Acc:Q0WSY2]	6855899	6860563	37.81
	AT1G19840		F6F9.11 protein [Source:UniProtKB/TrEMBL;Acc:Q9FXI2]	6872275	6873358	42.34
	AT1G19850	ARF5	Auxin response factor [Source:UniProtKB/TrEMBL;Acc:A0A178W993]	6886669	6891404	40.2
	AT1G19860		Zinc finger C-x8-C-x5-C-x3-H type family protein [Source:TAIR;Acc:AT1G19860]	6891382	6894752	39.45
	AT1G19870	IQD32	Protein IQ-DOMAIN 32 [Source:UniProtKB/Swiss-Prot;Acc:Q9FXI5]	6895009	6898818	40.39
	AT1G19880		Regulator of chromosome condensation (RCC1) family protein [Source:UniProtKB/TrEMBL;Acc:F4HQZ1]	6900337	6904155	39.49
	AT1G19890	MGH3	MGH3 [Source:UniProtKB/TrEMBL;Acc:A0A178WKL2]	6904963	6906028	39.02
	AT1G19900		At1g19900/F6F9_4 [Source:UniProtKB/TrEMBL;Acc:Q93Z02]	6906899	6908805	43.52
	AT1G19910	VHA-C2	V-type proton ATPase proteolipid subunit [Source:UniProtKB/TrEMBL;Acc:Q24JM2]	6913001	6914634	38.31
	AT1G19920	APS2	ATP sulfurylase 2 [Source:UniProtKB/Swiss-Prot;Acc:Q43870]	6914518	6916823	38.68
	AT1G19930		Putative F-box/kelch-repeat protein At1g19930 [Source:UniProtKB/Swiss-Prot;Acc:Q3ED92]	6917020	6918138	43.97
	AT1G19940	AtGH9B5	Endoglucanase 2 [Source:UniProtKB/Swiss-Prot;Acc:Q9FXI9]	6918159	6921979	35.23
	AT1G19950	HVA22H	HVA22-like protein h [Source:UniProtKB/Swiss-Prot;Acc:Q8LEM6]	6924091	6926741	38.93
	AT1G19960		Putative uncharacterized protein At1g19960 [Source:UniProtKB/TrEMBL;Acc:Q9LNS5]	6927736	6928656	38.11
	AT1G19968		other RNA [Source:TAIR;Acc:AT1G19968]	6930261	6930985	37.52
	AT1G19970		ER lumen protein retaining receptor family protein [Source:UniProtKB/TrEMBL;Acc:F4HR00]	6931012	6932826	38.79
	AT1G19980		Cytomatrix protein-like protein [Source:UniProtKB/TrEMBL;Acc:Q84JE5]	6933015	6935053	36.68
	AT1G19990		unknown protein; FUNCTIONS IN: molecular_function unknown; INVOLVED IN: biological_process unknown; LOCATEI	6935218	6936928	37.29
	AT1G20000	TAF11B	Transcription initiation factor TFIID subunit 11b [Source:UniProtKB/Swiss-Prot;Acc:F4HR03]	6936898	6937666	40.96
	AT1G20010	TUBB5	Tubulin beta-5 chain [Source:UniProtKB/Swiss-Prot;Acc:P29513]	6937719	6940832	38.09
	AT1G20015		snoRNA [Source:TAIR;Acc:AT1G20015]	6941285	6941373	46.07
	AT1G05517			6941873	6942155	44.17
	AT1G20020	LFNR2	Ferredoxin--NADP reductase, leaf isozyme 2, chloroplastic [Source:UniProtKB/Swiss-Prot;Acc:Q8W493]	6942625	6945091	36.32
	AT1G20030		Pathogenesis-related thaumatin superfamily protein [Source:UniProtKB/TrEMBL;Acc:Q9LNT0]	6945425	6947344	41.82
	AT1G20040		pre-tRNA [Source:TAIR;Acc:AT1G20040]	6948860	6948943	57.14
	AT1G20050	HYD1	Probable 3-beta-hydroxysterol-Delta(8),Delta(7)-isomerase [Source:UniProtKB/Swiss-Prot;Acc:O48962]	6949043	6950509	40.35
	AT1G20060		ATP binding microtubule motor family protein [Source:TAIR;Acc:AT1G20060]	6950393	6956400	38.52
	AT1G20065		DNA-directed RNA polymerase I subunit RPA12-like protein [Source:UniProtKB/TrEMBL;Acc:Q1G3U4]	6956374	6958075	36.08
	AT1G05523			6957968	6959124	39.07
	AT1G20070		T20H2.15 protein [Source:UniProtKB/TrEMBL;Acc:Q9LNT3]	6958010	6959148	40.04
	AT1G05527			6959160	6959605	41.48
	AT1G05533			6961365	6961574	36.67
	AT1G20080	SYT2	Synaptotagmin-2 [Source:UniProtKB/Swiss-Prot;Acc:B6ETT4]	6962030	6965443	35.15
	AT1G20090	ARAC4	ROP2 [Source:UniProtKB/TrEMBL;Acc:A0A384L636]	6966842	6969009	37.5
	AT1G20100		DNA ligase-like protein [Source:UniProtKB/TrEMBL;Acc:Q9LNT6]	6969025	6971036	38.32
	AT1G20110	FREE1	Protein FREE1 [Source:UniProtKB/Swiss-Prot;Acc:Q9ASS2]	6971424	6975130	40.28
	AT1G20120		GDSL esterase/lipase At1g20120 [Source:UniProtKB/Swiss-Prot;Acc:P0DKJ6]	6975339	6977267	36.96
	AT1G20130	APG	Anther-specific proline-rich protein APG [Source:UniProtKB/Swiss-Prot;Acc:P40602]	6977753	6980241	39.65
	AT1G20132		GDSL-like Lipase/Acylhydrolase superfamily protein [Source:UniProtKB/TrEMBL;Acc:B3H6L2]	6981264	6983627	32.49
	AT1G20135		GDSL-like Lipase/Acylhydrolase family protein [Source:UniProtKB/TrEMBL;Acc:B3H5G5]	6984042	6985703	34.78
	AT1G20140	ASK4	SK4 [Source:UniProtKB/TrEMBL;Acc:A0A178W187]	6986312	6987863	41.62
	AT1G20150	SBT5.1	Subtilisin-like protease SBT5.1 [Source:UniProtKB/Swiss-Prot;Acc:F4HSQ2]	6987272	6990362	41.28
	AT1G20160	CRSP	CO(2)-response secreted protease [Source:UniProtKB/Swiss-Prot;Acc:Q9LNU1]	6990642	6993989	40.98
	AT1G20170		pre-tRNA [Source:TAIR;Acc:AT1G20170]	6995282	6995353	59.72
	AT1G20180		UPF0496 protein At1g20180 [Source:UniProtKB/Swiss-Prot;Acc:Q6DYE5]	6996176	6997943	36.65
	AT1G20190	EXPA11	Expansin-A11 [Source:UniProtKB/Swiss-Prot;Acc:Q9LNU3]	6998335	6999839	38.54
	AT1G20200	RPN3A	26S proteasome non-ATPase regulatory subunit 3 homolog A [Source:UniProtKB/Swiss-Prot;Acc:Q9LNU4]	7001087	7004432	39.9
	AT1G20210		pre-tRNA [Source:TAIR;Acc:AT1G20210]	7004514	7004585	62.5
	AT1G20220		Alba DNA/RNA-binding protein [Source:UniProtKB/TrEMBL;Acc:Q944A2]	7004824	7007717	39.12
	AT1G20225		Thioredoxin superfamily protein [Source:UniProtKB/TrEMBL;Acc:Q8W456]	7007756	7009380	38.52
	AT1G20230	PCMP-H21	Pentatricopeptide repeat-containing protein At1g20230 [Source:UniProtKB/Swiss-Prot;Acc:Q9LNU6]	7009568	7012107	40.63
	AT1G20240		SWI-SNF-related chromatin binding protein [Source:UniProtKB/TrEMBL;Acc:F4HSR2]	7012113	7015216	36.02
	AT1G20250		pre-tRNA [Source:TAIR;Acc:AT1G20250]	7016516	7016588	63.01
	AT1G20260	VHA-B3	VAB3 [Source:UniProtKB/TrEMBL;Acc:A0A178WCF6]	7016599	7020690	37.22
	AT1G20270	P4H3	Probable prolyl 4-hydroxylase 3 [Source:UniProtKB/Swiss-Prot;Acc:Q9LN20]	7020664	7023162	36.05
	AT1G20280		homeobox-leucine zipper protein-related [Source:TAIR;Acc:AT1G20280]	7023682	7024439	34.96
	AT1G20290		SWI-SNF-related chromatin binding protein [Source:UniProtKB/TrEMBL;Acc:F4HSR6]	7025548	7029698	32.26
	AT1G20300		Pentatricopeptide repeat-containing protein At1g20300, mitochondrial [Source:UniProtKB/Swiss-Prot;Acc:Q9LN22]	7029629	7031535	41.37
	AT1G20310		Syringolide-induced protein [Source:UniProtKB/TrEMBL;Acc:Q8L956]	7032070	7033329	41.83
	AT1G20320		F14O10.8 protein [Source:UniProtKB/TrEMBL;Acc:Q9LN24]	7033657	7034881	40.57
	AT1G20330	SMT2	24-methylenesterol C-methyltransferase 2 [Source:UniProtKB/Swiss-Prot;Acc:Q39227]	7038527	7040231	43.46
	AT1G20340	DRT112	Plastocyanin major isoform, chloroplastic [Source:UniProtKB/Swiss-Prot;Acc:P42699]	7042046	7043392	38.83
	AT1G20350	TIM17-1	Mitochondrial import inner membrane translocase subunit TIM17-1 [Source:UniProtKB/Swiss-Prot;Acc:Q9LN27]	7043628	7044592	42.07
	AT1G05537			7046123	7046378	31.25
	AT1G20360		F-box protein At1g20360 [Source:UniProtKB/Swiss-Prot;Acc:Q84JN6]	7046504	7047744	42.06
	AT1G05543			7049116	7049939	39.08
	AT1G05547			7050831	7051140	40
	AT1G20370		F14O10.3 protein [Source:UniProtKB/TrEMBL;Acc:Q9LN29]	7051577	7053745	42.51
	AT1G20375	MIR394A	MIR394A; miRNA [Source:TAIR;Acc:AT1G20375]	7058194	7058310	39.32
	AT1G20380		Prolyl oligopeptidase family protein [Source:UniProtKB/TrEMBL;Acc:F4HSS5]	7061442	7065242	38.44
	AT1G20400		Protein of unknown function (DUF1204) [Source:TAIR;Acc:AT1G20400]	7072192	7076619	49.82
	AT1G20405		unknown protein; FUNCTIONS IN: molecular_function unknown; INVOLVED IN: biological_process unknown; LOCATEI	7077116	7077606	34.83
	AT1G20410		Pseudouridine synthase family protein [Source:UniProtKB/TrEMBL;Acc:F4HSS8]	7078804	7082566	37.66
	AT1G20420		pre-tRNA [Source:TAIR;Acc:AT1G20420]	7082668	7082739	58.33
	AT1G20430		Putative uncharacterized protein [Source:UniProtKB/TrEMBL;Acc:Q94B54]	7083012	7083910	40.6
	AT1G20440	COR47	RD17 [Source:UniProtKB/TrEMBL;Acc:A0A384KK16]	7084409	7086873	36.11
	AT1G20450	ERD10	Dehydrin ERD10 [Source:UniProtKB/Swiss-Prot;Acc:P42759]	7087903	7089606	38.2
MQTL-4/Chr1	AT1G20950	PFP-ALPHA1	Pyrophosphate--fructose 6-phosphate 1-phosphotransferase subunit alpha [Source:UniProtKB/TrEMBL;Acc:A0A178WMV9	7297239	7301633	37.36
	AT1G20960	BRR2A	DExH-box ATP-dependent RNA helicase DExH12 [Source:UniProtKB/Swiss-Prot;Acc:Q9SYP1]	7301599	7310271	42.64

AT1G20970		FUNCTIONS IN: molecular_function unknown; INVOLVED IN: biological_process unknown; LOCATED IN: plasma mem	7313901	7319921	41.17
AT1G20980	SPL14	Squamosa promoter-binding-like protein 14 [Source:UniProtKB/Swiss-Prot;Acc:Q8RY95]	7324471	7329441	40.31
AT1G20990		Cysteine/Histidine-rich C1 domain family protein [Source:UniProtKB/TrEMBL;Acc:F4HWC5]	7330256	7332100	40.7
AT1G05613			7336674	7336952	40.14
AT1G21000		At1g21000/F9H16_1 [Source:UniProtKB/TrEMBL;Acc:Q93V70]	7337605	7339452	35.06
AT1G05617			7344747	7345128	35.34
AT1G21010		At1g21010 [Source:UniProtKB/TrEMBL;Acc:Q7XJ58]	7346156	7347283	38.56
AT1G21050		Protein of unknown function, DUF617 [Source:TAIR;Acc:AT1G21050]	7366590	7368203	38.97
AT1G05623			7370611	7370903	35.49
AT1G21060		Protein of unknown function, DUF547 [Source:TAIR;Acc:AT1G21060]	7371657	7374114	37.35
AT1G21065		Secondary thiamine-phosphate synthase enzyme [Source:UniProtKB/TrEMBL;Acc:Q9LPU1]	7374115	7375789	37.13
AT1G21070	URGT2	UDP-rhamnose/UDP-galactose transporter 2 [Source:UniProtKB/Swiss-Prot;Acc:Q9LPU2]	7375869	7378227	38.15
AT1G21080		DNAJ heat shock N-terminal domain-containing protein [Source:UniProtKB/TrEMBL;Acc:F4HWD5]	7378538	7382661	37.2
AT1G21090		At1g21090 [Source:UniProtKB/TrEMBL;Acc:Q0V805]	7384560	7386580	36.71
AT1G21100	IGMT1	Indole glucosinolate O-methyltransferase 1 [Source:UniProtKB/Swiss-Prot;Acc:Q9LPU5]	7386762	7388512	40.26
AT1G21110	IGMT3	Indole glucosinolate O-methyltransferase 3 [Source:UniProtKB/Swiss-Prot;Acc:Q9LPU6]	7389981	7391603	39.86
AT1G21120		O-methyltransferase family protein [Source:TAIR;Acc:AT1G21120]	7395179	7396823	40.43
AT1G21130	IGMT4	Indole glucosinolate O-methyltransferase 4 [Source:UniProtKB/Swiss-Prot;Acc:Q9LPU8]	7398814	7400736	38.79
AT1G05627			7400802	7400992	27.23
AT1G05633			7401345	7401549	37.56
AT1G21140		Vacuolar iron transporter homolog 1 [Source:UniProtKB/Swiss-Prot;Acc:Q9LPU9]	7404328	7405426	42.77
AT1G21150		Mitochondrial transcription termination factor family protein [Source:TAIR;Acc:AT1G21150]	7406218	7407870	40.29
AT1G21160		Eukaryotic translation initiation factor 2 (eIF-2) family protein (Fragment) [Source:UniProtKB/TrEMBL;Acc:C0SUW8]	7407931	7413295	39.2
AT1G21170	SEC5B	Exocyst complex component SEC5 [Source:TAIR;Acc:AT1G21170]	7412830	7419643	38.7
AT1G21190	LSM3A	Sm-like protein LSM3A [Source:UniProtKB/Swiss-Prot;Acc:Q9LMN4]	7419664	7420957	38.25
AT1G21200		F16F4.11 protein [Source:UniProtKB/TrEMBL;Acc:Q9LMN5]	7421148	7423673	37.57
AT1G21202	MIR781A	MIR781a; miRNA [Source:TAIR;Acc:AT1G21202]	7423518	7423611	28.72
AT1G05637			7423521	7423607	28.74
AT1G21210	WAK4	Wall-associated receptor kinase 4 [Source:UniProtKB/Swiss-Prot;Acc:Q9LMN6]	7423704	7427041	41.46
AT1G21230	WAK5	Wall-associated receptor kinase 5 [Source:UniProtKB/Swiss-Prot;Acc:Q9LMN7]	7429895	7432477	41.08
AT1G05643			7433801	7434105	34.43
AT1G21240	WAK3	Wall-associated receptor kinase 3 [Source:UniProtKB/Swiss-Prot;Acc:Q9LMN8]	7434235	7436830	41.6
AT1G21245		F16F4.7 protein [Source:UniProtKB/TrEMBL;Acc:Q9LMN9]	7436842	7437342	45.31
AT1G21250	WAK1	Wall-associated receptor kinase 1 [Source:UniProtKB/Swiss-Prot;Acc:Q39191]	7439171	7442113	42.2
AT1G21270	WAK2	Wall-associated receptor kinase 2 [Source:UniProtKB/Swiss-Prot;Acc:Q9LMP1]	7444749	7448483	42.28
AT1G21280		Copia-like polyprotein/retrotransposon [Source:UniProtKB/TrEMBL;Acc:Q84JT5]	7447490	7448483	42.76
AT1G21310	EXT3	Extensin-3 [Source:UniProtKB/Swiss-Prot;Acc:Q9FS16]	7453245	7455009	45.44
AT1G21313		Transmembrane protein [Source:UniProtKB/TrEMBL;Acc:A0A1P8ATC7]	7453735	7454256	51.92
AT1G05647			7460104	7460501	38.19
AT1G21320	NSRB	Nuclear speckle RNA-binding protein B [Source:UniProtKB/Swiss-Prot;Acc:F4HWF9]	7462327	7466164	38.72
AT1G21323		unknown protein; Ha. [Source:TAIR;Acc:AT1G21323]	7466694	7467359	38.29
AT1G21326		F16F4.1 protein [Source:UniProtKB/TrEMBL;Acc:Q9LMP5]	7468765	7469995	39.89
AT1G05653			7471090	7471300	36.02
AT1G05657			7473011	7473358	39.94
AT1G21340	DOF1.2	Dof zinc finger protein DOF1.2 [Source:UniProtKB/Swiss-Prot;Acc:P68349]	7476022	7476899	45.33
AT1G21350		Thioredoxin superfamily protein [Source:TAIR;Acc:AT1G21350]	7476902	7479258	36.44
AT1G21360	GLTP2	Glycolipid transfer protein 2 [Source:UniProtKB/Swiss-Prot;Acc:Q6NLQ3]	7481184	7483513	32.53
AT1G21370		unknown protein; CONTAINS InterPro DOMAIN/s: Protein of unknown function DUF218 (InterPro:IPR003848); Ha. [Sourc	7483781	7485666	39.61
AT1G21380	TOL3	TOM1-like protein 3 [Source:UniProtKB/Swiss-Prot;Acc:Q9LPL6]	7485636	7488780	38.76
AT1G21390	emb2170	Emb2170 [Source:UniProtKB/TrEMBL;Acc:A0A178W1S8]	7489046	7490803	36.8
AT1G05663			7490992	7491645	35.93
AT1G05667			7491251	7491464	41.12
AT1G05673			7491379	7491489	50.45
AT1G21395		unknown protein; BEST Arabidopsis thaliana protein match is: unknown protein (TAIR:AT2G26865.1); Ha. [Source:TAIR;A	7491965	7492351	36.18
AT1G21400		Thiamin diphosphate-binding fold (THDP-binding) superfamily protein [Source:TAIR;Acc:AT1G21400]	7493213	7496741	37.72
AT1G21410	SKP2A	F-box protein SKP2A [Source:UniProtKB/Swiss-Prot;Acc:Q9LPL4]	7496998	7499616	38.49
AT1G21420		pre-tRNA [Source:TAIR;Acc:AT1G21420]	7499733	7499804	54.17
AT1G21430	YUC11	Probable indole-3-pyruvate monooxygenase YUCCA11 [Source:UniProtKB/Swiss-Prot;Acc:Q9LPL3]	7500845	7502186	44.34
AT1G21440		At1g21440 [Source:UniProtKB/TrEMBL;Acc:Q501F7]	7502082	7504275	39.88
AT1G21450	SCL1	Scarecrow-like protein 1 [Source:UniProtKB/Swiss-Prot;Acc:Q9SDQ3]	7508701	7511801	38.28
AT1G21460	SWEET1	Bidirectional sugar transporter SWEET1 [Source:UniProtKB/Swiss-Prot;Acc:Q8L9J7]	7511750	7513481	36.49
AT1G21470		BEST Arabidopsis thaliana protein match is: CLPC homologue 1 (TAIR:AT5G50920.1); Ha. [Source:TAIR;Acc:AT1G2147(	7516009	7517637	39.35
AT1G21480		Exostosin family protein [Source:UniProtKB/TrEMBL;Acc:F4HY10]	7518946	7521552	39.05
AT1G21475		Putative uncharacterized protein [Source:UniProtKB/TrEMBL;Acc:Q3ED80]	7523972	7524721	44.53
AT1G21500		F24J8.11 protein [Source:UniProtKB/TrEMBL;Acc:Q9LPK9]	7529705	7531108	36.61
AT1G21510		F24J8.12 protein [Source:UniProtKB/TrEMBL;Acc:Q9LPK8]	7531312	7532745	38.35
AT1G21520		Uncharacterized protein At1g21520/F24J8_4 [Source:UniProtKB/TrEMBL;Acc:Q8GYE6]	7534019	7534927	36.3
AT1G21528		Putative uncharacterized protein [Source:UniProtKB/TrEMBL;Acc:Q1G3U3]	7542615	7543077	39.31
AT1G21529			7543924	7544678	40.93
AT1G21530	AEE10	Probable acyl-activating enzyme 10 [Source:UniProtKB/Swiss-Prot;Acc:Q9LPK7]	7545149	7546988	47.12
AT1G21540	AEE9	Probable acyl-activating enzyme 9 [Source:UniProtKB/Swiss-Prot;Acc:Q9LPK6]	7548450	7550741	44.59
AT1G21550	CML44	Probable calcium-binding protein CML44 [Source:UniProtKB/Swiss-Prot;Acc:Q9LPK5]	7552987	7553975	37.71
AT1G21560		F14J8.16 protein [Source:UniProtKB/TrEMBL;Acc:Q9LPK4]	7554671	7557112	36.98
AT1G21580		Zinc finger C-x8-C-x5-C-x3-H type family protein [Source:TAIR;Acc:AT1G21580]	7557140	7565914	41.65
AT1G21590		At1g21590/F24J8_9 [Source:UniProtKB/TrEMBL;Acc:Q8VZG4]	7566229	7570014	39.43
AT1G21600	PTAC6	AT1G21600 protein [Source:UniProtKB/TrEMBL;Acc:Q9XI19]	7570845	7573847	38.79
AT1G21610		Wound-responsive family protein [Source:UniProtKB/TrEMBL;Acc:F4HY28]	7573830	7578963	38.04
AT1G21620	APUM20	pumilio 20 [Source:TAIR;Acc:AT1G21620]	7579129	7580171	47.36
AT1G21630		Calcium-binding EF hand family protein [Source:UniProtKB/TrEMBL;Acc:F4HY32]	7581008	7588153	40.96
AT1G21640	NADK2	NAD kinase 2 [Source:UniProtKB/TrEMBL;Acc:F4HY34]	7588414	7592890	41.08
AT1G21650	SECA2	Protein translocase subunit SecA [Source:UniProtKB/TrEMBL;Acc:F4HY36]	7592664	7604229	37.8
AT1G21651		Putative SecA-type chloroplast protein transport factor [Source:UniProtKB/TrEMBL;Acc:Q8VZ06]	7600805	7604215	40.28
AT1G05683			7604410	7604655	37.8
AT1G21660		At1g21660 [Source:UniProtKB/TrEMBL;Acc:Q9XI12]	7605620	7609223	38.68
AT1G21670		DPP6 amino-terminal domain protein [Source:UniProtKB/TrEMBL;Acc:Q9XI11]	7610241	7612721	46.55
AT1G21680		DPP6 N-terminal domain-like protein [Source:UniProtKB/TrEMBL;Acc:Q9XI10]	7612834	7615374	47.07
AT1G21690	EMB1968	ATPase family associated with various cellular activities (AAA) [Source:UniProtKB/TrEMBL;Acc:F4HY43]	7615505	7618628	37.39
AT1G21695		At1g21695 [Source:UniProtKB/TrEMBL;Acc:Q9XI08]	7618862	7619919	40.08
AT1G21700	SWI3C	SWI/SNF complex subunit SWI3C [Source:UniProtKB/Swiss-Prot;Acc:Q9XI07]	7620001	7624166	40.95
AT1G21710	OGG1	OGG1 [Source:UniProtKB/TrEMBL;Acc:A0A178WC14]	7624320	7626138	42.44
AT1G21720	PBC1	Proteasome subunit beta type [Source:UniProtKB/TrEMBL;Acc:A0A178WJZ7]	7626182	7628290	38.22
AT1G21722		F8K7.16 [Source:UniProtKB/TrEMBL;Acc:Q9XI04]	7628456	7629364	39.38
AT1G21730	KIN7C	Kinesin-like protein KIN-7C, mitochondrial [Source:UniProtKB/Swiss-Prot;Acc:Q8W5R6]	7630107	7636658	37.62
AT1G21740		DUF630 family protein, putative (DUF630 and DUF632) [Source:UniProtKB/TrEMBL;Acc:Q9XI02]	7640906	7645240	41.04
AT1G21738		unknown protein; Ha. [Source:TAIR;Acc:AT1G21738]	7640992	7641225	31.2
AT1G21750	PDIL1-1	Protein disulfide isomerase-like 1-1 [Source:UniProtKB/Swiss-Prot;Acc:Q9XI01]	7645610	7648855	38.72
AT1G21760	SKIP32	F-box protein 7 [Source:UniProtKB/Swiss-Prot;Acc:Q9XI00]	7649194	7651816	36.79
AT1G21770		Acyl-CoA N-acyltransferases (NAT) superfamily protein [Source:UniProtKB/TrEMBL;Acc:Q8H0Y9]	7651646	7652288	40.12

AT1G21780		BTB/POZ domain-containing protein At1g21780 [Source:UniProtKB/Swiss-Prot;Acc:Q9XHZ8]	7651882	7654014	41.02
AT1G21790		F8K7.23 [Source:UniProtKB/TrEMBL;Acc:Q9XHZ7]	7654088	7655981	40.13
AT1G21800		pre-tRNA [Source:TAIR;Acc:AT1G21800]	7656120	7656191	54.17
AT1G21810	FPP2	Filament-like plant protein 2 [Source:UniProtKB/Swiss-Prot;Acc:Q9SFF4]	7656319	7659167	37.7
AT1G21830		At1g21820 [Source:UniProtKB/TrEMBL;Acc:Q8VY47]	7660947	7662725	33.33
AT1G21835		Plant thionin family protein [Source:UniProtKB/TrEMBL;Acc:A8MRY8]	7665664	7665936	38.1
AT1G21840	UREF	Urease accessory protein F [Source:UniProtKB/Swiss-Prot;Acc:Q9XHZ3]	7666414	7667794	37.51
AT1G21850	sks8	Sks8 [Source:UniProtKB/TrEMBL;Acc:A0A178W9U5]	7667803	7670530	40.8
AT1G21860	sks7	SKU5 similar 7 [Source:UniProtKB/TrEMBL;Acc:Q9SFF1]	7671028	7674215	37.92
AT1G21864		Plant thionin family protein [Source:UniProtKB/TrEMBL;Acc:F4HZQ2]	7675502	7675971	34.47
AT1G21866		Plant thionin family protein [Source:UniProtKB/TrEMBL;Acc:F4HZQ3]	7677499	7677959	33.19
AT1G21870	GONST5	GDP-mannose transporter GONST5 [Source:UniProtKB/Swiss-Prot;Acc:Q9SFE9]	7678208	7679843	36.92
AT1G21880	LYM1	LYP2 [Source:UniProtKB/TrEMBL;Acc:A0A178W9K9]	7680390	7682641	41.74
AT1G05687			7680822	7681663	41.81
AT1G21890		WAT1-related protein At1g21890 [Source:UniProtKB/Swiss-Prot;Acc:F4HZQ7]	7682584	7685662	35.69
AT1G21900		Transmembrane emp24 domain-containing protein p24delta5 [Source:UniProtKB/Swiss-Prot;Acc:Q8RWM6]	7690802	7692459	38.12
AT1G21910	ERF012	DREB26 [Source:UniProtKB/TrEMBL;Acc:A0A178W3Q9]	7696427	7697831	36.23
AT1G21920		Histone H3 K4-specific methyltransferase SET7/9 family protein [Source:UniProtKB/TrEMBL;Acc:Q9SFE3]	7704173	7706254	39.96
AT1G21925		Plant thionin family protein [Source:UniProtKB/TrEMBL;Acc:A7REE4]	7710423	7710725	37.29
AT1G21928		Plant thionin family protein [Source:UniProtKB/TrEMBL;Acc:A8MR37]	7712211	7712513	37.29
AT1G21930		At1g21930 [Source:UniProtKB/TrEMBL;Acc:Q84VW7]	7712819	7714459	36.26
AT1G21940		unknown protein; FUNCTIONS IN: molecular_function unknown; INVOLVED IN: biological_process unknown; LOCATEI	7715170	7716975	37.87
AT1G21950		T26F17.18 [Source:UniProtKB/TrEMBL;Acc:Q9SFE0]	7723162	7723954	41.49
AT1G21960		RING/U-box superfamily protein [Source:UniProtKB/TrEMBL;Acc:Q9SFD9]	7725972	7726586	42.11
AT1G21970	NFYB9	Nuclear transcription factor Y subunit B-9 [Source:UniProtKB/Swiss-Prot;Acc:Q9SFD8]	7727577	7729649	36.23
AT1G21980	PIP5K1	Phosphatidylinositol 4-phosphate 5-kinase 1 [Source:UniProtKB/Swiss-Prot;Acc:Q56YP2]	7734661	7738772	38.76
AT1G05693			7739696	7740012	36.59
AT1G05697			7739737	7739935	42.21
AT1G21990		Putative F-box protein At1g21990 [Source:UniProtKB/Swiss-Prot;Acc:Q9LM64]	7740530	7742106	43.25
AT1G22000		Putative F-box/FBD/LRR-repeat protein At1g22000 [Source:UniProtKB/Swiss-Prot;Acc:Q9LM63]	7744094	7745744	40.4
AT1G22010		F2E2.5 [Source:UniProtKB/TrEMBL;Acc:Q9LM61]	7749571	7750062	42.07
AT1G22015	B3GALT5	DD46 [Source:UniProtKB/TrEMBL;Acc:A0A384KFJ9]	7750994	7753519	35.31
AT1G22020	SHM6	Serine hydroxymethyltransferase 6 [Source:UniProtKB/Swiss-Prot;Acc:Q9LM59]	7754281	7758029	38.68
AT1G22030		BPS1-like protein [Source:UniProtKB/TrEMBL;Acc:Q9LM58]	7759077	7760929	40.47
AT1G22040		F-box/kelch-repeat protein At1g22040 [Source:UniProtKB/Swiss-Prot;Acc:Q9LM55]	7767924	7770216	40.08
AT1G22050	MUB6	Membrane-anchored ubiquitin-fold protein 6 [Source:UniProtKB/Swiss-Prot;Acc:Q8GWJ6]	7771659	7773154	33.56
AT1G05703			7772133	7772582	32.22
AT1G22060		LOCATED IN: vacuole; EXPRESSED IN: 23 plant structures; EXPRESSED DURING: 13 growth stages; BEST Arabidopsi	7773063	7781539	39.18
AT1G05707			7781197	7781712	36.05
AT1G22065		Putative uncharacterized protein [Source:UniProtKB/TrEMBL;Acc:Q1G3T6]	7785708	7786477	34.68
AT1G22070	TGA3	At1g22070 [Source:UniProtKB/TrEMBL;Acc:Q147Q9]	7789133	7792139	38.18
AT1G22080		Cysteine proteinases superfamily protein [Source:UniProtKB/TrEMBL;Acc:F4HZT2]	7792192	7795440	39.43
AT1G22090	EMB2204	UPF0725 protein EMB2204 [Source:UniProtKB/Swiss-Prot;Acc:Q9LM50]	7795708	7797425	38.47
AT1G05713			7797593	7797802	36.19
AT1G22100		Inositol-pentakisphosphate 2-kinase [Source:UniProtKB/TrEMBL;Acc:Q1PFT7]	7798023	7800228	36.31
AT1G22110		F2E2.18 [Source:UniProtKB/TrEMBL;Acc:Q9LM48]	7800507	7804061	33.84
AT1G22120		unknown protein; Ha. [Source:TAIR;Acc:AT1G22120]	7806309	7809632	31.53
AT1G22130	AGL104	Agamous-like MADS-box protein AGL104 [Source:UniProtKB/Swiss-Prot;Acc:Q9LM46]	7812211	7814345	34.24
AT1G05717			7814473	7814738	34.21
AT1G22140		At1g22140/F2E2_13 [Source:UniProtKB/TrEMBL;Acc:Q93Z58]	7814663	7815722	35.75
AT1G22150	SULTR1;3	Sulfate transporter 1.3 [Source:UniProtKB/Swiss-Prot;Acc:Q9FEP7]	7817609	7821576	38.23
AT1G22160	FLZ5	FCS-Like Zinc finger 5 [Source:UniProtKB/Swiss-Prot;Acc:Q8VY80]	7823066	7823971	38.08
AT1G22170		At1g22170 [Source:UniProtKB/TrEMBL;Acc:Q9LM13]	7826268	7828326	37.15
AT1G05723			7826380	7826621	34.3
AT1G22180		F16L1.9 protein [Source:UniProtKB/TrEMBL;Acc:Q9LM14]	7828326	7830424	35.97
AT1G05727			7829731	7830154	29.95
AT1G22190	RAP2-13	Ethylene-responsive transcription factor RAP2-13 [Source:UniProtKB/Swiss-Prot;Acc:Q9LM15]	7835781	7837411	38.75
AT1G22200		Endoplasmic reticulum vesicle transporter protein [Source:UniProtKB/TrEMBL;Acc:Q9LM16]	7837563	7840898	36.42
AT1G22210	TPPC	Probable trehalose-phosphate phosphatase C [Source:UniProtKB/Swiss-Prot;Acc:F4I1A6]	7841432	7844056	33.18
AT1G22220		AUF2 [Source:UniProtKB/TrEMBL;Acc:A0A178W7K5]	7846459	7847836	43.98
AT1G22230		Nucleolar GTP-binding protein [Source:UniProtKB/TrEMBL;Acc:Q5XVK1]	7849776	7851164	39.88
AT1G22240	APUM8	Putative pumilio homolog 8, chloroplastic [Source:UniProtKB/Swiss-Prot;Acc:Q9LM20]	7852904	7855213	37.97
AT1G22250		F16L1.2 protein [Source:UniProtKB/TrEMBL;Acc:Q9LM21]	7858864	7859766	36.32
AT1G05733			7858868	7859504	37.52
AT1G22260	ZYP1A	Synaptonemal complex protein 1 [Source:UniProtKB/Swiss-Prot;Acc:Q9LME2]	7859630	7865263	37.42
AT1G22270	TRM112A	Multifunctional methyltransferase subunit TRM112 homolog A [Source:UniProtKB/Swiss-Prot;Acc:Q8LFJ5]	7865435	7866176	42.45
AT1G22275	ZYP1B	Synaptonemal complex protein 2 [Source:UniProtKB/Swiss-Prot;Acc:P61430]	7867067	7872715	37.4
AT1G05737			7872850	7873056	35.75
AT1G22280	PAPP2C	Phytochrome-associated protein phosphatase type 2C [Source:UniProtKB/TrEMBL;Acc:F4I1B4]	7873867	7876018	35.08
AT1G22290		14-3-3 family protein [Source:UniProtKB/TrEMBL;Acc:F4I1B7]	7876640	7877962	38.1
AT1G22300	GRF10	GRF10 [Source:UniProtKB/TrEMBL;Acc:A0A178W0Z4]	7878759	7881395	35.99
AT1G22310	MBD8	Methyl-CpG-binding domain-containing protein 8 [Source:UniProtKB/Swiss-Prot;Acc:Q9LME6]	7881503	7883824	41.39
AT1G22320		pre-tRNA [Source:TAIR;Acc:AT1G22320]	7884370	7884454	60
AT1G22330		RNA-binding (RRM/RBD/RNP motifs) family protein [Source:UniProtKB/TrEMBL;Acc:Q9LME7]	7886323	7889534	35.43
AT1G22340	UGT85A7	UDP-glycosyltransferase 85A7 [Source:UniProtKB/Swiss-Prot;Acc:Q9LME8]	7890259	7892118	41.4
AT1G22360	UGT85A2	UDP-glycosyltransferase 85A2 [Source:UniProtKB/Swiss-Prot;Acc:Q9ZWJ3]	7894807	7897761	38.88
AT1G22370	UGT85A5	UDP-glycosyltransferase 85A5 [Source:UniProtKB/Swiss-Prot;Acc:Q9LMF0]	7897915	7900335	39.57
AT1G22380	UGT85A3	Glycosyltransferase (Fragment) [Source:UniProtKB/TrEMBL;Acc:W8PW16]	7900355	7902427	42.35
AT1G22400	UGT85A1	UDP-glycosyltransferase 85A1 [Source:UniProtKB/Swiss-Prot;Acc:Q9SK82]	7903660	7906969	36.74
AT1G22403		other RNA [Source:TAIR;Acc:AT1G22403]	7908568	7909708	39.35
AT1G22410		Phospho-2-dehydro-3-deoxyheptonate aldolase [Source:UniProtKB/TrEMBL;Acc:Q9SK84]	7911843	7915004	38.77
AT1G22420		F12K8.23 protein [Source:UniProtKB/TrEMBL;Acc:Q9SK85]	7916076	7917518	46.29
AT1G22430		Alcohol dehydrogenase-like 1 [Source:UniProtKB/Swiss-Prot;Acc:Q9SK86]	7919077	7921893	33.76
AT1G22440		Alcohol dehydrogenase-like 2 [Source:UniProtKB/Swiss-Prot;Acc:Q9SK87]	7922276	7925097	33.63
AT1G22450	COX6B-1	COX6B [Source:UniProtKB/TrEMBL;Acc:A0A178WPQ4]	7925121	7927345	36.72
AT1G22460	OFUT7	O-fucosyltransferase 7 [Source:UniProtKB/Swiss-Prot;Acc:B7ZWR7]	7927287	7930657	38.12
AT1G05747			7931629	7932056	35.75
AT1G22470		At1g22470/F12K8_18 [Source:UniProtKB/TrEMBL;Acc:Q8L443]	7932777	7933604	34.06
AT1G22480		At1g22480 [Source:UniProtKB/TrEMBL;Acc:Q9SK90]	7933973	7935382	40.21
AT1G22490		basic helix-loop-helix (bHLH) DNA-binding superfamily protein [Source:TAIR;Acc:AT1G22490]	7938195	7940805	35.81
AT1G22500	ATL15	E3 ubiquitin-protein ligase ATL15 [Source:UniProtKB/Swiss-Prot;Acc:Q9SK92]	7949476	7950900	46.46
AT1G05753			7949502	7950049	49.09
AT1G22510		E3 ubiquitin-protein ligase RNF170-like protein (DUF 1232) [Source:UniProtKB/TrEMBL;Acc:F4I1E4]	7950818	7952832	38.06
AT1G05757			7952142	7952470	35.26
AT1G22520		Domain of unknown function (DUF543) [Source:TAIR;Acc:AT1G22520]	7952974	7954546	38.46
AT1G22530	PATL2	Patellin-2 [Source:UniProtKB/Swiss-Prot;Acc:Q56ZI2]	7955445	7958564	42.24
AT1G22540	NPF5.10	Protein NRT1/ PTR FAMILY 5.10 [Source:UniProtKB/Swiss-Prot;Acc:Q0WP01]	7963813	7966436	42
AT1G22550	NPF5.16	Protein NRT1/ PTR FAMILY 5.16 [Source:UniProtKB/Swiss-Prot;Acc:Q9SK96]	7966500	7968692	40.67

	AT1G22570	NPF5.15	Protein NRT1/ PTR FAMILY 5.15 [Source:UniProtKB/Swiss-Prot;Acc:Q9SK99]	7976424	7978810	39.92
	AT1G22590	AGL87	AGAMOUS-like 87 [Source:UniProtKB/TrEMBL;Acc:Q7X9H1]	7981722	7984334	30.88
	AT1G22600		Late embryogenesis abundant protein (LEA) family protein [Source:UniProtKB/TrEMBL;Acc:Q9SKA2]	7987141	7989217	40.44
	AT1G22620	SAC1	ATSAC1 [Source:UniProtKB/TrEMBL;Acc:A0A178WK36]	7994166	8002989	41.18
	AT1G22610		C2 calcium/lipid-binding plant phosphoribosyltransferase family protein [Source:UniProtKB/TrEMBL;Acc:Q9SKA3]	7994262	7997588	45.15
	AT1G22630		At1g22630/F12K8_2 [Source:UniProtKB/TrEMBL;Acc:Q9SKA5]	8003223	8004304	31.89
	AT1G22640	MYB3	MYB3 [Source:UniProtKB/TrEMBL;Acc:A0A178WFN1]	8006064	8007465	37.23
	AT1G22650	INVD	Probable alkaline/neutral invertase D [Source:UniProtKB/Swiss-Prot;Acc:F4I2X9]	8013353	8016150	40.49
	AT1G22660		Polynucleotide adenylyltransferase family protein [Source:TAIR;Acc:AT1G22660]	8017281	8021930	38.88
	AT1G22670	RMR3	Receptor homology region, transmembrane domain- and RING domain-containing protein 3 [Source:UniProtKB/Swiss-Prot;A	8021835	8023516	42.39
	AT1G22680		T22J18.15 [Source:UniProtKB/TrEMBL;Acc:O80553]	8024967	8026356	36.69
	AT1G22690	GASA9	Gibberellin-regulated protein 9 [Source:UniProtKB/Swiss-Prot;Acc:Q8GWK5]	8027126	8028168	35.47
	AT1G22700	PYG7	Tetratricopeptide repeat domain-containing protein PYG7, chloroplastic [Source:UniProtKB/Swiss-Prot;Acc:B9DHG0]	8028158	8030136	38.45
	AT1G05763			8029507	8029778	37.5
	AT1G22710	SUC2	Sucrose transport protein SUC2 [Source:UniProtKB/Swiss-Prot;Acc:Q39231]	8030587	8033156	41.44
	AT1G22720		Protein kinase superfamily protein [Source:UniProtKB/TrEMBL;Acc:F4I2Z2]	8044232	8045665	32.78
	AT1G22730		MA3 domain-containing protein [Source:UniProtKB/TrEMBL;Acc:O80548]	8046073	8049039	39.6
	AT1G22740	RABG3B	Ras-related protein RABG3b [Source:UniProtKB/Swiss-Prot;Acc:O04157]	8049090	8050784	35.69
	AT1G22750		AT1G22750 protein [Source:UniProtKB/TrEMBL;Acc:O64379]	8050770	8052930	36.28
	AT1G22760	PAB3	Polyadenylate-binding protein 3 [Source:UniProtKB/Swiss-Prot;Acc:O64380]	8055325	8059060	38.41
	AT1G22770	GI	Protein GIGANTEA [Source:UniProtKB/Swiss-Prot;Acc:Q9SQI2]	8061751	8067790	41.54
	AT1G22780	RPS18C	40S ribosomal protein S18 [Source:UniProtKB/Swiss-Prot;Acc:P34788]	8067864	8069356	38.11
	AT1G22790		Low affinity potassium transport system protein [Source:UniProtKB/TrEMBL;Acc:Q8L8X4]	8069377	8071593	39.24
	AT1G22800		Putative methyltransferase At1g22800, mitochondrial [Source:UniProtKB/Swiss-Prot;Acc:O80543]	8071789	8074357	39.31
	AT1G05767			8071789	8075020	40.01
	AT1G22810	ERF019	Ethylene-responsive transcription factor ERF019 [Source:UniProtKB/Swiss-Prot;Acc:O80542]	8074231	8075037	40.4
	AT1G22830	PCMP-E24	Pentatricopeptide repeat-containing protein At1g22830 [Source:UniProtKB/Swiss-Prot;Acc:Q4V389]	8075996	8079137	39.12
	AT1G22840	CYTC-1	CYTC-1 [Source:UniProtKB/TrEMBL;Acc:A0A178W284]	8079287	8080574	38.28
	AT1G05773			8079512	8079995	33.88
	AT1G22850		At1g22850/F29G20_19 [Source:UniProtKB/TrEMBL;Acc:O23137]	8080482	8082882	39.73
	AT1G05777			8080698	8080937	36.25
	AT1G22860	VPS3	Vacuolar sorting protein 39 [Source:TAIR;Acc:AT1G22860]	8083184	8089273	39.1
	AT1G22870		Kinase family with ARM repeat domain-containing protein [Source:UniProtKB/TrEMBL;Acc:F4I313]	8089267	8094505	39.34
	AT1G22880	CEL5	Endoglucanase 3 [Source:UniProtKB/Swiss-Prot;Acc:Q2V4L8]	8095449	8097790	42.95
	AT1G22882	SUN3	SUN domain-containing protein 3 [Source:UniProtKB/Swiss-Prot;Acc:F4I316]	8098392	8101464	40.29
	AT1G22885		unknown protein; FUNCTIONS IN: molecular_function unknown; INVOLVED IN: biological_process unknown; LOCATEI	8101424	8102500	34.26
	AT1G22890		AT1G22890 protein [Source:UniProtKB/TrEMBL;Acc:A0JQ18]	8102728	8103588	32.06
	AT1G22900	DIR11	Dirigent protein 11 [Source:UniProtKB/Swiss-Prot;Acc:Q67YM6]	8103612	8104605	41.15
	AT1G22910		RNA-binding (RRM/RBD/RNP motifs) family protein [Source:UniProtKB/TrEMBL;Acc:F4I321]	8104993	8108361	37.61
	AT1G22920	CSN5A	COP9 signalosome complex subunit 5a [Source:UniProtKB/Swiss-Prot;Acc:Q8LAZ7]	8109574	8112061	40.19
	AT1G22930		T-complex protein 11 [Source:UniProtKB/TrEMBL;Acc:O23129]	8117286	8122151	39.85
	AT1G22940	TH1	Thiamine biosynthetic bifunctional enzyme TH1, chloroplastic [Source:UniProtKB/Swiss-Prot;Acc:Q5M731]	8122339	8125068	39.52
	AT1G22950		Uncharacterized PKHD-type hydroxylase At1g22950 [Source:UniProtKB/Swiss-Prot;Acc:Q3ED68]	8125023	8127286	37.94
	AT1G22960		Pentatricopeptide repeat-containing protein At1g22960, mitochondrial [Source:UniProtKB/Swiss-Prot;Acc:P0C7Q9]	8127442	8130456	40
	AT1G22970		At1g22970/F19G10_8 [Source:UniProtKB/TrEMBL;Acc:O23126]	8130598	8132435	38.08
	AT1G22980		unknown protein; BEST Arabidopsis thaliana protein match is: unknown protein (TAIR:AT1G22970.1); Ha. [Source:TAIR;A	8132396	8134388	38.53
	AT1G22985	ERF069	CRF7 [Source:UniProtKB/TrEMBL;Acc:A0A178W874]	8135180	8136034	40.94
	AT1G22990	HIPP22	HIPP22 [Source:UniProtKB/TrEMBL;Acc:A0A178WF89]	8139114	8140233	39.82
	AT1G23000		Heavy metal transport/detoxification superfamily protein [Source:TAIR;Acc:AT1G23000]	8143227	8145174	39.78
	AT1G23010	LPR1	Multicopper oxidase LPR1 [Source:UniProtKB/Swiss-Prot;Acc:F4I4K5]	8147118	8149891	40.12
	AT1G23020	FRO3	Ferric reduction oxidase 3, mitochondrial [Source:UniProtKB/Swiss-Prot;Acc:F4I4K7]	8149868	8153796	37.34
	AT1G23030	PUB11	RING-type E3 ubiquitin transferase [Source:UniProtKB/TrEMBL;Acc:A0A178WCR1]	8156504	8159185	41.16
	AT1G23037		F-box associated ubiquitination effector family protein [Source:UniProtKB/TrEMBL;Acc:F4I4K9]	8162414	8163149	40.76
	AT1G23040		At1g23040 [Source:UniProtKB/TrEMBL;Acc:O23120]	8164782	8166716	33.75
	AT1G23052			8168443	8169267	41.09
	AT1G23050		At1g23050 [Source:UniProtKB/TrEMBL;Acc:O23119]	8168443	8169267	41.09
	AT1G23060		MDP40 [Source:UniProtKB/TrEMBL;Acc:A0A384LAT5]	8170693	8173085	36.06
	AT1G23070		Protein LAZ1 homolog 2 [Source:UniProtKB/Swiss-Prot;Acc:Q5BPZ5]	8173823	8175819	38.06
	AT1G23080	PIN7	Auxin efflux carrier component 7 [Source:UniProtKB/Swiss-Prot;Acc:Q940Y5]	8180189	8183650	39.69
	AT1G23090	SULTR3;3	Probable sulfate transporter 3.3 [Source:UniProtKB/Swiss-Prot;Acc:Q9SXS2]	8184925	8189234	36.36
MQTL-5/Chr1	AT1G29100		Heavy metal transport/detoxification superfamily protein [Source:TAIR;Acc:AT1G29100]	10168717	10169916	36.33
	AT1G29110		Cysteine proteinases superfamily protein [Source:UniProtKB/TrEMBL;Acc:F4HZW2]	10171683	10173071	42.26
	AT1G29120		AT1G29120 protein [Source:UniProtKB/TrEMBL;Acc:C0Z2A9]	10174162	10178521	38.21
	AT1G29140		F28N24.16 protein [Source:UniProtKB/TrEMBL;Acc:Q9LP44]	10178902	10180225	36.71
	AT1G29150	RPN6	26S proteasome non-ATPase regulatory subunit 11 homolog [Source:UniProtKB/Swiss-Prot;Acc:Q9LP45]	10180302	10182808	41.2
	AT1G29160	DOF1.5	Uncharacterized protein At1g29160 (Fragment) [Source:UniProtKB/TrEMBL;Acc:C0SUY0]	10183390	10184512	39.8
	AT1G29170	SCAR3	Protein SCAR3 [Source:UniProtKB/Swiss-Prot;Acc:Q9LP46]	10190137	10195213	38.9
	AT1G29179		BEST Arabidopsis thaliana protein match is: Cysteine/Histidine-rich C1 domain family protein (TAIR:AT1G44030.1); Ha. [S	10198764	10200255	41.42
	AT1G29180		Cysteine/Histidine-rich C1 domain family protein [Source:UniProtKB/TrEMBL;Acc:F4HZX5]	10199628	10200962	40.07
	AT1G29195		At1g29190/F28N24_12 [Source:UniProtKB/TrEMBL;Acc:Q9LP48]	10202414	10203491	39.7
	AT1G29210		pre-tRNA [Source:TAIR;Acc:AT1G29210]	10207306	10207378	61.64
	AT1G29200	OFUT8	O-fucosyltransferase 8 [Source:UniProtKB/Swiss-Prot;Acc:F4HZX7]	10207577	10210622	37.33
	AT1G29220		Transcriptional regulator family protein [Source:UniProtKB/TrEMBL;Acc:F4HZY1]	10210520	10212722	38.49
	AT1G29230	CIPK18	Non-specific serine/threonine protein kinase [Source:UniProtKB/TrEMBL;Acc:A0A178WD98]	10214681	10216563	45.03
	AT1G29240		F28N24.8 protein [Source:UniProtKB/TrEMBL;Acc:Q9LP52]	10216779	10219942	38.81
	AT1G29250		Alba DNA/RNA-binding protein [Source:UniProtKB/TrEMBL;Acc:Q9LP53]	10223266	10224799	35.2
	AT1G29260	PEX7	Peroxisome biogenesis protein 7 [Source:UniProtKB/Swiss-Prot;Acc:Q9XF57]	10224815	10226322	40.25
	AT1G29265	MIR399A	MIR399A; miRNA [Source:TAIR;Acc:AT1G29265]	10227073	10227195	35.77
	AT1G29270		unknown protein; BEST Arabidopsis thaliana protein match is: unknown protein (TAIR:AT2G40435.1); Ha. [Source:TAIR;A	10229808	10231384	32.4
	AT1G29280	WRKY65	At1g29280 [Source:UniProtKB/TrEMBL;Acc:Q0V866]	10236337	10237990	38.88
	AT1G29290	CEP14	Precursor of CEP14 [Source:UniProtKB/Swiss-Prot;Acc:Q52K95]	10244453	10245874	33.05
	AT1G29300	UNE1	F28N24.3 protein [Source:UniProtKB/TrEMBL;Acc:Q9LP57]	10247707	10249704	41.64
	AT1G29310		SecY protein transport family protein [Source:UniProtKB/TrEMBL;Acc:Q8RWJ5]	10252241	10254792	39.85
	AT1G29320		Transducin/WD40 repeat-like superfamily protein [Source:TAIR;Acc:AT1G29320]	10255250	10258343	38.36
	AT1G29330	ERD2A	ER lumen protein-retaining receptor A [Source:UniProtKB/Swiss-Prot;Acc:P35402]	10258248	10261232	37.49
	AT1G29340	PUB17	RING-type E3 ubiquitin transferase [Source:UniProtKB/TrEMBL;Acc:A0A178WA68]	10264043	10266917	41.36
	AT1G29350		Kinase-related protein of unknown function (DUF1296) [Source:TAIR;Acc:AT1G29350]	10268421	10273821	39.25
	AT1G29357		other RNA [Source:TAIR;Acc:AT1G29357]	10273931	10275765	41.63
	AT1G29355		unknown protein; Ha. [Source:TAIR;Acc:AT1G29355]	10274048	10275539	41.96
	AT1G29370		RNA polymerase II degradation factor-like protein (DUF1296) [Source:UniProtKB/TrEMBL;Acc:Q8VZT4]	10277748	10283225	39.07
	AT1G29380		Beta-1,3 glucanase [Source:UniProtKB/TrEMBL;Acc:Q6DST9]	10283396	10286164	39.36
	AT1G29390	COR413IM2	COR413IM2 [Source:UniProtKB/TrEMBL;Acc:A0A178WEF2]	10286067	10288103	38.59
	AT1G29395	COR413IM1	Cold-regulated 413 inner membrane protein 1, chloroplastic [Source:UniProtKB/Swiss-Prot;Acc:Q94AL8]	10288129	10289666	39.14
	AT1G29400	ML5	Protein MEI2-like 5 [Source:UniProtKB/Swiss-Prot;Acc:Q8VWF5]	10289998	10294630	38.4
	AT1G29410	PAI3	N-(5'-phosphoribosyl)anthranilate isomerase 3, chloroplastic [Source:UniProtKB/Swiss-Prot;Acc:Q8LPI9]	10297750	10300073	39.46
	AT1G06227			10300193	10300716	34.16
	AT1G06233			10300351	10300453	37.86
	AT1G06237			10300489	10300565	37.66

AT1G06243			10300619	10300699	40.74
AT1G29418		unknown protein; FUNCTIONS IN: molecular_function unknown; INVOLVED IN: biological_process unknown; LOCATEI	10300631	10301302	33.93
AT1G06247			10300749	10300834	31.4
AT1G06253			10300882	10301021	37.14
AT1G29420		SAUR-like auxin-responsive protein family [Source:TAIR;Acc:AT1G29420]	10301377	10302099	37.48
AT1G06257			10301646	10301987	40.06
AT1G29430	SAUR62	Auxin-responsive protein SAUR62 [Source:UniProtKB/Swiss-Prot;Acc:Q9C7Q8]	10302585	10303418	36.21
AT1G29435			10303890	10305584	35.34
AT1G29440	SAUR63	Auxin-responsive protein SAUR63 [Source:UniProtKB/Swiss-Prot;Acc:F4I1H5]	10304507	10305108	37.54
AT1G29450	SAUR64	Auxin-responsive protein SAUR64 [Source:UniProtKB/Swiss-Prot;Acc:Q0V7Z5]	10305553	10306595	37.01
AT1G29460		SAUR-like auxin-responsive protein family [Source:TAIR;Acc:AT1G29460]	10307352	10308246	35.64
AT1G29465		Putative uncharacterized protein [Source:UniProtKB/TrEMBL;Acc:Q0WQY3]	10308520	10309896	35.37
AT1G06263			10308751	10308840	42.22
AT1G06267			10308881	10308957	40.26
AT1G06273			10309011	10309092	41.46
AT1G06277			10309131	10309220	32.22
AT1G06283			10309265	10309404	37.86
AT1G29470		Probable methyltransferase PMT24 [Source:UniProtKB/Swiss-Prot;Acc:Q6NPR7]	10310131	10314147	40.05
AT1G29480		Uncharacterized protein F15D2.6 [Source:UniProtKB/TrEMBL;Acc:Q9C7Q3]	10317936	10318711	38.79
AT1G29490	SAUR68	Auxin-responsive protein SAUR68 [Source:UniProtKB/Swiss-Prot;Acc:Q29Q96]	10319732	10320402	36.66
AT1G29500	SAUR66	Auxin-responsive protein SAUR66 [Source:UniProtKB/Swiss-Prot;Acc:Q9C7Q1]	10321011	10321978	36.16
AT1G29510	SAUR67	Auxin-responsive protein SAUR67 [Source:UniProtKB/Swiss-Prot;Acc:F4I1I4]	10322423	10323239	36.84
AT1G29520		AWPM-19-like family protein [Source:UniProtKB/TrEMBL;Acc:Q9C7P9]	10323636	10324826	40.89
AT1G29530		At1g29530 [Source:UniProtKB/TrEMBL;Acc:Q6NL08]	10324745	10326848	37.79
AT1G29535		Uncharacterized protein (Fragment) [Source:UniProtKB/TrEMBL;Acc:A0A1P8AUY5]	10327138	10329116	37.49
AT1G29540		LOW protein: protein BOBBER-like protein [Source:UniProtKB/TrEMBL;Acc:Q5BPY8]	10327346	10328105	40.53
AT1G06287			10328819	10329121	41.25
AT1G29550	EIF4E3	Eukaryotic translation initiation factor 4E-3 [Source:UniProtKB/Swiss-Prot;Acc:Q9C7P6]	10330425	10332326	38.91
AT1G29560		Zinc finger C-x8-C-x5-C-x3-H type family protein [Source:UniProtKB/TrEMBL;Acc:B3H4U9]	10332371	10335714	39.03
AT1G29570		Putative zinc finger CCCH domain-containing protein 9 [Source:UniProtKB/Swiss-Prot;Acc:Q9C7P4]	10335910	10337854	38.71
AT1G29580		Mediator of RNA polymerase II transcription subunit [Source:UniProtKB/TrEMBL;Acc:Q9C7P3]	10338443	10339146	35.51
AT1G29590	EIF4E2	Eukaryotic translation initiation factor 4E-2 [Source:UniProtKB/Swiss-Prot;Acc:Q9C7P2]	10339992	10341832	39.65
AT1G29600		Zinc finger C-x8-C-x5-C-x3-H type family protein [Source:TAIR;Acc:AT1G29600]	10343785	10346364	36.59
AT1G29620		Cytochrome C oxidase polypeptide VIB family protein [Source:UniProtKB/TrEMBL;Acc:Q9C7N9]	10347363	10349017	37.34
AT1G29630	EXO1	Exonuclease 1 [Source:UniProtKB/Swiss-Prot;Acc:Q8L6Z7]	10349427	10353833	37.92
AT1G29640		At1g29640 [Source:UniProtKB/TrEMBL;Acc:Q9C7N7]	10355638	10356482	36.57
AT1G29660		GDSL esterase/lipase At1g29660 [Source:UniProtKB/Swiss-Prot;Acc:Q9C7N5]	10371675	10374005	36.64
AT1G29670		GDSL-like Lipase/Acylhydrolase superfamily protein [Source:TAIR;Acc:AT1G29670]	10375599	10377871	37.31
AT1G29680	OBAP2C	Oil body-associated protein 2C [Source:UniProtKB/Swiss-Prot;Acc:Q9C7N3]	10377790	10378961	43.17
AT1G29690	CAD1	MACPF domain-containing protein CAD1 [Source:UniProtKB/Swiss-Prot;Acc:Q9C7N2]	10379020	10382153	40.56
AT1G06293			10383999	10384275	38.63
AT1G29700		Metallo-hydrolase/oxidoreductase superfamily protein [Source:UniProtKB/TrEMBL;Acc:Q9C535]	10384945	10387441	37.36
AT1G29710	PCMP-H67	Pentatricopeptide repeat-containing protein At1g29710, mitochondrial [Source:UniProtKB/Swiss-Prot;Acc:Q9C6G2]	10387514	10389286	40.38
AT1G29720	RFK1	Probable LRR receptor-like serine/threonine-protein kinase At1g29720 [Source:UniProtKB/Swiss-Prot;Acc:Q9ASQ6]	10393659	10399873	36.57
AT1G29730		Leucine-rich repeat transmembrane protein kinase [Source:TAIR;Acc:AT1G29730]	10400564	10405913	37.93
AT1G29740		Leucine-rich repeat transmembrane protein kinase [Source:UniProtKB/TrEMBL;Acc:F4I337]	10407220	10413119	37.34
AT1G29750	RKF1	Probable LRR receptor-like serine/threonine-protein kinase RFK1 [Source:UniProtKB/Swiss-Prot;Acc:Q9FXF2]	10413853	10420774	36.91
AT1G29760	SEI2	Seipin-2 [Source:UniProtKB/Swiss-Prot;Acc:F4I340]	10422292	10424513	40.5
AT1G29770		At1g29770 [Source:UniProtKB/TrEMBL;Acc:Q9FXF4]	10424463	10425801	41
AT1G29775			10425622	10426947	33.26
AT1G29785		other RNA [Source:TAIR;Acc:AT1G29785]	10426779	10429143	38.73
AT1G29780		At1g29780 [Source:UniProtKB/TrEMBL;Acc:A2RVS1]	10426950	10427615	43.54
AT1G29790		At1g29790 [Source:UniProtKB/TrEMBL;Acc:Q8RWB7]	10429718	10432123	41.02
AT1G29800		RING/FYVE/PHD-type zinc finger family protein [Source:UniProtKB/TrEMBL;Acc:Q84WW7]	10432599	10435441	39.82
AT1G29810	PDL2	Pterin-4-alpha-carbinolamine dehydratase 2, mitochondrial [Source:UniProtKB/Swiss-Prot;Acc:Q6QJ72]	10435374	10437558	37.53
AT1G29820		Magnesium transporter CorA-like family protein [Source:UniProtKB/TrEMBL;Acc:F4I348]	10437657	10440786	38.15
AT1G29830		Magnesium transporter CorA-like family protein [Source:UniProtKB/TrEMBL;Acc:F4I350]	10441053	10445657	35.87
AT1G29840		alpha/beta-Hydrolases superfamily protein [Source:TAIR;Acc:AT1G29840]	10445619	10447753	36.72
AT1G29850		double-stranded DNA-binding family protein [Source:TAIR;Acc:AT1G29850]	10447347	10449969	35.42
AT1G29860	WRKY71	WRKY transcription factor 71 [Source:UniProtKB/Swiss-Prot;Acc:Q93WV4]	10454433	10455874	36.82
AT1G29870		Putative glycine--tRNA ligase, cytoplasmic [Source:UniProtKB/Swiss-Prot;Acc:Q9FXG2]	10456902	10458782	41.36
AT1G29880		Glycine--tRNA ligase, mitochondrial 1 [Source:UniProtKB/Swiss-Prot;Acc:O23627]	10459457	10462967	40.47
AT1G29890		O-acetyltransferase family protein [Source:TAIR;Acc:AT1G29890]	10463029	10467452	37.41
AT1G29900	CARB	Carbamoyl-phosphate synthase large chain, chloroplastic [Source:UniProtKB/Swiss-Prot;Acc:Q42601]	10467956	10472211	43.59
AT1G29910	LHCB1.1	Chlorophyll a-b binding protein 3, chloroplastic [Source:UniProtKB/Swiss-Prot;Acc:Q8VZ87]	10472280	10473502	47.67
AT1G29920	LHCB1.1	Chlorophyll a-b binding protein 3, chloroplastic [Source:UniProtKB/Swiss-Prot;Acc:Q8VZ87]	10474656	10475969	45.89
AT1G29930	LHCB1.3	Chlorophyll a-b binding protein 1, chloroplastic [Source:UniProtKB/Swiss-Prot;Acc:P04778]	10477885	10479114	47.48
AT1G29940	NRPA2	DNA-directed RNA polymerase subunit beta [Source:UniProtKB/TrEMBL;Acc:A0A178W5B4]	10479007	10486824	37.08
AT1G06297			10489782	10490022	43.98
AT1G06303			10489782	10490022	43.98
AT1G29950	BHLH144	Transcription factor bHLH144 [Source:UniProtKB/Swiss-Prot;Acc:Q9ASX9]	10491698	10494598	37.09
AT1G29960		Peptidase S24/S26A/S26B/S26C family protein [Source:UniProtKB/TrEMBL;Acc:Q67XF2]	10494627	10496205	36.92
AT1G29962	AGL64	AGAMOUS-like 64 [Source:UniProtKB/TrEMBL;Acc:Q7XJK9]	10496730	10497287	44.8
AT1G29965	RPL18AA	60S ribosomal protein L18a-1 [Source:UniProtKB/Swiss-Prot;Acc:Q8L7K0]	10498310	10499462	39.55
AT1G29970	RPL18AA	60S ribosomal protein L18A-1 [Source:UniProtKB/TrEMBL;Acc:A8MRF3]	10498351	10501498	38.18
AT1G29980		Uncharacterized protein T1P2.9 [Source:UniProtKB/TrEMBL;Acc:Q9C8S1]	10503239	10506189	39.04
AT1G29990	PFD6	At1g29990 [Source:UniProtKB/TrEMBL;Acc:Q2HIK4]	10507533	10509151	38.11
AT1G30000	MNS3	alpha-mannosidase 3 [Source:TAIR;Acc:AT1G30000]	10509156	10512558	38.79
AT1G30010	NMAT1	Nuclear intron maturase 1, mitochondrial [Source:UniProtKB/Swiss-Prot;Acc:Q9C8R8]	10513151	10516385	40.49
AT1G30020		Uncharacterized protein T1P2.11 [Source:UniProtKB/TrEMBL;Acc:Q9C8R7]	10515874	10516347	41.56
AT1G30040	GA2OX2	Gibberellin 2-beta-dioxygenase 2 [Source:UniProtKB/Swiss-Prot;Acc:Q9XFR9]	10537457	10540044	36.82
AT1G30050		Tropomyosin [Source:UniProtKB/TrEMBL;Acc:Q9C8R6]	10543022	10544594	40.5
AT1G30060		COP1-interacting protein-like protein [Source:UniProtKB/TrEMBL;Acc:Q9C8R5]	10545087	10545906	38.29
AT1G30070		SGS domain-containing protein [Source:UniProtKB/TrEMBL;Acc:F4I4Q9]	10546511	10548236	36.96
AT1G30080		Glycosyl hydrolase superfamily protein [Source:UniProtKB/TrEMBL;Acc:F4I4R0]	10550855	10553295	37.2
AT1G30090		F-box/kelch-repeat protein At1g30090 [Source:UniProtKB/Swiss-Prot;Acc:Q9C6Z0]	10559496	10561545	40.1
AT1G30100	NCED5	Probable 9-cis-epoxycarotenoid dioxygenase NCED5, chloroplastic [Source:UniProtKB/Swiss-Prot;Acc:Q9C6Z1]	10571310	10573365	45.57
AT1G30110	NUDT25	Nudix hydrolase 25 [Source:UniProtKB/Swiss-Prot;Acc:Q9C6Z2]	10581735	10584152	33.66
AT1G30120	PDH-E1 BETA	Pyruvate dehydrogenase E1 component subunit beta-2, chloroplastic [Source:UniProtKB/Swiss-Prot;Acc:Q9C6Z3]	10584105	10586680	40.3
AT1G30130		AT1G30130 protein [Source:UniProtKB/TrEMBL;Acc:Q9C6Z4]	10587471	10590209	35.74
AT1G06307			10590656	10590900	31.02
AT1G30135	TIFY5A	Protein TIFY 5A [Source:UniProtKB/Swiss-Prot;Acc:Q8LBM2]	10596352	10597341	32.02
AT1G30140		Myb/SANT-like DNA-binding domain protein [Source:UniProtKB/TrEMBL;Acc:Q6DYE3]	10598542	10599696	32.47
AT1G30160		At1g30160 [Source:UniProtKB/TrEMBL;Acc:Q6DBP6]	10606159	10607944	40.65
AT1G30170		Uncharacterized protein T2H7.3 [Source:UniProtKB/TrEMBL;Acc:Q9C6Z8]	10607888	10609618	41.13
AT1G30190		Cotton fiber protein [Source:UniProtKB/TrEMBL;Acc:Q9C6Z9]	10618893	10620067	39.32
AT1G30200		F-box protein At1g30200 [Source:UniProtKB/Swiss-Prot;Acc:Q9C534]	10625000	10627135	42.6
AT1G30210	TCP24	TCP24 [Source:UniProtKB/TrEMBL;Acc:A0A178W971]	10627185	10630630	34.76

AT1G30220	INT2	Probable inositol transporter 2 [Source:UniProtKB/Swiss-Prot;Acc:Q9C757]	10632720	10635581	38.36
AT1G30230		Translation elongation factor EF1B/ribosomal protein S6 family protein [Source:UniProtKB/TrEMBL;Acc:A8MRC4]	10638878	10640941	37.89
AT1G30240		Proline-, glutamic acid/leucine-rich protein [Source:UniProtKB/TrEMBL;Acc:Q0WV78]	10641031	10646195	38.22
AT1G30250		At1g30250 [Source:UniProtKB/TrEMBL;Acc:Q66GS7]	10646842	10647650	36.59
AT1G30260		At1g30260/F12P21_9 [Source:UniProtKB/TrEMBL;Acc:Q9C754]	10650914	10651829	34.61
AT1G30270	CIPK23	CBL-interacting serine/threonine-protein kinase 23 [Source:UniProtKB/Swiss-Prot;Acc:Q93VD3]	10654800	10658924	37.28
AT1G30280		Chaperone DnaJ-domain superfamily protein [Source:UniProtKB/TrEMBL;Acc:Q9C752]	10662608	10664668	41.63
AT1G30282		other RNA [Source:TAIR;Acc:AT1G30282]	10663332	10664763	43.78
AT1G06313			10667624	10668129	43.28
AT1G06317			10667813	10668056	43.85
AT1G30290		Tetratricopeptide repeat (TPR)-like superfamily protein [Source:TAIR;Acc:AT1G30290]	10670129	10672836	41.91
AT1G30300		At1g30300 [Source:UniProtKB/TrEMBL;Acc:Q6NNG9]	10672973	10675455	36.93
AT1G06323			10677329	10677671	28.28
AT1G30320		Remorin family protein [Source:UniProtKB/TrEMBL;Acc:Q9C8G3]	10680079	10683137	38.54
AT1G30330	ARF6	Auxin response factor 6 [Source:UniProtKB/Swiss-Prot;Acc:Q9ZTX8]	10685822	10690988	41.24
AT1G30350		Probable pectate lyase 4 [Source:UniProtKB/Swiss-Prot;Acc:Q9C8G4]	10709999	10711667	38.23
AT1G30360	ERD4	Hyperosmolality-gated Ca2+ permeable channel 3.1 [Source:UniProtKB/TrEMBL;Acc:A0A097NUQ9]	10715482	10718997	41.04
AT1G30370		DLAH [Source:UniProtKB/TrEMBL;Acc:A0A178W2K8]	10719065	10720843	44.46
AT1G30380	PSAK	Photosystem I reaction center subunit psaK, chloroplastic [Source:UniProtKB/Swiss-Prot;Acc:Q9SUI5]	10722183	10723247	36.81
AT1G30400	ABCC1	ABC transporter C family member 1 [Source:UniProtKB/Swiss-Prot;Acc:Q9C8G9]	10727317	10738092	38.14
AT1G30410	ATMRP13	multidrug resistance-associated protein 13 [Source:TAIR;Acc:AT1G30410]	10738683	10747563	37.72
AT1G30420	ABCC11	ABC transporter C family member 11 [Source:UniProtKB/Swiss-Prot;Acc:Q9C8H1]	10748261	10756695	38.09
AT1G30430		pre-tRNA [Source:TAIR;Acc:AT1G30430]	10756722	10756794	61.64
AT1G30440		BTB/POZ domain-containing protein At1g30440 [Source:UniProtKB/Swiss-Prot;Acc:Q9S9Q9]	10758743	10762429	39.73
AT1G30450	CCC1	Cation-chloride cotransporter 1 [Source:UniProtKB/Swiss-Prot;Acc:Q2UVJ5]	10762708	10769616	38.92
AT1G30455		Cyclin/Brf1-like TBP-binding domain-containing protein [Source:UniProtKB/TrEMBL;Acc:F4I4V4]	10769722	10770775	41.94
AT1G30460	CPSF30	30-kDa cleavage and polyadenylation specificity factor 30 [Source:UniProtKB/Swiss-Prot;Acc:A9LNK9]	10770813	10775428	39.04
AT1G06327			10775823	10776027	42.44
AT1G06333			10775860	10776319	36.09
AT1G30470		SIT4 phosphatase-associated family protein [Source:UniProtKB/TrEMBL;Acc:F4I6B2]	10779111	10786697	37.18
AT1G30473		Heavy metal transport/detoxification superfamily protein [Source:UniProtKB/TrEMBL;Acc:Q1G3T0]	10786979	10788683	39
AT1G30475		AT1G30475 protein [Source:UniProtKB/TrEMBL;Acc:A8MQH6]	10788226	10790241	35.96
AT1G06337			10789698	10790032	31.94
AT1G30480	DRT111	DNA-damage-repair/toleration protein DRT111, chloroplastic [Source:UniProtKB/Swiss-Prot;Acc:P42698]	10790022	10792725	40.01
AT1G30490	ATHB-9	Homeobox-leucine zipper protein ATHB-9 [Source:UniProtKB/Swiss-Prot;Acc:O04292]	10796124	10801093	39.54
AT1G06343			10803628	10803853	33.63
AT1G30500	NFYA7	Nuclear transcription factor Y subunit A-7 [Source:UniProtKB/Swiss-Prot;Acc:Q84JP1]	10804450	10806428	36.13
AT1G30510	RFNR2	Ferredoxin--NADP reductase, root isozyme 2, chloroplastic [Source:UniProtKB/Swiss-Prot;Acc:Q9S9P8]	10806930	10809188	39.49
AT1G30515		At1g30515 [Source:UniProtKB/TrEMBL;Acc:Q8L8T4]	10809200	10809939	39.46
AT1G30520	AAE14	2-succinylbenzoate--CoA ligase, chloroplastic/peroxisomal [Source:UniProtKB/Swiss-Prot;Acc:Q8VYJ1]	10810397	10813779	40.5
AT1G06347			10814090	10814376	35.19
AT1G30530	UGT78D1	Glycosyltransferase (Fragment) [Source:UniProtKB/TrEMBL;Acc:W8PVA4]	10814612	10816615	41.92
AT1G30540		Actin-like ATPase superfamily protein [Source:UniProtKB/TrEMBL;Acc:Q8LGE0]	10816616	10819419	39.8
AT1G30545		S-adenosyl-L-methionine-dependent methyltransferase superfamily protein [Source:UniProtKB/TrEMBL;Acc:A0A1P8ASE3]	10819495	10822055	37.33
AT1G30550		S-adenosyl-L-methionine-dependent methyltransferases superfamily protein [Source:TAIR;Acc:AT1G30550]	10822161	10823903	33.28
AT1G30560		G3Pp3 [Source:UniProtKB/TrEMBL;Acc:A0A178WI18]	10824637	10826405	43.41
AT1G30570	HERK2	Probable receptor-like protein kinase At1g30570 [Source:UniProtKB/Swiss-Prot;Acc:Q9SA72]	10828373	10831656	41.6
AT1G30580	YchF1	Obg-like ATPase 1 [Source:UniProtKB/TrEMBL;Acc:A0A178WF69]	10831673	10835635	37.57
AT1G30590		RNA polymerase I specific transcription initiation factor RRN3 protein [Source:TAIR;Acc:AT1G30590]	10835735	10840872	37.91
AT1G30600	SBT2.1	Subtilisin-like protease SBT2.1 [Source:UniProtKB/Swiss-Prot;Acc:Q9SA75]	10841107	10845242	40.81
AT1G30610	EMB2279	Pentatricopeptide repeat-containing protein At1g30610, chloroplastic [Source:UniProtKB/Swiss-Prot;Acc:Q9SA76]	10846513	10850724	39.15
AT1G30620	MUR4	UXE1 [Source:UniProtKB/TrEMBL;Acc:A0A384K925]	10854517	10858280	37.51
AT1G30630		Coatomer subunit epsilon-1 [Source:UniProtKB/Swiss-Prot;Acc:Q9SA78]	10858232	10860322	38.31
AT1G30640		Non-specific serine/threonine protein kinase [Source:UniProtKB/TrEMBL;Acc:F4I6E4]	10860619	10865074	36.33
AT1G30650	WRKY14	Probable WRKY transcription factor 14 [Source:UniProtKB/Swiss-Prot;Acc:Q9SA80]	10868218	10871297	37.69
AT1G30660		Primase homolog protein [Source:UniProtKB/Swiss-Prot;Acc:F4I6E6]	10876724	10879143	35.79
AT1G30670	BHLH52	Transcription factor bHLH52 [Source:UniProtKB/Swiss-Prot;Acc:Q9SA82]	10879173	10880188	40.65
AT1G30680		Twinkle homolog protein, chloroplastic/mitochondrial [Source:UniProtKB/Swiss-Prot;Acc:B5X582]	10881479	10886472	38.07
AT1G30690	PATL4	Patellin-4 [Source:UniProtKB/Swiss-Prot;Acc:Q94C59]	10887607	10890407	40.16
AT1G30700		Berberine bridge enzyme-like 8 [Source:UniProtKB/Swiss-Prot;Acc:Q9SA85]	10892445	10894714	38.85
AT1G30710		Berberine bridge enzyme-like 9 [Source:UniProtKB/Swiss-Prot;Acc:Q9SA86]	10895252	10897127	42.64
AT1G30720		Berberine bridge enzyme-like 10 [Source:UniProtKB/Swiss-Prot;Acc:Q9SA87]	10897925	10899975	42.08
AT1G30730		Berberine bridge enzyme-like 11 [Source:UniProtKB/Swiss-Prot;Acc:Q9SA88]	10900681	10902626	43.17
AT1G30740		FAD-binding Berberine family protein [Source:TAIR;Acc:AT1G30740]	10902995	10904739	43.95
AT1G30750		TPRXL [Source:UniProtKB/TrEMBL;Acc:Q949S2]	10904775	10905724	38.53
AT1G30755		Elongation factor G, putative (DUF668) [Source:UniProtKB/TrEMBL;Acc:Q8L5Y3]	10905731	10909760	39.11
AT1G30757		Putative uncharacterized protein [Source:UniProtKB/TrEMBL;Acc:Q8LGA4]	10908980	10909784	37.27
AT1G30760		FAD-binding Berberine family protein [Source:TAIR;Acc:AT1G30760]	10918267	10920709	40.73
AT1G06353			10921835	10922072	44.96
AT1G30780		Probable F-box protein At1g30780 [Source:UniProtKB/Swiss-Prot;Acc:Q9SY17]	10923919	10926561	39.61
AT1G06357			10928005	10928930	42.22
AT1G06363			10928659	10928968	42.9
AT1G30790		F-box protein At1g30790 [Source:UniProtKB/Swiss-Prot;Acc:Q9SY20]	10932713	10934066	38.55
AT1G30795		At1g30795 [Source:UniProtKB/TrEMBL;Acc:Q9SY21]	10935684	10936396	37.73
AT1G30810	JMJ18	Lysine-specific demethylase JMJ18 [Source:UniProtKB/Swiss-Prot;Acc:F4I6G4]	10936765	10942292	38.8
AT1G30800		At1g30800 [Source:UniProtKB/TrEMBL;Acc:Q9SY22]	10936923	10938099	43.84
AT1G30814		unknown protein; Ha. [Source:TAIR;Acc:AT1G30814]	10942648	10944727	34.47
AT1G30820		CTP synthase [Source:UniProtKB/TrEMBL;Acc:F4I6G9]	10944908	10949482	37.57
AT1G06373			10954288	10954711	33.25
AT1G30825	ARPC2A	Actin-related protein 2/3 complex subunit 2A [Source:UniProtKB/Swiss-Prot;Acc:Q8LGI3]	10960908	10963231	36.45
AT1G30830		pre-tRNA [Source:TAIR;Acc:AT1G30830]	10963573	10963646	54.05
AT1G30840	PUP4	Probable purine permease 4 [Source:UniProtKB/Swiss-Prot;Acc:Q9SY29]	10974383	10976298	41.96
AT1G30845		Cell growth defect factor-2 [Source:UniProtKB/TrEMBL;Acc:Q3C1C7]	10979646	10980725	35.09
AT1G06377			10981462	10981784	43.65
AT1G30850	RSH4	Root hair specific 4 [Source:UniProtKB/TrEMBL;Acc:Q9SY31]	10985063	10986295	41.93
AT1G30860		At1g30860 [Source:UniProtKB/TrEMBL;Acc:Q6NQ80]	10986496	10989593	40.61
AT1G30870	PER7	Peroxidase 7 [Source:UniProtKB/Swiss-Prot;Acc:Q9SY33]	10991478	10993083	42.9
AT1G30880		At1g30880 [Source:UniProtKB/TrEMBL;Acc:Q9FYH5]	10993002	10994152	37.71
AT1G30890		Integral membrane HRF1 family protein [Source:UniProtKB/TrEMBL;Acc:Q94BQ9]	10994243	10996223	38.47
AT1G30900	VSR6	Vacuolar-sorting receptor 6 [Source:UniProtKB/Swiss-Prot;Acc:Q9FYH7]	10996870	11000809	36.02
AT1G30910		F17F8.22 [Source:UniProtKB/TrEMBL;Acc:Q9FYH8]	11000862	11003226	39.53
AT1G30920		Putative F-box protein At1g30920 [Source:UniProtKB/Swiss-Prot;Acc:P0C2G2]	11004084	11005444	38.72
AT1G30925		Putative F-box protein At1g30925 [Source:UniProtKB/Swiss-Prot;Acc:P0C2G3]	11009888	11012280	37.69
AT1G06383			11013511	11013942	42.13
AT1G30930		Putative F-box protein At1g30930 [Source:UniProtKB/Swiss-Prot;Acc:P0C2D0]	11014783	11015913	39.43
AT1G06387			11016492	11016886	36.46
AT1G06393			11032358	11032898	29.76
AT1G30950	UFO	Uncharacterized protein At1g30950 (Fragment) [Source:UniProtKB/TrEMBL;Acc:C0SUY4]	11035992	11037508	45.02

AT1G30960	ERG	GTP-binding protein ERG [Source:UniProtKB/Swiss-Prot;Acc:O82653]	11037552	11040084	38.77
AT1G30970	SUF4	zinc finger (C2H2 type) family protein [Source:TAIR;Acc:AT1G30970]	11040250	11043773	37.57
AT1G06397			11040576	11040721	43.84
AT1G30972		Plant thionin family protein [Source:UniProtKB/TrEMBL;Acc:B3H566]	11044628	11045466	29.8
AT1G30974		Plant thionin family protein [Source:UniProtKB/TrEMBL;Acc:F4I7U4]	11046969	11047816	30.42
AT1G30990		F17F8.9 [Source:UniProtKB/TrEMBL;Acc:Q9FYJ0]	11051999	11053295	35.31
AT1G31000		Putative F-box protein At1g31000 [Source:UniProtKB/Swiss-Prot;Acc:Q9FYJ1]	11053377	11054567	40.47
AT1G31010	OSB4	Protein OSB4, chloroplastic [Source:UniProtKB/Swiss-Prot;Acc:Q9FYJ2]	11054731	11056959	37.33
AT1G31020	ATO2	Thioredoxin O2, mitochondrial [Source:UniProtKB/Swiss-Prot;Acc:Q93VQ9]	11057105	11059037	36.21
AT1G06403			11061425	11061700	42.39
AT1G31040		PLATZ transcription factor family protein [Source:UniProtKB/TrEMBL;Acc:F4I7U9]	11069510	11072564	31.55
AT1G31050		basic helix-loop-helix (bHLH) DNA-binding superfamily protein [Source:TAIR;Acc:AT1G31050]	11075398	11079289	32.86
AT1G31070	GLCNAC1PUT1	GlcNAc1pUT1 [Source:UniProtKB/TrEMBL;Acc:A0A178W850]	11084666	11088589	35.81
AT1G31080		F-box protein At1g31080 [Source:UniProtKB/Swiss-Prot;Acc:Q9SA02]	11091832	11092899	40.64
AT1G31090		Putative F-box protein At1g31090 [Source:UniProtKB/Swiss-Prot;Acc:Q9SA03]	11094407	11095649	39.42
AT1G06407			11102314	11102804	41.34
AT1G31095		Lactate/malate dehydrogenase, NAD-binding domain protein [Source:UniProtKB/TrEMBL;Acc:A0A1P8ARI1]	11102778	11103644	33.56
AT1G31110		pre-tRNA [Source:TAIR;Acc:AT1G31110]	11104113	11104186	51.35
AT1G31120	POT10	Potassium transporter 10 [Source:UniProtKB/Swiss-Prot;Acc:Q9SA05]	11104237	11108095	40.04
AT1G31130		F28K20.6 protein [Source:UniProtKB/TrEMBL;Acc:Q9SA06]	11114693	11116195	37.72
AT1G31140	AGL63	GOA [Source:UniProtKB/TrEMBL;Acc:A0A178W639]	11117941	11119785	32.41
AT1G31150		F28K20.8 protein [Source:UniProtKB/TrEMBL;Acc:Q9SA08]	11119827	11122675	39.7
AT1G31160	HINT 2	HISTIDINE TRIAD NUCLEOTIDE-BINDING 2 [Source:UniProtKB/TrEMBL;Acc:Q8GYJ9]	11122604	11124220	38.9
AT1G31163		F-box associated ubiquitination effector family protein [Source:UniProtKB/TrEMBL;Acc:F4I7W1]	11128732	11130239	38.33
AT1G31170	ATSRX	Sulfiredoxin [Source:UniProtKB/TrEMBL;Acc:F4I7W2]	11133410	11135189	37.64
AT1G06413			11136187	11136397	36.49
AT1G31173	MIR167D	MIR167D; miRNA [Source:TAIR;Acc:AT1G31173]	11137539	11137915	35.28
AT1G06417			11139772	11140052	30.25
AT1G31175		At1g31175 [Source:UniProtKB/TrEMBL;Acc:Q9SA13]	11140471	11142716	40.87
AT1G31180	ATIMD3	isopropylmalate dehydrogenase 3 [Source:TAIR;Acc:AT1G31180]	11142461	11144717	39.34
AT1G31190	IMPL1	Phosphatase IMPL1, chloroplastic [Source:UniProtKB/Swiss-Prot;Acc:Q94F00]	11144772	11146930	39.56
AT1G31200	ATPP2-A9	phloem protein 2-A9 [Source:TAIR;Acc:AT1G31200]	11146923	11147547	41.92
AT1G06423			11147007	11147252	44.31
AT1G31220	PUR3	Phosphoribosylglycinamide formyltransferase, chloroplastic [Source:UniProtKB/Swiss-Prot;Acc:P52422]	11156698	11158683	39.17
AT1G31230	AKHSDH1	Bifunctional aspartokinase/homoserine dehydrogenase 1, chloroplastic [Source:UniProtKB/Swiss-Prot;Acc:Q9SA18]	11158618	11163274	39.98
AT1G31240		At1g31240 [Source:UniProtKB/TrEMBL;Acc:Q9SA19]	11163858	11164928	46.13
AT1G31243			11164639	11165022	46.61
AT1G31250		At1g31250 [Source:UniProtKB/TrEMBL;Acc:Q58CN2]	11166557	11167547	37.34
AT1G31255			11169450	11170396	34
AT1G06427			11169645	11170059	38.31
AT1G31258		other RNA [Source:TAIR;Acc:AT1G31258]	11171225	11171835	34.86
AT1G31260	ZIP10	Probable zinc transporter 10 [Source:UniProtKB/Swiss-Prot;Acc:Q8W245]	11175559	11177362	38.36
AT1G31270		unknown protein; Ha. [Source:TAIR;Acc:AT1G31270]	11178349	11179203	40.12
AT1G31280	AGO2	Protein argonaute 2 [Source:UniProtKB/Swiss-Prot;Acc:Q9SHF3]	11181504	11185396	43.57
AT1G31290	AGO3	Protein argonaute 3 [Source:UniProtKB/Swiss-Prot;Acc:Q9SHF2]	11188120	11192585	43.8
AT1G31300		At1g31300/T19E23_12 [Source:UniProtKB/TrEMBL;Acc:Q93Z82]	11193625	11196397	36.31
AT1G06433			11197612	11198128	36.94
AT1G31310		Hydroxyproline-rich glycoprotein family protein [Source:UniProtKB/TrEMBL;Acc:F4I9C1]	11198353	11200141	40.86
AT1G31320	LBD4	LOB domain-containing protein 4 [Source:UniProtKB/Swiss-Prot;Acc:Q9SHE9]	11212958	11214567	38.76
AT1G31330	PSAF	Photosystem I reaction center subunit III, chloroplastic [Source:UniProtKB/Swiss-Prot;Acc:Q9SHE8]	11214676	11216141	42.36
AT1G31335		At1g31335 [Source:UniProtKB/TrEMBL;Acc:Q8LG39]	11216810	11217546	38.13
AT1G31340	RUB1	RUB1 [Source:UniProtKB/TrEMBL;Acc:A0A178W1F2]	11217695	11219618	36.43
AT1G31350	KUF1	KAR-UP F-box 1 [Source:TAIR;Acc:AT1G31350]	11221267	11222827	43.5
AT1G31358	MIR404	MIR404; miRNA [Source:TAIR;Acc:AT1G31358]	11230463	11230612	59.33
AT1G31360	RECQL2	RECQ helicase L2 [Source:TAIR;Acc:AT1G31360]	11232318	11237594	37.84
AT1G31370		Ubiquitin-specific protease family C19-related protein [Source:UniProtKB/TrEMBL;Acc:Q9C872]	11238298	11239363	39.59
AT1G31380		TRAF-like family protein [Source:UniProtKB/TrEMBL;Acc:Q9C871]	11240451	11241099	39.29
AT1G06437			11241229	11241474	38.62
AT1G31390		TRAF-like family protein [Source:TAIR;Acc:AT1G31390]	11243182	11244392	38.4
AT1G31400		MATH domain and coiled-coil domain-containing protein At1g31400 [Source:UniProtKB/Swiss-Prot;Acc:Q9C869]	11245225	11246561	38.74
AT1G31410		At1g31410 [Source:UniProtKB/TrEMBL;Acc:Q66GJ0]	11246817	11249587	41.72
AT1G31420	FEI1	Leucine-rich repeat protein kinase family protein [Source:UniProtKB/TrEMBL;Acc:F4I9D5]	11249600	11253915	37.72
AT1G31430	PCMP-E55	Pentatricopeptide repeat-containing protein At1g31430 [Source:UniProtKB/Swiss-Prot;Acc:Q9C866]	11253912	11255745	40.35
AT1G31440	SH3P1	SH3 domain-containing protein 1 [Source:UniProtKB/Swiss-Prot;Acc:Q9C865]	11255836	11258814	37.19
AT1G31450		Aspartyl protease family protein [Source:UniProtKB/TrEMBL;Acc:Q9C864]	11259851	11261234	45.74
AT1G31460		At1g31460 [Source:UniProtKB/TrEMBL;Acc:Q9C863]	11261443	11263364	38.35
AT1G31470	NFD4	Protein NUCLEAR FUSION DEFECTIVE 4 [Source:UniProtKB/Swiss-Prot;Acc:F4I9E1]	11262918	11264963	42.77
AT1G31480	SGR2	Phospholipase SGR2 [Source:UniProtKB/Swiss-Prot;Acc:Q8W5R2]	11265816	11271701	37.84
AT1G31490		HXXXD-type acyl-transferase family protein [Source:UniProtKB/TrEMBL;Acc:Q9C564]	11271228	11273294	41.46
AT1G31485		other RNA [Source:TAIR;Acc:AT1G31485]	11271744	11273075	45.2
AT1G31500		DNAse I-like superfamily protein [Source:TAIR;Acc:AT1G31500]	11273573	11276636	35.74
AT1G31510		F-box associated ubiquitination effector family protein [Source:UniProtKB/TrEMBL;Acc:Q9C6U5]	11277417	11278462	38.34
AT1G31520		Uncharacterized protein F27M3_25 [Source:UniProtKB/TrEMBL;Acc:Q9C6U6]	11278983	11279639	41.25
AT1G31530		DNAse I-like superfamily protein [Source:UniProtKB/TrEMBL;Acc:Q9C6U7]	11281188	11282468	36.53
AT1G31540		Disease resistance protein (TIR-NBS-LRR class) family [Source:UniProtKB/TrEMBL;Acc:F4I9F1]	11288378	11293799	36.87
AT1G31550		GDSL esterase/lipase At1g31550 [Source:UniProtKB/Swiss-Prot;Acc:Q9C857]	11295446	11297379	38.88
AT1G31555			11297813	11298409	36.01
AT1G06443			11300618	11300966	35.53
AT1G06447			11300693	11301038	34.97
AT1G31580	ECS1	Protein ECS1 [Source:UniProtKB/Swiss-Prot;Acc:Q39066]	11310997	11311989	36.46
AT1G31600			11312961	11316582	37.63
AT1G31620		Uncharacterized protein F27M3_18 [Source:UniProtKB/TrEMBL;Acc:Q9C6V2]	11316941	11317894	40.15
AT1G31630	AGL86	Agamous-like MADS-box protein AGL86 [Source:UniProtKB/Swiss-Prot;Acc:Q9C6V3]	11318528	11319547	47.65
AT1G31640	AGL92	Agamous-like MADS-box protein AGL92 [Source:UniProtKB/Swiss-Prot;Acc:Q9C6V4]	11322692	11324176	44.31
AT1G31650	ROPGEF14	Rop guanine nucleotide exchange factor 14 [Source:UniProtKB/Swiss-Prot;Acc:Q56WM6]	11326251	11330791	36.05
AT1G31660		Bystin-like protein [Source:UniProtKB/TrEMBL;Acc:Q8RWS4]	11330924	11333540	37.56
AT1G31670			11337536	11341889	36.91
AT1G31690		Amine oxidase [Source:UniProtKB/TrEMBL;Acc:F4IAX0]	11343854	11347945	35.97
AT1G31710		Amine oxidase [Source:UniProtKB/TrEMBL;Acc:F4IAX1]	11349697	11355587	34.66
AT1G31720		At1g31720 [Source:UniProtKB/TrEMBL;Acc:A2RVU1]	11356076	11358095	35.3
AT1G31730		AP-4 complex subunit epsilon [Source:UniProtKB/Swiss-Prot;Acc:Q8L7A9]	11359512	11364288	40.51
AT1G31740	BGAL15	Beta-galactosidase 15 [Source:UniProtKB/Swiss-Prot;Acc:Q9C6W4]	11365285	11369839	31.17
AT1G31750		At1g31750 [Source:UniProtKB/TrEMBL;Acc:Q9C4Z8]	11370585	11372197	36.7
AT1G31760		At1g31760 [Source:UniProtKB/TrEMBL;Acc:Q9C504]	11372394	11374301	37.05
AT1G31770	ABCG14	ABC transporter G family member 14 [Source:UniProtKB/Swiss-Prot;Acc:Q9C6W5]	11374760	11377885	42.29
AT1G31772		Defensin-like (DEFL) family protein [Source:TAIR;Acc:AT1G31772]	11377879	11379734	33.03
AT1G31780		At1g31780 [Source:UniProtKB/TrEMBL;Acc:Q6NMI3]	11391199	11394748	40.87
AT1G31790	PCMP-A1	Pentatricopeptide repeat-containing protein At1g31790 [Source:UniProtKB/Swiss-Prot;Acc:Q9C6R9]	11394690	11396018	40.86

	AT1G31800	CYP97A3	Protein LUTEIN DEFICIENT 5, chloroplastic [Source:UniProtKB/Swiss-Prot;Acc:Q93VK5]	11396402	11399714	37.34
	AT1G31810	FH14	Formin-like protein 14 [Source:UniProtKB/Swiss-Prot;Acc:Q9C6S1]	11399607	11406809	39.03
	AT1G31812	ACBP6	ACBP6 [Source:UniProtKB/TrEMBL;Acc:A0A178WJ33]	11410766	11412233	35.83
MQTL-6/Chr1	AT1G33600		Leucine-rich repeat (LRR) family protein [Source:UniProtKB/TrEMBL;Acc:Q9FW48]	12180483	12184528	34.68
	AT1G06677			12184200	12184398	41.71
	AT1G33607		Putative defensin-like protein 26 [Source:UniProtKB/Swiss-Prot;Acc:Q2V4J2]	12186941	12187423	34.16
	AT1G33610		Leucine-rich repeat (LRR) family protein [Source:UniProtKB/TrEMBL;Acc:F4HR91]	12188678	12190461	43.05
	AT1G33612		Leucine-rich repeat (LRR) family protein [Source:UniProtKB/TrEMBL;Acc:F4HR92]	12191133	12193270	40.48
	AT1G33615		other RNA [Source:TAIR;Acc:AT1G33615]	12192651	12197020	33.07
	AT1G06683			12195426	12195676	37.05
	AT1G33640		Uncharacterized protein T1E4.1 [Source:UniProtKB/TrEMBL;Acc:Q9FW50]	12195722	12196223	35.06
	AT1G06687			12199359	12199789	35.5
	AT1G06693			12200427	12200747	39.25
	AT1G33670		Leucine-rich repeat (LRR) family protein [Source:UniProtKB/TrEMBL;Acc:Q4PT10]	12201963	12203408	45.09
	AT1G33680		KH domain-containing protein [Source:UniProtKB/TrEMBL;Acc:Q0WLY0]	12203945	12208698	42.07
	AT1G33700		Non-lysosomal glucosylceramidase [Source:UniProtKB/TrEMBL;Acc:F4HR96]	12208308	12214313	38.03
	AT1G06697			12217892	12218338	38.26
	AT1G33710		RNA-directed DNA polymerase (Reverse transcriptase)-related family protein [Source:UniProtKB/TrEMBL;Acc:Q3ED23]	12219657	12220289	46.6
	AT1G33720	CYP76C6	Cytochrome P450, family 76, subfamily C, polypeptide 6 [Source:UniProtKB/TrEMBL;Acc:Q9LQ25]	12220858	12224108	40.23
	AT1G33730	CYP76C5	Cytochrome P450, family 76, subfamily C, polypeptide 5 [Source:UniProtKB/TrEMBL;Acc:F4HRA1]	12226852	12228556	40.7
	AT1G06703			12232827	12233098	41.18
	AT1G06707			12233006	12233266	37.16
	AT1G33750	TPS22	Terpenoid synthase 22 [Source:UniProtKB/Swiss-Prot;Acc:Q9LQ27]	12233632	12236576	37.05
	AT1G33760	ERF022	Ethylene-responsive transcription factor ERF022 [Source:UniProtKB/Swiss-Prot;Acc:Q9LQ28]	12237547	12238710	38.75
	AT1G33770		Protein kinase superfamily protein [Source:UniProtKB/TrEMBL;Acc:Q9LQ29]	12241614	12245292	39.9
	AT1G33780		Electron transporter, putative (DUF179) [Source:UniProtKB/TrEMBL;Acc:Q8W467]	12244560	12246231	39.17
	AT1G33790	JAL4	Jacalin-related lectin 4 [Source:UniProtKB/Swiss-Prot;Acc:Q9LQ31]	12256474	12260638	35.49
	AT1G33800	GXM3	GXMT1 [Source:UniProtKB/TrEMBL;Acc:A0A178W7W0]	12261165	12262750	38.02
	AT1G33810		Zinc finger/BTB domain protein [Source:UniProtKB/TrEMBL;Acc:Q8L9M8]	12264942	12266920	36.79
	AT1G33811		GDSL esterase/lipase At1g33811 [Source:UniProtKB/Swiss-Prot;Acc:Q8L5Z1]	12267808	12269913	36.56
	AT1G33820		unknown protein; Ha. [Source:TAIR;Acc:AT1G33820]	12278438	12279451	32.84
	AT1G33830	IAN1	Immune-associated nucleotide-binding protein 1 [Source:UniProtKB/Swiss-Prot;Acc:Q9C8U2]	12279947	12281406	33.77
	AT1G33840		Protein of unknown function (DUF567) [Source:TAIR;Acc:AT1G33840]	12283725	12285371	33.88
	AT1G33850		40S ribosomal protein S15 [Source:UniProtKB/TrEMBL;Acc:Q9LD48]	12287913	12288210	39.26
	AT1G33860		unknown protein; Ha. [Source:TAIR;Acc:AT1G33860]	12294394	12295305	45.72
	AT1G06713			12297238	12297525	38.89
	AT1G33870		AIG1-like protein; 48352-49494 [Source:UniProtKB/TrEMBL;Acc:Q9C8U4]	12301325	12303330	33.9
	AT1G33880	IAN2	Immune-associated nucleotide-binding protein 2 [Source:UniProtKB/Swiss-Prot;Acc:Q9C8U5]	12303862	12305203	34.58
	AT1G33890	IAN3	Immune-associated nucleotide-binding protein 3 [Source:UniProtKB/Swiss-Prot;Acc:Q9C8U6]	12308136	12309914	38.5
	AT1G33900	IAN4	Immune-associated nucleotide-binding protein 4 [Source:UniProtKB/Swiss-Prot;Acc:Q9C8U7]	12311518	12313518	37.08
	AT1G33910	IAN5	Immune-associated nucleotide-binding protein 5 [Source:UniProtKB/Swiss-Prot;Acc:Q9C8U8]	12314904	12316258	39.56
	AT1G33920	PP2A4	Uncharacterized protein PHLOEM PROTEIN 2-LIKE A4 [Source:UniProtKB/Swiss-Prot;Acc:Q9C8U9]	12319905	12320870	34.47
	AT1G33930	IAN6	Immune-associated nucleotide-binding protein 6 [Source:UniProtKB/Swiss-Prot;Acc:Q9C8V0]	12323474	12327311	31.58
	AT1G33940		Serine/Threonine-kinase ULK4-like protein [Source:UniProtKB/TrEMBL;Acc:Q9C8V1]	12330576	12332785	39.86
	AT1G33945			12331977	12332469	43.2
	AT1G33950	IAN7	Immune-associated nucleotide-binding protein 7 [Source:UniProtKB/Swiss-Prot;Acc:Q9C8V2]	12332834	12340409	25.37
	AT1G33960	AIG1	P-loop containing nucleoside triphosphate hydrolases superfamily protein [Source:TAIR;Acc:AT1G33960]	12346138	12348733	36.29
	AT1G33970	IAN9	Immune-associated nucleotide-binding protein 9 [Source:UniProtKB/Swiss-Prot;Acc:F4HT21]	12349304	12351318	38.76
	AT1G33980	ATUPF3	Smg-4/UPF3 family protein [Source:TAIR;Acc:AT1G33980]	12351566	12355173	38.61
	AT1G33990	MES14	Putative methylesterase 14, chloroplastic [Source:UniProtKB/Swiss-Prot;Acc:Q9FVW3]	12355557	12358031	37.82
	AT1G34000	OHP2	OHP2 [Source:UniProtKB/TrEMBL;Acc:A0A178WAB5]	12357910	12358966	37.65
	AT1G06717			12358198	12358531	35.93
	AT1G34010		unknown protein; BEST Arabidopsis thaliana protein match is: unknown protein (TAIR:AT1G22790.2); Ha. [Source:TAIR;A	12359454	12361313	36.13
	AT1G06723			12361353	12361763	37.96
	AT1G34015		S-locus glycoprotein family protein [Source:UniProtKB/TrEMBL;Acc:A0A1P8AWX0]	12361832	12363493	33.69
	AT1G34020	URGT6	UDP-rhamnose/UDP-galactose transporter 6 [Source:UniProtKB/Swiss-Prot;Acc:Q9FDZ5]	12366810	12370014	39.63
	AT1G34030	RPS18C	40S ribosomal protein S18 [Source:UniProtKB/Swiss-Prot;Acc:P34788]	12370065	12371554	38.59
	AT1G34040	TAR3	Tryptophan aminotransferase-related protein 3 [Source:UniProtKB/Swiss-Prot;Acc:Q9FE98]	12374259	12376367	37.41
	AT1G34042		unknown protein; Ha. [Source:TAIR;Acc:AT1G34042]	12379427	12379703	42.6
	AT1G06727			12381197	12381406	30
	AT1G06733			12381940	12382144	31.22
	AT1G06737			12383777	12384244	38.46
	AT1G06743			12384322	12385041	35.42
	AT1G34046		Ankyrin-repeat containing protein [Source:UniProtKB/TrEMBL;Acc:F4HT36]	12388595	12389447	36.46
	AT1G34047		Defensin-like (DEFL) family protein [Source:UniProtKB/TrEMBL;Acc:F4HT37]	12390238	12390915	39.97
	AT1G34049			12390448	12391149	37.32
	AT1G34050		Ankyrin repeat family protein [Source:UniProtKB/TrEMBL;Acc:Q9FX13]	12393426	12396194	38.82
	AT1G34060	TAR4	Tryptophan aminotransferase-related protein 4 [Source:UniProtKB/Swiss-Prot;Acc:Q93Z38]	12396397	12398418	38.82
	AT1G34065	SAMC2	Probable S-adenosylmethionine carrier 2, chloroplastic [Source:UniProtKB/Swiss-Prot;Acc:F4HT41]	12398428	12401036	36.3
	AT1G34070		Copia-like polyprotein/retrotransposon [Source:UniProtKB/TrEMBL;Acc:Q9FX16]	12402283	12403209	46.28
	AT1G34095		unknown protein; BEST Arabidopsis thaliana protein match is: unknown protein (TAIR:AT2G45403.1); Ha. [Source:TAIR;A	12411670	12412514	40.12
	AT1G34110		Probable LRR receptor-like serine/threonine-protein kinase At1g34110 [Source:UniProtKB/Swiss-Prot;Acc:C0LGF5]	12417012	12421792	40.79
	AT1G34120	IP5P1	Type I inositol polyphosphate 5-phosphatase 1 [Source:UniProtKB/Swiss-Prot;Acc:Q84MA2]	12426700	12429714	38.77
	AT1G34130	STT3B	Glycosyltransferase (Fragment) [Source:UniProtKB/TrEMBL;Acc:W8PVA0]	12429846	12433236	40.55
	AT1G34140	PAB1	poly(A) binding protein 1 [Source:TAIR;Acc:AT1G34140]	12433356	12434735	41.01
	AT1G34150		tRNA pseudouridine synthase [Source:UniProtKB/TrEMBL;Acc:Q9C5K6]	12435882	12439499	38.11
	AT1G34160	PCMP-H68	Pentatricopeptide repeat-containing protein At1g34160 [Source:UniProtKB/Swiss-Prot;Acc:Q9FX24]	12441244	12443368	41.6
	AT1G34170	ARF13	Auxin response factor [Source:UniProtKB/TrEMBL;Acc:F4HT52]	12443547	12446764	32.19
	AT1G34180	anac016	NAC domain containing protein 16 [Source:TAIR;Acc:AT1G34180]	12448543	12451287	38.65
	AT1G34190	NAC017	NAC017 [Source:UniProtKB/TrEMBL;Acc:A0A178WNW4]	12451405	12454542	41.24
	AT1G34200		At1g34200/F23M19.12 [Source:UniProtKB/TrEMBL;Acc:Q9ASQ9]	12455653	12457547	39.68
	AT1G34210	SERK2	Somatic embryogenesis receptor kinase 2 [Source:UniProtKB/Swiss-Prot;Acc:Q9XIC7]	12458564	12462905	38.9
	AT1G34220		Regulator of Vps4 activity in the MVB pathway protein [Source:UniProtKB/TrEMBL;Acc:F4HUX0]	12462849	12466089	40.33
	AT1G34245	EPF2	EPF2 [Source:UniProtKB/TrEMBL;Acc:A0A178W2V8]	12472868	12473974	35.14
	AT1G34260	FAB1D	Putative 1-phosphatidylinositol-3-phosphate 5-kinase FAB1D [Source:UniProtKB/Swiss-Prot;Acc:Q9XID0]	12485318	12492207	38.84
	AT1G34270		Exostosin family protein [Source:UniProtKB/TrEMBL;Acc:Q9XID1]	12492321	12494584	40.9
	AT1G34290	AtRLP5	Receptor-like protein 5 [Source:UniProtKB/Swiss-Prot;Acc:Q9XID2]	12498000	12498800	41.7
	AT1G34300		G-type lectin S-receptor-like serine/threonine-protein kinase At1g34300 [Source:UniProtKB/Swiss-Prot;Acc:Q9XID3]	12503360	12506172	43.37
	AT1G34310	ARF12	Auxin response factor 12 [Source:UniProtKB/Swiss-Prot;Acc:Q9XID4]	12508548	12511520	33.4
	AT1G06747			12512325	12512684	36.11
	AT1G34315		unknown protein; FUNCTIONS IN: molecular_function unknown; INVOLVED IN: biological_process unknown; LOCATEI	12514106	12516521	40.73
	AT1G34317			12516112	12516555	43.69
	AT1G34320		At1g34320 [Source:UniProtKB/TrEMBL;Acc:Q6NQ48]	12520059	12524377	38.92
	AT1G34340		Alpha/beta-Hydrolases superfamily protein [Source:UniProtKB/TrEMBL;Acc:Q9XID7]	12530815	12534306	37.14
	AT1G34350		unknown protein; Ha. [Source:TAIR;Acc:AT1G34350]	12534426	12536162	36.56
	AT1G34355	PS1	FHA domain-containing protein PS1 [Source:UniProtKB/Swiss-Prot;Acc:B7SY83]	12536310	12541938	41.45
	AT1G34360	IF3-1	Translation initiation factor IF3-1, mitochondrial [Source:UniProtKB/Swiss-Prot;Acc:Q6NLP2]	12542796	12546244	39.92
	AT1G34370	STOP1	Protein SENSITIVE TO PROTON RHIZOTOXICITY 1 [Source:UniProtKB/Swiss-Prot;Acc:Q9C8N5]	12550339	12552629	41.95

	AT1G06753			12550564	12550980	29.02
	AT1G34380		5'-3' exonuclease family protein [Source:UniProtKB/TrEMBL;Acc:Q66GR7]	12552655	12554363	38.85
	AT1G34390	ARF22	Auxin response factor 22 [Source:UniProtKB/Swiss-Prot;Acc:Q9C8N7]	12556005	12559082	33.85
	AT1G34392		Unknown gene [Source:TAIR;Acc:AT1G34392]	12560460	12561315	30.61
	AT1G34400		unknown protein; Ha. [Source:TAIR;Acc:AT1G34400]	12565992	12566615	52.08
	AT1G34403			12575892	12576173	41.13
	AT1G34410	ARF21	Putative auxin response factor 21 [Source:UniProtKB/Swiss-Prot;Acc:Q9C8N9]	12577722	12580824	33.55
	AT1G34418		other RNA [Source:TAIR;Acc:AT1G34418]	12582122	12582721	35.17
	AT1G34420		F12K21.25 [Source:UniProtKB/TrEMBL;Acc:Q9LNK3]	12584345	12587778	42.49
	AT1G34430	EMB3003	Dihydrolipoyllysine-residue acetyltransferase component 5 of pyruvate dehydrogenase complex, chloroplastic [Source:UniProt	12587726	12590223	41.87
	AT1G34440		unknown protein; BEST Arabidopsis thaliana protein match is: unknown protein (TAIR:AT4G05095.1); Ha. [Source:TAIR;A	12591348	12593579	34.05
	AT1G34460	CYCB1;5	CYCLIN B1;5 [Source:TAIR;Acc:AT1G34460]	12595110	12602379	34.66
	AT1G06757			12601014	12601360	30.84
	AT1G06773			12602898	12603141	40.57
	AT1G34470		Probable magnesium transporter NIPA3 [Source:UniProtKB/Swiss-Prot;Acc:Q9LNK7]	12604161	12607033	37.28
	AT1G34480		Cysteine/Histidine-rich C1 domain family protein [Source:UniProtKB/TrEMBL;Acc:Q9LNK8]	12607222	12609084	36.39
	AT1G34490		Putative long-chain-alcohol O-fatty-acyltransferase 10 [Source:UniProtKB/Swiss-Prot;Acc:Q3ED15]	12609440	12610495	42.14
	AT1G34500		Probable long-chain-alcohol O-fatty-acyltransferase 9 [Source:UniProtKB/Swiss-Prot;Acc:Q4PT07]	12611638	12612663	45.32
	AT1G34510	PER8	Peroxidase [Source:UniProtKB/TrEMBL;Acc:Q0WRX6]	12615690	12617032	39.91
	AT1G34520		Probable long-chain-alcohol O-fatty-acyltransferase 8 [Source:UniProtKB/Swiss-Prot;Acc:Q9LNL1]	12623434	12624508	42.42
	AT1G34540	CYP94D1	CYP94D1 [Source:UniProtKB/TrEMBL;Acc:A0A178WEM5]	12637054	12638692	44.05
	AT1G06777			12643648	12643882	37.02
	AT1G06783			12643681	12643997	35.65
	AT1G34550	EMB2756	Protein of unknown function (DUF616) [Source:TAIR;Acc:AT1G34550]	12646674	12652646	38.72
	AT1G34560		Protein of unknown function (DUF1184) [Source:TAIR;Acc:AT1G34560]	12653961	12654779	39.56
	AT1G34570		Essential protein Yae1, N-terminal [Source:UniProtKB/TrEMBL;Acc:F4HV08]	12654927	12656605	37.46
	AT1G34575		Berberine bridge enzyme-like 14 [Source:UniProtKB/Swiss-Prot;Acc:F4HV09]	12657027	12658756	43.58
	AT1G34580	STP5	Sugar transport protein 5 [Source:UniProtKB/Swiss-Prot;Acc:Q93Y91]	12660497	12663870	37.43
	AT1G34630		At1g34630/F12K21.3 [Source:UniProtKB/TrEMBL;Acc:Q8VZG0]	12685176	12687641	40.15
	AT1G06787			12687105	12687401	43.43
	AT1G34640		At1g34640 [Source:UniProtKB/TrEMBL;Acc:Q9LNM6]	12687552	12688543	34.27
	AT1G34650	HDG10	Homeobox-leucine zipper protein HDG10 [Source:UniProtKB/Swiss-Prot;Acc:Q9S9Z0]	12692995	12697834	33.16
	AT1G34670	AtMYB93	Transcription factor MYB93 [Source:UniProtKB/Swiss-Prot;Acc:Q9S9Z2]	12709090	12710665	38.01
MQTL-7/Chr1	AT1G59790		Putative cullin-like protein 2 [Source:UniProtKB/Swiss-Prot;Acc:Q9XIE9]	22001504	22003575	34.12
	AT1G59800		Cullin-like protein 3 [Source:UniProtKB/Swiss-Prot;Acc:Q9XIE8]	22004964	22006172	36.06
	AT1G08263			22007825	22008111	37.63
	AT1G59810	AGL50	AGAMOUS-like 50 [Source:UniProtKB/TrEMBL;Acc:Q9XIE7]	22008507	22009455	43.62
	AT1G59820	ALA3	Phospholipid-transporting ATPase [Source:UniProtKB/TrEMBL;Acc:A0A178WFR7]	22011305	22020386	37.37
	AT1G59830	PP2A1	Serine/threonine-protein phosphatase PP2A-1 catalytic subunit [Source:UniProtKB/Swiss-Prot;Acc:Q07099]	22020377	22022503	39.16
	AT1G59833		Putative defensin-like protein 63 [Source:UniProtKB/Swiss-Prot;Acc:Q2V4G1]	22023337	22023714	33.07
	AT1G59835	CEP2	Precursor of CEP2 [Source:UniProtKB/Swiss-Prot;Acc:Q3ECM0]	22024945	22025421	35.85
	AT1G59840	CCB4	Protein COFACTOR ASSEMBLY OF COMPLEX C SUBUNIT B CCB4, chloroplastic [Source:UniProtKB/Swiss-Prot;Acc:	22026462	22029379	38.07
	AT1G08267			22026927	22027168	35.95
	AT1G59850	TOR1L5	TORTIFOLIA1-like protein 5 [Source:UniProtKB/Swiss-Prot;Acc:Q9XIE4]	22028509	22030261	45.35
	AT1G08273			22029795	22030289	49.29
	AT1G59860	HSP17.6A	17.6 kDa class I heat shock protein 1 [Source:UniProtKB/Swiss-Prot;Acc:Q9XIE3]	22031343	22032250	39.98
	AT1G59865		unknown protein; Ha. [Source:TAIR;Acc:AT1G59865]	22032313	22033297	36.95
	AT1G59870	ABCG36	ABC transporter G family member 36 [Source:UniProtKB/Swiss-Prot;Acc:Q9XIE2]	22034423	22040126	41.65
	AT1G59880		pre-tRNA [Source:TAIR;Acc:AT1G59880]	22040612	22040683	56.94
	AT1G59885		unknown protein; Ha. [Source:TAIR;Acc:AT1G59885]	22042308	22042815	41.14
	AT1G59890	SNL5	SIN3-like 5 [Source:UniProtKB/TrEMBL;Acc:F4IEM8]	22043643	22051114	37.29
	AT1G59900	E1 ALPHA	Pyruvate dehydrogenase E1 component subunit alpha-1, mitochondrial [Source:UniProtKB/Swiss-Prot;Acc:P52901]	22051133	22053885	40.17
	AT1G59910	FH7	Formin-like protein 7 [Source:UniProtKB/Swiss-Prot;Acc:Q9XIE0]	22053863	22057268	44.01
	AT1G59920		F23H11.23 protein [Source:UniProtKB/TrEMBL;Acc:Q9XID9]	22059424	22059842	42
	AT1G59930		F23H11.24 protein [Source:UniProtKB/TrEMBL;Acc:Q9SXC1]	22060924	22061516	36.59
	AT1G59940	ARR3	response regulator 3 [Source:TAIR;Acc:AT1G59940]	22065617	22067233	38.34
	AT1G59950		Aldo/keto reductase [Source:UniProtKB/TrEMBL;Acc:Q1PFI5]	22068033	22070588	37.4
	AT1G59960		NAD(P)-linked oxidoreductase superfamily protein [Source:UniProtKB/TrEMBL;Acc:Q9SXC0]	22071217	22073267	38.23
	AT1G59970	5MMP	Metalloendoproteinase 5-MMP [Source:UniProtKB/Swiss-Prot;Acc:Q9ZUJ5]	22073352	22074931	41.39
	AT1G08277			22075614	22075885	39.71
	AT1G08283			22076533	22077068	33.21
	AT1G59980	ATJ39	Chaperone protein dnaJ 39 [Source:UniProtKB/Swiss-Prot;Acc:Q6XL73]	22080689	22086845	36.02
	AT1G08287			22084264	22084683	34.05
	AT1G08293			22087975	22088246	35.29
	AT1G59990	RH22	DEAD-box ATP-dependent RNA helicase 22 [Source:UniProtKB/Swiss-Prot;Acc:Q944S1]	22090226	22093385	39.34
	AT1G60000		AT1G60000 protein [Source:UniProtKB/TrEMBL;Acc:Q9ZUJ3]	22093353	22094802	40.34
	AT1G60010		D-ribose-binding periplasmic protein [Source:UniProtKB/TrEMBL;Acc:Q9ZUJ2]	22095285	22096620	35.33
	AT1G60025	MIR426	MIR426; miRNA [Source:TAIR;Acc:AT1G60025]	22107346	22107456	36.04
	AT1G60030	NAT7	Nucleobase-ascorbate transporter 7 [Source:UniProtKB/Swiss-Prot;Acc:Q0WPE9]	22113671	22117018	38.95
	AT1G60040	AGL49	Agamous-like MADS-box protein AGL49 [Source:UniProtKB/Swiss-Prot;Acc:Q9ZUI9]	22118974	22119929	42.99
	AT1G60050		WAT1-related protein [Source:UniProtKB/TrEMBL;Acc:A0A178WGH0]	22121375	22123881	33.07
	AT1G08297			22128871	22129201	35.05
	AT1G60060		Serine/threonine-protein kinase WNK (With No Lysine)-like protein [Source:UniProtKB/TrEMBL;Acc:Q9ZUI7]	22139170	22141815	34.09
	AT1G60070		AP-1 complex subunit gamma [Source:UniProtKB/TrEMBL;Acc:F4IEP9]	22142604	22149527	37.07
	AT1G60072	MIR859A	MIR859a; miRNA [Source:TAIR;Acc:AT1G60072]	22149718	22149843	31.75
	AT1G60073	MIR774A	MIR774a; miRNA [Source:TAIR;Acc:AT1G60073]	22149936	22150033	29.59
	AT1G08303			22150128	22150266	28.06
	AT1G60080		3'-5'-exoribonuclease family protein [Source:UniProtKB/TrEMBL;Acc:Q9ZUI4]	22152230	22154643	37.99
	AT1G60090	BGLU4	Beta-glucosidase 4 [Source:UniProtKB/Swiss-Prot;Acc:Q9ZUI3]	22155559	22158272	38.61
	AT1G60095		Mannose-binding lectin superfamily protein [Source:TAIR;Acc:AT1G60095]	22159694	22162426	36.99
	AT1G60110		Mannose-binding lectin superfamily protein [Source:TAIR;Acc:AT1G60110]	22168424	22170803	39.08
	AT1G08307			22173808	22174054	36.03
	AT1G60130	JAL18	Jacalin-related lectin 18 [Source:UniProtKB/Swiss-Prot;Acc:O80737]	22173937	22176343	38.76
	AT1G60140	TPS10	Probable alpha,alpha-trehalose-phosphate synthase [UDP-forming] 10 [Source:UniProtKB/Swiss-Prot;Acc:O80738]	22176934	22181740	37.7
	AT1G60160	POT12	Putative potassium transporter 12 [Source:UniProtKB/Swiss-Prot;Acc:O80739]	22188030	22191543	39.93
	AT1G60170	PRP31	Emb1220 [Source:UniProtKB/TrEMBL;Acc:A0A178WCJ2]	22192758	22195400	39.65
	AT1G60190	PUB19	U-box domain-containing protein 19 [Source:UniProtKB/Swiss-Prot;Acc:O80742]	22198266	22200687	43.02
	AT1G60200		Splicing factor PWI domain-containing protein / RNA recognition motif (RRM)-containing protein [Source:UniProtKB/TrEM	22200694	22205400	41.05
	AT1G60220	ULP1D	Ubiquitin-like-specific protease 1D [Source:UniProtKB/Swiss-Prot;Acc:Q2PS26]	22208089	22212171	37.72
	AT1G60230		Radical SAM superfamily protein [Source:UniProtKB/TrEMBL;Acc:Q93XX3]	22212084	22214431	40.76
	AT1G60240		At1g60240 [Source:UniProtKB/TrEMBL;Acc:Q1ECJ5]	22215168	22216329	36.4
	AT1G60250		B-box zinc finger family protein [Source:UniProtKB/TrEMBL;Acc:O80748]	22217077	22217832	43.92
	AT1G60260	BGLU5	beta glucosidase 5 [Source:TAIR;Acc:AT1G60260]	22218787	22221433	36.76
	AT1G60270	BGLU6	Putative beta-glucosidase 6 [Source:UniProtKB/Swiss-Prot;Acc:Q682B4]	22221575	22224358	37.18
	AT1G60280	ANAC023	At1g60280 [Source:UniProtKB/TrEMBL;Acc:O80751]	22226885	22227928	41.19
	AT1G60300		Apical meristem formation protein-related [Source:UniProtKB/TrEMBL;Acc:O80752]	22229780	22230748	40.66
	AT1G60320		Toll-Interleukin-Resistance (TIR) domain family protein [Source:UniProtKB/TrEMBL;Acc:F4IER8]	22235091	22235606	42.83
	AT1G60340		NAC (No Apical Meristem) domain transcriptional regulator superfamily protein [Source:UniProtKB/TrEMBL;Acc:O80755]	22238734	22240399	39.8

AT1G60350	anac024	NAC domain containing protein 24 [Source:UniProtKB/TrEMBL;Acc:O80756]	22241273	22242235	41.74
AT1G60360		RING/U-box superfamily protein [Source:UniProtKB/TrEMBL;Acc:O80757]	22242531	22243760	43.9
AT1G08313			22244179	22244406	33.77
AT1G60370		Putative F-box protein At1g60370 [Source:UniProtKB/Swiss-Prot;Acc:O80758]	22244963	22245814	41.67
AT1G60380		Apical meristem formation protein-related [Source:UniProtKB/TrEMBL;Acc:O80759]	22246455	22247411	41.8
AT1G60390	PGL2	Polygalacturonase 1 beta-like protein 2 [Source:UniProtKB/Swiss-Prot;Acc:O80760]	22247472	22249721	43.33
AT1G60400		F-box protein At1g60400 [Source:UniProtKB/Swiss-Prot;Acc:Q1PFI4]	22254405	22255798	37.73
AT1G60410		FBD-associated F-box protein At1g60410 [Source:UniProtKB/Swiss-Prot;Acc:O80762]	22258939	22260787	37.48
AT1G60420		Probable nucleoredoxin 1 [Source:UniProtKB/Swiss-Prot;Acc:O80763]	22261754	22264408	42.11
AT1G08317			22263727	22264033	46.91
AT1G60430	ARPC3	Actin-related protein 2/3 complex subunit 3 [Source:UniProtKB/Swiss-Prot;Acc:Q1ECJ7]	22264385	22266079	37.76
AT1G60440	PANK1	Pantothenate kinase 1 [Source:UniProtKB/Swiss-Prot;Acc:O80765]	22266416	22269258	36.12
AT1G60450	GOLS7	Galactinol synthase 7 [Source:UniProtKB/Swiss-Prot;Acc:Q4PSY4]	22271131	22273367	34.64
AT1G60460		unknown protein; FUNCTIONS IN: molecular_function unknown; INVOLVED IN: biological_process unknown; LOCATEI	22275484	22279007	37.43
AT1G08323			22278282	22278557	43.84
AT1G60470	GOLS4	Galactinol synthase 4 [Source:UniProtKB/Swiss-Prot;Acc:O22693]	22278975	22280593	40.09
AT1G60490	ATVPS34	Phosphatidylinositol 3-kinase VPS34 [Source:UniProtKB/Swiss-Prot;Acc:P42339]	22285572	22290389	39.37
AT1G60500	DRP4C	Dynamin-related protein 4C [Source:UniProtKB/Swiss-Prot;Acc:Q9ZP55]	22291582	22293964	43.6
AT1G60505		other RNA [Source:TAIR;Acc:AT1G60505]	22294362	22297374	43.05
AT1G60525		other RNA [Source:TAIR;Acc:AT1G60525]	22299435	22301441	40.51
AT1G60530	DRP4A	Putative dynamin-related protein 4A [Source:UniProtKB/Swiss-Prot;Acc:Q9ZP56]	22299797	22301167	41.79
AT1G60545		other RNA [Source:TAIR;Acc:AT1G60545]	22302306	22305242	41.91
AT1G60550	MENB	1,4-dihydroxy-2-naphthoyl-CoA synthase, peroxisomal [Source:UniProtKB/Swiss-Prot;Acc:Q8GYN9]	22305780	22308289	39.2
AT1G60560		At1g60560/F8A5_10 [Source:UniProtKB/TrEMBL;Acc:Q8RY56]	22308478	22311377	40.31
AT1G60570		Putative F-box/kelch-repeat protein At1g60570 [Source:UniProtKB/Swiss-Prot;Acc:O22698]	22311836	22312981	43.72
AT1G08327			22313324	22313411	43.18
AT1G60580		pre-tRNA [Source:TAIR;Acc:AT1G60580]	22313414	22313484	59.15
AT1G60590		Pectin lyase-like superfamily protein [Source:UniProtKB/TrEMBL;Acc:O22699]	22314089	22317070	38.93
AT1G60600	ABC4	2-carboxy-1,4-naphthoquinone phytyltransferase, chloroplastic [Source:UniProtKB/Swiss-Prot;Acc:Q0WUA3]	22324437	22327359	38.28
AT1G60610		At1g60610 [Source:UniProtKB/TrEMBL;Acc:Q4TU35]	22327792	22330318	37.12
AT1G60620	ATRPAC43	RNA polymerase I subunit 43 [Source:UniProtKB/TrEMBL;Acc:Q39216]	22330894	22333568	38.77
AT1G60625	RALFL6	Protein RALF-like 6 [Source:UniProtKB/Swiss-Prot;Acc:A8MQM2]	22333916	22334161	36.59
AT1G60630		Inactive leucine-rich repeat receptor-like serine/threonine-protein kinase At1g60630 [Source:UniProtKB/Swiss-Prot;Acc:Q84	22334540	22336994	43.22
AT1G60640		unknown protein; Ha. [Source:TAIR;Acc:AT1G60640]	22337253	22339736	37.4
AT1G60650	RZ1B	Glycine-rich RNA-binding protein RZ1B [Source:UniProtKB/Swiss-Prot;Acc:O22703]	22339809	22342337	40.81
AT1G60660	CB5LP	Cytochrome B5-like protein [Source:UniProtKB/Swiss-Prot;Acc:O22704]	22342290	22343269	37.24
AT1G60670		Uncharacterized protein At1g60670 [Source:UniProtKB/TrEMBL;Acc:Q94AP2]	22343585	22347624	36.93
AT1G08333			22346819	22347046	35.09
AT1G60680	AGD2	Probable aldo-keto reductase 2 [Source:UniProtKB/Swiss-Prot;Acc:Q84M96]	22347622	22349317	41.63
AT1G60690		Probable aldo-keto reductase 3 [Source:UniProtKB/Swiss-Prot;Acc:O22707]	22349759	22351780	40.95
AT1G60700		SMAD/FHA domain-containing protein [Source:UniProtKB/TrEMBL;Acc:F4HPY1]	22351804	22354517	38.32
AT1G60710	ATB2	Probable aldo-keto reductase 4 [Source:UniProtKB/Swiss-Prot;Acc:Q93ZN2]	22354698	22356875	42.06
AT1G60720		RNA-directed DNA polymerase (Reverse transcriptase)-related family protein [Source:UniProtKB/TrEMBL;Acc:F4HPY3]	22356860	22357941	46.77
AT1G60730		NAD(P)-linked oxidoreductase superfamily protein [Source:TAIR;Acc:AT1G60730]	22357976	22360277	40.36
AT1G60740	PRXIID	At1g60740 [Source:UniProtKB/TrEMBL;Acc:B4G289]	22360977	22362003	38.27
AT1G60750		Probable aldo-keto reductase 6 [Source:UniProtKB/Swiss-Prot;Acc:F4HPY8]	22362293	22363854	42.06
AT1G60760		F8A5.27 protein [Source:UniProtKB/TrEMBL;Acc:O22713]	22365799	22366598	42.88
AT1G60770		Pentatricopeptide repeat-containing protein At1g60770 [Source:UniProtKB/Swiss-Prot;Acc:O22714]	22366701	22368714	41.11
AT1G60780	AP1M2	AP-1 complex subunit mu-2 [Source:UniProtKB/Swiss-Prot;Acc:O22715]	22368953	22372223	37.69
AT1G08337			22372462	22372578	32.48
AT1G08343			22372670	22372790	31.4
AT1G60783	SMR15	Cyclin-dependent protein kinase inhibitor SMR15 [Source:UniProtKB/Swiss-Prot;Acc:Q1G3Y4]	22374909	22375846	34.75
AT1G60787		Cysteine/histidine-rich C1 domain protein [Source:UniProtKB/TrEMBL;Acc:A0A1P8ATJ8]	22378102	22379981	37.07
AT1G60790	TBL2	Protein trichome birefringence-like 2 [Source:UniProtKB/Swiss-Prot;Acc:Q8VYR3]	22380002	22382422	40.07
AT1G60800	NIK3	Protein NSP-INTERACTING KINASE 3 [Source:UniProtKB/Swiss-Prot;Acc:Q93ZS4]	22383393	22387766	37.36
AT1G60810	ACLA-2	ATP-citrate synthase alpha chain protein 2 [Source:UniProtKB/Swiss-Prot;Acc:O22718]	22388527	22391237	38.8
AT1G60815	RALFL7	Protein RALF-like 7 [Source:UniProtKB/Swiss-Prot;Acc:A8MRD4]	22392729	22392974	36.59
AT1G08347			22394549	22394634	45.35
AT1G60820		pre-tRNA [Source:TAIR;Acc:AT1G60820]	22394637	22394707	59.15
AT1G60830		RNA-binding (RRM/RBD/RNP motifs) family protein [Source:UniProtKB/TrEMBL;Acc:Q9C6C1]	22395003	22396133	37.84
AT1G60840		pre-tRNA [Source:TAIR;Acc:AT1G60840]	22397592	22397662	57.75
AT1G60850	ATRPAC42	DNA-directed RNA polymerase family protein [Source:UniProtKB/TrEMBL;Acc:Q9C6C2]	22397913	22400413	37.94
AT1G60860	AGD2	ADP-ribosylation factor GTPase-activating protein AGD2 [Source:UniProtKB/Swiss-Prot;Acc:Q9C6C3]	22400857	22407824	35.91
AT1G60870	MEE9	Maternal effect embryo arrest 9 [Source:UniProtKB/TrEMBL;Acc:Q9C529]	22409298	22410854	37.32
AT1G60880	AGL56	AGAMOUS-like-56 [Source:UniProtKB/TrEMBL;Acc:Q9C963]	22411575	22412180	43.73
AT1G60890		Phosphatidylinositol 4-phosphate 5-kinase [Source:UniProtKB/TrEMBL;Acc:F4HRM3]	22412188	22417312	38.3
AT1G60900	U2AF65B	Splicing factor U2af large subunit B [Source:UniProtKB/Swiss-Prot;Acc:Q8L716]	22423894	22428053	39.45
AT1G60910		pre-tRNA [Source:TAIR;Acc:AT1G60910]	22428167	22428237	57.75
AT1G08353			22428241	22428327	41.38
AT1G60913		Protein RALF-like 35 [Source:UniProtKB/Swiss-Prot;Acc:A8MRK3]	22428605	22428914	34.52
AT1G60920	AGL55	AGAMOUS-like 55 [Source:UniProtKB/TrEMBL;Acc:Q9C960]	22429692	22430361	42.69
AT1G60930	RECQL4B	RECQ helicase L4B [Source:TAIR;Acc:AT1G60930]	22430847	22438877	37.58
AT1G60940	SRK2B	Serine/threonine-protein kinase SRK2B [Source:UniProtKB/Swiss-Prot;Acc:Q9C958]	22439063	22442221	38.05
AT1G60950	FD2	Ferredoxin [Source:UniProtKB/TrEMBL;Acc:A0A178W2W5]	22444307	22445291	41.62
AT1G60960	IRT3	IRT3 [Source:UniProtKB/TrEMBL;Acc:A0A178WHL0]	22445299	22447299	41.38
AT1G60970		Coatomer subunit zeta-1 [Source:UniProtKB/Swiss-Prot;Acc:Q940S5]	22447811	22449536	34.41
AT1G60980	GA20OX4	Gibberellin 20 oxidase 4 [Source:UniProtKB/Swiss-Prot;Acc:Q9C955]	22452573	22454140	39.03
AT1G60983	SCRL8	Defensin-like protein 250 [Source:UniProtKB/Swiss-Prot;Acc:P82627]	22455320	22455589	40
AT1G60985	SCRL6	Defensin-like protein 247 [Source:UniProtKB/Swiss-Prot;Acc:P82625]	22456749	22457326	34.6
AT1G60986	SCRL4	Defensin-like protein 245 [Source:UniProtKB/Swiss-Prot;Acc:P82623]	22459000	22459650	34.87
AT1G60987	SCRL5	Defensin-like protein 246 [Source:UniProtKB/Swiss-Prot;Acc:P82624]	22460365	22461164	36.12
AT1G60990		Putative transferase At1g60990, chloroplastic [Source:UniProtKB/Swiss-Prot;Acc:Q681Y3]	22462026	22465495	37.9
AT1G60989	SCRL7	Defensin-like protein 249 [Source:UniProtKB/Swiss-Prot;Acc:P82626]	22462160	22462721	37.54
AT1G60995		Membralin-like protein At1g60995 [Source:UniProtKB/Swiss-Prot;Acc:Q8GWG6]	22465571	22470839	37.64
AT1G61000	NUF2	Kinetochore protein NUF2 homolog [Source:UniProtKB/Swiss-Prot;Acc:Q8RXJ0]	22470828	22474187	36.73
AT1G61010	CPSF73-I	Cleavage and polyadenylation specificity factor subunit 3-I [Source:UniProtKB/Swiss-Prot;Acc:Q9C952]	22474267	22477765	39.53
AT1G61020		pre-tRNA [Source:TAIR;Acc:AT1G61020]	22477773	22477846	59.46
AT1G61030		WAPL (Wings apart-like protein regulation of heterochromatin) protein [Source:UniProtKB/TrEMBL;Acc:Q9C951]	22477896	22482451	38.96
AT1G61040	VIP5	VIP5 [Source:UniProtKB/TrEMBL;Acc:A0A178WDP6]	22483104	22485969	43.68
AT1G61050		alpha 1,4-glycosyltransferase family protein [Source:TAIR;Acc:AT1G61050]	22486657	22488361	38.42
AT1G61060		Putative F-box protein At1g61060 [Source:UniProtKB/Swiss-Prot;Acc:Q9C948]	22488548	22489711	39
AT1G61065		1,3-beta-glucan synthase component (DUF1218) [Source:UniProtKB/TrEMBL;Acc:Q8W576]	22490221	22491539	37.6
AT1G61070	PDF2.4	Defensin-like protein 5 [Source:UniProtKB/Swiss-Prot;Acc:Q9C947]	22491692	22492542	33.49
AT1G61080		Hydroxyproline-rich glycoprotein family protein [Source:TAIR;Acc:AT1G61080]	22492878	22497267	39.68
AT1G61090		unknown protein; BEST Arabidopsis thaliana protein match is: unknown protein (TAIR:AT1G61095.1); Ha. [Source:TAIR;A	22499317	22500331	35.86
AT1G61093		unknown protein; FUNCTIONS IN: molecular_function unknown; INVOLVED IN: biological_process unknown; LOCATEI	22501885	22502941	33.77
AT1G61095		At1g61095 [Source:UniProtKB/TrEMBL;Acc:Q147K6]	22504514	22505511	35.07
AT1G61097		At1g61097 [Source:UniProtKB/TrEMBL;Acc:Q3ECL3]	22506919	22507947	34.89

AT1G61100		CSL1 [Source:UniProtKB/TrEMBL;Acc:F4HRR0]	22508524	22512333	39.21
AT1G61105		Toll-Interleukin-Resistance (TIR) domain family protein [Source:UniProtKB/TrEMBL;Acc:F4HRR1]	22513585	22514492	35.46
AT1G61110	NAC025	NAC transcription factor 25 [Source:UniProtKB/Swiss-Prot;Acc:Q8GY42]	22516602	22518292	37.43
AT1G08357			22522489	22522725	42.19
AT1G61120	GES	(E,E)-geranyllinalool synthase [Source:UniProtKB/Swiss-Prot;Acc:Q93YV0]	22523635	22528836	33.97
AT1G61130	SCPL32	Serine carboxypeptidase-like 32 [Source:UniProtKB/Swiss-Prot;Acc:Q4PSY2]	22528853	22531827	33.34
AT1G61140	EDA16	SNF2 domain-containing protein / helicase domain-containing protein / zinc finger protein-like protein [Source:UniProtKB/Tr	22534663	22540933	39.12
AT1G61150	GID8	Protein GID8 homolog [Source:UniProtKB/Swiss-Prot;Acc:Q84WK5]	22542402	22545661	39.11
AT1G61160		CONTAINS InterPro DOMAIN/s: Retrotransposon gag protein (InterPro:IPR005162); Ha. [Source:TAIR;Acc:AT1G61160]	22544868	22546230	34.92
AT1G61165		unknown protein; Ha. [Source:TAIR;Acc:AT1G61165]	22548023	22548243	38.91
AT1G61170		At1g61170 [Source:UniProtKB/TrEMBL;Acc:O22729]	22549523	22550711	41.97
AT1G61180		LRR and NB-ARC domains-containing disease resistance protein [Source:UniProtKB/TrEMBL;Acc:Q2V4G0]	22551271	22554684	39.1
AT1G61190		Probable disease resistance protein At1g61190 [Source:UniProtKB/Swiss-Prot;Acc:O22727]	22555689	22560757	37.84
AT1G61200		F11P17.8 protein [Source:UniProtKB/TrEMBL;Acc:O22726]	22562811	22563577	34.55
AT1G61210		Katanin p80 WD40 repeat-containing subunit B1 homolog [Source:UniProtKB/TrEMBL;Acc:F4HTH8]	22564359	22572029	38.57
AT1G61215	BRD4	At1g61215 [Source:UniProtKB/TrEMBL;Acc:Q6AWX2]	22572609	22575583	38.18
AT1G61224	MIR842A	MIR842a; miRNA [Source:TAIR;Acc:AT1G61224]	22577067	22577265	41.21
AT1G61226	MIR846A	MIR846a; miRNA [Source:TAIR;Acc:AT1G61226]	22577375	22577733	37.88
AT1G61230		Mannose-binding lectin superfamily protein [Source:UniProtKB/TrEMBL;Acc:F4HTI1]	22578951	22580781	38.72
AT1G08363			22581099	22581302	33.33
AT1G61240		At1g61240 [Source:UniProtKB/TrEMBL;Acc:Q8GYU5]	22581336	22585225	35.14
AT1G61250	SCAMP3	Secretory carrier-associated membrane protein 3 [Source:UniProtKB/Swiss-Prot;Acc:Q9M5P2]	22585752	22588969	34.52
AT1G61255		At1g61255 [Source:UniProtKB/TrEMBL;Acc:O22721]	22589098	22589789	41.33
AT1G61260		Protein of unknown function (DUF761) [Source:TAIR;Acc:AT1G61260]	22593465	22595338	39.91
AT1G61270		Lysine histidine transporter-like 3 [Source:UniProtKB/Swiss-Prot;Acc:O22719]	22599541	22602252	37.43
AT1G61275	U12	U12; snRNA [Source:TAIR;Acc:AT1G61275]	22603121	22603295	50.29
AT1G61280		Phosphatidylinositol N-acetylglucosaminyltransferase subunit P [Source:UniProtKB/Swiss-Prot;Acc:O64792]	22603586	22605008	36.33
AT1G61290	SYP124	Syntaxin-124 [Source:UniProtKB/Swiss-Prot;Acc:O64791]	22604777	22606385	38.1
AT1G08367			22606786	22606960	20
AT1G61300		Probable disease resistance protein At1g61300 [Source:UniProtKB/Swiss-Prot;Acc:O64790]	22607462	22610557	38.47
AT1G08377			22612258	22612474	34.1
AT1G61310		LRR and NB-ARC domains-containing disease resistance protein [Source:TAIR;Acc:AT1G61310]	22612998	22616373	39.96
AT1G61320		FBD-associated F-box protein At1g61320 [Source:UniProtKB/Swiss-Prot;Acc:O64788]	22617370	22619853	34.9
AT1G08383			22620085	22620307	47.53
AT1G61330		Putative FBD-associated F-box protein At1g61330 [Source:UniProtKB/Swiss-Prot;Acc:O64787]	22622975	22624527	40.18
AT1G61340		F-box protein At1g61340 [Source:UniProtKB/Swiss-Prot;Acc:Q8GX77]	22628264	22630020	34.83
AT1G61350		ARM repeat superfamily protein [Source:UniProtKB/TrEMBL;Acc:O64785]	22633774	22636101	38.83
AT1G61360		Serine/threonine-protein kinase [Source:UniProtKB/TrEMBL;Acc:A0A178W9H1]	22637688	22641999	39.01
AT1G61370		G-type lectin S-receptor-like serine/threonine-protein kinase At1g61370 [Source:UniProtKB/Swiss-Prot;Acc:O64783]	22641843	22645922	39.04
AT1G61380	SD129	G-type lectin S-receptor-like serine/threonine-protein kinase SD1-29 [Source:UniProtKB/Swiss-Prot;Acc:O64782]	22645923	22650072	38.48
AT1G61390		G-type lectin S-receptor-like serine/threonine-protein kinase At1g61390 [Source:UniProtKB/Swiss-Prot;Acc:O64781]	22650111	22654395	39.42
AT1G61400		S-locus lectin protein kinase family protein [Source:TAIR;Acc:AT1G61400]	22654507	22658469	39.59
AT1G61410		At1g61410 [Source:UniProtKB/TrEMBL;Acc:Q058G5]	22658552	22659552	43.76
AT1G61415		unknown protein; FUNCTIONS IN: molecular_function unknown; INVOLVED IN: biological_process unknown; LOCATEI	22659661	22660967	38.94
AT1G61420		S-locus lectin protein kinase family protein [Source:TAIR;Acc:AT1G61420]	22660460	22664502	39.45
AT1G08387			22662953	22664539	38.69
AT1G61430		G-type lectin S-receptor-like serine/threonine-protein kinase At1g61430 [Source:UniProtKB/Swiss-Prot;Acc:O64777]	22664501	22668651	39.32
AT1G61440		S-locus lectin protein kinase family protein [Source:TAIR;Acc:AT1G61440]	22669134	22673111	39.22
AT1G61450		unknown protein; BEST Arabidopsis thaliana protein match is: unknown protein (TAIR:AT1G61415.1); Ha. [Source:TAIR;A	22672992	22674109	39.53
AT1G61460		G-type lectin S-receptor-like serine/threonine-protein kinase At1g61460 [Source:UniProtKB/Swiss-Prot;Acc:O64774]	22674268	22677188	39.37
AT1G61470	CAF1-5	Probable CCR4-associated factor 1 homolog 5 [Source:UniProtKB/Swiss-Prot;Acc:O64773]	22678092	22679302	37.99
AT1G61475		ATP binding / protein kinase [Source:UniProtKB/TrEMBL;Acc:F4HVB3]	22679770	22680771	38.32
AT1G61480		G-type lectin S-receptor-like serine/threonine-protein kinase At1g61480 [Source:UniProtKB/Swiss-Prot;Acc:O64771]	22681256	22684626	38.98
AT1G61490		G-type lectin S-receptor-like serine/threonine-protein kinase At1g61490 [Source:UniProtKB/Swiss-Prot;Acc:O64770]	22684894	22689611	38.94
AT1G61500		S-locus lectin protein kinase family protein [Source:TAIR;Acc:AT1G61500]	22689623	22693799	39.41
AT1G61520	LHCA3	Chlorophyll a-b binding protein, chloroplastic [Source:UniProtKB/TrEMBL;Acc:A0A178W5Y6]	22699715	22701412	41.87
AT1G61540		Putative F-box/kelch-repeat protein At1g61540 [Source:UniProtKB/Swiss-Prot;Acc:Q9SY96]	22702498	22703706	42.76
AT1G61550		G-type lectin S-receptor-like serine/threonine-protein kinase At1g61550 [Source:UniProtKB/Swiss-Prot;Acc:Q9SY95]	22704198	22707829	39.51
AT1G61560	MLO6	MLO-like protein 6 [Source:UniProtKB/Swiss-Prot;Acc:Q94KB7]	22708468	22712289	36.45
AT1G61562			22709009	22710122	38.24
AT1G61563	RALFL8	Protein RALF-like 8 [Source:UniProtKB/Swiss-Prot;Acc:Q1ECR9]	22714921	22715448	36.17
AT1G61565			22715075	22717595	31.42
AT1G61566	RALFL9	Protein RALF-like 9 [Source:UniProtKB/Swiss-Prot;Acc:Q3ECL0]	22717060	22717610	33.94
AT1G61570	TIM13	Mitochondrial import inner membrane translocase subunit TIM13 [Source:UniProtKB/Swiss-Prot;Acc:Q9XH48]	22718462	22719535	36.22
AT1G61575		Serine/Threonine kinase kinase ATP-binding sugar-binding kinase carbohydrate-binding protein [Source:UniProtKB/TrEMBI	22719765	22720575	37.85
AT1G61580	ARP2	60S ribosomal protein L3-2 [Source:UniProtKB/Swiss-Prot;Acc:P22738]	22720560	22723152	37.95
AT1G61590	PBL15	Probable serine/threonine-protein kinase PBL15 [Source:UniProtKB/Swiss-Prot;Acc:Q9SY91]	22723368	22726190	35.92
AT1G08393			22728734	22729039	39.54
AT1G61600		DUF1262 family protein (DUF1262) [Source:UniProtKB/TrEMBL;Acc:Q9SY90]	22729816	22731332	41.99
AT1G61610		Serine/threonine-protein kinase [Source:UniProtKB/TrEMBL;Acc:A0A178W8X6]	22733359	22736858	39.51
AT1G61620	CSU1	Nitric oxide synthase-interacting protein homolog [Source:UniProtKB/TrEMBL;Acc:A0A178WN49]	22737475	22739443	40.12
AT1G08397			22740621	22740823	42.86
AT1G08403			22741575	22741816	37.19
AT1G08413			22741682	22741922	42.74
AT1G08417			22743172	22743472	40.86
AT1G61630	ENT7	Equilibrative nucleotide transporter 7 [Source:UniProtKB/Swiss-Prot;Acc:Q944P0]	22744207	22746147	38.69
AT1G61640		Protein kinase superfamily protein [Source:UniProtKB/TrEMBL;Acc:Q8L6Y8]	22746340	22749264	40.1
AT1G61660	BHLH112	Transcription factor bHLH112 [Source:UniProtKB/Swiss-Prot;Acc:Q94JL3]	22753611	22756387	34.64
AT1G61667		Protein of unknown function, DUF538 [Source:TAIR;Acc:AT1G61667]	22767464	22768645	33.08
AT1G61670		Lung seven transmembrane receptor family protein [Source:UniProtKB/TrEMBL;Acc:Q8GYD0]	22769364	22772426	36.14
AT1G08427			22771937	22772131	38.97
AT1G61680	TPS14	S-(+)-linalool synthase, chloroplastic [Source:UniProtKB/Swiss-Prot;Acc:Q84UV0]	22772315	22774720	37.57
AT1G61688		Defensin-like protein 106 [Source:UniProtKB/Swiss-Prot;Acc:Q1G3Y1]	22780732	22781388	31.81
AT1G61690		At1g61690 [Source:UniProtKB/TrEMBL;Acc:Q6NPS1]	22782427	22786942	41.56
AT1G61700	NRPB10L	DNA-directed RNA polymerase subunit 10-like protein [Source:UniProtKB/Swiss-Prot;Acc:Q9SYA6]	22787012	22788380	33.97
AT1G61710		Cysteine/Histidine-rich C1 domain family protein [Source:UniProtKB/TrEMBL;Acc:Q9SYA7]	22788859	22790576	37.66
AT1G61720	BAN	Anthocyanidin reductase [Source:UniProtKB/Swiss-Prot;Acc:Q9SEV0]	22790966	22792803	37.05
AT1G61730		Probable transcription factor At1g61730 [Source:UniProtKB/Swiss-Prot;Acc:Q9SYA9]	22793170	22794714	39.55
AT1G61732	MIR776A	MIR776a; miRNA [Source:TAIR;Acc:AT1G61732]	22795618	22795735	35.59
AT1G61740		Sulfite exporter TauE/SafE family protein 2 [Source:UniProtKB/Swiss-Prot;Acc:Q9SYB0]	22798035	22801416	33.32
AT1G61750	CRRSP1	Cysteine-rich repeat secretory protein 1 [Source:UniProtKB/Swiss-Prot;Acc:Q9SYB1]	22804427	22806351	34.86
AT1G61760		Late embryogenesis abundant (LEA) hydroxyproline-rich glycoprotein family [Source:UniProtKB/TrEMBL;Acc:Q9SYB2]	22807193	22808280	39.43
AT1G61770	C50	Chaperone protein dnaJ 50 [Source:UniProtKB/Swiss-Prot;Acc:Q8GUN6]	22810111	22812677	36.66
AT1G61780		Postsynaptic protein CRIPT, putative [Source:UniProtKB/TrEMBL;Acc:Q9SYB4]	22812573	22814162	34.03
AT1G61790	OST3B	Probable dolichyl-diphosphooligosaccharide--protein glycosyltransferase subunit 3B [Source:UniProtKB/Swiss-Prot;Acc:Q9S	22814216	22816688	38.66
AT1G61795	RIC9	CRIB domain-containing protein RIC9 [Source:UniProtKB/Swiss-Prot;Acc:Q1G3Y0]	22819601	22821053	30.42
AT1G61800	GPT2	glucose-6-phosphate/phosphate translocator 2 [Source:TAIR;Acc:AT1G61800]	22824414	22826902	38.45
AT1G61810	BGLU45	Beta-glucosidase 45 [Source:UniProtKB/TrEMBL;Acc:F4HVG0]	22829921	22834728	30.49

AT1G08433			22833811	22834062	26.19
AT1G08437			22834539	22834809	39.85
AT1G61820	BGLU46	Beta-glucosidase 46 [Source:UniProtKB/Swiss-Prot;Acc:O80690]	22835078	22838635	31.08
AT1G61840		Cysteine/Histidine-rich C1 domain family protein [Source:UniProtKB/TrEMBL;Acc:O80692]	22847247	22849989	37.91
AT1G61850		phospholipases;galactolipases [Source:TAIR;Acc:AT1G61850]	22855751	22862398	39.97
AT1G61860		Protein kinase superfamily protein [Source:UniProtKB/TrEMBL;Acc:F4HX16]	22862655	22864898	39.62
AT1G61870	PPR336	Pentatricopeptide repeat-containing protein At1g61870, mitochondrial [Source:UniProtKB/Swiss-Prot;Acc:Q8LE47]	22864951	22866666	39.39
AT1G61880		pre-tRNA [Source:TAIR;Acc:AT1G61880]	22866758	22866829	56.94
AT1G08443			22867316	22867541	40.71
AT1G61890	DTX37	Protein DETOXIFICATION 37 [Source:UniProtKB/Swiss-Prot;Acc:O80695]	22867689	22871358	34.66
AT1G61900		Uncharacterized GPI-anchored protein At1g61900 [Source:UniProtKB/Swiss-Prot;Acc:Q8GUI4]	22882176	22885125	37.97
AT1G61910		pre-tRNA [Source:TAIR;Acc:AT1G61910]	22887090	22887169	53.75
AT1G61920		Putative uncharacterized protein [Source:UniProtKB/TrEMBL;Acc:Q5XVH7]	22888452	22889037	42.83
AT1G61930		At1g61930 [Source:UniProtKB/TrEMBL;Acc:O80698]	22893101	22894064	43.05
AT1G61940	TULP4	Putative Tubby-like protein 4 [Source:UniProtKB/Swiss-Prot;Acc:O80699]	22897399	22898725	40.92
AT1G61950	CPK19	Calcium-dependent protein kinase 19 [Source:UniProtKB/Swiss-Prot;Acc:Q1PFH8]	22899389	22902118	36.96
AT1G61960		At1g61960 [Source:UniProtKB/TrEMBL;Acc:O80701]	22902150	22903960	38.87
AT1G61970		At1g61970 [Source:UniProtKB/TrEMBL;Acc:O80702]	22904449	22907730	38.36
AT1G61980		At1g61980 [Source:UniProtKB/TrEMBL;Acc:O80703]	22908018	22910878	38.2
AT1G61990		At1g61990/F8K4_18 [Source:UniProtKB/TrEMBL;Acc:O80704]	22911159	22913122	39.31
AT1G62000		UPF0540 protein At1g62000 [Source:UniProtKB/Swiss-Prot;Acc:Q39168]	22913359	22914146	40.61
AT1G08447			22914366	22915032	35.68
AT1G62010		F8K4.20 protein [Source:UniProtKB/TrEMBL;Acc:O80705]	22915699	22917363	40.36
AT1G08453			22918356	22918592	41.77
AT1G62020		Coatomer subunit alpha [Source:UniProtKB/TrEMBL;Acc:A0A178WAY4]	22919487	22924024	42.66
AT1G62030		Cysteine/Histidine-rich C1 domain family protein [Source:UniProtKB/TrEMBL;Acc:O80707]	22924039	22926625	40.51
AT1G62035	MIR171C	MIR171C; miRNA [Source:TAIR;Acc:AT1G62035]	22930089	22930204	40.52
AT1G62040	ATG8C	Autophagy-related protein [Source:UniProtKB/TrEMBL;Acc:A0A178W309]	22932760	22934549	34.92
AT1G62045		Ankyrin repeat protein [Source:UniProtKB/TrEMBL;Acc:Q8GYM5]	22934667	22935458	33.08
AT1G62050		Ankyrin repeat family protein [Source:UniProtKB/TrEMBL;Acc:Q94CF4]	22936049	22939024	42.07
AT1G62060		UPF0540 protein At1g62060 [Source:UniProtKB/Swiss-Prot;Acc:O04573]	22941569	22942300	41.39
AT1G62070		F19K23.2 protein [Source:UniProtKB/TrEMBL;Acc:O04574]	22943594	22944289	39.66
AT1G62080		UPF0540 protein At1g62080 [Source:UniProtKB/Swiss-Prot;Acc:O04575]	22946242	22946949	42.23
AT1G08457			22947414	22947891	38.08
AT1G08463			22947519	22947748	35.65
AT1G62085		F19K23.4 protein [Source:UniProtKB/TrEMBL;Acc:O04576]	22947843	22950488	38.44
AT1G08473			22955428	22955842	37.83
AT1G62110		At1g62110 [Source:UniProtKB/TrEMBL;Acc:Q8GY68]	22957876	22959884	37.83
AT1G62120		At1g62120 [Source:UniProtKB/TrEMBL;Acc:Q6DBE5]	22960139	22962103	41.02
AT1G62130		AAA-type ATPase family protein [Source:UniProtKB/TrEMBL;Acc:F4HX45]	22962129	22968998	37.9
AT1G62150		Mitochondrial transcription termination factor family protein [Source:UniProtKB/TrEMBL;Acc:Q8GWB0]	22969830	22971771	38.83
AT1G62160		Serine protease inhibitor (SERPIN) family protein [Source:UniProtKB/TrEMBL;Acc:F4HX47]	22972352	22973303	41.81
AT1G62170		Serine protease inhibitor (SERPIN) family protein [Source:UniProtKB/TrEMBL;Acc:F4HX48]	22973840	22975754	37.86
AT1G62180	02-Apr	5'-adenylylsulfate reductase 2, chloroplastic [Source:UniProtKB/Swiss-Prot;Acc:P92981]	22975530	22977885	40.24
AT1G08477			22975698	22976479	46.16
AT1G62190	FAD4L1	Fatty acid desaturase 4-like 1, chloroplastic [Source:UniProtKB/Swiss-Prot;Acc:O04584]	22980817	22982007	42.74
AT1G62200		Major facilitator superfamily protein [Source:TAIR;Acc:AT1G62200]	22980934	22984422	40.64
AT1G62210		F19K23.14 protein [Source:UniProtKB/TrEMBL;Acc:O04586]	22986002	22986861	37.09
AT1G62220		UPF0540 protein At1g62220 [Source:UniProtKB/Swiss-Prot;Acc:O04587]	22988196	22988844	42.06
AT1G08483			22988981	22989332	38.07
AT1G62225		At1g62225 [Source:UniProtKB/TrEMBL;Acc:O04588]	22989550	22990341	37.88
AT1G62250		F19K23.17 protein [Source:UniProtKB/TrEMBL;Acc:O04589]	22994538	22997709	39.41
AT1G62240		Putative uncharacterized protein At1g62240 [Source:UniProtKB/TrEMBL;Acc:Q84W21]	22994544	22995471	48.28
AT1G62260	PCMP-E10	Pentatricopeptide repeat-containing protein At1g62260, mitochondrial [Source:UniProtKB/Swiss-Prot;Acc:O04590]	22997755	22999829	42.46
AT1G62262	SLAH4	S-type anion channel SLAH4 [Source:UniProtKB/Swiss-Prot;Acc:A8MRV9]	23000157	23002096	37.16
AT1G08487			23003505	23003714	44.76
AT1G62270		Putative F-box/kelch-repeat protein At1g62270 [Source:UniProtKB/Swiss-Prot;Acc:O04591]	23004499	23005650	38.72
AT1G08493			23006714	23006912	43.72
AT1G62280	SLAH1	SLAH1 [Source:UniProtKB/TrEMBL;Acc:A0A178W8P4]	23007044	23008610	42.12
AT1G62290	APA2	Aspartic proteinase A2 [Source:UniProtKB/Swiss-Prot;Acc:Q8VYL3]	23009885	23013601	36.64
AT1G62300	WRKY6	Uncharacterized protein At1g62300 (Fragment) [Source:UniProtKB/TrEMBL;Acc:C0SV11]	23016569	23019494	39.27
AT1G08497			23023932	23024189	34.11
AT1G62305		At1g62305 [Source:UniProtKB/TrEMBL;Acc:Q6DST3]	23026692	23029531	37.11
AT1G08503			23030492	23030787	34.8
AT1G08507			23032347	23033035	36.28
AT1G62310		Transcription factor jumonji (JmjC) domain-containing protein (Fragment) [Source:UniProtKB/TrEMBL;Acc:C0SV12]	23033403	23039787	37.23
AT1G08513			23034689	23035381	42.57
AT1G62320		CSC1-like protein At1g62320 [Source:UniProtKB/Swiss-Prot;Acc:F4HYR3]	23041408	23045426	37.3
AT1G62330	OFUT15	O-fucosyltransferase 15 [Source:UniProtKB/Swiss-Prot;Acc:F4HYR4]	23046768	23050337	39.66
AT1G62333		F24O1.6 [Source:UniProtKB/TrEMBL;Acc:Q9MAV4]	23050381	23050940	41.61
AT1G62340	SBT2.4	Subtilisin-like protease SBT2.4 [Source:UniProtKB/Swiss-Prot;Acc:F4HYR6]	23051002	23055672	37.57
AT1G62350		Pentatricopeptide repeat (PPR) superfamily protein [Source:TAIR;Acc:AT1G62350]	23056386	23058114	38.23
AT1G62355	MIR413	MIR413; miRNA [Source:TAIR;Acc:AT1G62355]	23058034	23058156	42.28
AT1G62360	STM	Homeobox protein SHOOT MERISTEMLESS [Source:UniProtKB/Swiss-Prot;Acc:Q38874]	23058582	23062063	36.04
AT1G08517			23068047	23068354	35.71
AT1G62370		F2401.10 [Source:UniProtKB/TrEMBL;Acc:O48801]	23072222	23073545	36.63
AT1G08523			23077196	23077601	30.3
AT1G62380	ACO2	1-aminocyclopropane-1-carboxylate oxidase 2 [Source:UniProtKB/Swiss-Prot;Acc:Q41931]	23082036	23084253	35.75
AT1G62390	Phox2	Protein CLMP1 [Source:UniProtKB/Swiss-Prot;Acc:O48802]	23084293	23087141	41.84
AT1G62400	HT1	Serine/threonine-protein kinase HT1 [Source:UniProtKB/Swiss-Prot;Acc:Q2MHE4]	23089610	23091732	40.79
AT1G62410		MIF4G domain-containing protein [Source:UniProtKB/TrEMBL;Acc:F4HYS3]	23092915	23093869	38.53
AT1G62421		Putative uncharacterized protein [Source:UniProtKB/TrEMBL;Acc:Q1G3Y3]	23099484	23099889	38.42
AT1G62422		F2401.15 [Source:UniProtKB/TrEMBL;Acc:Q9MAV1]	23100119	23101335	37.55
AT1G62420		DUF506 family protein (DUF506) [Source:UniProtKB/TrEMBL;Acc:O48807]	23102777	23104756	35.15
AT1G62430	CDS1	Phosphatidate cytidylyltransferase 1 [Source:UniProtKB/Swiss-Prot;Acc:O04928]	23105905	23109534	36.14
AT1G62440	LRX2	Leucine-rich repeat extensin-like protein 2 [Source:UniProtKB/Swiss-Prot;Acc:O48809]	23112933	23115293	47.86
AT1G62450		Immunoglobulin E-set superfamily protein [Source:UniProtKB/TrEMBL;Acc:F4HYS9]	23115536	23117594	34.53
AT1G62480		At1g62480/T3P18_4 [Source:UniProtKB/TrEMBL;Acc:Q9SXE9]	23128651	23129759	37.6
AT1G62490		Mitochondrial transcription termination factor family protein [Source:UniProtKB/TrEMBL;Acc:Q9SXE8]	23129872	23131533	38.57
AT1G62500		Bifunctional inhibitor/lipid-transfer protein/seed storage 2S albumin superfamily protein [Source:UniProtKB/TrEMBL;Acc:Q	23131928	23133507	44.87
AT1G62510		Bifunctional inhibitor/lipid-transfer protein/seed storage 2S albumin superfamily protein [Source:UniProtKB/TrEMBL;Acc:Q	23136366	23137254	40.04
AT1G62515		Transmembrane protein [Source:UniProtKB/TrEMBL;Acc:A0A1P8ATX8]	23136720	23137413	42.07
AT1G62520		Sulfated surface-like glycoprotein [Source:UniProtKB/TrEMBL;Acc:Q9SXE5]	23144385	23145531	44.03
AT1G62530		Putative uncharacterized protein [Source:UniProtKB/TrEMBL;Acc:Q1PFH6]	23146200	23148367	34.18
AT1G62540	FMO GS-OX2	flavin-monooxygenase glucosinolate S-oxygenase 2 [Source:TAIR;Acc:AT1G62540]	23151634	23155728	33.63
AT1G62560	FMOGS-OX3	Flavin-containing monooxygenase FMO GS-OX3 [Source:UniProtKB/Swiss-Prot;Acc:Q9SXE1]	23159734	23162881	35.8
AT1G62570	FMOGS-OX4	Flavin-containing monooxygenase FMO GS-OX4 [Source:UniProtKB/Swiss-Prot;Acc:Q93Y23]	23168767	23172175	34.76
AT1G62580		Flavin-binding monooxygenase family protein [Source:TAIR;Acc:AT1G62580]	23173286	23176931	32.78

AT1G08533			23173397	23173691	55.25
AT1G62590		Pentatricopeptide repeat-containing protein At1g62590 [Source:UniProtKB/Swiss-Prot;Acc:Q9SXD8]	23176930	23179248	39.76
AT1G08537			23177935	23178261	38.53
AT1G08543			23179399	23180068	47.16
AT1G62600		Flavin-containing monooxygenase FMO GS-OX-like 4 [Source:UniProtKB/Swiss-Prot;Acc:Q94BV5]	23179461	23181519	42.01
AT1G62610		NAD(P)-binding Rossmann-fold superfamily protein [Source:UniProtKB/TrEMBL;Acc:F4HYU6]	23181463	23183227	43.63
AT1G08547			23181614	23181887	47.08
AT1G62620		Flavin-containing monooxygenase FMO GS-OX-like 3 [Source:UniProtKB/Swiss-Prot;Acc:Q9SXD5]	23182644	23185151	37.2
AT1G62630		Probable disease resistance protein At1g62630 [Source:UniProtKB/Swiss-Prot;Acc:Q9SI85]	23185760	23189224	38.79
AT1G62640	KAS III	3-oxoacyl-[acyl-carrier-protein] synthase III, chloroplastic [Source:UniProtKB/Swiss-Prot;Acc:P49243]	23192146	23195103	37.73
AT1G62660	BFRUCT3	Acid beta-fructofuranosidase 3, vacuolar [Source:UniProtKB/Swiss-Prot;Acc:Q43348]	23199612	23203808	36.81
AT1G62670	RPF2	Pentatricopeptide repeat-containing protein At1g62670, mitochondrial [Source:UniProtKB/Swiss-Prot;Acc:Q9SXD1]	23204256	23206822	39.93
AT1G62680		Pentatricopeptide repeat-containing protein At1g62680, mitochondrial [Source:UniProtKB/Swiss-Prot;Acc:Q3ECK2]	23207017	23209987	38.54
AT1G62690		unknown protein; Ha. [Source:TAIR;Acc:AT1G62690]	23210414	23211395	36.25
AT1G62700	NAC026	NAC domain-containing protein 26 [Source:UniProtKB/Swiss-Prot;Acc:F4HYV5]	23215953	23219209	34.23
AT1G08553			23219062	23219393	39.76
AT1G62710	bVPE	Vacuolar-processing enzyme beta-isozyme [Source:UniProtKB/Swiss-Prot;Acc:Q39044]	23223874	23226983	36.08
AT1G62720		Pentatricopeptide repeat-containing protein At1g62720 [Source:UniProtKB/Swiss-Prot;Acc:Q9SI78]	23227532	23229150	38.23
AT1G08557			23228101	23228395	46.1
AT1G62730		At1g62730 [Source:UniProtKB/TrEMBL;Acc:Q9SI77]	23229059	23230818	39.43
AT1G62740	HOP2	Hsp70-Hsp90 organizing protein 2 [Source:UniProtKB/Swiss-Prot;Acc:Q5XEP2]	23230922	23233543	41
AT1G62750	CPEFG	Elongation factor G, chloroplastic [Source:UniProtKB/Swiss-Prot;Acc:Q9SI75]	23233434	23236447	42.37
AT1G62760	PMEI10	Pectinesterase inhibitor 10 [Source:UniProtKB/Swiss-Prot;Acc:Q9SI74]	23237372	23238644	38.88
AT1G62770	PMEI9	Pectinesterase inhibitor 9 [Source:UniProtKB/Swiss-Prot;Acc:Q9SI72]	23245835	23246949	38.21
AT1G62780		Dimethylallyl, adenosine tRNA methylthiotransferase [Source:UniProtKB/TrEMBL;Acc:Q8VY70]	23249145	23251195	35.84
AT1G62790		At1g62790 [Source:UniProtKB/TrEMBL;Acc:Q6NLF7]	23252140	23253743	33.29
AT1G08563			23253055	23253291	27.85
AT1G62800	ASP4	Aspartate aminotransferase [Source:UniProtKB/TrEMBL;Acc:F4I0D4]	23253703	23257546	32.34
AT1G08567			23254364	23254714	29.06
AT1G62810		Primary amine oxidase [Source:UniProtKB/Swiss-Prot;Acc:Q8H1H9]	23257610	23262002	38.72
AT1G08573			23262295	23263024	36.16
AT1G62820	CML14	Probable calcium-binding protein CML14 [Source:UniProtKB/Swiss-Prot;Acc:Q8VZ50]	23263214	23264399	39.38
AT1G62830	LDL1	Lysine-specific histone demethylase 1 homolog 1 [Source:UniProtKB/Swiss-Prot;Acc:Q8VXV7]	23264486	23267221	43.93
AT1G62840		Ankyrin repeat/KH domain protein (DUF1442) [Source:UniProtKB/TrEMBL;Acc:Q9SI67]	23271416	23272578	39.04
AT1G08577			23271551	23271796	32.93
AT1G62850		Class I peptide chain release factor [Source:UniProtKB/TrEMBL;Acc:Q84JF2]	23271703	23274340	38.86
AT1G62870		F16P17.2 protein [Source:UniProtKB/TrEMBL;Acc:Q9LQ19]	23283912	23286797	42.03
AT1G62880		Protein cornichon homolog 3 [Source:UniProtKB/Swiss-Prot;Acc:Q8GWT5]	23291900	23295266	35.94
AT1G62886		Nucleotide excision repair, TFIIH, subunit TTDA [Source:UniProtKB/TrEMBL;Acc:B3H572]	23294413	23294710	37.92
AT1G62895			23295989	23296858	31.38
AT1G62900		F16P17.4 protein [Source:UniProtKB/TrEMBL;Acc:Q9LQ17]	23297720	23298682	36.14
AT1G62910		Pentatricopeptide repeat-containing protein At1g62910 [Source:UniProtKB/Swiss-Prot;Acc:Q9LQ16]	23298948	23301141	39.33
AT1G62914		Pentatricopeptide repeat-containing protein At1g62914, mitochondrial [Source:UniProtKB/Swiss-Prot;Acc:Q9LQ15]	23301315	23303162	39.72
AT1G62915			23303241	23304287	35.91
AT1G62920		CONTAINS InterPro DOMAIN/s: Proteasome maturation factor UMP1 (InterPro:IPR008012); BEST Arabidopsis thaliana p	23304313	23306374	36.03
AT1G62930		Pentatricopeptide repeat-containing protein At1g62930, chloroplastic [Source:UniProtKB/Swiss-Prot;Acc:Q9LQ14]	23306534	23308686	40.04
AT1G62935		unknown protein; FUNCTIONS IN: molecular_function unknown; INVOLVED IN: biological_process unknown; LOCATEI	23308760	23309410	34.56
AT1G62940	4CLL1	4-coumarate--CoA ligase-like 1 [Source:UniProtKB/Swiss-Prot;Acc:Q9LQ12]	23310532	23312795	39.49
AT1G08583			23314785	23315063	41.94
AT1G62950		F16P17.10 protein [Source:UniProtKB/TrEMBL;Acc:Q9LQ11]	23314871	23318234	39.98
AT1G62960	ACS10	Probable aminotransferase ACS10 [Source:UniProtKB/Swiss-Prot;Acc:Q9LQ10]	23318187	23320427	41.23
AT1G62970		Chaperone DnaJ-domain superfamily protein [Source:UniProtKB/TrEMBL;Acc:Q0WQM3]	23323258	23326188	46.81
AT1G62975	BHLH125	Transcription factor bHLH125 [Source:UniProtKB/Swiss-Prot;Acc:Q9LQ08]	23328727	23330812	30.54
AT1G62978		unknown protein; Ha. [Source:TAIR;Acc:AT1G62978]	23331462	23331608	34.01
AT1G62980	EXPA18	Expansin-A18 [Source:UniProtKB/Swiss-Prot;Acc:Q9LQ07]	23331563	23333246	38.06
AT1G62981		F16P17.15 [Source:UniProtKB/TrEMBL;Acc:Q9LQ06]	23333619	23335395	39.73
AT1G62990	KNAT7	Homeobox protein knotted-1-like 7 [Source:UniProtKB/Swiss-Prot;Acc:Q9FPQ8]	23337167	23340692	32.98
AT1G63000	NRS/ER	Bifunctional dTDP-4-dehydrorhamnose 3,5-epimerase/dTDP-4-dehydrorhamnose reductase [Source:UniProtKB/Swiss-Prot;A	23342211	23344120	37.17
AT1G63005	MIR399B	MIR399B; miRNA [Source:TAIR;Acc:AT1G63005]	23345377	23345511	40
AT1G63010		Major Facilitator Superfamily with SPX (SYG1/Pho81/XPR1) domain-containing protein [Source:UniProtKB/TrEMBL;Acc:	23347561	23352831	36.65
AT1G63020	NRPD1	DNA-directed RNA polymerase IV subunit 1 [Source:UniProtKB/Swiss-Prot;Acc:Q9LQ02]	23354685	23362589	39.28
AT1G08587			23365725	23365930	37.38
AT1G63030	DREB1E	Dehydration-responsive element-binding protein 1E [Source:UniProtKB/Swiss-Prot;Acc:Q9SGJ6]	23367394	23368416	43.01
AT1G63050	LPLAT2	LPLAT2 [Source:UniProtKB/TrEMBL;Acc:A0A178WEX3]	23375877	23378355	39.85
AT1G63055		Transmembrane protein [Source:UniProtKB/TrEMBL;Acc:Q5XVH6]	23379665	23380669	28.56
AT1G63057		unknown protein; FUNCTIONS IN: molecular_function unknown; INVOLVED IN: biological_process unknown; LOCATEI	23379665	23383179	27.82
AT1G63060		Ribosome biogenesis NEP1-like protein [Source:UniProtKB/TrEMBL;Acc:Q9CAN7]	23383971	23385109	35.38
AT1G63070		Pentatricopeptide repeat-containing protein At1g63070, mitochondrial [Source:UniProtKB/Swiss-Prot;Acc:Q9CAN6]	23385324	23387167	41.38
AT1G63080		Pentatricopeptide repeat-containing protein At1g63080, mitochondrial [Source:UniProtKB/Swiss-Prot;Acc:Q9CAN5]	23387574	23390838	38.13
AT1G63090	PP2A11	F-box protein PP2-A11 [Source:UniProtKB/Swiss-Prot;Acc:Q9CAN4]	23390968	23392873	37.41
AT1G63100	SCL28	Scarecrow-like protein 28 [Source:UniProtKB/Swiss-Prot;Acc:Q9CAN3]	23399112	23402176	40.98
AT1G63105		unknown protein; Ha. [Source:TAIR;Acc:AT1G63105]	23404141	23404537	39.8
AT1G63110		GPI transamidase subunit PIG-U [Source:UniProtKB/TrEMBL;Acc:F4I1Z0]	23404688	23408119	37.79
AT1G63120	RBL2	RHOMBOID-like protein 2 [Source:UniProtKB/Swiss-Prot;Acc:Q9CAN1]	23408804	23411139	37.03
AT1G63130		Pentatricopeptide repeat-containing protein At1g63130, mitochondrial [Source:UniProtKB/Swiss-Prot;Acc:Q9CAN0]	23412518	23415155	39.2
AT1G08597			23412989	23413436	41.52
AT1G08603			23415640	23415849	39.05
AT1G63140		O-methyltransferase family protein [Source:TAIR;Acc:AT1G63140]	23417441	23419045	39.19
AT1G63150		Pentatricopeptide repeat-containing protein At1g63150 [Source:UniProtKB/Swiss-Prot;Acc:Q9CAM8]	23419294	23421714	39.03
AT1G63160	RFC2	Replication factor C subunit 2 [Source:UniProtKB/Swiss-Prot;Acc:Q9CAM7]	23421834	23423914	38.97
AT1G63170		E3 ubiquitin-protein ligase At1g63170 [Source:UniProtKB/Swiss-Prot;Acc:Q8LDB8]	23425352	23427408	38.79
AT1G63180	UGE3	UGE3 [Source:UniProtKB/TrEMBL;Acc:A0A178WIC1]	23427405	23429652	35.19
AT1G63190		Cystatin/monellin superfamily protein [Source:TAIR;Acc:AT1G63190]	23431730	23433178	36.58
AT1G63200		Cystatin/monellin superfamily protein [Source:UniProtKB/TrEMBL;Acc:Q9CAM3]	23433520	23434600	37.65
AT1G63205		Cystatin/monellin superfamily protein [Source:UniProtKB/TrEMBL;Acc:Q9CAM2]	23437689	23438677	37.61
AT1G63206		Cystatin/monellin superfamily protein [Source:TAIR;Acc:AT1G63206]	23439526	23440931	36.77
AT1G63210		Transcription elongation factor SPT6-like [Source:UniProtKB/Swiss-Prot;Acc:Q9CAM1]	23443688	23447354	40.03
AT1G63220		C2 domain-containing protein At1g63220 [Source:UniProtKB/Swiss-Prot;Acc:Q9C8S6]	23448812	23450530	36.71
AT1G63230		At1g63230 [Source:UniProtKB/TrEMBL;Acc:Q0IGJ6]	23450560	23452372	40.93
AT1G63240		Uncharacterized protein At1g63240 [Source:UniProtKB/TrEMBL;Acc:Q949Y1]	23455711	23458928	38.32
AT1G63245	CLE14	CLE14 [Source:UniProtKB/TrEMBL;Acc:A0A178WFK0]	23460198	23460884	33.19
AT1G63250	RH48	Probable DEAD-box ATP-dependent RNA helicase 48 [Source:UniProtKB/Swiss-Prot;Acc:Q9C8S9]	23463023	23466508	39.21
AT1G63260	TET10	Tetraspanin-10 [Source:UniProtKB/Swiss-Prot;Acc:F4I214]	23466700	23469289	36.33
AT1G63270	ABCI1	ABC transporter I family member 1 [Source:UniProtKB/Swiss-Prot;Acc:Q9C8T1]	23469554	23471510	38.58
AT1G63280		Serine protease inhibitor (SERPIN) family protein [Source:UniProtKB/TrEMBL;Acc:Q9C8T2]	23471343	23471705	41.32
AT1G63290		Ribulose-phosphate 3-epimerase [Source:UniProtKB/TrEMBL;Acc:Q9C8T3]	23471844	23473922	40.69
AT1G63295		Remorin family protein [Source:TAIR;Acc:AT1G63295]	23473975	23475532	29.4
AT1G63300		Myosin heavy chain-related protein [Source:UniProtKB/TrEMBL;Acc:Q9C8T4]	23481907	23486220	38.92

	AT1G63310		Uncharacterized protein At1g63310/F9N12_7 [Source:UniProtKB/TrEMBL;Acc:Q9C8T5]	23486244	23487091	41.75
	AT1G63320		Pentatricopeptide repeat (PPR) superfamily protein [Source:UniProtKB/TrEMBL;Acc:F4I221]	23488884	23489578	40.14
MQTL-8/Chr1	AT1G74510		F-box/kelch-repeat protein At1g74510 [Source:UniProtKB/Swiss-Prot;Acc:Q9CA63]	28005264	28007748	39.8
	AT1G74520	HVA22A	HVA22-like protein a [Source:UniProtKB/Swiss-Prot;Acc:Q9S7V4]	28007961	28009451	35.41
	AT1G74530		Transmembrane protein [Source:UniProtKB/TrEMBL;Acc:Q0WPJ3]	28009465	28012634	35.62
	AT1G09477			28012571	28012893	35.6
	AT1G74545		Potential natural antisense gene, locus overlaps with AT1G74540 [Source:TAIR;Acc:AT1G74545]	28013118	28015826	41.01
	AT1G74540	CYP98A8	Cytochrome P450 98A8 [Source:UniProtKB/Swiss-Prot;Acc:Q9CA61]	28013180	28015049	43.26
	AT1G74550	CYP98A9	Cytochrome P450 98A9 [Source:UniProtKB/Swiss-Prot;Acc:Q9CA60]	28016045	28017711	44.27
	AT1G09487			28016582	28017771	43.78
	AT1G74560	NRP1	NAP1-related protein 1 [Source:TAIR;Acc:AT1G74560]	28017584	28020059	36.47
	AT1G74570		pre-tRNA [Source:TAIR;Acc:AT1G74570]	28020383	28020463	51.85
	AT1G74580		Putative pentatricopeptide repeat-containing protein At1g74580 [Source:UniProtKB/Swiss-Prot;Acc:Q9CA58]	28020548	28023905	39.43
	AT1G74590	GSTU10	Glutathione S-transferase U10 [Source:UniProtKB/Swiss-Prot;Acc:Q9CA57]	28023627	28024860	38.9
	AT1G74600	PCMP-E69	Pentatricopeptide repeat-containing protein At1g74600, chloroplastic [Source:UniProtKB/Swiss-Prot;Acc:Q9CA56]	28024840	28027869	40.43
	AT1G74610		pre-tRNA [Source:TAIR;Acc:AT1G74610]	28027960	28028040	54.32
	AT1G74620		Putative RING zinc finger protein; 84572-85321 [Source:UniProtKB/TrEMBL;Acc:Q9CA55]	28028252	28029001	44.27
	AT1G74630		Tetratricopeptide repeat (TPR)-like superfamily protein [Source:TAIR;Acc:AT1G74630]	28030465	28032556	42.5
	AT1G74640		Alpha/beta-Hydrolases superfamily protein [Source:UniProtKB/TrEMBL;Acc:Q8L475]	28032562	28034863	40.23
	AT1G74650	ATY13	At1g74650 [Source:UniProtKB/TrEMBL;Acc:Q9CA52]	28041146	28043022	36.44
	AT1G09493			28045332	28046073	33.29
	AT1G74660	MIF1	Mini zinc finger protein 1 [Source:UniProtKB/Swiss-Prot;Acc:Q9CA51]	28045472	28048289	33.11
	AT1G09497			28047169	28047416	31.45
	AT1G74670	GASA6	Gibberellin-regulated protein 6 [Source:UniProtKB/Swiss-Prot;Acc:Q6NMQ7]	28053030	28054149	31.34
	AT1G74675		Transmembrane protein [Source:UniProtKB/TrEMBL;Acc:A0A1P8AVR2]	28053944	28055437	30.92
	AT1G09503			28056529	28056771	36.21
	AT1G09507			28056529	28056805	34.66
	AT1G74680		At1g74680/F1M20_36 [Source:UniProtKB/TrEMBL;Acc:Q93ZD5]	28059108	28061130	38.95
	AT1G74690	IQD31	Protein IQ-DOMAIN 31 [Source:UniProtKB/Swiss-Prot;Acc:Q8L4D8]	28061159	28064771	38.42
	AT1G74700	TRZ1	At1g74700 [Source:UniProtKB/TrEMBL;Acc:Q1WW71]	28065014	28067423	36.89
	AT1G74710	EDS16	ADC synthase superfamily protein [Source:TAIR;Acc:AT1G74710]	28070295	28074177	37.11
	AT1G74720	QKY	Protein QUIRKY [Source:UniProtKB/Swiss-Prot;Acc:B8XCH5]	28074927	28078831	46.38
	AT1G74730		Transmembrane protein, putative (DUF1118) [Source:UniProtKB/TrEMBL;Acc:Q94F10]	28078852	28079987	41.55
	AT1G09513			28079103	28079431	33.43
	AT1G74740	CPK30	Calcium-dependent protein kinase 30 [Source:UniProtKB/Swiss-Prot;Acc:Q9SSF8]	28079946	28082671	39.69
	AT1G74750		Pentatricopeptide repeat-containing protein At1g74750 [Source:UniProtKB/Swiss-Prot;Acc:Q9SSF9]	28085753	28089642	41.26
	AT1G74770		Zinc finger protein BRUTUS-like At1g74770 [Source:UniProtKB/Swiss-Prot;Acc:F4HVS0]	28089172	28094956	38.48
	AT1G74780		F25A4.25 protein [Source:UniProtKB/TrEMBL;Acc:Q9SSG2]	28095729	28098225	41.61
	AT1G74790	HIPL1	HIPL1 protein [Source:UniProtKB/Swiss-Prot;Acc:Q9SSG3]	28098464	28101896	40.49
	AT1G74800	GALT5	Hydroxyproline O-galactosyltransferase GALT5 [Source:UniProtKB/Swiss-Prot;Acc:Q8RX55]	28101762	28105193	41.38
	AT1G74810	BOR5	Putative boron transporter 5 [Source:UniProtKB/Swiss-Prot;Acc:Q9SSG5]	28107822	28111940	39.38
	AT1G74820		Germin-like protein subfamily T member 3 [Source:UniProtKB/Swiss-Prot;Acc:Q9S772]	28111808	28112579	44.56
	AT1G74830	MYOB6	Probable myosin-binding protein 6 [Source:UniProtKB/Swiss-Prot;Acc:F4HVS6]	28113194	28115281	38.22
	AT1G74840		F25A4.19 protein [Source:UniProtKB/TrEMBL;Acc:Q9S7N6]	28115443	28117422	38.48
	AT1G74850	PTAC2	PTAC2 [Source:UniProtKB/TrEMBL;Acc:A0A178WNJ2]	28118664	28122577	42.21
	AT1G74860		At1g74860 [Source:UniProtKB/TrEMBL;Acc:Q9SSG6]	28123301	28126076	38.54
	AT1G74870		At1g74870 [Source:UniProtKB/TrEMBL;Acc:Q9S7I7]	28126928	28128790	38.81
	AT1G74875		FUNCTIONS IN: molecular_function unknown; INVOLVED IN: biological_process unknown; LOCATED IN: cellular_com	28128470	28129745	36.29
	AT1G74880	ndhO	NAD(P)H-quinone oxidoreductase subunit O, chloroplastic [Source:UniProtKB/Swiss-Prot;Acc:Q9S829]	28129828	28131077	39.52
	AT1G74890	ARR15	Two-component response regulator ARR15 [Source:UniProtKB/Swiss-Prot;Acc:Q7G8V2]	28131447	28133025	35.66
	AT1G74900	OTP43	Pentatricopeptide repeat (PPR) superfamily protein [Source:TAIR;Acc:AT1G74900]	28133933	28135667	43.34
	AT1G09517			28134653	28135270	42.39
	AT1G74910		ADP-glucose pyrophosphorylase family protein [Source:UniProtKB/TrEMBL;Acc:Q9C9P3]	28135361	28138971	38.74
	AT1G74920	ALDH10A8	ALDH10A8 [Source:UniProtKB/TrEMBL;Acc:A0A178W4Y2]	28138997	28142837	39.29
	AT1G74930	ERF018	Ethylene-responsive transcription factor ERF018 [Source:UniProtKB/Swiss-Prot;Acc:Q9S7L5]	28143851	28145058	42.3
	AT1G74929		unknown protein; Ha. [Source:TAIR;Acc:AT1G74929]	28143997	28144098	51.96
	AT1G74940	FLZ13	FCS-Like Zinc finger 13 [Source:UniProtKB/Swiss-Prot;Acc:Q8GRN0]	28145978	28147485	36.87
	AT1G74950	TIFY10B	TIFY10B [Source:UniProtKB/TrEMBL;Acc:A0A178WA69]	28148574	28150446	38.33
	AT1G74960	KAS2	3-oxoacyl-[acyl-carrier-protein] synthase II, chloroplastic [Source:UniProtKB/Swiss-Prot;Acc:Q9C9P4]	28151920	28156350	38.57
	AT1G74970	RPS9	30S ribosomal protein S9, chloroplastic [Source:UniProtKB/Swiss-Prot;Acc:Q9XJ27]	28157386	28159317	39.54
	AT1G74990		At1g74990 [Source:UniProtKB/TrEMBL;Acc:Q9S7D6]	28159715	28160510	37.56
	AT1G75000		F25A4.4 [Source:UniProtKB/TrEMBL;Acc:Q9S804]	28163344	28164814	43.17
	AT1G75010	ARC3	Protein ACCUMULATION AND REPLICATION OF CHLOROPLASTS 3 [Source:UniProtKB/Swiss-Prot;Acc:Q6F6B5]	28164828	28170113	38.14
	AT1G75020	LPAT4	Probable 1-acyl-sn-glycerol-3-phosphate acyltransferase 4 [Source:UniProtKB/Swiss-Prot;Acc:Q8L4Y2]	28170752	28173678	37.79
	AT1G75030	ATLP-3	At1g75030 [Source:UniProtKB/TrEMBL;Acc:Q9C9P9]	28174187	28175456	45.51
	AT1G75040	PR5	Pathogenesis-related protein 5 [Source:UniProtKB/Swiss-Prot;Acc:P28493]	28177670	28179022	43.02
	AT1G75050		Pathogenesis-related thaumatin superfamily protein [Source:UniProtKB/TrEMBL;Acc:Q1PFD2]	28180057	28181246	45.46
	AT1G75060		Histone deacetylase complex subunit [Source:UniProtKB/TrEMBL;Acc:Q9C9Q1]	28181275	28183517	36.38
	AT1G75070		pre-tRNA [Source:TAIR;Acc:AT1G75070]	28184532	28184603	61.11
	AT1G75080	BZR1	Protein BRASSINAZOLE-RESISTANT 1 [Source:UniProtKB/Swiss-Prot;Acc:Q8S307]	28185504	28188075	40.36
	AT1G09523			28186684	28186903	46.36
	AT1G75090		DNA glycosylase superfamily protein [Source:UniProtKB/TrEMBL;Acc:Q94CA9]	28187447	28189765	35.4
	AT1G09527			28188388	28188831	27.25
	AT1G75100	JAC1	JAC1 [Source:UniProtKB/TrEMBL;Acc:A0A178WHU1]	28190740	28194068	39.32
	AT1G75110	RRA2	Arabinosyltransferase RRA2 [Source:UniProtKB/Swiss-Prot;Acc:Q9C9Q5]	28194005	28196033	39.43
	AT1G75120	RRA1	Arabinosyltransferase RRA1 [Source:UniProtKB/Swiss-Prot;Acc:Q9C9Q6]	28196857	28198945	39.78
	AT1G75125			28199140	28199887	37.3
	AT1G75130	CYP721A1	CYP721A1 [Source:UniProtKB/TrEMBL;Acc:A0A178WDX2]	28199938	28201975	41.71
	AT1G75140		Uncharacterized membrane protein At1g75140 [Source:UniProtKB/Swiss-Prot;Acc:Q9FRK5]	28202053	28204362	42.68
	AT1G75150		unknown protein; Ha. [Source:TAIR;Acc:AT1G75150]	28204387	28208590	39.77
	AT1G75160		Protein of unknown function (DUF620) [Source:TAIR;Acc:AT1G75160]	28209407	28211826	38.76
	AT1G09533			28212219	28212357	36.69
	AT1G75163		snoRNA [Source:TAIR;Acc:AT1G75163]	28212395	28212474	35
	AT1G75166		snoRNA [Source:TAIR;Acc:AT1G75166]	28212518	28212603	45.35
	AT1G75170		Sec14p-like phosphatidylinositol transfer family protein [Source:UniProtKB/TrEMBL;Acc:Q9FRK8]	28213666	28215887	38.3
	AT1G09537			28213926	28214368	34.76
	AT1G75180		AT1G75180 protein [Source:UniProtKB/TrEMBL;Acc:Q9FRK9]	28215836	28218801	37.36
	AT1G09543			28216360	28219186	36.54
	AT1G75190		AT1G75190 protein [Source:UniProtKB/TrEMBL;Acc:Q9FRL0]	28219225	28220372	36.67
	AT1G75200	TYW1	S-adenosyl-L-methionine-dependent tRNA 4-demethylwyosine synthase [Source:UniProtKB/Swiss-Prot;Acc:Q8RXN5]	28220626	28223785	41.55
	AT1G75210		Cytosolic IMP-GMP specific 5-nucleotidase, putative [Source:UniProtKB/TrEMBL;Acc:Q8RWN4]	28223836	28229082	37.13
	AT1G75220		Sugar transporter ERD6-like 6 [Source:UniProtKB/Swiss-Prot;Acc:Q9FRL3]	28229026	28233134	36.7
	AT1G75230		DNA glycosylase superfamily protein [Source:UniProtKB/TrEMBL;Acc:F4HXH4]	28234086	28237290	40.59
	AT1G75240	ZHD5	ZHD5 [Source:UniProtKB/TrEMBL;Acc:A0A178WFZ4]	28241011	28242795	40.5
	AT1G75250	ATRL6	RAD-like 6 [Source:TAIR;Acc:AT1G75250]	28244317	28245581	31.94
	AT1G75260		Oxidoreductases, acting on NADH or NADPH [Source:UniProtKB/TrEMBL;Acc:Q0WVV5]	28247641	28249485	43.36
	AT1G75270	DHAR2	Glutathione S-transferase DHAR2 [Source:UniProtKB/Swiss-Prot;Acc:Q9FRL8]	28250075	28251469	38.64
	AT1G75280		Isoflavone reductase homolog P3 [Source:UniProtKB/Swiss-Prot;Acc:P52577]	28251626	28253686	38.33

AT1G75290		NAD(P)-binding Rossmann-fold superfamily protein [Source:TAIR;Acc:AT1G75290]	28253722	28255173	39.81
AT1G75295		other RNA [Source:TAIR;Acc:AT1G75295]	28254820	28257236	38.44
AT1G75300		At1g75300 [Source:UniProtKB/TrEMBL;Acc:Q9FRM1]	28255495	28257040	38.42
AT1G75310	AUL1	auxin-like 1 protein [Source:TAIR;Acc:AT1G75310]	28260801	28266323	40.97
AT1G75330	OTC	OTC [Source:UniProtKB/TrEMBL;Acc:A0A178W4A4]	28266259	28268416	39.9
AT1G75335		Nucleolar-like protein [Source:UniProtKB/TrEMBL;Acc:Q1G3X0]	28268541	28269002	40.48
AT1G75340	CG1	Zinc finger CCCH domain-containing protein 16 [Source:UniProtKB/Swiss-Prot;Acc:Q9FWS3]	28268968	28272081	38.7
AT1G75350	RPL31	50S ribosomal protein L31, chloroplastic [Source:UniProtKB/Swiss-Prot;Acc:Q9FWS4]	28272071	28273009	39.08
AT1G75360		Transmembrane protein [Source:UniProtKB/TrEMBL;Acc:Q5XVF5]	28274588	28276589	38.51
AT1G75370		Sec14p-like phosphatidylinositol transfer family protein [Source:UniProtKB/TrEMBL;Acc:F4HZ25]	28276281	28280033	37.6
AT1G75380	BBD1	Bifunctional nuclease 1 [Source:UniProtKB/Swiss-Prot;Acc:Q9FWS6]	28281195	28284168	36.11
AT1G09547			28288909	28289117	37.32
AT1G75390	BZIP44	bZIP transcription factor 44 [Source:UniProtKB/Swiss-Prot;Acc:C0Z2L5]	28291698	28293008	37.22
AT1G75400		RING/U-box superfamily protein [Source:UniProtKB/TrEMBL;Acc:Q8L7K7]	28296886	28299768	41.83
AT1G75410	BLH3	BEL1-like homeodomain protein 3 [Source:UniProtKB/Swiss-Prot;Acc:Q9FWS9]	28299786	28302728	36.53
AT1G75420		UDP-Glycosyltransferase superfamily protein [Source:UniProtKB/TrEMBL;Acc:Q7Y217]	28305218	28307548	39.47
AT1G75430	BLH11	Uncharacterized protein At1g75430 (Fragment) [Source:UniProtKB/TrEMBL;Acc:C0SV32]	28307893	28309610	35.62
AT1G75440	UBC16	Probable ubiquitin-conjugating enzyme E2 16 [Source:UniProtKB/Swiss-Prot;Acc:Q9FWT2]	28313379	28315122	37.21
AT1G09553			28314311	28314700	35.38
AT1G75450	CKX5	Cytokinin dehydrogenase 5 [Source:UniProtKB/Swiss-Prot;Acc:Q67YU0]	28314458	28318375	39.84
AT1G09557			28318139	28318355	31.34
AT1G75460		ATP-dependent protease La (LON) domain protein [Source:UniProtKB/TrEMBL;Acc:Q9FWT4]	28327698	28329764	39.91
AT1G75470	PUP15	Putative purine permease 15 [Source:UniProtKB/Swiss-Prot;Acc:Q9LQZ0]	28330054	28331300	45.55
AT1G75490	DREB2D	Dehydration-responsive element-binding protein 2D [Source:UniProtKB/Swiss-Prot;Acc:Q9LQZ2]	28335502	28336990	39.69
AT1G75500	WAT1	Protein WALLS ARE THIN 1 [Source:UniProtKB/Swiss-Prot;Acc:Q94AP3]	28337726	28340275	38.12
AT1G09563			28343829	28344031	44.83
AT1G09567			28343829	28344031	44.83
AT1G75510		At1g75700 [Source:UniProtKB/TrEMBL;Acc:Q9LQZ4]	28346998	28348776	38.28
AT1G75520	SRS5	Protein SHI RELATED SEQUENCE 5 [Source:UniProtKB/Swiss-Prot;Acc:Q9LQZ5]	28351522	28353549	38.91
AT1G75530		Forkhead-associated (FHA) domain-containing protein [Source:UniProtKB/TrEMBL;Acc:F4HZ48]	28359309	28361815	36.78
AT1G75540	BBX21	B-box zinc finger protein 21 [Source:UniProtKB/Swiss-Prot;Acc:Q9LQZ7]	28365824	28367738	36.5
AT1G75550		glycine-rich protein [Source:TAIR;Acc:AT1G75550]	28368447	28370061	36.47
AT1G75560		Putative DNA-binding protein [Source:UniProtKB/TrEMBL;Acc:Q8GXC5]	28371141	28373301	41.51
AT1G75570		pre-tRNA [Source:TAIR;Acc:AT1G75570]	28373489	28373561	61.64
AT1G75580		At1g75580 [Source:UniProtKB/TrEMBL;Acc:Q9LR00]	28377377	28378310	38.33
AT1G75590		SAUR-like auxin-responsive protein family [Source:UniProtKB/TrEMBL;Acc:F4HZ54]	28382813	28383769	43.47
AT1G75600		Histone H3-like 3 [Source:UniProtKB/Swiss-Prot;Acc:Q9LR02]	28390665	28391585	38.76
AT1G75620		F10A5.18 [Source:UniProtKB/TrEMBL;Acc:Q9LR03]	28394763	28396677	43.66
AT1G75630	AVA-P4	V-type proton ATPase proteolipid subunit [Source:UniProtKB/TrEMBL;Acc:F4HZ57]	28400439	28402355	38.29
AT1G75640		F10A5.16 [Source:UniProtKB/TrEMBL;Acc:Q9LR04]	28403371	28407182	45.23
AT1G75650		pre-tRNA [Source:TAIR;Acc:AT1G75650]	28407984	28408056	61.64
AT1G75660	XRN3	5'-3' exoribonuclease 3 [Source:UniProtKB/Swiss-Prot;Acc:Q9FQ03]	28408134	28415118	38.31
AT1G75670		DNA-directed RNA polymerase [Source:UniProtKB/TrEMBL;Acc:Q8H1F8]	28415152	28416961	35.36
AT1G75680	AtGH9B7	Endoglucanase 10 [Source:UniProtKB/Swiss-Prot;Acc:Q8LCP6]	28417052	28419635	39.98
AT1G09573			28420323	28420635	41.21
AT1G75690	LQY1	LQY1 [Source:UniProtKB/TrEMBL;Acc:A0A178W9N8]	28421730	28423520	39.98
AT1G75700	HVA22G	HVA22-like protein G [Source:TAIR;Acc:AT1G75700]	28423495	28425112	33.87
AT1G75710		C2H2-like zinc finger protein [Source:UniProtKB/TrEMBL;Acc:Q9LR10]	28428671	28431339	40.76
AT1G75717		F10A5.9 [Source:UniProtKB/TrEMBL;Acc:Q9LR11]	28433919	28434261	42.57
AT1G75720		Plant protein of unknown function (DUF827) [Source:TAIR;Acc:AT1G75720]	28434524	28435489	36.13
AT1G75730		At1g75730 [Source:UniProtKB/TrEMBL;Acc:A4FVS5]	28435625	28439835	36
AT1G75740		pre-tRNA [Source:TAIR;Acc:AT1G75740]	28440889	28440960	61.11
AT1G75750	GASA1	GASA1 [Source:UniProtKB/TrEMBL;Acc:A0A178W4S5]	28441501	28442435	37.86
AT1G75760		At1g75760 [Source:UniProtKB/TrEMBL;Acc:Q84TL5]	28446632	28448735	36.88
AT1G75770		At1g75770/F10A5_10 [Source:UniProtKB/TrEMBL;Acc:Q94A92]	28449905	28451148	36.09
AT1G75780	TUBB1	Tubulin beta-1 chain [Source:UniProtKB/Swiss-Prot;Acc:P12411]	28451138	28453820	40.14
AT1G75785		Protein TUB21 [Source:UniProtKB/TrEMBL;Acc:A0A1P8AQV4]	28451154	28451792	38.65
AT1G75790	sks18	Multi-copper oxidase type I family protein [Source:UniProtKB/TrEMBL;Acc:Q1PFD0]	28454781	28457528	36.79
AT1G75800		At1g75800/T4O12_2 [Source:UniProtKB/TrEMBL;Acc:Q9LQT4]	28458691	28460961	40.95
AT1G75810		At1g75810 [Source:UniProtKB/TrEMBL;Acc:Q9LQT3]	28461444	28462366	43.01
AT1G75820	CLV1	FLO5 [Source:UniProtKB/TrEMBL;Acc:A0A384L048]	28463344	28466971	41.95
AT1G09577			28467241	28467900	41.21
AT1G09583			28470024	28470292	40.52
AT1G75830	PDF1.1	Defensin-like protein 13 [Source:UniProtKB/Swiss-Prot;Acc:P30224]	28472357	28472898	36.72
AT1G75840	ARAC5	Rac-like GTP-binding protein ARAC5 [Source:UniProtKB/Swiss-Prot;Acc:Q38937]	28475649	28477802	35.89
AT1G75850	VPS35B	Vacuolar protein sorting-associated protein 35 [Source:UniProtKB/TrEMBL;Acc:A0A178WDK6]	28477768	28483946	38.52
AT1G75860		unknown protein; BEST Arabidopsis thaliana protein match is: unknown protein (TAIR:AT1G20100.1); Ha. [Source:TAIR;A	28484129	28486077	38.02
AT1G75870		T4O12.11 [Source:UniProtKB/TrEMBL;Acc:Q9LQS7]	28486229	28487443	37.7
AT1G09587			28487910	28488251	39.77
AT1G75880	EXL1	GDSL esterase/lipase EXL1 [Source:UniProtKB/Swiss-Prot;Acc:Q94CH8]	28490443	28492578	34.36
AT1G75890		GDSL-like Lipase/Acylhydrolase superfamily protein [Source:TAIR;Acc:AT1G75890]	28492944	28495246	32.91
AT1G75891		other RNA [Source:TAIR;Acc:AT1G75891]	28492958	28494327	33.65
AT1G09593			28495290	28495666	40.05
AT1G09597			28495429	28495666	40.34
AT1G75900	EXL3	GDSL esterase/lipase EXL3 [Source:UniProtKB/Swiss-Prot;Acc:Q94CH6]	28498821	28501254	33.77
AT1G75910	EXL4	GDSL esterase/lipase EXL4 [Source:UniProtKB/Swiss-Prot;Acc:Q0WUV7]	28501434	28503283	32.81
AT1G09603			28503779	28504403	36.64
AT1G75920		GDSL-like Lipase/Acylhydrolase superfamily protein [Source:UniProtKB/TrEMBL;Acc:F4I0R0]	28504041	28507168	32.32
AT1G75930	EXL6	GDSL esterase/lipase EXL6 [Source:UniProtKB/Swiss-Prot;Acc:Q93X94]	28508072	28509881	33.31
AT1G75940	BGLU20	Beta-glucosidase 20 [Source:UniProtKB/Swiss-Prot;Acc:Q84WV2]	28511142	28514284	33.98
AT1G75945			28515245	28515916	34.97
AT1G75950	SKP1A	SKP1-like protein 1A [Source:UniProtKB/Swiss-Prot;Acc:Q39255]	28516519	28517896	39.11
AT1G75960	AAE8	Probable acyl-activating enzyme 8 [Source:UniProtKB/Swiss-Prot;Acc:Q9LQS1]	28517939	28519938	46.45
AT1G75970		pre-tRNA [Source:TAIR;Acc:AT1G75970]	28519985	28520056	56.94
AT1G75980		At1g75980 [Source:UniProtKB/TrEMBL;Acc:A0JQ11]	28522554	28524418	36.68
AT1G75990	RPN3B	26S proteasome non-ATPase regulatory subunit 3 homolog B [Source:UniProtKB/Swiss-Prot;Acc:Q9LQR8]	28524404	28526848	39.92
AT1G76000		pre-tRNA [Source:TAIR;Acc:AT1G76000]	28527767	28527838	61.11
AT1G76010		Alba DNA/RNA-binding protein [Source:UniProtKB/TrEMBL;Acc:Q93VA8]	28528059	28531771	37.38
AT1G76020		At1g76020 [Source:UniProtKB/TrEMBL;Acc:A0JQ12]	28532045	28533694	39.94
AT1G76030	VHA-B1	V-type proton ATPase subunit B1 [Source:UniProtKB/Swiss-Prot;Acc:P11574]	28533710	28537194	37.99
AT1G76040	CPK29	calcium-dependent protein kinase 29 [Source:TAIR;Acc:AT1G76040]	28537593	28540707	36.24
AT1G76050		RNA pseudouridine synthase 2, chloroplastic [Source:UniProtKB/Swiss-Prot;Acc:Q3ECD0]	28540784	28543227	40.02
AT1G76060	EMB1793	At1g76060 [Source:UniProtKB/TrEMBL;Acc:Q63Z96]	28543100	28543850	45.67
AT1G76065		LYR family of Fe/S cluster biogenesis protein [Source:UniProtKB/TrEMBL;Acc:Q9LQR2]	28544338	28545708	35.08
AT1G76062	MIR835A	MIR835a; miRNA [Source:TAIR;Acc:AT1G76062]	28544454	28544853	35.25
AT1G76070		Uncharacterized protein At1g76070 [Source:UniProtKB/Swiss-Prot;Acc:Q9SGS5]	28546382	28547611	41.06
AT1G76080	CDSP32	CDSP32 [Source:UniProtKB/TrEMBL;Acc:A0A178WHK1]	28547786	28549586	39.14
AT1G76090	SMT3	24-methylenesterol C-methyltransferase 3 [Source:UniProtKB/Swiss-Prot;Acc:Q94JS4]	28550202	28551836	42.2

AT1G76100	PETE1	plastocyanin 1 [Source:TAIR;Acc:AT1G76100]	28553883	28554930	43.32
AT1G76110	HMGB9	High mobility group B protein 9 [Source:UniProtKB/Swiss-Prot;Acc:Q9SGS2]	28555034	28557615	34.93
AT1G76120		tRNA pseudouridine synthase [Source:UniProtKB/TrEMBL;Acc:Q9SGS1]	28558630	28560473	39.64
AT1G76130	AMY2	Probable alpha-amylase 2 [Source:UniProtKB/Swiss-Prot;Acc:Q8LFG1]	28560629	28564159	36.96
AT1G09607			28568368	28568677	37.1
AT1G76135	MIR394B	MIR394B; miRNA [Source:TAIR;Acc:AT1G76135]	28568808	28568928	42.15
AT1G76140		Prolyl oligopeptidase family protein [Source:UniProtKB/TrEMBL;Acc:F4I2A0]	28571119	28575036	40.61
AT1G76150	ECH2	Enoyl-CoA hydratase 2, peroxisomal [Source:UniProtKB/Swiss-Prot;Acc:Q8VYI3]	28574740	28577700	37.49
AT1G76160	sks5	Sks5 [Source:UniProtKB/TrEMBL;Acc:A0A178WJJ9]	28577842	28581315	39.87
AT1G76170		2-thiocytidine tRNA biosynthesis protein, TtcA [Source:TAIR;Acc:AT1G76170]	28583850	28586418	38.03
AT1G76180	ERD14	Dehydrin ERD14 [Source:UniProtKB/Swiss-Prot;Acc:P42763]	28586663	28587931	37.67
AT1G76185		At1g76185 [Source:UniProtKB/TrEMBL;Acc:Q8LCY3]	28590325	28591531	38.61
AT1G76190		SAUR-like auxin-responsive protein family [Source:UniProtKB/TrEMBL;Acc:Q9SGR4]	28592059	28592820	37.66
AT1G76200		NADH dehydrogenase [ubiquinone] 1 beta subcomplex subunit 2 [Source:UniProtKB/Swiss-Prot;Acc:Q8LDK3]	28593211	28594750	36.95
AT1G76210		At1g76210 [Source:UniProtKB/TrEMBL;Acc:Q9SGR3]	28594950	28595996	37.73
AT1G76220		T23E18.15 [Source:UniProtKB/TrEMBL;Acc:Q9SGR2]	28597376	28598420	38.85
AT1G76230		At1g76230 [Source:UniProtKB/TrEMBL;Acc:Q9SGR1]	28599678	28600491	36.12
AT1G09613			28600507	28600725	41.55
AT1G76240		At1g76240 [Source:UniProtKB/TrEMBL;Acc:Q501A3]	28602705	28603932	41.86
AT1G76250		At1g76250 [Source:UniProtKB/TrEMBL;Acc:Q8GX25]	28606444	28609225	40.4
AT1G76260	DWA2	WD repeat-containing protein DWA2 [Source:UniProtKB/Swiss-Prot;Acc:Q6NPN9]	28610193	28613263	37.35
AT1G76270	OFUT16	O-fucosyltransferase 16 [Source:UniProtKB/Swiss-Prot;Acc:Q949U4]	28613207	28616785	38.84
AT1G76280		Tetratricopeptide repeat (TPR)-like superfamily protein [Source:TAIR;Acc:AT1G76280]	28617577	28622777	37.67
AT1G76290	AEE21	Probable acyl-activating enzyme 21 [Source:UniProtKB/Swiss-Prot;Acc:Q9SFW5]	28623398	28625408	43.21
AT1G76300	SMD3A	Small nuclear ribonucleoprotein SmD3a [Source:UniProtKB/Swiss-Prot;Acc:Q9S7E6]	28625871	28627408	38.23
AT1G76310	CYCB2;4	CYCLIN B2;4 [Source:TAIR;Acc:AT1G76310]	28627694	28630789	36.27
AT1G76320	FRS4	Protein FAR1-RELATED SEQUENCE 4 [Source:UniProtKB/Swiss-Prot;Acc:Q6NQJ7]	28631223	28634588	40.14
AT1G76330		pre-tRNA [Source:TAIR;Acc:AT1G76330]	28634692	28634773	51.22
AT1G76340	GONST3	GDP-mannose transporter GONST3 [Source:UniProtKB/Swiss-Prot;Acc:Q9S845]	28634881	28636968	39.51
AT1G76350	NLP5	Protein NLP5 [Source:UniProtKB/Swiss-Prot;Acc:Q9SFW8]	28639453	28643086	38.88
AT1G09617			28639806	28640505	36.29
AT1G76360		At1g76360 [Source:UniProtKB/TrEMBL;Acc:A4FVS9]	28643038	28646721	36.59
AT1G09623			28645595	28645885	48.11
AT1G76370	PBL22	Probable serine/threonine-protein kinase PBL22 [Source:UniProtKB/Swiss-Prot;Acc:Q9SFX0]	28648327	28650577	37.67
AT1G76380		At1g76380 [Source:UniProtKB/TrEMBL;Acc:A4FVS4]	28651069	28654660	37.33
AT1G09627			28655208	28658086	39.46
AT1G76390	PUB43	U-box domain-containing protein 43 [Source:UniProtKB/Swiss-Prot;Acc:Q9SFX2]	28655208	28658711	39.24
AT1G76400	OST1A	Dolichyl-diphosphooligosaccharide--protein glycosyltransferase subunit 1A [Source:UniProtKB/Swiss-Prot;Acc:Q9SFX3]	28658554	28661710	39.28
AT1G76405	OEP21B	Outer envelope pore protein 21B, chloroplastic [Source:UniProtKB/Swiss-Prot;Acc:Q9FPG2]	28661789	28663826	37.49
AT1G76410	ATL8	ATL8 [Source:UniProtKB/TrEMBL;Acc:A0A178WF21]	28668664	28669652	42.37
AT1G76420	NAC031	Protein CUP-SHAPED COTYLEDON 3 [Source:UniProtKB/Swiss-Prot;Acc:Q9S851]	28671806	28674045	37.77
AT1G76430	PHT1-9	Putative phosphate transporter [Source:UniProtKB/TrEMBL;Acc:Q0WRM8]	28678935	28682062	38.59
AT1G76440		AT1G76440 protein [Source:UniProtKB/TrEMBL;Acc:Q9S842]	28682084	28683785	38.54
AT1G76450	PPD3	PsbP domain-containing protein 3, chloroplastic [Source:UniProtKB/Swiss-Prot;Acc:Q9S720]	28684551	28686244	37.84
AT1G76460		RNA-binding (RRM/RBD/RNP motifs) family protein [Source:UniProtKB/TrEMBL;Acc:F4I2E4]	28686181	28689208	39.4
AT1G76465			28688781	28689163	35.51
AT1G76470		NAD(P)-binding Rossmann-fold superfamily protein [Source:UniProtKB/TrEMBL;Acc:F4I2E5]	28689766	28691659	37.33
AT1G76480		unknown protein; BEST Arabidopsis thaliana protein match is: unknown protein (TAIR:AT1G20890.1); Ha. [Source:TAIR;/	28692070	28693428	36.35
AT1G76490	HMG1	hydroxy methylglutaryl CoA reductase 1 [Source:TAIR;Acc:AT1G76490]	28695760	28698852	42.29
AT1G76500	AHL29	AT-hook motif nuclear-localized protein 29 [Source:UniProtKB/Swiss-Prot;Acc:Q9C9K7]	28704963	28706691	45.23
AT1G76510		ARID/BRIGHT DNA-binding domain-containing protein [Source:UniProtKB/TrEMBL;Acc:F4I2F0]	28708339	28712968	38.36
AT1G76520	PILS3	Protein PIN-LIKES 3 [Source:UniProtKB/Swiss-Prot;Acc:Q9C9K5]	28714765	28717910	37.54
AT1G76530	PILS4	Protein PIN-LIKES 4 [Source:UniProtKB/Swiss-Prot;Acc:Q9C9K4]	28718069	28720371	37.99
AT1G76540	CDKB2-1	CDKB2 [Source:UniProtKB/TrEMBL;Acc:A0A178WJF2]	28720328	28722550	39.99
AT1G76550	PFP-ALPHA2	Pyrophosphate--fructose 6-phosphate 1-phosphotransferase subunit alpha 2 [Source:UniProtKB/Swiss-Prot;Acc:Q9C9K3]	28722657	28727111	38.27
AT1G76560	CP12-3	CP12-3 [Source:UniProtKB/TrEMBL;Acc:A0A178WDK7]	28728206	28728976	43.97
AT1G76570	LHCB7	Chlorophyll a-b binding protein 7, chloroplastic [Source:UniProtKB/Swiss-Prot;Acc:Q9C9K1]	28728998	28730851	39.64
AT1G76580		Squamosa promoter-binding protein-like (SBP domain) transcription factor family protein [Source:TAIR;Acc:AT1G76580]	28734207	28738967	39.74
AT1G09633			28739770	28740168	42.11
AT1G76590		At1g76590 [Source:UniProtKB/TrEMBL;Acc:Q2HIW3]	28740540	28742231	37.06
AT1G76600		Poly polymerase [Source:UniProtKB/TrEMBL;Acc:Q9C9J8]	28746833	28747848	39.76
AT1G76610		Uncharacterized protein F14G6.21 [Source:UniProtKB/TrEMBL;Acc:Q9C9J7]	28750872	28751875	42.33
AT1G76620		At1g76620/F14G6_22 [Source:UniProtKB/TrEMBL;Acc:Q8W4R7]	28756521	28759332	34.85
AT1G76630	SKI3	Tetratricopeptide repeat protein SKI3 [Source:UniProtKB/Swiss-Prot;Acc:F4I3Z5]	28759478	28765042	39.5
AT1G76640	CML39	Calcium-binding protein CML39 [Source:UniProtKB/Swiss-Prot;Acc:Q9SRE7]	28765254	28765866	37.85
AT1G76650	CML38	CML38 [Source:UniProtKB/TrEMBL;Acc:A0A178WMC5]	28766750	28768138	34.34
AT1G76660		Uncharacterized protein At1g76660 [Source:UniProtKB/Swiss-Prot;Acc:Q9SRE5]	28768759	28771538	38.2
AT1G76670	URGT1	UDP-rhamnose/UDP-galactose transporter 1 [Source:UniProtKB/Swiss-Prot;Acc:Q9SRE4]	28772563	28775002	38.77
AT1G76680	OPR1	12-oxophytodienoate reductase 1 [Source:UniProtKB/TrEMBL;Acc:F4I403]	28776626	28778837	40.87
AT1G76690	OPR2	OPR2 [Source:UniProtKB/TrEMBL;Acc:A0A178W9S3]	28778622	28780483	41.46
AT1G76700	ATJ10	Chaperone protein dnaJ 10 [Source:UniProtKB/Swiss-Prot;Acc:Q8GYX8]	28780408	28783257	38.04
AT1G09637			28782714	28782958	37.96
AT1G09647			28785165	28785372	47.6
AT1G76705		Calmodulin binding protein [Source:UniProtKB/TrEMBL;Acc:F4I407]	28786290	28787328	39.08
AT1G76710	ASHH1	SET domain group 26 [Source:TAIR;Acc:AT1G76710]	28789698	28792719	38.39
AT1G76720		Eukaryotic translation initiation factor 2 (eIF-2) family protein [Source:UniProtKB/TrEMBL;Acc:Q9SRE1]	28794144	28800402	38.87
AT1G09653			28801490	28801798	38.19
AT1G76728		unknown protein; LOCATED IN: endomembrane system; Ha. [Source:TAIR;Acc:AT1G76728]	28802633	28802734	47.06
AT1G76730	COG0212	COG0212 [Source:UniProtKB/TrEMBL;Acc:A0A178WEK7]	28802753	28804858	39.22
AT1G76740		Uncharacterized protein F28O16.11 [Source:UniProtKB/TrEMBL;Acc:Q9SRD9]	28805114	28810647	39.83
AT1G76750	EC1.1	Egg cell-secreted protein 1.1 [Source:UniProtKB/Swiss-Prot;Acc:Q9SRD8]	28811040	28811721	43.26
AT1G09657			28811060	28811379	47.81
AT1G76760	ATY1	TY1 [Source:UniProtKB/TrEMBL;Acc:A0A178WE50]	28811685	28813164	36.69
AT1G09663			28812404	28812786	33.42
AT1G09667			28813116	28813465	40.57
AT1G76770		HSP20-like chaperones superfamily protein [Source:UniProtKB/TrEMBL;Acc:Q9SRD6]	28813454	28814378	36.54
AT1G76780		HSP20-like chaperones superfamily protein [Source:TAIR;Acc:AT1G76780]	28814434	28822256	36.88
AT1G09673			28816622	28817970	38.25
AT1G76790	IGMT5	Indole glucosinolate O-methyltransferase 5 [Source:UniProtKB/Swiss-Prot;Acc:Q9SRD4]	28822186	28823727	38.85
AT1G76800		Vacuolar iron transporter homolog 2 [Source:UniProtKB/Swiss-Prot;Acc:Q9SRD3]	28829243	28830278	41.51
AT1G76810		Eukaryotic translation initiation factor 2 (eIF-2) family protein [Source:UniProtKB/TrEMBL;Acc:F4I420]	28830902	28836748	41.06

MQTLs	Gene stable ID	Gene name	Gene description	Gene start (bp)	Gene end (bp)	Gene % GC content
MQTL-1/Chr2	AT2G05685			4963770	4964218	37.42
	AT2G12400		Plasma membrane fusion protein [Source:UniProtKB/TrEMBL;Acc:Q8H0U2]	5004884	5008442	36.98
	AT2G05695			5009533	5009872	45.59
	AT2G12405		unknown protein; FUNCTIONS IN: molecular_function unknown; INVOLVED IN: biological_process unknown; LOCATED IN: en	5010792	5011590	36.92
	AT2G05705			5021920	5022199	41.79
	AT2G05725			5030903	5031230	36.28
	AT2G05785			5040157	5040392	39.41
	AT2G12461		unknown protein; Ha. [Source:TAIR;Acc:AT2G12461]	5049953	5050087	50.37
	AT2G12462		Sterile alpha motif (SAM) domain protein [Source:UniProtKB/TrEMBL;Acc:Q1G3Q5]	5050434	5051613	39.32
	AT2G12465	LCR50	Putative defensin-like protein 111 [Source:UniProtKB/Swiss-Prot;Acc:P82765]	5055415	5055880	30.26
	AT2G12475		Defensin-like protein 112 [Source:UniProtKB/Swiss-Prot;Acc:Q2V488]	5068402	5068918	33.08
	AT2G12480	SCPL43	serine carboxypeptidase-like 43 [Source:TAIR;Acc:AT2G12480]	5069544	5072656	37.97
	AT2G05815			5080634	5080714	48.15
	AT2G05825			5081722	5082662	42.08
	AT2G05835			5082130	5082518	47.3
	AT2G12550		At2g12550 [Source:UniProtKB/TrEMBL;Acc:Q500V3]	5114804	5118753	38.05
	AT2G12646		PLATZ transcription factor family protein [Source:UniProtKB/TrEMBL;Acc:Q1G3Q4]	5166984	5169303	35.09
	AT2G12875		unknown protein; Ha. [Source:TAIR;Acc:AT2G12875]	5289411	5291470	42.67
	AT2G12880		Putative CCHC-type zinc finger protein [Source:UniProtKB/TrEMBL;Acc:Q9SKG2]	5291523	5291882	41.67
	AT2G12900		BZIP transcription factor family protein [Source:UniProtKB/TrEMBL;Acc:Q9SKG1]	5293897	5295480	34.47
MQTL-2/Chr2	AT2G20110		Tesmin/TSO1-like CXC domain-containing protein [Source:TAIR;Acc:AT2G20110]	8683979	8687283	39.49
	AT2G20120	COV1	Protein of unknown function (DUF502) [Source:TAIR;Acc:AT2G20120]	8687330	8690214	34.56
	AT2G20130	LCV1	Protein LIKE COV 1 [Source:UniProtKB/Swiss-Prot;Acc:Q8VY49]	8690293	8692490	37.4
	AT2G20140	RPT2B	RPT2b [Source:UniProtKB/TrEMBL;Acc:A0A178VM16]	8692567	8695111	39.49
	AT2G20142		Toll-Interleukin-Resistance (TIR) domain family protein [Source:UniProtKB/TrEMBL;Acc:F4IUF0]	8695374	8696643	37.8
	AT2G20150		Uncharacterized protein At2g20150/T2G17.5 [Source:UniProtKB/TrEMBL;Acc:Q84X45]	8697300	8699299	38.4
	AT2G20160	ASK17	SKP1-like protein 17 [Source:UniProtKB/Swiss-Prot;Acc:Q9SL65]	8699511	8700186	39.64
	AT2G20170		NEP-interacting protein, putative (DUF239) [Source:UniProtKB/TrEMBL;Acc:Q9SL64]	8701013	8704357	32.83
	AT2G20180	PIF1	Transcription factor PIF1 [Source:UniProtKB/Swiss-Prot;Acc:Q8GZM7]	8704024	8707009	40.86
	AT2G20190	CLASP	CLIP-associated protein [Source:UniProtKB/Swiss-Prot;Acc:Q8RWY6]	8711579	8719115	40.2
	AT2G20208	LCR60	Defensin-like protein 172 [Source:UniProtKB/Swiss-Prot;Acc:P82774]	8720790	8721423	35.33
	AT2G20210		RNI-like superfamily protein [Source:UniProtKB/TrEMBL;Acc:F4IUG1]	8721325	8725127	38.5
	AT2G20230	TOM2AH2	Tetraspanin-18 [Source:UniProtKB/Swiss-Prot;Acc:Q93XY5]	8725541	8727742	37.87
	AT2G07265			8726067	8726447	36.22
	AT2G20240		GPI-anchored adhesin-like protein, putative (DUF3741) [Source:UniProtKB/TrEMBL;Acc:Q9SK77]	8727626	8730525	40.97
	AT2G07275			8728701	8729253	43.76
	AT2G07285			8732793	8732997	30.24
	AT2G20250		unknown protein; Ha. [Source:TAIR;Acc:AT2G20250]	8733285	8736597	38.48
	AT2G20260	PSAE2	Photosystem I reaction center subunit IV B, chloroplastic [Source:UniProtKB/Swiss-Prot;Acc:Q9S714]	8736734	8737767	40.81
	AT2G20270		Thioredoxin superfamily protein [Source:UniProtKB/TrEMBL;Acc:F4IUG7]	8737705	8739677	37.2
	AT2G20280		Zinc finger CCCH domain-containing protein 21 [Source:UniProtKB/Swiss-Prot;Acc:Q9SK74]	8739740	8742650	38.27
	AT2G20290	XI-G	Myosin-13 [Source:UniProtKB/Swiss-Prot;Acc:F4IUG9]	8742885	8752315	37.16
	AT2G20300	ALE2	Receptor-like serine/threonine-protein kinase ALE2 [Source:UniProtKB/Swiss-Prot;Acc:Q8RWW0]	8755852	8760298	41.74
	AT2G20310	RIN13	At2g20310 [Source:UniProtKB/TrEMBL;Acc:Q9SK71]	8761180	8763372	41.18
	AT2G20320		DENN (AEX-3) domain-containing protein [Source:UniProtKB/TrEMBL;Acc:F4IUH2]	8766486	8772668	38.65
	AT2G20330		At2g20330/F11A3.12 [Source:UniProtKB/TrEMBL;Acc:Q9SK69]	8772653	8775668	43.1
	AT2G20340	ELI5	Tyrosine decarboxylase 1 [Source:UniProtKB/Swiss-Prot;Acc:Q8RY79]	8779667	8782789	37.37
	AT2G20350	ERF120	Ethylene-responsive transcription factor ERF120 [Source:UniProtKB/Swiss-Prot;Acc:Q9SK67]	8784769	8785447	44.04
	AT2G20360		NADH dehydrogenase [ubiquinone] 1 alpha subcomplex subunit 9, mitochondrial [Source:UniProtKB/Swiss-Prot;Acc:Q9SK66]	8785960	8789570	38.41
	AT2G20362		Transmembrane protein [Source:UniProtKB/TrEMBL;Acc:Q1G3C1]	8789594	8790742	38.21
	AT2G20370	MUR3	Xyloglucan galactosyltransferase MUR3 [Source:UniProtKB/Swiss-Prot;Acc:Q7XJ98]	8791939	8794401	43.97
	AT2G20380		F-box protein At2g20380 [Source:UniProtKB/Swiss-Prot;Acc:Q9SK64]	8794818	8795980	43.85
	AT2G20390		Cytochrome oxidase complex assembly protein [Source:UniProtKB/TrEMBL;Acc:Q9SK63]	8796185	8798293	38.5
	AT2G20400		myb-like HTH transcriptional regulator family protein [Source:TAIR;Acc:AT2G20400]	8799041	8801991	38.8
	AT2G20410		Expressed protein [Source:UniProtKB/TrEMBL;Acc:Q9SK61]	8802091	8804422	37.78
	AT2G20420		Succinate--CoA ligase [ADP-forming] subunit beta, mitochondrial [Source:UniProtKB/Swiss-Prot;Acc:O82662]	8805342	8808171	39.08
	AT2G20430	RIC6	CRIB domain-containing protein RIC6 [Source:UniProtKB/Swiss-Prot;Acc:Q1PF35]	8808414	8810200	34.53
	AT2G07295			8809170	8809364	30.26
	AT2G20440		Ypt/Rab-GAP domain of gyp1p superfamily protein [Source:UniProtKB/TrEMBL;Acc:F4IVD6]	8810644	8813696	37.08
	AT2G20450	RPL14A	60S ribosomal protein L14-1 [Source:UniProtKB/Swiss-Prot;Acc:Q9SIM4]	8813832	8815379	35.85
	AT2G20463		Putative defensin-like protein 104 [Source:UniProtKB/Swiss-Prot;Acc:Q2V476]	8823669	8824150	33.2
	AT2G20465		Defensin-like protein 103 [Source:UniProtKB/Swiss-Prot;Acc:Q8GXR4]	8825053	8825544	32.11
	AT2G20470		Non-specific serine/threonine protein kinase [Source:UniProtKB/TrEMBL;Acc:Q1PF34]	8826017	8829771	38.3
	AT2G20480		Uncharacterized protein At2g20480 [Source:UniProtKB/TrEMBL;Acc:Q9SIM1]	8830123	8831615	36.03
	AT2G20490	NOP10	H/ACA ribonucleoprotein complex subunit 3-like protein [Source:UniProtKB/Swiss-Prot;Acc:Q93XX8]	8831681	8832929	35.55
	AT2G20495		CONTAINS InterPro DOMAIN/s: Serine-threonine protein kinase 19 (InterPro:IPR018865); Ha. [Source:TAIR;Acc:AT2G20495]	8832957	8835263	36.97
	AT2G07305			8835103	8836069	37.95
	AT2G20500		Uncharacterized protein At2g20500/T13C7.9 [Source:UniProtKB/TrEMBL;Acc:Q9SIL9]	8835120	8836044	37.84
	AT2G20510	ATTIM44-1	translocase inner membrane subunit 44-1 [Source:TAIR;Acc:AT2G20510]	8836018	8838710	39.36
	AT2G20515		At2g20515 [Source:UniProtKB/TrEMBL;Acc:Q8S8D0]	8838750	8839857	42.78
	AT2G20520	FLA6	Fasciclin-like arabinogalactan protein 6 [Source:UniProtKB/Swiss-Prot;Acc:Q9SIL7]	8840557	8841565	40.04
	AT2G20530	PHB6	Prohibitin-6, mitochondrial [Source:UniProtKB/Swiss-Prot;Acc:Q9SIL6]	8841972	8844022	40.76
	AT2G20540	PCMP-E78	Pentatricopeptide repeat-containing protein At2g20540 [Source:UniProtKB/Swiss-Prot;Acc:Q9SIL5]	8844079	8846095	39.07
	AT2G20550		At2g20550 [Source:UniProtKB/TrEMBL;Acc:Q9SIL4]	8845802	8847311	42.91
	AT2G20560		At2g20560/T13C7.15 [Source:UniProtKB/TrEMBL;Acc:Q9SIL3]	8848130	8850111	41.93
	AT2G20562		Putative uncharacterized protein [Source:UniProtKB/TrEMBL;Acc:Q1G309]	8850148	8851035	33.67
	AT2G20570	GPRI1	GBF's pro-rich region-interacting factor 1 [Source:UniProtKB/TrEMBL;Acc:F4IVF9]	8855302	8857758	40.66
	AT2G20580	RPN1A	26S proteasome non-ATPase regulatory subunit 2 homolog [Source:UniProtKB/TrEMBL;Acc:A0A178VZC8]	8858962	8864865	39.13
	AT2G20585	NFD6	Nuclear fusion defective 6 [Source:UniProtKB/TrEMBL;Acc:A0A1P8B0U7]	8865016	8866791	37.95
	AT2G20590	RTNLB17	Reticulon-like protein B17 [Source:UniProtKB/Swiss-Prot;Acc:Q6DR04]	8867040	8869329	39.91
	AT2G20595		Expressed protein [Source:UniProtKB/TrEMBL;Acc:Q8S8D5]	8871518	8872061	32.9
	AT2G20597		Plant thionin family protein [Source:UniProtKB/TrEMBL;Acc:A8MS59]	8873007	8873546	32.41
	AT2G20605		Plant thionin family protein [Source:TAIR;Acc:AT2G20605]	8876436	8876912	34.59
	AT2G20610	SUR1	S-alkyl-thiohydroximate lyase SUR1 [Source:UniProtKB/Swiss-Prot;Acc:Q9SIV0]	8877887	8880440	40.88
	AT2G20613		Probable transcription factor At2g20613 [Source:UniProtKB/Swiss-Prot;Acc:Q8S8D6]	8886037	8886444	42.89
	AT2G20616		Family of unknown function (DUF566) [Source:TAIR;Acc:AT2G20616]	8887116	8888305	39.58
	AT2G20618		Plant thionin family protein [Source:TAIR;Acc:AT2G20618]	8888880	8889303	37.03
	AT2G20619		Plant thionin family protein [Source:UniProtKB/TrEMBL;Acc:A8MS38]	8891191	8891391	39.3
	AT2G20620		UPF0725 protein At2g20620 [Source:UniProtKB/Swiss-Prot;Acc:Q9SIU9]	8891920	8893244	40.75
	AT2G07315			8894932	8895006	42.67
	AT2G20625		UPF0725 protein At2g20625 [Source:UniProtKB/Swiss-Prot;Acc:Q8S8D8]	8896154	8897218	37.93
	AT2G20630	PPC3-1.2	Probable protein phosphatase 2C 20 [Source:UniProtKB/Swiss-Prot;Acc:Q9SIU8]	8896912	8899898	36.46
	AT2G20635	BUB1	Mitotic checkpoint serine/threonine-protein kinase BUB1 [Source:UniProtKB/Swiss-Prot;Acc:F4IVI0]	8900360	8903698	38.63
	AT2G20650	FLY2	FLY2 [Source:UniProtKB/TrEMBL;Acc:A0A178W2C9]	8903343	8907339	38.48
	AT2G20660	RALFL14	Protein RALF-like 14 [Source:UniProtKB/Swiss-Prot;Acc:Q9SIU6]	8909467	8909772	41.5
	AT2G20670		Expressed protein [Source:UniProtKB/TrEMBL;Acc:Q9SIU5]	8911860	8913747	37.61
	AT2G20680	MAN2	Mannan endo-1,4-beta-mannosidase 2 [Source:UniProtKB/Swiss-Prot;Acc:Q7Y223]	8920447	8923227	39.52
	AT2G20690		At2g20690/F5H14.34 [Source:UniProtKB/TrEMBL;Acc:Q9SKU8]	8923305	8924974	42.04
	AT2G20700	LLG2	GPI-anchored protein LLG2 [Source:UniProtKB/Swiss-Prot;Acc:Q6NLF4]	8924894	8925804	38.31
	AT2G20710		Pentatricopeptide repeat-containing protein At2g20710, mitochondrial [Source:UniProtKB/Swiss-Prot;Acc:Q9SKU6]	8925926	8927844	43.98
	AT2G07325			8926399	8927650	44.89
	AT2G20720		At2g20720 [Source:UniProtKB/TrEMBL;Acc:Q8L705]	8927822	8930120	40.89
	AT2G07335			8927868	8929599	43.88
	AT2G20721		snoRNA [Source:TAIR;Acc:AT2G20721]	8931565	8931655	37.36

AT2G20722		snoRNA [Source:TAIR;Acc:AT2G20722]	8931708	8931782	30.67
AT2G20723		snoRNA [Source:TAIR;Acc:AT2G20723]	8931849	8931922	33.78
AT2G20725		CAAX amino terminal protease family protein [Source:UniProtKB/TrEMBL;Acc:Q94K61]	8933317	8935496	42.29
AT2G20740	TOM2AH3	Tetraspanin-19 [Source:UniProtKB/Swiss-Prot;Acc:Q940P5]	8935537	8937554	38.45
AT2G20750	EXPB1	Expansin-B1 [Source:UniProtKB/Swiss-Prot;Acc:Q9SKU2]	8940833	8942706	42.48
AT2G20760		Clathrin light chain 1 [Source:UniProtKB/Swiss-Prot;Acc:Q9SKU1]	8942750	8945244	40.84
AT2G20770	GCL2	LanC-like protein GCL2 [Source:UniProtKB/Swiss-Prot;Acc:Q8VZQ6]	8945243	8947310	42.7
AT2G20780	PLT4	Probable polyol transporter 4 [Source:UniProtKB/Swiss-Prot;Acc:Q0WUU6]	8947242	8949429	47.07
AT2G20790	AP5M	AP-5 complex subunit mu [Source:UniProtKB/Swiss-Prot;Acc:Q8W0Z6]	8949453	8952874	41.79
AT2G20784		unknown protein; Ha. [Source:TAIR;Acc:AT2G20784]	8949615	8949874	33.08
AT2G20800	NDB4	External alternative NAD(P)H-ubiquinone oxidoreductase B4, mitochondrial [Source:UniProtKB/Swiss-Prot;Acc:Q9SKT7]	8953057	8955831	41.87
AT2G20805		DNA-binding storekeeper protein transcriptional regulator-like protein [Source:UniProtKB/TrEMBL;Acc:Q1PF30]	8956169	8956737	33.04
AT2G20810	GAUT10	Hexosyltransferase (Fragment) [Source:UniProtKB/TrEMBL;Acc:W8PV18]	8957793	8959780	42.66
AT2G20815	QWRF3	Family of unknown function (DUF566) [Source:TAIR;Acc:AT2G20815]	8959255	8962149	39.76
AT2G20820		Expressed protein [Source:UniProtKB/TrEMBL;Acc:Q9SKT5]	8964199	8965568	40.29
AT2G20825	ULT2	Protein ULTRAPETALA 2 [Source:UniProtKB/Swiss-Prot;Acc:Q8S8I2]	8965556	8967243	41.88
AT2G20830		Folic acid binding / transferase [Source:UniProtKB/TrEMBL;Acc:F4IFK0]	8968195	8970739	42.12
AT2G20835		At2g20835 [Source:UniProtKB/TrEMBL;Acc:Q8S8I3]	8970886	8971339	41.85
AT2G20840	SCAMP1	Secretory carrier-associated membrane protein 1 [Source:UniProtKB/Swiss-Prot;Acc:Q9SKT3]	8971696	8974555	39.76
AT2G20850	SRF1	Protein STRUBBELIG-RECEPTOR FAMILY 1 [Source:UniProtKB/Swiss-Prot;Acc:Q06BH3]	8975313	8979437	40.53
AT2G20860	LIP1	Lipoyl synthase, mitochondrial [Source:UniProtKB/Swiss-Prot;Acc:Q9ZWT1]	8979554	8981923	42.91
AT2G20870		At2g20870 [Source:UniProtKB/TrEMBL;Acc:A6QRC7]	8981927	8982712	43
AT2G20873			8982123	8982194	41.67
AT2G20875	EPF1	Protein EPIDERMAL PATTERNING FACTOR 1 [Source:UniProtKB/Swiss-Prot;Acc:Q8S8I4]	8982863	8983814	40.65
AT2G20880	ERF053	Ethylene-responsive transcription factor ERF053 [Source:UniProtKB/Swiss-Prot;Acc:Q9SKT1]	8985959	8987302	45.16
AT2G20890	THF1	THF1 [Source:UniProtKB/TrEMBL;Acc:A0A178VVV5]	8987584	8989416	40.26
AT2G07345			8988633	8988845	37.09
AT2G20900	DGK5	Diacylglycerol kinase 5 [Source:UniProtKB/Swiss-Prot;Acc:Q9C5E5]	8989275	8993275	38.94
AT2G07355			8990844	8991189	32.95
AT2G20920		At2g20920/F5H14.11 [Source:UniProtKB/TrEMBL;Acc:Q9SKS8]	8998450	9000073	45.2
AT2G20921		unknown protein; Ha. [Source:TAIR;Acc:AT2G20921]	9000025	9000228	48.53
AT2G20930		AT2G20930 protein [Source:UniProtKB/TrEMBL;Acc:Q9SKS7]	9000450	9001837	39.05
AT2G20940		At2g20940 [Source:UniProtKB/TrEMBL;Acc:Q9SKR7]	9002001	9002908	41.85
AT2G20950		Arabidopsis phospholipase-like protein (PEARLI 4) family [Source:TAIR;Acc:AT2G20950]	9002843	9005944	44
AT2G20960	pEARLI4	PEARLI 4 protein [Source:UniProtKB/TrEMBL;Acc:Q9SKR5]	9006610	9009794	43.58
AT2G20970		unknown protein; Ha. [Source:TAIR;Acc:AT2G20970]	9009867	9011067	43.05
AT2G20980	MCM10	Minichromosome maintenance 10 [Source:UniProtKB/TrEMBL;Acc:Q5XVE2]	9011344	9013963	39.81
AT2G20990	SYTA	Synaptotagmin A [Source:UniProtKB/TrEMBL;Acc:F4IFM6]	9014470	9018099	39.94
AT2G21010		Calcium-dependent lipid-binding (CaLB domain) family protein [Source:UniProtKB/TrEMBL;Acc:F4IFM9]	9020944	9021870	42.39
AT2G21030		CONTAINS InterPro DOMAIN/s: Disease resistance/zinc finger/chromosome condensation-like region (InterPro:IPR013591); BEST	9024536	9025764	38.24
AT2G21040		Calcium-dependent lipid-binding (CaLB domain) family protein [Source:UniProtKB/TrEMBL;Acc:Q9SKQ7]	9026157	9027310	46.53
AT2G21045	HAC1	Protein HIGH ARSENIC CONTENT 1, mitochondrial [Source:UniProtKB/Swiss-Prot;Acc:Q8RUD6]	9027730	9029058	33.86
AT2G21050	LAX2	Auxin transporter-like protein 2 [Source:UniProtKB/Swiss-Prot;Acc:Q9S836]	9034056	9036636	40.64
AT2G21060	CSP4	GRP2B [Source:UniProtKB/TrEMBL;Acc:A0A178VU21]	9036766	9037782	48.87
AT2G21070	FIO1	U6 small nuclear RNA (adenine-(43)-N(6))-methyltransferase [Source:UniProtKB/TrEMBL;Acc:F4IGH3]	9040852	9043651	40.5
AT2G21080		At2g21080 [Source:UniProtKB/TrEMBL;Acc:Q8L726]	9043699	9045389	41.69
AT2G21090	PCMP-E48	Pentatricopeptide repeat-containing protein At2g21090 [Source:UniProtKB/Swiss-Prot;Acc:Q9SKQ4]	9045440	9047646	43.91
AT2G21100	DIR23	Dirigent protein 23 [Source:UniProtKB/Swiss-Prot;Acc:Q84TH6]	9048131	9049404	41.37
AT2G21105			9048192	9048203	25
AT2G21110	DIR4	Dirigent protein 4 [Source:UniProtKB/Swiss-Prot;Acc:Q9SKQ2]	9050220	9050868	44.38
AT2G21120		Probable magnesium transporter [Source:UniProtKB/TrEMBL;Acc:A0A178W254]	9051725	9054821	37.58
AT2G21130	CYP19-2	Peptidyl-prolyl cis-trans isomerase CYP19-2 [Source:UniProtKB/Swiss-Prot;Acc:Q9SKQ0]	9055398	9056481	41.61
AT2G21140	PRP2	Proline-rich protein 2 [Source:UniProtKB/Swiss-Prot;Acc:Q9SKP9]	9060561	9062093	40.7
AT2G21150	XCT	Protein XAP5 CIRCADIAN TIMEKEEPER [Source:UniProtKB/Swiss-Prot;Acc:Q8H110]	9065154	9068228	38.08
AT2G21160		Translocon-associated protein subunit alpha [Source:UniProtKB/Swiss-Prot;Acc:P45434]	9068252	9070473	38.25
AT2G21170	TIM	TIM [Source:UniProtKB/TrEMBL;Acc:A0A178VN28]	9070734	9074470	38.77
AT2G21180		At2g21180/F26H11.6 [Source:UniProtKB/TrEMBL;Acc:Q9SKP5]	9074660	9075631	37.45
AT2G21187		other RNA [Source:TAIR;Acc:AT2G21187]	9076289	9077494	37.73
AT2G21185		Expressed protein [Source:UniProtKB/TrEMBL;Acc:Q8S8H4]	9076437	9077048	43.14
AT2G21188		other RNA [Source:TAIR;Acc:AT2G21188]	9079042	9079538	41.05
AT2G21190		ER lumen protein retaining receptor family protein [Source:UniProtKB/TrEMBL;Acc:Q9SKP4]	9080760	9083009	38.4
AT2G21195		unknown protein; Ha. [Source:TAIR;Acc:AT2G21195]	9083144	9083909	38.12
AT2G21200		At2g21200 [Source:UniProtKB/TrEMBL;Acc:Q9SKP3]	9083906	9084803	41.65
AT2G21210		SAUR-like auxin-responsive protein family [Source:TAIR;Acc:AT2G21210]	9084628	9086029	38.37
AT2G07375			9086997	9087232	39.83
AT2G07385			9087633	9087989	35.01
AT2G21220		Putative auxin-regulated protein [Source:UniProtKB/TrEMBL;Acc:Q9SIG9]	9089226	9090052	38.33
AT2G07405			9090639	9090837	36.68
AT2G21230		Basic-leucine zipper (BZIP) transcription factor family protein [Source:UniProtKB/TrEMBL;Acc:B3H7M2]	9093136	9096414	40.13
AT2G21235		Basic-leucine zipper (BZIP) transcription factor family protein [Source:UniProtKB/TrEMBL;Acc:F4IGK0]	9096527	9098767	42.79
AT2G21237		At2g21237 [Source:UniProtKB/TrEMBL;Acc:Q8L8K9]	9100536	9101215	42.94
AT2G21240	BPC4	Protein BASIC PENTACYSTEINE4 [Source:UniProtKB/Swiss-Prot;Acc:Q8S8C6]	9101277	9103141	39.62
AT2G21250		NAD(P)-linked oxidoreductase superfamily protein [Source:UniProtKB/TrEMBL;Acc:Q9SJV2]	9103136	9105187	39.18
AT2G21260		At2g21260 [Source:UniProtKB/TrEMBL;Acc:Q9SJV1]	9105583	9107380	40.21
AT2G21270	UFD1	AT2G21270 protein [Source:UniProtKB/TrEMBL;Acc:A8MQW3]	9107546	9110275	38.75
AT2G21280	GC1	NAD(P)-binding Rossmann-fold superfamily protein [Source:TAIR;Acc:AT2G21280]	9110136	9112776	39.34
AT2G21290		30S ribosomal protein S31, mitochondrial [Source:UniProtKB/Swiss-Prot;Acc:Q9SJU8]	9112798	9113597	42.75
AT2G21300	KIN7E	Kinesin-like protein KIN-7E [Source:UniProtKB/Swiss-Prot;Acc:F4IGL2]	9114032	9119977	40.43
AT2G21320	BBX18	B-box zinc finger protein 18 [Source:UniProtKB/Swiss-Prot;Acc:Q9SJU5]	9126263	9127987	36.93
AT2G21330	FBA1	Fructose-bisphosphate aldolase 1, chloroplastic [Source:UniProtKB/Swiss-Prot;Acc:Q9SJU4]	9128122	9130619	42.87
AT2G21340	DTX46	Protein DETOXIFICATION 46, chloroplastic [Source:UniProtKB/Swiss-Prot;Acc:Q8W4G3]	9132381	9136787	39.35
AT2G21350		RNA-binding CRS1 / YhbY (CRM) domain protein [Source:UniProtKB/TrEMBL;Acc:F4IGM0]	9136203	9137284	42.88
AT2G21360		pre-tRNA [Source:TAIR;Acc:AT2G21360]	9137356	9137427	56.94
AT2G21370	XK-1	D-ribulose kinase [Source:UniProtKB/Swiss-Prot;Acc:Q8L794]	9137451	9140142	40.19
AT2G21380	KIN7M	Kinesin-like protein KIN-7M, chloroplastic [Source:UniProtKB/Swiss-Prot;Acc:Q9SJU0]	9141465	9149330	37.38
AT2G21385		Expressed protein [Source:UniProtKB/TrEMBL;Acc:Q94AU3]	9149577	9151970	37.34
AT2G21390		Coatomer subunit alpha-2 [Source:UniProtKB/Swiss-Prot;Acc:Q9SJT9]	9151940	9156906	42.52
AT2G21400	SRS3	SHI-related sequence3 [Source:TAIR;Acc:AT2G21400]	9158310	9159732	33.87
AT2G21410	VHA-A2	V-type proton ATPase subunit a2 [Source:UniProtKB/Swiss-Prot;Acc:Q9SJT7]	9162516	9168556	38.3
AT2G21420		RBR-type E3 ubiquitin transferase [Source:UniProtKB/TrEMBL;Acc:Q9SJT6]	9169713	9171746	36.68
AT2G21430	RD19B	Probable cysteine protease RD19B [Source:UniProtKB/Swiss-Prot;Acc:P43295]	9171872	9173490	42.12
AT2G21440		Expressed protein [Source:UniProtKB/TrEMBL;Acc:Q9SJT4]	9173384	9179166	38.87
AT2G21450	CHR34	chromatin remodeling 34 [Source:TAIR;Acc:AT2G21450]	9179410	9183037	40.16
AT2G21455		SNF2 domain CLASSY-like protein [Source:UniProtKB/TrEMBL;Acc:A0A1P8B2G7]	9184150	9185623	32.36
AT2G07415			9184422	9184943	37.36
AT2G21465		Defensin-like protein 291 [Source:UniProtKB/Swiss-Prot;Acc:Q2V472]	9195477	9195984	33.86
AT2G21470	SAE2	SUMO-activating enzyme subunit 2 [Source:UniProtKB/Swiss-Prot;Acc:Q9SJT1]	9198569	9202487	37.82
AT2G21480		Probable receptor-like protein kinase At2g21480 [Source:UniProtKB/Swiss-Prot;Acc:Q9SJT0]	9202494	9205471	43.22
AT2G21490	LEA	Probable dehydrin LEA [Source:UniProtKB/Swiss-Prot;Acc:Q96261]	9205990	9207125	41.37
AT2G21500		At2g21560 [Source:UniProtKB/TrEMBL;Acc:Q8RWF9]	9207432	9210231	38.25
AT2G21510		DNAJ heat shock N-terminal domain-containing protein [Source:TAIR;Acc:AT2G21510]	9210623	9213102	37.02
AT2G07425			9213595	9213903	36.89
AT2G21520		Sec14p-like phosphatidylinositol transfer family protein [Source:UniProtKB/TrEMBL;Acc:B3H588]	9215519	9219236	37.41
AT2G21530		At2g21530 [Source:UniProtKB/TrEMBL;Acc:Q8GWP4]	9219252	9220608	37.07
AT2G21540	SFH3	Phosphatidylinositol/phosphatidylcholine transfer protein SFH3 [Source:UniProtKB/Swiss-Prot;Acc:Q93ZE9]	9220505	9224496	36.3

AT2G21550		Bifunctional dihydrofolate reductase-thymidylate synthase [Source:UniProtKB/TrEMBL;Acc:Q9SIK4]	9226917	9230205	35.39
AT2G21560		Nucleolar-like protein [Source:UniProtKB/TrEMBL;Acc:Q9SIK3]	9230208	9231580	37
AT2G21570		pre-tRNA [Source:TAIR;Acc:AT2G21570]	9234193	9234276	53.57
AT2G21580	RPS25B	40S ribosomal protein S25-2 [Source:UniProtKB/Swiss-Prot;Acc:Q9SIK2]	9236481	9237846	38.29
AT2G21590	APL4	Probable glucose-1-phosphate adenylyltransferase large subunit, chloroplastic [Source:UniProtKB/Swiss-Prot;Acc:Q9SIK1]	9238296	9242481	35.81
AT2G21595			9242205	9242951	33.07
AT2G21600	RER1B	Protein RER1B [Source:UniProtKB/Swiss-Prot;Acc:O48671]	9242782	9244945	37.94
AT2G21610	PE11	PE11 [Source:UniProtKB/TrEMBL;Acc:A0A178W118]	9245038	9247052	34.34
AT2G21620	RD2	Adenine nucleotide alpha hydrolases-like superfamily protein [Source:UniProtKB/TrEMBL;Acc:Q94II5]	9248525	9250311	39.28
AT2G21630		At2g21630 [Source:UniProtKB/TrEMBL;Acc:Q9SIJ7]	9250298	9253915	40.55
AT2G21640		Marker for oxidative stress response protein [Source:UniProtKB/TrEMBL;Acc:Q9SIJ6]	9254378	9255743	36.31
AT2G07435			9257096	9257415	34.38
AT2G07445			9258716	9258942	36.12
AT2G21650	RL2	RSM1 [Source:UniProtKB/TrEMBL;Acc:A0A178VZA8]	9259511	9260697	36.23
AT2G21655		At2g21655 [Source:UniProtKB/TrEMBL;Acc:Q8S8C9]	9263537	9264098	30.78
AT2G21660	RBG7	Glycine-rich RNA-binding protein 7 [Source:UniProtKB/Swiss-Prot;Acc:Q03250]	9265084	9266557	44.23
AT2G21670		pre-tRNA [Source:TAIR;Acc:AT2G21670]	9266809	9266889	51.85
AT2G21680		Putative F-box/kelch-repeat protein At2g21680 [Source:UniProtKB/Swiss-Prot;Acc:Q9SIJ3]	9267730	9269019	45.66
AT2G21690		Putative glycine-rich RNA binding protein [Source:UniProtKB/TrEMBL;Acc:Q9SJ28]	9270114	9270597	36.36
AT2G21700		pre-tRNA [Source:TAIR;Acc:AT2G21700]	9270663	9270734	47.22
AT2G21710	MTERF2	Transcription termination factor MTERF2, chloroplastic [Source:UniProtKB/Swiss-Prot;Acc:F4IHL3]	9270784	9273444	40.4
AT2G21720		Plant protein of unknown function (DUF639) [Source:TAIR;Acc:AT2G21720]	9273696	9276961	38.27
AT2G21725		Putative defensin-like protein 79 [Source:UniProtKB/Swiss-Prot;Acc:Q2V470]	9277659	9278072	30.92
AT2G21727		ECA1 gametogenesis family protein (DUF784) [Source:UniProtKB/TrEMBL;Acc:A8MQ83]	9278567	9279080	34.05
AT2G21730	CAD2	Cinnamyl alcohol dehydrogenase 2 [Source:UniProtKB/Swiss-Prot;Acc:Q9SJ25]	9279931	9282178	40.04
AT2G21740	EC1.2	Egg cell-secreted protein 1.2 [Source:UniProtKB/Swiss-Prot;Acc:Q9SJ24]	9281986	9282387	48.51
AT2G21750	EC1.3	Egg cell-secreted protein 1.3 [Source:UniProtKB/Swiss-Prot;Acc:Q9SJ23]	9283367	9283774	49.51
AT2G21770	CESA9	Probable cellulose synthase A catalytic subunit 9 [UDP-forming] [Source:UniProtKB/Swiss-Prot;Acc:Q9SJ22]	9284391	9289744	39.73
AT2G21780		At2g21780 [Source:UniProtKB/TrEMBL;Acc:Q9SJ21]	9290705	9291669	37.82
AT2G21790	RNR1	Ribonucleoside-diphosphate reductase large subunit [Source:UniProtKB/Swiss-Prot;Acc:Q9SJ20]	9293261	9297861	38.95
AT2G21800	EME1A	essential meiotic endonuclease 1A [Source:TAIR;Acc:AT2G21800]	9298078	9301926	37.62
AT2G21810		Cysteine/Histidine-rich C1 domain family protein [Source:UniProtKB/TrEMBL;Acc:Q9SJ18]	9302254	9302640	39.79
AT2G21820		At2g21820 [Source:UniProtKB/TrEMBL;Acc:Q9SJ17]	9302853	9303408	43.17
AT2G21830		Cysteine/Histidine-rich C1 domain family protein [Source:UniProtKB/TrEMBL;Acc:Q9SJ16]	9303713	9306025	39.78
AT2G21840		Cysteine/Histidine-rich C1 domain family protein [Source:UniProtKB/TrEMBL;Acc:Q0WPH4]	9307024	9310358	38.89
AT2G21850		Cysteine/Histidine-rich C1 domain family protein [Source:UniProtKB/TrEMBL;Acc:F4III1]	9315204	9318238	41.68
AT2G21860		At2g21860/F7D8.18 [Source:UniProtKB/TrEMBL;Acc:Q9SJ13]	9318122	9320024	43.35
AT2G21870	MGP1	Probable ATP synthase 24 kDa subunit, mitochondrial [Source:UniProtKB/Swiss-Prot;Acc:Q9SJ12]	9320190	9322867	36.56
AT2G21880	RABG2	RAB7A [Source:UniProtKB/TrEMBL;Acc:A0A178VZI8]	9324633	9326426	35.23
AT2G21890	CAD3	Cinnamyl alcohol dehydrogenase 3 [Source:UniProtKB/Swiss-Prot;Acc:Q9SJ10]	9331058	9332646	40.59
AT2G21900	WRKY59	At2g21900 [Source:UniProtKB/TrEMBL;Acc:Q1LYW5]	9333844	9336278	30.8
AT2G21910	CYP96A5	Cytochrome P450, family 96, subfamily A, polypeptide 5 [Source:UniProtKB/TrEMBL;Acc:Q9SJ08]	9341431	9343220	40.28
AT2G21920		Putative B3 domain-containing protein At2g21920 [Source:UniProtKB/Swiss-Prot;Acc:Q9SJ07]	9343410	9347159	33.84
AT2G21930		F-box protein At2g21930 [Source:UniProtKB/Swiss-Prot;Acc:Q9SJ06]	9347641	9349178	38.82
AT2G21940	SK1	Shikimate kinase 1 [Source:UniProtKB/TrEMBL;Acc:F4IIJ2]	9350543	9353231	37.11
AT2G21950	SKIP6	F-box/kelch-repeat protein SKIP6 [Source:UniProtKB/Swiss-Prot;Acc:Q9SJ04]	9353174	9354645	44.7
AT2G07475			9353383	9354717	44.94
AT2G21960		Expressed protein [Source:UniProtKB/TrEMBL;Acc:Q9SJ03]	9354842	9357091	40
AT2G21970	02-Sep	SEP2 [Source:UniProtKB/TrEMBL;Acc:A0A178VQ84]	9356983	9357967	42.03
AT2G21980		HAUS augmin-like complex subunit [Source:UniProtKB/TrEMBL;Acc:Q9SJ01]	9358882	9359061	45
AT2G21990		Uncharacterized protein At2g21990/F7D8.31 [Source:UniProtKB/TrEMBL;Acc:Q9SJ00]	9359318	9360664	41.65
AT2G22000	PEP6	Elicitor peptide 6 [Source:UniProtKB/Swiss-Prot;Acc:Q9SIZ9]	9362113	9363203	31.71
AT2G07485			9362602	9362910	27.51
AT2G22010	RKP	Related to KPC1 [Source:UniProtKB/TrEMBL;Acc:F4IIK4]	9363109	9369461	39.08
AT2G07495			9363817	9364086	36.67
AT2G22030		F-box/kelch-repeat protein At2g22030 [Source:UniProtKB/Swiss-Prot;Acc:Q9SI02]	9372530	9373681	42.62
AT2G22040	LST8-2	Non-functional target of rapamycin complex subunit LST8-2 [Source:UniProtKB/Swiss-Prot;Acc:F4IIK6]	9374438	9376260	36.92
AT2G22050		F-box/kelch-repeat protein At2g22050 [Source:UniProtKB/Swiss-Prot;Acc:Q1PF20]	9376684	9377862	43.26
AT2G22055	RALFL15	Protein RALF-like 15 [Source:UniProtKB/Swiss-Prot;Acc:A8MQM7]	9379465	9380114	33.08
AT2G22060		Galactose oxidase/kelch repeat protein [Source:UniProtKB/TrEMBL;Acc:Q9SHZ9]	9380992	9382145	43.15
AT2G22070	PCMP-H41	Pentatricopeptide repeat-containing protein At2g22070 [Source:UniProtKB/Swiss-Prot;Acc:Q9SHZ8]	9383397	9386266	41.36
AT2G22080		At2g22080 [Source:UniProtKB/TrEMBL;Acc:Q7XA71]	9386431	9388241	36.22
AT2G22090	UBA1A	RNA-binding (RRM/RBD/RNP motifs) family protein [Source:UniProtKB/TrEMBL;Acc:F4IIL5]	9388260	9390575	40.16
AT2G22088		unknown protein; FUNCTIONS IN: molecular_function unknown; INVOLVED IN: biological_process unknown; LOCATED IN: cel	9388384	9389049	33.93
AT2G07515			9389725	9390526	44.14
AT2G22100	UBA1B	UBP1-associated proteins 1B [Source:UniProtKB/Swiss-Prot;Acc:Q9SHZ5]	9391554	9393594	39.74
AT2G22110		pre-tRNA [Source:TAIR;Acc:AT2G22110]	9393670	9393743	58.11
AT2G22120		RING/FYVE/PHD zinc finger superfamily protein [Source:UniProtKB/TrEMBL;Acc:F4IIL7]	9393791	9397098	37.73
AT2G22121	LCR35	Putative defensin-like protein 154 [Source:UniProtKB/Swiss-Prot;Acc:P82750]	9397864	9398259	32.32
AT2G22122		At2g22122 [Source:UniProtKB/TrEMBL;Acc:Q8LF43]	9403067	9403835	32.9
AT2G22125	CSI1	Protein CELLULOSE SYNTHASE INTERACTIVE 1 [Source:UniProtKB/Swiss-Prot;Acc:F4IIM1]	9406059	9414578	42.96
AT2G22140	EME1B	Crossover junction endonuclease EME1B [Source:UniProtKB/Swiss-Prot;Acc:C5H8J1]	9414557	9418711	37.18
AT2G22145		ECA1 gametogenesis related family protein [Source:UniProtKB/TrEMBL;Acc:A8MRJ4]	9420240	9420623	37.76
AT2G22155		Encodes a ECA1 gametogenesis related family protein [Source:TAIR;Acc:AT2G22155]	9423833	9424243	32.85
AT2G22160		Cysteine proteinases superfamily protein [Source:UniProtKB/TrEMBL;Acc:Q9SIE8]	9425143	9425460	41.19
AT2G22170	PLAT2	PLAT domain-containing protein 2 [Source:UniProtKB/Swiss-Prot;Acc:Q9SIE7]	9426796	9428170	40.07
AT2G22180		Hydroxyproline-rich glycoprotein family protein [Source:UniProtKB/TrEMBL;Acc:Q9SIE6]	9428743	9430141	43.03
AT2G07525			9430577	9430967	41.69
AT2G07535			9431751	9431984	32.91
AT2G07545			9432221	9432641	32.78
AT2G22190	TPPE	Probable trehalose-phosphate phosphatase E [Source:UniProtKB/Swiss-Prot;Acc:Q67X99]	9433616	9436658	29.44
AT2G22200	ERF056	Ethylene-responsive transcription factor ERF056 [Source:UniProtKB/Swiss-Prot;Acc:Q9SIE4]	9443073	9444428	39.23
AT2G22220		pre-tRNA [Source:TAIR;Acc:AT2G22220]	9449809	9449882	51.35
AT2G22230		At2g22230/T26C19.11 [Source:UniProtKB/TrEMBL;Acc:Q9SIE3]	9449930	9451623	39.2
AT2G22240	IPS2	Inositol-3-phosphate synthase isozyme 2 [Source:UniProtKB/Swiss-Prot;Acc:Q38862]	9451605	9454300	40.73
AT2G22241		unknown protein; LOCATED IN: mitochondrion; Ha. [Source:TAIR;Acc:AT2G22241]	9454244	9454399	52.56
AT2G22250	PAT	Bifunctional aspartate aminotransferase and glutamate/aspartate-prephenate aminotransferase [Source:UniProtKB/Swiss-Prot;Acc:Q	9457786	9460919	38.16
AT2G22260		oxidoreductase, 2OG-Fe(II) oxygenase family protein [Source:TAIR;Acc:AT2G22260]	9460918	9463561	39.71
AT2G22270		Expressed protein [Source:UniProtKB/TrEMBL;Acc:Q9SID9]	9463592	9465522	38.58
AT2G22280		pre-tRNA [Source:TAIR;Acc:AT2G22280]	9465581	9465653	57.53
AT2G22290	RABH1D	Ras-related protein RABH1d [Source:UniProtKB/Swiss-Prot;Acc:Q9SID8]	9466389	9467948	35.64
AT2G22300	CAMTA3	Calmodulin-binding transcription activator 3 [Source:UniProtKB/Swiss-Prot;Acc:Q8GSA7]	9471219	9476646	37.64
AT2G22310	UBP4	UBP4 [Source:UniProtKB/TrEMBL;Acc:A0A178W1M0]	9476578	9478959	38.12
AT2G22320		Uncharacterized protein At2g22320 [Source:UniProtKB/TrEMBL;Acc:Q9SID6]	9480105	9480440	38.39
AT2G07555			9481548	9482100	42.86
AT2G22330	CYP79B3	cytochrome P450, family 79, subfamily B, polypeptide 3 [Source:TAIR;Acc:AT2G22330]	9488413	9491404	39.61
AT2G22340		Transmembrane protein [Source:UniProtKB/TrEMBL;Acc:Q9SJZ9]	9492864	9494112	38.43
AT2G07575			9495178	9495376	37.69
AT2G22345		Putative defensin-like protein 72 [Source:UniProtKB/Swiss-Prot;Acc:Q2V2S9]	9495457	9495848	29.08
AT2G22360	DJA6	Chaperone protein dnaJ A6, chloroplastic [Source:UniProtKB/Swiss-Prot;Acc:Q9SJZ7]	9497570	9500719	38.73
AT2G22370	MED18	MED18 [Source:UniProtKB/TrEMBL;Acc:A0A178VRQ8]	9500784	9502210	37.77
AT2G22380		pre-tRNA [Source:TAIR;Acc:AT2G22380]	9502280	9502353	51.35
AT2G22400		S-adenosyl-L-methionine-dependent methyltransferases superfamily protein [Source:UniProtKB/TrEMBL;Acc:Q8L601]	9504659	9508895	39.11
AT2G22410	PCMP-E28	Pentatricopeptide repeat-containing protein At2g22410, mitochondrial [Source:UniProtKB/Swiss-Prot;Acc:Q9SJZ3]	9509016	9511231	40.84
AT2G22420	PER17	Peroxidase 17 [Source:UniProtKB/Swiss-Prot;Acc:Q9SJZ2]	9513024	9514552	38.13

AT2G22425		Probable signal peptidase complex subunit 1 [Source:UniProtKB/Swiss-Prot;Acc:Q944J0]	9514695	9515674	38.16
AT2G22426		unknown protein; Ha. [Source:TAIR;Acc:AT2G22426]	9516910	9517386	31.87
AT2G22430	ATHB-6	Homeobox-leucine zipper protein ATHB-6 [Source:UniProtKB/Swiss-Prot;Acc:P46668]	9525988	9527845	39.24
AT2G22440		Non-LTR retroelement reverse transcriptase [Source:UniProtKB/TrEMBL;Acc:Q9SJZ0]	9528910	9530060	43.35
AT2G22450	RIBA2	Monofunctional riboflavin biosynthesis protein RIBA 2, chloroplastic [Source:UniProtKB/Swiss-Prot;Acc:Q6NLQ7]	9530365	9533066	38.64
AT2G22460		Uncharacterized protein At2g22460/F14M13.14 [Source:UniProtKB/TrEMBL;Acc:Q84RJ3]	9533094	9534188	39.27
AT2G22465		Transmembrane protein [Source:UniProtKB/TrEMBL;Acc:A0A1P8AZV8]	9533559	9533988	42.09
AT2G22470	AGP2	Classical arabinogalactan protein 2 [Source:UniProtKB/Swiss-Prot;Acc:Q9SJY7]	9538127	9538963	41.34
AT2G22475	GEM	GLABRA2 expression modulator [Source:UniProtKB/Swiss-Prot;Acc:Q8S8F8]	9541128	9545038	36.61
AT2G22480	PFK5	ATP-dependent 6-phosphofructokinase 5, chloroplastic [Source:UniProtKB/Swiss-Prot;Acc:Q8VYN6]	9545283	9548640	37.19
AT2G07585			9549145	9550342	35.81
AT2G22482			9549649	9550084	38.07
AT2G07595			9549923	9550378	38.16
AT2G22490	CYCD2;1	Cyclin D21 [Source:UniProtKB/TrEMBL;Acc:F4IJJ3]	9553797	9556167	38.17
AT2G07605			9560355	9560446	38.04
AT2G07615			9560536	9560623	38.64
AT2G22496	MIR779A	MIR779a; miRNA [Source:TAIR;Acc:AT2G22496]	9560761	9560923	32.52
AT2G22500	PUMP5	Mitochondrial uncoupling protein 5 [Source:UniProtKB/Swiss-Prot;Acc:Q9SJY5]	9562774	9564850	40.06
AT2G22510		At2g22510 [Source:UniProtKB/TrEMBL;Acc:Q9SJY4]	9569023	9569784	38.06
AT2G22520		Uncharacterized protein At2g22520 [Source:UniProtKB/TrEMBL;Acc:Q9SJY3]	9572066	9572791	37.88
AT2G22530		Alkaline-phosphatase-like family protein [Source:UniProtKB/TrEMBL;Acc:F4IJJ8]	9572768	9577616	38.54
AT2G22540	SVP	SVP [Source:UniProtKB/TrEMBL;Acc:A0A384KDB8]	9579647	9583901	33.16
AT2G22560	NET2D	NET2D [Source:UniProtKB/TrEMBL;Acc:A0A178VLF4]	9585549	9589366	39.79
AT2G22570	NIC1	NIC1 [Source:UniProtKB/TrEMBL;Acc:A0A178VQ19]	9589435	9590932	36.72
AT2G22580		pre-tRNA [Source:TAIR;Acc:AT2G22580]	9591027	9591099	61.64
AT2G07645			9591789	9592305	42.55
AT2G22590	UGT91A1	UDP-glycosyltransferase 91A1 [Source:UniProtKB/Swiss-Prot;Acc:Q940V3]	9592956	9594579	44.83
AT2G22600		RNA-binding KH domain-containing protein [Source:UniProtKB/TrEMBL;Acc:F4IJK5]	9596526	9599227	41.34
AT2G22610		Di-glucose binding protein with Kinesin motor domain [Source:TAIR;Acc:AT2G22610]	9599420	9604776	38.17
AT2G07805			9604211	9604488	41.01
AT2G22620		Rhamnogalacturonate lyase family protein [Source:UniProtKB/TrEMBL;Acc:Q9ZQ51]	9604772	9610661	31.53
AT2G07840			9614748	9614994	32.39
AT2G22630	AGL17	AGL17 [Source:UniProtKB/TrEMBL;Acc:A0A384L577]	9618207	9622163	31.49
AT2G22640	BRK1	Protein BRICK 1 [Source:UniProtKB/Swiss-Prot;Acc:Q94JY4]	9622997	9624335	35.7
AT2G22650		FAD-dependent oxidoreductase family protein [Source:UniProtKB/TrEMBL;Acc:Q5S4X7]	9624505	9627072	40.03
AT2G22660	GRDP1	Glycine-rich domain-containing protein 1 [Source:UniProtKB/Swiss-Prot;Acc:Q9ZQ47]	9627342	9631283	40.49
AT2G22668	MIR405A	MIR405A; miRNA [Source:TAIR;Acc:AT2G22668]	9634957	9635113	35.03
AT2G22670	IAA8	Auxin-responsive protein [Source:UniProtKB/TrEMBL;Acc:F4IKE6]	9636346	9638761	37.87
AT2G22680	WAVH1	E3 ubiquitin-protein ligase WAVH1 [Source:UniProtKB/Swiss-Prot;Acc:Q9ZQ46]	9645270	9647655	46.65
AT2G22690		At2g22690 [Source:UniProtKB/TrEMBL;Acc:Q9ZQ45]	9649690	9651574	38.83
AT2G22720		SPT2 chromatin protein [Source:UniProtKB/TrEMBL;Acc:F4IKF3]	9657339	9660803	40.14
AT2G22730		Major facilitator superfamily protein [Source:TAIR;Acc:AT2G22730]	9660858	9664133	34.98
AT2G22740	SUVH6	Histone-lysine N-methyltransferase, H3 lysine-9 specific SUVH6 [Source:UniProtKB/Swiss-Prot;Acc:Q8VZ17]	9662638	9667038	40.45
AT2G07845			9665550	9666686	44.42
AT2G22750		basic helix-loop-helix (bHLH) DNA-binding superfamily protein [Source:TAIR;Acc:AT2G22750]	9671673	9673928	33.82
AT2G22760	BHLH19	Uncharacterized protein At2g22760 (Fragment) [Source:UniProtKB/TrEMBL;Acc:C0SV56]	9677844	9679502	34.42
AT2G22770	NAI1	NAI1 [Source:UniProtKB/TrEMBL;Acc:A0A178VWP9]	9684562	9686613	34.16
AT2G22780	PMDH1	PMDH1 [Source:UniProtKB/TrEMBL;Acc:A0A384LAR8]	9689295	9692154	38.39
AT2G22790		Uncharacterized protein At2g22790 [Source:UniProtKB/TrEMBL;Acc:O82400]	9695832	9697127	43.9
AT2G22795		unknown protein; BEST Arabidopsis thaliana protein match is: unknown protein (TAIR:AT4G37820.1); Ha. [Source:TAIR;Acc:AT2	9697131	9699944	39.37
AT2G22800	HAT9	HAT9 [Source:UniProtKB/TrEMBL;Acc:A0A178VZE8]	9704632	9706217	35.94
AT2G22805		Putative defensin-like protein 189 [Source:UniProtKB/Swiss-Prot;Acc:Q2V468]	9712264	9713135	27.87
AT2G22807		Putative defensin-like protein 190 [Source:UniProtKB/Swiss-Prot;Acc:Q2V467]	9715049	9716192	26.84
AT2G22810	ACS4	1-aminocyclopropane-1-carboxylate synthase 4 [Source:UniProtKB/Swiss-Prot;Acc:Q43309]	9717602	9719594	39.74
AT2G22820		At2g22820 [Source:UniProtKB/TrEMBL;Acc:O82813]	9721075	9721425	33.9
AT2G22821		other RNA [Source:TAIR;Acc:AT2G22821]	9721132	9721960	35.71
AT2G22830	SQE2	Squalene epoxidase 2, mitochondrial [Source:UniProtKB/Swiss-Prot;Acc:O81000]	9723615	9726384	39.64
AT2G22840	GRF1	Growth-regulating factor 1 [Source:UniProtKB/Swiss-Prot;Acc:O81001]	9728480	9731301	39.97
AT2G22850	AtbZIP6	At2g22850 [Source:UniProtKB/TrEMBL;Acc:O81002]	9731279	9733612	36.08
AT2G07850			9732473	9733328	40.42
AT2G22860	PSK2	Phytosulfokines 2 [Source:UniProtKB/Swiss-Prot;Acc:O81003]	9737583	9738384	33.92
AT2G22870	EMB2001	GTP-binding protein At2g22870 [Source:UniProtKB/Swiss-Prot;Acc:O81004]	9739408	9741700	37.37
AT2G22880		At2g22880 [Source:UniProtKB/TrEMBL;Acc:O81005]	9741119	9741463	45.22
AT2G22890	FAD4L2	Fatty acid desaturase 4-like 2, chloroplastic [Source:UniProtKB/Swiss-Prot;Acc:O81006]	9742224	9743467	40.11
AT2G22900	GT7	Putative glycosyltransferase 7 [Source:UniProtKB/Swiss-Prot;Acc:O81007]	9744146	9746435	37.64
AT2G22905		At2g22905 [Source:UniProtKB/TrEMBL;Acc:Q3EBW2]	9748771	9749542	38.21
AT2G22910	NAGS1	Probable amino-acid acetyltransferase NAGS1, chloroplastic [Source:UniProtKB/Swiss-Prot;Acc:Q84JF4]	9749857	9753103	41.39
AT2G22920	SCPL12	Serine carboxypeptidase-like 12 [Source:UniProtKB/Swiss-Prot;Acc:O81009]	9753631	9757600	30.76
AT2G22930	UGT79B8	UDP-glycosyltransferase 79B8 [Source:UniProtKB/Swiss-Prot;Acc:O81010]	9759595	9761240	41.37
AT2G22940		Uncharacterized protein At2g22940 [Source:UniProtKB/TrEMBL;Acc:O81011]	9761655	9763462	35.79
AT2G22941		Defensin-like (DEFL) family protein [Source:TAIR;Acc:AT2G22941]	9762066	9762586	34.55
AT2G22942		Growth factor [Source:UniProtKB/TrEMBL;Acc:B3H4C2]	9763740	9764612	32.99
AT2G22950	ACA7	Putative calcium-transporting ATPase 7, plasma membrane-type [Source:UniProtKB/Swiss-Prot;Acc:O64806]	9766058	9769957	39.79
AT2G22955		other RNA [Source:TAIR;Acc:AT2G22955]	9770700	9771553	32.32
AT2G22960		alpha/beta-Hydrolases superfamily protein [Source:TAIR;Acc:AT2G22960]	9770894	9772977	30.47
AT2G22970	SCPL11	Serine carboxypeptidase-like 11 [Source:UniProtKB/TrEMBL;Acc:A8MQP0]	9774749	9778561	31.26
AT2G22980	SCPL13	Serine carboxypeptidase-like 13 [Source:UniProtKB/TrEMBL;Acc:A8MQS0]	9778759	9783310	31.2
AT2G22990	SNG1	sinapoylglucose 1 [Source:TAIR;Acc:AT2G22990]	9786087	9790335	30.95
AT2G07855			9791892	9792186	38.31
AT2G23000	SCPL10	Scpl10 [Source:UniProtKB/TrEMBL;Acc:A0A178VYK6]	9792166	9796408	32.43
AT2G23010	SCPL9	SCPL9 [Source:UniProtKB/TrEMBL;Acc:A0A178W1Q9]	9798757	9802676	31.35
AT2G23020		pre-tRNA [Source:TAIR;Acc:AT2G23020]	9803217	9803288	59.72
AT2G23030	SRK2J	SNRK2.9 [Source:UniProtKB/TrEMBL;Acc:A0A178VSC8]	9803492	9806731	30.93
AT2G23040		unknown protein; Ha. [Source:TAIR;Acc:AT2G23040]	9808590	9810089	32.8
AT2G23050	NPY4	BTB/POZ domain-containing protein NPY4 [Source:UniProtKB/Swiss-Prot;Acc:O64814]	9810525	9812662	37.65
AT2G23060		Probable N-acetyltransferase HLS1-like [Source:UniProtKB/Swiss-Prot;Acc:O64815]	9812771	9814738	39.13
AT2G07860			9819622	9819962	40.47
AT2G07865			9820590	9820832	37.04
AT2G07870			9820590	9820832	37.04
AT2G23067		Putative membrane lipoprotein [Source:UniProtKB/TrEMBL;Acc:B3H7D9]	9822005	9822148	42.36
AT2G23070	CKA4	Casein kinase II subunit alpha-4, chloroplastic [Source:UniProtKB/Swiss-Prot;Acc:O64816]	9823642	9826898	38.07
AT2G23080	CKA3	Casein kinase II subunit alpha-3 [Source:UniProtKB/Swiss-Prot;Acc:O64817]	9827104	9829537	36.4
AT2G23090		Uncharacterized protein At2g23090 [Source:UniProtKB/Swiss-Prot;Acc:O64818]	9829450	9830434	34.82
AT2G07875			9829803	9830074	29.78
AT2G23093		Major facilitator superfamily protein [Source:UniProtKB/TrEMBL;Acc:Q8GXH4]	9832032	9834739	36.71
AT2G23096	P4H13	Prolyl 4-hydroxylase 13 [Source:UniProtKB/Swiss-Prot;Acc:F4ILF8]	9834766	9836309	37.89
AT2G23100		Cysteine/Histidine-rich C1 domain family protein [Source:UniProtKB/TrEMBL;Acc:F4ILF9]	9837120	9839824	40.85
AT2G23110		At2g23110 [Source:UniProtKB/TrEMBL;Acc:O64820]	9840456	9841366	37.65
AT2G23120		Expressed protein [Source:UniProtKB/TrEMBL;Acc:Q8S8R1]	9841842	9842634	40.61
AT2G23118		unknown protein; Ha. [Source:TAIR;Acc:AT2G23118]	9841882	9842025	46.53
AT2G23130	AGP17	Lysine-rich arabinogalactan protein 17 [Source:UniProtKB/Swiss-Prot;Acc:O22194]	9844134	9845385	39.38
AT2G23140		RING-type E3 ubiquitin transferase [Source:UniProtKB/TrEMBL;Acc:F4ILG6]	9845410	9849379	40.53
AT2G23142	SPH10	S-protein homolog 10 [Source:UniProtKB/Swiss-Prot;Acc:B3H6H8]	9850176	9850619	35.59
AT2G07885			9850973	9851565	39.63
AT2G23148	SPH11	S-protein homolog 11 [Source:UniProtKB/Swiss-Prot;Acc:B3H4B5]	9853609	9854156	32.85

	AT2G07890			9855388	9855804	42.21
	AT2G23150	NRAMP3	Metal transporter Nramp3 [Source:UniProtKB/Swiss-Prot;Acc:Q9SNV9]	9856149	9858778	39.66
	AT2G23160		F-box protein At2g23160 [Source:UniProtKB/Swiss-Prot;Acc:Q1KS79]	9862470	9863744	39.69
	AT2G23170	GH3.3	Indole-3-acetic acid-amido synthetase GH3.3 [Source:UniProtKB/Swiss-Prot;Acc:O22190]	9863855	9866562	40.36
	AT2G23180	CYP96A1	Cytochrome P450, family 96, subfamily A, polypeptide 1 [Source:UniProtKB/TrEMBL;Acc:O22189]	9874907	9876711	39.11
	AT2G23190	CYP81D7	Cytochrome P450, family 81, subfamily D, polypeptide 7 [Source:UniProtKB/TrEMBL;Acc:O22188]	9877058	9879007	41.49
	AT2G23200		Probable receptor-like protein kinase At2g23200 [Source:UniProtKB/Swiss-Prot;Acc:O22187]	9879275	9881924	42.83
	AT2G23210		UDP-Glycosyltransferase superfamily protein [Source:UniProtKB/TrEMBL;Acc:Q1PF14]	9881260	9883397	42.28
	AT2G23220	CYP81D6	Cytochrome P450, family 81, subfamily D, polypeptide 6 [Source:UniProtKB/TrEMBL;Acc:O22185]	9884550	9886752	38.49
	AT2G23230		Terpenoid cyclases/Protein prenyltransferases superfamily protein [Source:TAIR;Acc:AT2G23230]	9892181	9895430	36.71
	AT2G23240	MT4B	AtMT4b [Source:UniProtKB/TrEMBL;Acc:A0A178VZU5]	9895680	9896347	38.92
	AT2G23250	UGT84B2	UDP-glycosyltransferase 84B2 [Source:UniProtKB/Swiss-Prot;Acc:O22183]	9897809	9899125	45.71
	AT2G23260	UGT84B1	UDP-glycosyltransferase 84B1 [Source:UniProtKB/Swiss-Prot;Acc:O22182]	9899927	9901439	43.82
	AT2G23270		At2g23270 [Source:UniProtKB/TrEMBL;Acc:O22181]	9903215	9903845	36.13
	AT2G23290	AtMYB70	At2g23280 [Source:UniProtKB/TrEMBL;Acc:O22179]	9904506	9906032	43.88
	AT2G23300		Leucine-rich repeat protein kinase family protein [Source:UniProtKB/TrEMBL;Acc:O22178]	9914246	9917427	42.87
	AT2G23310	RER1C	Protein RER1C [Source:UniProtKB/Swiss-Prot;Acc:Q9ZWI7]	9917443	9919379	38.87
	AT2G07895			9919984	9920197	35.05
	AT2G23320	WRKY15	Probable WRKY transcription factor 15 [Source:UniProtKB/Swiss-Prot;Acc:O22176]	9924886	9926372	38.87
	AT2G23321		unknown protein; Ha. [Source:TAIR;Acc:AT2G23321]	9926378	9926518	36.17
	AT2G07900			9932687	9933019	37.84
	AT2G23340	ERF008	Ethylene-responsive transcription factor ERF008 [Source:UniProtKB/Swiss-Prot;Acc:O22174]	9937792	9938997	40.13
	AT2G23348		unknown protein; FUNCTIONS IN: molecular_function unknown; INVOLVED IN: biological_process unknown; LOCATED IN: en	9940742	9942932	35.74
	AT2G23347	MIR844A	MIR844a; miRNA [Source:TAIR;Acc:AT2G23347]	9942151	9942335	38.92
	AT2G23350	PAB4	Polyadenylate-binding protein 4 [Source:UniProtKB/Swiss-Prot;Acc:O22173]	9942915	9946347	40.84
	AT2G23360	FPP7	Filament-like plant protein 7 [Source:UniProtKB/Swiss-Prot;Acc:Q9SLN1]	9948594	9952896	39.16
	AT2G23370		Cyclin delta-3 [Source:UniProtKB/TrEMBL;Acc:Q8RWU1]	9952867	9955149	39.99
	AT2G23380	CLF	Histone-lysine N-methyltransferase CLF [Source:UniProtKB/Swiss-Prot;Acc:P93831]	9955092	9960386	38.81
	AT2G23390		Acyl-CoA [Source:UniProtKB/TrEMBL;Acc:O80456]	9960365	9963646	37.63
	AT2G23400		Undecaprenyl pyrophosphate synthetase family protein [Source:TAIR;Acc:AT2G23400]	9963809	9965414	37.17
	AT2G23410	DPS	Dehydrodolichyl diphosphate synthase 1 [Source:UniProtKB/Swiss-Prot;Acc:O80458]	9966127	9967788	37.06
	AT2G23420	NAPRT2	Nicotinate phosphoribosyltransferase 2 [Source:UniProtKB/Swiss-Prot;Acc:Q84WV8]	9971603	9975207	37.64
	AT2G23430	KRP1	Cyclin-dependent kinase inhibitor 1 [Source:UniProtKB/Swiss-Prot;Acc:Q67Y93]	9976590	9978090	33.04
MQTL-3/Chr2	AT2G26350	PEX10	PEX10 [Source:UniProtKB/TrEMBL;Acc:A0A178VWJ2]	11217142	11220541	37.29
	AT2G26355		other RNA [Source:TAIR;Acc:AT2G26355]	11220708	11221535	38.29
	AT2G26360		Mitochondrial substrate carrier family protein [Source:UniProtKB/TrEMBL;Acc:F4IUJ2]	11221516	11223400	37.35
	AT2G26370		At2g26370 [Source:UniProtKB/TrEMBL;Acc:O48704]	11224565	11226235	32.38
	AT2G26380		Leucine-rich repeat (LRR) family protein [Source:UniProtKB/TrEMBL;Acc:O48705]	11226488	11228123	44.5
	AT2G26390		Serpin-Z3 [Source:UniProtKB/Swiss-Prot;Acc:O48706]	11228825	11230598	39.46
	AT2G26400	ARD	acireductone dioxygenase 3 [Source:TAIR;Acc:AT2G26400]	11231770	11233388	33.66
	AT2G26410	Iqd4	IQ-domain 4 [Source:UniProtKB/TrEMBL;Acc:F4IUJ7]	11234267	11236995	38.07
	AT2G26420	PIP5K3	1-phosphatidylinositol-4-phosphate 5-kinase 3 [Source:TAIR;Acc:AT2G26420]	11238958	11242963	38.57
	AT2G26430	CYCL1-1	Cyclin-L1-1 [Source:UniProtKB/Swiss-Prot;Acc:Q8RWV3]	11243128	11246058	38.18
	AT2G26440	PME12	Probable pectinesterase/pectinesterase inhibitor 12 [Source:UniProtKB/Swiss-Prot;Acc:O48711]	11247160	11249620	39.58
	AT2G26450	PME13	Probable pectinesterase/pectinesterase inhibitor 13 [Source:UniProtKB/Swiss-Prot;Acc:Q7Y201]	11251040	11253612	41.04
	AT2G26460	SMU2	Suppressor of mec-8 and unc-52 protein homolog 2 [Source:UniProtKB/Swiss-Prot;Acc:O48713]	11254932	11259070	37.98
	AT2G26470		At2g26470 [Source:UniProtKB/TrEMBL;Acc:Q8GZ60]	11259142	11262586	36.69
	AT2G26480	UGT76D1	UDP-glycosyltransferase 76D1 [Source:UniProtKB/Swiss-Prot;Acc:O48715]	11263889	11265855	38.79
	AT2G26490	JGB	Protein JINGUBANG [Source:UniProtKB/Swiss-Prot;Acc:O48716]	11267857	11269750	39.49
	AT2G26500		At2g26500/T9J22.17 [Source:UniProtKB/TrEMBL;Acc:O48717]	11270082	11271074	37.97
	AT2G26510	NAT3	Nucleobase-ascorbate transporter 3 [Source:UniProtKB/Swiss-Prot;Acc:Q8GZD4]	11273842	11277783	36.78
	AT2G26515			11277945	11279084	35.79
	AT2G26520		Expressed protein [Source:UniProtKB/TrEMBL;Acc:O48719]	11279913	11280996	37.73
	AT2G26530	AR781	AR781 [Source:UniProtKB/TrEMBL;Acc:A0A178VPT1]	11282663	11284272	39.07
	AT2G26540	UROS	Uroporphyrinogen-III synthase, chloroplastic [Source:UniProtKB/Swiss-Prot;Acc:O48721]	11287308	11290361	37.23
	AT2G26550	HO2	Heme oxygenase 2 [Source:UniProtKB/TrEMBL;Acc:F4IUM0]	11291148	11293575	37.85
	AT2G26560	PLP2	Patatin-like protein 2 [Source:UniProtKB/Swiss-Prot;Acc:O48723]	11293702	11295918	35.99
	AT2G26570	WEB1	Protein WEAK CHLOROPLAST MOVEMENT UNDER BLUE LIGHT 1 [Source:UniProtKB/Swiss-Prot;Acc:O48724]	11298914	11302319	40.22
	AT2G26580	YAB5	Axial regulator YABBY 5 [Source:UniProtKB/Swiss-Prot;Acc:Q8GW46]	11303455	11307010	30.57
	AT2G26590	RPN13	RPN13 [Source:UniProtKB/TrEMBL;Acc:A0A178VY31]	11310957	11315524	36.41
	AT2G26600		Glycosyl hydrolase superfamily protein [Source:UniProtKB/TrEMBL;Acc:O48727]	11316346	11318520	36.83
	AT2G26610		Transducin family protein / WD-40 repeat family protein [Source:TAIR;Acc:AT2G26610]	11318771	11324213	41.25
	AT2G26620		Pectin lyase-like superfamily protein [Source:UniProtKB/TrEMBL;Acc:O48729]	11325069	11326835	34.69
	AT2G26640	KCS11	3-ketoacyl-CoA synthase 11 [Source:UniProtKB/Swiss-Prot;Acc:O48780]	11329719	11331858	40
	AT2G26650	AKT1	Potassium channel AKT1 [Source:UniProtKB/Swiss-Prot;Acc:Q38998]	11331850	11336738	37.39
	AT2G26660	SPX2	SPX domain-containing protein 2 [Source:UniProtKB/Swiss-Prot;Acc:O48781]	11338752	11340950	35.43
	AT2G26670	HO1	TED4 [Source:UniProtKB/TrEMBL;Acc:A0A178VP48]	11341548	11343693	36.07
	AT2G26680		Expressed protein [Source:UniProtKB/TrEMBL;Acc:O48783]	11343766	11345540	38.82
	AT2G26690	NPF6.2	Protein NRT1/ PTR FAMILY 6.2 [Source:UniProtKB/Swiss-Prot;Acc:Q9SZY4]	11347147	11350993	35.09
	AT2G26692		other RNA [Source:TAIR;Acc:AT2G26692]	11350517	11351293	33.59
	AT2G08150			11359186	11359800	40.33
	AT2G08155			11360496	11360698	34.48
	AT2G08160			11361270	11361518	32.93
	AT2G08165			11362197	11362426	36.52
	AT2G26695		At2g26695 [Source:UniProtKB/TrEMBL;Acc:Q6ID73]	11364996	11366030	37.87
	AT2G26700	PID2	Protein kinase PINOID 2 [Source:UniProtKB/Swiss-Prot;Acc:Q64FQ2]	11368474	11371124	37.16
	AT2G26710	CYP734A1	CYP734A1 [Source:UniProtKB/TrEMBL;Acc:A0A178VRF1]	11380463	11383821	36.17
	AT2G26720		Cupredoxin superfamily protein [Source:UniProtKB/TrEMBL;Acc:O48787]	11384782	11385402	44.28
	AT2G26730		Probable inactive receptor kinase At2g26730 [Source:UniProtKB/Swiss-Prot;Acc:O48788]	11388298	11391682	39.03
	AT2G26740	ATSEH	At2g26740/F18A8.11 [Source:UniProtKB/TrEMBL;Acc:Q42566]	11392849	11394363	42.18
	AT2G26750		Alpha/beta-Hydrolases superfamily protein [Source:UniProtKB/TrEMBL;Acc:O48789]	11395707	11397219	40.32
	AT2G26760	CYCB1-4	CYCB1 [Source:UniProtKB/TrEMBL;Acc:A0A178VPU5]	11401118	11403433	38.99
	AT2G26770	SCAB1	Stomatal closure-related actin-binding protein 1 [Source:UniProtKB/Swiss-Prot;Acc:O48791]	11403979	11408421	36.15
	AT2G08175			11408804	11409308	35.64
	AT2G26780		ARM repeat superfamily protein [Source:UniProtKB/TrEMBL;Acc:F4IVJ8]	11409828	11423776	37.12
	AT2G08180			11409844	11410075	43.97
	AT2G26790		Pentatricopeptide repeat-containing protein At2g26790, mitochondrial [Source:UniProtKB/Swiss-Prot;Acc:O81028]	11424163	11427792	39.12
	AT2G26800	HMGCL	Hydroxymethylglutaryl-CoA lyase, mitochondrial [Source:UniProtKB/Swiss-Prot;Acc:O81027]	11429019	11432797	37.63
	AT2G26810		Putative methyltransferase family protein [Source:UniProtKB/TrEMBL;Acc:F4IVK3]	11433662	11436300	35.47
	AT2G26820	ATPP2-A3	phloem protein 2-A3 [Source:TAIR;Acc:AT2G26820]	11437689	11442379	33.15
	AT2G26830	EMB1187	Probable ethanolamine kinase [Source:UniProtKB/Swiss-Prot;Acc:O81024]	11443241	11447171	35.82
	AT2G26840		unknown protein; FUNCTIONS IN: molecular_function unknown; INVOLVED IN: biological_process unknown; LOCATED IN: chl	11447169	11448949	37.17
	AT2G26850		F-box protein At2g26850 [Source:UniProtKB/Swiss-Prot;Acc:Q6NLB1]	11448976	11451270	36.38
	AT2G26860		FBD-associated F-box protein At2g26860 [Source:UniProtKB/Swiss-Prot;Acc:Q84X02]	11451950	11453947	35.94
	AT2G26865		Encodes a Plant thionin family protein [Source:TAIR;Acc:AT2G26865]	11455540	11455869	34.85
	AT2G26870	NPC2	Non-specific phospholipase C2 [Source:UniProtKB/Swiss-Prot;Acc:O81020]	11456926	11459481	37.6
	AT2G26880	AGL41	AGL41 [Source:UniProtKB/TrEMBL;Acc:A0A178VRS6]	11459906	11461099	35.43
	AT2G26882		other RNA [Source:TAIR;Acc:AT2G26882]	11460219	11461021	38.36
	AT2G26890	GRV2	DnaJ homolog subfamily C GRV2 [Source:UniProtKB/Swiss-Prot;Acc:F4IVL6]	11462067	11474371	41.06
	AT2G26900	BASS2	BASS2 [Source:UniProtKB/TrEMBL;Acc:A0A178VXV9]	11474752	11478142	37.19
	AT2G26910	ABCG32	PEC1 [Source:UniProtKB/TrEMBL;Acc:A0A178VNA5]	11481326	11488270	39.01
	AT2G26920		At2g26920 [Source:UniProtKB/TrEMBL;Acc:O81015]	11488988	11491751	40.88
	AT2G26930	ISPE	PDE277 [Source:UniProtKB/TrEMBL;Acc:A0A178VZZ2]	11491707	11494290	37.69
	AT2G26940		C2H2-type zinc finger family protein [Source:UniProtKB/TrEMBL;Acc:O81013]	11496518	11497702	39.24
	AT2G26950	MYB104	Transcription factor MYB104 [Source:UniProtKB/Swiss-Prot;Acc:Q9SM27]	11500721	11502136	39.19

AT2G26960	AtMYB81	Myb domain protein 81 [Source:UniProtKB/TrEMBL;Acc:Q9SLH1]	11506065	11507426	42.07
AT2G08190			11509082	11509384	34.98
AT2G26970		Oligoribonuclease [Source:UniProtKB/Swiss-Prot;Acc:Q9ZVE0]	11509721	11511977	36.11
AT2G26975	COPT6	Copper transporter 6 [Source:UniProtKB/Swiss-Prot;Acc:Q8GWP3]	11512727	11513695	39.01
AT2G26980	CIPK3	Non-specific serine/threonine protein kinase [Source:UniProtKB/TrEMBL;Acc:F4IVM7]	11514600	11518734	35.41
AT2G26990	CSN2	COP9 signalosome complex subunit 2 [Source:UniProtKB/Swiss-Prot;Acc:Q8W207]	11519388	11522616	37.53
AT2G27000	CYP705A8	CYP705A8 [Source:UniProtKB/TrEMBL;Acc:A0A178W1J9]	11523287	11525140	40.4
AT2G27010	CYP705A9	Cytochrome P450, family 705, subfamily A, polypeptide 9 [Source:UniProtKB/TrEMBL;Acc:Q9ZVD6]	11526168	11527854	43.39
AT2G27020	PAG1	20S proteasome alpha subunit G1 [Source:TAIR;Acc:AT2G27020]	11528230	11531178	37
AT2G27030	CAM5	Calmodulin 5 [Source:UniProtKB/TrEMBL;Acc:F4IVN6]	11531967	11534358	30.39
AT2G27035	ENODL20	Early nodulin-like protein 20 [Source:UniProtKB/TrEMBL;Acc:F4IVN9]	11535518	11536485	36.47
AT2G27040	AGO4	Protein argonaute 4 [Source:UniProtKB/Swiss-Prot;Acc:Q9ZVD5]	11536502	11542391	38.42
AT2G27050	EIL1	ETHYLENE INSENSITIVE 3-like 1 protein [Source:UniProtKB/Swiss-Prot;Acc:Q9SLH0]	11545656	11548293	40.79
AT2G27060		Leucine-rich repeat protein kinase family protein [Source:UniProtKB/TrEMBL;Acc:F4IVP3]	11550705	11554775	40.19
AT2G27070	ARR13	Putative two-component response regulator ARR13 [Source:UniProtKB/Swiss-Prot;Acc:Q9ZVD3]	11556392	11560215	32.58
AT2G27080	NHL13	NDR1/HIN1-like protein 13 [Source:UniProtKB/Swiss-Prot;Acc:Q9ZVD2]	11563933	11567504	30.99
AT2G27090		At2g27090 [Source:UniProtKB/TrEMBL;Acc:Q9ZVD1]	11567436	11571903	39.1
AT2G27100	SE	SE [Source:UniProtKB/TrEMBL;Acc:A0A178VZD4]	11572397	11576658	41.83
AT2G27110	FRS3	Protein FAR1-RELATED SEQUENCE 3 [Source:UniProtKB/Swiss-Prot;Acc:Q9ZVC9]	11576668	11580817	40.41
AT2G27120	POL2B	DNA polymerase epsilon catalytic subunit [Source:TAIR;Acc:AT2G27120]	11581214	11594397	37.95
AT2G27130	XYP11	Xylogen-like protein 11 [Source:UniProtKB/Swiss-Prot;Acc:Q9ZVC7]	11595076	11596548	35.57
AT2G27140		At2g27140 [Source:UniProtKB/TrEMBL;Acc:Q9ZVC6]	11598305	11599322	38.9
AT2G27145	LCR9	Putative defensin-like protein 146 [Source:UniProtKB/Swiss-Prot;Acc:P82724]	11600169	11600764	30.54
AT2G27150	AAO3	Abscisic-aldehyde oxidase [Source:UniProtKB/Swiss-Prot;Acc:Q7G9P4]	11601727	11608450	39.29
AT2G27160		unknown protein; Ha. [Source:TAIR;Acc:AT2G27160]	11607432	11608416	40
AT2G27170	SMC3	Structural maintenance of chromosomes protein 3 [Source:UniProtKB/Swiss-Prot;Acc:Q56YN8]	11609073	11617496	36.79
AT2G27180		Expressed protein [Source:UniProtKB/TrEMBL;Acc:Q9SHT0]	11620076	11621174	39.13
AT2G27190	PAP12	Fe(3+)-Zn(2+) purple acid phosphatase 12 [Source:UniProtKB/Swiss-Prot;Acc:Q38924]	11621222	11623618	37.46
AT2G27200	LSG1-1	GTPase LSG1-1 [Source:UniProtKB/Swiss-Prot;Acc:Q9SHS8]	11625157	11628290	38.9
AT2G27210	BSL3	Serine/threonine-protein phosphatase BSL3 [Source:UniProtKB/Swiss-Prot;Acc:Q9SHS7]	11629777	11636803	39.59
AT2G27220	BLH5	BEL1-like homeodomain 5 [Source:TAIR;Acc:AT2G27220]	11637106	11639809	34.84
AT2G27229		Pentatricopeptide repeat (PPR) superfamily protein [Source:UniProtKB/TrEMBL;Acc:Q1G3C0]	11649057	11649998	33.12
AT2G27230	LHW	LHW [Source:UniProtKB/TrEMBL;Acc:A0A178VXD3]	11650358	11654143	37.96
AT2G27240	ALMT7	Aluminum-activated malate transporter 7 [Source:UniProtKB/Swiss-Prot;Acc:Q9XIN1]	11659579	11662408	35.27
AT2G27250	CLV3	Protein CLAVATA 3 [Source:UniProtKB/Swiss-Prot;Acc:Q9XF04]	11664978	11665797	36.71
AT2G27260		At2g27260/F12K2.16 [Source:UniProtKB/TrEMBL;Acc:Q9XIN3]	11669629	11670644	40.85
AT2G27270		Transmembrane protein [Source:UniProtKB/TrEMBL;Acc:Q9XIN4]	11672271	11673208	40.83
AT2G27280		Coiled-coil protein (DUF2040) [Source:UniProtKB/TrEMBL;Acc:Q9XIN5]	11673826	11675613	39.32
AT2G27285		Expressed protein [Source:UniProtKB/TrEMBL;Acc:Q8S8I5]	11675814	11678118	38.22
AT2G27290		At2g27290 [Source:UniProtKB/TrEMBL;Acc:Q9XIN6]	11678305	11679919	36.16
AT2G27300	NTL8	NAC domain-containing protein 40 [Source:UniProtKB/Swiss-Prot;Acc:Q9XIN7]	11680276	11681971	39.15
AT2G27310		F-box protein At2g27310 [Source:UniProtKB/Swiss-Prot;Acc:Q9XIN8]	11683772	11685342	39.4
AT2G27313			11688938	11689141	41.67
AT2G27315		Protein of unknown function (DUF1278) [Source:TAIR;Acc:AT2G27315]	11689467	11690004	38.66
AT2G27320		NEP-interacting protein, putative (DUF239) [Source:UniProtKB/TrEMBL;Acc:Q5BPS8]	11692884	11694916	30.15
AT2G27330		At2g27330 [Source:UniProtKB/TrEMBL;Acc:Q1ECN0]	11695032	11696799	37.95
AT2G27340		At2g27340 [Source:UniProtKB/TrEMBL;Acc:Q6NLZ3]	11696595	11699224	35.63
AT2G27350		OTU-containing deubiquitinating enzyme OTU6 [Source:UniProtKB/TrEMBL;Acc:F4IFS7]	11699548	11704303	38.1
AT2G27360		GDSL esterase/lipase At2g27360 [Source:UniProtKB/Swiss-Prot;Acc:Q9ZQI3]	11706028	11708139	36.41
AT2G27370	CASP3	Casparian strip membrane protein 3 [Source:UniProtKB/Swiss-Prot;Acc:Q9ZQI2]	11708429	11710128	37.47
AT2G27380	EPR1	Proline-rich extensin-like protein EPR1 [Source:UniProtKB/Swiss-Prot;Acc:Q9ZQI0]	11713411	11715774	49.07
AT2G27385		Pollen Ole e 1 allergen and extensin family protein [Source:UniProtKB/TrEMBL;Acc:F4IFT4]	11716001	11717437	36.33
AT2G08195			11719064	11719337	40.15
AT2G08200			11719102	11719337	43.22
AT2G27389		unknown protein; LOCATED IN: endomembrane system. [Source:TAIR;Acc:AT2G27389]	11720294	11721081	43.78
AT2G27390		At2g27390 [Source:UniProtKB/TrEMBL;Acc:Q9XIP3]	11720484	11720888	54.07
AT2G27400	TAS1A	TAS1A; other RNA [Source:TAIR;Acc:AT2G27400]	11721539	11722468	33.76
AT2G27402		At2g27402 [Source:UniProtKB/TrEMBL;Acc:Q8GX30]	11722770	11724400	37.83
AT2G27410		Putative B3 domain-containing protein At2g27410 [Source:UniProtKB/Swiss-Prot;Acc:Q9XIP5]	11724427	11725419	41.79
AT2G27420		Cysteine proteinases superfamily protein [Source:UniProtKB/TrEMBL;Acc:Q9ZQH7]	11726134	11727654	39.84
AT2G27430		ARM repeat superfamily protein [Source:UniProtKB/TrEMBL;Acc:Q8GUS7]	11729759	11733371	32.91
AT2G27450	CPA	N-carbamoylputrescine amidase [Source:UniProtKB/Swiss-Prot;Acc:Q8VYF5]	11737374	11739690	37.72
AT2G27460		At2g27460 [Source:UniProtKB/TrEMBL;Acc:Q9ZQH3]	11740502	11745011	38.78
AT2G27470	NF-YB11	Nuclear factor Y, subunit B11 [Source:UniProtKB/TrEMBL;Acc:Q9ZQH2]	11744981	11746455	41.63
AT2G08205			11746004	11746186	31.69
AT2G27480	CML48	Probable calcium-binding protein CML48 [Source:UniProtKB/Swiss-Prot;Acc:Q9ZQH1]	11746683	11748051	40.25
AT2G27490	COAE	Dephospho-CoA kinase [Source:UniProtKB/Swiss-Prot;Acc:Q9ZQH0]	11748015	11750488	35.93
AT2G27500		Glucan endo-1,3-beta-glucosidase 14 [Source:UniProtKB/Swiss-Prot;Acc:Q9ZQG9]	11752064	11754066	35
AT2G27505		FBD-like domain family protein [Source:UniProtKB/TrEMBL;Acc:F4IGN8]	11756566	11757898	39.31
AT2G27507			11756694	11756949	44.53
AT2G27510	FD3	Ferredoxin [Source:UniProtKB/TrEMBL;Acc:A0A178VWS0]	11757955	11759588	38
AT2G27520		F-box/LRR-repeat/kelch-repeat protein At2g27520 [Source:UniProtKB/Swiss-Prot;Acc:Q9ZNQ3]	11762061	11763143	38.32
AT2G27530	RPL10AB	60S ribosomal protein L10a-2 [Source:UniProtKB/Swiss-Prot;Acc:P59230]	11763217	11764935	36.88
AT2G27535		Ribosomal protein L10A family protein [Source:UniProtKB/TrEMBL;Acc:Q8VYN3]	11765281	11766144	33.33
AT2G27540		Putative uncharacterized protein At2g27540 [Source:UniProtKB/TrEMBL;Acc:Q9ZUW2]	11768951	11769100	53.33
AT2G27550	CEN	Protein CENTRORADIALIS-like [Source:UniProtKB/Swiss-Prot;Acc:Q9ZNV5]	11773187	11774746	36.03
AT2G27560		pre-tRNA [Source:TAIR;Acc:AT2G27560]	11774852	11774923	62.5
AT2G27570	SOT4	Cytosolic sulfotransferase 4 [Source:UniProtKB/Swiss-Prot;Acc:Q8RUC1]	11775329	11776305	39.71
AT2G27580	SAP3	Zinc finger A20 and AN1 domain-containing stress-associated protein 3 [Source:UniProtKB/Swiss-Prot;Acc:Q9ZNU9]	11776410	11777553	40.12
AT2G27590		Expressed protein [Source:UniProtKB/TrEMBL;Acc:Q8VYF0]	11777554	11779912	39.08
AT2G27600	SKD1	Protein SUPPRESSOR OF K(+) TRANSPORT GROWTH DEFECT 1 [Source:UniProtKB/Swiss-Prot;Acc:Q9ZNT0]	11780896	11783992	39.04
AT2G27610	PCMP-H60	Pentatricopeptide repeat-containing protein At2g27610 [Source:UniProtKB/Swiss-Prot;Acc:Q9ZUW3]	11783927	11786533	41.35
AT2G27630		Ubiquitin carboxyl-terminal hydrolase-related protein [Source:UniProtKB/TrEMBL;Acc:F4IGQ2]	11787398	11792646	38.65
AT2G27650		Ubiquitin carboxyl-terminal hydrolase-related protein [Source:UniProtKB/TrEMBL;Acc:F4IGQ3]	11792825	11797712	38.48
AT2G27660		Cysteine/Histidine-rich C1 domain family protein [Source:UniProtKB/TrEMBL;Acc:Q9ZUW8]	11798417	11800928	44.23
AT2G27670		Domain of unknown function DUF220 [Source:TAIR;Acc:AT2G27670]	11801568	11802891	36.93
AT2G27680		At2g27680/F15K20.22 [Source:UniProtKB/TrEMBL;Acc:Q9ZUX0]	11803743	11806087	39.4
AT2G27690	CYP94C1	Cytochrome P450 94C1 [Source:UniProtKB/Swiss-Prot;Acc:Q9ZUX1]	11809279	11811280	41.26
AT2G27700		Eukaryotic translation initiation factor 2 family protein / eIF-2 family protein [Source:UniProtKB/TrEMBL;Acc:F4IGQ9]	11813949	11816002	36.81
AT2G27710	RPP2B	AT2G27710 protein [Source:UniProtKB/TrEMBL;Acc:B9DGN3]	11816634	11817972	38.61
AT2G27720		60S acidic ribosomal protein family [Source:UniProtKB/TrEMBL;Acc:F4IGR4]	11818230	11819646	38.6
AT2G27730		Uncharacterized protein At2g27730, mitochondrial [Source:UniProtKB/Swiss-Prot;Acc:Q9ZUX4]	11819728	11822036	36.03
AT2G27740		At2g27740 [Source:UniProtKB/TrEMBL;Acc:Q9ZUX5]	11822513	11823857	36.65
AT2G27750		Nucleolar matrix protein-related [Source:UniProtKB/TrEMBL;Acc:Q9ZUX6]	11823916	11824461	40.11
AT2G27760	IPT2	tRNA dimethylallyltransferase 2 [Source:UniProtKB/Swiss-Prot;Acc:Q9ZUX7]	11824772	11827609	37.81
AT2G08210			11829756	11830015	34.23
AT2G27770		At2g27770 [Source:UniProtKB/TrEMBL;Acc:Q9ZUX8]	11832543	11834427	34.32
AT2G27775		At2g27775 [Source:UniProtKB/TrEMBL;Acc:B1WCA4]	11842236	11843361	38.63
AT2G27780		Transcription factor IIS family protein [Source:UniProtKB/TrEMBL;Acc:Q9ZUX9]	11845472	11846578	42.37
AT2G27790		RNA-binding (RRM/RBD/RNP motifs) family protein [Source:UniProtKB/TrEMBL;Acc:F4IGS5]	11847109	11849559	35.21
AT2G27800		Pentatricopeptide repeat-containing protein At2g27800, mitochondrial [Source:UniProtKB/Swiss-Prot;Acc:Q9ZUY1]	11849647	11851457	38.6
AT2G27810	ATNAT12	nucleobase-ascorbate transporter 12 [Source:TAIR;Acc:AT2G27810]	11852078	11856335	39.17
AT2G27820	ADT3	Arogenate dehydratase 3, chloroplastic [Source:UniProtKB/Swiss-Prot;Acc:Q9ZUY3]	11856571	11858288	43.89
AT2G27830		Expressed protein [Source:UniProtKB/TrEMBL;Acc:Q9ZUY4]	11860218	11861525	38.61
AT2G27840	HDT4	Histone deacetylase HDT4 [Source:UniProtKB/Swiss-Prot;Acc:Q9M4T3]	11861806	11863908	33.38

	AT2G27850		pre-tRNA [Source:TAIR;Acc:AT2G27850]	11864154	11864225	55.56
	AT2G27860	AXS1	UDP-D-apiose/UDP-D-xylose synthase 1 [Source:UniProtKB/Swiss-Prot;Acc:Q9ZUY6]	11864277	11866998	39.2
	AT2G27880	AGO5	Protein argonaute 5 [Source:UniProtKB/Swiss-Prot;Acc:Q9SJK3]	11871378	11876989	38.92
	AT2G27900		At2g27890 [Source:UniProtKB/TrEMBL;Acc:Q8RXC2]	11877421	11886013	36.8
	AT2G27920	SCPL51	Serine carboxypeptidase-like 51 [Source:UniProtKB/Swiss-Prot;Acc:Q67Y83]	11885571	11889176	30.92
	AT2G27930		PLATZ transcription factor family protein [Source:TAIR;Acc:AT2G27930]	11891998	11893787	34.53
	AT2G27940	ATL57	RING-H2 finger protein ATL57 [Source:UniProtKB/Swiss-Prot;Acc:Q9SJJ7]	11897942	11898867	45.57
	AT2G27950		Ring/U-Box superfamily protein [Source:UniProtKB/TrEMBL;Acc:F4IHN6]	11899160	11904062	41.24
	AT2G27960	CKS1	Cyclin-dependent kinases regulatory subunit [Source:UniProtKB/TrEMBL;Acc:A0A178VPG9]	11910569	11911802	37.12
	AT2G27970	CKS2	Cyclin-dependent kinases regulatory subunit 2 [Source:UniProtKB/Swiss-Prot;Acc:Q9SJJ5]	11912151	11913425	36.71
	AT2G27980		Acyl-CoA N-acyltransferase with RING/FYVE/PHD-type zinc finger domain-containing protein [Source:UniProtKB/TrEMBL;Acc:F	11913359	11920001	38.61
	AT2G27990	BLH8	BEL1-like homeodomain protein 8 [Source:UniProtKB/Swiss-Prot;Acc:Q9SJJ3]	11921350	11924748	37.13
	AT2G28000	CPN60A1	SLP [Source:UniProtKB/TrEMBL;Acc:A0A178VZW6]	11926446	11929488	40.88
	AT2G28010		Eukaryotic aspartyl protease family protein [Source:UniProtKB/TrEMBL;Acc:Q9SJJ2]	11930494	11931813	43.48
	AT2G28020		Mediator of RNA polymerase II transcription subunit [Source:UniProtKB/TrEMBL;Acc:Q8S8F0]	11933702	11934060	39.55
	AT2G28030		Eukaryotic aspartyl protease family protein [Source:UniProtKB/TrEMBL;Acc:Q9ZUU5]	11934208	11935386	44.02
	AT2G28040		At2g28040 [Source:UniProtKB/TrEMBL;Acc:Q84WH0]	11936030	11937977	37.27
	AT2G28050		Pentatricopeptide repeat-containing protein At2g28050 [Source:UniProtKB/Swiss-Prot;Acc:Q9ZUU7]	11937871	11939653	38.75
	AT2G08215			11941296	11941533	30.25
	AT2G28056	MIR172A	MIR172/MIR172A; miRNA [Source:TAIR;Acc:AT2G28056]	11942914	11943015	44.12
	AT2G28060	KINB3	SNF1-related protein kinase regulatory subunit beta-3 [Source:UniProtKB/Swiss-Prot;Acc:Q9ZUU8]	11949871	11952253	36.38
	AT2G08220			11953652	11955213	34.76
	AT2G08230			11954741	11954995	36.47
	AT2G28070	ABCG3	ABC transporter G family member 3 [Source:UniProtKB/Swiss-Prot;Acc:Q9ZUU9]	11955873	11959994	37.58
	AT2G28080	UGT86A2	Glycosyltransferase (Fragment) [Source:UniProtKB/TrEMBL;Acc:W8Q3F0]	11960571	11963440	34.6
	AT2G28085		Auxin-responsive family protein [Source:UniProtKB/TrEMBL;Acc:Q8S8L5]	11967942	11968712	37.74
	AT2G28090	HIPP01	Heavy metal-associated isoprenylated plant protein 1 [Source:UniProtKB/Swiss-Prot;Acc:Q9ZUV1]	11972140	11974007	38.01
	AT2G28100	FUC1	Alpha-L-fucosidase 1 [Source:UniProtKB/Swiss-Prot;Acc:Q8GW72]	11974663	11976600	42.26
	AT2G08235			11974711	11974881	36.26
	AT2G28105		Replication factor-A carboxy-terminal domain protein [Source:UniProtKB/TrEMBL;Acc:Q5XVD2]	11976303	11977913	36.5
	AT2G28110	IRX7	Glycosyltransferase (Fragment) [Source:UniProtKB/TrEMBL;Acc:W8Q712]	11977865	11980003	38.1
	AT2G28120		At2g28120/F24D13.9 [Source:UniProtKB/TrEMBL;Acc:Q9ZUV4]	11985687	11987836	41.49
	AT2G28130		Actin protein 2/3 complex subunit-like protein [Source:UniProtKB/TrEMBL;Acc:Q0V7S1]	11988358	11991617	34.97
	AT2G28140		Enabled-like protein (DUF1635) [Source:UniProtKB/TrEMBL;Acc:Q9ZUV6]	11994954	11996236	40.92
	AT2G28150		UPSTREAM OF FLC protein (DUF966) [Source:UniProtKB/TrEMBL;Acc:Q8GYT8]	11999736	12002738	38.06
	AT2G28160	FIT	Transcription factor FER-LIKE IRON DEFICIENCY-INDUCED TRANSCRIPTION FACTOR [Source:UniProtKB/Swiss-Prot;Acc:(	12004658	12006276	38.17
	AT2G28170	CHX7	Cation/H(+) antiporter 7 [Source:UniProtKB/Swiss-Prot;Acc:Q9ZUV9]	12006371	12009956	37.79
	AT2G08240			12009870	12010152	37.46
	AT2G28180	CHX8	Cation/H(+) antiporter 8 [Source:UniProtKB/Swiss-Prot;Acc:Q58P71]	12010994	12013739	41.73
	AT2G28190	CSD2	Superoxide dismutase [Cu-Zn] 2, chloroplastic [Source:UniProtKB/Swiss-Prot;Acc:O78310]	12014412	12016569	38.69
	AT2G28200	ZAT5	Zinc finger protein ZAT5 [Source:UniProtKB/Swiss-Prot;Acc:Q681X4]	12023940	12025370	40.74
	AT2G08245			12027275	12027767	43.2
	AT2G08250			12027424	12027798	42.4
	AT2G28210	ATACA2	ATACA2 [Source:UniProtKB/TrEMBL;Acc:A0A178VW72]	12029666	12033096	28.07
	AT2G28220		Eukaryotic aspartyl protease family protein [Source:TAIR;Acc:AT2G28220]	12033998	12035327	43.16
	AT2G28225		Aspartic proteinase CDR1-like protein [Source:UniProtKB/TrEMBL;Acc:A0A1P8B0H2]	12036273	12037527	43.59
	AT2G28230	MED20A	Mediator of RNA polymerase II transcription subunit 20a [Source:UniProtKB/Swiss-Prot;Acc:Q6NPF4]	12037627	12039059	36.01
	AT2G28240		ATP-dependent helicase family protein [Source:UniProtKB/TrEMBL;Acc:Q84JK0]	12039208	12042175	42.18
	AT2G28250	NCRK	NCRK [Source:UniProtKB/TrEMBL;Acc:A0A178VW29]	12042348	12046616	37.5
	AT2G08255			12042707	12043103	39.55
	AT2G28260	CNGC15	Putative cyclic nucleotide-gated ion channel 15 [Source:UniProtKB/Swiss-Prot;Acc:Q9SL29]	12049443	12052643	37.71
	AT2G08260			12049444	12049677	35.9
	AT2G28270		Cysteine/Histidine-rich C1 domain family protein [Source:UniProtKB/TrEMBL;Acc:Q9SL28]	12053542	12054538	41.32
	AT2G28290	SYD	SYD [Source:UniProtKB/TrEMBL;Acc:A0A384L3H2]	12056213	12073279	39.73
	AT2G28305	LOG1	Cytokinin riboside 5'-monophosphate phosphoribohydrolase [Source:UniProtKB/TrEMBL;Acc:A0A178VS96]	12081023	12084678	30.28
	AT2G28310		Trimethylguanosine synthase (DUF707) [Source:UniProtKB/TrEMBL;Acc:Q56Y17]	12085356	12088121	35.79
	AT2G28315	UXT1	UDP-xylose transporter 1 [Source:UniProtKB/Swiss-Prot;Acc:F4IHS9]	12088667	12090765	35.68
	AT2G28320		Pleckstrin homology (PH) and lipid-binding START domains-containing protein [Source:UniProtKB/TrEMBL;Acc:F4IHT0]	12094621	12099644	37.66
	AT2G28330	SMR11	Cyclin-dependent protein kinase inhibitor SMR11 [Source:UniProtKB/Swiss-Prot;Acc:Q9SKN7]	12099742	12100757	37.89
	AT2G28340	GATA13	Putative GATA transcription factor 13 [Source:UniProtKB/Swiss-Prot;Acc:Q9SKN6]	12103672	12106262	36.13
MQTL-4/Chr2	AT2G42350	ATL40	RING-H2 finger protein ATL40 [Source:UniProtKB/Swiss-Prot;Acc:Q9SLC4]	17639001	17640300	40
	AT2G42360	ATL41	E3 ubiquitin-protein ligase ATL41 [Source:UniProtKB/Swiss-Prot;Acc:Q9SLC3]	17640546	17641790	41.69
	AT2G42365		other RNA [Source:TAIR;Acc:AT2G42365]	17640621	17645998	39.75
	AT2G42370		Uncharacterized protein At2g42370 [Source:UniProtKB/TrEMBL;Acc:Q9SLC2]	17643334	17645533	40.14
	AT2G42380	BZIP34	Basic leucine zipper 34 [Source:UniProtKB/Swiss-Prot;Acc:F4IN23]	17646857	17649062	35.9
	AT2G42388		other RNA [Source:TAIR;Acc:AT2G42388]	17650225	17651083	37.49
	AT2G42390		Expressed protein [Source:UniProtKB/TrEMBL;Acc:Q9SLC0]	17651106	17652643	38.75
	AT2G42395		At2g42395 [Source:UniProtKB/TrEMBL;Acc:Q84TF7]	17653080	17654069	37.37
	AT2G42400	VOZ2	VOZ2 [Source:UniProtKB/TrEMBL;Acc:A0A178VSV5]	17654170	17656863	40.61
	AT2G09375			17657354	17657732	34.56
	AT2G42410	ZFP11	zinc finger protein 11 [Source:TAIR;Acc:AT2G42410]	17657824	17658976	39.03
	AT2G42420		pre-tRNA [Source:TAIR;Acc:AT2G42420]	17660778	17660848	57.75
	AT2G42425		Unknown gene [Source:TAIR;Acc:AT2G42425]	17661834	17662358	36
	AT2G42430	LBD16	LOB domain-containing protein 16 [Source:UniProtKB/Swiss-Prot;Acc:Q9SLB7]	17663353	17665337	36.88
	AT2G42440	LBD17	LOB domain-containing protein 17 [Source:UniProtKB/Swiss-Prot;Acc:Q9SLB6]	17668701	17669836	37.68
	AT2G42450		Alpha/beta-Hydrolases superfamily protein [Source:UniProtKB/TrEMBL;Acc:F4IN30]	17672102	17675100	38.45
	AT2G42455		MATH domain/coiled-coil protein [Source:UniProtKB/TrEMBL;Acc:A0A1P8B135]	17676399	17676737	44.54
	AT2G42460		MATH domain and coiled-coil domain-containing protein At2g42460 [Source:UniProtKB/Swiss-Prot;Acc:F4IN32]	17677876	17679293	37.66
	AT2G42465		MATH domain and coiled-coil domain-containing protein At2g42465 [Source:UniProtKB/Swiss-Prot;Acc:P0DKG4]	17679869	17681456	37.78
	AT2G42470		MATH domain and coiled-coil domain-containing protein At2g42470 [Source:UniProtKB/Swiss-Prot;Acc:P0DKG5]	17683865	17685112	38.46
	AT2G42475		MATH domain and coiled-coil domain-containing protein At2g42475 [Source:UniProtKB/Swiss-Prot;Acc:P0DKG6]	17685737	17687146	36.81
	AT2G42480		MATH domain and coiled-coil domain-containing protein At2g42480 [Source:UniProtKB/Swiss-Prot;Acc:P0DKG7]	17688010	17689922	37.85
	AT2G42485		other RNA [Source:TAIR;Acc:AT2G42485]	17690531	17691018	42.21
	AT2G42490		Amine oxidase [Source:UniProtKB/TrEMBL;Acc:Q8L866]	17691407	17696023	40.81
	AT2G42500	PP2A3	Serine/threonine-protein phosphatase PP2A-3 catalytic subunit [Source:UniProtKB/Swiss-Prot;Acc:Q07100]	17697626	17701417	37.32
	AT2G42510		FUNCTIONS IN: molecular_function unknown; INVOLVED IN: spliceosome assembly, nuclear mRNA splicing, via spliceosome; I	17701381	17704690	40.18
	AT2G42520	RH37	DEAD-box ATP-dependent RNA helicase 37 [Source:UniProtKB/Swiss-Prot;Acc:Q84W89]	17704741	17709060	40.23
	AT2G42530	COR15B	Protein COLD-REGULATED 15B, chloroplastic [Source:UniProtKB/Swiss-Prot;Acc:Q9SIN5]	17708593	17710433	36.61
	AT2G42540	COR15A	Protein COLD-REGULATED 15A, chloroplastic [Source:UniProtKB/Swiss-Prot;Acc:Q42512]	17710441	17712330	35.4
	AT2G42550		Protein kinase superfamily protein [Source:UniProtKB/TrEMBL;Acc:Q9SIN4]	17713196	17714230	43.96
	AT2G42560		Late embryogenesis abundant domain-containing protein / LEA domain-containing protein [Source:UniProtKB/TrEMBL;Acc:Q9SIN	17714638	17716912	47.03
	AT2G42570	TBL39	Protein trichome birefringence-like 39 [Source:UniProtKB/Swiss-Prot;Acc:Q9SIN2]	17717332	17720366	34.7
	AT2G42580	TTL3	Inactive TPR repeat-containing thioredoxin TTL3 [Source:UniProtKB/Swiss-Prot;Acc:Q9SIN1]	17728479	17731836	41.81
	AT2G42590	GRF9	General regulatory factor 9 [Source:UniProtKB/TrEMBL;Acc:F4IP55]	17731830	17733982	38.23
	AT2G42600	PPC2	PPC2 [Source:UniProtKB/TrEMBL;Acc:A0A178VV64]	17734302	17739167	41.04
	AT2G42610	LSH10	LSH10 [Source:UniProtKB/TrEMBL;Acc:A0A178VLX9]	17747781	17750524	35.28
	AT2G09380			17751372	17751921	39.82
	AT2G42620	MAX2	F-box protein MAX2 [Source:UniProtKB/Swiss-Prot;Acc:Q9SIM9]	17756069	17758468	46.04
	AT2G42640		Mitogen activated protein kinase kinase kinase-like protein [Source:UniProtKB/TrEMBL;Acc:F4IP61]	17758532	17764185	37.99
	AT2G42650		Ribosomal protein L1p/L10e family [Source:UniProtKB/TrEMBL;Acc:Q8S8D2]	17765178	17766888	38.4
	AT2G42660		At2g45350/F14N22.7 [Source:UniProtKB/TrEMBL;Acc:Q9SJJ0]	17767050	17768379	41.28
	AT2G09385			17769881	17770118	36.55
	AT2G09390			17770017	17770388	36.29
	AT2G42670		Protein of unknown function (DUF1637) [Source:TAIR;Acc:AT2G42670]	17771697	17774647	37.99
	AT2G42680	MBF1A	MBF1A [Source:UniProtKB/TrEMBL;Acc:A0A178VSD5]	17774832	17776320	39.15

AT2G42690		Phospholipase A1-IIdelta [Source:UniProtKB/Swiss-Prot;Acc:Q9SJI7]	17776241	17777717	43.67
AT2G09395			17777597	17777692	40.62
AT2G42700		FUNCTIONS IN: molecular_function unknown; INVOLVED IN: vesicle-mediated transport, vesicle docking involved in exocytosis;	17778130	17782188	40.08
AT2G42710		Ribosomal protein L1p/L10e family [Source:UniProtKB/TrEMBL;Acc:Q8RWT4]	17782308	17785090	38.63
AT2G42720		F-box/LRR-repeat protein At2g42720 [Source:UniProtKB/Swiss-Prot;Acc:Q6DR13]	17785354	17787421	38.2
AT2G42725		Transcription factor [Source:UniProtKB/TrEMBL;Acc:A0A1P8AZ37]	17787454	17788331	43.85
AT2G42730		F-box/LRR-repeat protein At2g42730 [Source:UniProtKB/Swiss-Prot;Acc:Q0WR05]	17789404	17791582	37.45
AT2G42740	RPL11A	60S ribosomal protein L11-1 [Source:UniProtKB/Swiss-Prot;Acc:P42795]	17791692	17793142	39.63
AT2G42750		At2g42750/F7D19.25 [Source:UniProtKB/TrEMBL;Acc:Q9SJI1]	17793265	17795728	38.96
AT2G42760		At2g42760 [Source:UniProtKB/TrEMBL;Acc:Q9SJI0]	17795817	17797413	37.63
AT2G42770		Expressed protein [Source:UniProtKB/TrEMBL;Acc:Q9SJH9]	17798266	17800072	38.9
AT2G42780		At2g42780/F7D19.22 [Source:UniProtKB/TrEMBL;Acc:Q9SJH8]	17800312	17802816	37.05
AT2G42790	CSY3	Citrate synthase [Source:UniProtKB/TrEMBL;Acc:Q0WUX6]	17802841	17806073	40.18
AT2G09400			17804407	17804604	34.85
AT2G42800	AtRLP29	RLP29 [Source:UniProtKB/TrEMBL;Acc:A0A178VT48]	17807906	17810265	39.96
AT2G42810	PAPP5	Serine/threonine-protein phosphatase 5 [Source:UniProtKB/Swiss-Prot;Acc:Q84XU2]	17811935	17816772	37.21
AT2G42820	HVA22F	HVA22-like protein f [Source:UniProtKB/Swiss-Prot;Acc:Q682H0]	17817244	17818545	32.49
AT2G42830	SHP2	K-box region and MADS-box transcription factor family protein [Source:TAIR;Acc:AT2G42830]	17820114	17824013	33.1
AT2G42835		other RNA [Source:TAIR;Acc:AT2G42835]	17822258	17824975	35.21
AT2G42840	PDF1	Protodermal factor 1 [Source:UniProtKB/Swiss-Prot;Acc:Q9S728]	17826071	17827648	42.59
AT2G42850	CYP718	CYP718 [Source:UniProtKB/TrEMBL;Acc:A0A178VUF2]	17831408	17833545	38.73
AT2G42860		Expressed protein [Source:UniProtKB/TrEMBL;Acc:Q9SJH1]	17833700	17835113	39.67
AT2G42865			17835271	17835594	43.52
AT2G42870	PAR1	Transcription factor PAR1 [Source:UniProtKB/Swiss-Prot;Acc:Q9SJH0]	17836416	17837618	36.16
AT2G09405			17838121	17838596	43.91
AT2G42880	MPK20	Mitogen-activated protein kinase 20 [Source:UniProtKB/Swiss-Prot;Acc:Q9SJG9]	17840168	17844380	40.02
AT2G42885		Defensin-like protein 54 [Source:UniProtKB/Swiss-Prot;Acc:Q8GY39]	17845703	17846603	32.74
AT2G42890	ML2	ML2 [Source:UniProtKB/TrEMBL;Acc:A0A178VQK0]	17849819	17854553	39.18
AT2G42900		At2g42900 [Source:UniProtKB/TrEMBL;Acc:Q9SJG7]	17854557	17855511	45.86
AT2G42910	PRS4	Ribose-phosphate pyrophosphokinase 4 [Source:UniProtKB/Swiss-Prot;Acc:Q680A5]	17856249	17858667	38.65
AT2G42920	PCMP-E75	Pentatricopeptide repeat-containing protein At2g42920, chloroplastic [Source:UniProtKB/Swiss-Prot;Acc:Q9SJG6]	17858655	17863466	39.82
AT2G42930		At2g42930 [Source:UniProtKB/TrEMBL;Acc:Q9SJG5]	17860868	17861272	41.23
AT2G42940	AHL16	AT-hook motif nuclear-localized protein [Source:UniProtKB/TrEMBL;Acc:A0A178VQG3]	17862254	17863456	42.14
AT2G42950		At2g42950 [Source:UniProtKB/TrEMBL;Acc:Q8GY73]	17863678	17866572	39.21
AT2G42955		unknown protein; BEST Arabidopsis thaliana protein match is: unknown protein (TAIR:AT2G30615.1); Ha. [Source:TAIR;Acc:AT2	17866686	17867572	44.19
AT2G42960		Probable receptor-like protein kinase At2g42960 [Source:UniProtKB/Swiss-Prot;Acc:Q9SJG2]	17868150	17871599	37.48
AT2G42970		pre-tRNA [Source:TAIR;Acc:AT2G42970]	17873690	17873762	53.42
AT2G42975		Myosin-G heavy chain-like protein [Source:UniProtKB/TrEMBL;Acc:Q8RXT7]	17873869	17874936	38.39
AT2G42980		Eukaryotic aspartyl protease family protein [Source:UniProtKB/TrEMBL;Acc:Q9SJG1]	17875005	17876779	41.75
AT2G42990		GDSL esterase/lipase At2g42990 [Source:UniProtKB/Swiss-Prot;Acc:Q67ZI9]	17878944	17880563	39.44
AT2G43000	JUB1	Transcription factor JUNGBRUNNEN 1 [Source:UniProtKB/Swiss-Prot;Acc:Q9SK55]	17880457	17882636	35.28
AT2G09410			17884579	17884864	30.77
AT2G43010	PIF4	phytochrome interacting factor 4 [Source:TAIR;Acc:AT2G43010]	17886101	17889087	37.93
AT2G43020	PAO2	Probable polyamine oxidase 2 [Source:UniProtKB/Swiss-Prot;Acc:Q9SKX5]	17891503	17894635	40.79
AT2G43030	RPL3A	50S ribosomal protein L3-1, chloroplastic [Source:UniProtKB/Swiss-Prot;Acc:Q9SKX4]	17894796	17895882	42.5
AT2G43040	NPG1	NPG1 [Source:UniProtKB/TrEMBL;Acc:A0A178VP56]	17895883	17899556	38.89
AT2G43050	PME16	Probable pectinesterase/pectinesterase inhibitor 16 [Source:UniProtKB/Swiss-Prot;Acc:Q9SKX2]	17902370	17904443	43.15
AT2G43060	IBH1	Transcription factor IBH1 [Source:UniProtKB/Swiss-Prot;Acc:Q9SKX1]	17909007	17910210	36.05
AT2G43070	SPPL3	SIGNAL PEPTIDE PEPTIDASE-LIKE 3 [Source:TAIR;Acc:AT2G43070]	17910523	17915009	38.04
AT2G43080	P4H1	Prolyl 4-hydroxylase 1 [Source:UniProtKB/Swiss-Prot;Acc:Q9ZW86]	17915448	17918826	35.72
AT2G43090		3-isopropylmalate dehydratase small subunit 3 [Source:UniProtKB/Swiss-Prot;Acc:Q9ZW85]	17918926	17920496	40.36
AT2G43100	IPMI2	IPMI2 [Source:UniProtKB/TrEMBL;Acc:A0A178VZE1]	17920647	17921844	43.41
AT2G43110		At2g43110 [Source:UniProtKB/TrEMBL;Acc:Q8GWB4]	17922019	17924542	35.66
AT2G43137		snoRNA [Source:TAIR;Acc:AT2G43137]	17923081	17923157	38.96
AT2G43138		snoRNA [Source:TAIR;Acc:AT2G43138]	17923238	17923316	35.44
AT2G43139		snoRNA [Source:TAIR;Acc:AT2G43139]	17923399	17923477	35.44
AT2G43141		snoRNA [Source:TAIR;Acc:AT2G43141]	17923563	17923637	38.67
AT2G09415			17924750	17925039	32.76
AT2G43120		RmlC-like cupins superfamily protein [Source:TAIR;Acc:AT2G43120]	17927181	17929138	34.88
AT2G43130	RABA5C	Ras-related protein RABA5c [Source:UniProtKB/Swiss-Prot;Acc:P28187]	17929607	17931027	39.48
AT2G43140		Basic helix-loop-helix (BHLH) DNA-binding superfamily protein [Source:UniProtKB/TrEMBL;Acc:F4IQ66]	17931254	17935748	33.99
AT2G09420			17931463	17931852	33.33
AT2G43150		Proline-rich extensin-like family protein [Source:UniProtKB/TrEMBL;Acc:Q9ZW80]	17945662	17947155	41.63
AT2G43160	EPSIN2	Clathrin interactor EPSIN 2 [Source:UniProtKB/Swiss-Prot;Acc:Q67YI9]	17947800	17953669	39.98
AT2G09425			17948353	17948670	33.96
AT2G43180		Phosphoenolpyruvate carboxylase family protein [Source:UniProtKB/TrEMBL;Acc:Q8GYI4]	17953497	17955829	40.33
AT2G43190		POP4 [Source:UniProtKB/TrEMBL;Acc:A0A384KZC1]	17955953	17958226	36.68
AT2G43200		Probable methyltransferase PMT19 [Source:UniProtKB/Swiss-Prot;Acc:Q9ZW75]	17958059	17960672	40.63
AT2G43210	PUX11	Plant UBX domain-containing protein 11 [Source:UniProtKB/Swiss-Prot;Acc:Q9ZW74]	17960648	17963769	38.63
AT2G43220		Cysteine/Histidine-rich C1 domain family protein [Source:UniProtKB/TrEMBL;Acc:Q9ZW73]	17963747	17966211	39.39
AT2G43230		Protein kinase superfamily protein [Source:TAIR;Acc:AT2G43230]	17966251	17968726	39.05
AT2G43235		Phosphoribosylformylglycinamidine synthase [Source:UniProtKB/TrEMBL;Acc:Q56Y19]	17968698	17970835	39.99
AT2G09430			17968981	17969762	39.26
AT2G43240		CMP-sialic acid transporter 2 [Source:UniProtKB/Swiss-Prot;Acc:Q8GY97]	17970859	17975938	36.69
AT2G43250		Transmembrane protein [Source:UniProtKB/TrEMBL;Acc:Q9ZW70]	17977228	17979736	40.41
AT2G43255		CONTAINS InterPro DOMAIN/s: O-acyltransferase, WSD1, C-terminal (InterPro:IPR009721); BEST Arabidopsis thaliana protein r	17979703	17981309	33.29
AT2G43260		F-box and associated interaction domains-containing protein [Source:TAIR;Acc:AT2G43260]	17983506	17985185	41.79
AT2G43261		unknown protein; FUNCTIONS IN: molecular_function unknown; INVOLVED IN: biological_process unknown; LOCATED IN: cel	17985247	17986775	32.83
AT2G43270		F-box and associated interaction domains-containing protein [Source:UniProtKB/TrEMBL;Acc:F4IR26]	17988175	17989491	42.22
AT2G43280		Far-red impaired responsive (FAR1) family protein [Source:UniProtKB/TrEMBL;Acc:Q84J94]	17989602	17991022	36.95
AT2G43290	CML5	MSS3 [Source:UniProtKB/TrEMBL;Acc:A0A178VP84]	17991052	17992047	39.96
AT2G43300		pre-tRNA [Source:TAIR;Acc:AT2G43300]	17995573	17995644	52.78
AT2G43310		F14B2.25/F14B2.25 [Source:UniProtKB/TrEMBL;Acc:O22846]	17995901	17996688	38.2
AT2G09435			17996262	17996462	50.25
AT2G43320		At2g43320/T1O24.6 [Source:UniProtKB/TrEMBL;Acc:O22847]	17996650	18000027	37.15
AT2G09440			17998331	17999567	35.89
AT2G43330	INT1	Inositol transporter 1 [Source:UniProtKB/Swiss-Prot;Acc:Q8VZR6]	18000822	18004195	39.3
AT2G09445			18004724	18005223	33.8
AT2G09450			18005902	18006425	28.24
AT2G43340		At2g43340 [Source:UniProtKB/TrEMBL;Acc:Q94JW9]	18007629	18008665	37.13
AT2G43350	GPX3	Probable glutathione peroxidase 3, mitochondrial [Source:UniProtKB/Swiss-Prot;Acc:O22850]	18008482	18010614	36.1
AT2G09455			18008822	18009202	38.85
AT2G43360	BIO2	Biotin synthase, mitochondrial [Source:UniProtKB/Swiss-Prot;Acc:P54967]	18010676	18013252	39.08
AT2G43370	SNRNP35	U11/U12 small nuclear ribonucleoprotein 35 kDa protein [Source:UniProtKB/Swiss-Prot;Acc:Q8VY74]	18013268	18015530	39.33
AT2G43375		other RNA [Source:TAIR;Acc:AT2G43375]	18017116	18018691	35.41
AT2G09460			18017765	18017920	33.97
AT2G09465			18018220	18018382	40.49
AT2G43386		unknown protein; LOCATED IN: endomembrane system; Ha. [Source:TAIR;Acc:AT2G43386]	18019449	18020088	30.47
AT2G43390		Putative uncharacterized protein [Source:UniProtKB/TrEMBL;Acc:Q5BPR6]	18020229	18020879	39.02
AT2G43400	ETFQO	Electron transfer flavoprotein-ubiquinone oxidoreductase, mitochondrial [Source:UniProtKB/Swiss-Prot;Acc:O22854]	18021109	18025292	37.74
AT2G43410	FPA	Flowering time control protein FPA [Source:UniProtKB/Swiss-Prot;Acc:Q8LPQ9]	18025247	18031255	40.59
AT2G09470			18025969	18026380	34.71
AT2G43420	3BETAHSD/D3	3beta-hydroxysteroid-dehydrogenase/decarboxylase isoform 3 [Source:UniProtKB/Swiss-Prot;Acc:A9X4U2]	18031268	18035182	38.34
AT2G43430	GLX2-1	Hydroxyacylglutathione hydrolase 1, mitochondrial [Source:UniProtKB/Swiss-Prot;Acc:O24495]	18035349	18038334	37.17
AT2G43440		F-box protein At2g43440 [Source:UniProtKB/Swiss-Prot;Acc:A8MS20]	18039306	18040732	41.7

AT2G43445		F-box/kelch-repeat protein At2g43445 [Source:UniProtKB/Swiss-Prot;Acc:Q0WRU9]	18041407	18043110	37.97
AT2G43450		Uncharacterized protein At2g43450 [Source:UniProtKB/TrEMBL;Acc:O22859]	18043234	18044225	34.58
AT2G09475			18044316	18044778	38.44
AT2G43460	RPL38A	60S ribosomal protein L38 [Source:UniProtKB/Swiss-Prot;Acc:O22860]	18045965	18047401	35.42
AT2G43465		RNA-binding ASCH domain protein [Source:UniProtKB/TrEMBL;Acc:F4IR54]	18047533	18049794	37.8
AT2G43470		Protein of unknown function (DUF3755) [Source:TAIR;Acc:AT2G43470]	18049799	18051589	33.5
AT2G43480	PER26	Probable peroxidase 26 [Source:UniProtKB/Swiss-Prot;Acc:O22862]	18052967	18054417	41.49
AT2G43490		Ypt/Rab-GAP domain of gyp1p superfamily protein [Source:UniProtKB/TrEMBL;Acc:B3H765]	18054392	18058924	37.02
AT2G09480			18059267	18059674	40.44
AT2G43500	NLP8	Plant regulator RWP-RK family protein [Source:TAIR;Acc:AT2G43500]	18061716	18066708	39.21
AT2G43510	ATTI1	TI1 [Source:UniProtKB/TrEMBL;Acc:A0A178VRC4]	18066960	18067860	36.07
AT2G43520	ATTI2	TI2 [Source:UniProtKB/TrEMBL;Acc:A0A178W058]	18068425	18069416	37.7
AT2G43530	ATTI3	Defensin-like protein 194 [Source:UniProtKB/Swiss-Prot;Acc:O22867]	18070110	18070523	35.51
AT2G43535	ATTI4	Defensin-like protein 196 [Source:UniProtKB/Swiss-Prot;Acc:Q8RYE7]	18070960	18071826	35.87
AT2G43540		At2g43540 [Source:UniProtKB/TrEMBL;Acc:O22868]	18072127	18073100	41.99
AT2G43550	ATTI6	Defensin-like protein 197 [Source:UniProtKB/Swiss-Prot;Acc:O22869]	18073305	18073651	44.09
AT2G43560	FKBP16-3	Peptidylprolyl isomerase [Source:UniProtKB/TrEMBL;Acc:A0A178VVY6]	18073859	18075498	38.23
AT2G43570	CHI	CHI [Source:UniProtKB/TrEMBL;Acc:A0A178W3M3]	18076166	18077517	42.46
AT2G43580		Endochitinase At2g43580 [Source:UniProtKB/Swiss-Prot;Acc:O24598]	18078649	18080094	39.76
AT2G43590		Endochitinase At2g43590 [Source:UniProtKB/Swiss-Prot;Acc:O24658]	18081304	18082776	39.38
AT2G43600		Chitinase family protein [Source:TAIR;Acc:AT2G43600]	18085961	18087085	38.93
AT2G43610		Endochitinase At2g43610 [Source:UniProtKB/Swiss-Prot;Acc:O22842]	18087812	18089371	39.81
AT2G09490			18092549	18093025	33.96
AT2G43620		Endochitinase At2g43620 [Source:UniProtKB/Swiss-Prot;Acc:O22841]	18093694	18095093	41.71
AT2G43630		Nucleusenvelope protein [Source:UniProtKB/TrEMBL;Acc:Q8VYY8]	18095685	18097488	39.36
AT2G43640	SRP14	Signal recognition particle 14 kDa protein [Source:UniProtKB/Swiss-Prot;Acc:O04421]	18097400	18099136	35.75
AT2G09495			18098686	18099024	34.81
AT2G43650	EMB2777	Sas10/U3 ribonucleoprotein (Utp) family protein [Source:UniProtKB/TrEMBL;Acc:Q8L3P4]	18099236	18103438	37.62
AT2G43660		Carbohydrate-binding X8 domain superfamily protein [Source:UniProtKB/TrEMBL;Acc:F4IS25]	18103639	18104591	32.63
AT2G43670		At2g43670 [Source:UniProtKB/TrEMBL;Acc:Q6IDB5]	18105085	18106043	32.12
AT2G43680	IQD14	IQD14 [Source:UniProtKB/TrEMBL;Acc:A0A178VRE6]	18108119	18111831	42.53
AT2G43690	LECRK53	Probable L-type lectin-domain containing receptor kinase V.3 [Source:UniProtKB/Swiss-Prot;Acc:O22834]	18112026	18114834	40.73
AT2G43700	LECRK54	L-type lectin-domain containing receptor kinase V.4 [Source:UniProtKB/Swiss-Prot;Acc:O22833]	18115064	18118878	40.97
AT2G43710	FAB2	Stearoyl-[acyl-carrier-protein] 9-desaturase 7, chloroplastic [Source:UniProtKB/Swiss-Prot;Acc:O22832]	18119873	18122889	39.31
AT2G43720		At2g43720/F18O19.17 [Source:UniProtKB/TrEMBL;Acc:O22831]	18123167	18124471	36.17
AT2G43730	JAL24	Jacalin-related lectin 24 [Source:UniProtKB/Swiss-Prot;Acc:Q84X07]	18124648	18125868	33.5
AT2G43740	JAL25	Jacalin-related lectin 25 [Source:UniProtKB/Swiss-Prot;Acc:O22829]	18126578	18128293	34.91
AT2G43745		BEST Arabidopsis thaliana protein match is: Mannose-binding lectin superfamily protein (TAIR:AT2G43730.1); Ha. [Source:TAIR;	18128529	18129841	38.92
AT2G43750	OASB	Cysteine synthase [Source:UniProtKB/TrEMBL;Acc:Q0WW95]	18129435	18132638	38.33
AT2G43760	MOCS2	Molybdopterin synthase catalytic subunit [Source:UniProtKB/TrEMBL;Acc:A0A178VNE3]	18132484	18134068	37.92
AT2G43770		At2g43770 [Source:UniProtKB/TrEMBL;Acc:O22826]	18134061	18136036	41.4
AT2G43780		unknown protein; Ha. [Source:TAIR;Acc:AT2G43780]	18136108	18137803	37.79
AT2G43790	MPK6	Mitogen-activated protein kinase [Source:UniProtKB/TrEMBL;Acc:A0A178VTX8]	18138246	18140988	37.88
AT2G09500			18138686	18138952	30.34
AT2G43795		unknown protein; BEST Arabidopsis thaliana protein match is: unknown protein (TAIR:AT3G59800.1); Ha. [Source:TAIR;Acc:AT2	18140944	18142435	38.14
AT2G43800	FH2	Formin-like protein [Source:UniProtKB/TrEMBL;Acc:A0A178VS83]	18145439	18149031	45.03
AT2G43810	LSM6B	Sm-like protein LSM36B [Source:UniProtKB/Swiss-Prot;Acc:O22823]	18149197	18151295	36.06
AT2G43820	UGT74F2	UDP-glycosyltransferase 74F2 [Source:UniProtKB/Swiss-Prot;Acc:O22822]	18152078	18153908	41.23
AT2G43840	UGT74F1	Glycosyltransferase [Source:UniProtKB/TrEMBL;Acc:F4IS54]	18157529	18159231	42.69
AT2G09505			18158745	18159100	44.1
AT2G43850	ILK1	Integrin-linked protein kinase 1 [Source:UniProtKB/Swiss-Prot;Acc:F4IS56]	18159182	18162250	38.71
AT2G43860		Probable polygalacturonase At2g43860 [Source:UniProtKB/Swiss-Prot;Acc:O22818]	18162720	18164428	39.2
AT2G43865		Putative uncharacterized protein [Source:UniProtKB/TrEMBL;Acc:Q1G3R3]	18165419	18165878	43.48
AT2G43870		Pectin lyase-like superfamily protein [Source:UniProtKB/TrEMBL;Acc:O22817]	18166336	18168381	39.64
AT2G43880		Pectin lyase-like superfamily protein [Source:UniProtKB/TrEMBL;Acc:Q9SLM8]	18172889	18174705	38.64
AT2G43890		Pectin lyase-like superfamily protein [Source:UniProtKB/TrEMBL;Acc:O80559]	18176831	18179178	39.57
AT2G43900		Endonuclease/exonuclease/phosphatase family protein [Source:TAIR;Acc:AT2G43900]	18178588	18184089	42.97
AT2G43910	ATHOL1	HOL1 [Source:UniProtKB/TrEMBL;Acc:A0A178VXT1]	18184409	18187358	31.08
AT2G43920	HOL2	HOL2 [Source:UniProtKB/TrEMBL;Acc:A0A178VP97]	18189004	18191348	33.65
AT2G43930		Protein kinase superfamily protein [Source:TAIR;Acc:AT2G43930]	18193137	18195582	37.2
AT2G43940	HOL3	HOL3 [Source:UniProtKB/TrEMBL;Acc:A0A178VV10]	18195677	18197767	36.87
AT2G43945		unknown protein; LOCATED IN: chloroplast; BEST Arabidopsis thaliana protein match is: unknown protein (TAIR:AT3G59870.1);	18197822	18200198	37.61
AT2G43950	OEP37	Outer envelope pore protein 37, chloroplastic [Source:UniProtKB/Swiss-Prot;Acc:O80565]	18200316	18202786	38.37
AT2G43960		At2g43960 [Source:UniProtKB/TrEMBL;Acc:Q58FX2]	18203312	18204912	41.91
AT2G43970	LARP6B	La-related protein 6B [Source:UniProtKB/Swiss-Prot;Acc:O80567]	18205235	18208218	41.42
AT2G43980	ITPK4	Inositol 1,3,4-trisphosphate 5/6-kinase 4 [Source:UniProtKB/Swiss-Prot;Acc:O80568]	18208253	18211644	37.88
AT2G43990		GPI-anchored adhesin-like protein [Source:UniProtKB/TrEMBL;Acc:O80569]	18211734	18214042	40.06
AT2G44000		Late embryogenesis abundant (LEA) hydroxyproline-rich glycoprotein family [Source:UniProtKB/TrEMBL;Acc:O80570]	18214089	18215571	35.2
AT2G44010		At2g44010 [Source:UniProtKB/TrEMBL;Acc:O80571]	18216574	18217453	34.43
AT2G44020		Expressed protein [Source:UniProtKB/TrEMBL;Acc:O80572]	18217559	18219559	40.23
AT2G44030		Putative F-box/kelch-repeat protein At2g44030 [Source:UniProtKB/Swiss-Prot;Acc:O80573]	18220086	18221228	45.67
AT2G44040	DAPB1	4-hydroxy-tetrahydrodipicolinate reductase 1, chloroplastic [Source:UniProtKB/Swiss-Prot;Acc:O80574]	18221694	18224162	39.37
AT2G44050	COS1	6,7-dimethyl-8-ribityllumazine synthase, chloroplastic [Source:UniProtKB/Swiss-Prot;Acc:O80575]	18224213	18226233	38
AT2G44060		At2g44060 [Source:UniProtKB/TrEMBL;Acc:O80576]	18226263	18228293	39.49
AT2G44065		Expressed protein [Source:UniProtKB/TrEMBL;Acc:Q8VZU4]	18228430	18231407	36.74
AT2G44070		NagB/RpiA/CoA transferase-like superfamily protein [Source:UniProtKB/TrEMBL;Acc:F4IT40]	18230865	18232630	37.54
AT2G44080	ARL	ARGOS-like protein [Source:UniProtKB/Swiss-Prot;Acc:Q8RXL7]	18236789	18238173	36.46
AT2G44090		AT2G44090 protein [Source:UniProtKB/TrEMBL;Acc:O80579]	18238269	18241410	40.52
AT2G44100	GDI1	Guanosine nucleotide diphosphate dissociation inhibitor 1 [Source:UniProtKB/Swiss-Prot;Acc:Q96254]	18241469	18245008	37.49
AT2G09510			18241870	18242256	34.63
AT2G44110	MLO15	MLO-like protein [Source:UniProtKB/TrEMBL;Acc:A0A178VQX0]	18245357	18248459	36.26
AT2G44120		Ribosomal protein L30/L7 family protein [Source:UniProtKB/TrEMBL;Acc:F4IT48]	18248830	18251156	37.13
AT2G44140	ATG4A	Cysteine protease ATG4a [Source:UniProtKB/Swiss-Prot;Acc:Q8S929]	18253497	18258293	41.57
AT2G44130		F-box/kelch-repeat protein At2g44130 [Source:UniProtKB/Swiss-Prot;Acc:O80582]	18253610	18255054	47.13
AT2G44150	ASHH3	SDG7 [Source:UniProtKB/TrEMBL;Acc:A0A178VNH9]	18258569	18261542	37.9
AT2G44160	MTHFR2	Methylenetetrahydrofolate reductase 2 [Source:UniProtKB/Swiss-Prot;Acc:O80585]	18262189	18265346	38.85
AT2G44175		Acyl-CoA N-acyltransferases (NAT) superfamily protein [Source:UniProtKB/TrEMBL;Acc:Q3EBG4]	18268788	18269214	39.11
AT2G44180	MAP2A	Methionine aminopeptidase 2A [Source:UniProtKB/Swiss-Prot;Acc:Q9FV49]	18269211	18271941	36.76
AT2G44190	EDE1	Protein ENDOSPERM DEFECTIVE 1 [Source:UniProtKB/Swiss-Prot;Acc:O80588]	18272119	18274679	41.12
AT2G44195		Pre-mRNA splicing factor domain-containing protein [Source:UniProtKB/TrEMBL;Acc:Q1PEU6]	18274723	18275792	38.41
AT2G44198		unknown protein; Ha. [Source:TAIR;Acc:AT2G44198]	18276066	18276164	41.41
AT2G44200		At2g44200/F6E13.34 [Source:UniProtKB/TrEMBL;Acc:O64855]	18276152	18278627	41.4
AT2G44210		AT2G44210 protein [Source:UniProtKB/TrEMBL;Acc:B9DGK6]	18280580	18283014	36.71
AT2G44220		Protein of Unknown Function (DUF239) [Source:TAIR;Acc:AT2G44220]	18283789	18285837	37.09
AT2G44230		At2g44230/F4I1.4 [Source:UniProtKB/TrEMBL;Acc:O64858]	18286321	18288576	42.73
AT2G44240		At2g44240/F4I1.5 [Source:UniProtKB/TrEMBL;Acc:O64859]	18289824	18292374	35.05
AT2G44250		Protein of Unknown Function (DUF239) [Source:TAIR;Acc:AT2G44250]	18293457	18295536	37.16
AT2G44255		other RNA [Source:TAIR;Acc:AT2G44255]	18295758	18297860	41.08
AT2G44260		Plant protein of unknown function (DUF946) [Source:TAIR;Acc:AT2G44260]	18295838	18297867	41.53
AT2G44270	NCS6	Cytoplasmic tRNA 2-thiolation protein 1 [Source:UniProtKB/TrEMBL;Acc:A0A178VUZ0]	18297878	18300304	36.71
AT2G09515			18300725	18300931	42.03
AT2G44280		AT3g60070/T2O9_50 [Source:UniProtKB/TrEMBL;Acc:Q8L7S2]	18301223	18304627	35.12
AT2G44290	YLS3	Protein YLS3 [Source:UniProtKB/Swiss-Prot;Acc:O64864]	18305080	18306395	39.82
AT2G44300		At2g44300 [Source:UniProtKB/TrEMBL;Acc:O64865]	18307223	18308494	37.89
AT2G44310		Calcium-binding EF-hand family protein [Source:UniProtKB/TrEMBL;Acc:O64866]	18309022	18309826	39.88

AT2G44320		pre-tRNA [Source:TAIR;Acc:AT2G44320]	18309977	18310058	58.54
AT2G44330		At2g44330/F4I1.14 [Source:UniProtKB/TrEMBL;Acc:O64867]	18310586	18311185	46
AT2G44340	VQ18	VQ motif-containing protein 18 [Source:UniProtKB/Swiss-Prot;Acc:O64868]	18314188	18315322	38.06
AT2G44350	CSY4	Citrate synthase 4, mitochondrial [Source:UniProtKB/Swiss-Prot;Acc:P20115]	18316278	18320789	37.92
AT2G44360		Ecotropic viral integration site protein [Source:UniProtKB/TrEMBL;Acc:O64870]	18320732	18321696	37.51
AT2G44370		At2g44370 [Source:UniProtKB/TrEMBL;Acc:O64871]	18321951	18323121	41.33
AT2G44380		At2g44380 [Source:UniProtKB/TrEMBL;Acc:O64872]	18323366	18324561	39.63
AT2G44390		Cysteine/Histidine-rich C1 domain family protein [Source:UniProtKB/TrEMBL;Acc:O64873]	18325811	18326551	46.56
AT2G44400		Cysteine/Histidine-rich C1 domain family protein [Source:UniProtKB/TrEMBL;Acc:O64874]	18326954	18327888	43.74
AT2G44410		At2g44410 [Source:UniProtKB/TrEMBL;Acc:Q6NLR3]	18328322	18330420	40.02
AT2G44420		protein N-terminal asparagine amidohydrolase family protein [Source:TAIR;Acc:AT2G44420]	18330447	18332936	37.03
AT2G44430		At2g44430 [Source:UniProtKB/TrEMBL;Acc:O64877]	18333370	18336364	40.03
AT2G44440	EML4	Protein EMSY-LIKE 4 [Source:UniProtKB/Swiss-Prot;Acc:Q08A72]	18336400	18339275	38.98
AT2G44450	BGLU15	Beta-glucosidase 15 [Source:UniProtKB/Swiss-Prot;Acc:O64879]	18340602	18343927	34.64
AT2G44460	BGLU28	Beta-glucosidase 28 [Source:UniProtKB/Swiss-Prot;Acc:Q4V3B3]	18346306	18350026	33.16
AT2G09525			18350808	18351515	38.28
AT2G44470	BGLU29	Beta-glucosidase 29 [Source:UniProtKB/Swiss-Prot;Acc:Q8GXT2]	18354186	18358679	31.86
AT2G44480	BGLU17	beta glucosidase 17 [Source:TAIR;Acc:AT2G44480]	18359663	18363346	33.41
AT2G44490	BGLU26	Beta-glucosidase 26, peroxisomal [Source:UniProtKB/Swiss-Prot;Acc:O64883]	18364652	18367835	36.53
AT2G09530			18368812	18369052	41.49
AT2G44500	OFUT20	O-fucosyltransferase 20 [Source:UniProtKB/Swiss-Prot;Acc:O64884]	18374162	18376799	39.5
AT2G44510		Protein BCCIP homolog [Source:UniProtKB/TrEMBL;Acc:A0A178VXZ2]	18377048	18379265	35.84
AT2G44520	COX10	Protoheme IX farnesyltransferase, mitochondrial [Source:UniProtKB/Swiss-Prot;Acc:O64886]	18379527	18381959	39.79
AT2G44525		At2g44525 [Source:UniProtKB/TrEMBL;Acc:Q8RUX8]	18382070	18383513	37.4
AT2G44530	PRS5	Ribose-phosphate pyrophosphokinase 5, chloroplastic [Source:UniProtKB/Swiss-Prot;Acc:O64888]	18383515	18386208	39.35
AT2G44540	AtGH9B9	Endoglucanase 12 [Source:UniProtKB/Swiss-Prot;Acc:O64889]	18386219	18388110	42.07
AT2G44550	AtGH9B10	Endoglucanase 13 [Source:UniProtKB/Swiss-Prot;Acc:O64890]	18389381	18391142	43.76
AT2G44560	AtGH9B11	Endoglucanase 14 [Source:UniProtKB/Swiss-Prot;Acc:Q8S8Q4]	18391753	18393729	42.29
AT2G44570	AtGH9B12	Endoglucanase 15 [Source:UniProtKB/Swiss-Prot;Acc:O80497]	18394196	18396337	41.41
AT2G44581		RING/U-box superfamily protein [Source:UniProtKB/TrEMBL;Acc:B3H6J7]	18397874	18398504	43.58
AT2G44578		RING/U-box superfamily protein [Source:UniProtKB/TrEMBL;Acc:A8MS73]	18400646	18401339	43.08
AT2G44580		Zinc ion binding protein [Source:UniProtKB/TrEMBL;Acc:F4IU60]	18401777	18403654	38.6
AT2G44590	DRP1D	At2g44590 [Source:UniProtKB/TrEMBL;Acc:B5X4Z5]	18403682	18407103	37.96
AT2G44600		At2g44600/F16B22.9 [Source:UniProtKB/TrEMBL;Acc:O80500]	18408579	18410170	39.95
AT2G44610	RABH1B	Ras-related protein RABH1b [Source:UniProtKB/Swiss-Prot;Acc:O80501]	18411424	18414153	37.66
AT2G44620	MTACP1	Acyl carrier protein 1, mitochondrial [Source:UniProtKB/Swiss-Prot;Acc:P53665]	18414201	18415335	37.8
AT2G44630		F-box/kelch-repeat protein At2g44630 [Source:UniProtKB/Swiss-Prot;Acc:O80502]	18415596	18417013	42.31
AT2G44640		Expressed protein [Source:UniProtKB/TrEMBL;Acc:O80503]	18417243	18419396	42.01
AT2G44650	CPN10-2	10 kDa chaperonin 2, chloroplastic [Source:UniProtKB/Swiss-Prot;Acc:O80504]	18419370	18421110	36.99
AT2G44660		Probable dolichyl pyrophosphate Glc1Man9GlcNAc2 alpha-1,3-glucosyltransferase [Source:UniProtKB/Swiss-Prot;Acc:O80505]	18420832	18422839	41.38
AT2G09535			18423374	18423671	37.92
AT2G44670	FLZ3	FCS-Like Zinc finger 3 [Source:UniProtKB/Swiss-Prot;Acc:O80506]	18425111	18426121	36.4
AT2G44680	CKB4	Putative casein kinase II subunit beta-4 [Source:UniProtKB/Swiss-Prot;Acc:O80507]	18426481	18428397	37.09
AT2G44690	ARAC9	ROP8 [Source:UniProtKB/TrEMBL;Acc:A0A384LBU4]	18429121	18430882	35.3
AT2G44700		F-box/kelch-repeat protein At2g44700 [Source:UniProtKB/Swiss-Prot;Acc:Q84RE1]	18431006	18432333	43.07
AT2G44710		At2g44720/F16B22.21 [Source:UniProtKB/TrEMBL;Acc:Q8RWQ1]	18432686	18437221	42.39
AT2G44730		Alcohol dehydrogenase transcription factor Myb/SANT-like family protein [Source:UniProtKB/TrEMBL;Acc:O80512]	18437333	18438565	48.09
AT2G44735		BEST Arabidopsis thaliana protein match is: F-box family protein (TAIR:AT3G18720.1); Ha. [Source:TAIR;Acc:AT2G44735]	18438623	18441681	40.34
AT2G44740	CYCU4-1	Cyclin-U4-1 [Source:UniProtKB/Swiss-Prot;Acc:O80513]	18441828	18443405	34.66
AT2G44745	WRKY12	At2g44745 [Source:UniProtKB/TrEMBL;Acc:Q1PEU5]	18447203	18449093	32.73
AT2G44750	TPK2	Thiamine pyrophosphokinase 2 [Source:UniProtKB/Swiss-Prot;Acc:F4IV16]	18451426	18452875	38.69
AT2G44760		Domain of unknown function (DUF3598) [Source:TAIR;Acc:AT2G44760]	18452851	18454930	40.19
AT2G44770		ELMO/CED-12 family protein [Source:UniProtKB/TrEMBL;Acc:O80516]	18457322	18460383	38.37
AT2G44790	UCC2	Uclacyanin-2 [Source:UniProtKB/Swiss-Prot;Acc:O80517]	18461863	18463533	38.06
AT2G09540			18465129	18465445	37.85
AT2G09545			18465955	18466391	38.22
AT2G44798		other RNA [Source:TAIR;Acc:AT2G44798]	18466053	18471304	33.36
AT2G44800		2-oxoglutarate (2OG) and Fe(II)-dependent oxygenase superfamily protein [Source:UniProtKB/TrEMBL;Acc:F4IV21]	18466820	18468551	39.84
AT2G44810	DAD1	Phospholipase A(1) DAD1, chloroplastic [Source:UniProtKB/Swiss-Prot;Acc:Q948R1]	18478374	18480457	42.99
AT2G44820		unknown protein; Ha. [Source:TAIR;Acc:AT2G44820]	18484074	18485873	36.89
AT2G44830		Protein kinase superfamily protein [Source:UniProtKB/TrEMBL;Acc:F4IV25]	18489607	18493163	42.79
AT2G44840	ERF13	Ethylene-responsive transcription factor 13 [Source:UniProtKB/Swiss-Prot;Acc:Q8L9K1]	18495215	18496327	43.94
AT2G09550			18496740	18497215	37.18
AT2G09555			18497813	18498547	36.46
AT2G44850		Uncharacterized protein At2g44850 [Source:UniProtKB/TrEMBL;Acc:O22166]	18498672	18500879	39.95
AT2G09560			18499560	18499765	38.35
AT2G44860		Probable ribosome biogenesis protein RLP24 [Source:UniProtKB/Swiss-Prot;Acc:O22165]	18500803	18502562	35.17
AT2G09565			18501637	18501859	30.94
AT2G44870		Expressed protein [Source:UniProtKB/TrEMBL;Acc:O22164]	18502987	18504718	38.86
AT2G44880		Pentatricopeptide repeat (PPR-like) superfamily protein [Source:TAIR;Acc:AT2G44880]	18505101	18508298	41.21
AT2G09570			18507786	18508105	44.06
AT2G44890	CYP704A1	Cytochrome P450, family 704, subfamily A, polypeptide 1 [Source:UniProtKB/TrEMBL;Acc:F4IV34]	18508271	18510364	40.11
AT2G09575			18510690	18511293	37.58
AT2G44900	FBX5	Protein ARABIDILLO 1 [Source:UniProtKB/Swiss-Prot;Acc:O22161]	18511313	18516468	39.43
AT2G44910	ATHB-4	Uncharacterized protein At2g44910 (Fragment) [Source:UniProtKB/TrEMBL;Acc:C0SV86]	18517656	18519731	38.92
AT2G09585			18523233	18523560	40.85
AT2G09590			18523395	18523783	43.96
AT2G44920		Thylakoid lumenal 15 kDa protein 1, chloroplastic [Source:UniProtKB/Swiss-Prot;Acc:O22160]	18524311	18526827	35.48
AT2G44925		unknown protein; FUNCTIONS IN: molecular_function unknown; INVOLVED IN: biological_process unknown; LOCATED IN: cel	18527251	18528295	37.13
AT2G44930		Expressed protein [Source:UniProtKB/TrEMBL;Acc:O22159]	18529488	18531752	37.4
AT2G44940	ERF034	Ethylene-responsive transcription factor ERF034 [Source:UniProtKB/Swiss-Prot;Acc:Q8LBQ7]	18537177	18538479	42.06
AT2G44950	HUB1	E3 ubiquitin-protein ligase BRE1-like 1 [Source:UniProtKB/Swiss-Prot;Acc:Q8RXD6]	18542175	18548652	37.77
AT2G44970		Alpha/beta-Hydrolases superfamily protein [Source:UniProtKB/TrEMBL;Acc:Q84WV5]	18548813	18552171	38.85
AT2G44980	CHR10	Probable helicase CHR10 [Source:UniProtKB/Swiss-Prot;Acc:F4IV45]	18552173	18556674	38.72
AT2G44990	CCD7	carotenoid cleavage dioxygenase 7 [Source:TAIR;Acc:AT2G44990]	18558885	18561621	38.55
AT2G44995		Unknown gene [Source:TAIR;Acc:AT2G44995]	18562102	18563678	35
AT2G44993			18563938	18565339	50.78
AT2G45000	NUP62	Nuclear pore complex protein NUP62 [Source:UniProtKB/Swiss-Prot;Acc:Q8L7F7]	18564017	18567892	42.52
AT2G45010		PLAC8 family protein [Source:UniProtKB/TrEMBL;Acc:Q8L3T0]	18567852	18569859	40.34
AT2G45020		pre-tRNA [Source:TAIR;Acc:AT2G45020]	18569911	18569983	54.79
AT2G45023		other RNA [Source:TAIR;Acc:AT2G45023]	18570156	18572173	38.06
AT2G45030	MEFG2	Elongation factor G-2, mitochondrial [Source:UniProtKB/Swiss-Prot;Acc:F4IW10]	18572191	18576970	38.83
AT2G45040	4MMP	Metalloendoproteinase 4-MMP [Source:UniProtKB/Swiss-Prot;Acc:Q8GWW6]	18577500	18578945	43.08
AT2G45050	GATA2	GATA transcription factor 2 [Source:UniProtKB/Swiss-Prot;Acc:O49741]	18582697	18584127	44.3
AT2G45060		Alanine-tRNA ligase [Source:UniProtKB/TrEMBL;Acc:Q94AZ5]	18584291	18586873	37.98
AT2G45070	SEC61 BETA	Protein transport protein Sec61 subunit beta [Source:UniProtKB/Swiss-Prot;Acc:P38389]	18586974	18588452	37.8
AT2G45080	CYCU2-1	Cyclin [Source:UniProtKB/TrEMBL;Acc:A0A178VTM7]	18591628	18592729	36.75
AT2G45100		Cyclin/Brf1-like TBP-binding protein [Source:UniProtKB/TrEMBL;Acc:F4IW19]	18595178	18598481	37.53
AT2G45110	EXPB4	Expansin-B4 [Source:UniProtKB/Swiss-Prot;Acc:Q9SHD1]	18599498	18601339	32.9
AT2G45120	ZAT4	Zinc finger protein ZAT4 [Source:UniProtKB/Swiss-Prot;Acc:Q9SHD0]	18603455	18604878	39.04
AT2G45130	SPX3	SPX domain-containing protein 3 [Source:UniProtKB/Swiss-Prot;Acc:Q5PP62]	18606410	18607853	35.11
AT2G45135		RING/U-box superfamily protein [Source:UniProtKB/TrEMBL;Acc:F4IW24]	18608824	18610574	38.49
AT2G45140	PVA12	Vesicle-associated protein 1-2 [Source:UniProtKB/Swiss-Prot;Acc:Q9SHC8]	18610806	18613302	37.16
AT2G45150	CDS4	cytidinediphosphate diacylglycerol synthase 4 [Source:TAIR;Acc:AT2G45150]	18613305	18615770	40.02
AT2G45160	SCL27	Scarecrow-like protein 27 [Source:UniProtKB/Swiss-Prot;Acc:Q7XJM8]	18617862	18620451	44.21

AT2G45170	ATG8E	Autophagy-related protein 8e [Source:UniProtKB/Swiss-Prot;Acc:Q8S926]	18624264	18625791	33.77
AT2G45180		At2g45180/T14P1.1 [Source:UniProtKB/TrEMBL;Acc:Q42044]	18626188	18627157	38.04
AT2G45190	YAB1	Axial regulator YABBY 1 [Source:UniProtKB/Swiss-Prot;Acc:O22152]	18628252	18630779	35.25
AT2G45200	GOS12	Golgi SNAP receptor complex member 1-2 [Source:UniProtKB/Swiss-Prot;Acc:O22151]	18637335	18640039	37.6
AT2G45210	SAUR36	Auxin-responsive protein SAUR36 [Source:UniProtKB/Swiss-Prot;Acc:O22150]	18641563	18642739	37.72
AT2G45220	PME17	Probable pectinesterase/pectinesterase inhibitor 17 [Source:UniProtKB/Swiss-Prot;Acc:O22149]	18644061	18646606	38.41
AT2G45240	MAP1A	Methionine aminopeptidase 1A [Source:UniProtKB/Swiss-Prot;Acc:Q9SLN5]	18655760	18659111	38.34
AT2G45245		other RNA [Source:TAIR;Acc:AT2G45245]	18659235	18661294	36.6
AT2G45243			18660696	18661296	37.27
AT2G45250		Integral membrane hemolysin-III-like protein [Source:UniProtKB/TrEMBL;Acc:A8MRU4]	18661460	18663064	42.18
AT2G45260		Myosin-4 protein (DUF641) [Source:UniProtKB/TrEMBL;Acc:O22146]	18664390	18666475	40.22
AT2G45270	GCP1	Probable tRNA N6-adenosine threonylcarbamoyltransferase, mitochondrial [Source:UniProtKB/Swiss-Prot;Acc:O22145]	18666409	18669873	39.74
AT2G45280	ATRAD51C	RAS associated with diabetes protein 51C [Source:UniProtKB/TrEMBL;Acc:F4IW45]	18670009	18672469	39.98
AT2G45290	TKL-2	Transketolase-2, chloroplastic [Source:UniProtKB/Swiss-Prot;Acc:F4IW47]	18672497	18676484	40.12
AT2G45300		3-phosphoshikimate 1-carboxyvinyltransferase, chloroplastic [Source:UniProtKB/Swiss-Prot;Acc:P05466]	18677311	18681889	39.57
AT2G45310	GAE4	UDP-glucuronate 4-epimerase 4 [Source:UniProtKB/Swiss-Prot;Acc:O22141]	18682081	18684042	41.54
AT2G45315		other RNA [Source:TAIR;Acc:AT2G45315]	18682089	18684072	41.43
AT2G45320		unknown protein; FUNCTIONS IN: molecular_function unknown; INVOLVED IN: biological_process unknown; LOCATED IN: mi	18684116	18686197	39.63
AT2G45330	emb1067	RNA 2'-phosphotransferase, Tpt1 / KptA family [Source:UniProtKB/TrEMBL;Acc:F4IW51]	18685898	18688061	39.05
AT2G45340		Leucine-rich repeat protein kinase family protein [Source:UniProtKB/TrEMBL;Acc:O22138]	18691664	18694725	40.37
AT2G45350	CRR4	Pentatricopeptide repeat-containing protein At2g45350, chloroplastic [Source:UniProtKB/Swiss-Prot;Acc:O22137]	18694816	18696691	41.74
AT2G45360		Ankyrin repeat/KH domain protein (DUF1442) [Source:UniProtKB/TrEMBL;Acc:O22136]	18698592	18699514	44.1
AT2G09605			18698902	18699138	52.32
AT2G45380		FUNCTIONS IN: molecular_function unknown; INVOLVED IN: biological_process unknown; LOCATED IN: cellular_component u	18699428	18703093	35.73
AT2G09610			18702738	18703101	37.91
AT2G45390		pre-tRNA [Source:TAIR;Acc:AT2G45390]	18703678	18703750	63.01
AT2G45400	BEN1	Protein BRI1-5 ENHANCED 1 [Source:UniProtKB/Swiss-Prot;Acc:O22133]	18703772	18706401	35.51
AT2G09615			18708285	18709003	41.03
AT2G45403		unknown protein; BEST Arabidopsis thaliana protein match is: unknown protein (TAIR:AT1G34095.1); Ha. [Source:TAIR;Acc:AT2	18709301	18710153	42.56
AT2G45405			18710344	18710917	37.98
AT2G45406		Galactose oxidase/kelch repeat superfamily protein [Source:UniProtKB/TrEMBL;Acc:F4IG53]	18710961	18711548	42.86
AT2G45410	LBD19	LBD19 [Source:UniProtKB/TrEMBL;Acc:A0A178VSY1]	18712220	18713211	42.74
AT2G45420	LBD18	LBD18 [Source:UniProtKB/TrEMBL;Acc:A0A178VVX7]	18718348	18720729	37.66
AT2G09620			18724063	18724598	41.04
AT2G09625			18724177	18724523	47.26
AT2G09630			18725411	18725851	42.86
AT2G09635			18725411	18725887	42.98
AT2G45430	AHL22	AT-hook motif nuclear-localized protein [Source:UniProtKB/TrEMBL;Acc:A0A178VXD2]	18727504	18729272	42.79
AT2G45440	DHDPS2	Putative dihydrodipicolinate synthase [Source:UniProtKB/TrEMBL;Acc:Q0WSN6]	18730840	18733119	39.96
AT2G45450	ZPR1	Protein LITTLE ZIPPER 1 [Source:UniProtKB/Swiss-Prot;Acc:F4IG60]	18733275	18734341	33.93
AT2G45460		SMAD/FHA domain-containing protein [Source:UniProtKB/TrEMBL;Acc:F4IG63]	18736803	18741916	39.36
AT2G45470	FLA8	Fasciclin-like arabinogalactan protein 8 [Source:UniProtKB/Swiss-Prot;Acc:O22126]	18742477	18744322	45.83
AT2G45480	GRF9	Growth-regulating factor 9 [Source:UniProtKB/Swiss-Prot;Acc:Q8S9M3]	18745249	18747634	39.77
AT2G45490	AUR3	AUR3 [Source:UniProtKB/TrEMBL;Acc:A0A178VSR7]	18747509	18749215	39.72
AT2G45500		AAA-type ATPase family protein [Source:TAIR;Acc:AT2G45500]	18749352	18752866	36.07
AT2G45510	CYP704A2	At2g45510 [Source:UniProtKB/TrEMBL;Acc:O64631]	18753023	18755172	39.63
AT2G45520		Coiled-coil protein [Source:UniProtKB/TrEMBL;Acc:O64632]	18755045	18756302	39.03
AT2G45530		At2g45530 [Source:UniProtKB/TrEMBL;Acc:O64633]	18756471	18757513	43.72
AT2G09640			18756673	18757928	42.6
AT2G45540	BCHC2	BEACH domain-containing protein C2 [Source:UniProtKB/Swiss-Prot;Acc:F4IG73]	18757489	18772906	40.63
AT2G09645			18768025	18768224	42.5
AT2G09650			18772608	18772858	53.78
AT2G45550	CYP76C4	Cytochrome P450 76C4 [Source:UniProtKB/Swiss-Prot;Acc:O64635]	18773517	18775797	40.42
AT2G45560	CYP76C1	CYP76C1 [Source:UniProtKB/TrEMBL;Acc:A0A178VLJ2]	18776015	18778676	40.01
AT2G45570	CYP76C2	Cytochrome P450 76C2 [Source:UniProtKB/Swiss-Prot;Acc:O64637]	18779321	18781973	41.69
AT2G45580	CYP76C3	Cytochrome P450 76C3 [Source:UniProtKB/Swiss-Prot;Acc:O64638]	18782231	18784530	39.22
AT2G45590		Receptor-like serine/threonine-protein kinase At2g45590 [Source:UniProtKB/Swiss-Prot;Acc:O64639]	18785990	18789113	43.41
AT2G45600	CXE8	Probable carboxylesterase 8 [Source:UniProtKB/Swiss-Prot;Acc:O64640]	18789489	18791015	42.24
AT2G45610	CXE9	Probable carboxylesterase 9 [Source:UniProtKB/Swiss-Prot;Acc:O64641]	18791289	18792586	44.92
AT2G45620	URT1	UTP:RNA uridylyltransferase 1 [Source:UniProtKB/Swiss-Prot;Acc:O64642]	18792864	18795904	42.72
AT2G45630		D-isomer specific 2-hydroxyacid dehydrogenase family protein [Source:UniProtKB/TrEMBL;Acc:Q67Y01]	18795926	18797263	44.17
AT2G45640	SAP18	Histone deacetylase complex subunit SAP18 [Source:UniProtKB/TrEMBL;Acc:A0A178VW39]	18799528	18801471	37.14
AT2G45650	AGL6	AGAMOUS-like protein 6 [Source:UniProtKB/TrEMBL;Acc:Q1PEU3]	18804190	18806586	33.71
AT2G45660	SOC1	SOC1 [Source:UniProtKB/TrEMBL;Acc:A0A178VZL4]	18807505	18811125	32.62
AT2G09655			18814232	18814480	42.97
AT2G45670	LPEAT2	Lysophospholipid acyltransferase LPEAT2 [Source:UniProtKB/Swiss-Prot;Acc:Q8S8S2]	18814660	18818545	37.93
AT2G45680	TCP9	Transcription factor TCP9 [Source:UniProtKB/Swiss-Prot;Acc:O64647]	18820242	18821889	47.63
AT2G45685		other RNA [Source:TAIR;Acc:AT2G45685]	18820265	18821944	47.62
AT2G45690	PEX16	Peroxisome biogenesis protein 16 [Source:UniProtKB/Swiss-Prot;Acc:Q8S8S1]	18823167	18825763	38.31
AT2G45695	URM1-1	Ubiquitin-related modifier 1 homolog [Source:UniProtKB/TrEMBL;Acc:A0A178VS32]	18825954	18826899	35.84
AT2G45700		Sterile alpha motif (SAM) domain-containing protein [Source:UniProtKB/TrEMBL;Acc:O64649]	18826937	18830664	39.73
AT2G45710	RPS27A	40S ribosomal protein S27-1 [Source:UniProtKB/Swiss-Prot;Acc:O64650]	18831130	18832673	37.44
AT2G45720		ARM repeat superfamily protein [Source:UniProtKB/TrEMBL;Acc:O64651]	18833086	18836374	40.04
AT2G09660			18834709	18835461	45.15
AT2G45730		tRNA (adenine(58)-N(1))-methyltransferase non-catalytic subunit TRM6 [Source:UniProtKB/TrEMBL;Acc:O80846]	18836264	18839273	35.85
AT2G45740	PEX11D	Peroxisomal membrane protein 11D [Source:UniProtKB/Swiss-Prot;Acc:O80845]	18839435	18841403	37.18
AT2G45750		Probable methyltransferase PMT16 [Source:UniProtKB/Swiss-Prot;Acc:O80844]	18842516	18845576	41.29
AT2G45760	BAP2	BON1-associated protein 2 [Source:UniProtKB/Swiss-Prot;Acc:Q58FX0]	18846944	18847835	39.57
AT2G45770	CPFTSY	Cell division protein FtsY homolog, chloroplastic [Source:UniProtKB/Swiss-Prot;Acc:O80842]	18851088	18853741	39.98
AT2G45780		other RNA [Source:TAIR;Acc:AT2G45780]	18854555	18855184	38.1
AT2G45790	PMM	Phosphomannomutase [Source:UniProtKB/Swiss-Prot;Acc:O80840]	18855675	18858018	36.09
AT2G45800	PLIM2A	LIM domain-containing protein PLIM2a [Source:UniProtKB/Swiss-Prot;Acc:O80839]	18857941	18859278	38.19
AT2G09665			18858184	18859278	40.09
AT2G45810	RH6	DEAD-box ATP-dependent RNA helicase 6 [Source:UniProtKB/Swiss-Prot;Acc:Q94BV4]	18859472	18862970	40.07
AT2G45820	DBP	Remorin [Source:UniProtKB/Swiss-Prot;Acc:O80837]	18862953	18864741	35.77
AT2G45830	DTA2	Downstream target of AGL15 2 [Source:UniProtKB/TrEMBL;Acc:F4IH50]	18865923	18868542	37.71
AT2G45840		Glycosyltransferase [Source:UniProtKB/TrEMBL;Acc:F4IH52]	18869153	18871786	37.78
AT2G45850	AHL9	AT-hook motif nuclear-localized protein 9 [Source:UniProtKB/Swiss-Prot;Acc:O80834]	18871479	18873972	38.57
AT2G45860		At2g45860 [Source:UniProtKB/TrEMBL;Acc:O80833]	18874566	18875653	35.02
AT2G45870		UPF0187 protein At2g45870, chloroplastic [Source:UniProtKB/Swiss-Prot;Acc:O80832]	18875736	18877679	41.51
AT2G45880	BAM7	Beta-amylase 7 [Source:UniProtKB/Swiss-Prot;Acc:O80831]	18878518	18882995	41.74
AT2G45890	ROPGEF4	Rop guanine nucleotide exchange factor 4 [Source:UniProtKB/Swiss-Prot;Acc:Q0WNP7]	18883377	18885729	39.4
AT2G45900		TRM13 [Source:UniProtKB/TrEMBL;Acc:A0A384LCE0]	18885751	18889178	38.86
AT2G45910	PUB33	U-box domain-containing protein 33 [Source:UniProtKB/Swiss-Prot;Acc:Q8GUH1]	18894030	18898528	40.88
AT2G45920	PUB37	U-box domain-containing protein 37 [Source:UniProtKB/Swiss-Prot;Acc:Q683D5]	18898629	18901326	38.36
AT2G45930		Expressed protein [Source:UniProtKB/TrEMBL;Acc:O80826]	18901392	18902914	42.94
AT2G09670			18902541	18903035	43.03
AT2G45940		Protein of unknown function (DUF295) [Source:TAIR;Acc:AT2G45940]	18902963	18904325	44.97
AT2G45950	ASK20	SKP1-like protein 20 [Source:UniProtKB/Swiss-Prot;Acc:A8MQG7]	18904324	18908121	37.52
AT2G45960	PIP1B	Plasma membrane intrinsic protein 1B [Source:UniProtKB/TrEMBL;Acc:A8MRW1]	18910081	18911982	41.27
AT2G45970	CYP86A8	Cytochrome P450 86A8 [Source:UniProtKB/Swiss-Prot;Acc:O80823]	18912095	18914739	43.86
AT2G45980	ATI1	ATG8-interacting protein 1 [Source:UniProtKB/Swiss-Prot;Acc:O82775]	18917272	18919198	40.11
AT2G45990		AT2G45990 protein [Source:UniProtKB/TrEMBL;Acc:O82790]	18919316	18921511	38.43
AT2G09675			18919458	18919640	35.52
AT2G46000		At2g46000 [Source:UniProtKB/TrEMBL;Acc:O82812]	18921024	18922179	39.97

AT2G46020	BRM	ATP-dependent helicase BRM [Source:UniProtKB/Swiss-Prot;Acc:Q6EVK6]	18922533	18932055	42.35
AT2G46030	UBC6	ubiquitin-conjugating enzyme 6 [Source:TAIR;Acc:AT2G46030]	18932019	18934138	34.62
AT2G46040		ARID/BRIGHT DNA-binding domain;ELM2 domain protein [Source:TAIR;Acc:AT2G46040]	18935236	18938447	39.73
AT2G46050	PCMP-E39	Pentatricopeptide repeat-containing protein At2g46050, mitochondrial [Source:UniProtKB/Swiss-Prot;Acc:O82363]	18939262	18941034	41.79
AT2G46060		Transmembrane protein-like protein [Source:UniProtKB/TrEMBL;Acc:Q8RWX6]	18941099	18945112	39.11
AT2G46070	ATMPK12	MPK12 [Source:UniProtKB/TrEMBL;Acc:A0A178VX75]	18945937	18948067	39.75
AT2G46080		BPS2 [Source:UniProtKB/TrEMBL;Acc:A0A178VNI3]	18948116	18950120	38.95
AT2G46090	LCKB2	Sphingoid long-chain bases kinase 2, mitochondrial [Source:UniProtKB/Swiss-Prot;Acc:O82359]	18950802	18953275	40.3
AT2G46100		At2g46100 [Source:UniProtKB/TrEMBL;Acc:Q6ID84]	18953275	18954619	39.26
AT2G46110	KPHMT1	3-methyl-2-oxobutanoate hydroxymethyltransferase 1, mitochondrial [Source:UniProtKB/Swiss-Prot;Acc:O82357]	18954570	18956582	40.93
AT2G46120		pre-tRNA [Source:TAIR;Acc:AT2G46120]	18956641	18956713	61.64
AT2G46130	WRKY43	Probable WRKY transcription factor 43 [Source:UniProtKB/Swiss-Prot;Acc:Q8GY11]	18957212	18957925	33.75
AT2G46140		Desiccation-related protein At2g46140 [Source:UniProtKB/Swiss-Prot;Acc:O82355]	18958999	18960645	36.19
AT2G46150		Late embryogenesis abundant (LEA) hydroxyproline-rich glycoprotein family [Source:UniProtKB/TrEMBL;Acc:O82354]	18961283	18962423	40.4
AT2G46160	ATL67	RING-H2 finger protein ATL67 [Source:UniProtKB/Swiss-Prot;Acc:O82353]	18962743	18964689	38.88
AT2G46170	RTNLB5	Reticulon-like protein B5 [Source:UniProtKB/Swiss-Prot;Acc:O82352]	18965068	18967271	38.34
AT2G46180	GC4	Golgin candidate 4 [Source:UniProtKB/Swiss-Prot;Acc:Q8VYU6]	18967269	18971409	37.21
AT2G46190		Mitochondrial glycoprotein family protein [Source:UniProtKB/TrEMBL;Acc:O82350]	18971769	18972804	39.86
AT2G09680			18973209	18973330	32.79
AT2G46192		other RNA [Source:TAIR;Acc:AT2G46192]	18973307	18974133	37.61
AT2G09685			18973424	18973508	41.18
AT2G09690			18973544	18973705	38.27
AT2G09695			18973735	18973879	40.69
AT2G09700			18973915	18974055	41.13
AT2G09705			18974209	18974352	40.97
AT2G46200	SNRNP59	U11/U12 small nuclear ribonucleoprotein 59 kDa protein [Source:UniProtKB/Swiss-Prot;Acc:Q8VYD3]	18974367	18979061	40.75
AT2G46210	SLD2	Delta(8)-fatty-acid desaturase 2 [Source:UniProtKB/Swiss-Prot;Acc:Q3EBF7]	18977344	18979309	44.4
AT2G46220		At2g46220/T3F17.13 [Source:UniProtKB/TrEMBL;Acc:O82347]	18979399	18980940	39.62
AT2G46225	ABIL1	ABI-1-like 1 [Source:UniProtKB/TrEMBL;Acc:B3H5V3]	18981911	18984292	37.87
AT2G09710			18982224	18982649	35.92
AT2G46230		At2g46230/T3F17.12 [Source:UniProtKB/TrEMBL;Acc:O82346]	18984199	18985959	37.59
AT2G46240	BAG6	BAG family molecular chaperone regulator 6 [Source:UniProtKB/Swiss-Prot;Acc:O82345]	18986133	18990220	41.98
AT2G46250		At2g46250/T3F17.10 [Source:UniProtKB/TrEMBL;Acc:O82344]	18991171	18993371	40.94
AT2G46255	MIR159C	MIR159C; miRNA [Source:TAIR;Acc:AT2G46255]	18994632	18994856	42.67
AT2G46260		BTB/POZ domain-containing protein At2g46260 [Source:UniProtKB/Swiss-Prot;Acc:O82343]	18995645	18998672	41.08
AT2G46270	GBF3	G-box-binding factor 3 [Source:UniProtKB/Swiss-Prot;Acc:P42776]	19000180	19003403	38.34
AT2G46280	TIF3I1	Eukaryotic translation initiation factor 3 subunit I [Source:UniProtKB/Swiss-Prot;Acc:Q38884]	19003158	19005732	38.87
AT2G46290		Eukaryotic translation initiation factor 3 subunit I [Source:UniProtKB/TrEMBL;Acc:A0A178VR74]	19005771	19007952	39.6
AT2G46300		Late embryogenesis abundant (LEA) hydroxyproline-rich glycoprotein family [Source:UniProtKB/TrEMBL;Acc:Q1PEU2]	19008175	19009651	40.42
AT2G46308		unknown protein; Ha. [Source:TAIR;Acc:AT2G46308]	19011167	19011355	37.04
AT2G46310	CRF5	Ethylene-responsive transcription factor CRF5 [Source:UniProtKB/Swiss-Prot;Acc:O82339]	19011462	19013018	41.94
AT2G09715			19013563	19013785	34.08
AT2G46320		Mitochondrial substrate carrier family protein [Source:UniProtKB/TrEMBL;Acc:F4II70]	19015808	19018491	39.38
AT2G46330	AGP16	ATAGP16 [Source:UniProtKB/TrEMBL;Acc:A0A178VYP5]	19018264	19019364	38.33
AT2G46340	SPA1	SPA1 [Source:UniProtKB/TrEMBL;Acc:A0A178VSM9]	19022154	19027528	37.97
AT2G46360		At2g46360/F11C10.5 [Source:UniProtKB/TrEMBL;Acc:Q9SKE3]	19028359	19029127	35.63
AT2G46370	JAR1	Auxin-responsive GH3 family protein [Source:UniProtKB/TrEMBL;Acc:F4II77]	19033586	19036683	39.86
AT2G46375		Expressed protein [Source:UniProtKB/TrEMBL;Acc:Q8S8G5]	19037800	19038431	38.45
AT2G46380		Extra-large G-like protein, putative (DUF3133) [Source:UniProtKB/TrEMBL;Acc:Q0WVG1]	19039024	19042348	41.11
AT2G46390	SDH8	Succinate dehydrogenase subunit 8, mitochondrial [Source:UniProtKB/Swiss-Prot;Acc:Q9SKE0]	19042301	19043234	36.94
AT2G46400	WRKY46	Probable WRKY transcription factor 46 [Source:UniProtKB/Swiss-Prot;Acc:Q9SKD9]	19043411	19044955	35.92
AT2G46410	CPC	Transcription factor CPC [Source:UniProtKB/Swiss-Prot;Acc:O22059]	19049021	19050369	35.51
AT2G46420		At2g46420/F11C10.11 [Source:UniProtKB/TrEMBL;Acc:Q9SKD8]	19053470	19057162	35.77
AT2G46430	CNGC3	Probable cyclic nucleotide-gated ion channel 3 [Source:UniProtKB/Swiss-Prot;Acc:Q9SKD7]	19057999	19061492	37.03
AT2G46440	CNGC11	Cyclic nucleotide-gated ion channel 11 [Source:UniProtKB/Swiss-Prot;Acc:Q9SKD6]	19061688	19064937	39.51
AT2G46450	CNGC12	Probable cyclic nucleotide-gated ion channel 12 [Source:UniProtKB/Swiss-Prot;Acc:Q8GWD2]	19065536	19069569	39.29
AT2G46455		OxaA/YidC-like membrane insertion protein [Source:TAIR;Acc:AT2G46455]	19069385	19071083	36.9
AT2G46460		Polynucleotidyl transferase, ribonuclease H-like superfamily protein [Source:UniProtKB/TrEMBL;Acc:Q9SKD4]	19071537	19072019	42.24
AT2G46470	OXA1L	Mitochondrial inner membrane protein OXA1-like [Source:UniProtKB/Swiss-Prot;Acc:Q9SKD3]	19072277	19075124	38.48
AT2G09720			19075625	19075974	44.86
AT2G46480	GAUT2	Hexosyltransferase (Fragment) [Source:UniProtKB/TrEMBL;Acc:A0A068FL09]	19076362	19078540	40.8
AT2G46490		Uncharacterized protein At2g46490 [Source:UniProtKB/TrEMBL;Acc:Q9ZPZ0]	19079305	19080143	41.36
AT2G46493		RING/U-box superfamily protein [Source:TAIR;Acc:AT2G46493]	19080254	19081751	39.45
AT2G46494	ATL21B	Putative RING-H2 finger protein ATL21B [Source:UniProtKB/Swiss-Prot;Acc:P0CH02]	19082344	19083811	41.49
AT2G46495	ATL21A	Putative RING-H2 finger protein ATL21A [Source:UniProtKB/Swiss-Prot;Acc:P0CH01]	19083910	19085776	38.14
AT2G46500	PI4KG4	Phosphatidylinositol 4-kinase gamma 4 [Source:UniProtKB/Swiss-Prot;Acc:Q9ZPY9]	19086492	19089229	41.53
AT2G46505	SDH4	SDH4 [Source:UniProtKB/TrEMBL;Acc:A0A178VQB2]	19089486	19090776	38.42
AT2G46510	AIB	Transcription factor ABA-INDUCIBLE bHLH-TYPE [Source:UniProtKB/Swiss-Prot;Acc:Q9ZPY8]	19090953	19093373	39.69
AT2G09725			19094968	19095173	40.78
AT2G46520	CAS	Exportin-2 [Source:UniProtKB/Swiss-Prot;Acc:Q9ZPY7]	19096413	19100373	42.67
AT2G09730			19100909	19101260	33.81
AT2G46530	ARF11	Auxin response factor 11 [Source:UniProtKB/Swiss-Prot;Acc:Q9ZPY6]	19104632	19108486	36.81
AT2G46535		Uncharacterized protein At2g46535 [Source:UniProtKB/TrEMBL;Acc:Q84JE7]	19109513	19110497	40.71
AT2G46540		At2g46540/F11C10.23 [Source:UniProtKB/TrEMBL;Acc:Q9ZPY5]	19110472	19111837	37.99
AT2G46550		Expressed protein [Source:UniProtKB/TrEMBL;Acc:Q9ZPY4]	19111961	19114227	37.98
AT2G46560		Transducin family protein / WD-40 repeat family protein [Source:UniProtKB/TrEMBL;Acc:F4IJ68]	19115322	19125856	40.48
AT2G46572		other RNA [Source:TAIR;Acc:AT2G46572]	19126134	19129105	38.16
AT2G46570	LAC6	Laccase-6 [Source:UniProtKB/Swiss-Prot;Acc:Q9ZPY2]	19126272	19129069	39.21
AT2G46567		unknown protein; Ha. [Source:TAIR;Acc:AT2G46567]	19126641	19126751	42.34
AT2G46580	PPOX2	Pyridoxine/pyridoxamine 5'-phosphate oxidase 2 [Source:UniProtKB/Swiss-Prot;Acc:Q9ZPY1]	19129230	19130712	36.95
AT2G46590	DOF2.5	Dof zinc finger protein DOF2.5 [Source:UniProtKB/Swiss-Prot;Acc:Q9ZPY0]	19132925	19135383	35.22
AT2G46600	KIC	Calcium-binding protein KIC [Source:UniProtKB/Swiss-Prot;Acc:Q9ZPX9]	19136019	19136600	40.21
AT2G09735			19136029	19136127	39.39
AT2G46610	RS31A	Serine/arginine-rich splicing factor RS31A [Source:UniProtKB/Swiss-Prot;Acc:Q9ZPX8]	19136566	19138762	40.65
AT2G09740			19137869	19138359	39.71
AT2G46620		AAA-ATPase At2g46620 [Source:UniProtKB/Swiss-Prot;Acc:F4IJ77]	19138769	19140849	41.23
AT2G46630		Putative extensin [Source:UniProtKB/TrEMBL;Acc:Q9ZNU3]	19145218	19147147	41.5
AT2G46640		unknown protein; Ha. [Source:TAIR;Acc:AT2G46640]	19148662	19151080	33.77
AT2G46650	CYTB5-C	Cytochrome B5 isoform C [Source:UniProtKB/Swiss-Prot;Acc:Q9ZNV4]	19151481	19152739	36.62
AT2G46660	CYP78A6	Cytochrome P450 78A6 [Source:UniProtKB/Swiss-Prot;Acc:Q9ZNR0]	19153328	19155579	41.79
AT2G09745			19163718	19164100	41.51
AT2G46670		CCT motif family protein (Fragment) [Source:UniProtKB/TrEMBL;Acc:C0SV91]	19164357	19165233	40.14
AT2G46680	ATHB-7	Homeobox-leucine zipper protein ATHB-7 [Source:UniProtKB/Swiss-Prot;Acc:P46897]	19165274	19166967	36.95
AT2G46685	MIR166A	MIR166/MIR166A; miRNA [Source:TAIR;Acc:AT2G46685]	19176108	19176277	47.06
AT2G09750			19179082	19179319	36.55
AT2G46690	SAUR32	Auxin-responsive protein SAUR32 [Source:UniProtKB/Swiss-Prot;Acc:Q9ZUZ3]	19180705	19181600	35.6
AT2G46700	CRK3	CDPK-related kinase 3 [Source:UniProtKB/Swiss-Prot;Acc:Q9ZUZ2]	19182653	19186812	39.06
AT2G09755			19186764	19187146	29.5
AT2G46710	ROPGAP3	Rho GTPase-activating protein 3 [Source:UniProtKB/Swiss-Prot;Acc:Q8GYY5]	19191247	19195093	36.24
AT2G46720	HIC	3-ketoacyl-CoA synthase 13 [Source:UniProtKB/Swiss-Prot;Acc:Q9ZUZ0]	19197330	19200442	38.29
AT2G46735		At2g46730/F19D11.1 [Source:UniProtKB/TrEMBL;Acc:Q8VYU9]	19204185	19205136	42.44
AT2G46740	GULLO5	GulLO5 [Source:UniProtKB/TrEMBL;Acc:A0A178VXG4]	19205157	19207608	39.52
AT2G46750	GULLO2	L-gulonolactone oxidase 2 [Source:UniProtKB/Swiss-Prot;Acc:Q6NQ66]	19207991	19211021	38.57
AT2G46760	GULLO6	Probable L-gulonolactone oxidase 6 [Source:UniProtKB/Swiss-Prot;Acc:O81032]	19212881	19215210	41.42
AT2G46765			19220194	19220586	43.26

AT2G46770	NAC043	NAC domain-containing protein 43 [Source:UniProtKB/Swiss-Prot;Acc:Q84WP6]	19220725	19222916	37
AT2G46780		At2g46780 [Source:UniProtKB/TrEMBL;Acc:Q501B0]	19229070	19231539	36.84
AT2G46790	APRR9	Two-component response regulator-like APRR9 [Source:UniProtKB/Swiss-Prot;Acc:Q8L500]	19232607	19235179	37.58
AT2G46800	MTP1	Metal tolerance protein 1 [Source:UniProtKB/Swiss-Prot;Acc:Q9ZT63]	19235374	19239577	33.99
AT2G46810	BHLH70	Transcription factor bHLH70 [Source:UniProtKB/Swiss-Prot;Acc:O81037]	19239694	19242772	37.03
AT2G46820	CURT1B	Protein CURVATURE THYLAKOID 1B, chloroplastic [Source:UniProtKB/Swiss-Prot;Acc:Q8LCA1]	19243348	19245141	37.9
AT2G46830	CCA1	Protein CCA1 [Source:UniProtKB/Swiss-Prot;Acc:P92973]	19245591	19248915	38.92
AT2G46840	DUF4	DUF4 [Source:UniProtKB/TrEMBL;Acc:A0A178VVQ8]	19248971	19249909	43.13
AT2G46850		Probably inactive receptor-like protein kinase At2g46850 [Source:UniProtKB/Swiss-Prot;Acc:Q8S8N4]	19251066	19253484	41.67
AT2G46860	PPA3	PPa3 [Source:UniProtKB/TrEMBL;Acc:A0A178VZ15]	19253670	19255328	37.43
AT2G46870	NGA1	B3 domain-containing transcription factor NGA1 [Source:UniProtKB/Swiss-Prot;Acc:O82799]	19260906	19262533	38.57
AT2G09760			19262657	19263193	33.15
AT2G46880	PAP14	Probable inactive purple acid phosphatase 14 [Source:UniProtKB/Swiss-Prot;Acc:Q84LR6]	19264573	19266456	38.96
AT2G46890		3-oxo-5-alpha-steroid 4-dehydrogenase (DUF1295) [Source:UniProtKB/TrEMBL;Acc:O81042]	19266664	19268195	40.34
AT2G46900		Expressed protein [Source:UniProtKB/TrEMBL;Acc:O80734]	19269558	19272263	41.61
AT2G46910	PAP10	Probable plastid-lipid-associated protein 10, chloroplastic [Source:UniProtKB/Swiss-Prot;Acc:Q8W4F1]	19272281	19274002	39.61
AT2G46915		Protein of unknown function (DUF3754) [Source:TAIR;Acc:AT2G46915]	19273899	19277773	38.84
AT2G46920	POL	Protein phosphatase 2C 32 [Source:UniProtKB/Swiss-Prot;Acc:Q8RWN7]	19277884	19281811	41.6
AT2G46930	PAE3	Pectin acetylesterase 3 [Source:UniProtKB/Swiss-Prot;Acc:O80731]	19283456	19286400	38.95
AT2G46940		unknown protein; BEST Arabidopsis thaliana protein match is: unknown protein (TAIR:AT3G62070.1); Ha. [Source:TAIR;Acc:AT2	19286434	19287590	40.19
AT2G09765			19288868	19289103	37.71
AT2G46950	CYP709B2	Cytochrome P450 709B2 [Source:UniProtKB/Swiss-Prot;Acc:F4IK45]	19289087	19291632	40.06
AT2G46960	CYP709B1	Cytochrome P450 709B1 [Source:UniProtKB/Swiss-Prot;Acc:Q9ASR3]	19292035	19294375	39.98
AT2G46970	PIL1	Transcription factor PIL1 [Source:UniProtKB/Swiss-Prot;Acc:Q8L5W8]	19295431	19297858	33.48
AT2G46980	ASY3	Meiosis-specific protein ASY3 [Source:UniProtKB/Swiss-Prot;Acc:Q0WR66]	19299492	19304271	38.51
AT2G09770			19304256	19304523	45.52
AT2G46990	IAA20	Auxin-responsive protein [Source:UniProtKB/TrEMBL;Acc:A0A178W181]	19307714	19309071	35.42
AT2G46995		unknown protein; LOCATED IN: endomembrane system; Ha. [Source:TAIR;Acc:AT2G46995]	19309394	19309673	42.14
AT2G47000	ABCB4	ABC transporter B family member 4 [Source:UniProtKB/Swiss-Prot;Acc:O80725]	19309643	19315343	40.34
AT2G09775			19315192	19315401	31.9
AT2G09780			19316345	19316551	37.68
AT2G47010		At2g47010/F14M4.16 [Source:UniProtKB/TrEMBL;Acc:Q944A3]	19317126	19319397	41.46
AT2G47020		Peptide chain release factor 1 [Source:TAIR;Acc:AT2G47020]	19319685	19322507	39.25
AT2G47015	MIR408	MIR408; miRNA [Source:TAIR;Acc:AT2G47015]	19319814	19320031	39.91
AT2G47030	PME4	Pectinesterase 4 [Source:UniProtKB/Swiss-Prot;Acc:O80722]	19324134	19326484	43.77
AT2G47040	PME5	Pectinesterase 5 [Source:UniProtKB/Swiss-Prot;Acc:Q5MFV8]	19327853	19330197	43.16
AT2G47050		Invertase/pectin methylesterase inhibitor family protein [Source:UniProtKB/TrEMBL;Acc:O80720]	19331545	19332659	38.12
AT2G47060		Protein kinase superfamily protein [Source:TAIR;Acc:AT2G47060]	19332825	19335163	37.71
AT2G47070	SPL1	Squamosa promoter-binding-like protein 1 [Source:UniProtKB/Swiss-Prot;Acc:Q9SMX9]	19336653	19340869	40.95
AT2G47090		Zinc ion binding/nucleic acid binding protein [Source:UniProtKB/TrEMBL;Acc:F4IK69]	19341225	19344343	40.62
AT2G47100		pre-tRNA [Source:TAIR;Acc:AT2G47100]	19344427	19344499	60.27
AT2G47110	RPS27AB	UBQ6 [Source:UniProtKB/TrEMBL;Acc:A0A178VYW3]	19344635	19345345	43.04
AT2G09785			19345150	19345317	30.95
AT2G47115		unknown protein; FUNCTIONS IN: molecular_function unknown; INVOLVED IN: biological_process unknown; LOCATED IN: en	19345350	19346921	39.95
AT2G47120		NAD(P)-binding Rossmann-fold superfamily protein [Source:TAIR;Acc:AT2G47120]	19347239	19348362	45.11
AT2G47130	SDR3A	Short-chain dehydrogenase reductase 3a [Source:UniProtKB/Swiss-Prot;Acc:O80713]	19349348	19350650	43.44
AT2G47140	SDR3B	Short-chain dehydrogenase reductase 3b [Source:UniProtKB/Swiss-Prot;Acc:Q94K41]	19350672	19352088	41
AT2G47150		NAD(P)-binding Rossmann-fold superfamily protein [Source:UniProtKB/TrEMBL;Acc:O80711]	19352324	19353114	50.7
AT2G47160	BOR1	HCO3-transporter family [Source:UniProtKB/TrEMBL;Acc:A8MS82]	19357420	19361328	39.55
AT2G47170	ARF1	ADP-ribosylation factor 1 [Source:UniProtKB/Swiss-Prot;Acc:P36397]	19366596	19368840	38.4
AT2G09805			19366758	19367274	40.04
AT2G09815			19367708	19367822	34.78
AT2G47180	GOLS1	Galactinol synthase 1 [Source:UniProtKB/Swiss-Prot;Acc:O22893]	19368798	19370478	43.55
AT2G09825			19374524	19374875	36.08
AT2G47190	ATMYB2	ATMYB2 [Source:UniProtKB/TrEMBL;Acc:Q39028]	19375985	19377543	37.14
AT2G47200		Uncharacterized protein At2g47200 [Source:UniProtKB/TrEMBL;Acc:O22894]	19377575	19378331	36.86
AT2G47210	SWC4	SWR1-complex protein 4 [Source:UniProtKB/Swiss-Prot;Acc:Q8VZL6]	19377719	19382436	38.11
AT2G09835			19383151	19383358	38.46
AT2G47220	DUF5	DOMAIN OF UNKNOWN FUNCTION 724 5 [Source:TAIR;Acc:AT2G47220]	19383743	19386954	39.85
AT2G47230	DUF6	DOMAIN OF UNKNOWN FUNCTION 724 6 [Source:TAIR;Acc:AT2G47230]	19386966	19390234	39.4
AT2G47240	LACS1	Long chain acyl-CoA synthetase 1 [Source:UniProtKB/Swiss-Prot;Acc:O22898]	19392497	19397907	34.5
AT2G47245		Long chain acyl-CoA synthetase [Source:UniProtKB/TrEMBL;Acc:A0A1P8B2A8]	19398184	19399097	33.26
AT2G47250		Probable pre-mRNA-splicing factor ATP-dependent RNA helicase DEAH3 [Source:UniProtKB/Swiss-Prot;Acc:O22899]	19399648	19403044	41.92
AT2G47260	WRKY23	WRKY transcription factor 23 [Source:UniProtKB/Swiss-Prot;Acc:O22900]	19404802	19407098	37.35
AT2G47270	UPB1	UPB1 [Source:UniProtKB/TrEMBL;Acc:A0A178VV95]	19411430	19412328	33.82
AT2G47275	MIR403	MIR403; miRNA [Source:TAIR;Acc:AT2G47275]	19415052	19415186	31.85
AT2G09845			19415680	19415903	29.91
AT2G47280	PME66	Probable pectinesterase 66 [Source:UniProtKB/Swiss-Prot;Acc:Q4PSQ5]	19416775	19418383	38.41
AT2G47300		Ribonuclease Ps [Source:UniProtKB/TrEMBL;Acc:F4IL30]	19418443	19422241	39.22
AT2G09875			19422804	19423152	43.84
AT2G47310		At2g47310 [Source:UniProtKB/TrEMBL;Acc:Q6AWW6]	19423589	19427277	38.19
AT2G47320	CYP21-3	Peptidyl-prolyl cis-trans isomerase CYP21-3, mitochondrial [Source:UniProtKB/Swiss-Prot;Acc:Q94A16]	19427316	19428933	38.2
AT2G47330	RH24	DEAD-box ATP-dependent RNA helicase 24 [Source:UniProtKB/Swiss-Prot;Acc:O22907]	19428897	19431821	42.84
AT2G47340		Plant invertase/pectin methylesterase inhibitor superfamily protein [Source:UniProtKB/TrEMBL;Acc:O22908]	19432147	19433156	44.36
AT2G47350		At2g47350 [Source:UniProtKB/TrEMBL;Acc:O22909]	19433725	19437544	39.37
AT2G47360		Transmembrane protein [Source:UniProtKB/TrEMBL;Acc:O22910]	19437648	19438920	40.93
AT2G47370		At2g47370 [Source:UniProtKB/TrEMBL;Acc:O22911]	19438951	19440487	42.88
AT2G47380		Probable cytochrome c oxidase subunit 5C-1 [Source:UniProtKB/Swiss-Prot;Acc:O22912]	19440923	19442053	37.14
AT2G09885			19440926	19441694	36.15
AT2G47390		Prolyl oligopeptidase family protein [Source:TAIR;Acc:AT2G47390]	19441992	19446365	41.43
AT2G09895			19445346	19445616	41.7
AT2G47400	CP12-1	Calvin cycle protein CP12-1, chloroplastic [Source:UniProtKB/Swiss-Prot;Acc:O22914]	19446730	19447642	41.07
AT2G47410		WD40/YVTN repeat-like-containing domain;Bromodomain [Source:TAIR;Acc:AT2G47410]	19448139	19457303	39.16
AT2G47420	DIM1A	rRNA adenine N(6)-methyltransferase [Source:UniProtKB/TrEMBL;Acc:A0A178VWJ8]	19457374	19458924	42.75
AT2G47430	CKI1	Histidine kinase CKI1 [Source:UniProtKB/Swiss-Prot;Acc:O22267]	19459045	19464087	38.79
AT2G47440		Tetratricopeptide repeat (TPR)-like superfamily protein [Source:UniProtKB/TrEMBL;Acc:O22266]	19469571	19472006	43.72
AT2G47450	CAO	Signal recognition particle 43 kDa protein, chloroplastic [Source:UniProtKB/Swiss-Prot;Acc:O22265]	19472573	19474387	45.84
AT2G47460	MYB12	Transcription factor MYB12 [Source:UniProtKB/Swiss-Prot;Acc:O22264]	19476326	19479597	35.39
AT2G47470	PDIL2-1	UNE5 [Source:UniProtKB/TrEMBL;Acc:A0A178W1F8]	19481254	19484015	38.02
AT2G47480		DUF3511 domain protein, putative (DUF3511) [Source:UniProtKB/TrEMBL;Acc:O22262]	19483919	19484710	40.15
AT2G09905			19484345	19484679	46.87
AT2G47485		At2g47485 [Source:UniProtKB/TrEMBL;Acc:Q6DBG0]	19485809	19486833	39.51
AT2G47490	NDT1	Nicotinamide adenine dinucleotide transporter 1, chloroplastic [Source:UniProtKB/Swiss-Prot;Acc:O22261]	19486842	19489732	38.08
AT2G09915			19491160	19491620	42.3
AT2G47500	KIN14I	Kinesin-like protein KIN-14I [Source:UniProtKB/Swiss-Prot;Acc:F4IL57]	19492867	19498189	38.47
AT2G47510	FUM1	FUM1 [Source:UniProtKB/TrEMBL;Acc:A0A178VMD4]	19498229	19502488	39.51
AT2G47520	ERF071	Ethylene-responsive transcription factor ERF071 [Source:UniProtKB/Swiss-Prot;Acc:O22259]	19502776	19503702	41.53
AT2G47530		Pollen Ole e 1 allergen and extensin family protein [Source:UniProtKB/TrEMBL;Acc:O22258]	19504171	19505048	43.28
AT2G47540		Pollen Ole e 1 allergen and extensin family protein [Source:UniProtKB/TrEMBL;Acc:O22257]	19505827	19506681	40.35
AT2G47550	PME20	Probable pectinesterase/pectinesterase inhibitor 20 [Source:UniProtKB/Swiss-Prot;Acc:O22256]	19508929	19511792	39.21
AT2G09925			19510548	19510832	34.74
AT2G47560	ATL64	RING-H2 finger protein ATL64 [Source:UniProtKB/Swiss-Prot;Acc:O22255]	19511717	19512728	46.94
AT2G09935			19512021	19512259	49.79
AT2G47570		Ribosomal protein L18e/L15 superfamily protein [Source:TAIR;Acc:AT2G47570]	19515764	19516922	40.21
AT2G47580	U1A	U1A [Source:UniProtKB/TrEMBL;Acc:A0A178VZ67]	19517187	19518913	39.26

	AT2G09945			19517593	19518626	40.72
	AT2G47585	MIR164A	MIR164/MIR164A; miRNA [Source:TAIR;Acc:AT2G47585]	19520752	19520864	46.9
	AT2G47590	PHR2	At2g47590 [Source:UniProtKB/TrEMBL;Acc:Q058P5]	19521774	19524095	44.32
	AT2G47600	MHX	Magnesium/proton exchanger [Source:UniProtKB/Swiss-Prot;Acc:O22252]	19524117	19527580	38.71
	AT2G47610	RPL7AA	60S ribosomal protein L7a-1 [Source:UniProtKB/Swiss-Prot;Acc:P49692]	19529743	19531677	40.1
	AT2G47620	SWI3A	SWI/SNF complex subunit SWI3A [Source:UniProtKB/Swiss-Prot;Acc:Q8W475]	19531939	19534471	41.18
	AT2G47630		Alpha/beta-Hydrolases superfamily protein [Source:UniProtKB/TrEMBL;Acc:O22248]	19534473	19537074	38.05
	AT2G47640		Putative small nuclear ribonucleoprotein D2 [Source:UniProtKB/TrEMBL;Acc:Q8RUH0]	19537090	19538820	38.53
	AT2G47650	UXS4	AT2G47650 protein [Source:UniProtKB/TrEMBL;Acc:C0Z2I3]	19538609	19541585	42.02
	AT2G47670	PMEI6	Pectinesterase inhibitor 6 [Source:UniProtKB/Swiss-Prot;Acc:O22244]	19543921	19544910	44.34
	AT2G47680		DExH-box ATP-dependent RNA helicase DExH8 [Source:UniProtKB/Swiss-Prot;Acc:O22243]	19545656	19551171	40.74
	AT2G47690		NADH dehydrogenase [ubiquinone] iron-sulfur protein 5-A [Source:UniProtKB/Swiss-Prot;Acc:O82238]	19551195	19552358	39.69
	AT2G47700	RFI2	E3 ubiquitin-protein ligase RFI2 [Source:UniProtKB/Swiss-Prot;Acc:O82239]	19552314	19554647	43.87
	AT2G47710		Adenine nucleotide alpha hydrolases-like superfamily protein [Source:UniProtKB/TrEMBL;Acc:O82240]	19554724	19556126	39.56
	AT2G47730	GSTF8	Glutathione S-transferase F8, chloroplastic [Source:UniProtKB/Swiss-Prot;Acc:Q96266]	19556326	19559485	38.64
	AT2G47720		Uncharacterized protein At2g47720 [Source:UniProtKB/TrEMBL;Acc:O82241]	19556624	19557139	43.41
	AT2G47740		pre-tRNA [Source:TAIR;Acc:AT2G47740]	19559855	19559925	56.34
	AT2G47750	GH3.9	Putative indole-3-acetic acid-amido synthetase GH3.9 [Source:UniProtKB/Swiss-Prot;Acc:O82243]	19560127	19563960	40.66
MQTL-5/Chr2	AT2G39010	PIP2-6	Probable aquaporin PIP2-6 [Source:UniProtKB/Swiss-Prot;Acc:Q9ZV07]	16291280	16294101	37.07
	AT2G39020		Probable acetyltransferase NATA1-like [Source:UniProtKB/Swiss-Prot;Acc:Q9ZV06]	16295140	16296495	41.67
	AT2G39030	NATA1	L-ornithine N5-acetyltransferase NATA1 [Source:UniProtKB/Swiss-Prot;Acc:Q9ZV05]	16298110	16299205	43.07
	AT2G39040	PER24	Peroxidase 24 [Source:UniProtKB/Swiss-Prot;Acc:Q9ZV04]	16299242	16301245	38.12
	AT2G39050	EULS3	Ricin B-like lectin EULS3 [Source:UniProtKB/Swiss-Prot;Acc:Q945P1]	16303295	16305633	40.27
	AT2G08995			16305974	16306173	43
	AT2G39060	SWEET9	Bidirectional sugar transporter SWEET [Source:UniProtKB/TrEMBL;Acc:A0A178VR23]	16306491	16308362	34.13
	AT2G39080		NAD(P)-binding Rossmann-fold superfamily protein [Source:UniProtKB/TrEMBL;Acc:Q8VZB1]	16309798	16312622	37.24
	AT2G39090	APC7	AtAPC7 [Source:UniProtKB/TrEMBL;Acc:A0A178VSZ9]	16312657	16317390	36.52
	AT2G39100		Putative RING zinc finger protein [Source:UniProtKB/TrEMBL;Acc:Q8VZ73]	16317356	16319387	39.37
	AT2G39110		Protein kinase superfamily protein [Source:UniProtKB/TrEMBL;Acc:Q0WSF6]	16319447	16321685	40.11
	AT2G39120	WTF9	Protein WHAT'S THIS FACTOR 9, mitochondrial [Source:UniProtKB/Swiss-Prot;Acc:Q9ZUZ6]	16321721	16322957	39.45
	AT2G09000			16321728	16321921	44.85
	AT2G39130	AVT1C	Amino acid transporter AVT1C [Source:UniProtKB/Swiss-Prot;Acc:F4IUW3]	16322956	16327312	36.52
	AT2G09005			16328852	16329115	32.58
	AT2G39140	SVR1	Putative ribosomal large subunit pseudouridine synthase SVR1, chloroplastic [Source:UniProtKB/Swiss-Prot;Acc:Q8L960]	16330129	16333405	37.41
	AT2G39160		Uncharacterized protein At2g39160 [Source:UniProtKB/TrEMBL;Acc:O80965]	16333490	16334173	34.5
	AT2G39170		MEF2BNB-like protein [Source:UniProtKB/TrEMBL;Acc:O80964]	16334546	16336297	38.13
	AT2G09010			16339187	16339531	40
	AT2G39175	MIR160A	MIR160/MIR160A; miRNA [Source:TAIR;Acc:AT2G39175]	16340279	16340363	54.12
	AT2G39180	CCR2	Serine/threonine-protein kinase-like protein CCR2 [Source:UniProtKB/Swiss-Prot;Acc:O80963]	16344151	16346769	41.5
	AT2G39190	ATATH8	Protein kinase superfamily protein [Source:UniProtKB/TrEMBL;Acc:O80962]	16349968	16356097	38.14
	AT2G39200	MLO12	MLO-like protein [Source:UniProtKB/TrEMBL;Acc:A0A178VNS1]	16356041	16359943	33.54
	AT2G39210		At2g39210/T16B24.15 [Source:UniProtKB/TrEMBL;Acc:O80960]	16366102	16368570	42.08
	AT2G39220	PLP6	Patatin-like protein 6 [Source:UniProtKB/Swiss-Prot;Acc:O80959]	16374881	16377168	40.95
	AT2G09015			16379256	16379461	30.1
	AT2G09020			16379294	16379534	33.2
	AT2G39230	LOJ	Pentatricopeptide repeat-containing protein At2g39230, mitochondrial [Source:UniProtKB/Swiss-Prot;Acc:O80958]	16381565	16384730	39.48
	AT2G39240		RNA polymerase I specific transcription initiation factor RRN3 family protein [Source:UniProtKB/TrEMBL;Acc:Q1PEV9]	16384786	16388502	38.96
	AT2G39250	SNZ	AP2-like ethylene-responsive transcription factor SNZ [Source:UniProtKB/Swiss-Prot;Acc:Q6PV67]	16388691	16391176	35.08
	AT2G39260	UPF2	Regulator of nonsense transcripts UPF2 [Source:UniProtKB/Swiss-Prot;Acc:F4IUX6]	16391924	16399812	37.93
	AT2G39270		Probable adenylate kinase 6, chloroplastic [Source:UniProtKB/Swiss-Prot;Acc:Q84JF7]	16399969	16401671	40.4
	AT2G09025			16400296	16400711	35.82
	AT2G39280		Ypt/Rab-GAP domain of gyp1p superfamily protein [Source:UniProtKB/TrEMBL;Acc:F4IUX9]	16401526	16406886	38.56
	AT2G39290	PGPS1	PGS1 [Source:UniProtKB/TrEMBL;Acc:A0A178VXZ6]	16407135	16409156	39.22
	AT2G39300		unknown protein; INVOLVED IN: biological_process unknown; LOCATED IN: vacuole; EXPRESSED IN: 8 plant structures; EXPI	16409539	16413874	38.51
	AT2G39310	JAL22	Jacalin-related lectin 22 [Source:UniProtKB/Swiss-Prot;Acc:O80950]	16414034	16416845	36.31
	AT2G39320		Cysteine proteinases superfamily protein [Source:UniProtKB/TrEMBL;Acc:O80949]	16417477	16418517	35.54
	AT2G39330	JAL23	jacalin-related lectin 23 [Source:TAIR;Acc:AT2G39330]	16419483	16422158	38.12
	AT2G39340	SAC3A	SAC3 family protein A [Source:UniProtKB/Swiss-Prot;Acc:F4IUY8]	16423541	16430108	38.52
	AT2G09035			16428857	16429040	33.7
	AT2G39350	ABCG1	ABC transporter G family member 1 [Source:UniProtKB/Swiss-Prot;Acc:O80946]	16430085	16432751	43.46
	AT2G09040			16435909	16436561	39.97
	AT2G39355		F-box/associated interaction domain protein [Source:UniProtKB/TrEMBL;Acc:A0A1P8AY04]	16436504	16436833	39.39
	AT2G39360		Probable receptor-like protein kinase At2g39360 [Source:UniProtKB/Swiss-Prot;Acc:O80623]	16437338	16440544	38.42
	AT2G39370	MAKR4	Probable membrane-associated kinase regulator 4 [Source:UniProtKB/Swiss-Prot;Acc:O80624]	16443996	16445408	40.41
	AT2G39375			16446514	16449180	39.41
	AT2G39380	ATEXO70H2	Exocyst subunit Exo70 family protein [Source:UniProtKB/TrEMBL;Acc:O80625]	16447006	16449083	41.53
	AT2G39390	RPL35B	60S ribosomal protein L35-2 [Source:UniProtKB/Swiss-Prot;Acc:O80626]	16450554	16451878	35.77
	AT2G39400		Alpha/beta-Hydrolases superfamily protein [Source:UniProtKB/TrEMBL;Acc:O80627]	16452612	16455001	33.81
	AT2G39410		Alpha/beta-Hydrolases superfamily protein [Source:UniProtKB/TrEMBL;Acc:O80628]	16455569	16458363	32.13
	AT2G39415		Putative F-box protein At2g39415 [Source:UniProtKB/Swiss-Prot;Acc:Q3EBJ8]	16458607	16459014	44.36
	AT2G39420		Alpha/beta-Hydrolases superfamily protein [Source:UniProtKB/TrEMBL;Acc:Q8RXN7]	16460247	16463220	33.93
	AT2G09045			16463300	16463441	35.92
	AT2G39430	DIR9	Dirigent protein 9 [Source:UniProtKB/Swiss-Prot;Acc:O80630]	16463347	16464531	46.92
	AT2G39435		Phosphatidylinositol N-acetylglucosaminyltransferase subunit P-like protein [Source:UniProtKB/TrEMBL;Acc:Q0WT61]	16464414	16467033	36.98
	AT2G39440		Ribonuclease H2 subunit C-like protein [Source:UniProtKB/TrEMBL;Acc:F4IVV8]	16467830	16469628	35.8
	AT2G39445		Phosphatidylinositol N-acetylglucosaminyltransferase, GPI19/PIG-P subunit [Source:UniProtKB/TrEMBL;Acc:B3H5S3]	16469779	16471274	38.3
	AT2G39450	MTP11	MTP11 [Source:UniProtKB/TrEMBL;Acc:A0A178VPD8]	16471560	16474060	39.26
	AT2G39460	RPL23AA	60S ribosomal protein L23a-1 [Source:UniProtKB/Swiss-Prot;Acc:Q8LD46]	16474149	16476141	36.03
	AT2G39470	PNSL1	Photosynthetic NDH subunit of lumenal location 1, chloroplastic [Source:UniProtKB/Swiss-Prot;Acc:O80634]	16476198	16477843	37.36
	AT2G39480	ABCB6	ABC transporter B family member 6 [Source:UniProtKB/Swiss-Prot;Acc:Q8LPT1]	16477792	16485039	40.27
	AT2G09050			16485621	16486092	38.35
	AT2G39490		F-box protein At2g39490 [Source:UniProtKB/Swiss-Prot;Acc:Q5S4V7]	16486902	16488645	38.7
	AT2G39500		At2g39500/F12L6.16 [Source:UniProtKB/TrEMBL;Acc:O80637]	16489013	16490108	36.04
	AT2G09055			16489714	16490023	35.16
	AT2G39510		WAT1-related protein At2g39510 [Source:UniProtKB/Swiss-Prot;Acc:O80638]	16491143	16493343	35.07
	AT2G39518		CASP-like protein 4D2 [Source:UniProtKB/Swiss-Prot;Acc:Q56X75]	16495819	16497145	33.01
	AT2G39520		Uncharacterized protein At2g39520 [Source:UniProtKB/TrEMBL;Acc:O80639]	16496813	16497621	35.48
	AT2G39530		CASP-like protein 4D1 [Source:UniProtKB/Swiss-Prot;Acc:Q8GWD5]	16498364	16499524	36.09
	AT2G39540		Gibberellin-regulated protein 8 [Source:UniProtKB/Swiss-Prot;Acc:O80641]	16500788	16501414	33.49
	AT2G39550	GGB	PGGT-I [Source:UniProtKB/TrEMBL;Acc:A0A178VS16]	16501571	16504379	37.56
	AT2G39560		At2g39560/F12L6.22 [Source:UniProtKB/TrEMBL;Acc:O80643]	16504838	16506150	38.31
	AT2G39570	ACR9	ACT domain-containing protein ACR9 [Source:UniProtKB/Swiss-Prot;Acc:O80644]	16507882	16510264	39.57
	AT2G39580		CONTAINS InterPro DOMAIN/s: Putative zinc-finger domain (InterPro:IPR019607); Ha. [Source:TAIR;Acc:AT2G39580]	16510273	16517200	39.55
	AT2G39590	RPS15AC	40S ribosomal protein S15a-3 [Source:UniProtKB/Swiss-Prot;Acc:O80646]	16517588	16518247	37.73
	AT2G39600		pre-tRNA [Source:TAIR;Acc:AT2G39600]	16518564	16518635	51.39
	AT2G39610		pre-tRNA [Source:TAIR;Acc:AT2G39610]	16518769	16518840	51.39
	AT2G39620	PCMP-E33	Pentatricopeptide repeat-containing protein At2g39620 [Source:UniProtKB/Swiss-Prot;Acc:O80647]	16518890	16521544	41.17
	AT2G39630		At2g39630/F12L6.29 [Source:UniProtKB/TrEMBL;Acc:Q9SLN0]	16521736	16524783	36.91
	AT2G39640		Glycosyl hydrolase family 17 protein [Source:UniProtKB/TrEMBL;Acc:O48812]	16525086	16527038	40.91
	AT2G39650		At2g39650 [Source:UniProtKB/TrEMBL;Acc:O48813]	16527730	16529494	40.11
	AT2G39660	BIK1	BIK1 [Source:UniProtKB/TrEMBL;Acc:A0A178VYW0]	16531527	16533986	40.28
	AT2G39670		Radical SAM superfamily protein [Source:UniProtKB/TrEMBL;Acc:F4IVY6]	16534175	16537318	38.96
	AT2G39675	TAS1C	TAS1C; other RNA [Source:TAIR;Acc:AT2G39675]	16537288	16538277	35.25
	AT2G39681	TAS2	TAS2; other RNA [Source:TAIR;Acc:AT2G39681]	16539384	16540417	33.95
	AT2G39690		Protein of unknown function, DUF547 [Source:TAIR;Acc:AT2G39690]	16540927	16543411	38.59

AT2G39700	EXPA4	Expansin-A4 [Source:UniProtKB/Swiss-Prot;Acc:O48818]	16543832	16545746	38.8
AT2G09060			16546752	16547255	36.11
AT2G09065			16546787	16547100	39.49
AT2G09070			16553373	16553755	45.43
AT2G09075			16553405	16553907	41.75
AT2G39705	RTFL8	RTFL8 [Source:UniProtKB/TrEMBL;Acc:A0A178VQG8]	16556801	16558268	32.97
AT2G39710		Eukaryotic aspartyl protease family protein [Source:UniProtKB/TrEMBL;Acc:O22282]	16561788	16563441	43.23
AT2G39720	RHC2A	Probable E3 ubiquitin-protein ligase RHC2A [Source:UniProtKB/Swiss-Prot;Acc:O22283]	16566586	16568960	41.14
AT2G39725		Expressed protein [Source:UniProtKB/TrEMBL;Acc:Q8VZU1]	16569430	16570748	35.63
AT2G39730	RCA	Ribulose bisphosphate carboxylase/oxygenase activase, chloroplastic [Source:UniProtKB/Swiss-Prot;Acc:P10896]	16570746	16573692	41.81
AT2G39740	HESO1	Protein HESO1 [Source:UniProtKB/Swiss-Prot;Acc:Q5XET5]	16575752	16579120	40.69
AT2G39750		Probable methyltransferase PMT11 [Source:UniProtKB/Swiss-Prot;Acc:O22285]	16578824	16582436	40.24
AT2G39760	BPM3	BTB/POZ and MATH domain-containing protein 3 [Source:UniProtKB/Swiss-Prot;Acc:O22286]	16583003	16586260	37.45
AT2G09080			16587970	16588110	26.95
AT2G39770	CYT1	VTC1 [Source:UniProtKB/TrEMBL;Acc:A0A178VW16]	16588556	16591032	38.64
AT2G39780	RNS2	Ribonuclease 2 [Source:UniProtKB/Swiss-Prot;Acc:P42814]	16591117	16593775	35.69
AT2G39782		unknown protein; FUNCTIONS IN: molecular_function unknown; INVOLVED IN: biological_process unknown; LOCATED IN: cel	16594037	16594516	38.96
AT2G39790		Uncharacterized protein At2g39790, mitochondrial [Source:UniProtKB/Swiss-Prot;Acc:O22288]	16594833	16595800	40.6
AT2G39795		Uncharacterized protein At2g39795, mitochondrial [Source:UniProtKB/Swiss-Prot;Acc:Q8W487]	16596838	16598162	40
AT2G39800	P5CSA	Delta-1-pyrroline-5-carboxylate synthase A [Source:UniProtKB/Swiss-Prot;Acc:P54887]	16598072	16603319	38.64
AT2G39805		Protein YIPF [Source:UniProtKB/TrEMBL;Acc:A0A178VTF3]	16609797	16612554	36.26
AT2G39810	HOS1	HOS1 [Source:UniProtKB/TrEMBL;Acc:A0A178W236]	16612777	16618122	39.11
AT2G39820	EIF6-1	Eukaryotic translation initiation factor 6-1 [Source:UniProtKB/Swiss-Prot;Acc:O22290]	16618226	16619750	37.05
AT2G39830	DAR2	DA1-related protein 2 [Source:TAIR;Acc:AT2G39830]	16619743	16624150	34.69
AT2G39840	TOPP4	Serine/threonine-protein phosphatase [Source:UniProtKB/TrEMBL;Acc:A0A178VUA1]	16627216	16629412	38.55
AT2G39850	SBT4.1	Subtilisin-like protease SBT4.1 [Source:UniProtKB/Swiss-Prot;Acc:F4IG09]	16630411	16634253	39.01
AT2G39851		Proteinase inhibitor, propeptide [Source:UniProtKB/TrEMBL;Acc:B3H4B7]	16634519	16635568	32.67
AT2G39855		Plant/protein [Source:UniProtKB/TrEMBL;Acc:Q84WZ4]	16637661	16639497	32.83
AT2G39860		pre-tRNA [Source:TAIR;Acc:AT2G39860]	16639780	16639851	61.11
AT2G39865		unknown protein; Ha. [Source:TAIR;Acc:AT2G39865]	16641761	16641907	46.26
AT2G39870		At2g39870/T5I7.17 [Source:UniProtKB/TrEMBL;Acc:O04191]	16643381	16645866	39.26
AT2G39880	MYB25	Transcription factor MYB25 [Source:UniProtKB/Swiss-Prot;Acc:O04192]	16647711	16649527	39.46
AT2G39885	MIR393A	MIR393A; miRNA [Source:TAIR;Acc:AT2G39885]	16652101	16652233	35.34
AT2G09085			16653249	16653524	43.84
AT2G39890	PROT1	Proline transporter 1 [Source:UniProtKB/Swiss-Prot;Acc:P92961]	16655577	16658412	36.57
AT2G39900	WLIN2A	WLIM2a [Source:UniProtKB/TrEMBL;Acc:A0A178VU35]	16658468	16660443	36.54
AT2G39910		Uncharacterized protein At2g39910 [Source:UniProtKB/Swiss-Prot;Acc:Q8GXP4]	16660596	16663048	38.85
AT2G39920		Uncharacterized protein At2g39920 [Source:UniProtKB/Swiss-Prot;Acc:O04195]	16663017	16664539	34.73
AT2G39930	ISA1	ISA1 [Source:UniProtKB/TrEMBL;Acc:A0A178VW25]	16665851	16672494	37.42
AT2G09090			16671195	16672443	39.87
AT2G39940	COI1	Coronatine-insensitive protein 1 [Source:UniProtKB/Swiss-Prot;Acc:O04197]	16672493	16675864	40.42
AT2G39950		At2g39950 [Source:UniProtKB/TrEMBL;Acc:O04198]	16676542	16680503	38.69
AT2G39960		Probable signal peptidase complex subunit 2 [Source:UniProtKB/Swiss-Prot;Acc:P58684]	16681296	16683718	36.03
AT2G39970	PXN	Peroxisomal nicotinamide adenine dinucleotide carrier [Source:UniProtKB/Swiss-Prot;Acc:O04200]	16683832	16686815	38.81
AT2G39980		At2g39980/T28M21.14 [Source:UniProtKB/TrEMBL;Acc:O04201]	16687673	16690182	43.59
AT2G39975		CONTAINS InterPro DOMAIN/s: C2 calcium-dependent membrane targeting (InterPro:IPR000008); BEST Arabidopsis thaliana pro	16687717	16688248	37.03
AT2G09095			16693953	16694155	32.02
AT2G09100			16694629	16694948	30.62
AT2G39990	TIF3F1	Eukaryotic translation initiation factor 3 subunit F [Source:UniProtKB/Swiss-Prot;Acc:O04202]	16698041	16700309	38.96
AT2G40000	HSPRO2	Nematode resistance protein-like HSPRO2 [Source:UniProtKB/Swiss-Prot;Acc:O04203]	16700490	16702336	41.2
AT2G40004		unknown protein; LOCATED IN: endomembrane system; Ha. [Source:TAIR;Acc:AT2G40004]	16704063	16704954	28.14
AT2G09105			16704087	16704331	26.53
AT2G40008		other RNA [Source:TAIR;Acc:AT2G40008]	16708056	16709563	40.12
AT2G40010	RPP0A	60S acidic ribosomal protein P0-1 [Source:UniProtKB/Swiss-Prot;Acc:O04204]	16708379	16710772	38.81
AT2G40020		Nucleolar histone methyltransferase-related protein [Source:TAIR;Acc:AT2G40020]	16710929	16713339	37.2
AT2G40030	NRPE1	DNA-directed RNA polymerase V subunit 1 [Source:UniProtKB/Swiss-Prot;Acc:Q5D869]	16714460	16723718	41.29
AT2G40050		Cysteine/Histidine-rich C1 domain family protein [Source:UniProtKB/TrEMBL;Acc:O04208]	16723881	16725803	37.91
AT2G40060	CLC2	Clathrin light chain [Source:UniProtKB/TrEMBL;Acc:A0A178W0Q3]	16726424	16728207	37.16
AT2G40070		At2g40070 [Source:UniProtKB/TrEMBL;Acc:Q494P4]	16728193	16731736	41.34
AT2G40080	ELF4	Protein EARLY FLOWERING 4 [Source:UniProtKB/Swiss-Prot;Acc:O04211]	16734294	16734953	39.7
AT2G09110			16735563	16735801	33.47
AT2G40085		unknown protein; Ha. [Source:TAIR;Acc:AT2G40085]	16736317	16737294	38.45
AT2G40090	ATATH9	ABC transporter like protein [Source:UniProtKB/TrEMBL;Acc:Q67ZT0]	16737466	16740486	38.83
AT2G40095		Alpha/beta hydrolase related protein [Source:UniProtKB/TrEMBL;Acc:Q8GZ10]	16742896	16745152	35.09
AT2G40100	LHCB4.3	Chlorophyll a-b binding protein, chloroplastic [Source:UniProtKB/TrEMBL;Acc:A0A178VTY4]	16745628	16747424	40.29
AT2G40110		Protein yippee-like [Source:UniProtKB/TrEMBL;Acc:Q8LE51]	16747831	16749590	35.11
AT2G40113		Pollen Ole e 1 allergen and extensin family protein [Source:UniProtKB/TrEMBL;Acc:Q58FY6]	16750035	16751314	36.88
AT2G40116	PLC6	Phosphoinositide phospholipase C 6 [Source:UniProtKB/Swiss-Prot;Acc:Q8GV43]	16751561	16754599	37.74
AT2G40120		Hypothetical Ser-Thr protein kinase [Source:UniProtKB/TrEMBL;Acc:Q9XEE4]	16754891	16757599	37.43
AT2G09115			16765369	16765864	37.7
AT2G40130	SMXL8	Protein SMAX1-LIKE 8 [Source:UniProtKB/Swiss-Prot;Acc:F4IGZ2]	16765709	16769279	41.19
AT2G40140	CZF1	Zinc finger CCCH domain-containing protein 29 [Source:UniProtKB/Swiss-Prot;Acc:Q9XEE6]	16771997	16774734	41.49
AT2G40150	TBL28	Protein trichome birefringence-like 28 [Source:UniProtKB/Swiss-Prot;Acc:Q94K00]	16775424	16777243	39.67

MQTLs	Gene stable ID	Gene name	Gene description	Gene start (bp)	Gene end (bp)	Gene % GC content
MQTL-1/Chr3	AT3G01140	MYB106	Myb domain protein 106 [Source:UniProtKB/TrEMBL;Acc:F4J2A8]	46330	48296	40.32
	AT3G01142		other RNA [Source:TAIR;Acc:AT3G01142]	46718	48296	43.07
	AT3G00975			48372	48583	36.32
	AT3G01150	PTB	PTB1 [Source:UniProtKB/TrEMBL;Acc:A0A384LJ51]	51496	54544	40.18
	AT3G01160		Pre-rRNA-processing ESF1-like protein [Source:UniProtKB/TrEMBL;Acc:Q9MAC6]	54524	57541	39.73
	AT3G01170		At3g01170 [Source:UniProtKB/TrEMBL;Acc:Q6NLZ8]	57545	58924	40.51
	AT3G01175		Transmembrane protein [Source:UniProtKB/TrEMBL;Acc:F4J4Q8]	59423	62291	33.77
	AT3G01180	SS2	Starch synthase, chloroplastic/amyloplastic (Fragment) [Source:UniProtKB/TrEMBL;Acc:W8QNS2]	62245	66111	40.96
	AT3G01185		Prolamin-like protein (DUF784) [Source:UniProtKB/TrEMBL;Acc:Q3EBD9]	66151	66980	30.6
	AT3G01190	PER27	Peroxidase [Source:UniProtKB/TrEMBL;Acc:A0A178V802]	67032	68665	37.58
	AT3G01200	RP2	Pyruvate, phosphate dikinase regulatory protein 2 [Source:UniProtKB/Swiss-Prot;Acc:Q9MAC9]	69468	71362	41.79
	AT3G01202		other RNA [Source:TAIR;Acc:AT3G01202]	69854	71415	43.79
	AT3G01210		RNA-binding (RRM/RBD/RNP motifs) family protein [Source:UniProtKB/TrEMBL;Acc:Q9MAD0]	71556	73407	38.66
	AT3G01220	ATHB-20	Homeobox-leucine zipper protein ATHB-20 [Source:UniProtKB/Swiss-Prot;Acc:Q8LAT0]	73307	75539	32.2
	AT3G01230		Splicing regulatory glutamine/lysine-rich-like protein [Source:UniProtKB/TrEMBL;Acc:Q9MAD2]	76631	77411	33.8
	AT3G01240		Splicing regulatory glutamine/lysine-rich-like protein [Source:UniProtKB/TrEMBL;Acc:Q9MAD3]	78095	79037	32.98
	AT3G01250		At3g01250 [Source:UniProtKB/TrEMBL;Acc:Q9MAD4]	79297	80173	32.5
	AT3G01260		Galactose mutarotase-like superfamily protein [Source:UniProtKB/TrEMBL;Acc:F4J5Z7]	80003	82182	33.62
	AT3G01270		Pectate lyase [Source:UniProtKB/TrEMBL;Acc:Q08A70]	82581	84901	40.8
	AT3G01280	VDAC1	Mitochondrial outer membrane protein porin 1 [Source:UniProtKB/Swiss-Prot;Acc:Q9SRH5]	85551	88070	39.17
	AT3G01290	HIR3	HIR2 [Source:UniProtKB/TrEMBL;Acc:A0A178V7M0]	87684	89593	39.01
	AT3G01300		Receptor-like serine/threonine-protein kinase At3g01300 [Source:UniProtKB/Swiss-Prot;Acc:Q9SRH7]	90396	93588	39.18
	AT3G01310		Phosphoglycerate mutase-like family protein [Source:UniProtKB/TrEMBL;Acc:F4J8C7]	93995	102010	38.49
	AT3G01311		Actin cross-linking protein, putative (DUF569) [Source:UniProtKB/TrEMBL;Acc:F4J8C8]	102679	104495	36.43
	AT3G01313		snoRNA [Source:TAIR;Acc:AT3G01313]	104687	104769	32.53
	AT3G01316		snoRNA [Source:TAIR;Acc:AT3G01316]	104835	104909	36
	AT3G00980			104968	105271	35.53
	AT3G00985			104978	105057	32.5
	AT3G01320	SNL1	Paired amphipathic helix protein Sin3-like 1 [Source:UniProtKB/Swiss-Prot;Acc:Q9SRH9]	106294	113679	39.11
	AT3G01319		unknown protein; Ha. [Source:TAIR;Acc:AT3G01319]	106296	106436	41.84
	AT3G01322		ECA1-like gametogenesis related family protein [Source:UniProtKB/TrEMBL;Acc:A8MQI0]	114260	114604	34.78
	AT3G01326		ECA1 gametogenesis related family protein [Source:UniProtKB/TrEMBL;Acc:A8MRE2]	115250	115756	32.15
	AT3G01327		ECA1-like gametogenesis related family protein [Source:UniProtKB/TrEMBL;Acc:A8MRI2]	116226	116568	36.44
	AT3G01328		ECA1-like gametogenesis related family protein [Source:UniProtKB/TrEMBL;Acc:A8MQL3]	117218	117630	33.17
	AT3G01329		ECA1-like gametogenesis related family protein [Source:UniProtKB/TrEMBL;Acc:A7REG8]	118849	119178	40.91
	AT3G01331		ECA1 gametogenesis related family protein [Source:UniProtKB/TrEMBL;Acc:A8MRV4]	119546	120010	37.85
	AT3G01323		ECA1-like gametogenesis related family protein [Source:UniProtKB/TrEMBL;Acc:Q2V3Z5]	121057	121503	36.24
	AT3G01324		ECA1-like gametogenesis related family protein [Source:UniProtKB/TrEMBL;Acc:A7REG9]	122118	122480	38.57
	AT3G01325		At3g01325 [Source:UniProtKB/TrEMBL;Acc:Q3EBD8]	123369	123741	36.19
	AT3G01330	E2FF	E2F transcription factor-like E2FF [Source:UniProtKB/Swiss-Prot;Acc:Q8RWL0]	124319	126876	36.59
	AT3G01340	SEC13A	Protein transport protein SEC13 homolog A [Source:UniProtKB/Swiss-Prot;Acc:Q9SRI1]	127303	128994	42.26
	AT3G01345		Expressed protein [Source:UniProtKB/TrEMBL;Acc:Q3EBD7]	129138	130749	44.67
	AT3G00995			132826	133195	32.97
	AT3G01350	NPF5.9	Protein NRT1/ PTR FAMILY 5.9 [Source:UniProtKB/Swiss-Prot;Acc:Q9SRI2]	134925	137460	39.43
	AT3G01360		At3g01360 [Source:UniProtKB/TrEMBL;Acc:Q9SRI3]	136399	138799	41.07
	AT3G01005			137987	138174	42.02
	AT3G01370	CFM2	CRM-domain containing factor CFM2, chloroplastic [Source:UniProtKB/Swiss-Prot;Acc:Q8L7C2]	139011	143782	41.68
	AT3G01380		Sulfatase and phosphatidylinositolglycan class N domain-containing protein [Source:UniProtKB/TrEMBL;Acc:A0A1I9LQ21]	143880	149880	38.68
	AT3G01390	VHA-G1	V-type proton ATPase subunit G [Source:UniProtKB/TrEMBL;Acc:A0A178VL96]	149940	151572	34.91
	AT3G01400		ARM repeat superfamily protein [Source:UniProtKB/TrEMBL;Acc:Q9SGH8]	151725	153263	45.09
	AT3G01410		Polynucleotidyl transferase, ribonuclease H-like superfamily protein [Source:UniProtKB/TrEMBL;Acc:F4JEB4]	153343	155657	39.09
	AT3G01415		Tubulin-tyrosine ligase [Source:UniProtKB/TrEMBL;Acc:A0A1I9LSK1]	156148	156937	37.97
	AT3G01025			158529	158772	42.62
	AT3G01420	DOX1	Alpha-dioxygenase 1 [Source:UniProtKB/Swiss-Prot;Acc:Q9SGH6]	159450	162905	36.14
	AT3G01430		NHL domain protein [Source:UniProtKB/TrEMBL;Acc:Q9SGH5]	165392	166542	44.48
	AT3G01435		Mediator of RNA polymerase II transcription subunit 11 [Source:UniProtKB/TrEMBL;Acc:A0A178VPC5]	166514	168288	38.25
	AT3G01440	PNSL3	Photosynthetic NDH subunit of lumenal location 3, chloroplastic [Source:UniProtKB/Swiss-Prot;Acc:Q9SGH4]	168408	169662	36.41
	AT3G01450		ARM repeat superfamily protein [Source:UniProtKB/TrEMBL;Acc:Q9SGH3]	169597	172045	39.57
	AT3G01460	MBD9	MBD9 [Source:UniProtKB/TrEMBL;Acc:A0A178VJY9]	173190	182454	41.13
	AT3G01470	HAT5	HD-ZIP-1 [Source:UniProtKB/TrEMBL;Acc:A0A178VBZ2]	182396	184403	40.94
	AT3G01035			182977	183321	33.62
MQTL-2/Chr3	AT3G03340	UNE6	LUC7 related protein [Source:UniProtKB/TrEMBL;Acc:A0A1I9LRM4]	786205	790152	37.72
	AT3G03341		Cold-regulated protein [Source:UniProtKB/TrEMBL;Acc:Q1G3B4]	790130	790693	39.01
	AT3G03350		NAD(P)-binding Rossmann-fold superfamily protein [Source:UniProtKB/TrEMBL;Acc:F4J128]	791183	794812	37.08
	AT3G03360		F-box/RNI-like superfamily protein [Source:TAIR;Acc:AT3G03360]	794906	797013	39.71
	AT3G03370		BEST Arabidopsis thaliana protein match is: DegP protease 7 (TAIR:AT3G03380.1); Ha. [Source:TAIR;Acc:AT3G03370]	797254	799148	37.1
	AT3G03380	DEGP7	Protease Do-like 7 [Source:UniProtKB/Swiss-Prot;Acc:Q8RY22]	799500	808540	37.77
	AT3G03400	CML33	Probable calcium-binding protein CML33 [Source:UniProtKB/Swiss-Prot;Acc:Q9SRP4]	808745	809158	43.96
	AT3G03405		F-box associated ubiquitination effector family protein [Source:UniProtKB/TrEMBL;Acc:F4J135]	809875	810795	38.44
	AT3G03410	CML34	Probable calcium-binding protein CML34 [Source:UniProtKB/Swiss-Prot;Acc:Q9SRP5]	811324	811719	44.95
	AT3G03420		Mitochondrial inner membrane protease ATP23 [Source:UniProtKB/Swiss-Prot;Acc:Q9SRP6]	812396	814101	36.64
	AT3G03430	CML28	Probable calcium-binding protein CML28 [Source:UniProtKB/Swiss-Prot;Acc:Q9SRP7]	814181	814888	33.76
	AT3G03440		ARM repeat superfamily protein [Source:UniProtKB/TrEMBL;Acc:F4J139]	815550	818876	38.23
	AT3G03450	RGL2	RGL2 [Source:UniProtKB/TrEMBL;Acc:A0A178VH73]	819337	821652	44.78
	AT3G03460		Mediator of RNA polymerase II transcription subunit-like protein [Source:UniProtKB/TrEMBL;Acc:Q9SRQ0]	822896	824546	40.76
	AT3G03470	CYP89A9	Cytochrome P450 89A9 [Source:UniProtKB/Swiss-Prot;Acc:Q9SRQ1]	824552	826585	42.77
	AT3G03480	CHAT	(Z)-3-hexen-1-ol acetyltransferase [Source:UniProtKB/Swiss-Prot;Acc:Q9SRQ2]	828235	829926	46.45
	AT3G03490	PEX19-1	Peroxisome biogenesis protein 19-1 [Source:UniProtKB/Swiss-Prot;Acc:Q9SRQ3]	830116	831997	38.42
	AT3G03500		T21P5.8 protein [Source:UniProtKB/TrEMBL;Acc:Q9SRQ4]	832718	834600	36.75
	AT3G01255			835692	836022	34.44
	AT3G03510		Phototropic-responsive NPH3 family protein [Source:TAIR;Acc:AT3G03510]	836039	837707	40.2
	AT3G03520	NPC3	NPC3 [Source:UniProtKB/TrEMBL;Acc:A0A178VBD6]	837805	840645	38.61
	AT3G03530	NPC4	Non-specific phospholipase C4 [Source:UniProtKB/Swiss-Prot;Acc:Q9SRQ7]	842499	845096	36.99
	AT3G03540	NPC5	Non-specific phospholipase C5 [Source:UniProtKB/Swiss-Prot;Acc:Q9S816]	846221	848297	39.53
	AT3G03550	ATL51	Uncharacterized protein At3g03550 [Source:UniProtKB/TrEMBL;Acc:Q0WS90]	850108	851741	40.45
	AT3G03560		unknown protein; LOCATED IN: plasma membrane; EXPRESSED IN: 22 plant structures; EXPRESSED DURING: 13 growth stage	852889	856643	35.31
	AT3G03570		Signal transducer, putative (DUF3550/UPF0682) [Source:UniProtKB/TrEMBL;Acc:F4J153]	856876	860469	39.59
	AT3G03580	PCMP-H23	Pentatricopeptide repeat-containing protein At3g03580 [Source:UniProtKB/Swiss-Prot;Acc:Q9SS60]	860512	863428	41.72
	AT3G01275			864583	864796	39.25
	AT3G01285			864583	864865	39.58
	AT3G03590		At3g03590 [Source:UniProtKB/TrEMBL;Acc:Q9SS61]	865122	867008	37.41
	AT3G03600	RPS2	Putative ribosomal protein S2 [Source:UniProtKB/TrEMBL;Acc:Q0WU50]	867548	868706	43.4
	AT3G03610		ELMO/CED-12 family protein [Source:UniProtKB/TrEMBL;Acc:A0A1I9LNB6]	868741	871837	36.33
	AT3G01295			872996	873346	39.03
	AT3G03620	DTX24	Protein DETOXIFICATION 24 [Source:UniProtKB/Swiss-Prot;Acc:F4J158]	873749	876800	34.67
	AT3G03630	CS26	Probable S-sulfocysteine synthase, chloroplastic [Source:UniProtKB/Swiss-Prot;Acc:O22682]	877844	880461	38.92
	AT3G03640	BGLU25	Probable inactive beta-glucosidase 25 [Source:UniProtKB/Swiss-Prot;Acc:O82772]	880988	884157	37.76
	AT3G03650	EDA5	At3g03650 [Source:UniProtKB/TrEMBL;Acc:Q9SS65]	884133	886410	39.77
	AT3G03660	WOX11	WUSCHEL related homeobox 11 [Source:UniProtKB/TrEMBL;Acc:A0A1I9LND8]	889166	892229	33.58
	AT3G01305			900554	900978	41.41
	AT3G01315			900706	900940	51.06
	AT3G03670	PER28	Peroxidase 28 [Source:UniProtKB/Swiss-Prot;Acc:Q9SS67]	901728	903411	41.45
	AT3G03680		C2 calcium/lipid-binding plant phosphoribosyltransferase family protein [Source:UniProtKB/TrEMBL;Acc:Q9SS68]	907384	910928	47.28
	AT3G03690	UNE7	Core-2/I-branching beta-1,6-N-acetylglucosaminyltransferase family protein [Source:UniProtKB/TrEMBL;Acc:Q9SS69]	911178	913824	36.46
	AT3G03702		other RNA [Source:TAIR;Acc:AT3G03702]	916306	918887	38.3
	AT3G03700		F20H23.27 protein [Source:UniProtKB/TrEMBL;Acc:Q9S7M7]	916435	918490	40.47
	AT3G03710	PNP1	Polyribonucleotide nucleotidyltransferase 1, chloroplastic [Source:UniProtKB/Swiss-Prot;Acc:Q8GZQ3]	919362	925372	38.95
	AT3G03720	CAT4	Cationic amino acid transporter 4, vacuolar [Source:UniProtKB/Swiss-Prot;Acc:Q8W4K3]	925556	930002	37.4

	AT3G03726		F-box/kelch-repeat protein, putative (DUF295) [Source:UniProtKB/TrEMBL;Acc:F4J173]	933341	934464	39.77
	AT3G03730	KIB4	F-box protein KIB4 [Source:UniProtKB/Swiss-Prot;Acc:Q9SRV0]	935139	936320	39.09
	AT3G03740	BPM4	BTB/POZ and MATH domain-containing protein 4 [Source:UniProtKB/Swiss-Prot;Acc:Q9SRV1]	936878	941670	40.66
	AT3G03750	SUVR3	Histone-lysine N-methyltransferase SUVR3 [Source:UniProtKB/Swiss-Prot;Acc:Q9SRV2]	939900	942094	40.91
	AT3G03760	LBD20	LOB domain-containing protein 20 [Source:UniProtKB/Swiss-Prot;Acc:Q9SRV3]	943557	944834	42.96
	AT3G03770		Probable inactive leucine-rich repeat receptor-like protein kinase At3g03770 [Source:UniProtKB/Swiss-Prot;Acc:Q8LFN2]	945077	949238	38.03
	AT3G03773		HSP20-like chaperones superfamily protein [Source:UniProtKB/TrEMBL;Acc:F4J2B8]	951628	954055	36.12
	AT3G03776		Hydroxyproline-rich glycoprotein family protein [Source:UniProtKB/TrEMBL;Acc:A0A1I9LP11]	954021	955371	34.57
	AT3G03780	MS2	5-methyltetrahydropteroyltriglutamate--homocysteine methyltransferase 2 [Source:UniProtKB/Swiss-Prot;Acc:Q9SRV5]	956862	961032	41.52
	AT3G03790		Ankyrin repeat family protein / regulator of chromosome condensation (RCC1) family protein [Source:UniProtKB/TrEMBL;Acc:F4J2	961750	968152	39.7
	AT3G03800	SYP131	Syntaxin of plants 131 [Source:UniProtKB/TrEMBL;Acc:A0A1I9LQP3]	969050	971691	38
	AT3G03810	EDA30	Protein EMBRYO SAC DEVELOPMENT ARREST 30 [Source:UniProtKB/Swiss-Prot;Acc:F4J2C8]	971648	976414	38.26
	AT3G03820		Putative auxin-induced protein [Source:UniProtKB/Swiss-Prot;Acc:Q9SRV9]	976715	977474	36.58
	AT3G03826		Transmembrane protein [Source:UniProtKB/TrEMBL;Acc:B3H4D2]	978265	978570	31.05
	AT3G03828		Transmembrane protein [Source:UniProtKB/TrEMBL;Acc:B3H4H1]	979379	979900	27.39
	AT3G03830	SAUR28	Auxin-responsive protein SAUR28 [Source:UniProtKB/Swiss-Prot;Acc:Q9SRW0]	979976	980562	36.8
	AT3G03840		At3g03840 [Source:UniProtKB/TrEMBL;Acc:Q9SRW1]	981072	981801	35.07
	AT3G03845		pre-tRNA [Source:TAIR;Acc:AT3G03845]	981969	982040	56.94
	AT3G03847		At3g03847 [Source:UniProtKB/TrEMBL;Acc:Q3EBC5]	982192	982587	41.92
	AT3G03850		At3g03850 [Source:UniProtKB/TrEMBL;Acc:Q6NMM4]	983057	983768	35.96
	AT3G03852		pre-tRNA [Source:TAIR;Acc:AT3G03852]	984099	984170	56.94
	AT3G03860	APRL5	5'-adenylylsulfate reductase-like 5 [Source:UniProtKB/Swiss-Prot;Acc:Q93YX4]	992074	994697	37.88
	AT3G03870		F20H23.8 protein [Source:UniProtKB/TrEMBL;Acc:Q9SRW4]	995062	997410	34.31
	AT3G03880		Sterol O-acyltransferase, putative (DUF1639) [Source:UniProtKB/TrEMBL;Acc:Q84JV8]	997724	999359	37.96
	AT3G03890		AT3G03890 protein [Source:UniProtKB/TrEMBL;Acc:Q8LDU1]	999350	1002595	38.63
	AT3G03900	APK3	Adenylyl-sulfate kinase 3 [Source:UniProtKB/Swiss-Prot;Acc:Q9SRW7]	1002765	1005025	36.97
	AT3G01365			1004758	1005154	36.52
	AT3G03910	GSH3	Glutamate dehydrogenase [Source:UniProtKB/TrEMBL;Acc:A0A178VBI2]	1006780	1009018	40.24
	AT3G03920		Putative H/ACA ribonucleoprotein complex subunit 1-like protein 1 [Source:UniProtKB/Swiss-Prot;Acc:Q8VZT0]	1008995	1010573	41.48
	AT3G01375			1009803	1010048	34.96
	AT3G03930		F20H23.2 protein [Source:UniProtKB/TrEMBL;Acc:Q9S828]	1011310	1012810	37.31
	AT3G03940		Protein kinase family protein [Source:UniProtKB/TrEMBL;Acc:Q9SQR8]	1013929	1019418	37.69
	AT3G03950	ECT1	YTH domain-containing protein ECT1 [Source:UniProtKB/Swiss-Prot;Acc:Q3MK94]	1021146	1024015	38.99
	AT3G03960	CCT8	T-complex protein 1 subunit theta [Source:UniProtKB/Swiss-Prot;Acc:Q94K05]	1024144	1027891	38.45
	AT3G03970	SINE2	Protein SINE2 [Source:UniProtKB/Swiss-Prot;Acc:Q9SQR5]	1027866	1030677	38.62
	AT3G03980		NADPH-dependent aldehyde reductase-like protein, chloroplastic [Source:UniProtKB/Swiss-Prot;Acc:Q9SQR4]	1031660	1033237	41.44
	AT3G03990	D14	Strigolactone esterase D14 [Source:UniProtKB/Swiss-Prot;Acc:Q9SQR3]	1033579	1035237	38.94
	AT3G04000	ChlADR2	NADPH-dependent aldehyde reductase 2, chloroplastic [Source:UniProtKB/Swiss-Prot;Acc:Q9SQR2]	1035238	1036672	40.63
	AT3G04010		At3g04010 [Source:UniProtKB/TrEMBL;Acc:Q9SQR1]	1036194	1039603	36.72
	AT3G04020		unknown protein; Ha. [Source:TAIR;Acc:AT3G04020]	1040331	1042547	36.18
	AT3G04030	MYR2	Myb-related protein 2 [Source:UniProtKB/Swiss-Prot;Acc:Q9SQQ9]	1042700	1044813	37.8
	AT3G01385			1045178	1045658	33.89
	AT3G04040		AT3G04040 protein [Source:UniProtKB/TrEMBL;Acc:Q6NNK6]	1047640	1049444	36.34
	AT3G04050		Pyruvate kinase [Source:UniProtKB/TrEMBL;Acc:Q9SQQ7]	1049653	1051608	43.51
	AT3G04060	anac046	NAC domain-containing protein 46 [Source:UniProtKB/Swiss-Prot;Acc:Q9SQQ6]	1053360	1055196	38.76
	AT3G04070	NAC047	NAC transcription factor 47 [Source:UniProtKB/Swiss-Prot;Acc:Q84TD6]	1061300	1063234	34.88
	AT3G04080	APY1	Apyrase 1 [Source:UniProtKB/Swiss-Prot;Acc:Q9SQG2]	1067558	1071438	38.96
	AT3G04090	SIP1-1	SIP1A [Source:UniProtKB/TrEMBL;Acc:A0A178V5Y6]	1071984	1074244	38.83
	AT3G04100	AGL57	AGAMOUS-like 57 [Source:UniProtKB/TrEMBL;Acc:Q9M8W6]	1075299	1075922	44.55
	AT3G01395			1076492	1076805	39.49
	AT3G04110	GLR1.1	Glutamate receptor 1.1 [Source:UniProtKB/Swiss-Prot;Acc:Q9M8W7]	1077236	1080504	38.76
	AT3G04120	GAPC1	Glyceraldehyde-3-phosphate dehydrogenase GAPC1, cytosolic [Source:UniProtKB/Swiss-Prot;Acc:P25858]	1080942	1083690	39.07
MQTL-3/Chr3	AT3G14720	MPK19	Mitogen-activated protein kinase 19 [Source:UniProtKB/Swiss-Prot;Acc:Q9LUC3]	4945601	4949042	39.13
	AT3G14730	PCMP-E31	Pentatricopeptide repeat-containing protein At3g14730 [Source:UniProtKB/Swiss-Prot;Acc:Q9LUC2]	4949178	4951346	42.88
	AT3G14735	U6-1	U6-1; snRNA [Source:TAIR;Acc:AT3G14735]	4951597	4951697	44.55
	AT3G14740		RING/FYVE/PHD zinc finger superfamily protein [Source:UniProtKB/TrEMBL;Acc:Q9LUC1]	4952004	4953510	39.15
	AT3G14750	FLXL1	Protein FLX-like 1 [Source:UniProtKB/Swiss-Prot;Acc:Q93V84]	4953547	4955754	39.72
	AT3G14760		Transmembrane protein [Source:UniProtKB/TrEMBL;Acc:Q9LH80]	4956349	4957306	41.02
	AT3G14770	SWEET2	Bidirectional sugar transporter SWEET [Source:UniProtKB/TrEMBL;Acc:A0A178VM79]	4957334	4959704	34.42
	AT3G02995			4959718	4959962	31.02
	AT3G14780		Callose synthase [Source:UniProtKB/TrEMBL;Acc:Q8GW77]	4961777	4963569	35.86
	AT3G14790	RHM3	Rhamnose biosynthesis 3 [Source:UniProtKB/TrEMBL;Acc:A0A1I9LN83]	4964105	4967075	42.04
	AT3G14810	MSL5	Mechanosensitive ion channel protein 5 [Source:UniProtKB/Swiss-Prot;Acc:Q9LH74]	4970944	4974534	40.71
	AT3G03005			4976881	4977193	38.02
	AT3G14820		GDSL esterase/lipase At3g14820 [Source:UniProtKB/Swiss-Prot;Acc:Q9LH73]	4978726	4980241	32.39
	AT3G14830		AT3G14830 protein [Source:UniProtKB/TrEMBL;Acc:Q8L634]	4982588	4985860	39.66
	AT3G14840	LRR-RLK	Probable leucine-rich repeat receptor-like serine/threonine-protein kinase At3g14840 [Source:UniProtKB/Swiss-Prot;Acc:C0LGN2]	4988008	4994109	37.28
	AT3G14850	TBL41	Protein trichome birefringence-like 41 [Source:UniProtKB/Swiss-Prot;Acc:F4IWA8]	4995426	4997785	36.82
	AT3G14855		pre-tRNA [Source:TAIR;Acc:AT3G14855]	4998141	4998212	56.94
	AT3G14860		NHL domain-containing protein [Source:UniProtKB/TrEMBL;Acc:F4IXC9]	4998304	5001046	39.01
	AT3G14870		AT3G14870 protein [Source:UniProtKB/TrEMBL;Acc:Q9LKB7]	5002271	5005877	37.18
	AT3G14880		DNA-binding protein-like protein [Source:UniProtKB/TrEMBL;Acc:Q9LKB6]	5006526	5008037	40.87
	AT3G14890	ZDP	Polynucleotide 3'-phosphatase ZDP [Source:UniProtKB/Swiss-Prot;Acc:Q84JE8]	5008676	5013265	37.65
	AT3G14900		Uncharacterized protein At3g14900 [Source:UniProtKB/TrEMBL;Acc:Q9LKB4]	5013291	5015342	43.37
	AT3G14910		At3g14910 [Source:UniProtKB/TrEMBL;Acc:Q9LKB3]	5015411	5017861	38.43
	AT3G14920		Peptide-N4-(N-acetyl-beta-glucosaminyl)asparagine amidase A protein [Source:UniProtKB/TrEMBL;Acc:Q9LKB2]	5017941	5020476	40.46
	AT3G14930	HEME1	Uroporphyrinogen decarboxylase 1, chloroplastic [Source:UniProtKB/Swiss-Prot;Acc:Q93ZB6]	5020461	5023146	40.73
	AT3G14940	PPC3	Phosphoenolpyruvate carboxylase 3 [Source:UniProtKB/Swiss-Prot;Acc:Q84VW9]	5025184	5029886	41.82
	AT3G14950	TTL2	TPR repeat-containing thioredoxin TTL2 [Source:UniProtKB/Swiss-Prot;Acc:F4IXE4]	5029987	5033102	38.51
	AT3G14960	B3GALT13	Hexosyltransferase (Fragment) [Source:UniProtKB/TrEMBL;Acc:W8Q363]	5035841	5038114	41.25
	AT3G14970		RING/U-box superfamily protein [Source:UniProtKB/TrEMBL;Acc:Q9LKA8]	5039071	5039733	42.99
	AT3G14980	IDM1	Increased DNA methylation 1 [Source:UniProtKB/Swiss-Prot;Acc:F4IXE7]	5039812	5045525	39.87
	AT3G14981		unknown protein; Ha. [Source:TAIR;Acc:AT3G14981]	5045110	5045328	42.47
	AT3G14990	DJ1A	Protein DJ-1 homolog A [Source:UniProtKB/Swiss-Prot;Acc:Q9FPF0]	5047376	5049971	39.83
	AT3G15000	MORF8	Multiple organellar RNA editing factor 8, chloroplastic/mitochondrial [Source:UniProtKB/Swiss-Prot;Acc:Q9LKA5]	5050195	5052455	46.17
	AT3G15010	UBA2C	UBP1-associated protein 2C [Source:UniProtKB/Swiss-Prot;Acc:Q9LKA4]	5052725	5056017	41.33
	AT3G15020	mMDH2	Malate dehydrogenase 2, mitochondrial [Source:UniProtKB/Swiss-Prot;Acc:Q9LKA3]	5055741	5058249	40.06
	AT3G15030	TCP4	TCP4 [Source:UniProtKB/TrEMBL;Acc:A0A384KY84]	5061681	5064152	46.08
	AT3G15040		At3g15040 [Source:UniProtKB/TrEMBL;Acc:Q9LKA1]	5064704	5066745	38.98
	AT3G15050	IQD10	IQ-domain 10 [Source:UniProtKB/TrEMBL;Acc:Q9LKA0]	5066879	5068013	40.62
	AT3G15055		pre-tRNA [Source:TAIR;Acc:AT3G15055]	5068294	5068367	63.51
	AT3G15060	RABA1G	Ras-related protein RABA1g [Source:UniProtKB/Swiss-Prot;Acc:Q9LK99]	5069104	5070540	39.74
	AT3G15070		AT3g15070/K15M2_22 [Source:UniProtKB/TrEMBL;Acc:Q94F54]	5070134	5073440	38.22
	AT3G15080		At3g15080 [Source:UniProtKB/TrEMBL;Acc:Q8LAA0]	5074216	5076566	37.81
	AT3G15090		AT3g15090/K15M2_24 [Source:UniProtKB/TrEMBL;Acc:Q9LK96]	5076624	5079159	39.39
	AT3G15095		HCF243 [Source:UniProtKB/TrEMBL;Acc:A0A384K8R0]	5081780	5084240	46.24
	AT3G15110		At3g15110 [Source:UniProtKB/TrEMBL;Acc:Q2HIU0]	5084278	5085942	40.6
	AT3G15111		unknown protein; LOCATED IN: endomembrane system; Ha. [Source:TAIR;Acc:AT3G15111]	5085860	5085976	41.03
	AT3G15115		Serine/arginine repetitive matrix protein [Source:UniProtKB/TrEMBL;Acc:Q9LIM3]	5085992	5087496	38.47
	AT3G15120		P-loop containing nucleoside triphosphate hydrolases superfamily protein [Source:UniProtKB/TrEMBL;Acc:Q9LIM2]	5088093	5095965	41.09
	AT3G15130	PCMP-H86	Putative pentatricopeptide repeat-containing protein At3g15130 [Source:UniProtKB/Swiss-Prot;Acc:P0C898]	5096402	5099371	42.02
	AT3G15140		Uncharacterized exonuclease domain-containing protein At3g15140 [Source:UniProtKB/Swiss-Prot;Acc:Q8W566]	5099426	5101880	38.04
	AT3G15150	MMS21	E3 SUMO-protein ligase MMS21 [Source:UniProtKB/Swiss-Prot;Acc:Q8GYH7]	5101844	5104159	37.87
	AT3G15160		AP-5 complex subunit zeta-1 [Source:UniProtKB/TrEMBL;Acc:Q8H1F1]	5104367	5108509	38.18
	AT3G15170	NAC054	CUC1 [Source:UniProtKB/TrEMBL;Acc:A0A178V703]	5109782	5111608	41.22
	AT3G15180		ARM repeat superfamily protein [Source:UniProtKB/TrEMBL;Acc:F4IXH9]	5111873	5116068	38.13
	AT3G15190	RPS20	PRPS20 [Source:UniProtKB/TrEMBL;Acc:A0A178VMN1]	5116021	5117615	41.13
	AT3G03025			5116694	5117175	35.68
	AT3G15200		Putative pentatricopeptide repeat-containing protein At3g15200 [Source:UniProtKB/Swiss-Prot;Acc:Q9LIL5]	5117489	5119060	40.9

AT3G15210	ERF4	Ethylene-responsive transcription factor 4 [Source:UniProtKB/Swiss-Prot;Acc:O80340]	5121303	5122562	46.59
AT3G03035			5125409	5126398	37.58
AT3G03045			5125830	5126100	43.17
AT3G15220		MAP kinase [Source:UniProtKB/TrEMBL;Acc:Q9LDN6]	5126590	5132330	37.01
AT3G03055			5132447	5132969	43.59
AT3G03065			5132771	5132969	47.74
AT3G15240		Serine/threonine-protein kinase WNK (With No Lysine)-like protein [Source:UniProtKB/TrEMBL;Acc:F4IXI5]	5133444	5136032	37.62
AT3G15250		TPRXL [Source:UniProtKB/TrEMBL;Acc:Q5BPR0]	5136262	5137195	44.11
AT3G15251		unknown protein; Ha. [Source:TAIR;Acc:AT3G15251]	5137205	5137318	48.25
AT3G15260		Probable protein phosphatase 2C 39 [Source:UniProtKB/Swiss-Prot;Acc:Q9LDA7]	5138684	5142873	38.62
AT3G15270	SPL5	SPL5 [Source:UniProtKB/TrEMBL;Acc:A0A178VL13]	5140365	5142323	39
AT3G03075			5142717	5143104	35.82
AT3G15280		AT3g15280/K7L4_8 [Source:UniProtKB/TrEMBL;Acc:Q9LDN8]	5143965	5144750	44.02
AT3G15290		3-hydroxyacyl-CoA dehydrogenase family protein [Source:UniProtKB/TrEMBL;Acc:Q9LDF5]	5144938	5146860	39.63
AT3G15300	VQ19	VQ motif-containing protein 19 [Source:UniProtKB/Swiss-Prot;Acc:Q9LDZ1]	5147244	5148340	39.47
AT3G03085			5156175	5156420	41.46
AT3G15340	PPI2	PPI2 [Source:UniProtKB/TrEMBL;Acc:A0A178VC28]	5159698	5162945	37.28
AT3G15350		AT3g15350/K7L4_15 [Source:UniProtKB/TrEMBL;Acc:Q9LE60]	5166175	5169229	38.1
AT3G15354	SPA3	Protein SPA1-RELATED 3 [Source:UniProtKB/Swiss-Prot;Acc:Q9LJR3]	5169095	5173105	39.42
AT3G15359		unknown protein; Ha. [Source:TAIR;Acc:AT3G15359]	5173496	5173805	41.61
AT3G15356	LEC	Lectin-like protein LEC [Source:UniProtKB/Swiss-Prot;Acc:Q9LJR2]	5174402	5175523	41
AT3G15351		At3g15351 [Source:UniProtKB/TrEMBL;Acc:Q9LJR1]	5175948	5177721	36.36
AT3G15358		Transmembrane protein [Source:UniProtKB/TrEMBL;Acc:Q9LJR0]	5177811	5178966	35.81
AT3G15352	ATCOX17	Cytochrome c oxidase 17 [Source:UniProtKB/TrEMBL;Acc:A0A1I9LSX1]	5179358	5180665	35.86
AT3G03095			5179533	5180286	32.76
AT3G15353	MT3	Metallothionein-like protein 3 [Source:UniProtKB/Swiss-Prot;Acc:O22433]	5180642	5181586	38.84
AT3G03105			5181021	5181298	41.37
AT3G15355	UBC25	Probable ubiquitin-conjugating enzyme E2 25 [Source:UniProtKB/Swiss-Prot;Acc:Q9LUQ5]	5183641	5186929	38.64
AT3G15357		At3g15357 [Source:UniProtKB/TrEMBL;Acc:Q8LDR8]	5187082	5187789	42.51
AT3G15360	ATHM4	TRX-M4 [Source:UniProtKB/TrEMBL;Acc:A0A178VJ83]	5188241	5189836	40.79
AT3G15370	ATEXPA12	Expansin [Source:UniProtKB/TrEMBL;Acc:A0A1I9LSN2]	5190554	5192197	37.83
AT3G15380	CHER1	Choline transporter protein 1 [Source:UniProtKB/Swiss-Prot;Acc:Q94AN2]	5193203	5196591	39.36
AT3G15390	SDE5	Putative nuclear RNA export factor SDE5 [Source:UniProtKB/Swiss-Prot;Acc:Q9LUQ3]	5196522	5200394	37.36
AT3G15395		AT3G15395 protein [Source:UniProtKB/TrEMBL;Acc:Q8L9Y4]	5200639	5201514	35.62
AT3G15400	ATA20	ATA20 protein [Source:UniProtKB/TrEMBL;Acc:Q9LD84]	5201622	5203197	46.89
AT3G15410		Leucine-rich repeat (LRR) family protein [Source:UniProtKB/TrEMBL;Acc:F4IYR7]	5203205	5207574	36.68
AT3G15420		At3g15420 [Source:UniProtKB/TrEMBL;Acc:Q9LDR3]	5207575	5208509	33.9
AT3G15430		MJK13.9 protein [Source:UniProtKB/TrEMBL;Acc:Q9LDU3]	5208559	5211779	40.92
AT3G15440		MJK13.10 protein [Source:UniProtKB/TrEMBL;Acc:Q9LE10]	5211795	5212506	34.27
AT3G15450		AT3G15450/MJK13_11 [Source:UniProtKB/TrEMBL;Acc:Q9LE80]	5212984	5214124	38.91
AT3G15460	BRIX1-1	Ribosome biogenesis protein BRX1 homolog 1 [Source:UniProtKB/Swiss-Prot;Acc:Q9LE16]	5214084	5216261	37.37
AT3G15470		AT3g15470/MJK13_13 [Source:UniProtKB/TrEMBL;Acc:Q9LDG7]	5216262	5220274	41.61
AT3G15480		AT3g15480/MJK13_14 [Source:UniProtKB/TrEMBL;Acc:Q9LDK1]	5226161	5227935	36.45
AT3G15490		Regulator of Vps4 activity in the MVB pathway protein [Source:UniProtKB/TrEMBL;Acc:Q4PSP2]	5229124	5230646	35.13
AT3G15500	NAC055	NAC3 [Source:UniProtKB/TrEMBL;Acc:A0A178VLD3]	5234457	5236196	39.08
AT3G03115			5239222	5239643	29.62
AT3G15510	NAC056	NAC transcription factor 56 [Source:UniProtKB/Swiss-Prot;Acc:Q9LD44]	5243432	5245510	38.72
AT3G03125			5248893	5249487	45.04
AT3G15518		Putative uncharacterized protein [Source:UniProtKB/TrEMBL;Acc:Q8GYA5]	5249112	5249754	45.1
AT3G15520	CYP37	Peptidyl-prolyl cis-trans isomerase CYP37, chloroplastic [Source:UniProtKB/Swiss-Prot;Acc:P82869]	5249599	5252792	39.29
AT3G03135			5251157	5251646	35.71
AT3G15530		Expressed protein [Source:UniProtKB/TrEMBL;Acc:Q9LDW4]	5252699	5254562	44.1
AT3G15534		unknown protein; FUNCTIONS IN: molecular_function unknown; INVOLVED IN: biological_process unknown; LOCATED IN: cel	5258715	5259354	37.03
AT3G15536		Unknown gene [Source:TAIR;Acc:AT3G15536]	5260723	5261322	32.67
AT3G15540	IAA19	Auxin-responsive protein [Source:UniProtKB/TrEMBL;Acc:Q2VWA2]	5264001	5265695	35.69
AT3G15548		Transmembrane protein [Source:UniProtKB/TrEMBL;Acc:B3H4Q8]	5267063	5267164	48.04
AT3G15550		Trichohyalin [Source:UniProtKB/TrEMBL;Acc:Q9LRP9]	5267204	5270118	39.62
AT3G15570		Non-phototropic hypocotyl-like protein [Source:UniProtKB/TrEMBL;Acc:Q9LRP8]	5269849	5271984	42.37
AT3G03145			5270258	5270755	46.59
AT3G15580	ATG8I	Autophagy-related protein [Source:UniProtKB/TrEMBL;Acc:A0A178VBL7]	5273526	5275095	36.69
AT3G15578		unknown protein; Ha. [Source:TAIR;Acc:AT3G15578]	5273691	5273873	41.53
AT3G15585		pre-tRNA [Source:TAIR;Acc:AT3G15585]	5275149	5275221	58.9
AT3G15590		Pentatricopeptide repeat-containing protein At3g15590, mitochondrial [Source:UniProtKB/Swiss-Prot;Acc:Q9LRP6]	5275387	5278019	39.27
AT3G15605		Nucleic acid binding protein [Source:UniProtKB/TrEMBL;Acc:F4J000]	5287323	5290971	38.34
AT3G15604		unknown protein; Ha. [Source:TAIR;Acc:AT3G15604]	5287380	5287544	47.27
AT3G15610		Expressed protein [Source:UniProtKB/TrEMBL;Acc:Q9LW17]	5290847	5293241	37.58
AT3G03155			5292030	5292702	36.85
AT3G15620	UVR3	(6-4)DNA photolyase [Source:UniProtKB/Swiss-Prot;Acc:O48652]	5293256	5296811	37.96
AT3G15630		AT3g15630/MSJ11_3 [Source:UniProtKB/TrEMBL;Acc:Q9LW16]	5296841	5297851	40.36
AT3G15635			5297396	5297786	40.92
AT3G15640	COX5B-1	Cytochrome c oxidase subunit 5b-1, mitochondrial [Source:UniProtKB/Swiss-Prot;Acc:Q9LW15]	5298991	5301745	37.5
AT3G15650		Alpha/beta-Hydrolases superfamily protein [Source:UniProtKB/TrEMBL;Acc:F4J010]	5305549	5307979	36.57
AT3G15660	GRXS15	GRX4 [Source:UniProtKB/TrEMBL;Acc:A0A178VHP8]	5307694	5309624	37.34
AT3G15670	LEA29	Late embryogenesis abundant protein 29 [Source:UniProtKB/Swiss-Prot;Acc:Q9LW12]	5309886	5310988	43.88
AT3G15680		At3g15680 [Source:UniProtKB/TrEMBL;Acc:Q9LW11]	5314817	5316617	35.7
AT3G15690		Putative acetyl-CoA carboxylase biotin-containing subunit [Source:UniProtKB/TrEMBL;Acc:Q8GRT9]	5316916	5320121	36.68
AT3G15700		P-loop containing nucleoside triphosphate hydrolases superfamily protein [Source:TAIR;Acc:AT3G15700]	5320644	5322432	39.97
AT3G15710		Signal peptidase I [Source:UniProtKB/TrEMBL;Acc:Q9LW08]	5323262	5324966	37.65
AT3G15720		Probable polygalacturonase At3g15720 [Source:UniProtKB/Swiss-Prot;Acc:Q9LW07]	5325127	5327672	36.61
AT3G15730	PLDALPHA1	Phospholipase D alpha 1 [Source:UniProtKB/Swiss-Prot;Acc:Q38882]	5330194	5333745	42.51
AT3G15740		RING/U-box superfamily protein [Source:UniProtKB/TrEMBL;Acc:Q9LW05]	5333769	5334553	43.18
AT3G15750		Essential protein Yae1, N-terminal [Source:UniProtKB/TrEMBL;Acc:Q9LW04]	5334844	5336485	36.97
AT3G15760		At3g15760 [Source:UniProtKB/TrEMBL;Acc:Q9LW03]	5337475	5338562	37.04
AT3G15770		AT3G15770 protein [Source:UniProtKB/TrEMBL;Acc:B9DGF6]	5339992	5341339	37.02
AT3G15780		Transmembrane protein [Source:UniProtKB/TrEMBL;Acc:Q9LW01]	5341356	5342557	39.35
AT3G15790	MBD11	Methyl-CpG-binding domain-containing protein 11 [Source:UniProtKB/Swiss-Prot;Acc:Q9LW00]	5342712	5344715	38.42
AT3G15800		Glycosyl hydrolase superfamily protein [Source:UniProtKB/TrEMBL;Acc:F4J030]	5344989	5347147	39.37
AT3G15810		Protein LURP-one-related 12 [Source:UniProtKB/Swiss-Prot;Acc:Q9LVZ8]	5347762	5349321	37.69
AT3G15820	ROD1	ROD1 [Source:UniProtKB/TrEMBL;Acc:A0A178VDV2]	5350814	5353752	36.47
AT3G15830		Phosphatidylcholine:diacylglycerol cholinephosphotransferase 2 [Source:UniProtKB/Swiss-Prot;Acc:Q9LVZ6]	5354874	5356426	40.18
AT3G15840	PIFI	Post-illumination chlorophyll fluorescence increase [Source:UniProtKB/TrEMBL;Acc:Q9LVZ5]	5356587	5358596	34.98
AT3G15850	ADS3	JB67 [Source:UniProtKB/TrEMBL;Acc:A0A178VBZ6]	5358601	5361185	37.83
AT3G15860		Plant self-incompatibility protein S1 family protein [Source:UniProtKB/TrEMBL;Acc:Q5XVB9]	5361207	5361719	45.61
AT3G15870	ADS3.2	Probable lipid desaturase ADS3.2, chloroplastic [Source:UniProtKB/Swiss-Prot;Acc:Q9LVZ3]	5362888	5364544	40.25
AT3G15880	WSIP2	WUS-interacting protein 2 [Source:TAIR;Acc:AT3G15880]	5364081	5372293	38.13
AT3G15890		PTI1-like tyrosine-protein kinase At3g15890 [Source:UniProtKB/Swiss-Prot;Acc:Q9LSC2]	5374248	5376275	37.97
AT3G15900		At3g15900 [Source:UniProtKB/TrEMBL;Acc:Q9LSC1]	5376251	5377179	39.83
AT3G03165			5380310	5380726	45.08
AT3G15909		unknown protein; LOCATED IN: mitochondrion; Ha. [Source:TAIR;Acc:AT3G15909]	5381603	5381740	39.86
AT3G15910		unknown protein; Ha. [Source:TAIR;Acc:AT3G15910]	5382061	5383069	42.91
AT3G15920	EREX	PX domain-containing protein EREX [Source:UniProtKB/Swiss-Prot;Acc:Q9LSB9]	5383595	5390219	40.65
AT3G03175			5387184	5387268	50.59
AT3G15930	PCMP-E51	Putative pentatricopeptide repeat-containing protein At3g15930 [Source:UniProtKB/Swiss-Prot;Acc:Q9LSB8]	5387444	5389690	41.34
AT3G15940		AT3g15940/MVC8_7 [Source:UniProtKB/TrEMBL;Acc:Q9LSB5]	5393451	5396994	41.51
AT3G03195			5396819	5397040	43.24
AT3G15950	NAI2	TSA1-like protein [Source:UniProtKB/Swiss-Prot;Acc:Q9LSB4]	5397541	5402653	36.06
AT3G15960		NAI2-like protein [Source:UniProtKB/Swiss-Prot;Acc:F4J1D9]	5403970	5406461	36.96
AT3G15970	NUP50B	Nuclear pore complex protein NUP50B [Source:UniProtKB/Swiss-Prot;Acc:Q9LW88]	5408792	5411153	42
AT3G15980		Coatomer subunit beta' [Source:UniProtKB/TrEMBL;Acc:F4J1E5]	5411278	5418632	37.46

	AT3G03205			5422229	5422464	36.86
	AT3G15990	SULTR3;4	Probable sulfate transporter 3.4 [Source:UniProtKB/Swiss-Prot;Acc:Q9LW86]	5426987	5430897	36.51
	AT3G16000	MFP1	MAR-binding filament-like protein 1 [Source:UniProtKB/Swiss-Prot;Acc:Q9LW85]	5430758	5433851	40.01
	AT3G16010		Pentatricopeptide repeat-containing protein At3g16010 [Source:UniProtKB/Swiss-Prot;Acc:Q9LW84]	5433526	5436828	39.18
	AT3G16020		F-box associated ubiquitination effector protein [Source:UniProtKB/TrEMBL;Acc:F4J1E9]	5437962	5439461	38.2
	AT3G16030	CES101	Lectin protein kinase family protein [Source:UniProtKB/TrEMBL;Acc:A0A1I9LPL5]	5439311	5442997	39.76
	AT3G16040		At3g16040 [Source:UniProtKB/TrEMBL;Acc:Q1EBS5]	5443081	5443952	35.55
	AT3G16050	PDX12	Pyridoxal 5'-phosphate synthase-like subunit PDX1.2 [Source:UniProtKB/Swiss-Prot;Acc:Q9ZNR6]	5443950	5445329	44.71
	AT3G03215			5444368	5444626	45.17
	AT3G16060	KIN13B	Kinesin-like protein [Source:UniProtKB/TrEMBL;Acc:A0A178V575]	5447272	5451562	39.69
	AT3G16070		LOW protein: ATP-dependent RNA helicase-like protein [Source:UniProtKB/TrEMBL;Acc:Q9LW80]	5451533	5452363	38.75
	AT3G16080	RPL37C	60S ribosomal protein L37-3 [Source:UniProtKB/Swiss-Prot;Acc:Q8LEM8]	5454762	5455942	38.27
	AT3G16090	HRD1A	ERAD-associated E3 ubiquitin-protein ligase HRD1A [Source:UniProtKB/Swiss-Prot;Acc:Q9LW77]	5456093	5459006	38.16
	AT3G16100	RABG3C	Ras-related protein RABG3c [Source:UniProtKB/Swiss-Prot;Acc:Q9LW76]	5459162	5460816	36.19
	AT3G16110	PDIL1-6	PDIL1-6 [Source:UniProtKB/TrEMBL;Acc:A0A178VNL8]	5460793	5463899	37.85
	AT3G16117		unknown protein; Ha. [Source:TAIR;Acc:AT3G16117]	5464543	5464710	41.67
	AT3G16120		Dynein light chain [Source:UniProtKB/TrEMBL;Acc:Q9LW74]	5464728	5465585	34.38
	AT3G16130	ATROPGEF13	RHO guanyl-nucleotide exchange factor 13 [Source:TAIR;Acc:AT3G16130]	5466246	5468774	38.04
	AT3G16140	PSAH1	Photosystem I reaction center subunit VI-1, chloroplastic [Source:UniProtKB/Swiss-Prot;Acc:Q9SUI7]	5467987	5469594	39.05
	AT3G16150		ASPGB1 [Source:UniProtKB/TrEMBL;Acc:A0A384KV24]	5471735	5473360	43.36
	AT3G16160	TCX8	Protein tesmin/TSO1-like CXC 8 [Source:UniProtKB/Swiss-Prot;Acc:Q700D0]	5473366	5475347	40.16
	AT3G16170	AAE13	AMP-dependent synthetase and ligase family protein [Source:TAIR;Acc:AT3G16170]	5475855	5480349	37.46
	AT3G16175		At3g16179/At3g16179 [Source:UniProtKB/TrEMBL;Acc:Q940V5]	5480416	5481379	36.31
	AT3G16180	NPF1.1	Protein NRT1/ PTR FAMILY 1.1 [Source:UniProtKB/Swiss-Prot;Acc:Q8LPL2]	5481325	5485187	37.04
	AT3G03225			5483017	5484530	31.18
	AT3G16190		Probable inactive nicotinamidase At3g16190 [Source:UniProtKB/Swiss-Prot;Acc:Q93Z51]	5489701	5491332	37.75
	AT3G16200		DNA-directed RNA polymerase subunit beta [Source:UniProtKB/TrEMBL;Acc:Q9LU25]	5491398	5493681	41.68
	AT3G16210		Putative F-box protein At3g16210 [Source:UniProtKB/Swiss-Prot;Acc:Q9LU24]	5494480	5495725	41.41
	AT3G03235			5496985	5497119	34.81
	AT3G16220		At3g16220 [Source:UniProtKB/TrEMBL;Acc:Q6NKQ6]	5497481	5499474	35.71
	AT3G03245			5500032	5500152	31.4
	AT3G03255			5500037	5500147	33.33
	AT3G16230		Putative eukaryotic LigT [Source:UniProtKB/TrEMBL;Acc:F4J1H2]	5500054	5503573	36.53
	AT3G16240	TIP2-1	Aquaporin TIP2-1 [Source:UniProtKB/Swiss-Prot;Acc:Q41951]	5505360	5507050	39.38
	AT3G03265			5505992	5506436	31.01
	AT3G16250	PNSB3	Photosynthetic NDH subunit of subcomplex B 3, chloroplastic [Source:UniProtKB/Swiss-Prot;Acc:Q9LU21]	5506931	5508414	34.64
	AT3G03275			5507834	5508115	31.21
	AT3G16260	TRZ4	TRZ4 [Source:UniProtKB/TrEMBL;Acc:A0A178V7J3]	5509344	5513245	40.39
	AT3G16270	MTV1	ENTH/VHS family protein [Source:UniProtKB/TrEMBL;Acc:A0A178VIA7]	5513267	5517606	39.15
	AT3G16280		Integrase-type DNA-binding superfamily protein [Source:UniProtKB/TrEMBL;Acc:A0A1I9LT94]	5518211	5519271	41.85
	AT3G16290	FTSHI2	Probable inactive ATP-dependent zinc metalloprotease FTSHI 2, chloroplastic [Source:UniProtKB/Swiss-Prot;Acc:A8MPR5]	5520880	5525150	39.29
	AT3G16300		CASP-like protein 3A1 [Source:UniProtKB/Swiss-Prot;Acc:Q3EB59]	5525322	5526212	43.77
	AT3G16310	NUP35	Nuclear pore complex protein NUP35 [Source:UniProtKB/Swiss-Prot;Acc:O04326]	5526249	5528602	40.99
	AT3G16320	CDC27a	Tetratricopeptide repeat (TPR)-like superfamily protein [Source:TAIR;Acc:AT3G16320]	5529313	5534734	37.62
	AT3G16330		At3g16330 [Source:UniProtKB/TrEMBL;Acc:O04324]	5535124	5536237	41.56
	AT3G16340	ABCG29	ABC transporter G family member 29 [Source:UniProtKB/Swiss-Prot;Acc:Q94A18]	5539385	5546487	37.52
	AT3G16350		At3g16350 [Source:UniProtKB/TrEMBL;Acc:Q84TG2]	5547492	5549862	40.28
	AT3G03285			5552692	5553114	33.81
	AT3G16360	AHP4	AHP4 [Source:UniProtKB/TrEMBL;Acc:A0A384KT90]	5554197	5555789	33.33
	AT3G03295			5555487	5555698	34.91
	AT3G16370	APG	GDSL esterase/lipase APG [Source:UniProtKB/Swiss-Prot;Acc:Q9LU14]	5556710	5558982	38.54
	AT3G16380	PAB6	Polyadenylate-binding protein 6 [Source:UniProtKB/Swiss-Prot;Acc:O04319]	5558445	5561126	39.45
	AT3G16390	NSP3	Nitrile-specifier protein 3 [Source:UniProtKB/Swiss-Prot;Acc:O04318]	5562400	5564574	40.69
	AT3G16400	NSP1	Nitrile-specifier protein 1 [Source:UniProtKB/Swiss-Prot;Acc:Q9SDM9]	5566083	5568545	39.63
MQTL-4/Chr3	AT3G22990	LFR	Armadillo repeat-containing protein LFR [Source:UniProtKB/Swiss-Prot;Acc:Q9LS90]	8163941	8166350	39.88
	AT3G23000	CIPK7	CBL-interacting serine/threonine-protein kinase 7 [Source:UniProtKB/Swiss-Prot;Acc:Q9XIW0]	8172479	8174142	43.27
	AT3G23010	AtRLP36		8174341	8176911	38.55
	AT3G23020		Pentatricopeptide repeat-containing protein At3g23020 [Source:UniProtKB/Swiss-Prot;Acc:Q9LS88]	8177002	8180122	39.35
	AT3G23030	IAA2	Auxin-responsive protein [Source:UniProtKB/TrEMBL;Acc:A0A1I9LQ54]	8180646	8182095	37.59
	AT3G04375			8184871	8185112	38.84
	AT3G23050	IAA7	Auxin-responsive protein IAA7 [Source:UniProtKB/Swiss-Prot;Acc:Q38825]	8194606	8197161	34
	AT3G23060		Probable E3 ubiquitin protein ligase DRIPH [Source:UniProtKB/Swiss-Prot;Acc:Q9LS86]	8200636	8203335	38.52
	AT3G23070	CFM3A	CRM-domain containing factor CFM3A, chloroplastic/mitochondrial [Source:UniProtKB/Swiss-Prot;Acc:F4J2U9]	8203393	8207419	39.51
	AT3G23080		Membrane related protein-like [Source:UniProtKB/TrEMBL;Acc:Q9LS84]	8207287	8209329	40.23
	AT3G23090		TPX2 (Targeting protein for Xklp2) protein family [Source:UniProtKB/TrEMBL;Acc:A0A1I9LQY2]	8214289	8217206	35.23
	AT3G23100	XRCC4	DNA repair protein XRCC4 [Source:UniProtKB/Swiss-Prot;Acc:Q682V0]	8220682	8226400	37.8
	AT3G23110	AtRLP37	Receptor-like protein 37 [Source:UniProtKB/Swiss-Prot;Acc:Q9LS80]	8222364	8224871	40.23
	AT3G23120	AtRLP38	Receptor-like protein 38 [Source:UniProtKB/Swiss-Prot;Acc:Q9LS79]	8227222	8229619	40.7
	AT3G04385			8232927	8233189	44.87
	AT3G04395			8232927	8233323	43.32
	AT3G23122			8234261	8234588	40.55
	AT3G23123		Transmembrane protein [Source:UniProtKB/TrEMBL;Acc:A0A1I9LM80]	8234314	8234559	42.68
	AT3G23125	MIR173	MIR173; miRNA [Source:TAIR;Acc:AT3G23125]	8236153	8236254	38.24
	AT3G23130	SUP	Uncharacterized protein At3g23130 (Fragment) [Source:UniProtKB/TrEMBL;Acc:C0SVC6]	8242256	8243372	38.59
	AT3G23140	URO	URO [Source:UniProtKB/TrEMBL;Acc:A0A384L0A0]	8250519	8251483	39.9
	AT3G23145		ATP binding / aminoacyl-tRNA ligase/ nucleotide binding protein [Source:UniProtKB/TrEMBL;Acc:F4J2W0]	8252138	8253196	34.66
	AT3G23150	ETR2	Ethylene receptor 2 [Source:UniProtKB/Swiss-Prot;Acc:Q0WPQ2]	8254559	8257888	41.05
	AT3G23160		Plant/protein (DUF668) [Source:UniProtKB/TrEMBL;Acc:Q9LTD4]	8259774	8262089	38.04
	AT3G23165	LCR42	LCR42 [Source:UniProtKB/TrEMBL;Acc:A0A178VM71]	8264650	8265060	28.22
	AT3G23167	LCR39	Putative defensin-like protein 185 [Source:UniProtKB/Swiss-Prot;Acc:P82754]	8266218	8266585	31.79
	AT3G23170		At3g23170 [Source:UniProtKB/TrEMBL;Acc:Q9LTD3]	8267920	8268961	37.72
	AT3G04405			8270724	8270923	38
	AT3G23172		unknown protein; Ha. [Source:TAIR;Acc:AT3G23172]	8272138	8272657	37.31
	AT3G04415			8272862	8273270	34.47
	AT3G23175		HR-like lesion-inducing protein-like protein [Source:UniProtKB/TrEMBL;Acc:Q8GYI3]	8273772	8275565	34.28
	AT3G23180		HR-like lesion-inducing protein [Source:UniProtKB/TrEMBL;Acc:Q9LTD0]	8276808	8278561	33.41
	AT3G23190		At3g23190 [Source:UniProtKB/TrEMBL;Acc:Q9LTC9]	8279271	8280912	32.7
	AT3G23200		CASP-like protein 5B3 [Source:UniProtKB/Swiss-Prot;Acc:Q8L7R5]	8281338	8282786	35.89
	AT3G23210	BHLH34	Transcription factor bHLH34 [Source:UniProtKB/Swiss-Prot;Acc:Q9LTC7]	8283003	8285081	36.94
	AT3G23220	ERF095	Ethylene-responsive transcription factor ERF095 [Source:UniProtKB/Swiss-Prot;Acc:Q9LTC6]	8287942	8288602	42.36
	AT3G23230	ERF098	Ethylene-responsive transcription factor ERF098 [Source:UniProtKB/Swiss-Prot;Acc:Q9LTC5]	8289379	8290164	40.84
	AT3G23240	ERF1B	Ethylene-responsive transcription factor 1B [Source:UniProtKB/Swiss-Prot;Acc:Q8LDC8]	8295617	8296604	40.49
	AT3G23245		unknown protein; Ha. [Source:TAIR;Acc:AT3G23245]	8300381	8300833	48.79
	AT3G23250	MYB15	Transcription factor MYB15 [Source:UniProtKB/Swiss-Prot;Acc:Q9LTC4]	8309209	8310910	36.6
	AT3G23255		tRNA dimethylallyltransferase [Source:UniProtKB/TrEMBL;Acc:F4J2X9]	8311722	8313125	37.61
	AT3G23260		Putative F-box protein At3g23260 [Source:UniProtKB/Swiss-Prot;Acc:Q9LTC3]	8313334	8314575	39.21
	AT3G04425			8315002	8315400	39.85
	AT3G04435			8316010	8316335	30.98
	AT3G23270		Regulator of chromosome condensation (RCC1) family with FYVE zinc finger domain-containing protein [Source:UniProtKB/TrEMB	8316218	8320487	37.96
	AT3G23280	XBAT35	Putative E3 ubiquitin-protein ligase XBAT35 [Source:UniProtKB/Swiss-Prot;Acc:Q4FE47]	8321329	8324477	38.2
	AT3G23290	LSH4	LSH4 [Source:UniProtKB/TrEMBL;Acc:A0A178VHD2]	8326628	8328557	35.08
	AT3G23300		S-adenosyl-L-methionine-dependent methyltransferases superfamily protein [Source:UniProtKB/TrEMBL;Acc:A0A178VBP4]	8332797	8336235	38.18
	AT3G23310		Non-specific serine/threonine protein kinase [Source:UniProtKB/TrEMBL;Acc:Q9LW66]	8338871	8343737	36.26
	AT3G23320		Polynucleotidyl transferase, ribonuclease H-like superfamily protein [Source:UniProtKB/TrEMBL;Acc:Q9LW65]	8344629	8345204	41.15
	AT3G23325		Uncharacterized protein At3g23325 [Source:UniProtKB/Swiss-Prot;Acc:Q9LW64]	8345770	8347142	35.76
	AT3G23326	MIR853A	MIR853a; miRNA [Source:TAIR;Acc:AT3G23326]	8346367	8346656	41.38
	AT3G23330	PCMP-H32	Putative pentatricopeptide repeat-containing protein At3g23330 [Source:UniProtKB/Swiss-Prot;Acc:Q9LW63]	8347127	8349514	40.62
	AT3G23340	CKL10	Casein kinase 1-like protein 10 [Source:UniProtKB/Swiss-Prot;Acc:Q9LW62]	8350803	8354021	36
	AT3G23350		ENTH/VHS family protein [Source:UniProtKB/TrEMBL;Acc:Q9LW61]	8354077	8355410	36.28

AT3G23360		Putative protein phosphatase 2C-like protein 44 [Source:UniProtKB/Swiss-Prot;Acc:Q9LW60]	8355238	8356393	37.63
AT3G23370		RNA-binding (RRM/RBD/RNP motifs) family protein [Source:UniProtKB/TrEMBL;Acc:Q9LW59]	8357569	8361171	39.58
AT3G23380	RIC5	CRIB domain-containing protein RIC5 [Source:UniProtKB/Swiss-Prot;Acc:F4J424]	8373375	8375098	35.44
AT3G23390	RPL36AB	60S ribosomal protein L36a [Source:UniProtKB/Swiss-Prot;Acc:O23290]	8375333	8376436	35.42
AT3G23400	PAP6	Plastid-lipid-associated protein 6, chloroplastic [Source:UniProtKB/Swiss-Prot;Acc:Q9LW57]	8376444	8378364	39.25
AT3G23410	FAO3	Long-chain-alcohol oxidase FAO3 [Source:UniProtKB/Swiss-Prot;Acc:Q9LW56]	8382672	8386181	38.97
AT3G23420		F-box and associated interaction domains-containing protein [Source:TAIR;Acc:AT3G23420]	8386273	8387366	41.77
AT3G23430	PHO1	Phosphate transporter PHO1 [Source:UniProtKB/Swiss-Prot;Acc:Q8S403]	8387398	8393467	34.56
AT3G23440	EDA6	Embryo sac development arrest 6 [Source:UniProtKB/TrEMBL;Acc:Q9LW53]	8404062	8405289	40.23
AT3G23450		Transmembrane protein [Source:UniProtKB/TrEMBL;Acc:Q9LW52]	8410048	8412213	48.06
AT3G23460		S-adenosyl-L-methionine-dependent methyltransferases superfamily protein [Source:UniProtKB/TrEMBL;Acc:F4J432]	8412319	8414376	36.15
AT3G23470		Cyclopropane-fatty-acyl-phospholipid synthase [Source:UniProtKB/TrEMBL;Acc:A0A1I9LMW4]	8414616	8418784	35.24
AT3G23480		Cyclopropane-fatty-acyl-phospholipid synthase [Source:UniProtKB/TrEMBL;Acc:F4J434]	8418778	8422975	33.25
AT3G23490	CYN	Cyanate hydratase [Source:UniProtKB/TrEMBL;Acc:A0A178VD28]	8422649	8424652	37.82
AT3G04455			8426630	8426923	43.2
AT3G23510		Cyclopropane-fatty-acyl-phospholipid synthase [Source:UniProtKB/TrEMBL;Acc:Q8GZ28]	8427557	8433340	35.79
AT3G04475			8434558	8434871	39.81
AT3G04495			8434558	8435008	41.46
AT3G23530		AT3g23510/MEE5_5 [Source:UniProtKB/TrEMBL;Acc:Q8VZH1]	8436436	8442847	35.18
AT3G23540		Alpha/beta-Hydrolases superfamily protein [Source:UniProtKB/TrEMBL;Acc:Q6NKN2]	8443233	8448341	36.74
AT3G23550	DTX18	Protein DETOXIFICATION 18 [Source:UniProtKB/Swiss-Prot;Acc:Q9LUH3]	8448273	8450799	36.84
AT3G23560	DTX19	Protein DETOXIFICATION 19 [Source:UniProtKB/Swiss-Prot;Acc:Q9LUH2]	8453903	8456708	35.82
AT3G23570		Alpha/beta-Hydrolases superfamily protein [Source:UniProtKB/TrEMBL;Acc:Q94JY3]	8457767	8459990	36.92
AT3G23580	RNR2A	Ribonucleoside-diphosphate reductase small chain A [Source:UniProtKB/Swiss-Prot;Acc:P50651]	8459838	8462815	36.6
AT3G04505			8463156	8463539	32.81
AT3G04515			8464229	8464561	34.53
AT3G04535			8464538	8464801	37.88
AT3G23590	MED33A	Mediator of RNA polymerase II transcription subunit 33A [Source:UniProtKB/Swiss-Prot;Acc:Q9LUG9]	8467398	8473627	41.16
AT3G23600		AT3g23600/MDB19_9 [Source:UniProtKB/TrEMBL;Acc:Q9LUG8]	8473627	8476024	36.41
AT3G23605	PUX12	Plant UBX domain-containing protein 12 [Source:UniProtKB/Swiss-Prot;Acc:Q9LUG7]	8476482	8477392	35.68
AT3G04555			8477471	8477704	42.74
AT3G23610	DSPTP1	dual specificity protein phosphatase 1 [Source:TAIR;Acc:AT3G23610]	8477800	8479877	36.24
AT3G23620		Ribosome production factor 2 homolog [Source:UniProtKB/Swiss-Prot;Acc:Q9LUG5]	8480025	8482222	37.63
AT3G23630	IPT7	Adenylate isopentenyltransferase 7, mitochondrial [Source:UniProtKB/Swiss-Prot;Acc:Q94ID1]	8488695	8490266	39.89
AT3G04565			8490850	8491373	36.45
AT3G04575			8490884	8491275	37.5
AT3G23633		F-box associated ubiquitination effector family protein [Source:UniProtKB/TrEMBL;Acc:Q9LUG3]	8496440	8497281	35.63
AT3G23635	RTFL13	ROTUNDIFOLIA like 13 [Source:UniProtKB/TrEMBL;Acc:A8MRE9]	8497679	8498390	35.81
AT3G23637	DVL21	DVL family protein [Source:UniProtKB/TrEMBL;Acc:Q6IM80]	8499727	8499888	37.65
AT3G23640	HGL1	Heteroglycan glucosidase 1 [Source:UniProtKB/TrEMBL;Acc:A0A1I9LTS6]	8501556	8509649	37.23
AT3G23650		Kinase-like protein [Source:UniProtKB/TrEMBL;Acc:F4J460]	8510689	8511196	32.28
AT3G04585			8511333	8511982	37.38
AT3G23660		Sec23/Sec24 protein transport family protein [Source:UniProtKB/TrEMBL;Acc:F4J462]	8513453	8517944	38.74
AT3G23670	KIN12B	Kinesin-like protein KIN-12B [Source:UniProtKB/Swiss-Prot;Acc:Q8L7Y8]	8519136	8525289	39.57
AT3G23680		F-box associated ubiquitination effector family protein [Source:UniProtKB/TrEMBL;Acc:F4J465]	8526366	8527173	40.97
AT3G23685		F-box associated ubiquitination effector family protein [Source:UniProtKB/TrEMBL;Acc:F4J466]	8527239	8528317	41.89
AT3G23690	BHLH77	Transcription factor bHLH77 [Source:UniProtKB/Swiss-Prot;Acc:Q9LK48]	8528609	8531358	36.69
AT3G23700		AT3g23700/MYM9_3 [Source:UniProtKB/TrEMBL;Acc:Q9LK47]	8531374	8533834	37.22
AT3G23710	TIC22L	Tic22-III [Source:UniProtKB/TrEMBL;Acc:A0A178V7Q4]	8533848	8536541	38.01
AT3G23715	SCRL13	Putative defensin-like protein 252 [Source:UniProtKB/Swiss-Prot;Acc:P82632]	8536850	8537273	33.49
AT3G23727	SCRL12	Putative defensin-like protein 251 [Source:UniProtKB/Swiss-Prot;Acc:P82631]	8544798	8545613	29.04
AT3G23730	XTH16	Probable xyloglucan endotransglucosylase/hydrolase protein 16 [Source:UniProtKB/Swiss-Prot;Acc:Q8LG58]	8550037	8551403	37.53
AT3G23740		unknown protein; FUNCTIONS IN: molecular_function unknown; INVOLVED IN: biological_process unknown; LOCATED IN: chl	8551772	8554839	37.35
AT3G23750	TMK4	Receptor-like kinase TMK4 [Source:UniProtKB/Swiss-Prot;Acc:Q9LK43]	8558060	8561766	42
AT3G23760		Transferring glycosyl group transferase [Source:UniProtKB/TrEMBL;Acc:Q8L9Q7]	8562745	8564717	36.39
AT3G23770		Beta-1,3-glucanase [Source:UniProtKB/TrEMBL;Acc:Q9LK41]	8565494	8567496	42.14
AT3G23780	NRPD2	DNA-directed RNA polymerase subunit beta [Source:UniProtKB/TrEMBL;Acc:A0A178VA17]	8567572	8574209	39.94
AT3G23790	AAE16	Probable acyl-activating enzyme 16, chloroplastic [Source:UniProtKB/Swiss-Prot;Acc:Q9LK39]	8575051	8581331	37.29
AT3G23800	SBP3	SBP3 [Source:UniProtKB/TrEMBL;Acc:A0A178VEQ9]	8581446	8583987	41.07
AT3G23805	RALFL24	Protein RALF-like 24 [Source:UniProtKB/Swiss-Prot;Acc:Q9LK37]	8586359	8587271	37.9
AT3G23810	SAHH2	Adenosylhomocysteinase [Source:UniProtKB/TrEMBL;Acc:A0A178VEP9]	8587622	8589754	43.27
AT3G04595			8602098	8602302	36.1
AT3G23820	GAE6	UDP-glucuronate 4-epimerase 6 [Source:UniProtKB/Swiss-Prot;Acc:Q9LIS3]	8603212	8605810	41.44
AT3G23830	RBG4	GRP4 [Source:UniProtKB/TrEMBL;Acc:A0A178VD35]	8606429	8608297	37.08
AT3G23840	CER26L	Protein ECERIFERUM 26-like [Source:UniProtKB/Swiss-Prot;Acc:Q9LIS1]	8610979	8612742	42.52
AT3G23860		GTP-binding protein-like protein [Source:UniProtKB/TrEMBL;Acc:Q9LIS0]	8616804	8619285	35.13
AT3G23870		Probable magnesium transporter NIPA1 [Source:UniProtKB/Swiss-Prot;Acc:Q9LIR9]	8619192	8621949	34.05
AT3G23880		F-box/kelch-repeat protein At3g23880 [Source:UniProtKB/Swiss-Prot;Acc:Q9LIR8]	8621986	8623214	41.33
AT3G23890	TOP2	DNA topoisomerase 2 [Source:UniProtKB/Swiss-Prot;Acc:P30182]	8624636	8631392	39.63
AT3G23900		RNA recognition motif (RRM)-containing protein [Source:UniProtKB/TrEMBL;Acc:F4J5A7]	8631482	8636066	40.5
AT3G23910		HAPp48,5 protein (Fragment) [Source:UniProtKB/TrEMBL;Acc:P92952]	8636331	8639580	36.74
AT3G23920	BAM1	Beta-amylase [Source:UniProtKB/TrEMBL;Acc:A0A178VNG4]	8641181	8644449	41.11
AT3G23930		Troponin T, skeletal protein [Source:UniProtKB/TrEMBL;Acc:Q9LIR5]	8646746	8647947	36.69
AT3G23940	DHAD	Dihydroxy-acid dehydratase, chloroplastic [Source:UniProtKB/Swiss-Prot;Acc:Q9LIR4]	8648669	8652605	38.66
AT3G23950		Putative F-box protein At3g23950 [Source:UniProtKB/Swiss-Prot;Acc:Q9LIR3]	8652908	8654164	44.23
AT3G23955		F-box family protein [Source:UniProtKB/TrEMBL;Acc:F4J5B7]	8655023	8657439	39.93
AT3G23960		Putative F-box protein At3g23960 [Source:UniProtKB/Swiss-Prot;Acc:Q9LIR1]	8657736	8658980	40.96
AT3G23970		Putative F-box protein At3g23970 [Source:UniProtKB/Swiss-Prot;Acc:Q9LIR0]	8660447	8661688	44.36
AT3G23980	BLI	KOS1 [Source:UniProtKB/TrEMBL;Acc:A0A178VL19]	8662413	8668040	37.28
AT3G23990	CPN60	Chaperonin CPN60, mitochondrial [Source:UniProtKB/Swiss-Prot;Acc:P29197]	8668772	8672578	39.72
AT3G24000	PCMP-H87	Pentatricopeptide repeat-containing protein At3g24000, mitochondrial [Source:UniProtKB/Swiss-Prot;Acc:Q9LIQ7]	8672718	8674881	44.13
AT3G24010	ING1	PHD finger protein ING1 [Source:UniProtKB/Swiss-Prot;Acc:Q9LIQ6]	8675905	8678286	37.57
AT3G24020	DIR16	Dirigent protein [Source:UniProtKB/TrEMBL;Acc:A0A178V7A9]	8678678	8679656	44.13
AT3G04615			8678704	8679535	45.67
AT3G24030	THIM	Hydroxyethylthiazole kinase [Source:UniProtKB/Swiss-Prot;Acc:Q9LIQ4]	8679819	8680905	44.71
AT3G24040		Core-2/I-branching beta-1,6-N-acetylglucosaminyltransferase family protein [Source:UniProtKB/TrEMBL;Acc:Q9LIQ3]	8680877	8683289	40.65
AT3G24050	GATA1	GATA transcription factor 1 [Source:UniProtKB/Swiss-Prot;Acc:Q8LAU9]	8685739	8687726	40.39
AT3G24060		Plant self-incompatibility protein S1 family [Source:UniProtKB/TrEMBL;Acc:Q9LIQ2]	8689589	8690032	42.57
AT3G24065		Plant self-incompatibility protein S1 family [Source:UniProtKB/TrEMBL;Acc:Q9LIQ1]	8690574	8691155	37.29
AT3G24068		Plant self-incompatibility protein S1 family [Source:UniProtKB/TrEMBL;Acc:Q2V3T1]	8692191	8692676	38.68
AT3G24070		At3g24060 [Source:UniProtKB/TrEMBL;Acc:Q940K6]	8693061	8694770	43.39
AT3G24080		KRR1 family protein [Source:UniProtKB/TrEMBL;Acc:F4J5D3]	8694867	8697142	41.12
AT3G24090		GFAT [Source:UniProtKB/TrEMBL;Acc:A0A384L621]	8697685	8701052	38.21
AT3G24093		Paired amphipathic helix (PAH2) superfamily protein [Source:UniProtKB/TrEMBL;Acc:Q1G3L8]	8701322	8702466	37.82
AT3G24100		Putative uncharacterized protein AT3g24100 [Source:UniProtKB/TrEMBL;Acc:Q94K36]	8703220	8704311	36.54
AT3G24110		Calcium-binding EF-hand family protein [Source:TAIR;Acc:AT3G24110]	8704208	8705550	35.44
AT3G24120		Homeodomain-like superfamily protein [Source:TAIR;Acc:AT3G24120]	8705608	8708336	39.57
AT3G24130	PME29	Probable pectinesterase 29 [Source:UniProtKB/Swiss-Prot;Acc:Q4PSN0]	8711663	8713424	37.91
AT3G24140	FAMA	FMA [Source:UniProtKB/TrEMBL;Acc:A0A178VKX8]	8715118	8718211	35.16
AT3G04625			8718849	8719084	31.36
AT3G24150		Uncharacterized protein At3g24150 [Source:UniProtKB/TrEMBL;Acc:Q0WQ76]	8724097	8725986	40.53
AT3G24160	PMP	PMP [Source:UniProtKB/TrEMBL;Acc:A0A384KKU3]	8726056	8729427	36.89
AT3G24170	ATGR1	Glutathione reductase, cytosolic [Source:UniProtKB/Swiss-Prot;Acc:P48641]	8729354	8734624	36.2
AT3G24180		Non-lysosomal glucosylceramidase [Source:UniProtKB/TrEMBL;Acc:Q8VZ08]	8734611	8741819	38.94
AT3G24190		AT3g24190/MUJ8_17 [Source:UniProtKB/TrEMBL;Acc:Q9LRN0]	8743113	8747901	39.57
AT3G24200		Ubiquinone biosynthesis monooxygenase COQ6, mitochondrial [Source:UniProtKB/TrEMBL;Acc:F4J6I6]	8747831	8751685	38.18
AT3G04635			8751134	8751580	32.66
AT3G24210		Ankyrin repeat family protein [Source:UniProtKB/TrEMBL;Acc:F4J6I7]	8753385	8756151	39.07
AT3G24220	NCED6	9-cis-epoxycarotenoid dioxygenase NCED6, chloroplastic [Source:UniProtKB/Swiss-Prot;Acc:Q9LRM7]	8761419	8763303	46.47

	AT3G24225	CLE19	ESR19 [Source:UniProtKB/TrEMBL;Acc:A0A178VF65]	8768846	8769695	33.18
	AT3G04645			8770519	8770733	33.02
	AT3G24230		Probable pectate lyase 9 [Source:UniProtKB/Swiss-Prot;Acc:Q9LRM5]	8774541	8777612	37.01
	AT3G24240	RCH2	Receptor-like protein kinase 2 [Source:UniProtKB/Swiss-Prot;Acc:Q9LHP4]	8780238	8784453	41.67
	AT3G24250		At3g24250 [Source:UniProtKB/TrEMBL;Acc:Q6NPS5]	8786689	8787391	45.66
	AT3G24255		RNA-directed DNA polymerase (Reverse transcriptase)-related family protein [Source:UniProtKB/TrEMBL;Acc:A0A1I9LSY4]	8788814	8793371	39.47
	AT3G24260		Paired amphipathic helix Sin3-like protein [Source:UniProtKB/TrEMBL;Acc:Q9LK19]	8793837	8794961	41.6
	AT3G24270	APUM25	Putative pumilio homolog 25 [Source:UniProtKB/Swiss-Prot;Acc:Q9LK18]	8796541	8797031	41.75
	AT3G24280	SMAP2	Small acidic protein 2 [Source:UniProtKB/Swiss-Prot;Acc:Q1ECS0]	8798934	8799482	38.62
	AT3G24290	AMT1-5	Putative ammonium transporter 1 member 5 [Source:UniProtKB/Swiss-Prot;Acc:Q9LK16]	8801400	8802890	50.5
	AT3G24300	AMT1-3	Ammonium transporter 1 member 3 [Source:UniProtKB/Swiss-Prot;Acc:Q9SQH9]	8805519	8807460	46.76
MQTL-5/Chr3	AT3G06385			14539507	14539777	39.48
	AT3G42473	LCR47	Defensin-like protein 91 [Source:UniProtKB/Swiss-Prot;Acc:P82762]	14607080	14607971	29.93
	AT3G06395			14656219	14656528	30.32
	AT3G42550		Eukaryotic aspartyl protease family protein [Source:UniProtKB/TrEMBL;Acc:F4JF07]	14665728	14669135	36.5
	AT3G06405			14670455	14670951	35.01
	AT3G06415			14670574	14670951	35.71
	AT3G42560		Pentatricopeptide repeat (PPR) superfamily protein [Source:UniProtKB/TrEMBL;Acc:Q9M2A8]	14680265	14680760	36.69
	AT3G42565		Cysteine rich peptide [Source:UniProtKB/TrEMBL;Acc:A8MS80]	14683051	14683410	39.17
	AT3G42570		Peroxidase family protein [Source:UniProtKB/TrEMBL;Acc:F4JF10]	14689058	14690388	32.98
MQTL-6/Chr3	AT3G62270	BOR2	Probable boron transporter 2 [Source:UniProtKB/Swiss-Prot;Acc:Q9M1P7]	23042212	23046153	38.05
	AT3G62280		GDSL esterase/lipase At3g62280 [Source:UniProtKB/Swiss-Prot;Acc:Q6NLP7]	23049324	23051309	36.56
	AT3G62285		pre-tRNA [Source:TAIR;Acc:AT3G62285]	23051470	23051542	58.9
	AT3G62290	ARFA1E	ADP-ribosylation factor A1E [Source:UniProtKB/TrEMBL;Acc:Q9M1P5]	23051544	23053893	37.53
	AT3G62300	DUF7	DOMAIN OF UNKNOWN FUNCTION 724 7 [Source:TAIR;Acc:AT3G62300]	23054004	23057283	40.88
	AT3G62310		Probable pre-mRNA-splicing factor ATP-dependent RNA helicase DEAH2 [Source:UniProtKB/Swiss-Prot;Acc:Q9LZQ9]	23057240	23060731	41.24
	AT3G62320		Polynucleotidyl transferase, ribonuclease H-like superfamily protein [Source:UniProtKB/TrEMBL;Acc:Q9LZQ8]	23061805	23062398	44.61
	AT3G62330		At3g62330 [Source:UniProtKB/TrEMBL;Acc:Q9LZQ7]	23062639	23065796	39.46
	AT3G09765			23067220	23067466	32.79
	AT3G62340	WRKY68	WRKY transcription factor 68 [Source:UniProtKB/Swiss-Prot;Acc:Q93WV6]	23069489	23071207	39.03
	AT3G62350		F-box/associated interaction domain protein [Source:UniProtKB/TrEMBL;Acc:Q9LZQ5]	23071080	23071766	36.54
	AT3G62360		Carbohydrate-binding-like fold [Source:UniProtKB/TrEMBL;Acc:Q9LZQ4]	23072746	23080706	37.28
	AT3G62370		Heme binding protein [Source:UniProtKB/TrEMBL;Acc:Q9LZQ3]	23080706	23083528	40.21
	AT3G62380		F-box/associated interaction domain protein [Source:UniProtKB/TrEMBL;Acc:Q9LZQ2]	23083523	23085035	34.3
	AT3G62390	TBL6	Protein trichome birefringence-like 6 [Source:UniProtKB/Swiss-Prot;Acc:Q9LZQ1]	23086989	23089357	38.96
	AT3G62400		unknown protein; Ha. [Source:TAIR;Acc:AT3G62400]	23089596	23090821	37.28
	AT3G62410	CP12-2	Calvin cycle protein CP12-2, chloroplastic [Source:UniProtKB/Swiss-Prot;Acc:Q9LZP9]	23090944	23091580	45.05
	AT3G62420	BZIP53	bZIP transcription factor 53 [Source:UniProtKB/Swiss-Prot;Acc:Q9LZP8]	23091586	23092850	37.71
	AT3G62430		F-box protein At3g62430 [Source:UniProtKB/Swiss-Prot;Acc:Q9LZP7]	23093451	23095657	39.37
	AT3G62440		SAF1 [Source:UniProtKB/TrEMBL;Acc:A0A178VA18]	23096104	23097726	40.17
	AT3G62450		DNA mismatch repair protein [Source:UniProtKB/TrEMBL;Acc:Q9LZP5]	23098019	23099070	35.36
	AT3G62460		AT3g62460/T12C14_160 [Source:UniProtKB/TrEMBL;Acc:Q9LZP4]	23104665	23105650	41.68
	AT3G62470		Pentatricopeptide repeat-containing protein At3g62470, mitochondrial [Source:UniProtKB/Swiss-Prot;Acc:Q9LZP3]	23106437	23108694	42.69
	AT3G09775			23116274	23116904	33.76
	AT3G62499		YTH family protein [Source:UniProtKB/TrEMBL;Acc:Q1G393]	23119844	23120149	38.24
	AT3G62500		F-box protein RMF [Source:UniProtKB/TrEMBL;Acc:A0A1I9LQ20]	23120423	23123188	38.65
	AT3G62510		Disulfide isomerase-like protein [Source:UniProtKB/TrEMBL;Acc:A0A1I9LTP0]	23122831	23123802	36.52
	AT3G09785			23129280	23129525	39.43
	AT3G62528		unknown protein; Ha. [Source:TAIR;Acc:AT3G62528]	23131482	23131655	47.13
	AT3G62530		ARM repeat superfamily protein [Source:UniProtKB/TrEMBL;Acc:Q94K48]	23132122	23133298	38.32
	AT3G62540		Pentatricopeptide repeat-containing protein At3g62540, mitochondrial [Source:UniProtKB/Swiss-Prot;Acc:Q3EAF8]	23133330	23135384	43.41
	AT3G62550		Adenine nucleotide alpha hydrolases-like superfamily protein [Source:UniProtKB/TrEMBL;Acc:Q93W91]	23135630	23137173	33.16
	AT3G62560		Putative Sar1 GTP binding protein [Source:UniProtKB/TrEMBL;Acc:Q8VYP7]	23137286	23139145	38.55
	AT3G62570		AT3g62570/T12C14_270 [Source:UniProtKB/TrEMBL;Acc:Q8W591]	23142485	23144747	41.54
	AT3G62580		Late embryogenesis abundant protein (LEA) family protein [Source:UniProtKB/TrEMBL;Acc:F4IYB7]	23146809	23147951	41.03
	AT3G09795			23147450	23147836	40.31
	AT3G62590	PLIP3	Phospholipase A1 PLIP3, chloroplastic [Source:UniProtKB/Swiss-Prot;Acc:Q940L4]	23147918	23150606	42.51
	AT3G09815			23150247	23150606	36.39
	AT3G62600	ERDJ3B	DnaJ protein ERDJ3B [Source:UniProtKB/Swiss-Prot;Acc:Q9LZK5]	23150779	23153649	38.56
	AT3G62610	MYB11	Transcription factor MYB11 [Source:UniProtKB/Swiss-Prot;Acc:Q9LZK4]	23154630	23156585	38.14
	AT3G62615			23155360	23155854	38.59
	AT3G09825			23156946	23157188	36.21
	AT3G09835			23156983	23157404	36.26
	AT3G09845			23157845	23158090	41.46
	AT3G62620		At3g62620 [Source:UniProtKB/TrEMBL;Acc:Q29Q18]	23159656	23161949	37.75
	AT3G62630		Stress response NST1-like protein (DUF1645) [Source:UniProtKB/TrEMBL;Acc:Q9LZK2]	23163747	23165387	41.56
	AT3G62640		At3g62640 [Source:UniProtKB/TrEMBL;Acc:Q9LZK1]	23166219	23166979	40.74
	AT3G62650		Uncharacterized protein At3g62650; F26K9_80 [Source:UniProtKB/TrEMBL;Acc:Q9LZK0]	23168061	23169187	37.71
	AT3G62660	GATL7	Probable galacturonosyltransferase-like 7 [Source:UniProtKB/Swiss-Prot;Acc:Q8VYF4]	23172754	23174993	43.48
	AT3G62670	ARR20	Putative two-component response regulator ARR20 [Source:UniProtKB/Swiss-Prot;Acc:Q9LZJ8]	23176556	23178294	39.74
	AT3G09855			23181621	23182015	33.16
	AT3G09865			23181704	23181833	36.92
	AT3G62680	PRP3	Proline-rich protein 3 [Source:UniProtKB/Swiss-Prot;Acc:Q9LZJ7]	23182722	23184042	44.06
	AT3G62690	ATL5	RING-H2 zinc finger protein ATL5 [Source:UniProtKB/TrEMBL;Acc:Q0WRF5]	23185348	23186720	44.87
	AT3G62695			23185929	23186129	53.23
	AT3G62700	ABCC14	ABC transporter C family member 14 [Source:UniProtKB/Swiss-Prot;Acc:Q9LZJ5]	23190265	23195917	42.35
	AT3G62710		Beta-D-glucan exohydrolase-like protein [Source:UniProtKB/TrEMBL;Acc:Q9LZJ4]	23197574	23200723	40.7
	AT3G62720	XXT1	XXT1 [Source:UniProtKB/TrEMBL;Acc:A0A178VEJ0]	23201032	23202988	43.23
	AT3G62730		AT3g62730/F26K9_160 [Source:UniProtKB/TrEMBL;Acc:Q9LZJ2]	23207854	23209533	38.75
	AT3G62735		pre-tRNA [Source:TAIR;Acc:AT3G62735]	23210707	23210777	56.34
	AT3G62740	BGLU7	Beta-glucosidase 7 [Source:UniProtKB/Swiss-Prot;Acc:Q9LZJ1]	23211287	23214066	37.3
	AT3G62750	BGLU8	Beta glucosidase 8 [Source:UniProtKB/TrEMBL;Acc:A0A1I9LTV6]	23214247	23217084	36.89
	AT3G62760	GSTF13	Glutathione S-transferase F13 [Source:UniProtKB/Swiss-Prot;Acc:Q9LZI9]	23217233	23218312	42.5
	AT3G62770	ATG18A	Autophagy-related protein 18a [Source:UniProtKB/Swiss-Prot;Acc:Q93VB2]	23218610	23221243	41.91
	AT3G62780		At3g62780 [Source:UniProtKB/TrEMBL;Acc:Q9LZI7]	23221719	23222995	45.58
	AT3G62790		NADH dehydrogenase [ubiquinone] iron-sulfur protein 5-B [Source:UniProtKB/Swiss-Prot;Acc:Q9LZI6]	23223030	23224326	37.55
	AT3G62800	DBR4	Double-stranded RNA-binding protein 4 [Source:UniProtKB/Swiss-Prot;Acc:Q8H1D4]	23225526	23227512	38.8
	AT3G62810		At3g62810 [Source:UniProtKB/TrEMBL;Acc:Q9LZI4]	23227622	23229027	40.68
	AT3G62820		Plant invertase/pectin methylesterase inhibitor superfamily protein [Source:UniProtKB/TrEMBL;Acc:Q9LZI3]	23229381	23230575	39.67
	AT3G62830	UXS2	UDP-glucuronic acid decarboxylase 2 [Source:UniProtKB/Swiss-Prot;Acc:Q9LZI2]	23232272	23235567	40.81
	AT3G09875			23232375	23233105	46.65
	AT3G62840		Putative small nuclear ribonucleoprotein D2 [Source:UniProtKB/TrEMBL;Acc:Q8RUH0]	23235490	23236816	37.91
	AT3G62850		Zinc finger protein-like protein [Source:UniProtKB/TrEMBL;Acc:Q9LZI1]	23237487	23239155	45.54
	AT3G62860		Alpha/beta-Hydrolases superfamily protein [Source:UniProtKB/TrEMBL;Acc:F4IZK0]	23239264	23242274	36.17
	AT3G62870	RPL7AB	60S ribosomal protein L7a-2 [Source:UniProtKB/Swiss-Prot;Acc:Q9LZH9]	23242561	23244397	40.34
	AT3G62880	OEP164	ATOEP16-4 [Source:UniProtKB/TrEMBL;Acc:A0A178VCJ7]	23244614	23246066	37.65
	AT3G62890	PCMP-H82	Pentatricopeptide repeat-containing protein At3g62890 [Source:UniProtKB/Swiss-Prot;Acc:Q683I9]	23246129	23248063	42.69
	AT3G62900		CW-type Zinc Finger [Source:UniProtKB/TrEMBL;Acc:F4IZK5]	23248129	23255297	40.35
	AT3G62910	APG3	Peptide chain release factor APG3, chloroplastic [Source:UniProtKB/Swiss-Prot;Acc:Q8RX79]	23257404	23260480	39.26
	AT3G62920		Zinc metalloproteinase aureolysin [Source:UniProtKB/TrEMBL;Acc:Q9LYC9]	23260597	23261297	36.66
	AT3G62930	GRXS6	Monothiol glutaredoxin-S6 [Source:UniProtKB/Swiss-Prot;Acc:Q9LYC8]	23261442	23261927	41.98
	AT3G62940		Cysteine proteinases superfamily protein [Source:UniProtKB/TrEMBL;Acc:Q9LYC7]	23262795	23264731	40.27
	AT3G62950	GRXC11	Glutaredoxin-C11 [Source:UniProtKB/Swiss-Prot;Acc:Q9LYC6]	23266227	23267090	38.66
	AT3G62960	GRXC14	Glutaredoxin-C14 [Source:UniProtKB/Swiss-Prot;Acc:Q9LYC5]	23268641	23269324	40.35
	AT3G62970		Zinc finger (C3HC4-type RING finger) family protein [Source:UniProtKB/TrEMBL;Acc:F4IZL5]	23270403	23272992	37.3
	AT3G62980	TIR1	TIR1 [Source:UniProtKB/TrEMBL;Acc:A0A178VA54]	23273065	23276630	42.18
	AT3G62990		At3g62990 [Source:UniProtKB/TrEMBL;Acc:A0JPZ2]	23282583	23283368	36.26
	AT3G63000	NPL41	NPL4-like protein 1 [Source:UniProtKB/Swiss-Prot;Acc:Q9LYC2]	23283592	23285540	43.71
	AT3G63003		pre-tRNA [Source:TAIR;Acc:AT3G63003]	23285593	23285665	60.27

AT3G63006		pre-tRNA [Source:TAIR;Acc:AT3G63006]	23285754	23285826	60.27
AT3G63010	GID1B	Gibberellin receptor GID1B [Source:UniProtKB/Swiss-Prot;Acc:Q9LYC1]	23289243	23291486	41.84
AT3G63020		Uncharacterized protein T20O10_120 [Source:UniProtKB/TrEMBL;Acc:Q9LYC0]	23294382	23295143	47.9
AT3G63030	MBD4	Methyl-CpG-binding domain-containing protein 4 [Source:UniProtKB/Swiss-Prot;Acc:Q9LYB9]	23295285	23296626	40.31
AT3G63040		At3g63040 [Source:UniProtKB/TrEMBL;Acc:Q9LYB8]	23296572	23297225	42.2
AT3G63050		unknown protein; BEST Arabidopsis thaliana protein match is: unknown protein (TAIR:AT2G48075.1); Ha. [Source:TAIR;Acc:AT3C	23297744	23298534	36.41
AT3G63052		unknown protein; Ha. [Source:TAIR;Acc:AT3G63052]	23298729	23299126	44.47
AT3G63060	EDL3	EID1-like F-box protein 3 [Source:UniProtKB/Swiss-Prot;Acc:Q93ZT5]	23300290	23301865	48.98
AT3G63070	HULK3	Protein HUA2-LIKE 3 [Source:UniProtKB/Swiss-Prot;Acc:F4IZM8]	23302365	23309658	40.72
AT3G63080	GPX5	Probable glutathione peroxidase 5 [Source:UniProtKB/Swiss-Prot;Acc:Q9LYB4]	23309737	23311397	37.75
AT3G63088	RTFL14	DVL14 [Source:UniProtKB/TrEMBL;Acc:Q6IM87]	23313004	23313388	34.03
AT3G63090		Ubiquitin carboxyl-terminal hydrolase family protein [Source:UniProtKB/TrEMBL;Acc:Q9LYB3]	23313428	23315062	42.81
AT3G63093			23315085	23316615	45.13
AT3G63095		Tetratricopeptide repeat (TPR)-like superfamily protein [Source:UniProtKB/TrEMBL;Acc:A8MRN4]	23315506	23316258	53.92
AT3G63110	IPT3	IPT3 [Source:UniProtKB/TrEMBL;Acc:A0A178V8J2]	23317971	23319636	42.74
AT3G63120	CYCU3-1	Cyclin-U3-1 [Source:UniProtKB/Swiss-Prot;Acc:Q8LB60]	23322359	23324283	42.81
AT3G63130	RANGAP1	RAN GTPase-activating protein 1 [Source:UniProtKB/Swiss-Prot;Acc:Q9LE82]	23324479	23326973	42.08
AT3G09895			23326001	23326218	45.41
AT3G63140	CSP41A	Chloroplast stem-loop binding protein of 41 kDa a, chloroplastic [Source:UniProtKB/Swiss-Prot;Acc:Q9LYA9]	23326867	23328789	44.93
AT3G09905			23328322	23328607	47.2
AT3G63150	MIRO2	Mitochondrial Rho GTPase [Source:UniProtKB/TrEMBL;Acc:A0A178VC39]	23328916	23333078	39.15
AT3G63160		OEP6 [Source:UniProtKB/TrEMBL;Acc:A0A384KZL8]	23333399	23334034	38.99
AT3G63170	FAP1	Fatty-acid-binding protein 1 [Source:UniProtKB/Swiss-Prot;Acc:Q9M1X2]	23334574	23336081	42.64
AT3G63180	ATTKL	TIC-like protein [Source:UniProtKB/TrEMBL;Acc:F4J0W8]	23336092	23340340	44.48
AT3G63190	RRF	Ribosome-recycling factor, chloroplastic [Source:UniProtKB/Swiss-Prot;Acc:Q9M1X0]	23342550	23344801	39.61
AT3G63200	PLP9	Probable inactive patatin-like protein 9 [Source:UniProtKB/Swiss-Prot;Acc:Q93ZQ3]	23345754	23347672	47.47
AT3G09935			23352294	23352511	33.94
AT3G63210	MARD1	Protein MARD1 [Source:UniProtKB/Swiss-Prot;Acc:Q8LGS1]	23353514	23355480	40.67
AT3G63215			23353991	23354340	44
AT3G63220	SKIP30	F-box/kelch-repeat protein SKIP30 [Source:UniProtKB/Swiss-Prot;Acc:Q9M1W7]	23357328	23359291	45.67
AT3G63230		Senescence-associated-like protein (DUF581) [Source:UniProtKB/TrEMBL;Acc:F4J0X4]	23359633	23360338	39.66
AT3G63240	IP5P4	Type I inositol polyphosphate 5-phosphatase 4 [Source:UniProtKB/Swiss-Prot;Acc:Q8GTS0]	23363872	23367592	41.76
AT3G09945			23368032	23368429	33.92
AT3G63250	HMT-2	Homocysteine S-methyltransferase 2 [Source:UniProtKB/Swiss-Prot;Acc:Q9M1W4]	23369781	23372823	40.32
AT3G63255			23372870	23373545	43.49
AT3G63260	ATMRK1	ATMRK1 [Source:UniProtKB/TrEMBL;Acc:O22100]	23372878	23375405	44.58
AT3G63270	ALP1	Protein ANTAGONIST OF LIKE HETEROCHROMATIN PROTEIN 1 [Source:UniProtKB/Swiss-Prot;Acc:Q94K49]	23375584	23377661	45.72
AT3G63280	ATNEK4	NIMA-related kinase 4 [Source:UniProtKB/TrEMBL;Acc:A0A1I9LPR2]	23377976	23381590	40.19
AT3G63290		2-oxoglutarate (2OG) and Fe(II)-dependent oxygenase superfamily protein [Source:UniProtKB/TrEMBL;Acc:Q8VYD7]	23381604	23383538	43.98
AT3G63300	VAB	VAN3-binding protein [Source:UniProtKB/Swiss-Prot;Acc:Q8W4K5]	23384988	23387842	43.96
AT3G63310	BIL4	Protein LIFEGUARD 2 [Source:UniProtKB/Swiss-Prot;Acc:Q9M1V9]	23387718	23389038	43.75
AT3G63320		Putative protein phosphatase 2C 50 [Source:UniProtKB/Swiss-Prot;Acc:Q9M1V8]	23389348	23391593	42.92

MQTLs	Gene stable ID	Gene name	Gene description	Gene start (bp)	Gene end (bp)	Gene % GC content
MQTL-1/Chr4	AT4G00950	MEE47	Uncharacterized protein At4g00950 [Source:UniProtKB/Swiss-Prot;Acc:Q9M160]	405623	407142	37.76
	AT4G00953			410090	410816	46.77
	AT4G00955		FUNCTIONS IN: molecular_function unknown; INVOLVED IN: biological_process unknown; LOCATED IN: endomembrane system; E	412107	413810	38.97
	AT4G00960		Putative receptor-like protein kinase At4g00960 [Source:UniProtKB/Swiss-Prot;Acc:O23082]	414204	416355	32.95
	AT4G00970	CRK41	Cysteine-rich receptor-like protein kinase 41 [Source:UniProtKB/Swiss-Prot;Acc:O23081]	418327	421885	37.43
	AT4G00975		other RNA [Source:TAIR;Acc:AT4G00975]	421973	423258	41.29
	AT4G00980		At4g00980 [Source:UniProtKB/TrEMBL;Acc:Q8GXX5]	422478	424588	41.78
	AT4G00985		pre-tRNA [Source:TAIR;Acc:AT4G00985]	424717	424788	47.22
	AT4G03805			426018	426395	42.86
	AT4G03815			426053	426395	44.61
	AT4G00990		Transcription factor jumonji (JmjC) domain-containing protein [Source:UniProtKB/TrEMBL;Acc:Q8VYB9]	426847	431862	38.26
	AT4G01000		AT4g01000/F3I3_20 [Source:UniProtKB/TrEMBL;Acc:Q9SV28]	431516	434029	38.86
	AT4G01010	CNGC13	Putative cyclic nucleotide-gated ion channel 13 [Source:UniProtKB/Swiss-Prot;Acc:Q9LD40]	434217	437605	38.27
	AT4G01020		ATP-dependent RNA helicase DEAH11, chloroplastic [Source:UniProtKB/Swiss-Prot;Acc:P0CE10]	438991	445218	42.63
	AT4G01023		RING/U-box superfamily protein [Source:TAIR;Acc:AT4G01023]	445422	446948	39.16
	AT4G01026	PYL7	RCAR2 [Source:UniProtKB/TrEMBL;Acc:A0A178UTD1]	446978	448511	39.63
	AT4G03845			447309	447577	35.32
	AT4G01030	PCMP-H65	Pentatricopeptide repeat-containing protein At4g01030, mitochondrial [Source:UniProtKB/Swiss-Prot;Acc:Q9SV26]	447941	450642	41.78
	AT4G01037	WTF1	WTF1 [Source:UniProtKB/TrEMBL;Acc:A0A178UW54]	451194	453243	41.07
	AT4G01040		Glycosyl hydrolase superfamily protein [Source:UniProtKB/TrEMBL;Acc:Q9M159]	453347	455611	39.91
	AT4G01050	TROL	thylakoid rhodanese-like [Source:TAIR;Acc:AT4G01050]	455583	458510	40.03
	AT4G01060	ETC3	CAPRICE-like MYB3 [Source:TAIR;Acc:AT4G01060]	460395	461246	35.21
	AT4G01070	UGT72B1	UDP-glycosyltransferase 72B1 [Source:UniProtKB/Swiss-Prot;Acc:Q9M156]	461513	463556	42.47
	AT4G01080	TBL26	Mannan O-acetyltransferase 4 [Source:UniProtKB/TrEMBL;Acc:A0A346P851]	466298	468294	40.51
	AT4G03855			469398	469635	37.39
	AT4G01090		AT4G01090 protein [Source:UniProtKB/TrEMBL;Acc:Q9M155]	470506	473879	40.4
	AT4G01100	ADNT1	Adenine nucleotide transporter 1 [Source:UniProtKB/Swiss-Prot;Acc:F4JHS4]	477141	480028	39.09
	AT4G01110		Late embryogenesis abundant hydroxyproline-rich glycoprotein family protein [Source:UniProtKB/TrEMBL;Acc:Q5XV90]	479963	481160	42.32
	AT4G01120	GBF2	G-box-binding factor 2 [Source:UniProtKB/Swiss-Prot;Acc:P42775]	481569	484524	37.92
	AT4G01130		GDSL esterase/lipase At4g01130 [Source:UniProtKB/Swiss-Prot;Acc:Q9M153]	485743	488226	34.58
	AT4G01140		At4g01140 [Source:UniProtKB/TrEMBL;Acc:Q9M152]	490749	492418	36.65
	AT4G01150	CURT1A	Protein CURVATURE THYLAKOID 1A, chloroplastic [Source:UniProtKB/Swiss-Prot;Acc:O04616]	493546	494885	39.03
	AT4G01160		LRB3 [Source:UniProtKB/TrEMBL;Acc:A0A178UYH5]	494958	497006	38.07
	AT4G01170		Uncharacterized protein AT4g01170 [Source:UniProtKB/TrEMBL;Acc:Q9M151]	499497	500831	40.07
	AT4G01180		XH/XS domain-containing protein [Source:UniProtKB/TrEMBL;Acc:Q9M150]	501287	503394	39.23
	AT4G01190	ATPIPK10	phosphatidylinositol phosphate kinase 10 [Source:TAIR;Acc:AT4G01190]	504135	506403	34.77
	AT4G01200		Calcium-dependent lipid-binding (CaLB domain) family protein [Source:UniProtKB/TrEMBL;Acc:Q9M148]	506511	507626	46.77
	AT4G01210		Glycosyl transferase family 1 protein [Source:UniProtKB/TrEMBL;Acc:F4JHZ4]	507648	512852	38.96
	AT4G01220	MGP4	UDP-D-xylose:L-fucose alpha-1,3-D-xylosyltransferase MGP4 [Source:UniProtKB/Swiss-Prot;Acc:Q9M146]	513053	515853	37.84
	AT4G01230	RTNLB7	Reticulon-like protein B7 [Source:UniProtKB/Swiss-Prot;Acc:Q9M145]	516021	517516	39.57
	AT4G01240		S-adenosyl-L-methionine-dependent methyltransferases superfamily protein [Source:UniProtKB/TrEMBL;Acc:Q9M144]	517795	521972	36.31
	AT4G01250	WRKY22	WRKY transcription factor 22 [Source:UniProtKB/Swiss-Prot;Acc:O04609]	522530	524249	40.12
	AT4G01245		FUNCTIONS IN: molecular_function unknown; INVOLVED IN: biological_process unknown; LOCATED IN: endomembrane system; E	527591	528144	37
	AT4G01260		Probable transcription factor At4g01260 [Source:UniProtKB/Swiss-Prot;Acc:O04608]	528570	529547	40.7
	AT4G01270		Putative RING zinc finger protein [Source:UniProtKB/TrEMBL;Acc:Q9M143]	532247	534984	38.17
	AT4G01280	RVE5	Protein REVEILLE 5 [Source:UniProtKB/Swiss-Prot;Acc:C0SVG5]	535198	538098	39.57
	AT4G01290		Chorismate synthase [Source:UniProtKB/TrEMBL;Acc:Q56YR0]	537418	543939	40.48
	AT4G01310	RPL5	50S ribosomal protein L5, chloroplastic [Source:UniProtKB/Swiss-Prot;Acc:O04603]	543911	545566	41.3
	AT4G01320	FACE1	CAAX prenyl protease 1 homolog [Source:UniProtKB/Swiss-Prot;Acc:Q8RX88]	545796	549413	37.23
	AT4G01330		Protein kinase superfamily protein [Source:UniProtKB/TrEMBL;Acc:F4JI10]	550175	553097	39.86
	AT4G01335		FUNCTIONS IN: molecular_function unknown; INVOLVED IN: biological_process unknown; LOCATED IN: endomembrane system; E	553332	554187	32.83
	AT4G01340		CHP-rich zinc finger protein-like protein [Source:UniProtKB/TrEMBL;Acc:Q9M138]	554539	556961	39.37
	AT4G01350		Cysteine/Histidine-rich C1 domain family protein [Source:UniProtKB/TrEMBL;Acc:F4JI14]	560146	562438	40.95
	AT4G01355		pre-tRNA [Source:TAIR;Acc:AT4G01355]	562561	562634	43.24
	AT4G01360		BPS1-like protein [Source:UniProtKB/TrEMBL;Acc:F4JI15]	564760	566408	39.84
	AT4G01370	MPK4	Mitogen-activated protein kinase 4 [Source:UniProtKB/Swiss-Prot;Acc:Q39024]	566917	570299	39.88
	AT4G01380		Plastocyanin-like domain-containing protein [Source:UniProtKB/TrEMBL;Acc:Q9M135]	569148	570076	45.21
	AT4G01390		TRAF-like family protein [Source:UniProtKB/TrEMBL;Acc:F4JI18]	570200	571595	38.68
	AT4G01395	COG4	Conserved oligomeric Golgi complex subunit 4 [Source:UniProtKB/Swiss-Prot;Acc:Q8L838]	572987	575773	43.6
	AT4G01400		Pentatricopeptide repeat-containing protein At4g01400, mitochondrial [Source:UniProtKB/Swiss-Prot;Acc:Q8LDU5]	575756	577303	40.96
	AT4G01410		At4g01410 [Source:UniProtKB/TrEMBL;Acc:Q9M132]	578028	579287	40.48
	AT4G01420	CBL5	Calcineurin B-like protein 5 [Source:UniProtKB/Swiss-Prot;Acc:Q7FZF1]	580038	581521	31.47
	AT4G01430		WAT1-related protein At4g01430 [Source:UniProtKB/Swiss-Prot;Acc:Q9M131]	585598	588126	32.46
	AT4G01435			591825	592592	36.85
	AT4G01440		WAT1-related protein At4g01440 [Source:UniProtKB/Swiss-Prot;Acc:Q9M130]	596397	598752	32.72
	AT4G01450		WAT1-related protein At4g01450 [Source:UniProtKB/Swiss-Prot;Acc:Q9M129]	608273	610765	32.53
	AT4G01460	BHLH57	Transcription factor bHLH57 [Source:UniProtKB/Swiss-Prot;Acc:Q9M128]	620691	623000	34.98
	AT4G01470	TIP1-3	Aquaporin TIP1-3 [Source:UniProtKB/Swiss-Prot;Acc:O82598]	624786	625885	43
	AT4G01480	PPA5	PPa5 [Source:UniProtKB/TrEMBL;Acc:A0A178V127]	626069	628070	34.77
	AT4G03875			630579	630795	49.77
	AT4G01500	NGA4	NGA4 [Source:UniProtKB/TrEMBL;Acc:A0A178V2Z9]	639247	640976	40.23
	AT4G01510	ARV2	Arv1-like protein [Source:TAIR;Acc:AT4G01510]	642714	644400	34.56
	AT4G01516		unknown protein; Ha. [Source:TAIR;Acc:AT4G01516]	649862	649957	54.17
	AT4G01520	anac067	F11O4.3 [Source:UniProtKB/TrEMBL;Acc:O82593]	656407	659178	33.77
	AT4G01533		other RNA [Source:TAIR;Acc:AT4G01533]	667148	669073	37.44
	AT4G01535		unknown protein; FUNCTIONS IN: molecular_function unknown; INVOLVED IN: biological_process unknown; LOCATED IN: endom	669339	669830	41.06
	AT4G01540	NAC68	NAC domain-containing protein 68 [Source:UniProtKB/Swiss-Prot;Acc:A8MQY1]	670483	672789	39.14
	AT4G01550	NAC69	NAC domain-containing protein 69 [Source:UniProtKB/Swiss-Prot;Acc:Q9M126]	673862	676445	37.77
	AT4G01560	MEE49	AT4g01560/F11O4_6 [Source:UniProtKB/TrEMBL;Acc:Q9M125]	676982	679276	35.95
	AT4G01570		Pentatricopeptide repeat-containing protein At4g01570 [Source:UniProtKB/Swiss-Prot;Acc:Q8VZE4]	679472	681940	42
	AT4G01575		Serine protease inhibitor, Kazal-type family protein [Source:UniProtKB/TrEMBL;Acc:F4JG23]	681862	682601	38.51
	AT4G03895			681862	682660	37.67
	AT4G01580		B3 domain-containing protein At4g01580 [Source:UniProtKB/Swiss-Prot;Acc:Q9ZSH7]	685670	686886	38.7
	AT4G03905			687493	687992	36.4
	AT4G03915			687715	687867	33.99
	AT4G03925			687898	688053	37.82
	AT4G01590		DNA-directed RNA polymerase III subunit [Source:UniProtKB/TrEMBL;Acc:Q9M123]	688813	690460	35.8
	AT4G01593		other RNA [Source:TAIR;Acc:AT4G01593]	690643	692176	36.57
	AT4G01595		Protein kinase superfamily protein [Source:UniProtKB/TrEMBL;Acc:F4JG28]	690726	691993	37.62
	AT4G01600		GEM-like protein 2 [Source:UniProtKB/Swiss-Prot;Acc:Q9M122]	692853	694381	37.28
	AT4G01610	CATHB3	Cathepsin B-like protease 3 [Source:UniProtKB/Swiss-Prot;Acc:Q94K85]	694582	697206	34.82
	AT4G01630	EXPA17	Expansin [Source:UniProtKB/TrEMBL;Acc:A0A178UWZ2]	700566	701769	40.03
	AT4G01640		F-box associated ubiquitination effector family protein [Source:UniProtKB/TrEMBL;Acc:Q9M121]	702563	703600	41.43
	AT4G01650		Polyketide cyclase / dehydrase and lipid transport protein [Source:UniProtKB/TrEMBL;Acc:Q9M120]	704377	707045	33.16
	AT4G01660	ABC1	Protein ABC transporter 1, mitochondrial [Source:UniProtKB/Swiss-Prot;Acc:Q9SBB2]	708357	711256	40.9
	AT4G01670		AT4G01670 protein [Source:UniProtKB/TrEMBL;Acc:Q8GXD3]	711192	712643	37.19
	AT4G03955			712342	712526	44.32
	AT4G01671		unknown protein; LOCATED IN: endomembrane system; Ha. [Source:TAIR;Acc:AT4G01671]	713216	713389	39.08
	AT4G01680	MYB55	Myb domain protein 55 [Source:UniProtKB/TrEMBL;Acc:Q9ZSI4]	715970	717603	35.43
	AT4G01690	PPOX1	Protoporphyrinogen oxidase [Source:UniProtKB/TrEMBL;Acc:A0A178UVN5]	729863	732433	41.73
	AT4G01700		At4g01700 [Source:UniProtKB/TrEMBL;Acc:Q9ZSI6]	732313	733510	41.24
	AT4G03985			733149	733419	50.55
	AT4G01703		unknown protein; Ha. [Source:TAIR;Acc:AT4G01703]	734252	734365	40.35
	AT4G01710	ARPC5A	Actin-related protein 2/3 complex subunit 5 [Source:UniProtKB/TrEMBL;Acc:A0A178UZC8]	735653	736965	38.39
	AT4G01720	WRKY47	Probable WRKY transcription factor 47 [Source:UniProtKB/Swiss-Prot;Acc:Q9ZSI7]	744804	748554	33.32
	AT4G01730	PAT18	Protein S-acyltransferase 18 [Source:UniProtKB/Swiss-Prot;Acc:Q9M115]	749307	752034	37.39
	AT4G01735		unknown protein; Ha. [Source:TAIR;Acc:AT4G01735]	752288	752873	40.96
	AT4G01740		Cysteine/Histidine-rich C1 domain family protein [Source:UniProtKB/TrEMBL;Acc:Q9ZSI9]	753422	755456	42.16

	AT4G01750	RGXT2	UDP-D-xylose:L-fucose alpha-1,3-D-xylosyltransferase [Source:UniProtKB/Swiss-Prot;Acc:Q9ZSJ0]	756348	758725	38.69
	AT4G01760		Cysteine/Histidine-rich C1 domain family protein [Source:UniProtKB/TrEMBL;Acc:Q9ZSJ1]	759055	761224	41.11
	AT4G01770	RGXT1	rhamnogalacturonan xylosyltransferase 1 [Source:TAIR;Acc:AT4G01770]	764473	766876	36.65
	AT4G01780		XH/XS domain-containing protein [Source:UniProtKB/TrEMBL;Acc:F4JG55]	767310	769246	40.22
	AT4G01790		At4g01790 [Source:UniProtKB/TrEMBL;Acc:Q9SYH9]	769799	770835	38.86
	AT4G01800	AGY1	Albino or Glassy Yellow 1 [Source:UniProtKB/TrEMBL;Acc:F4JG57]	770722	776439	39.44
	AT4G01810		Sec23/Sec24 protein transport family protein [Source:UniProtKB/TrEMBL;Acc:Q84WV4]	776414	780355	40.92
	AT4G01820	ABCB3	ABC transporter B family member 3 [Source:UniProtKB/Swiss-Prot;Acc:Q9SYI2]	780646	785340	40.51
	AT4G01830	ABCB5	ABC transporter B family member 5 [Source:UniProtKB/Swiss-Prot;Acc:Q9SYI3]	785551	790447	40.51
	AT4G01840	TPK5	Two-pore potassium channel 5 [Source:UniProtKB/Swiss-Prot;Acc:Q9S6Z8]	793177	795237	37.17
	AT4G01850	SAM2	S-adenosylmethionine synthase [Source:UniProtKB/TrEMBL;Acc:A0A178V3V9]	795986	798381	41.32
	AT4G01860		At4g01860 [Source:UniProtKB/TrEMBL;Acc:Q8RXC4]	801032	808087	38.8
	AT4G01865		pre-tRNA [Source:TAIR;Acc:AT4G01865]	808270	808342	58.9
	AT4G01870		tolB protein-related [Source:TAIR;Acc:AT4G01870]	808376	810611	46.69
	AT4G01880		Methyltransferase [Source:UniProtKB/TrEMBL;Acc:F4JG68]	810657	812930	38.04
	AT4G01883		Polyketide cyclase / dehydrase and lipid transport protein [Source:UniProtKB/TrEMBL;Acc:Q1G3L2]	813048	815170	35.47
	AT4G03995			815274	815466	46.11
	AT4G01890		Pectin lyase-like superfamily protein [Source:TAIR;Acc:AT4G01890]	816200	818468	37.02
	AT4G01895		At4g01895 [Source:UniProtKB/TrEMBL;Acc:Q9FNY8]	819957	820379	38.53
	AT4G01897		At4g01897 [Source:UniProtKB/TrEMBL;Acc:Q8LC12]	820336	821618	34.29
	AT4G04005			820726	821117	34.44
	AT4G01900	GLB1	Nitrogen regulatory protein P-II homolog [Source:UniProtKB/Swiss-Prot;Acc:Q9ZST4]	821546	823569	35.13
	AT4G01910		Cysteine/Histidine-rich C1 domain family protein [Source:UniProtKB/TrEMBL;Acc:Q9SYI8]	824555	826857	41.47
	AT4G01915		unknown protein; Ha. [Source:TAIR;Acc:AT4G01915]	827216	828824	38.47
	AT4G01920		AT4g01920/T7B11_18 [Source:UniProtKB/TrEMBL;Acc:Q9SYI9]	828826	831288	40.07
	AT4G01925		Cysteine/Histidine-rich C1 domain family protein [Source:UniProtKB/TrEMBL;Acc:F4JH56]	833063	834340	41.71
	AT4G04015			837666	838160	36.57
	AT4G01930		Cysteine/Histidine-rich C1 domain family protein [Source:UniProtKB/TrEMBL;Acc:Q9SYJ0]	838677	840825	41.14
	AT4G01935		unknown protein; FUNCTIONS IN: molecular_function unknown; INVOLVED IN: biological_process unknown; LOCATED IN: chlorop	841057	842364	39.45
	AT4G01940	NIFU1	NifU-like protein 1, chloroplastic [Source:UniProtKB/Swiss-Prot;Acc:Q93W77]	841989	843531	38.95
	AT4G04035			842657	843035	37.2
	AT4G01950	ATGPAT3	glycerol-3-phosphate acyltransferase 3 [Source:TAIR;Acc:AT4G01950]	844342	846814	39.18
	AT4G01960		At4g01960 [Source:UniProtKB/TrEMBL;Acc:Q9SYJ3]	850914	853329	35.64
	AT4G01970	AtSTS	stachyose synthase [Source:TAIR;Acc:AT4G01970]	853922	857401	40.83
	AT4G01985		unknown protein; FUNCTIONS IN: molecular_function unknown; INVOLVED IN: biological_process unknown; LOCATED IN: endom	866151	868253	57.11
	AT4G01990		Pentatricopeptide repeat-containing protein At4g01990, mitochondrial [Source:UniProtKB/Swiss-Prot;Acc:Q93WC5]	871126	872920	41.5
	AT4G01995		Beta-carotene isomerase D27 [Source:UniProtKB/TrEMBL;Acc:Q8W4C4]	872923	874772	35.62
	AT4G02000		Uncharacterized protein At4g02000 [Source:UniProtKB/Swiss-Prot;Acc:O04244]	874880	876181	40.09
	AT4G02005		other RNA [Source:TAIR;Acc:AT4G02005]	876876	879774	32.46
	AT4G02010		AT4g02010/T10M13_2 [Source:UniProtKB/TrEMBL;Acc:Q8L7V7]	881061	885463	39.47
	AT4G02020	EZA1	Histone-lysine N-methyltransferase EZA1 [Source:UniProtKB/Swiss-Prot;Acc:Q9ZSM8]	886580	891987	38.35
	AT4G02030		Vps51/Vps67 family (Components of vesicular transport) protein [Source:UniProtKB/TrEMBL;Acc:F4JH69]	892082	897359	38.01
	AT4G04055			897472	898253	42.33
	AT4G02040		At4g02040 [Source:UniProtKB/TrEMBL;Acc:O04248]	897547	898370	40.66
	AT4G02050	STP7	Sugar transport protein 7 [Source:UniProtKB/Swiss-Prot;Acc:O04249]	898307	901072	37.96
	AT4G02060	MCM7	DNA replication licensing factor MCM7 [Source:UniProtKB/Swiss-Prot;Acc:P43299]	900636	905591	38.46
MQTL-2/Chr4	AT4G01430		WAT1-related protein At4g01430 [Source:UniProtKB/Swiss-Prot;Acc:Q9M131]	585598	588126	32.46
	AT4G01435			591825	592592	36.85
	AT4G01440		WAT1-related protein At4g01440 [Source:UniProtKB/Swiss-Prot;Acc:Q9M130]	596397	598752	32.72
	AT4G01450		WAT1-related protein At4g01450 [Source:UniProtKB/Swiss-Prot;Acc:Q9M129]	608273	610765	32.53
	AT4G01460	BHLH57	Transcription factor bHLH57 [Source:UniProtKB/Swiss-Prot;Acc:Q9M128]	620691	623000	34.98
	AT4G01470	TIP1-3	Aquaporin TIP1-3 [Source:UniProtKB/Swiss-Prot;Acc:O82598]	624786	625885	43
	AT4G01480	PPA5	PPa5 [Source:UniProtKB/TrEMBL;Acc:A0A178V127]	626069	628070	34.77
	AT4G03875			630579	630795	49.77
	AT4G01500	NGA4	NGA4 [Source:UniProtKB/TrEMBL;Acc:A0A178V2Z9]	639247	640976	40.23
	AT4G01510	ARV2	Arv1-like protein [Source:TAIR;Acc:AT4G01510]	642714	644400	34.56
	AT4G01516		unknown protein; Ha. [Source:TAIR;Acc:AT4G01516]	649862	649957	54.17
	AT4G01520	anac067	F11O4.3 [Source:UniProtKB/TrEMBL;Acc:O82593]	656407	659178	33.77
	AT4G01533		other RNA [Source:TAIR;Acc:AT4G01533]	667148	669073	37.44
	AT4G01535		unknown protein; FUNCTIONS IN: molecular_function unknown; INVOLVED IN: biological_process unknown; LOCATED IN: endom	669339	669830	41.06
	AT4G01540	NAC68	NAC domain-containing protein 68 [Source:UniProtKB/Swiss-Prot;Acc:A8MQY1]	670483	672789	39.14
	AT4G01550	NAC69	NAC domain-containing protein 69 [Source:UniProtKB/Swiss-Prot;Acc:Q9M126]	673862	676445	37.77
	AT4G01560	MEE49	AT4g01560/F11O4_6 [Source:UniProtKB/TrEMBL;Acc:Q9M125]	676982	679276	35.95
	AT4G01570		Pentatricopeptide repeat-containing protein At4g01570 [Source:UniProtKB/Swiss-Prot;Acc:Q8VZE4]	679472	681940	42
	AT4G01575		Serine protease inhibitor, Kazal-type family protein [Source:UniProtKB/TrEMBL;Acc:F4JG23]	681862	682601	38.51
	AT4G03895			681862	682660	37.67
	AT4G01580		B3 domain-containing protein At4g01580 [Source:UniProtKB/Swiss-Prot;Acc:Q9ZSH7]	685670	686886	38.7
	AT4G03905			687493	687992	36.4
	AT4G03915			687715	687867	33.99
	AT4G03925			687898	688053	37.82
	AT4G01590		DNA-directed RNA polymerase III subunit [Source:UniProtKB/TrEMBL;Acc:Q9M123]	688813	690460	35.8
	AT4G01593		other RNA [Source:TAIR;Acc:AT4G01593]	690643	692176	36.57
	AT4G01595		Protein kinase superfamily protein [Source:UniProtKB/TrEMBL;Acc:F4JG28]	690726	691993	37.62
	AT4G01600		GEM-like protein 2 [Source:UniProtKB/Swiss-Prot;Acc:Q9M122]	692853	694381	37.28
	AT4G01610	CATHB3	Cathepsin B-like protease 3 [Source:UniProtKB/Swiss-Prot;Acc:Q94K85]	694582	697206	34.82
	AT4G01630	EXPA17	Expansin [Source:UniProtKB/TrEMBL;Acc:A0A178UWZ2]	700566	701769	40.03
	AT4G01640		F-box associated ubiquitination effector family protein [Source:UniProtKB/TrEMBL;Acc:Q9M121]	702563	703600	41.43
	AT4G01650		Polyketide cyclase / dehydrase and lipid transport protein [Source:UniProtKB/TrEMBL;Acc:Q9M120]	704377	707045	33.16
	AT4G01660	ABC1	Protein ABC transporter 1, mitochondrial [Source:UniProtKB/Swiss-Prot;Acc:Q9SBB2]	708357	711256	40.9
	AT4G01670		AT4G01670 protein [Source:UniProtKB/TrEMBL;Acc:Q8GXD3]	711192	712643	37.19
	AT4G03955			712342	712526	44.32
	AT4G01671		unknown protein; LOCATED IN: endomembrane system; Ha. [Source:TAIR;Acc:AT4G01671]	713216	713389	39.08
	AT4G01680	MYB55	Myb domain protein 55 [Source:UniProtKB/TrEMBL;Acc:Q9ZSI4]	715970	717603	35.43
	AT4G01690	PPOX1	Protoporphyrinogen oxidase [Source:UniProtKB/TrEMBL;Acc:A0A178UVN5]	729863	732433	41.73
	AT4G01700		At4g01700 [Source:UniProtKB/TrEMBL;Acc:Q9ZSI6]	732313	733510	41.24
	AT4G03985			733149	733419	50.55
	AT4G01703		unknown protein; Ha. [Source:TAIR;Acc:AT4G01703]	734252	734365	40.35
	AT4G01710	ARPC5A	Actin-related protein 2/3 complex subunit 5 [Source:UniProtKB/TrEMBL;Acc:A0A178UZC8]	735653	736965	38.39
	AT4G01720	WRKY47	Probable WRKY transcription factor 47 [Source:UniProtKB/Swiss-Prot;Acc:Q9ZSI7]	744804	748554	33.32
	AT4G01730	PAT18	Protein S-acyltransferase 18 [Source:UniProtKB/Swiss-Prot;Acc:Q9M115]	749307	752034	37.39
	AT4G01735		unknown protein; Ha. [Source:TAIR;Acc:AT4G01735]	752288	752873	40.96
	AT4G01740		Cysteine/Histidine-rich C1 domain family protein [Source:UniProtKB/TrEMBL;Acc:Q9ZSI9]	753422	755456	42.16
	AT4G01750	RGXT2	UDP-D-xylose:L-fucose alpha-1,3-D-xylosyltransferase [Source:UniProtKB/Swiss-Prot;Acc:Q9ZSJ0]	756348	758725	38.69
	AT4G01760		Cysteine/Histidine-rich C1 domain family protein [Source:UniProtKB/TrEMBL;Acc:Q9ZSJ1]	759055	761224	41.11
	AT4G01770	RGXT1	rhamnogalacturonan xylosyltransferase 1 [Source:TAIR;Acc:AT4G01770]	764473	766876	36.65
	AT4G01780		XH/XS domain-containing protein [Source:UniProtKB/TrEMBL;Acc:F4JG55]	767310	769246	40.22
	AT4G01790		At4g01790 [Source:UniProtKB/TrEMBL;Acc:Q9SYH9]	769799	770835	38.86
	AT4G01800	AGY1	Albino or Glassy Yellow 1 [Source:UniProtKB/TrEMBL;Acc:F4JG57]	770722	776439	39.44
	AT4G01810		Sec23/Sec24 protein transport family protein [Source:UniProtKB/TrEMBL;Acc:Q84WV4]	776414	780355	40.92
	AT4G01820	ABCB3	ABC transporter B family member 3 [Source:UniProtKB/Swiss-Prot;Acc:Q9SYI2]	780646	785340	40.51
	AT4G01830	ABCB5	ABC transporter B family member 5 [Source:UniProtKB/Swiss-Prot;Acc:Q9SYI3]	785551	790447	40.51
	AT4G01840	TPK5	Two-pore potassium channel 5 [Source:UniProtKB/Swiss-Prot;Acc:Q9S6Z8]	793177	795237	37.17
	AT4G01850	SAM2	S-adenosylmethionine synthase [Source:UniProtKB/TrEMBL;Acc:A0A178V3V9]	795986	798381	41.32
	AT4G01860		At4g01860 [Source:UniProtKB/TrEMBL;Acc:Q8RXC4]	801032	808087	38.8
	AT4G01865		pre-tRNA [Source:TAIR;Acc:AT4G01865]	808270	808342	58.9
	AT4G01870		tolB protein-related [Source:TAIR;Acc:AT4G01870]	808376	810611	46.69
	AT4G01880		Methyltransferase [Source:UniProtKB/TrEMBL;Acc:F4JG68]	810657	812930	38.04
	AT4G01883		Polyketide cyclase / dehydrase and lipid transport protein [Source:UniProtKB/TrEMBL;Acc:Q1G3L2]	813048	815170	35.47
	AT4G03995			815274	815466	46.11

AT4G01890		Pectin lyase-like superfamily protein [Source:TAIR;Acc:AT4G01890]	816200	818468	37.02
AT4G01895		At4g01895 [Source:UniProtKB/TrEMBL;Acc:Q9FNY8]	819957	820379	38.53
AT4G01897		At4g01897 [Source:UniProtKB/TrEMBL;Acc:Q8LC12]	820336	821618	34.29
AT4G04005			820726	821117	34.44
AT4G01900	GLB1	Nitrogen regulatory protein P-II homolog [Source:UniProtKB/Swiss-Prot;Acc:Q9ZST4]	821546	823569	35.13
AT4G01910		Cysteine/Histidine-rich C1 domain family protein [Source:UniProtKB/TrEMBL;Acc:Q9SYI8]	824555	826857	41.47
AT4G01915		unknown protein; Ha. [Source:TAIR;Acc:AT4G01915]	827216	828824	38.47
AT4G01920		AT4g01920/T7B11_18 [Source:UniProtKB/TrEMBL;Acc:Q9SYI9]	828826	831288	40.07
AT4G01925		Cysteine/Histidine-rich C1 domain family protein [Source:UniProtKB/TrEMBL;Acc:F4JH56]	833063	834340	41.71
AT4G04015			837666	838160	36.57
AT4G01930		Cysteine/Histidine-rich C1 domain family protein [Source:UniProtKB/TrEMBL;Acc:Q9SYJ0]	838677	840825	41.14
AT4G01935		unknown protein; FUNCTIONS IN: molecular_function unknown; INVOLVED IN: biological_process unknown; LOCATED IN: chlorop	841057	842364	39.45
AT4G01940	NIFU1	NifU-like protein 1, chloroplastic [Source:UniProtKB/Swiss-Prot;Acc:Q93W77]	841989	843531	38.95
AT4G04035			842657	843035	37.2
AT4G01950	ATGPAT3	glycerol-3-phosphate acyltransferase 3 [Source:TAIR;Acc:AT4G01950]	844342	846814	39.18
AT4G01960		At4g01960 [Source:UniProtKB/TrEMBL;Acc:Q9SYJ3]	850914	853329	35.64
AT4G01970	AtSTS	stachyose synthase [Source:TAIR;Acc:AT4G01970]	853922	857401	40.83
AT4G01985		unknown protein; FUNCTIONS IN: molecular_function unknown; INVOLVED IN: biological_process unknown; LOCATED IN: endom	866151	868253	57.11
AT4G01990		Pentatricopeptide repeat-containing protein At4g01990, mitochondrial [Source:UniProtKB/Swiss-Prot;Acc:Q93WC5]	871126	872920	41.5
AT4G01995		Beta-carotene isomerase D27 [Source:UniProtKB/Swiss-Prot;Acc:Q8W4C4]	872923	874772	35.62
AT4G02000		Uncharacterized protein At4g02000 [Source:UniProtKB/Swiss-Prot;Acc:O04244]	874880	876181	40.09
AT4G02005		other RNA [Source:TAIR;Acc:AT4G02005]	876876	879774	32.46
AT4G02010		AT4g02010/T10M13_2 [Source:UniProtKB/TrEMBL;Acc:Q8L7V7]	881061	885463	39.47
AT4G02020	EZA1	Histone-lysine N-methyltransferase EZA1 [Source:UniProtKB/Swiss-Prot;Acc:Q9ZSM8]	886580	891987	38.35
AT4G02030		Vps51/Vps67 family (Components of vesicular transport) protein [Source:UniProtKB/TrEMBL;Acc:F4JH69]	892082	897359	38.01
AT4G04055			897472	898253	42.33
AT4G02040		At4g02040 [Source:UniProtKB/TrEMBL;Acc:O04248]	897547	898370	40.66
AT4G02050	STP7	Sugar transport protein 7 [Source:UniProtKB/Swiss-Prot;Acc:O04249]	898307	901072	37.96
AT4G02060	MCM7	DNA replication licensing factor MCM7 [Source:UniProtKB/Swiss-Prot;Acc:P43299]	900636	905591	38.46
AT4G02055		pre-tRNA [Source:TAIR;Acc:AT4G02055]	901176	901247	61.11
AT4G02070	MSH6	DNA mismatch repair protein MSH6 [Source:UniProtKB/Swiss-Prot;Acc:O04716]	905865	913290	39.56
AT4G02075	PIT1	At4g02075 [Source:UniProtKB/TrEMBL;Acc:Q9XF50]	913334	916509	29.72
AT4G02080	SAR1A	SAR2 [Source:UniProtKB/TrEMBL;Acc:A0A178V5S8]	921447	922759	39.53
AT4G02090		AGAA.5 [Source:UniProtKB/TrEMBL;Acc:O04712]	923122	923893	46.11
AT4G02100		At4g02100 [Source:UniProtKB/TrEMBL;Acc:Q84VX5]	929869	932397	42.55
AT4G02110		BRCT domain-containing protein At4g02110 [Source:UniProtKB/Swiss-Prot;Acc:O04251]	935006	940328	41.76
AT4G02120		CTP synthase family protein [Source:TAIR;Acc:AT4G02120]	940721	944327	39.4
AT4G02130	GATL6	Probable galacturonosyltransferase-like 6 [Source:UniProtKB/Swiss-Prot;Acc:O04253]	944324	947249	38.38
AT4G02140		At4g02140 [Source:UniProtKB/TrEMBL;Acc:O04254]	948598	951063	37.15
AT4G04065			948994	949369	46.01
AT4G02150	IMPA3	Importin subunit alpha-3 [Source:UniProtKB/Swiss-Prot;Acc:O04294]	950546	953690	39.05
AT4G02160		Cotton fiber protein [Source:UniProtKB/TrEMBL;Acc:Q9ZU01]	954968	955734	40.55
AT4G02170		unknown protein; BEST Arabidopsis thaliana protein match is: unknown protein (TAIR:AT5G38700.1); Ha. [Source:TAIR;Acc:AT4G021	958045	958834	36.96
AT4G02180		DC1 domain-containing protein [Source:UniProtKB/TrEMBL;Acc:Q9ZU00]	959964	963678	39.81
AT4G02190		Cysteine/Histidine-rich C1 domain family protein [Source:UniProtKB/TrEMBL;Acc:O04257]	967372	969351	41.97
AT4G02195	SYP42	Syntaxin-42 [Source:UniProtKB/Swiss-Prot;Acc:Q9SWH4]	969994	972342	37.38
AT4G02200		Drought-responsive family protein [Source:UniProtKB/TrEMBL;Acc:F4JH94]	972707	974692	36.46
AT4G04075			973174	973552	36.41
AT4G02210		Myb/SANT-like DNA-binding domain protein [Source:UniProtKB/TrEMBL;Acc:O81424]	973924	976154	38.77
AT4G02220		Putative zinc finger protein [Source:UniProtKB/TrEMBL;Acc:Q93VB4]	976246	979163	38.21
AT4G02230	RPL19C	60S ribosomal protein L19-3 [Source:UniProtKB/Swiss-Prot;Acc:P49693]	979158	980698	40.23
AT4G02235	AGL51	AGL51 [Source:UniProtKB/TrEMBL;Acc:A0A178UXV6]	980955	981711	40.95
AT4G02250		Plant invertase/pectin methylesterase inhibitor superfamily protein [Source:UniProtKB/TrEMBL;Acc:F4JHA1]	983970	984523	49.64
AT4G02260	RSH1	RSH1 [Source:UniProtKB/TrEMBL;Acc:A0A178V5K1]	985123	991506	38.49
AT4G02270	RHS13	At4g02270 [Source:UniProtKB/TrEMBL;Acc:O81417]	992111	993039	38
AT4G02280	SUS3	Sucrose synthase (Fragment) [Source:UniProtKB/TrEMBL;Acc:W8Q6L8]	994726	998991	39.1
AT4G02290	AtGH9B13	Endoglucanase 17 [Source:UniProtKB/Swiss-Prot;Acc:O81416]	1002338	1005305	36.59
AT4G04085			1008263	1008833	36.95
AT4G02300	PME39	Probable pectinesterase/pectinesterase inhibitor 39 [Source:UniProtKB/Swiss-Prot;Acc:O81415]	1009301	1013036	31.05
AT4G02310		Putative F-box/kelch-repeat protein At4g02310 [Source:UniProtKB/Swiss-Prot;Acc:O81414]	1014680	1015729	43.24
AT4G02320	PME40	Pectinesterase [Source:UniProtKB/TrEMBL;Acc:A0A178V0A5]	1022725	1026179	35.17
AT4G04095			1023912	1024149	39.08
AT4G04115			1030935	1031328	36.04
AT4G02330	PME41	Probable pectinesterase/pectinesterase inhibitor 41 [Source:UniProtKB/Swiss-Prot;Acc:Q8RXK7]	1032350	1035055	36.92
AT4G02340		AT4g02340 protein [Source:UniProtKB/TrEMBL;Acc:O81299]	1035596	1037615	39.95
AT4G02350	SEC15B	Exocyst complex component SEC15B [Source:UniProtKB/Swiss-Prot;Acc:F4JHH5]	1038114	1041064	42.93
AT4G02360		T14P8.17 [Source:UniProtKB/TrEMBL;Acc:O81297]	1041086	1041802	40.86
AT4G02370		AT4g02370 protein [Source:UniProtKB/TrEMBL;Acc:O81296]	1042235	1043283	39.37
AT4G02380	SAG21	senescence-associated gene 21 [Source:TAIR;Acc:AT4G02380]	1046042	1047105	39.38
AT4G04125			1046804	1046983	41.11
AT4G02390	PARP2	Poly [ADP-ribose] polymerase 2 [Source:UniProtKB/Swiss-Prot;Acc:Q11207]	1049766	1054186	37.1
AT4G02400		U3 ribonucleoprotein (Utp) family protein [Source:UniProtKB/TrEMBL;Acc:F4JHI1]	1054199	1057998	39.42
AT4G02405		S-adenosyl-L-methionine-dependent methyltransferases superfamily protein [Source:UniProtKB/TrEMBL;Acc:B3H6G9]	1057854	1059671	38.94
AT4G02410	LECRK43	L-type lectin-domain containing receptor kinase IV.3 [Source:UniProtKB/Swiss-Prot;Acc:O81292]	1059799	1062159	42.99
AT4G02420	LECRK44	L-type lectin-domain containing receptor kinase IV.4 [Source:UniProtKB/Swiss-Prot;Acc:O81291]	1064114	1066571	44.22
AT4G02425		LYR motif protein [Source:UniProtKB/TrEMBL;Acc:Q8GYI5]	1067052	1068821	40.79
AT4G02430	SR34b	RNA-binding (RRM/RBD/RNP motifs) family protein [Source:TAIR;Acc:AT4G02430]	1068974	1072263	38.63
AT4G02440	EID1	Phytochrome A-associated F-box protein [Source:UniProtKB/Swiss-Prot;Acc:Q8LEA8]	1072376	1073779	44.59
AT4G02450	P23-1	Co-chaperone protein p23-1 [Source:UniProtKB/Swiss-Prot;Acc:Q8L7U4]	1073753	1075930	39.07
AT4G04135			1074079	1074344	34.21
AT4G02460	PMS1	DNA mismatch repair protein PMS1 [Source:UniProtKB/Swiss-Prot;Acc:Q941I6]	1076090	1080727	39.48
AT4G02465		unknown protein; Ha. [Source:TAIR;Acc:AT4G02465]	1080962	1081680	39.78
AT4G02480		AAA-type ATPase family protein [Source:UniProtKB/TrEMBL;Acc:Q0WM93]	1081671	1088846	39.67
AT4G02482		chloroplast outer envelope GTP-binding protein, putative [Source:TAIR;Acc:AT4G02482]	1092012	1092610	42.57
AT4G02485		2-oxoglutarate (2OG) and Fe(II)-dependent oxygenase superfamily protein [Source:UniProtKB/TrEMBL;Acc:Q1ECQ5]	1092522	1094600	36.84
AT4G02489		unknown protein; LOCATED IN: endomembrane system; Ha. [Source:TAIR;Acc:AT4G02489]	1097532	1097645	50.88
AT4G02500	XXT2	Xyloglucan 6-xylosyltransferase 2 [Source:UniProtKB/Swiss-Prot;Acc:O22775]	1101378	1103612	40.94
AT4G02510	TOC159	Translocase of chloroplast 159, chloroplastic [Source:UniProtKB/Swiss-Prot;Acc:O81283]	1104429	1111784	43.42
AT4G02520	GSTF2	Glutathione S-transferase F2 [Source:UniProtKB/Swiss-Prot;Acc:P46422]	1110343	1112033	39.56
AT4G02530		chloroplast thylakoid lumen protein [Source:TAIR;Acc:AT4G02530]	1112082	1114158	36.74
AT4G04185			1114529	1114629	48.51
AT4G04195			1114529	1114766	42.44
AT4G02540		Cysteine/Histidine-rich C1 domain family protein [Source:UniProtKB/TrEMBL;Acc:Q9ZQX3]	1116972	1119877	41.78
AT4G02541		unknown protein; Ha. [Source:TAIR;Acc:AT4G02541]	1119849	1119989	39.72
AT4G02550		unknown protein; BEST Arabidopsis thaliana protein match is: unknown protein (TAIR:AT4G02210.2); Ha. [Source:TAIR;Acc:AT4G025	1120365	1122223	38.57
AT4G02555		snoRNA [Source:TAIR;Acc:AT4G02555]	1122603	1122678	32.89
AT4G04205			1122744	1122852	32.11
AT4G02560	LD	Homeobox protein LUMINIDEPENDENS [Source:UniProtKB/Swiss-Prot;Acc:Q38796]	1123475	1128490	39.51
AT4G02570	CUL1	AT4G02570 protein [Source:UniProtKB/TrEMBL;Acc:B9DGE3]	1128515	1133774	37.24
AT4G02580		NADH dehydrogenase [ubiquinone] flavoprotein 2, mitochondrial [Source:UniProtKB/Swiss-Prot;Acc:O22769]	1134429	1137204	38.18
AT4G02590	UNE12	UNE12 [Source:UniProtKB/TrEMBL;Acc:A0A384KS03]	1137487	1140540	37.69
AT4G04215			1141044	1141387	38.08
AT4G02600	MLO1	MLO-like protein 1 [Source:UniProtKB/Swiss-Prot;Acc:O49621]	1143888	1147562	36.35
AT4G02610	TRPA1	Tryptophan synthase alpha chain [Source:UniProtKB/Swiss-Prot;Acc:O22765]	1147608	1149336	38.98
AT4G02620	VHA-F	V-type proton ATPase subunit F [Source:UniProtKB/TrEMBL;Acc:A0A178V4M8]	1149248	1151355	35.25
AT4G04225			1149555	1149979	35.76
AT4G02630		Protein kinase superfamily protein [Source:UniProtKB/TrEMBL;Acc:O22764]	1151611	1153409	41.47
AT4G02640	BZO2H1	bZIP transcription factor family protein [Source:TAIR;Acc:AT4G02640]	1153945	1156356	39.3
AT4G02650		Putative clathrin assembly protein At4g02650 [Source:UniProtKB/Swiss-Prot;Acc:Q8GX47]	1156379	1158788	40.95

AT4G02655		unknown protein; FUNCTIONS IN: molecular_function unknown; INVOLVED IN: biological_process unknown; LOCATED IN: endom	1158783	1159422	38.59
AT4G02660		Beige/BEACH domain ;WD domain, G-beta repeat protein [Source:TAIR;Acc:AT4G02660]	1159494	1174321	40.35
AT4G02670	AtIDD12	IDD12 [Source:UniProtKB/TrEMBL;Acc:A0A178V4W8]	1176000	1178624	36.08
AT4G04235			1178514	1178768	30.59
AT4G02680	EOL1	EOL1 [Source:UniProtKB/TrEMBL;Acc:A0A178V4A7]	1180923	1185012	40.64
AT4G02690	LFG3	Protein LIFEGUARD 3 [Source:UniProtKB/Swiss-Prot;Acc:Q9ZQX7]	1185751	1187816	39.06
AT4G02700	SULTR3;2	Sulfate transporter 3.2 [Source:UniProtKB/Swiss-Prot;Acc:O04289]	1188983	1193611	34.93
AT4G02710	NET1C	Protein NETWORKED 1C [Source:UniProtKB/Swiss-Prot;Acc:Q9ZQX8]	1193516	1197630	40.46
AT4G04275			1195269	1195677	43.03
AT4G02715		At4g02715 [Source:UniProtKB/TrEMBL;Acc:Q8L757]	1200670	1203380	39.32
AT4G04285			1200791	1201034	35.25
AT4G02725		At4g02725 [Source:UniProtKB/TrEMBL;Acc:Q6DBF6]	1204013	1207436	39.78
AT4G02720		AT4g02720/T10P11_1 [Source:UniProtKB/TrEMBL;Acc:Q8S9I4]	1204089	1205892	43.07
AT4G02730	WDR5B	COMPASS-like H3K4 histone methylase component WDR5B [Source:UniProtKB/Swiss-Prot;Acc:Q9SY00]	1207683	1209287	39.5
AT4G04295			1209575	1209854	41.43
AT4G02733		F-box protein At4g02733 [Source:UniProtKB/Swiss-Prot;Acc:Q2V3L6]	1210140	1211532	37.26
AT4G02735		F-box SKIP17-like protein [Source:UniProtKB/TrEMBL;Acc:A0A1P8B5P2]	1215092	1217509	36.56
AT4G02740		F-box/RNI-like superfamily protein [Source:TAIR;Acc:AT4G02740]	1217589	1221019	37.77
AT4G02750	PCMP-H24	Pentatricopeptide repeat-containing protein At4g02750 [Source:UniProtKB/Swiss-Prot;Acc:Q9SY02]	1221017	1223617	43.06
AT4G02760		RNI-like superfamily protein [Source:UniProtKB/TrEMBL;Acc:F4JHU9]	1223959	1229014	38.01
AT4G02770	PSAD1	Photosystem I reaction center subunit II-1, chloroplastic [Source:UniProtKB/Swiss-Prot;Acc:Q9S7H1]	1229031	1230164	41.27
AT4G02780	GA1	Ent-copalyl diphosphate synthase, chloroplastic [Source:UniProtKB/Swiss-Prot;Acc:Q38802]	1237671	1244822	32.13
AT4G02790	DGP3	DAR GTPase 3, chloroplastic [Source:UniProtKB/Swiss-Prot;Acc:Q8H1F6]	1247327	1249640	37.6
AT4G02800		At4g02800 [Source:UniProtKB/TrEMBL;Acc:Q9SY05]	1249971	1251867	35.9
AT4G02810	FAF1	Protein FANTASTIC FOUR 1 [Source:UniProtKB/Swiss-Prot;Acc:Q9SY06]	1256144	1257320	39.42
AT4G02820		Pentatricopeptide repeat-containing protein At4g02820, mitochondrial [Source:UniProtKB/Swiss-Prot;Acc:Q9SY07]	1258532	1260696	40.6
AT4G02830		Uncharacterized protein AT4g02830 [Source:UniProtKB/TrEMBL;Acc:Q9SY08]	1262245	1264101	31.18
AT4G02840			1264518	1266486	37.18
AT4G02850		At4g02850 [Source:UniProtKB/TrEMBL;Acc:Q66GM9]	1266463	1268612	37.49
AT4G02860		Phenazine biosynthesis PhzC/PhzF protein [Source:UniProtKB/TrEMBL;Acc:F4JHW1]	1268618	1270539	38.55
AT4G02870	ARF42	B3 domain-containing protein At4g02870 [Source:UniProtKB/Swiss-Prot;Acc:Q9SY12]	1271654	1272746	40.71
AT4G04305			1274064	1274302	40.59
AT4G02880		unknown protein; BEST Arabidopsis thaliana protein match is: unknown protein (TAIR:AT1G03290.2); Ha. [Source:TAIR;Acc:AT4G028	1274465	1278718	37.82
AT4G04325			1276901	1277094	34.54
AT4G02890	UBQ14	Polyubiquitin 14 [Source:UniProtKB/Swiss-Prot;Acc:Q3E7T8]	1278530	1280166	43.07
AT4G04335			1279643	1279985	34.69
AT4G02900		Hyperosmolality-gated Ca2+ permeable channel 1.7 [Source:UniProtKB/TrEMBL;Acc:A0A097NUP8]	1283655	1287832	38.18
AT4G04345			1283762	1284017	39.06
AT4G02910		Uncharacterized protein AT4g02910 [Source:UniProtKB/TrEMBL;Acc:Q9ZT89]	1288486	1289615	31.68
AT4G02920		Uncharacterized protein At4g02920 [Source:UniProtKB/TrEMBL;Acc:Q8RXG5]	1292483	1295263	38.87
AT4G02930	TUFA	Elongation factor Tu, mitochondrial [Source:UniProtKB/Swiss-Prot;Acc:Q9ZT91]	1295375	1298500	39.28
AT4G04355			1299566	1299842	37.91
AT4G02940	ALKBH10B	RNA demethylase ALKBH10B [Source:UniProtKB/Swiss-Prot;Acc:Q9ZT92]	1306313	1311038	37.16
AT4G02950		Putative ubiquitin-like protein [Source:UniProtKB/TrEMBL;Acc:Q9ZT93]	1311198	1312154	41.8
AT4G02970	AT7SL-1		1317499	1319179	41.4
AT4G02980	ERABP1	Auxin-binding protein 1 [Source:UniProtKB/Swiss-Prot;Acc:P33487]	1319603	1321608	37.44
AT4G02990	MTERF4	Transcription termination factor MTERF4, chloroplastic [Source:UniProtKB/Swiss-Prot;Acc:Q9ZT96]	1321758	1324062	41.3
AT4G03000	RF298	Putative E3 ubiquitin-protein ligase RF298 [Source:UniProtKB/Swiss-Prot;Acc:Q0WPJ7]	1324063	1327520	40.8
AT4G03010		Leucine-rich repeat family protein [Source:UniProtKB/TrEMBL;Acc:Q9ZT98]	1329548	1331387	42.61
AT4G03020		Putative WD-repeat protein [Source:UniProtKB/TrEMBL;Acc:Q8LPI5]	1331399	1335463	37.22
AT4G03030	OR23	F-box/kelch-repeat protein OR23 [Source:UniProtKB/Swiss-Prot;Acc:Q0V7S6]	1335680	1337565	42.84
AT4G03038		other RNA [Source:TAIR;Acc:AT4G03038]	1340115	1341289	32.94
AT4G03039	MIR826A	MIR826a; miRNA [Source:TAIR;Acc:AT4G03039]	1340479	1340656	38.76
AT4G04365			1340521	1340614	32.98
AT4G03040		Uncharacterized protein AT4g03040 [Source:UniProtKB/TrEMBL;Acc:Q9ZTA0]	1341083	1343140	31.15
AT4G03050	AOP3	2-oxoglutarate-dependent dioxygenase AOP3 [Source:UniProtKB/Swiss-Prot;Acc:Q9ZTA1]	1343845	1346436	35.26
AT4G04385			1348264	1348470	36.23
AT4G03070	AOP1	Probable 2-oxoglutarate-dependent dioxygenase AOP1 [Source:UniProtKB/Swiss-Prot;Acc:Q9ZTA3]	1358267	1359698	35.82
AT4G03080	BSL1	Serine/threonine-protein phosphatase BSL1 [Source:UniProtKB/Swiss-Prot;Acc:Q8L7U5]	1359655	1365498	40.26
AT4G04425			1360000	1360276	36.1
AT4G03090	NDX	Nodulin homeobox [Source:UniProtKB/Swiss-Prot;Acc:F4JI44]	1366053	1371557	37.8
AT4G03100	ROPGAP2	Rho GTPase-activating protein 2 [Source:UniProtKB/Swiss-Prot;Acc:F4JI46]	1374160	1376287	40.13
AT4G03110	BRN1	RNA-binding protein BRN1 [Source:UniProtKB/Swiss-Prot;Acc:Q8LFS6]	1376306	1379747	36.72
AT4G03115		Mitochondrial substrate carrier family protein [Source:TAIR;Acc:AT4G03115]	1382438	1385525	36.85
AT4G03113		unknown protein; LOCATED IN: mitochondrion; Ha. [Source:TAIR;Acc:AT4G03113]	1382553	1382741	40.74
AT4G03120		U1 small nuclear ribonucleoprotein C [Source:UniProtKB/Swiss-Prot;Acc:Q56XE4]	1385613	1387803	37.84
AT4G03130		BRCT domain-containing DNA repair protein [Source:TAIR;Acc:AT4G03130]	1387781	1390781	40.79
AT4G03135		pre-tRNA [Source:TAIR;Acc:AT4G03135]	1390895	1390966	54.17
AT4G03140		NAD(P)-binding Rossmann-fold superfamily protein [Source:UniProtKB/TrEMBL;Acc:F4JI53]	1392121	1393842	40.19
AT4G04435			1392584	1393519	42.31
AT4G03150		AT4g03150/F4C21_7 [Source:UniProtKB/TrEMBL;Acc:Q944G6]	1393614	1394583	40.52
AT4G03153	NET3B	NET3B [Source:UniProtKB/TrEMBL;Acc:A0A178V312]	1394845	1395588	37.77
AT4G03156		Small GTPase-like protein [Source:UniProtKB/TrEMBL;Acc:Q3EAC2]	1396002	1396784	36.53
AT4G03157		Uncharacterized protein (Fragment) [Source:UniProtKB/TrEMBL;Acc:A0A1P8B6S2]	1396953	1397351	35.34
AT4G03160		Putative B3 domain-containing protein At4g03160 [Source:UniProtKB/Swiss-Prot;Acc:Q9ZR15]	1397979	1398685	42.43
AT4G03165		unknown protein; Ha. [Source:TAIR;Acc:AT4G03165]	1399378	1400291	37.31
AT4G03170		Putative B3 domain-containing protein At4g03170 [Source:UniProtKB/Swiss-Prot;Acc:Q9ZR14]	1400832	1401584	39.31
AT4G03175		Protein kinase superfamily protein [Source:TAIR;Acc:AT4G03175]	1402088	1402869	37.08
AT4G03180		At4g03180 [Source:UniProtKB/TrEMBL;Acc:Q9ZR13]	1403117	1404937	37.89
AT4G04445			1403428	1404292	35.49
AT4G03190	GRH1	GRR1-like protein 1 [Source:UniProtKB/Swiss-Prot;Acc:Q9ZR12]	1404887	1407402	42.13
AT4G04455			1405104	1405526	44.92
AT4G03200		catalytics [Source:TAIR;Acc:AT4G03200]	1408159	1412756	39.19
AT4G03205	hemf2	Coproporphyrinogen III oxidase [Source:TAIR;Acc:AT4G03205]	1412785	1414902	40.18
AT4G03210	XTH9	Xyloglucan endotransglucosylase/hydrolase (Fragment) [Source:UniProtKB/TrEMBL;Acc:C0SVH2]	1415617	1417444	38.02
AT4G03220		Putative F-box/FBD/LRR-repeat protein At4g03220 [Source:UniProtKB/Swiss-Prot;Acc:Q9ZR09]	1417472	1419148	38.76
AT4G03230		S-locus lectin protein kinase family protein [Source:TAIR;Acc:AT4G03230]	1418841	1423337	40
AT4G03240	FH	FH [Source:UniProtKB/TrEMBL;Acc:A0A178V3B4]	1423238	1424772	37.46
AT4G03250		Homeodomain-like superfamily protein [Source:UniProtKB/TrEMBL;Acc:F4JI72]	1424902	1428062	38.03
AT4G03260		At4g03260 [Source:UniProtKB/TrEMBL;Acc:Q8GUJ5]	1428102	1432690	40.27
AT4G03270	CYCD6-1	Putative cyclin-D6-1 [Source:UniProtKB/Swiss-Prot;Acc:Q9ZR04]	1431844	1433861	37.07
AT4G03280	PETC	Cytochrome b6-f complex iron-sulfur subunit, chloroplastic [Source:UniProtKB/Swiss-Prot;Acc:Q9ZR03]	1440146	1441863	41.27
AT4G03285		pre-tRNA [Source:TAIR;Acc:AT4G03285]	1442005	1442076	54.17
AT4G03290	CML6	Calmodulin-like protein 6 [Source:UniProtKB/Swiss-Prot;Acc:Q9ZR02]	1442546	1443638	36.32
AT4G03292		Polynucleotidyl transferase, ribonuclease H-like superfamily protein [Source:UniProtKB/TrEMBL;Acc:Q3E7R1]	1444469	1445220	41.89
AT4G04465			1445731	1445846	42.24
AT4G03295		snoRNA [Source:TAIR;Acc:AT4G03295]	1445879	1445954	38.16
AT4G04475			1445997	1446085	33.71
AT4G04485			1446117	1446259	43.36
AT4G03298		unknown protein; FUNCTIONS IN: molecular_function unknown; INVOLVED IN: biological_process unknown; LOCATED IN: cellular	1446664	1447718	34.12
AT4G03320	TIC20-IV	Protein TIC 20-IV, chloroplastic [Source:UniProtKB/Swiss-Prot;Acc:Q9ZQZ9]	1464467	1466174	40.81
AT4G03330	SYP123	At4g03330 [Source:UniProtKB/TrEMBL;Acc:Q0WPM4]	1466284	1467750	40.63
AT4G04505			1466474	1466768	41.69
AT4G03340		Core-2/I-branching beta-1,6-N-acetylglucosaminyltransferase family protein [Source:UniProtKB/TrEMBL;Acc:Q9ZQZ7]	1467727	1470080	39.55
AT4G04515			1469052	1469672	36.71
AT4G04535			1470463	1471229	40.42
AT4G04565			1472018	1473136	39.77
AT4G04585			1473079	1473602	35.31
AT4G03350	7SL2	Ubiquitin domain-containing protein 7SL RNA2 [Source:UniProtKB/Swiss-Prot;Acc:Q9ZQZ6]	1473718	1474509	43.31

AT4G03360		Ubiquitin family protein [Source:TAIR;Acc:AT4G03360]	1474869	1475968	40.73
AT4G03370		Putative ubiquitin-like protein [Source:UniProtKB/TrEMBL;Acc:Q9ZQZ4]	1478078	1478965	44.26
AT4G04615			1483021	1483121	45.54
AT4G03380		Uncharacterized protein AT4g03380 [Source:UniProtKB/TrEMBL;Acc:Q9ZQZ3]	1485049	1490228	34.44
AT4G03390	SRF3	Protein STRUBBELIG-RECEPTOR FAMILY 3 [Source:UniProtKB/Swiss-Prot;Acc:Q6R2K3]	1490426	1495214	38.9
AT4G04625			1495561	1495881	34.27
AT4G03400	DFL2	Indole-3-acetic acid-amido synthetase GH3.10 [Source:UniProtKB/Swiss-Prot;Acc:Q9ZNS2]	1497536	1501599	40.7
AT4G03410		Peroxisomal membrane 22 kDa (Mpv17/PMP22) family protein [Source:UniProtKB/TrEMBL;Acc:F4JI90]	1501168	1503699	39.38
AT4G03405		pre-tRNA [Source:TAIR;Acc:AT4G03405]	1501607	1501678	50
AT4G03415		Probable protein phosphatase 2C 52 [Source:UniProtKB/Swiss-Prot;Acc:Q8GY60]	1503492	1506535	39.85
AT4G03420		AT4g03420/F9H3_4 [Source:UniProtKB/TrEMBL;Acc:Q8S9K9]	1511597	1514072	37.2
AT4G03430	STA1	Protein STABILIZED1 [Source:UniProtKB/Swiss-Prot;Acc:Q9ZT71]	1516030	1520563	44.66
AT4G03435		pre-tRNA [Source:TAIR;Acc:AT4G03435]	1520784	1520856	58.9
AT4G03438	MIR447C	MIR447C; miRNA [Source:TAIR;Acc:AT4G03438]	1523306	1523503	34.85
AT4G03440		Ankyrin repeat family protein [Source:UniProtKB/TrEMBL;Acc:Q9ZT72]	1523947	1527628	39.49
AT4G03443		unknown protein; FUNCTIONS IN: molecular_function unknown; INVOLVED IN: biological_process unknown; LOCATED IN: cellular	1527716	1528057	45.03
AT4G03445	MIR447A	MIR447A; miRNA [Source:TAIR;Acc:AT4G03445]	1528134	1528370	36.29
AT4G03450		Ankyrin repeat family protein [Source:UniProtKB/TrEMBL;Acc:Q9ZT73]	1529372	1531819	39.91
AT4G04645			1533414	1533620	43
AT4G03455	MIR447B	MIR447B; miRNA [Source:TAIR;Acc:AT4G03455]	1535426	1535661	37.29
AT4G03460		Ankyrin repeat family protein [Source:TAIR;Acc:AT4G03460]	1537118	1540713	39.21
AT4G03470		Ankyrin repeat family protein [Source:UniProtKB/TrEMBL;Acc:Q9M109]	1542018	1544457	40.29
AT4G03480		Ankyrin repeat family protein [Source:UniProtKB/TrEMBL;Acc:F4JG86]	1545470	1548871	40.21
AT4G03490		Ankyrin repeat family protein [Source:TAIR;Acc:AT4G03490]	1549176	1554159	39.69
AT4G03495			1549515	1550451	41.3
AT4G03500		Ankyrin repeat family protein [Source:UniProtKB/TrEMBL;Acc:Q9ZT78]	1553092	1556704	38.31
AT4G03505		Putative uncharacterized protein [Source:UniProtKB/TrEMBL;Acc:Q2V3L5]	1556888	1557319	44.68
AT4G03510	RMA1	E3 ubiquitin-protein ligase RMA1 [Source:UniProtKB/Swiss-Prot;Acc:O64425]	1557120	1559437	38.74
AT4G03520	ATHM2	Thioredoxin M2, chloroplastic [Source:UniProtKB/Swiss-Prot;Acc:Q9SEU8]	1562203	1564204	38.21
AT4G03540		CASP-like protein [Source:UniProtKB/TrEMBL;Acc:A0A178V2N5]	1569937	1571737	33.54
AT4G03550	CALS12	Callose synthase 12 [Source:UniProtKB/Swiss-Prot;Acc:Q9ZT82]	1573266	1579820	44.06
AT4G03560	TPC1	AT4G03560 protein [Source:UniProtKB/TrEMBL;Acc:B9DFD5]	1579929	1585980	37.08
AT4G03565		Cystatin/monellin superfamily protein [Source:UniProtKB/TrEMBL;Acc:F4JG98]	1586747	1587734	39.57
AT4G03566		Ribonuclease H [Source:UniProtKB/TrEMBL;Acc:Q1G3K1]	1588564	1589230	42.58
AT4G03570		Cystatin/monellin superfamily protein [Source:UniProtKB/TrEMBL;Acc:Q9SY35]	1590413	1594105	37.8
AT4G03580		Uncharacterized protein AT4g03580 [Source:UniProtKB/TrEMBL;Acc:Q9SY36]	1596985	1599091	33.51
AT4G03590		Cystatin/monellin superfamily protein [Source:UniProtKB/TrEMBL;Acc:Q9SY37]	1600780	1602950	35.24
AT4G03600		Pyrroline-5-carboxylate reductase [Source:UniProtKB/TrEMBL;Acc:Q9SY38]	1603638	1604628	36.93
AT4G03610		Metallo-hydrolase/oxidoreductase superfamily protein [Source:TAIR;Acc:AT4G03610]	1604886	1607496	37.11
AT4G03620		Myosin heavy chain-like protein [Source:UniProtKB/TrEMBL;Acc:Q9SY40]	1606702	1608665	38.8
AT4G03625		F-box/LRR protein [Source:UniProtKB/TrEMBL;Acc:A0A1P8B8Z3]	1608516	1608710	51.79
AT4G03630		Putative nodulin [Source:UniProtKB/TrEMBL;Acc:Q9SY41]	1608750	1610037	38.9
AT4G03635		F-box/LRR protein [Source:UniProtKB/TrEMBL;Acc:Q1G3K0]	1611651	1613117	40.29
AT4G04665			1639205	1639419	33.02
AT4G03645			1648416	1648994	36.27
AT4G03728		unknown protein; FUNCTIONS IN: molecular_function unknown; INVOLVED IN: biological_process unknown; LOCATED IN: cellular	1652242	1652976	40
AT4G03740		Uncharacterized protein AT4g03740 [Source:UniProtKB/TrEMBL;Acc:Q9SY52]	1661992	1663515	35.5
AT4G03811		other RNA [Source:TAIR;Acc:AT4G03811]	1756584	1757097	33.27
AT4G04675			1757453	1757738	39.86
AT4G03820		Protein of unknown function (DUF3537) [Source:TAIR;Acc:AT4G03820]	1771833	1774532	38.67
AT4G04755			1785645	1786404	35.26
AT4G04765			1785788	1786439	36.81
AT4G03830		Uncharacterized protein AT4g03830 [Source:UniProtKB/TrEMBL;Acc:Q9M107]	1790440	1792458	50.92
AT4G03935		other RNA [Source:TAIR;Acc:AT4G03935]	1868783	1869715	34.3
AT4G03930	PME42	Probable pectinesterase/pectinesterase inhibitor 42 [Source:UniProtKB/Swiss-Prot;Acc:Q1PEC0]	1870422	1872387	40.08
AT4G03940		T24M8.7 protein [Source:UniProtKB/TrEMBL;Acc:O81515]	1873624	1875660	40.21
AT4G03945		Plant invertase/pectin methylesterase inhibitor superfamily protein [Source:UniProtKB/TrEMBL;Acc:Q1G390]	1877872	1878545	31.75
AT4G03950		Putative glucose-6-phosphate/phosphate-translocator-like protein 1 [Source:UniProtKB/Swiss-Prot;Acc:O81514]	1879518	1881111	39.27
AT4G03960	DSP4	Probable tyrosine-protein phosphatase DSP4 [Source:UniProtKB/Swiss-Prot;Acc:Q940L5]	1887462	1889255	36.12
AT4G03965		RING/U-box superfamily protein [Source:UniProtKB/TrEMBL;Acc:Q5XV87]	1889360	1890412	43.3
AT4G04020	PAP1	Probable plastid-lipid-associated protein 1, chloroplastic [Source:UniProtKB/Swiss-Prot;Acc:O81439]	1931883	1933950	40.18
AT4G04025		Transcription repressor [Source:UniProtKB/TrEMBL;Acc:A0A1P8B3X9]	1934009	1934635	39.55
AT4G04030	ATOFP9	ovate family protein 9 [Source:TAIR;Acc:AT4G04030]	1936653	1937972	43.18
AT4G04040	PFP-BETA2	Pyrophosphate--fructose 6-phosphate 1-phosphotransferase subunit beta 2 [Source:UniProtKB/Swiss-Prot;Acc:F4JGR5]	1939113	1943024	36.38
AT4G04077		other RNA [Source:TAIR;Acc:AT4G04077]	1961126	1961951	38.74
AT4G04078		unknown protein; BEST Arabidopsis thaliana protein match is: unknown protein (TAIR:AT3G42148.1); Ha. [Source:TAIR;Acc:AT4G040	1961640	1961747	50.93
AT4G04080	ISU3	Iron-sulfur cluster assembly protein 3 [Source:UniProtKB/Swiss-Prot;Acc:O81433]	1963291	1964471	36.66
AT4G04090		Putative BTB/POZ domain-containing protein At4g04090 [Source:UniProtKB/Swiss-Prot;Acc:O81432]	1964980	1965650	41.73
AT4G04110		Putative RPP1-like disease resistance protein [Source:UniProtKB/TrEMBL;Acc:O81430]	1972591	1974014	37.29
AT4G04785			1996721	1997293	38.22
AT4G04180		P-loop containing nucleoside triphosphate hydrolases superfamily protein [Source:TAIR;Acc:AT4G04180]	2020420	2023992	38.79
AT4G04190		At4g04190 [Source:UniProtKB/TrEMBL;Acc:Q0IGH7]	2024445	2026517	35.84
AT4G04200		Microsomal signal peptidase 25 kDa subunit (SPC25) [Source:UniProtKB/TrEMBL;Acc:F4JGB4]	2027165	2029013	36.88
AT4G04210	PUX4	PUX4 [Source:UniProtKB/TrEMBL;Acc:A0A178V2K4]	2029855	2031942	39.08
AT4G04220	AtRLP46	Receptor-like protein 46 [Source:UniProtKB/Swiss-Prot;Acc:F4JGB6]	2033168	2035946	40.19
AT4G04221		other RNA [Source:TAIR;Acc:AT4G04221]	2033168	2036495	40.02
AT4G04223		other RNA [Source:TAIR;Acc:AT4G04223]	2036511	2037816	34.23
AT4G04260		Bromo-adjacent homology (BAH) domain-containing protein [Source:TAIR;Acc:AT4G04260]	2054037	2055849	34.97
AT4G04265		PttA/En/Spm family plant transposase [Source:UniProtKB/TrEMBL;Acc:A0A1P8B5V0]	2057570	2058299	39.45
AT4G04795			2075902	2076243	39.18
AT4G04805			2076002	2076433	39.58
AT4G04315		other RNA [Source:TAIR;Acc:AT4G04315]	2095259	2096518	33.02
AT4G04320		Malonyl-CoA decarboxylase family protein [Source:UniProtKB/TrEMBL;Acc:Q7Y1Z8]	2113341	2116693	37.28
AT4G04330	RBCX1	Chaperonin-like RBCX protein 1, chloroplastic [Source:UniProtKB/Swiss-Prot;Acc:Q94AU9]	2116553	2118617	31.72
AT4G04340	OSCA1	Protein OSCA1 [Source:UniProtKB/Swiss-Prot;Acc:Q9XEA1]	2122507	2126910	38.78
AT4G04350	EMB2369	Leucine--tRNA ligase, chloroplastic/mitochondrial [Source:UniProtKB/Swiss-Prot;Acc:Q9XEA0]	2128090	2133150	39.34
AT4G04360		Protein of unknown function (DUF1068) [Source:TAIR;Acc:AT4G04360]	2133139	2133935	42.41
AT4G04370	PCMP-E99	Pentatricopeptide repeat-containing protein At4g04370 [Source:UniProtKB/Swiss-Prot;Acc:Q9XE98]	2134060	2136321	41.11
AT4G04402		Two-component phosphorelay mediator [Source:UniProtKB/TrEMBL;Acc:F4JGC7]	2164007	2165404	35.48
AT4G04404		C2H2 and C2HC zinc fingers superfamily protein [Source:UniProtKB/TrEMBL;Acc:F4JGC8]	2166973	2167980	38.99
AT4G04815			2168144	2168414	38.01
AT4G04825			2179000	2179122	34.96
AT4G04835			2184135	2184629	37.78
AT4G04855			2186834	2187073	31.25
AT4G04865			2188106	2188645	41.48
AT4G04415			2194160	2195495	38.55
AT4G04875			2194585	2194815	39.39
AT4G04895			2197650	2198150	46.71
AT4G04423		unknown protein; Ha. [Source:TAIR;Acc:AT4G04423]	2203424	2204362	40.68
AT4G04450	WRKY42	WRKY transcription factor 42 [Source:UniProtKB/Swiss-Prot;Acc:Q9XEC3]	2218042	2221260	37.78
AT4G04460	APA3	Aspartic proteinase A3 [Source:UniProtKB/Swiss-Prot;Acc:Q9XEC4]	2224422	2227874	36.69
AT4G04470	PMP22	Peroxisomal membrane protein PMP22 [Source:UniProtKB/TrEMBL;Acc:Q0WTY9]	2227661	2230485	39.61
AT4G04480		F-box protein with a domain protein [Source:UniProtKB/TrEMBL;Acc:Q9XEC5]	2229649	2230851	44.22
AT4G04490	CRK36	Cysteine-rich receptor-like protein kinase 36 [Source:UniProtKB/Swiss-Prot;Acc:Q9XEC6]	2231717	2234689	38.21
AT4G04500	CRK37	Cysteine-rich receptor-like protein kinase 37 [Source:UniProtKB/Swiss-Prot;Acc:Q9XEC7]	2238370	2240921	39.46
AT4G04510	CRK38	cysteine-rich RLK (RECEPTOR-like protein kinase) 38 [Source:TAIR;Acc:AT4G04510]	2242072	2244930	39.7
AT4G04540	CRK39	Putative cysteine-rich receptor-like protein kinase 39 [Source:UniProtKB/Swiss-Prot;Acc:Q9SYS7]	2259498	2262320	38.86
AT4G04555		Myb family transcription factor [Source:UniProtKB/TrEMBL;Acc:A0A1P8B772]	2265647	2266799	28.62
AT4G04570	CRK40	Cysteine-rich receptor-like protein kinase 40 [Source:UniProtKB/Swiss-Prot;Acc:Q9SYS3]	2288616	2292965	35.56

AT4G04580		Homeodomain-like superfamily protein [Source:UniProtKB/TrEMBL;Acc:Q9XEB2]	2293704	2294906	31.01
AT4G04601		unknown protein; Ha. [Source:TAIR;Acc:AT4G04601]	2309413	2309571	37.74
AT4G04605		Myb-like DNA-binding domain protein [Source:UniProtKB/TrEMBL;Acc:A0A1P8B8R6]	2316471	2318152	30.5
AT4G04905			2322090	2322310	31.67
AT4G04610	01-Apr	5'-adenylylsulfate reductase 1, chloroplastic [Source:UniProtKB/Swiss-Prot;Acc:P92979]	2324878	2327064	40.28
AT4G04614		At4g04614 [Source:UniProtKB/TrEMBL;Acc:Q1G3K2]	2327044	2328264	38
AT4G04620	ATG8B	Autophagy-related protein 8b [Source:UniProtKB/Swiss-Prot;Acc:Q9XEB5]	2328442	2330590	34.62
AT4G04630		Putative uncharacterized protein [Source:UniProtKB/TrEMBL;Acc:Q8LFI5]	2331677	2332814	36.12
AT4G04632		Protein kinase superfamily protein [Source:UniProtKB/TrEMBL;Acc:F4JGV7]	2339586	2340049	39.87
AT4G04640	ATPC1	ATP synthase gamma chain 1, chloroplastic [Source:UniProtKB/Swiss-Prot;Acc:Q01908]	2350498	2352025	40.77
AT4G04650		AT4g04650 protein [Source:UniProtKB/TrEMBL;Acc:Q9S9V5]	2353989	2355213	44.57
AT4G04945			2356071	2356457	41.86
AT4G04670		tRNA wybutosine-synthesizing protein 2/3/4 [Source:UniProtKB/Swiss-Prot;Acc:Q8W4K1]	2367434	2372220	39.52
AT4G04680		INVOLVED IN: biological_process unknown; LOCATED IN: endomembrane system; BEST Arabidopsis thaliana protein match is: ARM	2372266	2373406	37.07
AT4G04690	FBX15	Putative F-box only protein 15 [Source:UniProtKB/Swiss-Prot;Acc:Q9S9V1]	2373999	2375135	41.25
AT4G04695	CPK31	Calcium-dependent protein kinase 31 [Source:UniProtKB/Swiss-Prot;Acc:Q9S9V0]	2381348	2384121	37.31
AT4G04700	CPK27	Calcium-dependent protein kinase 27 [Source:UniProtKB/Swiss-Prot;Acc:Q9ZSA4]	2385038	2388105	36.38
AT4G04710	CPK22	calcium-dependent protein kinase 22 [Source:TAIR;Acc:AT4G04710]	2390307	2392989	37.31
AT4G04720	CPK21	Calcium-dependent protein kinase 21 [Source:UniProtKB/Swiss-Prot;Acc:Q9ZSA2]	2393653	2397804	37.96
AT4G04730		Putative zinc finger protein [Source:UniProtKB/TrEMBL;Acc:Q9M102]	2401927	2402349	43.74
AT4G04740	CPK23	Calcium-dependent protein kinase 23 [Source:UniProtKB/TrEMBL;Acc:F4JGW8]	2402897	2408737	36.14
AT4G04745		At4g04745 [Source:UniProtKB/TrEMBL;Acc:Q6NL28]	2412618	2413447	35.66
AT4G04965			2416337	2416646	34.19
AT4G04750		Major facilitator superfamily protein [Source:TAIR;Acc:AT4G04750]	2417800	2422822	31.2
AT4G04760		Sugar transporter ERD6-like 15 [Source:UniProtKB/Swiss-Prot;Acc:Q9M0Z9]	2424030	2427769	32.97
AT4G04770	ABCI8	UPF0051 protein ABCI8, chloroplastic [Source:UniProtKB/Swiss-Prot;Acc:Q9ZS97]	2427686	2429899	39.57
AT4G04775		Uncharacterized protein At4g04775 [Source:UniProtKB/Swiss-Prot;Acc:Q9ZS96]	2431120	2431672	40.87
AT4G04780	MED21	Mediator of RNA polymerase II transcription subunit 21 [Source:UniProtKB/Swiss-Prot;Acc:C0LU16]	2432252	2433635	38.58
AT4G04975			2434368	2434857	42.45
AT4G04790		Pentatricopeptide repeat-containing protein At4g04790, mitochondrial [Source:UniProtKB/Swiss-Prot;Acc:Q6NQ81]	2434869	2439403	36.69
AT4G04800	MSRB3	Peptide methionine sulfoxide reductase B3 [Source:UniProtKB/Swiss-Prot;Acc:Q9M0Z6]	2439520	2441205	36.89
AT4G04810	MSRB4	Peptide methionine sulfoxide reductase B4 [Source:UniProtKB/Swiss-Prot;Acc:Q9M0Z5]	2441241	2442785	35.47
AT4G04830	MSRB5	Peptide methionine sulfoxide reductase B5 [Source:UniProtKB/Swiss-Prot;Acc:Q9ZS91]	2445775	2447086	35.82
AT4G04995			2448002	2448230	34.5
AT4G04840	MSRB6	MSRB6 [Source:UniProtKB/TrEMBL;Acc:A0A178V4R5]	2449434	2451651	32.33
AT4G05005			2451874	2452081	38.46
AT4G04850	KEA3	K(+) efflux antiporter 3, chloroplastic [Source:UniProtKB/Swiss-Prot;Acc:Q9M0Z3]	2452664	2457767	37.5
AT4G04860	DER2.2	Derlin-2.2 [Source:UniProtKB/Swiss-Prot;Acc:Q9ZS88]	2459853	2461739	39.48
AT4G05015			2460404	2460620	34.56
AT4G04870	CLS	Cardiolipin synthase (CMP-forming), mitochondrial [Source:UniProtKB/Swiss-Prot;Acc:Q93YW7]	2461674	2464150	38.6
AT4G04880		adenosine/AMP deaminase family protein [Source:TAIR;Acc:AT4G04880]	2464840	2467259	35.5
AT4G04885	PCFS4	Polyadenylation and cleavage factor homolog 4 [Source:UniProtKB/Swiss-Prot;Acc:Q0WPF2]	2471866	2476025	41.15
AT4G04890	PDF2	Homeobox-leucine zipper protein PROTODERMAL FACTOR 2 [Source:UniProtKB/Swiss-Prot;Acc:Q93V99]	2476390	2482447	35.74
AT4G04900	RIC10	CRIB domain-containing protein RIC10 [Source:UniProtKB/Swiss-Prot;Acc:Q9M0Y9]	2485994	2488611	29.41
AT4G04910	NSF	NSF [Source:UniProtKB/TrEMBL;Acc:A0A178UTB9]	2489364	2495863	36.97
AT4G04920	MED16	Mediator of RNA polymerase II transcription subunit 16 [Source:UniProtKB/Swiss-Prot;Acc:F4JGZ1]	2497598	2505554	40.07
AT4G04925		At4g04925 [Source:UniProtKB/TrEMBL;Acc:Q8LFB8]	2505979	2506786	44.55
AT4G04930	DES-1-LIKE	Sphingolipid delta(4)-desaturase DES1-like [Source:UniProtKB/Swiss-Prot;Acc:Q9ZPH4]	2509053	2511145	35.55
AT4G04940		Putative WD-repeat membrane protein [Source:UniProtKB/TrEMBL;Acc:Q93YS7]	2511028	2517217	37.17
AT4G04950	GRXS17	Monothiol glutaredoxin-S17 [Source:UniProtKB/Swiss-Prot;Acc:Q9ZPH2]	2517596	2520062	40.13
AT4G04955	ALN	Allantoinase [Source:UniProtKB/Swiss-Prot;Acc:Q94AP0]	2522034	2525352	38.42
AT4G04957		Domain of unknown function (DUF1985) [Source:TAIR;Acc:AT4G04957]	2526806	2527362	34.65
AT4G04960	LECRK71	L-type lectin-domain containing receptor kinase VII.1 [Source:UniProtKB/Swiss-Prot;Acc:Q9S9U1]	2532906	2535386	39.98
AT4G04970	CALS11	Callose synthase 11 [Source:UniProtKB/Swiss-Prot;Acc:Q9S9U0]	2536925	2543273	41.96
AT4G04972		unknown protein. [Source:TAIR;Acc:AT4G04972]	2543422	2544249	33.33
AT4G04980		unknown protein; BEST Arabidopsis thaliana protein match is: unknown protein (TAIR:AT1G11070.1); Ha. [Source:TAIR;Acc:AT4G049	2543692	2548130	36.67
AT4G04985		Eukaryotic aspartyl protease family protein [Source:UniProtKB/TrEMBL;Acc:Q3EAA9]	2548995	2549471	39.2
AT4G04990		AT4g04990 protein [Source:UniProtKB/TrEMBL;Acc:Q9S9T8]	2554997	2557258	33.73
AT4G05000	VPS28-2	Vacuolar protein sorting-associated protein 28 homolog [Source:UniProtKB/TrEMBL;Acc:A0A178V397]	2563040	2564835	37.58
AT4G05010		F-box protein At4g05010 [Source:UniProtKB/Swiss-Prot;Acc:Q9S9T6]	2567183	2568437	32.59
AT4G05018		unknown protein; FUNCTIONS IN: molecular_function unknown; INVOLVED IN: biological_process unknown; LOCATED IN: endom	2571040	2572208	37.81
AT4G05020	NDB2	NAD(P)H dehydrogenase B2 [Source:UniProtKB/TrEMBL;Acc:F4JGL5]	2572427	2576599	37.55
AT4G05030		AT4g05030 protein [Source:UniProtKB/TrEMBL;Acc:Q9S9T4]	2576677	2577974	35.29
AT4G05040		AT4G05040 protein [Source:UniProtKB/TrEMBL;Acc:Q94A85]	2578594	2582290	39.19
AT4G05025			2582655	2583046	50.77
AT4G05045			2586717	2586745	37.93
AT4G05055			2586794	2586946	41.18
AT4G05048	U49.1	U49.1; snoRNA [Source:TAIR;Acc:AT4G05048]	2586978	2587104	46.46
AT4G05050	UBQ11	Polyubiquitin [Source:UniProtKB/TrEMBL;Acc:Q8H0Y0]	2588004	2589437	42.75
AT4G05060	PVA43	Vesicle-associated protein 4-3 [Source:UniProtKB/Swiss-Prot;Acc:Q8LPQ7]	2590065	2592541	37.06
AT4G05065			2591712	2591988	45.13
AT4G05070		AT4g05070 protein [Source:UniProtKB/TrEMBL;Acc:Q9S9T1]	2593595	2594125	39.36
AT4G05071		unknown protein; Ha. [Source:TAIR;Acc:AT4G05071]	2594154	2594297	44.44
AT4G05080		F-box/kelch-repeat protein At4g05080 [Source:UniProtKB/Swiss-Prot;Acc:Q9S9T0]	2603001	2604176	38.44
AT4G05090		3'(2'),5'-bisphosphate nucleotidase-like protein [Source:UniProtKB/TrEMBL;Acc:Q682R6]	2609066	2611762	37.34
AT4G05091		unknown protein; Ha. [Source:TAIR;Acc:AT4G05091]	2611781	2612195	38.8
AT4G05095		BEST Arabidopsis thaliana protein match is: RNA-directed DNA polymerase (reverse transcriptase)-related family protein (TAIR:AT4G0	2613798	2614214	44.84
AT4G05097			2614015	2614563	46.27
AT4G05100	AtMYB74	Transcription factor MYB74 [Source:UniProtKB/Swiss-Prot;Acc:Q9M0Y5]	2618372	2619947	36.42
AT4G05110	ATENT6	equilibrative nucleoside transporter 6 [Source:TAIR;Acc:AT4G05110]	2624867	2629058	34.04
AT4G05105	MIR397A	MIR397A; miRNA [Source:TAIR;Acc:AT4G05105]	2625950	2626056	31.78
AT4G05120	ENT3	Equilibrative nucleotide transporter 3 [Source:UniProtKB/Swiss-Prot;Acc:Q9M0Y3]	2632524	2635143	36.53
AT4G05130	ENT4	Equilibrative nucleotide transporter 4 [Source:UniProtKB/Swiss-Prot;Acc:Q9M0Y2]	2636547	2639279	34.28
AT4G05140	ENT5	Equilibrative nucleotide transporter 5 [Source:UniProtKB/Swiss-Prot;Acc:Q9M0Y1]	2651015	2653208	35.32
AT4G05075			2655694	2656034	39.59
AT4G05150		AT4g05150/C17L7_70 [Source:UniProtKB/TrEMBL;Acc:Q940N7]	2660156	2662908	38.03
AT4G05160	4CLL7	4-coumarate--CoA ligase-like 7 [Source:UniProtKB/Swiss-Prot;Acc:Q9M0X9]	2664314	2666973	39.29
AT4G05170		basic helix-loop-helix (bHLH) DNA-binding superfamily protein [Source:TAIR;Acc:AT4G05170]	2667798	2669441	34.85
AT4G05180	PSBQ2	Oxygen-evolving enhancer protein 3-2, chloroplastic [Source:UniProtKB/Swiss-Prot;Acc:Q41932]	2671523	2673243	37.19
AT4G05190	KIN14D	Kinesin-like protein KIN-14D [Source:UniProtKB/Swiss-Prot;Acc:F4JGP4]	2675064	2680214	38.25
AT4G05200	CRK25	cysteine-rich RLK (RECEPTOR-like protein kinase) 25 [Source:TAIR;Acc:AT4G05200]	2679723	2682384	39.14
AT4G05210	LPXD1	Probable UDP-3-O-acylglucosamine N-acyltransferase 1, mitochondrial [Source:UniProtKB/Swiss-Prot;Acc:F4JGP6]	2682613	2685896	38.37
AT4G05220		Late embryogenesis abundant (LEA) hydroxyproline-rich glycoprotein family [Source:UniProtKB/TrEMBL;Acc:Q9M0X3]	2684997	2685793	44.29
AT4G05230		Ubiquitin-like superfamily protein [Source:UniProtKB/TrEMBL;Acc:Q9M0X2]	2687003	2687623	42.83
AT4G05240		Ubiquitin-like superfamily protein [Source:UniProtKB/TrEMBL;Acc:Q9M0X1]	2688200	2689018	39.93
AT4G05250		Ubiquitin-like superfamily protein [Source:UniProtKB/TrEMBL;Acc:Q9M0X0]	2691460	2692416	41.8
AT4G05260		Ubiquitin-like superfamily protein [Source:UniProtKB/TrEMBL;Acc:Q9M0W9]	2693680	2694459	42.44
AT4G05270		Ubiquitin-like superfamily protein [Source:UniProtKB/TrEMBL;Acc:Q9M0W8]	2694939	2695328	44.1
AT4G05085			2714178	2714697	35.96
AT4G05310		Ubiquitin-like superfamily protein [Source:UniProtKB/TrEMBL;Acc:Q9M0W4]	2714765	2716012	43.03
AT4G05320	UBQ10	Polyubiquitin 10 [Source:UniProtKB/Swiss-Prot;Acc:Q8H159]	2717977	2720308	43.95
AT4G05330	AGD13	AGD13 [Source:UniProtKB/TrEMBL;Acc:A0A178V602]	2720493	2723214	34.72
AT4G05115			2724567	2725038	38.14
AT4G05125			2724942	2725201	34.62
AT4G05340		P-loop containing nucleoside triphosphate hydrolases superfamily protein [Source:UniProtKB/TrEMBL;Acc:Q9M0W1]	2725690	2726065	36.44
AT4G05350		RING/U-box superfamily protein [Source:UniProtKB/TrEMBL;Acc:Q9M0W0]	2726910	2727530	47.5
AT4G05360		Zinc knuckle (CCHC-type) family protein [Source:UniProtKB/TrEMBL;Acc:Q9M0V9]	2728204	2732337	38.7
AT4G05135			2732791	2733242	41.15
AT4G05380		P-loop containing nucleoside triphosphate hydrolases superfamily protein [Source:TAIR;Acc:AT4G05380]	2736109	2738255	38.19

AT4G05390	RFNR1	Ferredoxin--NADP reductase, root isozyme 1, chloroplastic [Source:UniProtKB/Swiss-Prot;Acc:Q9M0V6]	2738531	2740927	39.09
AT4G05400		At4g05400 [Source:UniProtKB/TrEMBL;Acc:Q9M0V5]	2741124	2742949	39.43
AT4G05155			2742346	2742762	32.61
AT4G05410	YAO	U3 snoRNP-associated protein-like YAO [Source:UniProtKB/Swiss-Prot;Acc:Q9M0V4]	2742932	2745635	39.28
AT4G05420	DDB1A	DDB1A [Source:UniProtKB/TrEMBL;Acc:A0A178UYD4]	2746114	2753014	37.75
AT4G05430		Carbohydrate-binding X8 domain superfamily protein [Source:TAIR;Acc:AT4G05430]	2753124	2754586	34.24
AT4G05440	EDA35	Cell division cycle protein 123 homolog [Source:UniProtKB/TrEMBL;Acc:Q9M0V1]	2757163	2758585	39.63
AT4G05450	MFDX1	Adrenodoxin-like protein 1, mitochondrial [Source:UniProtKB/Swiss-Prot;Acc:Q9M0V0]	2758643	2760912	37.09
AT4G05460	SKIP19	F-box protein SKIP19 [Source:UniProtKB/Swiss-Prot;Acc:Q9M0U9]	2760502	2762445	39.51
AT4G05470		RNI-like superfamily protein [Source:TAIR;Acc:AT4G05470]	2763211	2765351	36.85
AT4G05475		Putative F-box protein At4g05475 [Source:UniProtKB/Swiss-Prot;Acc:Q9M0U7]	2765962	2767957	36.97
AT4G05490	FBL22	Putative F-box/LRR-repeat protein 22 [Source:UniProtKB/Swiss-Prot;Acc:Q9M0U6]	2771044	2772252	41.52
AT4G05497	FBL9	Putative F-box/LRR-repeat protein 9 [Source:UniProtKB/Swiss-Prot;Acc:Q9S9V8]	2777723	2778839	41.63
AT4G05508	MIR405D	MIR405D; miRNA [Source:TAIR;Acc:AT4G05508]	2789656	2789741	33.72
AT4G05520	EHD2	EH domain-containing protein 2 [Source:UniProtKB/Swiss-Prot;Acc:B3LF48]	2804403	2808015	36.06
AT4G05523		BEST Arabidopsis thaliana protein match is: TAIR:AT1G13120.1; Ha. [Source:TAIR;Acc:AT4G05523]	2808054	2809025	38.07
AT4G05530	SDRA	Short-chain dehydrogenase/reductase SDRA [Source:UniProtKB/Swiss-Prot;Acc:Q9S9W2]	2816317	2818518	37.51
AT4G05540		P-loop containing nucleoside triphosphate hydrolases superfamily protein [Source:UniProtKB/TrEMBL;Acc:F4JGH5]	2818521	2821065	40.24
AT4G05553		Zinc knuckle (CCHC-type) family protein [Source:UniProtKB/TrEMBL;Acc:F4JGH6]	2826047	2826659	39.97
AT4G05555			2845303	2848125	36.77
AT4G05590		CONTAINS InterPro DOMAIN/s: Uncharacterised protein family UPF0041 (InterPro:IPR005336); BEST Arabidopsis thaliana protein ma	2907012	2908631	33.58
AT4G05612		unknown protein; FUNCTIONS IN: molecular_function unknown; INVOLVED IN: biological_process unknown; LOCATED IN: cellula	2959094	2963744	36.12
AT4G05620		Putative F-box protein At4g05620 [Source:UniProtKB/Swiss-Prot;Acc:Q9M0U2]	2985051	2985449	42.61
AT4G05630	ARF46	B3 domain-containing protein At4g05630 [Source:UniProtKB/Swiss-Prot;Acc:Q9M0U1]	2988448	2988822	33.6
AT4G05631		unknown protein; Ha. [Source:TAIR;Acc:AT4G05631]	2990006	2992618	39.19
AT4G05632		unknown protein; Ha. [Source:TAIR;Acc:AT4G05632]	3001664	3002411	45.05
AT4G06479		Nucleic acid binding / zinc ion binding protein [Source:UniProtKB/TrEMBL;Acc:F4JGI5]	3067503	3068769	46.01
AT4G06490		Protein of unknown function (DUF3287) [Source:TAIR;Acc:AT4G06490]	3116894	3117334	46.26
AT4G06526		Nucleic acid binding / zinc ion binding protein [Source:UniProtKB/TrEMBL;Acc:F4JGI7]	3318302	3320945	46.14
AT4G06534		unknown protein; FUNCTIONS IN: molecular_function unknown; INVOLVED IN: biological_process unknown; LOCATED IN: endom	3356535	3358162	42.2
AT4G06536		At4g06536 [Source:UniProtKB/TrEMBL;Acc:Q1PEB2]	3360719	3361516	40.6
AT4G06639		unknown protein; FUNCTIONS IN: molecular_function unknown; INVOLVED IN: biological_process unknown; LOCATED IN: cellula	3523431	3523937	47.34
AT4G06583		unknown protein; FUNCTIONS IN: molecular_function unknown; INVOLVED IN: biological_process unknown; LOCATED IN: cellula	3576112	3576681	50.88
AT4G06598		Uncharacterized protein At4g06598 [Source:UniProtKB/Swiss-Prot;Acc:Q8W3M7]	3663328	3664857	38.1
AT4G06599		Ubiquitin-like domain-containing CTD phosphatase [Source:UniProtKB/Swiss-Prot;Acc:Q8W3M6]	3665925	3667547	39.62
AT4G05205			3758734	3759286	47.2
AT4G06643			3758957	3759880	42.42
AT4G06634	YY1	Zinc finger transcription factor YY1 [Source:UniProtKB/Swiss-Prot;Acc:Q2V3L3]	3764241	3766671	37.93
AT4G06655		Transmembrane protein [Source:UniProtKB/TrEMBL;Acc:A0A1P8B634]	3880990	3881431	42.08
AT4G06676		Protein EI24 homolog [Source:UniProtKB/Swiss-Prot;Acc:Q5BPL5]	3898811	3901990	36.48
AT4G06688		FUNCTIONS IN: molecular_function unknown; INVOLVED IN: biological_process unknown; LOCATED IN: cellular_component unkn	3920693	3924437	45.55
AT4G06701		other RNA [Source:TAIR;Acc:AT4G06701]	3939266	3940797	34.99
AT4G06744		Uncharacterized protein At4g06744 [Source:UniProtKB/Swiss-Prot;Acc:Q8W3M4]	4070549	4072470	40.37
AT4G06746	RAP2-9	Ethylene-responsive transcription factor RAP2-9 [Source:UniProtKB/Swiss-Prot;Acc:Q8W3M3]	4073717	4074735	41.32
AT4G07325		Replication-like protein [Source:UniProtKB/TrEMBL;Acc:A0A1P8B3C0]	4164954	4165570	34.2
AT4G07350		AT4g07350 protein [Source:UniProtKB/TrEMBL;Acc:Q9S9X7]	4172609	4175227	38.22
AT4G07380		AT4g07380 protein [Source:UniProtKB/TrEMBL;Acc:Q9S9X6]	4192429	4193016	42.01
AT4G07390		Mannose-P-dolichol utilization defect 1 protein homolog 2 [Source:UniProtKB/Swiss-Prot;Acc:Q8VY63]	4195564	4197571	37.85
AT4G07400	FBL8	Putative F-box/LRR-repeat protein 8 [Source:UniProtKB/Swiss-Prot;Acc:Q9S9X4]	4197666	4199511	42.31
AT4G07410	PCN	WD repeat-containing protein PCN [Source:UniProtKB/Swiss-Prot;Acc:Q8RXU6]	4200506	4205356	40.09
AT4G07408		unknown protein; Ha. [Source:TAIR;Acc:AT4G07408]	4200595	4201226	41.61
AT4G07445			4265751	4266300	38.55
AT4G07455		Putative uncharacterized protein [Source:UniProtKB/TrEMBL;Acc:Q0WTS9]	4311373	4311961	29.54
AT4G07515		At4g07515 [Source:UniProtKB/TrEMBL;Acc:Q058H0]	4314957	4315510	31.77
AT4G07524		Ras-related small GTP-binding family protein [Source:UniProtKB/TrEMBL;Acc:F4JGT3]	4345348	4345975	44.11
AT4G07526		unknown protein; BEST Arabidopsis thaliana protein match is: unknown protein (TAIR:AT3G33528.1); Ha. [Source:TAIR;Acc:AT4G075	4358652	4360161	35.56
AT4G07666		unknown protein; Ha. [Source:TAIR;Acc:AT4G07666]	4456702	4458842	38.58
AT4G07670		F10A2.10 protein [Source:UniProtKB/TrEMBL;Acc:Q9XH28]	4462911	4464347	39.87
AT4G07675		unknown protein; BEST Arabidopsis thaliana protein match is: Plant protein 1589 of unknown function (TAIR:AT1G49700.2); Ha. [Sour	4466013	4467147	32.07
AT4G07740		T6L9.5 protein [Source:UniProtKB/TrEMBL;Acc:Q9XH18]	4539638	4540682	44.59
AT4G05215			4620776	4621090	34.29
AT4G07820		At4g07820 [Source:UniProtKB/TrEMBL;Acc:Q9ZPG2]	4636624	4637450	37.12
AT4G05225			4641102	4641967	33.95
AT4G07825		At4g07825 [Source:UniProtKB/TrEMBL;Acc:Q8GZ72]	4643747	4645338	42.71
AT4G07835			4659714	4660348	43.78
AT4G05235			4659865	4660254	39.23
AT4G05245			4667745	4668362	30.1
AT4G07868		Transmembrane protein [Source:UniProtKB/TrEMBL;Acc:Q5XV83]	4691762	4692653	41.26
AT4G07932		unknown protein; FUNCTIONS IN: molecular_function unknown; INVOLVED IN: biological_process unknown; LOCATED IN: endom	4732198	4733424	48
AT4G05255			4790903	4791176	36.13
AT4G07940		Pre-mRNA-splicing factor CWC22-like protein, putative (DUF3245) [Source:UniProtKB/TrEMBL;Acc:Q9ZQC1]	4792453	4794109	36.27
AT4G05275			4796764	4797004	49.38
AT4G07950		DNA-directed RNA polymerase subunit [Source:UniProtKB/TrEMBL;Acc:Q9ZQC0]	4797841	4799795	36.16
AT4G07960	CSLC12	CSLC12 [Source:UniProtKB/TrEMBL;Acc:A0A384KRZ4]	4802253	4805647	37.91
AT4G07965		unknown protein; Ha. [Source:TAIR;Acc:AT4G07965]	4808635	4809352	45.96
AT4G07990		Chaperone DnaJ-domain superfamily protein [Source:UniProtKB/TrEMBL;Acc:F4JG06]	4824624	4829167	37.21
AT4G05285			4824699	4825139	40.14
AT4G05295			4841574	4842389	35.42
AT4G07995			4842688	4843814	31.5
AT4G08025		ECA1 gametogenesis family protein (DUF784) [Source:UniProtKB/TrEMBL;Acc:Q1PEB1]	4846798	4847379	31.96
AT4G08028		Putative defensin-like protein 102 [Source:UniProtKB/Swiss-Prot;Acc:Q2V3L0]	4851530	4851882	31.16
AT4G08035		other RNA [Source:TAIR;Acc:AT4G08035]	4866554	4868343	35.36
AT4G08039		Putative defensin-like protein 312 [Source:UniProtKB/Swiss-Prot;Acc:Q2V3K9]	4884848	4886035	29.12
AT4G08040	ACS11	1-aminocyclopropane-1-carboxylate synthase 11 [Source:UniProtKB/Swiss-Prot;Acc:Q9S9U6]	4886962	4889224	37.12
AT4G05305			4889421	4889914	30.36
AT4G05315			4971300	4971726	35.6
AT4G05335			4971524	4971907	36.2
AT4G08097		BEST Arabidopsis thaliana protein match is: myosin heavy chain-related (TAIR:AT3G30230.1); Ha. [Source:TAIR;Acc:AT4G08097]	5004096	5005932	44.15
AT4G08116	MIR401	MIR401; miRNA [Source:TAIR;Acc:AT4G08116]	5020237	5020486	42.4
AT4G05355			5122215	5122841	29.98
AT4G08140		26S proteasome non-ATPase regulatory subunit-like protein [Source:UniProtKB/TrEMBL;Acc:Q9SYE4]	5135673	5136140	36.97
AT4G08150	KNAT1	Homeobox protein knotted-1-like 1 [Source:UniProtKB/Swiss-Prot;Acc:P46639]	5147699	5151159	34.64
AT4G05365			5156471	5156884	32.61
AT4G05375			5157396	5157671	36.96
AT4G05385			5157432	5157637	38.35
AT4G08160	XYN3	Endo-1,4-beta-xylanase 3 [Source:UniProtKB/Swiss-Prot;Acc:F4JG10]	5159033	5162867	36.38
AT4G08170	ITPK3	Inositol-tetrakisphosphate 1-kinase 3 [Source:UniProtKB/Swiss-Prot;Acc:Q9SUG3]	5163220	5167192	37.35
AT4G08180	ORP1C	Oxysterol-binding protein-related protein 1C [Source:UniProtKB/Swiss-Prot;Acc:Q8L751]	5169459	5174107	39.71
AT4G08190		P-loop containing nucleoside triphosphate hydrolases superfamily protein [Source:UniProtKB/TrEMBL;Acc:Q9SUG1]	5174523	5175476	37.63
AT4G08210	PCMP-E100	Pentatricopeptide repeat-containing protein At4g08210 [Source:UniProtKB/Swiss-Prot;Acc:Q9SUF9]	5183110	5185906	39.08
AT4G08230		Glycine-rich protein [Source:UniProtKB/TrEMBL;Acc:Q8VY68]	5187785	5189794	36.87
AT4G08240		At4g08240 [Source:UniProtKB/TrEMBL;Acc:Q9SUF6]	5194657	5196763	35.45
AT4G08250	SCL26	Scarecrow-like protein 26 [Source:UniProtKB/Swiss-Prot;Acc:Q9SUF5]	5196787	5198238	46.49
AT4G08260		Putative protein phosphatase 2C 53 [Source:UniProtKB/Swiss-Prot;Acc:Q9SUF4]	5200847	5202034	38.3
AT4G08263		FUNCTIONS IN: molecular_function unknown; INVOLVED IN: biological_process unknown; BEST Arabidopsis thaliana protein match	5211604	5212137	44.01
AT4G08267		HAT transposon superfamily protein [Source:UniProtKB/TrEMBL;Acc:F4JFZ6]	5216854	5217697	34.24
AT4G08270		Glutathione S-transferase T3-like protein [Source:UniProtKB/TrEMBL;Acc:Q9SUF3]	5218105	5218404	41.33
AT4G08280		Glutaredoxin-like protein [Source:UniProtKB/TrEMBL;Acc:Q9SUF2]	5230245	5232196	37.19
AT4G08290		WAT1-related protein At4g08290 [Source:UniProtKB/Swiss-Prot;Acc:Q9SUF1]	5238773	5241112	35.26

AT4G08300		WAT1-related protein At4g08300 [Source:UniProtKB/Swiss-Prot;Acc:Q501F8]	5244865	5248394	34.14
AT4G08310		DNA ligase [Source:UniProtKB/TrEMBL;Acc:Q9SUE9]	5248806	5252327	38.25
AT4G08320	TPR8	Tetratricopeptide repeat (TPR)-like superfamily protein [Source:UniProtKB/TrEMBL;Acc:F4JG03]	5252640	5255200	38.58
AT4G05395			5254688	5254959	45.96
AT4G08330		Uncharacterized protein At4g08330, chloroplastic [Source:UniProtKB/Swiss-Prot;Acc:Q9STN5]	5255120	5256778	36.47
AT4G05405			5255757	5256393	29.36
AT4G08345		pre-tRNA [Source:TAIR;Acc:AT4G08345]	5268128	5268213	54.65
AT4G08350	GTA02	Putative transcription elongation factor SPT5 homolog 1 [Source:UniProtKB/Swiss-Prot;Acc:Q9STN3]	5286158	5292270	39.36
AT4G05415			5292626	5292820	40.51
AT4G08360		KOW domain-containing protein [Source:UniProtKB/TrEMBL;Acc:Q9STN2]	5295756	5297084	35.67
AT4G05425			5299016	5299363	43.39
AT4G05435			5299266	5299471	45.63
AT4G08370		Extensin-like protein [Source:UniProtKB/TrEMBL;Acc:Q9STN1]	5301962	5303314	45.16
AT4G08380		Extensin-like protein [Source:UniProtKB/TrEMBL;Acc:Q9STN0]	5311093	5312406	48.78
AT4G08390	APXS	L-ascorbate peroxidase S, chloroplastic/mitochondrial [Source:UniProtKB/Swiss-Prot;Acc:Q42592]	5314871	5317963	37.31
AT4G05445			5318175	5318441	32.58
AT4G08395		unknown protein; Ha. [Source:TAIR;Acc:AT4G08395]	5319634	5320712	43.28
AT4G05455			5321227	5321643	30.94
AT4G08400		Extensin-like protein [Source:UniProtKB/TrEMBL;Acc:Q9STM8]	5323606	5325374	46.58
AT4G08406		unknown protein; LOCATED IN: endomembrane system; Ha. [Source:TAIR;Acc:AT4G08406]	5331769	5331909	35.46
AT4G08410		Extensin-like protein [Source:UniProtKB/TrEMBL;Acc:Q9STM7]	5338819	5341209	47.22
AT4G08430		Ulp1 protease family protein [Source:UniProtKB/TrEMBL;Acc:Q9SW62]	5347396	5350436	40.02
AT4G05465			5353044	5353365	34.16
AT4G05485			5364659	5364950	32.53
AT4G08450		Disease resistance protein (TIR-NBS-LRR class) family [Source:UniProtKB/TrEMBL;Acc:Q9SW60]	5365501	5371211	33.27
AT4G05525			5372082	5372568	40.25
AT4G05535			5374079	5375266	42.09
AT4G08455		BTB/POZ domain-containing protein [Source:TAIR;Acc:AT4G08455]	5375714	5376922	41.36
AT4G08460		AT4G08460 protein [Source:UniProtKB/TrEMBL;Acc:Q9M0T4]	5377064	5380882	37.99
AT4G08470	MAPKKK10	Mitogen-activated protein kinase kinase kinase 3 [Source:UniProtKB/Swiss-Prot;Acc:Q9M0T3]	5383688	5387260	38.68
AT4G05545			5387321	5387565	31.84
AT4G08480	MEKK2	Mitogen-activated protein kinase kinase kinase 9 [Source:UniProtKB/Swiss-Prot;Acc:O81472]	5387351	5391575	38.96
AT4G08485		Putative defensin-like protein 211 [Source:UniProtKB/Swiss-Prot;Acc:Q2V3K7]	5394425	5394782	31.01
AT4G08500	MEKK1	Mitogen-activated protein kinase kinase kinase 1 [Source:UniProtKB/Swiss-Prot;Acc:Q39008]	5403671	5407320	40.25
AT4G08510		At4g08510 [Source:UniProtKB/TrEMBL;Acc:O81469]	5410253	5413453	40.67
AT4G08520		Coatomer subunit zeta-3 [Source:UniProtKB/Swiss-Prot;Acc:Q8H1F4]	5417209	5420639	35.67
AT4G08530	LTP15	Non-specific lipid-transfer protein 15 [Source:UniProtKB/Swiss-Prot;Acc:Q9M0T1]	5421857	5422237	38.85
AT4G08535		F-box SKIP17-like protein [Source:UniProtKB/TrEMBL;Acc:A0A1P8B460]	5424946	5427501	34
AT4G05565			5427929	5428169	40.66
AT4G08540		DNA-directed RNA polymerase II protein [Source:UniProtKB/TrEMBL;Acc:Q8H1E1]	5433621	5436524	38.36
AT4G08545		LRR receptor-like protein [Source:UniProtKB/TrEMBL;Acc:A0A1P8B502]	5436600	5439167	30.3
AT4G08550		electron carriers;protein disulfide oxidoreductases [Source:TAIR;Acc:AT4G08550]	5444345	5446891	38.99
AT4G08555		At4g08555 [Source:UniProtKB/TrEMBL;Acc:Q8GWL6]	5448049	5448638	32.37
AT4G08560			5450434	5453183	35.49
AT4G08570	HIPP24	Heavy metal-associated isoprenylated plant protein 24 [Source:UniProtKB/Swiss-Prot;Acc:O81464]	5454580	5456100	35.9
AT4G08580		Microfibrillar-associated protein-like protein [Source:UniProtKB/TrEMBL;Acc:O22281]	5461398	5463907	39.72
AT4G08590	ORTHL	E3 ubiquitin-protein ligase ORTHRUS-LIKE 1 [Source:UniProtKB/Swiss-Prot;Acc:Q681I0]	5463794	5466747	38.69
AT4G08593		unknown protein; BEST Arabidopsis thaliana protein match is: unknown protein (TAIR:AT5G45095.1); Ha. [Source:TAIR;Acc:AT4G085	5469345	5470466	43.49
AT4G08620	SULTR1;1	Sulfate transporter 1.1 [Source:UniProtKB/Swiss-Prot;Acc:Q9SAY1]	5500313	5506313	31.96
AT4G08630		unknown protein; BEST Arabidopsis thaliana protein match is: unknown protein (TAIR:AT3G48860.2); Ha. [Source:TAIR;Acc:AT4G086	5506809	5512046	37.74
AT4G08640		ATP binding protein [Source:UniProtKB/TrEMBL;Acc:F4JIB7]	5513313	5514174	35.5
AT4G08670		Bifunctional inhibitor/lipid-transfer protein/seed storage 2S albumin superfamily protein [Source:UniProtKB/TrEMBL;Acc:Q2PE70]	5536545	5538310	33.47
AT4G08685	SAH7	SAH7 [Source:UniProtKB/TrEMBL;Acc:A0A178UVW6]	5550404	5551370	40.95
AT4G08690		AT4G08690 protein [Source:UniProtKB/TrEMBL;Acc:O22270]	5551315	5553216	36.75
AT4G08691		Putative uncharacterized protein [Source:UniProtKB/TrEMBL;Acc:Q1G388]	5555316	5556616	40.89
AT4G08700	PUP13	Probable purine permease 13 [Source:UniProtKB/Swiss-Prot;Acc:Q8RY83]	5565773	5567458	38.14
AT4G08730		RNA-binding protein [Source:UniProtKB/TrEMBL;Acc:Q9LDC3]	5577390	5578779	34.68
AT4G08740		Uncharacterized protein AT4g08740 [Source:UniProtKB/TrEMBL;Acc:Q9LE68]	5579034	5579745	49.72
AT4G08760		Uncharacterized protein AT4g08760 [Source:UniProtKB/TrEMBL;Acc:Q9LE31]	5584739	5591133	40.72
AT4G08770	PER37	Peroxidase 37 [Source:UniProtKB/Swiss-Prot;Acc:Q9LDN9]	5598037	5600398	34
AT4G08780	PER38	Peroxidase 38 [Source:UniProtKB/Swiss-Prot;Acc:Q9LDA4]	5604105	5608290	29.29
AT4G08790	NLP2	Nitrilase-like protein 2 [Source:UniProtKB/Swiss-Prot;Acc:Q94JV5]	5608148	5611215	35.53
AT4G08800		Protein kinase superfamily protein [Source:UniProtKB/TrEMBL;Acc:F4JID0]	5614134	5615919	37.23
AT4G08810	SUB1	SUB1 [Source:UniProtKB/TrEMBL;Acc:A0A178UWI5]	5615921	5618066	39.89
AT4G08840	APUM11	Pumilio homolog 11 [Source:UniProtKB/Swiss-Prot;Acc:Q9LDW3]	5631298	5633779	36.18
AT4G05605			5634932	5635365	36.87
AT4G08850	MIK2	MDIS1-interacting receptor like kinase 2 [Source:UniProtKB/Swiss-Prot;Acc:Q8VZG8]	5636479	5640952	38
AT4G08870	ARGAH2	ARGAH2 [Source:UniProtKB/TrEMBL;Acc:A0A178V1K8]	5646395	5648868	36.3
AT4G05615			5650797	5651048	35.71
AT4G08869		Defensin-like protein 210 [Source:UniProtKB/Swiss-Prot;Acc:Q2V3K4]	5657228	5659574	31.7
AT4G05625			5659241	5659700	50
AT4G08867		Putative uncharacterized protein [Source:UniProtKB/TrEMBL;Acc:Q2V3K6]	5659793	5660903	40.32
AT4G05645			5667894	5668474	40.62
AT4G08868		unknown protein; Ha. [Source:TAIR;Acc:AT4G08868]	5668435	5669270	45.81
AT4G05650			5669154	5669541	43.04
AT4G08874		BEST Arabidopsis thaliana protein match is: Beta-galactosidase related protein (TAIR:AT3G43580.1); Ha. [Source:TAIR;Acc:AT4G0887	5673259	5673809	40.83
AT4G08875		Defensin-like protein 209 [Source:UniProtKB/Swiss-Prot;Acc:Q2V3K2]	5673746	5674220	25.26
AT4G08876		Pyrophosphate-fructose-6-phosphate 1-phosphotransferase-related / pyrophosphate-dependent 6-phosphofructose-1-kinase-like protein [Sou	5677395	5677728	30.24
AT4G08878		Major facilitator superfamily protein [Source:UniProtKB/TrEMBL;Acc:F4JJ77]	5683180	5684022	48.99
AT4G08895		Inorganic phosphate transporter family protein [Source:UniProtKB/TrEMBL;Acc:F4JJ78]	5697896	5698615	45.56
AT4G05655			5699242	5699592	31.62
AT4G05660			5700720	5701278	37.21
AT4G08900	ARGAH1	Arginase 1, mitochondrial [Source:UniProtKB/Swiss-Prot;Acc:P46637]	5703311	5705439	38.84
AT4G08910		At4g08910 [Source:UniProtKB/TrEMBL;Acc:A2RVU4]	5712328	5713515	34.51
AT4G05665			5714987	5715190	39.71
AT4G05670			5715752	5716170	38.9
AT4G05675			5715807	5716202	38.89
AT4G08920	CRY1	Cryptochrome-1 [Source:UniProtKB/Swiss-Prot;Acc:Q43125]	5723651	5727268	39.05
AT4G08930	APRL6	5'-adenylylsulfate reductase-like 6 [Source:UniProtKB/Swiss-Prot;Acc:Q9ZPE9]	5729169	5730850	38.94
AT4G08940		At4g08940 [Source:UniProtKB/TrEMBL;Acc:Q6NQB8]	5730797	5732195	40.96
AT4G05680			5731144	5731408	43.4
AT4G08950	EXO	Protein EXORDIUM [Source:UniProtKB/Swiss-Prot;Acc:Q9ZPE7]	5740219	5741591	40.28
AT4G08960		Serine/threonine-protein phosphatase 2A activator [Source:UniProtKB/TrEMBL;Acc:Q9C5N3]	5746271	5748509	40.06
AT4G08980	FBW2	FBW2 [Source:UniProtKB/TrEMBL;Acc:A0A178UUC5]	5758014	5760569	35.88
AT4G08990	MET2	DNA (cytosine-5)-methyltransferase 2 [Source:UniProtKB/Swiss-Prot;Acc:Q9M0S8]	5764781	5770496	38.98
AT4G09000	GRF1	General regulatory factor 1 [Source:UniProtKB/TrEMBL;Acc:F4JJ94]	5775210	5777481	37.5
AT4G05685			5776265	5777106	33.14
AT4G09010	APX4	ascorbate peroxidase 4 [Source:TAIR;Acc:AT4G09010]	5777397	5779572	38.79
AT4G05690			5778891	5779084	39.69
AT4G09012		Mitochondrial ribosomal protein L27 [Source:TAIR;Acc:AT4G09012]	5780311	5783464	32.34
AT4G09020	ISA3	Isoamylase 3, chloroplastic [Source:UniProtKB/Swiss-Prot;Acc:Q9M0S5]	5783909	5789100	38.15
AT4G09030	AGP10	Classical arabinogalactan protein 10 [Source:UniProtKB/Swiss-Prot;Acc:Q9M0S4]	5792137	5792966	41.2
AT4G09035			5792289	5792625	55.79
AT4G09040		At4g09040 [Source:UniProtKB/TrEMBL;Acc:Q6NPL0]	5794529	5797420	36.96
AT4G09060		unknown protein; LOCATED IN: membrane; EXPRESSED IN: leaf; BEST Arabidopsis thaliana protein match is: unknown protein (TAII	5797613	5800383	36.02
AT4G05695			5800336	5800556	36.65
AT4G09070	MED20B	Mediator of RNA polymerase II transcription subunit 20b [Source:UniProtKB/Swiss-Prot;Acc:Q9M0S0]	5802308	5803392	37.05
AT4G09080	TOC75-IV	Outer membrane OMP85 family protein [Source:TAIR;Acc:AT4G09080]	5803788	5806422	38.06
AT4G09090		At4g09090 [Source:UniProtKB/TrEMBL;Acc:Q6NKV5]	5807220	5807741	33.91

	AT4G09100	ATL39	RING-H2 finger protein ATL39 [Source:UniProtKB/Swiss-Prot;Acc:Q9M0R7]	5811115	5811846	41.26
	AT4G09110	ATL35	Putative RING-H2 finger protein ATL35 [Source:UniProtKB/Swiss-Prot;Acc:Q9M0R6]	5812335	5813642	41.82
	AT4G09120	ATL36	Putative RING-H2 finger protein ATL36 [Source:UniProtKB/Swiss-Prot;Acc:Q9M0R5]	5813998	5815119	44.74
	AT4G09130	ATL37	Putative RING-H2 finger protein ATL37 [Source:UniProtKB/Swiss-Prot;Acc:Q9M0R4]	5815775	5816922	45.64
	AT4G09140	MLH1	DNA mismatch repair protein MLH1 [Source:UniProtKB/Swiss-Prot;Acc:Q9ZRV4]	5816942	5821066	38.69
	AT4G09150		T-complex protein 11 [Source:UniProtKB/TrEMBL;Acc:F4JJB2]	5828505	5833725	40.22
	AT4G09153	LCR36	Defensin-like protein 155 [Source:UniProtKB/Swiss-Prot;Acc:P82751]	5835020	5835413	32.74
	AT4G09160	PATL5	Patellin-5 [Source:UniProtKB/Swiss-Prot;Acc:Q9M0R2]	5839599	5842456	41.08
	AT4G09170		At4g09170 [Source:UniProtKB/TrEMBL;Acc:Q9M0R1]	5842945	5843783	33.85
	AT4G09180	BHLH81	Transcription factor bHLH81 [Source:UniProtKB/Swiss-Prot;Acc:Q9M0R0]	5847312	5849763	35.89
	AT4G05700			5850995	5851199	42.44
	AT4G09190		Putative F-box protein At4g09190 [Source:UniProtKB/Swiss-Prot;Acc:Q9M0Q9]	5855176	5856327	37.59
	AT4G09200		At4g09200 [Source:UniProtKB/TrEMBL;Acc:Q8RX25]	5859244	5861880	37.39
	AT4G09210		Transmembrane protein [Source:UniProtKB/TrEMBL;Acc:Q9M0Q7]	5870289	5871160	39.91
	AT4G09250		SPla/RYanodine receptor (SPRY) domain-containing protein [Source:UniProtKB/TrEMBL;Acc:Q9SMS3]	5876578	5878978	38.15
	AT4G09260		Transmembrane protein [Source:UniProtKB/TrEMBL;Acc:Q9SMS8]	5887592	5888463	40.25
	AT4G09300		LisH and RanBPM domains containing protein [Source:TAIR;Acc:AT4G09300]	5898761	5901022	37.4
	AT4G09310		At4g09200 [Source:UniProtKB/TrEMBL;Acc:Q8RX25]	5902813	5905449	37.39
	AT4G05705			5917486	5917729	47.54
	AT4G05710			5918647	5918848	42.57
	AT4G09320	NDK1	Nucleoside diphosphate kinase [Source:UniProtKB/TrEMBL;Acc:A0A178UXQ6]	5923484	5924366	37.94
	AT4G09340		SPla/RYanodine receptor (SPRY) domain-containing protein [Source:UniProtKB/TrEMBL;Acc:Q94BS7]	5924602	5927738	36.98
	AT4G09350	ndhT	NAD(P)H-quinone oxidoreductase subunit T, chloroplastic [Source:UniProtKB/Swiss-Prot;Acc:Q9SMS0]	5931110	5932475	36.9
	AT4G05715			5937697	5938281	40.68
	AT4G09355			5937725	5937751	44.44
	AT4G09360		NB-ARC domain-containing disease resistance protein [Source:UniProtKB/TrEMBL;Acc:Q9M0Q5]	5940186	5943280	42.55
	AT4G09420		Disease resistance protein (TIR-NBS class) [Source:UniProtKB/TrEMBL;Acc:Q9M0Q0]	5961979	5964054	38.82
	AT4G09430		Disease resistance protein (TIR-NBS-LRR class) family [Source:TAIR;Acc:AT4G09430]	5970908	5973113	39.62
	AT4G09432		Potential natural antisense gene, locus overlaps with AT4G09430 [Source:TAIR;Acc:AT4G09432]	5972915	5975195	41.91
	AT4G09435		Disease resistance protein (TIR-NBS-LRR class) family protein [Source:UniProtKB/TrEMBL;Acc:A0A1P8B723]	5973083	5974684	42.38
	AT4G09440		Uncharacterized protein AT4g09440 [Source:UniProtKB/TrEMBL;Acc:Q9M0P8]	5982215	5982748	42.51
	AT4G09450		Duplicated homeodomain-like superfamily protein [Source:UniProtKB/TrEMBL;Acc:Q9M0P7]	5983277	5984531	40.64
	AT4G05720			5985500	5985811	38.46
	AT4G09460	MYB6	Transcription repressor MYB6 [Source:UniProtKB/Swiss-Prot;Acc:Q38851]	5992908	5994258	36.64
	AT4G09462		Carbohydrate-binding X8 domain superfamily protein [Source:UniProtKB/TrEMBL;Acc:Q1G387]	5996803	5997270	35.04
	AT4G09464		Carbohydrate-binding X8 domain superfamily protein [Source:UniProtKB/TrEMBL;Acc:Q1G3J4]	5999071	5999608	35.32
	AT4G09465		Carbohydrate-binding X8 domain superfamily protein [Source:UniProtKB/TrEMBL;Acc:Q1G3J2]	6001351	6001815	35.7
	AT4G09466		Carbohydrate-binding X8 domain superfamily protein [Source:UniProtKB/TrEMBL;Acc:B3H4S7]	6003632	6004167	34.51
	AT4G09467		Carbohydrate-binding X8 domain superfamily protein [Source:UniProtKB/TrEMBL;Acc:Q1G3J4]	6005905	6006442	35.13
	AT4G09490		Polynucleotidyl transferase, ribonuclease H-like superfamily protein [Source:UniProtKB/TrEMBL;Acc:Q9M0P4]	6015562	6016575	36.98
	AT4G09500	UGT79B7	Glycosyltransferase (Fragment) [Source:UniProtKB/TrEMBL;Acc:W8Q6L4]	6018021	6019759	39.68
	AT4G09510	CINV2	Alkaline/neutral invertase CINV2 [Source:UniProtKB/Swiss-Prot;Acc:Q67XD9]	6021160	6024042	39.99
	AT4G09520		Cofactor-independent phosphoglycerate mutase [Source:UniProtKB/TrEMBL;Acc:F4JK17]	6024804	6027123	39.83
	AT4G09530		SAUR-like auxin-responsive protein family [Source:UniProtKB/TrEMBL;Acc:Q9M0P0]	6029238	6029817	39.31
	AT4G09545		Cysteine rich peptide [Source:UniProtKB/TrEMBL;Acc:A8MRC9]	6037565	6037937	39.41
	AT4G09550	GIP1	Mitotic-spindle organizing protein 1B [Source:UniProtKB/Swiss-Prot;Acc:Q9M0N8]	6039055	6040226	34.98
	AT4G09560	RMR4	Receptor homology region, transmembrane domain- and RING domain-containing protein 4 [Source:UniProtKB/Swiss-Prot;Acc:Q0WPW	6040944	6043849	39.61
	AT4G09570	CPK4	Calcium-dependent protein kinase 4 [Source:UniProtKB/Swiss-Prot;Acc:Q38869]	6049441	6052380	37.24
	AT4G09580		Uncharacterized membrane protein At4g09580 [Source:UniProtKB/Swiss-Prot;Acc:Q8L586]	6052352	6054454	36.95
	AT4G09585			6061648	6062128	32.22
	AT4G09589			6065666	6066323	39.36
	AT4G09590	NHL22	NDR1/HIN1-like 22 [Source:UniProtKB/TrEMBL;Acc:Q9SST3]	6066128	6066763	48.9
	AT4G09600	GASA3	Gibberellin-regulated protein 3 [Source:UniProtKB/Swiss-Prot;Acc:P46687]	6072804	6073646	37.25
	AT4G09610	GASA2	Gibberellin-regulated protein 2 [Source:UniProtKB/Swiss-Prot;Acc:P46688]	6074770	6075645	37.79
	AT4G09620		Mitochondrial transcription termination factor family protein [Source:UniProtKB/TrEMBL;Acc:F4JK28]	6076805	6079151	36.09
	AT4G09630		Transmembrane protein (DUF616) [Source:UniProtKB/TrEMBL;Acc:Q9SST1]	6083585	6088134	39.78
	AT4G09640		Probable magnesium transporter NIPA5 [Source:UniProtKB/Swiss-Prot;Acc:F4JKQ7]	6087768	6090995	39.37
MQTL-3/Chr4	AT4G09980	EMB1691	EMB1691 [Source:UniProtKB/TrEMBL;Acc:A0A178V0G2]	6247735	6252314	42.71
	AT4G09984	LCR34	Putative defensin-like protein 142 [Source:UniProtKB/Swiss-Prot;Acc:P82749]	6254210	6255550	26.77
	AT4G09990	GXM2	Glucuronoxylan 4-O-methyltransferase 2 [Source:UniProtKB/Swiss-Prot;Acc:Q9T0F7]	6258880	6260259	41.45
	AT4G10000		Thioredoxin family protein [Source:UniProtKB/TrEMBL;Acc:F4JKW0]	6260302	6263181	38.23
	AT4G10010		Protein kinase superfamily protein [Source:UniProtKB/TrEMBL;Acc:Q9T0F9]	6263509	6266938	39.91
	AT4G10020	HSD5	11-beta-hydroxysteroid dehydrogenase-like 5 [Source:UniProtKB/Swiss-Prot;Acc:Q9T0G0]	6268273	6270434	41.07
	AT4G05755			6269079	6270196	43.29
	AT4G10030		Alpha/beta-Hydrolases superfamily protein [Source:UniProtKB/TrEMBL;Acc:Q9T0G1]	6270241	6273442	39.66
	AT4G10040	CYTC-2	CYTC-2 [Source:UniProtKB/TrEMBL;Acc:A0A178UZN3]	6276948	6278622	36
	AT4G10050		Protein phosphatase methylesterase 1 [Source:UniProtKB/TrEMBL;Acc:Q94F26]	6284308	6287293	37.51
	AT4G10060		Non-lysosomal glucosylceramidase [Source:UniProtKB/TrEMBL;Acc:F4JLJ2]	6288821	6295402	35.95
	AT4G10070		KH domain-containing protein [Source:TAIR;Acc:AT4G10070]	6295393	6299867	42.17
	AT4G10080		Transmembrane protein [Source:UniProtKB/TrEMBL;Acc:Q8GSF0]	6300019	6302744	38.15
	AT4G10090	ELP6	ELP6 [Source:UniProtKB/TrEMBL;Acc:A0A178V2M9]	6305320	6307552	36.5
	AT4G05760			6308680	6308879	44
	AT4G10100	CNX7	Molybdopterin synthase sulfur carrier subunit [Source:UniProtKB/Swiss-Prot;Acc:Q9S7A3]	6308683	6309546	40.51
	AT4G10110		At4g10100 [Source:UniProtKB/TrEMBL;Acc:Q8GW25]	6309393	6310910	36.5
	AT4G10115	SCRL20	Putative defensin-like protein 236 [Source:UniProtKB/Swiss-Prot;Acc:P82639]	6311969	6312588	30.16
	AT4G10120	SPS4	Probable sucrose-phosphate synthase 4 [Source:UniProtKB/Swiss-Prot;Acc:F4JLK2]	6313914	6320094	39.56
	AT4G10130		DNAJ heat shock N-terminal domain-containing protein [Source:UniProtKB/TrEMBL;Acc:O82623]	6319928	6321688	38.5
	AT4G10140		Transmembrane protein [Source:UniProtKB/TrEMBL;Acc:Q9SN29]	6322313	6324694	36.31
	AT4G10150	ATL7	RING-H2 finger protein ATL7 [Source:UniProtKB/Swiss-Prot;Acc:Q9SN28]	6327917	6329751	35.97
	AT4G05765			6330142	6330510	39.57
	AT4G05770			6330179	6330510	39.76
	AT4G10160	ATL59	E3 ubiquitin-protein ligase ATL59 [Source:UniProtKB/Swiss-Prot;Acc:Q9SN27]	6335919	6337478	36.22
	AT4G10170	PHYL1.1	Phytolongin Phyl1.1 [Source:UniProtKB/Swiss-Prot;Acc:Q9SN26]	6344301	6345610	38.02
	AT4G10180	DET1	Light-mediated development protein DET1 [Source:UniProtKB/Swiss-Prot;Acc:P48732]	6346411	6349314	38.46
	AT4G10190		Putative F-box protein At4g10190 [Source:UniProtKB/Swiss-Prot;Acc:O82622]	6350928	6352031	39.49
	AT4G10200		TTF-type zinc finger protein with HAT dimerization domain-containing protein [Source:UniProtKB/TrEMBL;Acc:Q9SN25]	6352957	6355609	36.52
	AT4G10210		Protein of Unknown Function (DUF239) [Source:TAIR;Acc:AT4G10210]	6357052	6360377	29.83
	AT4G10220		Protein of Unknown Function (DUF239) [Source:TAIR;Acc:AT4G10220]	6362189	6365079	30.16
	AT4G10230		No-apical-meristem-associated carboxy-terminal domain protein [Source:UniProtKB/TrEMBL;Acc:O82618]	6366261	6367185	32.86
	AT4G10240	BBX23	Bbx23 [Source:UniProtKB/TrEMBL;Acc:A0A178UTH9]	6368829	6369950	35.83
	AT4G10250	HSP22.0	22.0 kDa heat shock protein [Source:UniProtKB/Swiss-Prot;Acc:Q38806]	6370338	6371372	37
	AT4G10260		Probable fructokinase-5 [Source:UniProtKB/Swiss-Prot;Acc:O82616]	6371377	6372789	40.69
	AT4G10265		At4g10262 [Source:UniProtKB/TrEMBL;Acc:Q8GY66]	6373008	6373667	38.33
	AT4G05775			6374674	6375110	40.27
	AT4G10270		At4g10270 [Source:UniProtKB/TrEMBL;Acc:O82615]	6374734	6375210	38.57
	AT4G10280		AT4g10280/F24G24_80 [Source:UniProtKB/TrEMBL;Acc:Q9SV93]	6375125	6376142	37.52
	AT4G10290		RmlC-like cupins superfamily protein [Source:UniProtKB/TrEMBL;Acc:F4JLM2]	6379701	6381829	30.72
	AT4G10300		At4g10300 [Source:UniProtKB/TrEMBL;Acc:Q9SV91]	6384451	6386239	36.11
	AT4G10305		Reverse transcriptase [Source:UniProtKB/TrEMBL;Acc:A0A1P8B5L0]	6390669	6391116	40.85
	AT4G10310	HKT1	Sodium transporter HKT1 [Source:UniProtKB/Swiss-Prot;Acc:Q84TI7]	6391854	6395922	31.85
	AT4G10320		Isoleucine--tRNA ligase, cytoplasmic [Source:UniProtKB/Swiss-Prot;Acc:F4JLM5]	6397271	6404672	37.75
	AT4G10330		Glycine-rich protein [Source:UniProtKB/TrEMBL;Acc:Q9SV88]	6406495	6407959	39.66
	AT4G10340	LHCB5	Chlorophyll a-b binding protein, chloroplastic [Source:UniProtKB/TrEMBL;Acc:A0A178UW55]	6408007	6409849	40.37
	AT4G10345	MIR832A	MIR832a; miRNA [Source:TAIR;Acc:AT4G10345]	6413010	6413342	39.04
	AT4G10350	BRN2	Protein BEARSKIN2 [Source:UniProtKB/Swiss-Prot;Acc:Q9SV87]	6415120	6416961	36.59
	AT4G10360		AT4G10360 protein [Source:UniProtKB/TrEMBL;Acc:Q93ZA9]	6420284	6422460	35.6
	AT4G10370		Cysteine/Histidine-rich C1 domain family protein [Source:UniProtKB/TrEMBL;Acc:F4JLN1]	6423353	6425678	40.28
	AT4G05780			6430009	6430575	44.44
	AT4G10380	NIP5-1	At4g10380 [Source:UniProtKB/TrEMBL;Acc:A3KPG0]	6431161	6435396	35.08

	AT4G10390		Probable receptor-like protein kinase At4g10390 [Source:UniProtKB/Swiss-Prot;Acc:Q9SV83]	6441596	6443554	38.69
	AT4G10400		FBD-associated F-box protein At4g10400 [Source:UniProtKB/Swiss-Prot;Acc:Q9SV82]	6446088	6448136	38.75
	AT4G10410		Leucine-rich repeat (LRR) family protein [Source:UniProtKB/TrEMBL;Acc:Q9SV81]	6448983	6449998	39.07
	AT4G10420		FBD / Leucine Rich Repeat domains containing protein [Source:UniProtKB/TrEMBL;Acc:F4JLN7]	6452811	6454209	37.24
	AT4G10430		TMPIT-like protein [Source:UniProtKB/TrEMBL;Acc:Q93XX0]	6454703	6458146	37.89
	AT4G10440		Probable methyltransferase PMT17 [Source:UniProtKB/Swiss-Prot;Acc:Q9SZX8]	6459539	6462092	38.53
	AT4G10450	RPL9D	60S ribosomal protein L9-2 [Source:UniProtKB/Swiss-Prot;Acc:Q9SZX9]	6462918	6464558	35.22
	AT4G10457	SCRL1	Putative defensin-like protein 225 [Source:UniProtKB/Swiss-Prot;Acc:P82620]	6466900	6467270	36.39
	AT4G10465	HIPP44	Heavy metal-associated isoprenylated plant protein 44 [Source:UniProtKB/Swiss-Prot;Acc:F4JMB8]	6473547	6475434	33.26
	AT4G10470		AT4g10470/F7L13_50 [Source:UniProtKB/TrEMBL;Acc:Q944H1]	6475984	6477883	37.53
	AT4G10480		Putative alpha NAC [Source:UniProtKB/TrEMBL;Acc:Q0WWN5]	6477876	6479171	40.2
	AT4G05785			6478619	6478855	37.13
	AT4G10490	DLO2	Protein DMR6-LIKE OXYGENASE 2 [Source:UniProtKB/Swiss-Prot;Acc:Q9ZSA7]	6483867	6485360	40.9
	AT4G05790			6485740	6486245	39.33
	AT4G05795			6485842	6486245	40.35
	AT4G10500	DLO1	Protein DMR6-LIKE OXYGENASE 1 [Source:UniProtKB/Swiss-Prot;Acc:Q9ZSA8]	6491017	6492578	40.65
	AT4G10507		other RNA [Source:TAIR;Acc:AT4G10507]	6494707	6495211	33.47
	AT4G10510	SBT3.7	Subtilisin-like protease SBT3.7 [Source:UniProtKB/Swiss-Prot;Acc:Q9SZY2]	6495799	6499205	40.68
	AT4G10520	SBT3.9	Subtilisin-like protease SBT3.9 [Source:UniProtKB/Swiss-Prot;Acc:Q9ZSB0]	6499794	6502933	40.41
	AT4G10530		Subtilase family protein [Source:TAIR;Acc:AT4G10530]	6508600	6511670	40.74
	AT4G10540	SBT3.8	Subtilisin-like protease SBT3.8 [Source:UniProtKB/Swiss-Prot;Acc:Q9SZY3]	6512515	6515743	40.63
	AT4G10550		Subtilase family protein [Source:TAIR;Acc:AT4G10550]	6516424	6520322	40.14
	AT4G10560	MEE53	Cysteine/Histidine-rich C1 domain family protein [Source:UniProtKB/TrEMBL;Acc:Q9ZSB4]	6520771	6523014	40.33
	AT4G10570	UBP10	Ubiquitin carboxyl-terminal hydrolase 10 [Source:UniProtKB/Swiss-Prot;Acc:Q9ZSB5]	6523225	6528330	38.86
	AT4G10590	UBP9	AT4G10590 protein [Source:UniProtKB/TrEMBL;Acc:B9DFI6]	6538406	6544915	37.11
	AT4G10595	LCR2	low-molecular-weight cysteine-rich 2 [Source:TAIR;Acc:AT4G10595]	6545340	6546616	25.29
	AT4G05800			6546647	6546827	42.54
	AT4G10600		RING/FYVE/PHD zinc finger superfamily protein [Source:UniProtKB/TrEMBL;Acc:Q9T0B9]	6547235	6548878	37.29
	AT4G10603		Putative defensin-like protein 169 [Source:UniProtKB/Swiss-Prot;Acc:Q2V3K0]	6550922	6551975	25.24
	AT4G10610	CID12	RBP37 [Source:UniProtKB/TrEMBL;Acc:A0A178UWM0]	6556982	6559602	39.22
	AT4G10613		RNA-directed DNA polymerase (Reverse transcriptase)-related family protein [Source:UniProtKB/TrEMBL;Acc:Q2V3J8]	6560363	6561726	39.22
	AT4G10620		F3H7.11 protein [Source:UniProtKB/TrEMBL;Acc:Q9ZSB8]	6564183	6566504	40.4
	AT4G10630		F3H7.9 protein [Source:UniProtKB/TrEMBL;Acc:Q9ZSB9]	6566625	6567740	46.77
	AT4G10640	IQD16	At4g10640 [Source:UniProtKB/TrEMBL;Acc:Q7XA83]	6571894	6574586	35.09
	AT4G10650	DGP2	DAR GTPase 2, mitochondrial [Source:UniProtKB/Swiss-Prot;Acc:O82497]	6573792	6576250	41.24
	AT4G05805			6580008	6580296	36.68
	AT4G10660		CDC68-like protein [Source:UniProtKB/TrEMBL;Acc:Q9T0C3]	6582535	6583488	42.87
	AT4G10670	GTC2	GTC2 [Source:UniProtKB/TrEMBL;Acc:F4JME7]	6584727	6586262	45.38
	AT4G10680		T12H20.13 protein [Source:UniProtKB/TrEMBL;Acc:O82494]	6587521	6588069	44.63
	AT4G10695		CDC68-like protein [Source:UniProtKB/TrEMBL;Acc:Q3EA58]	6593774	6594981	38.99
	AT4G10700		F-box/kelch-repeat protein [Source:UniProtKB/TrEMBL;Acc:O82492]	6597863	6598658	43.47
	AT4G10710	SPT16	FACT complex subunit SPT16 [Source:UniProtKB/Swiss-Prot;Acc:O82491]	6601903	6606357	42.56
	AT4G10720		Ankyrin repeat family protein [Source:UniProtKB/TrEMBL;Acc:Q4V397]	6607797	6609559	37.83
	AT4G10730		Protein kinase superfamily protein [Source:UniProtKB/TrEMBL;Acc:F4JMF4]	6609418	6614887	37.18
	AT4G05810			6614223	6614456	35.04
MQTL-4/Chr4	AT4G21690	GA3OX3	GA3OX3 [Source:UniProtKB/TrEMBL;Acc:A0A178V5K5]	11527229	11529060	38.54
	AT4G21700		DUF2921 family protein, putative (DUF2921) [Source:UniProtKB/TrEMBL;Acc:Q9SVS7]	11529956	11532878	42.46
	AT4G21705		Pentatricopeptide repeat-containing protein At4g21705, mitochondrial [Source:UniProtKB/Swiss-Prot;Acc:Q84JR3]	11532971	11534867	38.75
	AT4G21710	NRPB2	DNA-directed RNA polymerase II subunit 2 [Source:UniProtKB/Swiss-Prot;Acc:P38420]	11535404	11542405	38.67
	AT4G21720		At4g21720 [Source:UniProtKB/TrEMBL;Acc:Q9SVS5]	11542430	11544657	36.85
	AT4G21740		At4g21740 [Source:UniProtKB/TrEMBL;Acc:Q9SVS3]	11545631	11546716	37.85
	AT4G21745	RIC11	CRIB domain-containing protein RIC11 [Source:UniProtKB/Swiss-Prot;Acc:Q0WL69]	11548606	11550622	31.33
	AT4G21750	ATML1	ATML1 [Source:UniProtKB/TrEMBL;Acc:A0A178UZ61]	11555039	11560771	37.59
	AT4G21760	BGLU47	beta-glucosidase 47 [Source:TAIR;Acc:AT4G21760]	11561123	11563950	36.85
	AT4G21770		RNA pseudouridine synthase 6, chloroplastic [Source:UniProtKB/Swiss-Prot;Acc:Q9SVS0]	11563517	11566377	38.97
	AT4G21780		Uncharacterized protein At4g21780 [Source:UniProtKB/TrEMBL;Acc:Q9SVR9]	11566990	11568494	37.28
	AT4G21790	TOM1	TOM1 [Source:UniProtKB/TrEMBL;Acc:A0A178UT97]	11569524	11572595	36.95
	AT4G21800	QQT2	GPN-loop GTPase QQT2 [Source:UniProtKB/Swiss-Prot;Acc:Q8W586]	11572789	11575145	37.8
	AT4G21810	DER2.1	Derlin-2.1 [Source:UniProtKB/Swiss-Prot;Acc:Q8VZ96]	11575096	11577254	37.84
	AT4G07030			11577025	11577254	36.96
	AT4G21820		binding;calmodulin binding [Source:TAIR;Acc:AT4G21820]	11577419	11584073	37.78
	AT4G21830	MSRB7	Peptide methionine sulfoxide reductase B7 [Source:UniProtKB/Swiss-Prot;Acc:Q8VY86]	11584346	11585542	37.18
	AT4G21840	MSRB8	MSRB8 [Source:UniProtKB/TrEMBL;Acc:A0A178V0W9]	11586980	11588373	34.29
	AT4G21850	MSRB9	MSRB9 [Source:UniProtKB/TrEMBL;Acc:A0A178V287]	11591116	11592425	36.72
	AT4G21860	MSRB2	Peptide methionine sulfoxide reductase B2, chloroplastic [Source:UniProtKB/Swiss-Prot;Acc:Q9C5C8]	11599645	11601724	39.42
	AT4G21865		At4g21865 [Source:UniProtKB/TrEMBL;Acc:A0JPV5]	11601706	11603283	35.3
	AT4G21870	HSP15.4	15.4 kDa class V heat shock protein [Source:UniProtKB/Swiss-Prot;Acc:O49710]	11603481	11604637	33.62
	AT4G21880		Pentatricopeptide repeat-containing protein At4g21880, mitochondrial [Source:UniProtKB/Swiss-Prot;Acc:O49711]	11604986	11610651	36.5
	AT4G21890		At4g21890 [Source:UniProtKB/TrEMBL;Acc:O49712]	11610808	11612903	43.46
	AT4G21895		DNA binding protein [Source:UniProtKB/TrEMBL;Acc:B3H641]	11613225	11615063	37.63
	AT4G21900	PRORP3	Proteinaceous RNase P 3 [Source:UniProtKB/Swiss-Prot;Acc:F4JKB6]	11616338	11619421	38.55
	AT4G21902		unknown protein; FUNCTIONS IN: molecular_function unknown; INVOLVED IN: biological_process unknown; LOCATED IN: cellular	11619735	11620201	37.04
	AT4G21903		Protein DETOXIFICATION [Source:UniProtKB/TrEMBL;Acc:F4JKB8]	11620889	11624459	36.96
	AT4G21910		MATE efflux family protein [Source:TAIR;Acc:AT4G21910]	11625309	11631255	34.56
	AT4G21920		Uncharacterized protein AT4g21920 [Source:UniProtKB/TrEMBL;Acc:O49715]	11635877	11636720	36.49
	AT4G21926		unknown protein; FUNCTIONS IN: molecular_function unknown; INVOLVED IN: biological_process unknown; LOCATED IN: cellular	11638658	11640164	38.69
	AT4G21930		Uncharacterized protein At4g21930 [Source:UniProtKB/TrEMBL;Acc:Q8L864]	11639757	11640583	45.1
	AT4G21940	CPK15	Calcium-dependent protein kinase 15 [Source:UniProtKB/TrEMBL;Acc:F4JKC7]	11640735	11643762	36.1
	AT4G21950		unknown protein; Ha. [Source:TAIR;Acc:AT4G21950]	11644122	11644655	39.51
	AT4G21960	PER42	Peroxidase 42 [Source:UniProtKB/Swiss-Prot;Acc:Q9SB81]	11646109	11648644	39.79
	AT4G21970		Protein of unknown function, DUF584 [Source:TAIR;Acc:AT4G21970]	11653234	11654462	37.1
	AT4G21980	APG8A	Autophagy-related protein [Source:UniProtKB/TrEMBL;Acc:A8MS84]	11655562	11657034	35.3
	AT4G21990	03-Apr	5'-adenylylsulfate reductase 3, chloroplastic [Source:UniProtKB/Swiss-Prot;Acc:P92980]	11657008	11659167	39.86
	AT4G22000		At4g22000 [Source:UniProtKB/TrEMBL;Acc:O65448]	11659563	11660919	37.07
	AT4G22010	sks4	At4g22010 [Source:UniProtKB/TrEMBL;Acc:O65449]	11663186	11666716	37.61
	AT4G22030		F-box family protein with a domain of unknown function (DUF295) [Source:TAIR;Acc:AT4G22030]	11672349	11673748	44.36
	AT4G22035		F-box only protein [Source:UniProtKB/TrEMBL;Acc:A0A1P8B474]	11674333	11675463	36.25
	AT4G22050		Eukaryotic aspartyl protease family protein [Source:UniProtKB/TrEMBL;Acc:F4JKD9]	11683815	11685644	37.27
	AT4G07040			11685773	11686212	36.59
	AT4G22060		Probable F-box protein At4g22060 [Source:UniProtKB/Swiss-Prot;Acc:O65454]	11687595	11689021	33.36
	AT4G22070	WRKY31	Probable WRKY transcription factor 31 [Source:UniProtKB/Swiss-Prot;Acc:Q93WT0]	11691172	11694590	38.72
	AT4G07045			11699821	11700099	44.8
	AT4G07050			11699821	11700427	41.35
	AT4G22080	RHS14	Probable pectate lyase 16 [Source:UniProtKB/Swiss-Prot;Acc:O65456]	11700555	11702785	38.91
	AT4G22090		Putative pectate lyase 17 [Source:UniProtKB/Swiss-Prot;Acc:O65457]	11704003	11706042	39.31
	AT4G22100	BGLU3	Beta-glucosidase 3 [Source:UniProtKB/Swiss-Prot;Acc:O65458]	11707370	11709932	38.31
	AT4G22105	SCRL26	Putative defensin-like protein 235 [Source:UniProtKB/Swiss-Prot;Acc:P82645]	11710054	11710651	33.95
	AT4G22110		Alcohol dehydrogenase-like 5 [Source:UniProtKB/Swiss-Prot;Acc:Q0V7W6]	11711314	11714228	32.04
	AT4G22115	SCRL14	Putative defensin-like protein 234 [Source:UniProtKB/Swiss-Prot;Acc:P82633]	11714743	11715243	35.73
	AT4G22120	CSC1	Hyperosmolality-gated Ca2+ permeable channel 1.2 [Source:UniProtKB/TrEMBL;Acc:A0A097NUP4]	11715607	11719873	38.88
	AT4G22130	SRF8	SRF8 [Source:UniProtKB/TrEMBL;Acc:A0A384KGH4]	11723388	11727693	37.11
	AT4G22140	EBS	Chromatin remodeling protein EBS [Source:UniProtKB/Swiss-Prot;Acc:F4JL28]	11727706	11730509	38.45
	AT4G22150	PUX3	Plant UBX domain-containing protein 3 [Source:UniProtKB/Swiss-Prot;Acc:Q9SUG6]	11731149	11732674	38.66
	AT4G22160		Uncharacterized protein At4g22160 [Source:UniProtKB/Swiss-Prot;Acc:Q8L8L9]	11733032	11734146	39.37
	AT4G22165		Probable F-box protein At4g22165 [Source:UniProtKB/Swiss-Prot;Acc:P0CG94]	11734328	11735419	40.11
	AT4G22170		Putative F-box protein At4g22170 [Source:UniProtKB/Swiss-Prot;Acc:Q9SUG4]	11736472	11737767	40.05
	AT4G22180		Putative F-box protein At4g22180 [Source:UniProtKB/Swiss-Prot;Acc:O49624]	11738511	11739782	39.94
	AT4G22190		Serine/arginine repetitive matrix-like protein [Source:UniProtKB/TrEMBL;Acc:Q8L4C5]	11741780	11743318	42.76
	AT4G22200	AKT2	Potassium channel AKT2/3 [Source:UniProtKB/Swiss-Prot;Acc:Q38898]	11746490	11750698	37.23

	AT4G22210	LCR85	low-molecular-weight cysteine-rich 85 [Source:TAIR;Acc:AT4G22210]	11752395	11752816	38.86
	AT4G22212		Defensin-like protein 98 [Source:UniProtKB/Swiss-Prot;Acc:Q94AZ8]	11753842	11754752	34.69
	AT4G22214		Defensin-like protein 99 [Source:UniProtKB/Swiss-Prot;Acc:Q7X6T3]	11754912	11756270	35.98
	AT4G07055			11757370	11757923	40.25
	AT4G07060			11757440	11757674	42.98
	AT4G22217		Defensin-like protein 100 [Source:UniProtKB/Swiss-Prot;Acc:Q3E6U0]	11758578	11759098	41.27
	AT4G22220	ISU1	Iron-sulfur cluster assembly protein [Source:UniProtKB/TrEMBL;Acc:A0A178UZH3]	11759103	11761057	38.72
	AT4G22230		Defensin-like protein 96 [Source:UniProtKB/Swiss-Prot;Acc:Q8L7G7]	11762233	11762732	38.8
	AT4G22233		other RNA [Source:TAIR;Acc:AT4G22233]	11763229	11764250	36.5
	AT4G22235		Defensin-like protein 95 [Source:UniProtKB/Swiss-Prot;Acc:Q6NMS3]	11763625	11764328	36.79
	AT4G22240	PAP2	Probable plastid-lipid-associated protein 2, chloroplastic [Source:UniProtKB/Swiss-Prot;Acc:O49629]	11765803	11767422	42.59
	AT4G22250		RING/U-box superfamily protein [Source:UniProtKB/TrEMBL;Acc:Q8GXD8]	11767455	11768563	43.91
	AT4G22260	AOX4	Ubiquinol oxidase 4, chloroplastic/chromoplastic [Source:UniProtKB/Swiss-Prot;Acc:Q56X52]	11769668	11772599	37.07
	AT4G22270	MRB1	Transmembrane protein, putative (DUF3537) [Source:UniProtKB/TrEMBL;Acc:Q94AL2]	11773221	11776039	35.44
	AT4G22265		pre-tRNA [Source:TAIR;Acc:AT4G22265]	11774461	11774546	51.16
	AT4G22280		F-box/RNI-like superfamily protein [Source:UniProtKB/TrEMBL;Acc:A8MS33]	11775969	11778151	38.34
	AT4G07065			11777849	11778090	37.6
	AT4G22285		Ubiquitin C-terminal hydrolases superfamily protein [Source:UniProtKB/TrEMBL;Acc:Q8H148]	11778749	11782695	38.64
	AT4G22290		At4g22290 [Source:UniProtKB/TrEMBL;Acc:Q8L6X7]	11782840	11785937	40.8
	AT4G22300	SOBER1	Probable carboxylesterase SOBER1-like [Source:UniProtKB/TrEMBL;Acc:Q8GYK2]	11787299	11789312	37.54
	AT4G22305	SOBER1	Carboxylesterase SOBER1 [Source:UniProtKB/Swiss-Prot;Acc:Q84WK4]	11789327	11791207	38.65
	AT4G22310	MPC4	Mitochondrial pyruvate carrier [Source:UniProtKB/TrEMBL;Acc:A0A178USR1]	11791321	11793137	36.54
	AT4G22320		Golgin family A protein [Source:UniProtKB/TrEMBL;Acc:Q8GYJ0]	11792813	11794778	38.05
	AT4G07070			11795096	11795527	36.81
	AT4G07075			11795167	11795267	47.52
	AT4G07080			11795177	11795275	43.43
	AT4G22330	ATCES1	ATCES1 [Source:UniProtKB/TrEMBL;Acc:A0A178UXI2]	11797548	11799946	38.02
	AT4G22340	CDS2	Phosphatidate cytidylyltransferase [Source:UniProtKB/TrEMBL;Acc:F4JL60]	11799852	11803522	35.44
	AT4G22350		Ubiquitin C-terminal hydrolases superfamily protein [Source:UniProtKB/TrEMBL;Acc:F4JL64]	11803768	11807417	38.58
	AT4G22360		SWIB complex BAF60b domain-containing protein [Source:UniProtKB/TrEMBL;Acc:Q93YR5]	11807257	11810117	38.94
	AT4G22370		unknown protein; Ha. [Source:TAIR;Acc:AT4G22370]	11810169	11811602	38.91
	AT4G07085			11810298	11810875	39.62
	AT4G22380		At4g22380 [Source:UniProtKB/TrEMBL;Acc:Q8LCC7]	11811731	11813000	36.93
	AT4G22390		F-box protein At4g22390 [Source:UniProtKB/Swiss-Prot;Acc:Q9SUY0]	11813313	11815164	40.98
	AT4G22400		At4g22400 [Source:UniProtKB/TrEMBL;Acc:Q9SUX9]	11816729	11817912	41.89
	AT4G22410		Ubiquitin C-terminal hydrolases superfamily protein [Source:TAIR;Acc:AT4G22410]	11818033	11826143	43.53
	AT4G22430		Putative F-box/kelch-repeat protein At4g22430 [Source:UniProtKB/Swiss-Prot;Acc:Q1PE57]	11827659	11829039	38.09
	AT4G22440		Uncharacterized protein AT4g22440 [Source:UniProtKB/TrEMBL;Acc:Q9SUX5]	11831024	11832268	38.07
	AT4G22460		Bifunctional inhibitor/lipid-transfer protein/seed storage 2S albumin superfamily protein [Source:UniProtKB/TrEMBL;Acc:Q9SUX3]	11838960	11839659	40.14
	AT4G22463		Transmembrane protein [Source:UniProtKB/TrEMBL;Acc:A0A1P8B8X3]	11838997	11839571	41.39
	AT4G22467			11840119	11841517	46.18
	AT4G22470		Protease inhibitor/seed storage/lipid transfer protein (LTP) family protein [Source:UniProtKB/TrEMBL;Acc:F4JLV4]	11840134	11841450	47.08
	AT4G22475		Transmembrane protein [Source:UniProtKB/TrEMBL;Acc:A0A1P8B5W2]	11844242	11846630	50.02
	AT4G22485		Bifunctional inhibitor/lipid-transfer protein/seed storage 2S albumin superfamily protein [Source:UniProtKB/TrEMBL;Acc:A8MRQ5]	11844506	11846476	54.08
	AT4G22490		Bifunctional inhibitor/lipid-transfer protein/seed storage 2S albumin superfamily protein [Source:UniProtKB/TrEMBL;Acc:Q9SUX0]	11849653	11850436	39.54
	AT4G22495			11851144	11853026	51.41
	AT4G22505		Bifunctional inhibitor/lipid-transfer protein/seed storage 2S albumin superfamily protein [Source:UniProtKB/TrEMBL;Acc:F4JLV7]	11851155	11853018	51.66
	AT4G22510		unknown protein; FUNCTIONS IN: molecular_function unknown; INVOLVED IN: biological_process unknown; LOCATED IN: mitoch	11854796	11857417	35.66
	AT4G22513		Bifunctional inhibitor/lipid-transfer protein/seed storage 2S albumin superfamily protein [Source:UniProtKB/TrEMBL;Acc:A8MQX6]	11854809	11855439	40.41
	AT4G22517		Bifunctional inhibitor/lipid-transfer protein/seed storage 2S albumin superfamily protein [Source:UniProtKB/TrEMBL;Acc:A8MQX9]	11856743	11857362	38.71
	AT4G22530		S-adenosyl-L-methionine-dependent methyltransferases superfamily protein [Source:UniProtKB/TrEMBL;Acc:Q9SUW6]	11858444	11860426	41.55
	AT4G22520		Bifunctional inhibitor/lipid-transfer protein/seed storage 2S albumin superfamily protein [Source:UniProtKB/TrEMBL;Acc:Q9SUW7]	11858614	11858970	45.1
	AT4G22540	ORP2A	ORP2A [Source:UniProtKB/TrEMBL;Acc:A0A178UYT2]	11860409	11866475	36.62
	AT4G22550	LPPB	Probable lipid phosphate phosphatase beta [Source:UniProtKB/Swiss-Prot;Acc:Q9SUW4]	11877949	11879021	43.34
	AT4G22560		Sulfated surface-like glycoprotein [Source:UniProtKB/TrEMBL;Acc:Q9SUW3]	11880080	11881088	44.3
	AT4G22570	APT3	Adenine phosphoribosyltransferase 3 [Source:UniProtKB/Swiss-Prot;Acc:Q9SUW2]	11882066	11885533	31.98
	AT4G22580	GT19	Probable xyloglucan galactosyltransferase GT19 [Source:UniProtKB/Swiss-Prot;Acc:Q9SUW1]	11889098	11890808	42.14
	AT4G07090			11891731	11892178	44.87
	AT4G22590	TPPG	Trehalose 6-phosphate phosphatase [Source:UniProtKB/TrEMBL;Acc:A0A178UWC3]	11893499	11896480	36.05
	AT4G22600	INP1	Protein INAPERTURATE POLLEN1 [Source:UniProtKB/Swiss-Prot;Acc:Q9SUV9]	11900211	11901032	45.74
	AT4G22610		AT4g22610/F7K2_190 [Source:UniProtKB/TrEMBL;Acc:Q9SUV8]	11901703	11902642	38.09
	AT4G22620		Auxin-responsive family protein [Source:UniProtKB/TrEMBL;Acc:O49643]	11907355	11908357	39.38
	AT4G22630		Bifunctional inhibitor/lipid-transfer protein/seed storage 2S albumin superfamily protein [Source:UniProtKB/TrEMBL;Acc:O49644]	11908673	11910013	37.21
	AT4G22635		pre-tRNA [Source:TAIR;Acc:AT4G22635]	11910289	11910362	55.41
	AT4G22640		Bifunctional inhibitor/lipid-transfer protein/seed storage 2S albumin superfamily protein [Source:UniProtKB/TrEMBL;Acc:O49645]	11911575	11912665	34.46
	AT4G22650		Lipid transfer protein [Source:UniProtKB/TrEMBL;Acc:Q9SUV6]	11913557	11914027	43.31
	AT4G22660		Putative F-box protein At4g22660 [Source:UniProtKB/Swiss-Prot;Acc:O49647]	11914981	11916171	40.3
	AT4G22666		Bifunctional inhibitor/lipid-transfer protein/seed storage 2S albumin superfamily protein [Source:UniProtKB/TrEMBL;Acc:Q1G3I0]	11917057	11917959	38.98
	AT4G22670	AtHip1	FAM10 family protein At4g22670 [Source:UniProtKB/Swiss-Prot;Acc:Q93YR3]	11917987	11920971	40.74
	AT4G22680	MYB85	MYB transcription factor [Source:UniProtKB/TrEMBL;Acc:Q94GA6]	11922340	11924230	34
	AT4G22690	CYP706A1	Cytochrome P450, family 706, subfamily A, polypeptide 1 [Source:UniProtKB/TrEMBL;Acc:F4JLY4]	11929359	11931750	39.09
	AT4G22700	LBD32	LOB domain-containing protein 32 [Source:UniProtKB/Swiss-Prot;Acc:O49651]	11933297	11933886	37.12
	AT4G22710	CYP706A2	Cytochrome P450 - like protein [Source:UniProtKB/TrEMBL;Acc:O49652]	11934969	11936823	44.26
	AT4G22720		Probable tRNA N6-adenosine threonylcarbamoyltransferase [Source:UniProtKB/TrEMBL;Acc:O49653]	11936942	11938969	38.61
	AT4G22730		At4g22730 [Source:UniProtKB/TrEMBL;Acc:O49654]	11941001	11943885	39.72
	AT4G07095			11942107	11943027	41.48
	AT4G22740		AT4g22740/T12H17_130 [Source:UniProtKB/TrEMBL;Acc:Q940N4]	11943849	11946139	38.93
	AT4G22745	MBD1	MBD1 [Source:UniProtKB/TrEMBL;Acc:A0A178UWM3]	11947210	11948804	38.18
	AT4G22750	PAT13	Probable protein S-acyltransferase 13 [Source:UniProtKB/Swiss-Prot;Acc:Q94C49]	11949180	11951424	38.17
	AT4G22755	SMO1-3	SMO1-3 [Source:UniProtKB/TrEMBL;Acc:A0A178V2R2]	11951830	11953731	33.86
	AT4G22754		pre-tRNA [Source:TAIR;Acc:AT4G22754]	11954741	11954813	63.01
	AT4G22756	SMO1-2	Methylsterol monooxygenase 1-2 [Source:UniProtKB/Swiss-Prot;Acc:Q1EC69]	11954840	11956918	38
	AT4G22758		Uncharacterized protein At4g22758 [Source:UniProtKB/Swiss-Prot;Acc:Q56XJ7]	11958128	11960071	34.41
	AT4G22760	PCMP-E6	Pentatricopeptide repeat-containing protein At4g22760 [Source:UniProtKB/Swiss-Prot;Acc:P0C8Q5]	11960229	11963162	40.8
	AT4G07100			11962901	11963078	30.34
	AT4G22770	AHL2	AT-hook motif nuclear-localized protein 2 [Source:UniProtKB/Swiss-Prot;Acc:O49658]	11963579	11965769	38.98
MQTL-5/Chr4	AT5G03240	UBQ3	Ubiquitin 4 [Source:UniProtKB/TrEMBL;Acc:A0A1P8BGQ7]	771660	773515	40.25
	AT5G03250		BTB/POZ domain-containing protein At5g03250 [Source:UniProtKB/Swiss-Prot;Acc:Q9LYW0]	774374	777048	41.64
	AT5G03260	LAC11	Laccase-11 [Source:UniProtKB/Swiss-Prot;Acc:Q8VZA1]	777021	779424	43.22
	AT5G03270	LOG6	Cytokinin riboside 5'-monophosphate phosphoribohydrolase [Source:UniProtKB/TrEMBL;Acc:A0A178UQW2]	781725	784368	32.49
	AT5G03280	EIN2	Ethylene-insensitive protein 2 [Source:UniProtKB/Swiss-Prot;Acc:Q9S814]	787428	793404	42.26
	AT5G03285		other RNA [Source:TAIR;Acc:AT5G03285]	793514	793717	50.98
	AT5G03290	IDH5	Isocitrate dehydrogenase [NAD] catalytic subunit 5, mitochondrial [Source:UniProtKB/Swiss-Prot;Acc:Q945K7]	793974	796218	40.18
	AT5G03300	ADK2	Adenosine kinase 2 [Source:UniProtKB/Swiss-Prot;Acc:Q9LZG0]	796361	799810	37.97
	AT5G03310		Arabidopsis thaliana genomic DNA, chromosome 5, P1 clone:MOK16 [Source:UniProtKB/TrEMBL;Acc:Q9LZF9]	799224	799706	41.61
	AT5G00910			801292	801869	38.75
	AT5G03320	PCRK2	Serine/threonine-protein kinase PCRK2 [Source:UniProtKB/Swiss-Prot;Acc:Q9LZF8]	801947	804448	38.89
	AT5G03330		At5g03330 [Source:UniProtKB/TrEMBL;Acc:Q9LZF7]	806964	810401	35.92
	AT5G03340	CDC48E	Cell division control protein 48 homolog E [Source:UniProtKB/Swiss-Prot;Acc:Q9LZF6]	809830	813293	42.29
	AT5G03345		FUNCTIONS IN: molecular_function unknown; INVOLVED IN: biological_process unknown; LOCATED IN: endomembrane system; E	814004	815659	35.93
	AT5G03350	LLP	Lectin-like protein [Source:UniProtKB/Swiss-Prot;Acc:Q9LZF5]	815610	816670	43.83
	AT5G03355		Cysteine/histidine-rich C1 domain protein [Source:UniProtKB/TrEMBL;Acc:Q9LZF4]	817835	820008	42.09
	AT5G03360		DC1 domain-containing protein [Source:TAIR;Acc:AT5G03360]	822077	825184	42.18
	AT5G03370		Acylphosphatase [Source:UniProtKB/TrEMBL;Acc:Q9LZF2]	825537	827502	37.64
	AT5G03380	HIPP06	Heavy metal-associated isoprenylated plant protein 6 [Source:UniProtKB/Swiss-Prot;Acc:Q9LZF1]	832035	834549	37.1
	AT5G03390		At5g03390 [Source:UniProtKB/TrEMBL;Acc:Q9LZF0]	834859	836751	43.74
	AT5G03400		Uncharacterized protein F12E4_140 [Source:UniProtKB/TrEMBL;Acc:Q9LZE9]	837919	839202	34.42
	AT5G03406		Class II aaRS and biotin synthetases superfamily protein [Source:UniProtKB/TrEMBL;Acc:F4KFB0]	839754	841900	39.17
	AT5G03415	DPB	Transcription factor-like protein DPB [Source:UniProtKB/Swiss-Prot;Acc:Q9FNY2]	842289	845454	38.28

AT5G03420	PTST	Protein PTST homolog 3, chloroplastic [Source:UniProtKB/Swiss-Prot;Acc:F4KFB3]	845601	848914	38.47
AT5G00920			846529	846760	40.09
AT5G03430		Phosphoadenosine phosphosulfate (PAPS) reductase family protein [Source:UniProtKB/TrEMBL;Acc:Q9LZE4]	848864	853146	37.26
AT5G00925			850260	850444	39.46
AT5G03435		Anthranilate phosphoribosyltransferase-like protein [Source:UniProtKB/TrEMBL;Acc:Q9LZE5]	853293	855955	44.95
AT5G03440		At5g03440 [Source:UniProtKB/TrEMBL;Acc:Q8LBJ0]	856815	858444	37.67
AT5G03445		pre-tRNA [Source:TAIR;Acc:AT5G03445]	858536	858619	52.38
AT5G03450		At5g03450 [Source:UniProtKB/TrEMBL;Acc:Q9LZE2]	858735	862345	39.3
AT5G03452		pre-tRNA [Source:TAIR;Acc:AT5G03452]	862397	862469	58.9
AT5G03455	CDC25	Dual specificity phosphatase Cdc25 [Source:UniProtKB/Swiss-Prot;Acc:Q8GY31]	862556	864176	37.69
AT5G03460		Transmembrane protein [Source:UniProtKB/TrEMBL;Acc:Q9LZE0]	864261	865794	36.05
AT5G03470	B'ALPHA	Serine/threonine protein phosphatase 2A regulatory subunit [Source:UniProtKB/TrEMBL;Acc:A0A178UNX1]	866339	869252	38.64
AT5G03480		RNA-binding (RRM/RBD/RNP motifs) family protein [Source:UniProtKB/TrEMBL;Acc:Q9LZD9]	869059	870855	38.29
AT5G03490	UGT89A2	UDP-glycosyltransferase 89A2 [Source:UniProtKB/Swiss-Prot;Acc:Q9LZD8]	871204	873072	44.14
AT5G03495		RNA-binding (RRM/RBD/RNP motifs) family protein [Source:UniProtKB/TrEMBL;Acc:Q67YA0]	873293	875251	37.98
AT5G03500		Mediator complex, subunit Med7 [Source:TAIR;Acc:AT5G03500]	876354	878549	37.66
AT5G03510		C2H2-type zinc finger family protein [Source:UniProtKB/TrEMBL;Acc:Q9LZD5]	880148	881347	41.33
AT5G03520	RABE1D	RAB8C [Source:UniProtKB/TrEMBL;Acc:A0A178UJN5]	883311	885451	35.73
AT5G03530	RABC2A	RABC2A [Source:UniProtKB/TrEMBL;Acc:A0A178UJG3]	885437	887700	36.04
AT5G03540	ATEXO70A1	Exocyst subunit Exo70 family protein [Source:UniProtKB/TrEMBL;Acc:F4KGM7]	889385	894205	36.9
AT5G03545	AT4	At5g03545 [Source:UniProtKB/TrEMBL;Acc:Q9LZD2]	894582	895517	36.97
AT5G03550		MATH domain/coiled-coil protein [Source:UniProtKB/TrEMBL;Acc:Q9LZD1]	895655	896433	36.07
AT5G03553			896846	897746	36.63
AT5G03552	MIR822A	MIR822a; miRNA [Source:TAIR;Acc:AT5G03552]	897020	897356	32.94
AT5G03555	NCS1	Purine-uracil permease NCS1 [Source:UniProtKB/Swiss-Prot;Acc:Q9LZD0]	898165	900298	44.61
AT5G03560		Tetratricopeptide repeat (TPR)-like superfamily protein [Source:UniProtKB/TrEMBL;Acc:F4KGN4]	900315	902770	41.16
AT5G03570	IREG2	iron regulated 2 [Source:TAIR;Acc:AT5G03570]	903470	906833	35.97
AT5G03580		RNA-binding (RRM/RBD/RNP motifs) family protein [Source:UniProtKB/TrEMBL;Acc:F4KGN7]	906682	907440	34.78
AT5G03590		GDSL-like Lipase/Acylhydrolase superfamily protein [Source:TAIR;Acc:AT5G03590]	908251	910292	35.8
AT5G00930			910546	910883	37.87
AT5G03600		SGNH hydrolase-type esterase superfamily protein [Source:TAIR;Acc:AT5G03600]	912476	914553	36.62
AT5G03610		GDSL esterase/lipase At5g03610 [Source:UniProtKB/Swiss-Prot;Acc:Q9LZS7]	915385	918485	34.38
AT5G03620	SBT4.15	Subtilisin-like protease SBT4.15 [Source:UniProtKB/Swiss-Prot;Acc:Q9LZS6]	918689	921937	41.21
AT5G03630	MDAR2	Monodehydroascorbate reductase 2 [Source:UniProtKB/Swiss-Prot;Acc:Q93WJ8]	922048	924728	39.95
AT5G03640		Protein kinase superfamily protein [Source:UniProtKB/TrEMBL;Acc:Q9LZS4]	927136	930921	41.02
AT5G03650	SBE2.2	1,4-alpha-glucan-branching enzyme 2-2, chloroplastic/amyloplastic [Source:UniProtKB/Swiss-Prot;Acc:Q9LZS3]	931762	937702	37.77
AT5G03660		AT5g03660/F17C15_80 [Source:UniProtKB/TrEMBL;Acc:Q940P0]	937817	940115	37.02
AT5G03668		other RNA [Source:TAIR;Acc:AT5G03668]	947101	948967	38.08
AT5G03670		TRM28 [Source:UniProtKB/TrEMBL;Acc:A0A178UNJ0]	947146	950239	37.88
AT5G03680	PTL	Trihelix transcription factor PTL [Source:UniProtKB/Swiss-Prot;Acc:Q9LZS0]	957611	961047	36.81
AT5G03690	FBA4	Fructose-bisphosphate aldolase 4, cytosolic [Source:UniProtKB/Swiss-Prot;Acc:F4KGQ0]	963147	965327	39.29
AT5G03700		PAN domain-containing protein At5g03700 [Source:UniProtKB/Swiss-Prot;Acc:Q9LZR8]	965562	967432	41.64
AT5G00935			968641	968841	36.32
AT5G03705		pre-tRNA [Source:TAIR;Acc:AT5G03705]	970799	970879	51.85
AT5G03710		Putative uncharacterized protein F17C15_130 [Source:UniProtKB/TrEMBL;Acc:Q9LZR7]	970923	971627	37.73
AT5G03720	HSFA3	HSFA3 [Source:UniProtKB/TrEMBL;Acc:A0A178UHR4]	971142	974326	38.68
AT5G03730	CTR1	Serine/threonine-protein kinase CTR1 [Source:UniProtKB/Swiss-Prot;Acc:Q05609]	974360	979849	39.07
AT5G03740	HDT3	HDT3 [Source:UniProtKB/TrEMBL;Acc:A0A178UP12]	981859	984289	39.49
AT5G03745		pre-tRNA [Source:TAIR;Acc:AT5G03745]	984367	984449	55.42
AT5G03750		E3 ubiquitin-protein ligase [Source:UniProtKB/TrEMBL;Acc:Q9LZR4]	984796	985497	42.88
AT5G03760	CSLA9	Glucomannan 4-beta-mannosyltransferase 9 [Source:UniProtKB/Swiss-Prot;Acc:Q9LZR3]	985622	990630	34.24
AT5G03770	KDTA	Probable 3-deoxy-D-manno-octulosonic acid transferase, mitochondrial [Source:UniProtKB/Swiss-Prot;Acc:Q8VZA5]	995095	997849	39.42
AT5G03775		pre-tRNA [Source:TAIR;Acc:AT5G03775]	998942	999014	54.79
AT5G03780	TRFL10	TRF-like 10 (Fragment) [Source:UniProtKB/TrEMBL;Acc:C0SVN2]	999102	1001056	40.92
AT5G03790	ATHB-51	Putative homeobox-leucine zipper protein ATHB-51 [Source:UniProtKB/Swiss-Prot;Acc:Q9LZR0]	1004782	1006631	37.95
AT5G03795		Probable glycosyltransferase At5g03795 [Source:UniProtKB/Swiss-Prot;Acc:Q9FFN2]	1007390	1010613	36.51
AT5G03800	EMB175	Glycosyltransferase (Fragment) [Source:UniProtKB/TrEMBL;Acc:W8PUG9]	1010766	1013678	41.57
AT5G03810		GDSL esterase/lipase At5g03810 [Source:UniProtKB/Swiss-Prot;Acc:Q9FFN0]	1013939	1015345	40.87
AT5G03820		GDSL-like Lipase/Acylhydrolase family protein [Source:TAIR;Acc:AT5G03820]	1015586	1017514	38.52
AT5G03830		CDK inhibitor P21 binding protein [Source:UniProtKB/TrEMBL;Acc:F4KI15]	1018334	1020460	36.81
AT5G03840	TFL1	Protein TERMINAL FLOWER 1 [Source:UniProtKB/Swiss-Prot;Acc:P93003]	1024500	1025872	34.09
AT5G03850	RPS28A	40S ribosomal protein S28-1 [Source:UniProtKB/Swiss-Prot;Acc:Q9SR73]	1027887	1029131	37.83
AT5G00940			1030430	1031043	42.67
AT5G03860	MLS	Malate synthase [Source:UniProtKB/Swiss-Prot;Acc:Q9LZC3]	1032099	1034973	41.74
AT5G03870		Emb [Source:UniProtKB/TrEMBL;Acc:Q9LZC2]	1035578	1037370	38.98
AT5G03880		Thioredoxin family protein [Source:UniProtKB/TrEMBL;Acc:Q940I2]	1038381	1041480	37.58
AT5G03885			1041881	1043519	30.51
AT5G03890		Emb [Source:UniProtKB/TrEMBL;Acc:Q9LZC0]	1046981	1047955	36.21
AT5G03900		Uncharacterized protein At5g03900, chloroplastic [Source:UniProtKB/Swiss-Prot;Acc:Q8GW20]	1048162	1052439	37.21
AT5G03905		Iron-sulfur assembly protein IscA-like 2, mitochondrial [Source:UniProtKB/Swiss-Prot;Acc:Q8LCY2]	1052580	1054274	35.28
AT5G03910	ABCB29	ATH12 [Source:UniProtKB/TrEMBL;Acc:A0A178USA1]	1054078	1057172	40.06
AT5G03920		unknown protein; BEST Arabidopsis thaliana protein match is: unknown protein (TAIR:AT5G03930.1); Ha. [Source:TAIR;Acc:AT5G039	1058146	1058769	39.9
AT5G03930		unknown protein; BEST Arabidopsis thaliana protein match is: unknown protein (TAIR:AT5G03920.1); Ha. [Source:TAIR;Acc:AT5G039	1059504	1060154	40.25
AT5G03940	FFC	SRP54CP [Source:UniProtKB/TrEMBL;Acc:A0A178UD49]	1060106	1063425	39.58
AT5G03944		unknown protein; LOCATED IN: endomembrane system; Ha. [Source:TAIR;Acc:AT5G03944]	1064611	1064808	48.99
AT5G03960	IQD12	IQ-domain 12 [Source:TAIR;Acc:AT5G03960]	1068158	1070164	40.96
AT5G03970		F-box protein At5g03970 [Source:UniProtKB/Swiss-Prot;Acc:Q94A64]	1071498	1073489	41.52
AT5G03980		GDSL esterase/lipase At5g03980 [Source:UniProtKB/Swiss-Prot;Acc:Q9LZB2]	1074205	1075289	46.82
AT5G03990		FK506-binding-like protein [Source:UniProtKB/TrEMBL;Acc:Q93XY4]	1075671	1077549	37.73
AT5G03995		Putative uncharacterized protein [Source:UniProtKB/TrEMBL;Acc:Q0WRE7]	1077483	1078304	33.33
AT5G04000		unknown protein; Ha. [Source:TAIR;Acc:AT5G04000]	1079033	1079899	39.1
AT5G04010	NSFBX	Probable F-box protein At5g04010 [Source:UniProtKB/Swiss-Prot;Acc:Q5EAF6]	1080105	1081096	45.87
AT5G04020	PICBP	calmodulin binding [Source:TAIR;Acc:AT5G04020]	1081521	1086772	40.29
AT5G04030		Transmembrane protein [Source:UniProtKB/TrEMBL;Acc:Q9LZA7]	1088462	1088692	44.59
AT5G04040	SDP1	SDP1 [Source:UniProtKB/TrEMBL;Acc:A0A178UB07]	1089342	1093204	40.41
AT5G04045		Defensin-like protein 301 [Source:UniProtKB/Swiss-Prot;Acc:Q3E715]	1093679	1094230	31.7
AT5G04047		Plant self-incompatibility protein S1 family [Source:UniProtKB/TrEMBL;Acc:B3H5F7]	1095030	1095702	29.57
AT5G04050			1096016	1099042	42.48
AT5G04060		Probable methyltransferase PMT7 [Source:UniProtKB/Swiss-Prot;Acc:Q9LZA4]	1099029	1102058	40.23
AT5G04070		At5g04070 [Source:UniProtKB/TrEMBL;Acc:Q6NLR7]	1102071	1104446	37.29
AT5G04080		At5g04080 [Source:UniProtKB/TrEMBL;Acc:Q6NN03]	1104524	1105398	36
AT5G04090		Plant protein 1589 of unknown function [Source:TAIR;Acc:AT5G04090]	1105352	1108282	36.88
AT5G04110	GYRB3	DNA GYRASE B3 [Source:TAIR;Acc:AT5G04110]	1110342	1115007	37.55
AT5G04120	IPSP	Metal-independent phosphoserine phosphatase [Source:UniProtKB/Swiss-Prot;Acc:F4KI56]	1119937	1121813	35.59
AT5G04130	GYRBM	DNA gyrase subunit B, mitochondrial [Source:UniProtKB/Swiss-Prot;Acc:Q94BZ7]	1121800	1128144	37.16
AT5G04140	GLU1	glutamate synthase 1 [Source:TAIR;Acc:AT5G04140]	1129785	1138488	39.14
AT5G04150	BHLH101	basic helix-loop-helix (bHLH) DNA-binding superfamily protein [Source:TAIR;Acc:AT5G04150]	1138410	1139633	37.83
AT5G04160	UUAT1	UDP-URONIC ACID TRANSPORTER 1 [Source:UniProtKB/Swiss-Prot;Acc:Q9FYE5]	1142735	1145008	37.91
AT5G04170	CML50	Probable calcium-binding protein CML50 [Source:UniProtKB/Swiss-Prot;Acc:Q9FYE4]	1145431	1147781	40.83
AT5G04180	ACA3	Alpha carbonic anhydrase 3 [Source:UniProtKB/Swiss-Prot;Acc:Q9FYE3]	1147648	1149284	37.08
AT5G04190	PKS4	Protein PHYTOCHROME KINASE SUBSTRATE 4 [Source:UniProtKB/Swiss-Prot;Acc:Q9FYE2]	1150886	1152627	42.42
AT5G04200	AMC9	MCP2f [Source:UniProtKB/TrEMBL;Acc:A0A178U6S6]	1153740	1155040	43.66
AT5G04210		CCCH-type zinc fingerfamily protein with RNA-binding domain-containing protein [Source:UniProtKB/TrEMBL;Acc:Q9FYE0]	1155049	1155724	43.79
AT5G04220	SYT3	Synaptotagmin-3 [Source:UniProtKB/Swiss-Prot;Acc:Q7XA06]	1155725	1158944	38.39
AT5G04230	PAL3	Phenylalanine ammonia-lyase [Source:UniProtKB/TrEMBL;Acc:F4JW69]	1160569	1163957	42.4
AT5G04240	ELF6	ELF6 [Source:UniProtKB/TrEMBL;Acc:A0A178UAR7]	1169153	1174992	40.91
AT5G04238		unknown protein; Ha. [Source:TAIR;Acc:AT5G04238]	1169217	1169411	44.1
AT5G04250		At5g04250 [Source:UniProtKB/TrEMBL;Acc:Q8LBW2]	1176005	1179008	36.72
AT5G04260	WCRKC2	Thioredoxin-like 3-2, chloroplastic [Source:UniProtKB/Swiss-Prot;Acc:Q8VZT6]	1178644	1180060	39.1

AT5G04267		unknown protein; Ha. [Source:TAIR;Acc:AT5G04267]	1182025	1182294	42.22
AT5G04270		DHHC-type zinc finger family protein [Source:TAIR;Acc:AT5G04270]	1182626	1184863	38.2
AT5G04275	MIR172B	MIR172/MIR172B; miRNA [Source:TAIR;Acc:AT5G04275]	1188207	1188301	35.79
AT5G04280	RZ1C	Glycine-rich RNA-binding protein RZ1C [Source:UniProtKB/Swiss-Prot;Acc:Q8RWN5]	1192259	1195759	40.82
AT5G04290	RDM3	Protein RNA-directed DNA methylation 3 [Source:UniProtKB/Swiss-Prot;Acc:F4JW79]	1195969	1202936	42.65
AT5G04310		Pectate lyase [Source:UniProtKB/TrEMBL;Acc:F4JW80]	1202967	1207946	37.17
AT5G04320	SGO2	SHUGOSHIN 2 [Source:UniProtKB/Swiss-Prot;Acc:Q0WTB8]	1209197	1212578	37.55
AT5G04330	CYP84A4	Cytochrome P450 84A4 [Source:UniProtKB/Swiss-Prot;Acc:F4JW83]	1212532	1214439	46.23
AT5G04340	ZAT6	Zinc finger protein ZAT6 [Source:UniProtKB/Swiss-Prot;Acc:O22533]	1216058	1217362	42.15
AT5G04347		Plant self-incompatibility protein S1 family [Source:TAIR;Acc:AT5G04347]	1219777	1220612	34.69
AT5G04350		Plant self-incompatibility protein S1 family [Source:UniProtKB/TrEMBL;Acc:F4JW86]	1220829	1221269	40.14
AT5G04360	PU1	Pullulanase 1, chloroplastic [Source:UniProtKB/Swiss-Prot;Acc:Q8GTR4]	1221448	1228841	37.42
AT5G00950			1228957	1229271	39.37
AT5G00955			1229899	1230504	41.75
AT5G00960			1230181	1230463	45.58
AT5G04370	NAMT1	S-adenosyl-L-methionine-dependent methyltransferases superfamily protein [Source:UniProtKB/TrEMBL;Acc:Q0WL44]	1231609	1234147	33.4
AT5G04380		S-adenosyl-L-methionine-dependent methyltransferases superfamily protein [Source:TAIR;Acc:AT5G04380]	1234712	1236391	35.36
AT5G04390		C2H2-type zinc finger family protein [Source:UniProtKB/TrEMBL;Acc:Q84K31]	1238820	1240517	41.34
AT5G04395		NAC transcription factor-like protein [Source:UniProtKB/TrEMBL;Acc:A0A1P8BGF2]	1241555	1242374	44.15
AT5G04400	anac077	NAC domain containing protein 77 [Source:TAIR;Acc:AT5G04400]	1242500	1243358	41.33
AT5G04410	NAC078	NAC domain-containing protein 78 [Source:UniProtKB/Swiss-Prot;Acc:Q84K00]	1243684	1246684	39.75
AT5G00965			1244826	1245200	36.8
AT5G04420		AT5G04420 protein [Source:UniProtKB/TrEMBL;Acc:Q9LZ83]	1246604	1249943	39.58
AT5G04430	BTR1	binding to TOMV RNA 1L (long form) [Source:TAIR;Acc:AT5G04430]	1250248	1253953	37.83
AT5G04440		At5g04440 [Source:UniProtKB/TrEMBL;Acc:Q500X2]	1254757	1257040	40.11
AT5G04460		RING/U-box superfamily protein [Source:UniProtKB/TrEMBL;Acc:F4JWA1]	1259435	1264025	40.38
AT5G04470	SIM	Cyclin-dependent protein kinase inhibitor SIM [Source:UniProtKB/Swiss-Prot;Acc:Q9LZ78]	1266625	1267416	40.66
AT5G04475			1267020	1267357	45.86
AT5G04480		AT5g04480/T32M21_80 [Source:UniProtKB/TrEMBL;Acc:Q940Y7]	1271505	1278102	39.3
AT5G04490	VTE5	Phytol kinase 1, chloroplastic [Source:UniProtKB/Swiss-Prot;Acc:Q9LZ76]	1279577	1281746	39.08
AT5G04500		Glycosyltransferase family protein 64 protein C5 [Source:UniProtKB/Swiss-Prot;Acc:Q84WB7]	1283378	1286374	40.71
AT5G04510	PDPK1	3-phosphoinositide-dependent protein kinase 1 [Source:UniProtKB/Swiss-Prot;Acc:Q9XF67]	1286843	1289908	39.69
AT5G04520		3-oxoacyl-[acyl-carrier-protein] synthase-like protein [Source:UniProtKB/TrEMBL;Acc:Q9LZ73]	1289885	1291104	44.02
AT5G04530	KCS19	3-ketoacyl-CoA synthase 19 [Source:UniProtKB/Swiss-Prot;Acc:Q9LZ72]	1291630	1293723	41.02
AT5G00970			1294133	1294339	40.1
AT5G04540	MTM2	Phosphatidylinositol-3-phosphatase myotubularin-2 [Source:UniProtKB/Swiss-Prot;Acc:F4JWB3]	1296356	1302477	38.57
AT5G04550		AT5g04550/T32M21_140 [Source:UniProtKB/TrEMBL;Acc:Q9LZ71]	1303167	1306145	41.99
AT5G04560	DME	Transcriptional activator DEMETER [Source:UniProtKB/Swiss-Prot;Acc:Q8LK56]	1309099	1318520	40.66

MQTLs	Gene stable ID	Gene name	Gene description	Gene start (bp)	Gene end (bp)	Gene % GC conten
MQTL-1/Chr5	AT5G03240	UBQ3	Ubiquitin 4 [Source:UniProtKB/TrEMBL;Acc:A0A1P8BGQ7]	771660	773515	40.25
	AT5G03250		BTB/POZ domain-containing protein At5g03250 [Source:UniProtKB/Swiss-Prot;Acc:Q9LYW0]	774374	777048	41.64
	AT5G03260	LAC11	Laccase-11 [Source:UniProtKB/Swiss-Prot;Acc:Q8VZA1]	777021	779424	43.22
	AT5G03270	LOG6	Cytokinin riboside 5'-monophosphate phosphoribohydrolase [Source:UniProtKB/TrEMBL;Acc:A0A178UQW2]	781725	784368	32.49
	AT5G03280	EIN2	Ethylene-insensitive protein 2 [Source:UniProtKB/Swiss-Prot;Acc:Q9S814]	787428	793404	42.26
	AT5G03285		other RNA [Source:TAIR;Acc:AT5G03285]	793514	793717	50.98
	AT5G03290	IDH5	Isocitrate dehydrogenase [NAD] catalytic subunit 5, mitochondrial [Source:UniProtKB/Swiss-Prot;Acc:Q945K7]	793974	796218	40.18
	AT5G03300	ADK2	Adenosine kinase 2 [Source:UniProtKB/Swiss-Prot;Acc:Q9LZG0]	796361	799810	37.97
	AT5G03310		Arabidopsis thaliana genomic DNA, chromosome 5, P1 clone:MOK16 [Source:UniProtKB/TrEMBL;Acc:Q9LZF9]	799224	799706	41.61
	AT5G00910			801292	801869	38.75
	AT5G03320	PCRK2	Serine/threonine-protein kinase PCRK2 [Source:UniProtKB/Swiss-Prot;Acc:Q9LZF8]	801947	804448	38.89
	AT5G03330		At5g03330 [Source:UniProtKB/TrEMBL;Acc:Q9LZF7]	806964	810401	35.92
	AT5G03340	CDC48E	Cell division control protein 48 homolog E [Source:UniProtKB/Swiss-Prot;Acc:Q9LZF6]	809830	813293	42.29
	AT5G03345		FUNCTIONS IN: molecular_function unknown; INVOLVED IN: biological_process unknown; LOCATED IN: endomembrane system; EXPRES	814004	815659	35.93
	AT5G03350	LLP	Lectin-like protein [Source:UniProtKB/Swiss-Prot;Acc:Q9LZF5]	815610	816670	43.83
	AT5G03355		Cysteine/histidine-rich C1 domain protein [Source:UniProtKB/TrEMBL;Acc:Q9LZF4]	817835	820008	42.09
	AT5G03360		DC1 domain-containing protein [Source:TAIR;Acc:AT5G03360]	822077	825184	42.18
	AT5G03370		Acylphosphatase [Source:UniProtKB/TrEMBL;Acc:Q9LZF2]	825537	827502	37.64
	AT5G03380	HIPP06	Heavy metal-associated isoprenylated plant protein 6 [Source:UniProtKB/Swiss-Prot;Acc:Q9LZF1]	832035	834549	37.1
	AT5G03390		At5g03390 [Source:UniProtKB/TrEMBL;Acc:Q9LZF0]	834859	836751	43.74
	AT5G03400		Uncharacterized protein F12E4_140 [Source:UniProtKB/TrEMBL;Acc:Q9LZE9]	837919	839202	34.42
	AT5G03406		Class II aaRS and biotin synthetases superfamily protein [Source:UniProtKB/TrEMBL;Acc:F4KFB0]	839754	841900	39.17
	AT5G03415	DPB	Transcription factor-like protein DPB [Source:UniProtKB/Swiss-Prot;Acc:Q9FNY2]	842289	845454	38.28
	AT5G03420	PTST	Protein PTST homolog 3, chloroplastic [Source:UniProtKB/Swiss-Prot;Acc:F4KFB3]	845601	848914	38.47
	AT5G00920			846529	846760	40.09
	AT5G03430		Phosphoadenosine phosphosulfate (PAPS) reductase family protein [Source:UniProtKB/TrEMBL;Acc:Q9LZE4]	848864	853146	37.26
	AT5G00925			850260	850444	39.46
	AT5G03435		Anthranilate phosphoribosyltransferase-like protein [Source:UniProtKB/TrEMBL;Acc:Q9LZE5]	853293	855955	44.95
	AT5G03440		At5g03440 [Source:UniProtKB/TrEMBL;Acc:Q8LBJ0]	856815	858444	37.67
	AT5G03445		pre-tRNA [Source:TAIR;Acc:AT5G03445]	858536	858619	52.38
	AT5G03450		At5g03450 [Source:UniProtKB/TrEMBL;Acc:Q9LZE2]	858735	862345	39.3
	AT5G03452		pre-tRNA [Source:TAIR;Acc:AT5G03452]	862397	862469	58.9
	AT5G03455	CDC25	Dual specificity phosphatase Cdc25 [Source:UniProtKB/Swiss-Prot;Acc:Q8GY31]	862556	864176	37.69
	AT5G03460		Transmembrane protein [Source:UniProtKB/TrEMBL;Acc:Q9LZE0]	864261	865794	36.05
	AT5G03470	B'ALPHA	Serine/threonine protein phosphatase 2A regulatory subunit [Source:UniProtKB/TrEMBL;Acc:A0A178UNX1]	866339	869252	38.64
	AT5G03480		RNA-binding (RRM/RBD/RNP motifs) family protein [Source:UniProtKB/TrEMBL;Acc:Q9LZD9]	869059	870855	38.29
	AT5G03490	UGT89A2	UDP-glycosyltransferase 89A2 [Source:UniProtKB/Swiss-Prot;Acc:Q9LZD8]	871204	873072	44.14
	AT5G03495		RNA-binding (RRM/RBD/RNP motifs) family protein [Source:UniProtKB/TrEMBL;Acc:Q67YA0]	873293	875251	37.98
	AT5G03500		Mediator complex, subunit Med7 [Source:TAIR;Acc:AT5G03500]	876354	878549	37.66
	AT5G03510		C2H2-type zinc finger family protein [Source:UniProtKB/TrEMBL;Acc:Q9LZD5]	880148	881347	41.33
	AT5G03520	RABE1D	RAB8C [Source:UniProtKB/TrEMBL;Acc:A0A178UJN5]	883311	885451	35.73
	AT5G03530	RABC2A	RABC2A [Source:UniProtKB/TrEMBL;Acc:A0A178UJG3]	885437	887700	36.04
	AT5G03540	ATEXO70A1	Exocyst subunit Exo70 family protein [Source:UniProtKB/TrEMBL;Acc:F4KGM7]	889385	894205	36.9
	AT5G03545	AT4	At5g03545 [Source:UniProtKB/TrEMBL;Acc:Q9LZD2]	894582	895517	36.97
	AT5G03550		MATH domain/coiled-coil protein [Source:UniProtKB/TrEMBL;Acc:Q9LZD1]	895655	896433	36.07
	AT5G03553			896846	897746	36.63
	AT5G03552	MIR822A	MIR822a; miRNA [Source:TAIR;Acc:AT5G03552]	897020	897356	32.94
	AT5G03555	NCS1	Purine-uracil permease NCS1 [Source:UniProtKB/Swiss-Prot;Acc:Q9LZD0]	898165	900298	44.61
	AT5G03560		Tetratricopeptide repeat (TPR)-like superfamily protein [Source:UniProtKB/TrEMBL;Acc:F4KGN4]	900315	902770	41.16
	AT5G03570	IREG2	iron regulated 2 [Source:TAIR;Acc:AT5G03570]	903470	906833	35.97
	AT5G03580		RNA-binding (RRM/RBD/RNP motifs) family protein [Source:UniProtKB/TrEMBL;Acc:F4KGN7]	906682	907440	34.78
	AT5G03590		GDSL-like Lipase/Acylhydrolase superfamily protein [Source:TAIR;Acc:AT5G03590]	908251	910292	35.8
	AT5G00930			910546	910883	37.87
	AT5G03600		SGNH hydrolase-type esterase superfamily protein [Source:TAIR;Acc:AT5G03600]	912476	914553	36.62
	AT5G03610		GDSL esterase/lipase At5g03610 [Source:UniProtKB/Swiss-Prot;Acc:Q9LZS7]	915385	918485	34.38
	AT5G03620	SBT4.15	Subtilisin-like protease SBT4.15 [Source:UniProtKB/Swiss-Prot;Acc:Q9LZS6]	918689	921937	41.21
	AT5G03630	MDAR2	Monodehydroascorbate reductase 2 [Source:UniProtKB/Swiss-Prot;Acc:Q93WJ8]	922048	924728	39.95
	AT5G03640		Protein kinase superfamily protein [Source:UniProtKB/TrEMBL;Acc:Q9LZS4]	927136	930921	41.02
	AT5G03650	SBE2.2	1,4-alpha-glucan-branching enzyme 2-2, chloroplastic/amyloplastic [Source:UniProtKB/Swiss-Prot;Acc:Q9LZS3]	931762	937702	37.77
	AT5G03660		AT5g03660/F17C15_80 [Source:UniProtKB/TrEMBL;Acc:Q940P0]	937817	940115	37.02
	AT5G03668		other RNA [Source:TAIR;Acc:AT5G03668]	947101	948967	38.08
	AT5G03670		TRM28 [Source:UniProtKB/TrEMBL;Acc:A0A178UNJ0]	947146	950239	37.88
	AT5G03680	PTL	Trihelix transcription factor PTL [Source:UniProtKB/Swiss-Prot;Acc:Q9LZS0]	957611	961047	36.81
	AT5G03690	FBA4	Fructose-bisphosphate aldolase 4, cytosolic [Source:UniProtKB/Swiss-Prot;Acc:F4KGQ0]	963147	965327	39.29
	AT5G03700		PAN domain-containing protein At5g03700 [Source:UniProtKB/Swiss-Prot;Acc:Q9LZR8]	965562	967432	41.64
	AT5G00935			968641	968841	36.32
	AT5G03705		pre-tRNA [Source:TAIR;Acc:AT5G03705]	970799	970879	51.85
	AT5G03710		Putative uncharacterized protein F17C15_130 [Source:UniProtKB/TrEMBL;Acc:Q9LZR7]	970923	971627	37.73
	AT5G03720	HSFA3	HSFA3 [Source:UniProtKB/TrEMBL;Acc:A0A178UHR4]	971142	974326	38.68
	AT5G03730	CTR1	Serine/threonine-protein kinase CTR1 [Source:UniProtKB/Swiss-Prot;Acc:Q05609]	974360	979849	39.07
	AT5G03740	HDT3	HDT3 [Source:UniProtKB/TrEMBL;Acc:A0A178UP12]	981859	984289	39.49
	AT5G03745		pre-tRNA [Source:TAIR;Acc:AT5G03745]	984367	984449	55.42
	AT5G03750		E3 ubiquitin-protein ligase [Source:UniProtKB/TrEMBL;Acc:Q9LZR4]	984796	985497	42.88
	AT5G03760	CSLA9	Glucomannan 4-beta-mannosyltransferase 9 [Source:UniProtKB/Swiss-Prot;Acc:Q9LZR3]	985622	990630	34.24
	AT5G03770	KDTA	Probable 3-deoxy-D-manno-octulosonic acid transferase, mitochondrial [Source:UniProtKB/Swiss-Prot;Acc:Q8VZA5]	995095	997849	39.42
	AT5G03775		pre-tRNA [Source:TAIR;Acc:AT5G03775]	998942	999014	54.79
	AT5G03780	TRFL10	TRF-like 10 (Fragment) [Source:UniProtKB/TrEMBL;Acc:C0SVN2]	999102	1001056	40.92
	AT5G03790	ATHB-51	Putative homeobox-leucine zipper protein ATHB-51 [Source:UniProtKB/Swiss-Prot;Acc:Q9LZR0]	1004782	1006631	37.95
	AT5G03795		Probable glycosyltransferase At5g03795 [Source:UniProtKB/Swiss-Prot;Acc:Q9FFN2]	1007390	1010613	36.51
	AT5G03800	EMB175	Glycosyltransferase (Fragment) [Source:UniProtKB/TrEMBL;Acc:W8PUG9]	1010766	1013678	41.57
	AT5G03810		GDSL esterase/lipase At5g03810 [Source:UniProtKB/Swiss-Prot;Acc:Q9FFN0]	1013939	1015345	40.87
	AT5G03820		GDSL-like Lipase/Acylhydrolase family protein [Source:TAIR;Acc:AT5G03820]	1015586	1017514	38.52
	AT5G03830		CDK inhibitor P21 binding protein [Source:UniProtKB/TrEMBL;Acc:F4KI15]	1018334	1020460	36.81
	AT5G03840	TFL1	Protein TERMINAL FLOWER 1 [Source:UniProtKB/Swiss-Prot;Acc:P93003]	1024500	1025872	34.09
	AT5G03850	RPS28A	40S ribosomal protein S28-1 [Source:UniProtKB/Swiss-Prot;Acc:Q9SR73]	1027887	1029131	37.83
	AT5G00940			1030430	1031043	42.67
	AT5G03860	MLS	Malate synthase [Source:UniProtKB/Swiss-Prot;Acc:Q9LZC3]	1032099	1034973	41.74
	AT5G03870		Emb [Source:UniProtKB/TrEMBL;Acc:Q9LZC2]	1035578	1037370	38.98
	AT5G03880		Thioredoxin family protein [Source:UniProtKB/TrEMBL;Acc:Q940I2]	1038381	1041480	37.58
	AT5G03885			1041881	1043519	30.51
	AT5G03890		Emb [Source:UniProtKB/TrEMBL;Acc:Q9LZC0]	1046981	1047955	36.21
	AT5G03900		Uncharacterized protein At5g03900, chloroplastic [Source:UniProtKB/Swiss-Prot;Acc:Q8GW20]	1048162	1052439	37.21
	AT5G03905		Iron-sulfur assembly protein IscA-like 2, mitochondrial [Source:UniProtKB/Swiss-Prot;Acc:Q8LCY2]	1052580	1054274	35.28
	AT5G03910	ABCB29	ATH12 [Source:UniProtKB/TrEMBL;Acc:A0A178USA1]	1054078	1057172	40.06
	AT5G03920		unknown protein; BEST Arabidopsis thaliana protein match is: unknown protein (TAIR:AT5G03930.1); Ha. [Source:TAIR;Acc:AT5G03920]	1058146	1058769	39.9
	AT5G03930		unknown protein; BEST Arabidopsis thaliana protein match is: unknown protein (TAIR:AT5G03920.1); Ha. [Source:TAIR;Acc:AT5G03930]	1059504	1060154	40.25
	AT5G03940	FFC	SRP54CP [Source:UniProtKB/TrEMBL;Acc:A0A178UD49]	1060106	1063425	39.58
	AT5G03944		unknown protein; LOCATED IN: endomembrane system; Ha. [Source:TAIR;Acc:AT5G03944]	1064611	1064808	48.99
	AT5G03960	IQD12	IQ-domain 12 [Source:TAIR;Acc:AT5G03960]	1068158	1070164	40.96
	AT5G03970		F-box protein At5g03970 [Source:UniProtKB/Swiss-Prot;Acc:Q94A64]	1071498	1073489	41.52
	AT5G03980		GDSL esterase/lipase At5g03980 [Source:UniProtKB/Swiss-Prot;Acc:Q9LZB2]	1074205	1075289	46.82
	AT5G03990		FK506-binding-like protein [Source:UniProtKB/TrEMBL;Acc:Q93XY4]	1075671	1077549	37.73
	AT5G03995		Putative uncharacterized protein [Source:UniProtKB/TrEMBL;Acc:Q0WRE7]	1077483	1078304	33.33
	AT5G04000		unknown protein; Ha. [Source:TAIR;Acc:AT5G04000]	1079033	1079899	39.1

	AT5G04010	NSFBX	Probable F-box protein At5g04010 [Source:UniProtKB/Swiss-Prot;Acc:Q5EAF6]	1080105	1081096	45.87
	AT5G04020	PICBP	calmodulin binding [Source:TAIR;Acc:AT5G04020]	1081521	1086772	40.29
	AT5G04030		Transmembrane protein [Source:UniProtKB/TrEMBL;Acc:Q9LZA7]	1088462	1088692	44.59
	AT5G04040	SDP1	SDP1 [Source:UniProtKB/TrEMBL;Acc:A0A178UB07]	1089342	1093204	40.41
	AT5G04045		Defensin-like protein 301 [Source:UniProtKB/Swiss-Prot;Acc:Q3E715]	1093679	1094230	31.7
	AT5G04047		Plant self-incompatibility protein S1 family [Source:UniProtKB/TrEMBL;Acc:B3H5F7]	1095030	1095702	29.57
	AT5G04050			1096016	1099042	42.48
	AT5G04060		Probable methyltransferase PMT7 [Source:UniProtKB/Swiss-Prot;Acc:Q9LZA4]	1099029	1102058	40.23
	AT5G04070		At5g04070 [Source:UniProtKB/TrEMBL;Acc:Q6NLR7]	1102071	1104446	37.29
	AT5G04080		At5g04080 [Source:UniProtKB/TrEMBL;Acc:Q6NN03]	1104524	1105398	36
	AT5G04090		Plant protein 1589 of unknown function [Source:TAIR;Acc:AT5G04090]	1105352	1108282	36.88
	AT5G04110	GYRB3	DNA GYRASE B3 [Source:TAIR;Acc:AT5G04110]	1110342	1115007	37.55
	AT5G04120	IPSP	Metal-independent phosphoserine phosphatase [Source:UniProtKB/Swiss-Prot;Acc:F4KI56]	1119937	1121813	35.59
	AT5G04130	GYRBM	DNA gyrase subunit B, mitochondrial [Source:UniProtKB/Swiss-Prot;Acc:Q94BZ7]	1121800	1128144	37.16
	AT5G04140	GLU1	glutamate synthase 1 [Source:TAIR;Acc:AT5G04140]	1129785	1138488	39.14
	AT5G04150	BHLH101	basic helix-loop-helix (bHLH) DNA-binding superfamily protein [Source:TAIR;Acc:AT5G04150]	1138410	1139633	37.83
	AT5G04160	UUAT1	UDP-URONIC ACID TRANSPORTER 1 [Source:UniProtKB/Swiss-Prot;Acc:Q9FYE5]	1142735	1145008	37.91
	AT5G04170	CML50	Probable calcium-binding protein CML50 [Source:UniProtKB/Swiss-Prot;Acc:Q9FYE4]	1145431	1147781	40.83
	AT5G04180	ACA3	Alpha carbonic anhydrase 3 [Source:UniProtKB/Swiss-Prot;Acc:Q9FYE3]	1147648	1149284	37.08
	AT5G04190	PKS4	Protein PHYTOCHROME KINASE SUBSTRATE 4 [Source:UniProtKB/Swiss-Prot;Acc:Q9FYE2]	1150886	1152627	42.42
	AT5G04200	AMC9	MCP2f [Source:UniProtKB/TrEMBL;Acc:A0A178U6S6]	1153740	1155040	43.66
	AT5G04210		CCCH-type zinc fingerfamily protein with RNA-binding domain-containing protein [Source:UniProtKB/TrEMBL;Acc:Q9FYE0]	1155049	1155724	43.79
	AT5G04220	SYT3	Synaptotagmin-3 [Source:UniProtKB/Swiss-Prot;Acc:Q7XA06]	1155725	1158944	38.39
	AT5G04230	PAL3	Phenylalanine ammonia-lyase [Source:UniProtKB/TrEMBL;Acc:F4JW69]	1160569	1163957	42.4
	AT5G04240	ELF6	ELF6 [Source:UniProtKB/TrEMBL;Acc:A0A178UAR7]	1169153	1174992	40.91
	AT5G04238		unknown protein; Ha. [Source:TAIR;Acc:AT5G04238]	1169217	1169411	44.1
	AT5G04250		At5g04250 [Source:UniProtKB/TrEMBL;Acc:Q8LBW2]	1176005	1179008	36.72
	AT5G04260	WCRKC2	Thioredoxin-like 3-2, chloroplastic [Source:UniProtKB/Swiss-Prot;Acc:Q8VZT6]	1178644	1180060	39.1
	AT5G04267		unknown protein; Ha. [Source:TAIR;Acc:AT5G04267]	1182025	1182294	42.22
	AT5G04270		DHHC-type zinc finger family protein [Source:TAIR;Acc:AT5G04270]	1182626	1184863	38.2
	AT5G04275	MIR172B	MIR172/MIR172B; miRNA [Source:TAIR;Acc:AT5G04275]	1188207	1188301	35.79
	AT5G04280	RZ1C	Glycine-rich RNA-binding protein RZ1C [Source:UniProtKB/Swiss-Prot;Acc:Q8RWN5]	1192259	1195759	40.82
	AT5G04290	RDM3	Protein RNA-directed DNA methylation 3 [Source:UniProtKB/Swiss-Prot;Acc:F4JW79]	1195969	1202936	42.65
	AT5G04310		Pectate lyase [Source:UniProtKB/TrEMBL;Acc:F4JW80]	1202967	1207946	37.17
	AT5G04320	SGO2	SHUGOSHIN 2 [Source:UniProtKB/Swiss-Prot;Acc:Q0WTB8]	1209197	1212578	37.55
	AT5G04330	CYP84A4	Cytochrome P450 84A4 [Source:UniProtKB/Swiss-Prot;Acc:F4JW83]	1212532	1214439	46.23
	AT5G04340	ZAT6	Zinc finger protein ZAT6 [Source:UniProtKB/Swiss-Prot;Acc:O22533]	1216058	1217362	42.15
	AT5G04347		Plant self-incompatibility protein S1 family [Source:TAIR;Acc:AT5G04347]	1219777	1220612	34.69
	AT5G04350		Plant self-incompatibility protein S1 family [Source:UniProtKB/TrEMBL;Acc:F4JW86]	1220829	1221269	40.14
	AT5G04360	PU1	Pullulanase 1, chloroplastic [Source:UniProtKB/Swiss-Prot;Acc:Q8GTR4]	1221448	1228841	37.42
	AT5G00950			1228957	1229271	39.37
	AT5G00955			1229899	1230504	41.75
	AT5G00960			1230181	1230463	45.58
	AT5G04370	NAMT1	S-adenosyl-L-methionine-dependent methyltransferases superfamily protein [Source:UniProtKB/TrEMBL;Acc:Q0WL44]	1231609	1234147	33.4
	AT5G04380		S-adenosyl-L-methionine-dependent methyltransferases superfamily protein [Source:TAIR;Acc:AT5G04380]	1234712	1236391	35.36
	AT5G04390		C2H2-type zinc finger family protein [Source:UniProtKB/TrEMBL;Acc:Q84K31]	1238820	1240517	41.34
	AT5G04395		NAC transcription factor-like protein [Source:UniProtKB/TrEMBL;Acc:A0A1P8BGF2]	1241555	1242374	44.15
	AT5G04400	anac077	NAC domain containing protein 77 [Source:TAIR;Acc:AT5G04400]	1242500	1243358	41.33
	AT5G04410	NAC078	NAC domain-containing protein 78 [Source:UniProtKB/Swiss-Prot;Acc:Q84K00]	1243684	1246684	39.75
	AT5G00965			1244826	1245200	36.8
	AT5G04420		AT5G04420 protein [Source:UniProtKB/TrEMBL;Acc:Q9LZ83]	1246604	1249943	39.58
	AT5G04430	BTR1	binding to TOMV RNA 1L (long form) [Source:TAIR;Acc:AT5G04430]	1250248	1253953	37.83
	AT5G04440		At5g04440 [Source:UniProtKB/TrEMBL;Acc:Q500X2]	1254757	1257040	40.11
	AT5G04460		RING/U-box superfamily protein [Source:UniProtKB/TrEMBL;Acc:F4JWA1]	1259435	1264025	40.38
	AT5G04470	SIM	Cyclin-dependent protein kinase inhibitor SIM [Source:UniProtKB/Swiss-Prot;Acc:Q9LZ78]	1266625	1267416	40.66
	AT5G04475			1267020	1267357	45.86
	AT5G04480		AT5g04480/T32M21_80 [Source:UniProtKB/TrEMBL;Acc:Q940Y7]	1271505	1278102	39.3
	AT5G04490	VTE5	Phytol kinase 1, chloroplastic [Source:UniProtKB/Swiss-Prot;Acc:Q9LZ76]	1279577	1281746	39.08
	AT5G04500		Glycosyltransferase family protein 64 protein C5 [Source:UniProtKB/Swiss-Prot;Acc:Q84WB7]	1283378	1286374	40.71
	AT5G04510	PDPK1	3-phosphoinositide-dependent protein kinase 1 [Source:UniProtKB/Swiss-Prot;Acc:Q9XF67]	1286843	1289908	39.69
	AT5G04520		3-oxoacyl-[acyl-carrier-protein] synthase-like protein [Source:UniProtKB/TrEMBL;Acc:Q9LZ73]	1289885	1291104	44.02
	AT5G04530	KCS19	3-ketoacyl-CoA synthase 19 [Source:UniProtKB/Swiss-Prot;Acc:Q9LZ72]	1291630	1293723	41.02
	AT5G00970			1294133	1294339	40.1
	AT5G04540	MTM2	Phosphatidylinositol-3-phosphatase myotubularin-2 [Source:UniProtKB/Swiss-Prot;Acc:F4JWB3]	1296356	1302477	38.57
	AT5G04550		AT5g04550/T32M21_140 [Source:UniProtKB/TrEMBL;Acc:Q9LZ71]	1303167	1306145	41.99
	AT5G04560	DME	Transcriptional activator DEMETER [Source:UniProtKB/Swiss-Prot;Acc:Q8LK56]	1309099	1318520	40.66
MQTL-2/Chr5	AT5G10960	CAF1-10	Probable CCR4-associated factor 1 homolog 10 [Source:UniProtKB/Swiss-Prot;Acc:Q9LEU4]	3464098	3466314	38.93
	AT5G10965			3467492	3472089	36.6
	AT5G10970		C2H2 and C2HC zinc fingers superfamily protein [Source:UniProtKB/TrEMBL;Acc:Q9LEU3]	3469268	3470086	44.57
	AT5G10980	HTR8	Histone H3.3 [Source:UniProtKB/Swiss-Prot;Acc:P59169]	3472405	3473570	38.85
	AT5G10990		At5g10990 [Source:UniProtKB/TrEMBL;Acc:Q9LEU2]	3476820	3477723	42.92
	AT5G11000		AT5g11000/T30N20_270 [Source:UniProtKB/TrEMBL;Acc:Q9LEU1]	3478672	3480621	39.38
	AT5G11010		Polynucleotide 5'-hydroxyl-kinase NOL9 [Source:UniProtKB/Swiss-Prot;Acc:Q8VYP6]	3483632	3486267	36.76
	AT5G11020		Protein kinase superfamily protein [Source:UniProtKB/TrEMBL;Acc:F4JWD4]	3486242	3489316	36.1
	AT5G11030	ALF4	Aberrant root formation protein 4 [Source:UniProtKB/Swiss-Prot;Acc:Q84VX3]	3490788	3495173	37.14
	AT5G11040	TRS120	Trafficking protein particle complex II-specific subunit 120 homolog [Source:UniProtKB/Swiss-Prot;Acc:Q9FY61]	3495195	3500945	41.87
	AT5G11050	MYB64	Transcription factor MYB64 [Source:UniProtKB/Swiss-Prot;Acc:Q9FY60]	3502092	3503834	37.98
	AT5G11060	KNAT4	KNAT4 [Source:UniProtKB/TrEMBL;Acc:A0A178UC59]	3509833	3513741	36.61
	AT5G11070		Uncharacterized protein At5g11070 [Source:UniProtKB/TrEMBL;Acc:Q9FY58]	3516092	3517035	38.56
	AT5G11080		Ubiquitin-like superfamily protein [Source:UniProtKB/TrEMBL;Acc:F4JWE4]	3517223	3520003	36.57
	AT5G01845			3522144	3522167	54.17
	AT5G11090		At5g11090 [Source:UniProtKB/TrEMBL;Acc:Q9FY56]	3524677	3525846	41.97
	AT5G11100	SYTD	Calcium-dependent lipid-binding (CaLB domain) family protein [Source:TAIR;Acc:AT5G11100]	3532402	3535221	36.74
	AT5G11110	SPS2	Glycosyltransferase (Fragment) [Source:UniProtKB/TrEMBL;Acc:W8QNB0]	3536189	3541149	40.96
	AT5G11130		Probable glycosyltransferase At5g11130 [Source:UniProtKB/Swiss-Prot;Acc:Q9LFP3]	3542139	3544780	36.87
	AT5G11140		Phospholipase-like protein (PEARLI 4) family protein [Source:UniProtKB/TrEMBL;Acc:Q9LFP2]	3545211	3546169	40.56
	AT5G11150	ATVAMP713	vesicle-associated membrane protein 713 [Source:TAIR;Acc:AT5G11150]	3546200	3548117	36.81
	AT5G11160	APT5	Adenine phosphoribosyltransferase 5 [Source:UniProtKB/Swiss-Prot;Acc:Q9LFP0]	3550300	3552255	37.37
	AT5G11170	RH15	DEAD-box ATP-dependent RNA helicase 56 [Source:UniProtKB/Swiss-Prot;Acc:Q9LFN6]	3553096	3557018	39.15
	AT5G11180	GLR2.6	Glutamate receptor 2.6 [Source:UniProtKB/Swiss-Prot;Acc:Q9LFN8]	3557238	3561575	37.21
	AT5G01855			3564583	3564925	35.86
	AT5G11190	SHN2	Ethylene-responsive transcription factor SHINE 2 [Source:UniProtKB/Swiss-Prot;Acc:Q9LFN7]	3564824	3566260	35.28
	AT5G11200		DEAD/DEAH box RNA helicase family protein [Source:UniProtKB/TrEMBL;Acc:F4JWF6]	3567174	3571039	38.85
	AT5G11210	GLR2.5	Glutamate receptor 2.5 [Source:UniProtKB/Swiss-Prot;Acc:Q9LFN5]	3571020	3574817	38.68
	AT5G01865			3574666	3574970	33.77
	AT5G11220		Uncharacterized protein F2I11_110 [Source:UniProtKB/TrEMBL;Acc:Q9LFN4]	3577057	3577978	38.61
	AT5G11225		pre-tRNA [Source:TAIR;Acc:AT5G11225]	3578295	3578366	55.56
	AT5G11230		Probable sugar phosphate/phosphate translocator At5g11230 [Source:UniProtKB/Swiss-Prot;Acc:Q9LFN3]	3580396	3582090	40.18
	AT5G11240		At5g11240 [Source:UniProtKB/TrEMBL;Acc:B5X503]	3582506	3587076	37.58
	AT5G11250		Disease resistance protein (TIR-NBS-LRR class) [Source:UniProtKB/TrEMBL;Acc:Q9LFN1]	3587978	3593271	39.25
	AT5G11260	HY5	Basic-leucine zipper (bZIP) transcription factor family protein [Source:TAIR;Acc:AT5G11260]	3593335	3595073	38.3
	AT5G11270	OCP3	OCP3 [Source:UniProtKB/TrEMBL;Acc:A0A178UCN6]	3595274	3597237	39.15
	AT5G11280		Tail fiber [Source:UniProtKB/TrEMBL;Acc:Q94AQ7]	3597216	3598687	39.74
	AT5G11290		Transmembrane protein, putative (DUF247) [Source:UniProtKB/TrEMBL;Acc:Q9LFM8]	3598926	3600549	40.76
	AT5G11300	CYCA2-2	Cyclin-A2-2 [Source:UniProtKB/Swiss-Prot;Acc:Q147G5]	3601513	3605350	37.94

AT5G11310		Pentatricopeptide repeat-containing protein At5g11310, mitochondrial [Source:UniProtKB/Swiss-Prot;Acc:Q9LFM6]	3606385	3608628	41.67
AT5G11320	YUC4	YUC4 [Source:UniProtKB/TrEMBL;Acc:A0A178UJ31]	3611247	3613640	38.68
AT5G11325		pre-tRNA [Source:TAIR;Acc:AT5G11325]	3617058	3617129	55.56
AT5G11330		At5g11330 [Source:UniProtKB/TrEMBL;Acc:Q9LFM4]	3617138	3618913	39.02
AT5G11340		Acyl-CoA N-acyltransferases (NAT) superfamily protein [Source:UniProtKB/TrEMBL;Acc:Q9LFM3]	3619152	3621511	36.69
AT5G11350		DNAse I-like superfamily protein [Source:TAIR;Acc:AT5G11350]	3621454	3625645	40.55
AT5G11360		Interleukin-1 receptor-associated kinase 4 protein [Source:UniProtKB/TrEMBL;Acc:F4JWH8]	3625570	3626735	36.79
AT5G11370		FBD / Leucine Rich Repeat domains containing protein [Source:UniProtKB/TrEMBL;Acc:Q9LFM0]	3627849	3629717	35.21
AT5G11380	DXPS3	1-D-deoxyxylulose 5-phosphate synthase-like protein [Source:UniProtKB/TrEMBL;Acc:Q9LFL9]	3629988	3633511	39.95
AT5G11390	WIT1	WPP domain-interacting tail-anchored protein 1 [Source:UniProtKB/Swiss-Prot;Acc:Q8L7E5]	3633649	3636934	38.53
AT5G11400		Protein kinase superfamily protein [Source:TAIR;Acc:AT5G11400]	3636614	3638059	38.45
AT5G11410		Protein kinase superfamily protein [Source:UniProtKB/TrEMBL;Acc:Q9LFL6]	3638191	3639965	34.7
AT5G11412		RNA-binding (RRM/RBD/RNP motifs) family protein [Source:UniProtKB/TrEMBL;Acc:Q1G378]	3640026	3640876	38.07
AT5G01875			3641586	3641908	30.96
AT5G11416		unknown protein; Ha. [Source:TAIR;Acc:AT5G11416]	3642336	3643010	40.3
AT5G11420		Uncharacterized protein At5g11420 [Source:UniProtKB/TrEMBL;Acc:Q9LYE7]	3644547	3647411	38.92
AT5G11430		SPOC domain / Transcription elongation factor S-II protein [Source:UniProtKB/TrEMBL;Acc:F4JXV3]	3647862	3652477	40.29
AT5G11425		unknown protein; Ha. [Source:TAIR;Acc:AT5G11425]	3647959	3648168	44.29
AT5G11440	CID5	Polyadenylate-binding protein-interacting protein 5 [Source:UniProtKB/Swiss-Prot;Acc:Q9LYE5]	3652714	3654202	36
AT5G11450		PPD5 [Source:UniProtKB/TrEMBL;Acc:A0A384KVS7]	3654392	3656647	38.08
AT5G11460	FLZ10	FCS-Like Zinc finger 10 [Source:UniProtKB/Swiss-Prot;Acc:Q9LYE4]	3656582	3659025	39.44
AT5G11470		bromo-adjacent homology (BAH) domain-containing protein [Source:TAIR;Acc:AT5G11470]	3662634	3668214	37.07
AT5G11475		pre-tRNA [Source:TAIR;Acc:AT5G11475]	3669000	3669072	58.9
AT5G11480		AT5g11480/F15N18_70 [Source:UniProtKB/TrEMBL;Acc:Q9LYE2]	3669193	3671684	39.93
AT5G11490		Beta-adaptin-like protein [Source:UniProtKB/TrEMBL;Acc:F4JXV9]	3671699	3676452	39.55
AT5G11500		Coiled-coil protein [Source:UniProtKB/TrEMBL;Acc:Q9LYE1]	3676548	3679738	37.01
AT5G11510	MYB3R4	Transcription factor MYB3R-4 [Source:UniProtKB/Swiss-Prot;Acc:Q94FL9]	3679748	3684884	38.41
AT5G11520	ASP3	Aspartate aminotransferase 3, chloroplastic [Source:UniProtKB/Swiss-Prot;Acc:P46644]	3684946	3687854	38.81
AT5G11530	EMF1	Protein EMBRYONIC FLOWER 1 [Source:UniProtKB/Swiss-Prot;Acc:Q9LYD9]	3695792	3701549	39.34
AT5G11540	GULLO3	L-gulonolactone oxidase 3 [Source:UniProtKB/Swiss-Prot;Acc:Q9LYD8]	3702700	3705129	41.23
AT5G11550		ARM repeat superfamily protein [Source:UniProtKB/TrEMBL;Acc:Q9LYD7]	3707961	3709403	50.17
AT5G11560		Catalytics [Source:UniProtKB/TrEMBL;Acc:F4JXW9]	3709442	3714124	40.17
AT5G11565		Reverse transcriptase zinc-binding protein [Source:UniProtKB/TrEMBL;Acc:A0A1P8BDW1]	3714655	3714912	41.86
AT5G11570	NPF1.3	Protein NRT1/ PTR FAMILY 1.3 [Source:UniProtKB/Swiss-Prot;Acc:Q9LYD5]	3715931	3718276	39.56
AT5G11580		AT5g11580/F15N18_170 [Source:UniProtKB/TrEMBL;Acc:Q93Z10]	3718804	3721425	41.08
AT5G11590	DREB3	Dehydration-responsive element-binding protein 3 [Source:UniProtKB/Swiss-Prot;Acc:Q9LYD3]	3727453	3728726	41.92
AT5G11600		At5g11600 [Source:UniProtKB/TrEMBL;Acc:Q9LYD2]	3733635	3735265	38.01
AT5G11610		Exostosin family protein [Source:UniProtKB/TrEMBL;Acc:Q9LYD1]	3735350	3738230	36.2
AT5G11620		PHD family protein [Source:UniProtKB/TrEMBL;Acc:Q9LYG8]	3738729	3740164	41.16
AT5G11630		Uncharacterized protein T22P22_20 [Source:UniProtKB/TrEMBL;Acc:Q9LYG7]	3740460	3741827	37.5
AT5G11640		At5g11640 [Source:UniProtKB/TrEMBL;Acc:Q9LYG6]	3741782	3743994	37.14
AT5G11650		Alpha/beta-Hydrolases superfamily protein [Source:UniProtKB/TrEMBL;Acc:Q9LYG5]	3744698	3747198	40.74
AT5G11660		NEP-interacting protein, putative (DUF239) [Source:UniProtKB/TrEMBL;Acc:Q9LYG4]	3748880	3750317	31.78
AT5G11670	NADP-ME2	NADP-dependent malic enzyme 2 [Source:UniProtKB/Swiss-Prot;Acc:Q9LYG3]	3754091	3758342	39.23
AT5G11680		Classical AGP protein [Source:UniProtKB/TrEMBL;Acc:Q9LYG2]	3758809	3760913	37.91
AT5G11690	TIM17-3	Mitochondrial import inner membrane translocase subunit TIM17-3 [Source:UniProtKB/Swiss-Prot;Acc:Q9LYG1]	3761188	3762346	40.47
AT5G11700		LOCATED IN: vacuole; EXPRESSED IN: 24 plant structures; EXPRESSED DURING: 13 growth stages; BEST Arabidopsis thaliana protein ma	3762391	3771449	39.6
AT5G11710	EPSIN1	Clathrin interactor EPSIN 1 [Source:UniProtKB/Swiss-Prot;Acc:Q8VY07]	3772401	3776671	37.95
AT5G11720		AT5g11720/T22P22_110 [Source:UniProtKB/TrEMBL;Acc:Q9LYF8]	3776723	3780255	42
AT5G11730		At5g11730 [Source:UniProtKB/TrEMBL;Acc:Q9LYF7]	3780302	3782793	40.93
AT5G11740	AGP15	ATAGP15 [Source:UniProtKB/TrEMBL;Acc:C0SVP3]	3783930	3784963	39.26
AT5G01885			3786071	3786453	38.12
AT5G11750		Ribosomal protein L19 family protein [Source:TAIR;Acc:AT5G11750]	3787230	3789212	37.22
AT5G11760		AT5g11760/T22P22_150 [Source:UniProtKB/TrEMBL;Acc:Q9LYF4]	3789259	3790573	40.08
AT5G11770		NADH dehydrogenase [ubiquinone] iron-sulfur protein 7, mitochondrial [Source:UniProtKB/Swiss-Prot;Acc:Q42577]	3790708	3793026	41.05
AT5G11780		SNF2 domain protein [Source:UniProtKB/TrEMBL;Acc:Q56Y63]	3793830	3796202	39.82
AT5G11790	NDL2	Protein NDL2 [Source:UniProtKB/Swiss-Prot;Acc:Q9ASU8]	3799408	3803216	35.36
AT5G01915			3802714	3803062	28.65
AT5G11800	KEA6	K(+) efflux antiporter 6 [Source:UniProtKB/Swiss-Prot;Acc:B5X0N6]	3803315	3808273	37.81
AT5G11810		At5g11810 [Source:UniProtKB/TrEMBL;Acc:Q9LYF0]	3808678	3810748	38.1
AT5G11820		Plant self-incompatibility protein S1 family [Source:TAIR;Acc:AT5G11820]	3810649	3811280	45.09
AT5G11830		Plant self-incompatibility protein S1 family [Source:UniProtKB/TrEMBL;Acc:Q9LYE8]	3812520	3813044	41.9
AT5G11840		At5g11840 [Source:UniProtKB/TrEMBL;Acc:B6IDH6]	3813302	3814906	40.69
AT5G11850		AT5g11850/F14F18_20 [Source:UniProtKB/TrEMBL;Acc:Q8S9K4]	3816080	3821170	39.13
AT5G11860	SSP5	At5g11860 [Source:UniProtKB/TrEMBL;Acc:Q9LYI7]	3821733	3824298	37.76
AT5G11870		Alkaline phytoceramidase (APHC) [Source:UniProtKB/TrEMBL;Acc:F4JZD5]	3825420	3827573	37.56
AT5G11880	LYSA2	Diaminopimelate decarboxylase 2, chloroplastic [Source:UniProtKB/Swiss-Prot;Acc:Q94A94]	3827526	3830228	38.92
AT5G11890		EMB3135 [Source:UniProtKB/TrEMBL;Acc:A0A178UG53]	3831500	3833151	40.25
AT5G11900		Translation machinery-associated protein 22 [Source:UniProtKB/TrEMBL;Acc:Q8VZK2]	3833919	3836359	38.39
AT5G11910		alpha/beta-Hydrolases superfamily protein [Source:TAIR;Acc:AT5G11910]	3836300	3838243	37.65
AT5G11920	CWINV6	Beta-fructofuranosidase, insoluble isoenzyme CWINV6 [Source:UniProtKB/Swiss-Prot;Acc:Q8W4S6]	3839316	3842409	35.84
AT5G11930	GRXC10	Glutaredoxin-C10 [Source:UniProtKB/Swiss-Prot;Acc:Q29PZ1]	3844940	3845711	43.26
AT5G11940	SBT3.11	Subtilisin-like protease SBT3.11 [Source:UniProtKB/Swiss-Prot;Acc:Q1PDX5]	3849283	3852417	41.05
AT5G11950	LOG8	Cytokinin riboside 5'-monophosphate phosphoribohydrolase [Source:UniProtKB/TrEMBL;Acc:A0A178UJ28]	3854388	3857243	35.92
AT5G11960		Probable magnesium transporter NIPA9 [Source:UniProtKB/Swiss-Prot;Acc:Q8RWH8]	3858428	3861528	38.25
AT5G11970		ABC family ABC transporter, putative (DUF3511) [Source:UniProtKB/TrEMBL;Acc:Q9LYH6]	3862849	3863824	36.68
AT5G11975			3863277	3863671	45.32
AT5G11977	MIR156E	MIR156E; miRNA [Source:TAIR;Acc:AT5G11977]	3867207	3867313	45.79
AT5G11980		Conserved oligomeric Golgi complex subunit 8 [Source:UniProtKB/TrEMBL;Acc:Q84K25]	3868652	3872300	39.76
AT5G11990		Proline-rich family protein [Source:UniProtKB/TrEMBL;Acc:Q9LYH4]	3872563	3873349	42.82
AT5G12000		Kinase with adenine nucleotide alpha hydrolases-like domain-containing protein [Source:UniProtKB/TrEMBL;Acc:Q0WVJ0]	3873799	3877004	39.58
AT5G12010		AT5g12010/F14F18_180 [Source:UniProtKB/TrEMBL;Acc:Q9LYH2]	3877680	3879657	42.62
AT5G12020	HSP17.6	17.6 kDa class II heat shock protein [Source:UniProtKB/Swiss-Prot;Acc:P29830]	3882231	3883111	40.75
AT5G12030	HSP17.7	17.7 kDa class II heat shock protein [Source:UniProtKB/Swiss-Prot;Acc:O81822]	3883957	3884838	39.91
AT5G12040	NLP3	Omega-amidase, chloroplastic [Source:UniProtKB/Swiss-Prot;Acc:Q8RUF8]	3884949	3888077	37.39
AT5G12043		unknown protein; Ha. [Source:TAIR;Acc:AT5G12043]	3888549	3888894	49.13
AT5G01925			3889361	3889583	34.53
AT5G12050		Protein BIG GRAIN 1-like D [Source:UniProtKB/Swiss-Prot;Acc:Q9LYH0]	3889906	3891514	37.79
AT5G12060	SPH3	S-protein homolog 3 [Source:UniProtKB/Swiss-Prot;Acc:Q9FMQ4]	3894773	3895393	35.1
AT5G12070	SPH4	S-protein homolog 4 [Source:UniProtKB/Swiss-Prot;Acc:F4JZG1]	3896950	3897408	39.43
AT5G12080	MSL10	Mechanosensitive ion channel protein 10 [Source:UniProtKB/Swiss-Prot;Acc:Q9LYG9]	3897893	3901790	38.61
AT5G12090		Protein kinase superfamily protein [Source:UniProtKB/TrEMBL;Acc:Q9FMQ2]	3909703	3910877	41.79
AT5G12100		Pentatricopeptide repeat-containing protein At5g12100, mitochondrial [Source:UniProtKB/Swiss-Prot;Acc:Q9FMQ1]	3911334	3914167	41.64
AT5G12110		At5g12110 [Source:UniProtKB/TrEMBL;Acc:Q29PY2]	3914468	3916896	41.66
AT5G12120		AT5g12120/MXC9_8 [Source:UniProtKB/TrEMBL;Acc:Q9FMQ0]	3915676	3918933	41.62
AT5G12130	TERC	Thylakoid membrane protein TERC, chloroplastic [Source:UniProtKB/Swiss-Prot;Acc:F4JZG9]	3919428	3922485	37.9
AT5G12140	CYS1	Cysteine proteinase inhibitor [Source:UniProtKB/TrEMBL;Acc:A0A178UCE6]	3922591	3924058	38.15
AT5G12150	ROPGAP6	Rho GTPase-activating protein 6 [Source:UniProtKB/Swiss-Prot;Acc:Q9FMP8]	3924107	3930441	38.28
AT5G12170	CLT3	Protein CLT3, chloroplastic [Source:UniProtKB/Swiss-Prot;Acc:Q8RWL5]	3931349	3935142	36.56
AT5G12180	CPK17	CPK17 [Source:UniProtKB/TrEMBL;Acc:A0A178URQ6]	3936813	3939596	40.7
AT5G12190		Splicing factor 3B subunit 6-like protein [Source:UniProtKB/Swiss-Prot;Acc:Q9FMP4]	3940481	3941436	41
AT5G12200	PYD2	Dihydropyrimidinase [Source:UniProtKB/Swiss-Prot;Acc:Q9FMP3]	3941528	3944866	38.1
AT5G12210	RGTB1	Geranylgeranyl transferase type-2 subunit beta 1 [Source:UniProtKB/Swiss-Prot;Acc:Q84J75]	3947022	3949866	37.72
AT5G12220		Las1-like family protein [Source:UniProtKB/TrEMBL;Acc:F4JZH9]	3950133	3953150	39.76
AT5G12230	MED19A	Mediator of RNA polymerase II transcription subunit 19a [Source:UniProtKB/Swiss-Prot;Acc:Q9FMP0]	3953140	3956185	36.34

AT5G12235	CLE22	CLAVATA3/ESR (CLE)-related protein 22 [Source:UniProtKB/Swiss-Prot;Acc:Q3E9I4]	3957521	3958129	33.66
AT5G12236		unknown protein; LOCATED IN: endomembrane system; Ha. [Source:TAIR;Acc:AT5G12236]	3958148	3958282	34.81
AT5G12240		At5g12240 [Source:UniProtKB/TrEMBL;Acc:Q6NQD2]	3958861	3961020	34.95
AT5G12250	TUBB6	Tubulin beta chain [Source:UniProtKB/TrEMBL;Acc:A0A178UPR2]	3960938	3963624	41.61
AT5G12260		AT5g12260/MXC9_22 [Source:UniProtKB/TrEMBL;Acc:Q9FMN8]	3966321	3968937	41.15
AT5G12270		1-AMINOCYCLOPROPANE-1-CARBOXYLATE OXIDASE-like protein [Source:UniProtKB/TrEMBL;Acc:Q94CL5]	3969925	3971452	40.58
AT5G12280		SWAP (Suppressor-of-White-APricot)/surp RNA-binding domain-containing protein [Source:UniProtKB/TrEMBL;Acc:Q94CL4]	3971715	3973800	41.28
AT5G12290	DGS1	DGS1 [Source:UniProtKB/TrEMBL;Acc:A0A178UP20]	3974119	3978221	38.05
AT5G12300		At5g12300 [Source:UniProtKB/TrEMBL;Acc:Q94CL2]	3978281	3980021	37.85
AT5G12310		AT5g19430/F7K24_180 [Source:UniProtKB/TrEMBL;Acc:Q94CL1]	3980179	3982528	39.19
AT5G12320		Ankyrin repeat family protein [Source:UniProtKB/TrEMBL;Acc:Q8GWI1]	3982577	3984112	37.76
AT5G12330	LRP1	Lateral root primordium (LRP) protein-related [Source:TAIR;Acc:AT5G12330]	3987375	3989876	41.89
AT5G12340		DUF4228 domain protein [Source:UniProtKB/TrEMBL;Acc:Q1PDX4]	3992623	3993767	36.68
AT5G12350		Regulator of chromosome condensation (RCC1) family with FYVE zinc finger domain-containing protein [Source:UniProtKB/TrEMBL;Acc:F4K	3995310	4000801	40.42
AT5G12360	PANS2	Protein PATRONUS 2 [Source:UniProtKB/Swiss-Prot;Acc:Q94CK6]	4000699	4002742	37.62
AT5G12370	SEC10a	Exocyst complex component SEC10a [Source:UniProtKB/Swiss-Prot;Acc:X5JA13]	4002742	4008804	38.33
AT5G01935			4008968	4010413	39.42
AT5G12380	ANNAT8	Annexin [Source:UniProtKB/TrEMBL;Acc:X5JAK8]	4009168	4010687	40.59
AT5G12390	FIS1B	Mitochondrial fission 1 protein [Source:UniProtKB/TrEMBL;Acc:A0A178U9A0]	4010443	4012134	39.78
AT5G01945			4011615	4011841	36.56
AT5G12400		PHD-finger and DNA binding domain-containing protein [Source:UniProtKB/TrEMBL;Acc:F4K0Y2]	4013424	4021591	39.88
AT5G12410		At5g12410/At5g12410 [Source:UniProtKB/TrEMBL;Acc:Q94CK1]	4021815	4024218	38.27
AT5G12420		O-acyltransferase (WSD1-like) family protein [Source:UniProtKB/TrEMBL;Acc:Q94CK0]	4024286	4026849	36.27
AT5G12430	TPR16	Heat shock protein DnaJ with tetratricopeptide repeat-containing protein [Source:UniProtKB/TrEMBL;Acc:F4K0Y5]	4027974	4034682	38.31
AT5G12440		CCCH-type zinc fingerfamily protein with RNA-binding domain [Source:TAIR;Acc:AT5G12440]	4035181	4042254	36.91
AT5G12450		FBD-like domain family protein [Source:UniProtKB/TrEMBL;Acc:Q94CJ7]	4040937	4041176	39.58
AT5G12460		Protein of unknown function (DUF604) [Source:TAIR;Acc:AT5G12460]	4043057	4044583	42.17
AT5G12470	RER4	Protein RETICULATA-RELATED 4, chloroplastic [Source:UniProtKB/Swiss-Prot;Acc:Q94CJ5]	4044620	4047448	40.65
AT5G12480	CPK7	CPK7 [Source:UniProtKB/TrEMBL;Acc:A0A178UIX4]	4047513	4050643	38.68
AT5G12840	NFYA1	Nuclear transcription factor Y subunit A-1 [Source:UniProtKB/Swiss-Prot;Acc:Q9LXV5]	4050691	4053669	38.44
AT5G12850		Zinc finger CCCH domain-containing protein 56 [Source:UniProtKB/Swiss-Prot;Acc:Q9LXV4]	4056086	4059580	41.46
AT5G12860	DIT1	Dicarboxylate transporter 1, chloroplastic [Source:UniProtKB/Swiss-Prot;Acc:Q9LXV3]	4059579	4062289	45.11
AT5G12870	MYB46	Transcription factor MYB46 [Source:UniProtKB/Swiss-Prot;Acc:Q9LXV2]	4062666	4065075	34.44
AT5G12880		At5g12880 [Source:UniProtKB/TrEMBL;Acc:Q9LXV1]	4068426	4069094	35.58
AT5G12890	UGT92A1	Glycosyltransferase (Fragment) [Source:UniProtKB/TrEMBL;Acc:W8Q2U4]	4069417	4071249	42.23
AT5G12900	IRKI	IRK-interacting protein [Source:UniProtKB/Swiss-Prot;Acc:Q9LXU9]	4071896	4074619	38.36
AT5G12910		Histone H3-like 4 [Source:UniProtKB/Swiss-Prot;Acc:Q9LXU8]	4077162	4077557	47.73
AT5G12920		Transducin/WD40 repeat-like superfamily protein [Source:UniProtKB/TrEMBL;Acc:F4K108]	4077458	4082634	36.58
AT5G12930		At5g12930 [Source:UniProtKB/TrEMBL;Acc:Q9LXU6]	4082890	4085376	39.77
AT5G12940		Leucine-rich repeat (LRR) family protein [Source:UniProtKB/TrEMBL;Acc:Q9LXU5]	4087689	4089146	44.03
AT5G12950		Proline-tRNA ligase (DUF1680) [Source:UniProtKB/TrEMBL;Acc:Q9LXU4]	4092519	4097059	39.24
AT5G12960		Proline-tRNA ligase (DUF1680) [Source:UniProtKB/TrEMBL;Acc:Q84W43]	4097693	4101766	40.18
AT5G12970		Anthranilate phosphoribosyltransferase-like protein [Source:UniProtKB/TrEMBL;Acc:Q9LXU2]	4102115	4105414	41.67
AT5G12980		Cell differentiation, Rcd1-like protein [Source:UniProtKB/TrEMBL;Acc:Q9LXU1]	4105383	4108232	38.46
AT5G12990	CLE40	CLAVATA3/ESR (CLE)-related protein 40 [Source:UniProtKB/Swiss-Prot;Acc:Q9LXU0]	4109234	4109784	33.21
AT5G13000	CALS3	Callose synthase 3 [Source:UniProtKB/Swiss-Prot;Acc:Q9LXT9]	4110171	4121489	38.48
AT5G13010	CUV	Pre-mRNA-splicing factor ATP-dependent RNA helicase DEAH7 [Source:UniProtKB/Swiss-Prot;Acc:F4K2E9]	4122400	4129005	40.04
AT5G13020	EML3	EML3 [Source:UniProtKB/TrEMBL;Acc:A0A178UIH0]	4129154	4133023	38.29
AT5G13030		Selenoprotein O [Source:UniProtKB/TrEMBL;Acc:Q8H126]	4133134	4136688	38.99
AT5G13050	5FCL	5-formyltetrahydrofolate cyclo-ligase, mitochondrial [Source:UniProtKB/Swiss-Prot;Acc:Q8L539]	4136556	4138792	38.49
AT5G13060	ABAP1	ARMADILLO BTB ARABIDOPSIS PROTEIN 1 [Source:UniProtKB/Swiss-Prot;Acc:B7U179]	4142910	4147140	38.08
AT5G13070		At5g13070 [Source:UniProtKB/TrEMBL;Acc:Q9FYA3]	4147596	4149391	38.47
AT5G13080	WRKY75	WRKY75 [Source:UniProtKB/TrEMBL;Acc:A0A178UJF4]	4149738	4151201	31.83
AT5G13090		Uncharacterized protein At5g13090 [Source:UniProtKB/TrEMBL;Acc:Q8H0Z9]	4153369	4154730	39.35
AT5G13100		AT5g13100/T19L5_60 [Source:UniProtKB/TrEMBL;Acc:Q9FYA0]	4156501	4158554	42.79
AT5G13110	G6PD2	Glucose-6-phosphate 1-dehydrogenase 2, chloroplastic [Source:UniProtKB/Swiss-Prot;Acc:Q9FY99]	4158639	4161845	38.14
AT5G13120	PNSL5	Peptidyl-prolyl cis-trans isomerase [Source:UniProtKB/TrEMBL;Acc:A0A178UN74]	4162374	4164923	39.14
AT5G13130	MORC5	Protein MICRORCHIDIA 5 [Source:UniProtKB/Swiss-Prot;Acc:F4K2G3]	4166609	4170643	36.83
AT5G13140		Pollen Ole e 1 allergen and extensin family protein [Source:UniProtKB/TrEMBL;Acc:Q9FY96]	4170560	4171954	41.15
AT5G01955			4171915	4172122	32.21
AT5G13150	ATEXO70C1	Exocyst subunit Exo70 family protein [Source:UniProtKB/TrEMBL;Acc:Q9FY95]	4172734	4175362	41.61
AT5G13160	PBS1	PBS1 [Source:UniProtKB/TrEMBL;Acc:A0A384K8Z2]	4176506	4180009	38.93
AT5G13170	SWEET15	Bidirectional sugar transporter SWEET15 [Source:UniProtKB/Swiss-Prot;Acc:Q9FY94]	4181042	4183354	35.28
AT5G01965			4190250	4190488	31.38
AT5G13180	NAC083	NAC domain-containing protein 83 [Source:UniProtKB/Swiss-Prot;Acc:Q9FY93]	4196256	4197940	35.43
AT5G13181		unknown protein; Ha. [Source:TAIR;Acc:AT5G13181]	4198752	4199513	30.84
AT5G01975			4200093	4200305	35.21
AT5G01985			4201625	4201856	33.19
AT5G01995			4202200	4202509	30.65
AT5G02015			4202662	4202928	29.96
AT5G13190		CONTAINS InterPro DOMAIN/s: LPS-induced tumor necrosis factor alpha factor (InterPro:IPR006629); Ha. [Source:TAIR;Acc:AT5G13190]	4204085	4206475	38.73
AT5G13200		GEM-like protein 5 [Source:UniProtKB/Swiss-Prot;Acc:Q9LYV6]	4206738	4208289	40.66
AT5G13210		AT5g13210/T31B5_30 [Source:UniProtKB/TrEMBL;Acc:Q9LYV5]	4213955	4216908	41.4
AT5G02045			4218551	4218856	31.37
AT5G13220	JAZ10	jasmonate-zim-domain protein 10 [Source:TAIR;Acc:AT5G13220]	4218786	4221077	35.38
AT5G02055			4221882	4222321	34.77
AT5G13225		snoRNA [Source:TAIR;Acc:AT5G13225]	4222003	4222085	34.94
AT5G13230	PCMP-H89	Putative pentatricopeptide repeat-containing protein At5g13230, mitochondrial [Source:UniProtKB/Swiss-Prot;Acc:Q9LYV3]	4222512	4224982	42.49
AT5G13240		transcription regulators [Source:TAIR;Acc:AT5G13240]	4225005	4227717	37.27
AT5G13250		RING finger protein [Source:UniProtKB/TrEMBL;Acc:F4K2H9]	4234156	4241934	32.32
AT5G02075			4241003	4241471	34.33
AT5G13260		Myosin [Source:UniProtKB/TrEMBL;Acc:Q8VZL1]	4242896	4246882	38.85
AT5G13270	PCMP-H90	Pentatricopeptide repeat-containing protein At5g13270, chloroplastic [Source:UniProtKB/Swiss-Prot;Acc:Q9LYU9]	4246954	4249307	42.61
AT5G13280	AK1	AK1 [Source:UniProtKB/TrEMBL;Acc:A0A178UUC2]	4249312	4253264	39.36
AT5G13290	CRN	Inactive leucine-rich repeat receptor-like protein kinase CORYNE [Source:UniProtKB/Swiss-Prot;Acc:Q9LYU7]	4252842	4255378	40.13
AT5G13295		Transmembrane protein [Source:UniProtKB/TrEMBL;Acc:A0A1P8BF06]	4253953	4254321	35.77
AT5G13300	AGD3	ADP-ribosylation factor GTPase-activating protein AGD3 [Source:UniProtKB/Swiss-Prot;Acc:Q5W7F2]	4255547	4262568	38.78
AT5G13310		Uncharacterized protein At5g13310 [Source:UniProtKB/TrEMBL;Acc:Q9LYU5]	4263482	4265952	40.67
AT5G02085			4265635	4265909	37.82
AT5G13320	PBS3	Auxin-responsive GH3 family protein [Source:TAIR;Acc:AT5G13320]	4267456	4271064	36.35
AT5G13330	ERF113	Rap2.6L [Source:UniProtKB/TrEMBL;Acc:A0A178UKL8]	4271730	4274674	34.19
AT5G13340		Arginine/glutamate-rich 1 protein [Source:UniProtKB/TrEMBL;Acc:Q9LYU2]	4277546	4279493	38.81
AT5G13350		Auxin-responsive GH3 family protein [Source:UniProtKB/TrEMBL;Acc:Q9LYU1]	4279769	4282395	40.77
AT5G13360		Auxin-responsive GH3 family protein [Source:UniProtKB/TrEMBL;Acc:F4K2J2]	4283356	4286342	40.54
AT5G13370		At5g13370 [Source:UniProtKB/TrEMBL;Acc:Q8GZ29]	4286740	4289663	40.63
AT5G13380		Auxin-responsive GH3 family protein [Source:UniProtKB/TrEMBL;Acc:F4K2J6]	4289839	4292441	42.14
AT5G13390	NEF1	no exine formation 1 [Source:TAIR;Acc:AT5G13390]	4292704	4296725	44.53
AT5G13400	NPF6.1	Protein NRT1/ PTR FAMILY 6.1 [Source:UniProtKB/Swiss-Prot;Acc:Q9LYR6]	4296688	4299264	42.41
AT5G13410	FKBP19	Peptidyl-prolyl cis-trans isomerase FKBP19, chloroplastic [Source:UniProtKB/Swiss-Prot;Acc:Q9LYR5]	4299705	4301787	37.78
AT5G13420		Aldolase-type TIM barrel family protein [Source:UniProtKB/TrEMBL;Acc:Q9LYR4]	4301792	4304315	40.06
AT5G13430	UCR1-1	Cytochrome b-c1 complex subunit Rieske-1, mitochondrial [Source:UniProtKB/Swiss-Prot;Acc:Q94JS0]	4305106	4307570	38.82
AT5G13440	UCR1-2	Cytochrome b-c1 complex subunit Rieske-2, mitochondrial [Source:UniProtKB/Swiss-Prot;Acc:Q9LYR2]	4307973	4310273	38.9
AT5G13450	ATP5	ATP synthase subunit O, mitochondrial [Source:UniProtKB/Swiss-Prot;Acc:Q96251]	4310254	4312066	38.61
AT5G13460	IQD11	IQ-domain 11 [Source:UniProtKB/TrEMBL;Acc:Q9LYR0]	4315759	4318414	38.82
AT5G13470		At5g13470 [Source:UniProtKB/TrEMBL;Acc:Q9LYQ9]	4318460	4319924	38.43

AT5G13480	FY	Transducin/WD40 repeat-like superfamily protein [Source:TAIR;Acc:AT5G13480]	4326397	4331971	38.96
AT5G13490	AAC2	ADP,ATP carrier protein 2, mitochondrial [Source:UniProtKB/Swiss-Prot;Acc:P40941]	4335272	4337730	42.37
AT5G13500	HPAT3	Hydroxyproline O-arabinosyltransferase 3 [Source:UniProtKB/Swiss-Prot;Acc:Q9FY51]	4337894	4341088	37.97
AT5G02095			4341236	4341690	49.89
AT5G13510	RPL10	50S ribosomal protein L10, chloroplastic [Source:UniProtKB/Swiss-Prot;Acc:Q9FY50]	4341262	4342095	46.28
AT5G13520	LKHA4	Leucine aminopeptidase [Source:UniProtKB/Swiss-Prot;Acc:Q9FY49]	4341948	4344792	43.9
AT5G13530	KEG	E3 ubiquitin-protein ligase KEG [Source:UniProtKB/Swiss-Prot;Acc:Q9FY48]	4345202	4354723	40.6
AT5G13550	SULTR4;1	Sulfate transporter 4.1, chloroplastic [Source:UniProtKB/Swiss-Prot;Acc:Q9FY46]	4355276	4359692	38.8
AT5G13560		At5g13560 [Source:UniProtKB/TrEMBL;Acc:Q8GY46]	4360366	4366501	38.2
AT5G13565			4361047	4361171	40.8
AT5G13570	DCP2	decapping 2 [Source:TAIR;Acc:AT5G13570]	4367262	4370429	38.54
AT5G13580	ABCG6	ABC transporter G family member 6 [Source:UniProtKB/Swiss-Prot;Acc:Q9FNB5]	4370692	4373201	46.41
AT5G13590		unknown protein; Ha. [Source:TAIR;Acc:AT5G13590]	4373239	4379401	39.67
AT5G13600		Putative BTB/POZ domain-containing protein At5g13600 [Source:UniProtKB/Swiss-Prot;Acc:Q9FNB3]	4380432	4382497	41.82
AT5G13610		AT5g13610/MSH12_7 [Source:UniProtKB/TrEMBL;Acc:Q9FNB2]	4382975	4385099	39.11
AT5G13620		Gb [Source:UniProtKB/TrEMBL;Acc:Q9FNB1]	4385385	4386386	43.01
AT5G13630	CHLH	Magnesium-chelatase subunit ChlH, chloroplastic [Source:UniProtKB/Swiss-Prot;Acc:Q9FNB0]	4387307	4392429	45.17
AT5G13640	PDAT1	Phospholipid:diacylglycerol acyltransferase 1 [Source:UniProtKB/Swiss-Prot;Acc:Q9FNA9]	4392937	4397541	40.48
AT5G13650		Elongation factor family protein [Source:UniProtKB/TrEMBL;Acc:F4K409]	4397578	4402692	38.89
AT5G13660		unknown protein; BEST Arabidopsis thaliana protein match is: unknown protein (TAIR:AT5G59830.2); Ha. [Source:TAIR;Acc:AT5G13660]	4404553	4407288	40.02
AT5G13670		WAT1-related protein At5g13670 [Source:UniProtKB/Swiss-Prot;Acc:Q9FNA5]	4407077	4409020	40.64
AT5G13680	ELP1	Elongator complex protein 1 [Source:UniProtKB/Swiss-Prot;Acc:Q9FNA4]	4410314	4415622	41.31
AT5G13690	NAGLU	Alpha-N-acetylglucosaminidase [Source:UniProtKB/Swiss-Prot;Acc:Q9FNA3]	4415754	4420371	38.03
AT5G13700	PAO1	Polyamine oxidase 1 [Source:UniProtKB/Swiss-Prot;Acc:Q9FNA2]	4420069	4423095	39.28
AT5G13710	SMT1	Cycloartenol-C-24-methyltransferase [Source:UniProtKB/Swiss-Prot;Acc:Q9LM02]	4423555	4427324	37.35
AT5G13720		Uncharacterized protein At5g13720 [Source:UniProtKB/TrEMBL;Acc:Q9FNA1]	4427783	4429295	41.51
AT5G13730	SIGD	RNA polymerase sigma factor sigD, chloroplastic [Source:UniProtKB/Swiss-Prot;Acc:Q9ZSL6]	4429095	4430901	41.12
AT5G13740	ZIF1	Protein ZINC INDUCED FACILITATOR 1 [Source:UniProtKB/Swiss-Prot;Acc:Q8RWN2]	4432297	4437148	37.47
AT5G13750	ZIFL1	ZIFL1 [Source:UniProtKB/TrEMBL;Acc:A0A178UFA6]	4437804	4441701	36.69
AT5G13760		Plasma-membrane choline transporter family protein [Source:UniProtKB/TrEMBL;Acc:F4K425]	4441858	4445014	39.31
AT5G13770		Pentatricopeptide repeat-containing protein At5g13770, chloroplastic [Source:UniProtKB/Swiss-Prot;Acc:Q66GP4]	4445423	4447476	42.84
AT5G13780	NAA10	N-terminal acetyltransferase A complex catalytic subunit NAA10 [Source:UniProtKB/Swiss-Prot;Acc:Q9FKI4]	4447259	4448675	41.35
AT5G13790	AGL15	AGL15 [Source:UniProtKB/TrEMBL;Acc:A0A384LKZ8]	4448862	4450958	37.58
AT5G13800	PPH	pheophytinase [Source:TAIR;Acc:AT5G13800]	4451733	4454813	39.63
AT5G13810		AT5g13810/MAC12_24 [Source:UniProtKB/TrEMBL;Acc:Q9FFZ0]	4455148	4457019	39.26
AT5G13820	TRP4	Telomere repeat-binding protein 4 [Source:UniProtKB/Swiss-Prot;Acc:Q9FFY9]	4460840	4464737	37.61
AT5G13825		Putative uncharacterized protein [Source:UniProtKB/TrEMBL;Acc:Q5XV41]	4464967	4466052	32.41
AT5G13830		FtsJ-like methyltransferase family protein [Source:UniProtKB/TrEMBL;Acc:Q9SXH3]	4467311	4468487	42.14
AT5G13840	FZR3	Protein FIZZY-RELATED 3 [Source:UniProtKB/Swiss-Prot;Acc:Q8LPL5]	4468508	4471466	41.77
AT5G02105			4470453	4470828	45.48
AT5G13845		pre-tRNA [Source:TAIR;Acc:AT5G13845]	4471048	4471119	62.5
AT5G13850	NACA3	Nascent polypeptide-associated complex subunit alpha-like protein 3 [Source:UniProtKB/Swiss-Prot;Acc:Q6ICZ8]	4471277	4472946	38.86
AT5G02115			4471502	4471690	34.92
AT5G13860	ELCL	Protein ELC-like [Source:UniProtKB/Swiss-Prot;Acc:Q9FFY6]	4472986	4474530	43.04
AT5G13870	XTH5	Xyloglucan endotransglucosylase/hydrolase [Source:UniProtKB/TrEMBL;Acc:Q67Z90]	4474922	4477090	40.66
AT5G13880		Cotton fiber protein [Source:UniProtKB/TrEMBL;Acc:Q9FFY5]	4477242	4478306	39.53
AT5G13890		At5g13890 [Source:UniProtKB/TrEMBL;Acc:Q9FFY4]	4478787	4481149	40.46
AT5G13887	MIR848A	MIR848a; miRNA [Source:TAIR;Acc:AT5G13887]	4479397	4479591	37.95
AT5G13900	VAS	Lipid transfer-like protein VAS [Source:UniProtKB/Swiss-Prot;Acc:Q9FFY3]	4481122	4481950	38.6
AT5G13910	LEP	LEP [Source:UniProtKB/TrEMBL;Acc:A0A178UQP2]	4482128	4483247	42.5
AT5G13920		GRF zinc finger / Zinc knuckle protein [Source:TAIR;Acc:AT5G13920]	4485626	4487526	40.29
AT5G13930	CHS	Chalcone synthase family protein [Source:UniProtKB/TrEMBL;Acc:Q460R0]	4488476	4490264	47.01
AT5G13940		Aminopeptidase [Source:UniProtKB/TrEMBL;Acc:F4K5D9]	4490263	4495753	37.33
AT5G02125			4495324	4495851	40.15
AT5G13950		unknown protein; BEST Arabidopsis thaliana protein match is: unknown protein (TAIR:AT1G02290.1); Ha. [Source:TAIR;Acc:AT5G13950]	4495844	4500735	41.09
AT5G13960	SUVH4	Histone-lysine N-methyltransferase, H3 lysine-9 specific SUVH4 [Source:UniProtKB/Swiss-Prot;Acc:Q8GZB6]	4501362	4506250	37.25
AT5G13970		AT5g13970/MAC12_6 [Source:UniProtKB/TrEMBL;Acc:Q9FFX8]	4506191	4507864	40.86
AT5G13980		Probable alpha-mannosidase At5g13980 [Source:UniProtKB/Swiss-Prot;Acc:Q8LPJ3]	4508406	4514666	39.08
AT5G13990	ATEXO70C2	Exocyst subunit Exo70 family protein [Source:UniProtKB/TrEMBL;Acc:A0A178UPG0]	4514568	4516892	47.91
AT5G14000	anac084	NAC domain containing protein 84 [Source:TAIR;Acc:AT5G14000]	4517694	4519426	37.22
AT5G02135			4520185	4520428	34.43
AT5G14010	KNU	KNU [Source:UniProtKB/TrEMBL;Acc:A0A384L1F1]	4522149	4522935	44.73
AT5G14020		Endosomal targeting BRO1-like domain-containing protein [Source:UniProtKB/TrEMBL;Acc:Q0WSL5]	4523421	4526167	38.37
AT5G14030		Translocon-associated protein subunit beta [Source:UniProtKB/TrEMBL;Acc:Q94BY3]	4526766	4529614	37.03
AT5G14035		pre-tRNA [Source:TAIR;Acc:AT5G14035]	4530474	4530546	63.01
AT5G14040	MPT3	Mitochondrial phosphate carrier protein 3, mitochondrial [Source:UniProtKB/Swiss-Prot;Acc:Q9FMU6]	4530643	4533119	41.06
AT5G14050		U3 small nucleolar RNA-associated protein 18 homolog [Source:UniProtKB/Swiss-Prot;Acc:Q9FMU5]	4533131	4535327	42.1
AT5G14060	AK2	Aspartokinase [Source:UniProtKB/TrEMBL;Acc:A0A178UPF4]	4535367	4539323	38.44
AT5G02145			4540486	4540695	35.24
AT5G14070	GRXC8	Glutaredoxin-C8 [Source:UniProtKB/Swiss-Prot;Acc:Q8LF89]	4541780	4542585	44.42
AT5G14080		Pentatricopeptide repeat-containing protein At5g14080 [Source:UniProtKB/Swiss-Prot;Acc:Q9FMU2]	4543068	4545416	41.76
AT5G14090	LA1	Protein LAZY 1 [Source:UniProtKB/Swiss-Prot;Acc:Q5XV40]	4547202	4549675	38.28
AT5G14100	ABCI11	ABC transporter I family member 11, chloroplastic [Source:UniProtKB/Swiss-Prot;Acc:Q8LEF6]	4549438	4551843	40.07
AT5G14105		At5g14105 [Source:UniProtKB/TrEMBL;Acc:Q8LEU9]	4551864	4553051	36.62
AT5G14110		Peroxidase (DUF 3339) [Source:UniProtKB/TrEMBL;Acc:Q9FMT9]	4553803	4554597	34.09
AT5G14120		Major facilitator superfamily protein [Source:UniProtKB/TrEMBL;Acc:Q9FMT8]	4555821	4558710	41.97
AT5G02155			4556624	4556997	32.62
AT5G14130		Peroxidase superfamily protein [Source:TAIR;Acc:AT5G14130]	4558704	4560315	42.43
AT5G14140		Nucleic acid binding / zinc ion binding protein [Source:UniProtKB/TrEMBL;Acc:Q0WRF4]	4561449	4563893	38.85
AT5G14150		Emb [Source:UniProtKB/TrEMBL;Acc:Q9FMT6]	4565135	4566988	40.78
AT5G14160		Putative F-box protein At5g14160 [Source:UniProtKB/Swiss-Prot;Acc:Q9FMT5]	4567333	4568467	45.64
AT5G14170	CHC1	SWI/SNF complex component SNF12 homolog [Source:UniProtKB/Swiss-Prot;Acc:Q9FMT4]	4568480	4570994	42.27
AT5G14180	LIP2	Triacylglycerol lipase 2 [Source:UniProtKB/Swiss-Prot;Acc:Q67ZU1]	4571294	4574679	35.85
AT5G14200	IMDH3	3-isopropylmalate dehydrogenase [Source:UniProtKB/TrEMBL;Acc:Q5XF32]	4576123	4578402	39.74
AT5G14210		Leucine-rich repeat protein kinase family protein [Source:TAIR;Acc:AT5G14210]	4578378	4582856	38.49
AT5G14220	HEMG2	Flavin containing amine oxidoreductase family [Source:TAIR;Acc:AT5G14220]	4582629	4587653	37.03
AT5G02175			4589862	4590299	36.53
AT5G02185			4590781	4591189	36.19
AT5G14230		CONTAINS InterPro DOMAIN/s: Ankyrin repeat-containing domain (InterPro:IPR020683), Ankyrin repeat (InterPro:IPR002110); BEST Arabid	4591690	4595964	42.6
AT5G02195			4595271	4595920	46.77
AT5G14240		Thioredoxin superfamily protein [Source:UniProtKB/TrEMBL;Acc:Q9LYA5]	4595928	4597708	40.76
AT5G14250	CSN3	COP9 signalosome complex subunit 3 [Source:UniProtKB/Swiss-Prot;Acc:Q8W575]	4597868	4600835	38.38
AT5G14260		Rubisco methyltransferase family protein [Source:UniProtKB/TrEMBL;Acc:Q8VZB5]	4600904	4604482	39.73
AT5G14270	BET9	Bromodomain and extraterminal domain protein 9 [Source:UniProtKB/TrEMBL;Acc:F4K6Q7]	4604608	4608921	40.59
AT5G14275			4604789	4605007	39.73
AT5G14280	GPL2	GLABROUS1 enhancer-binding protein-like 2 [Source:UniProtKB/Swiss-Prot;Acc:P0DKL0]	4608745	4610448	38.32
AT5G14285		TLC domain-containing protein At5g14285 [Source:UniProtKB/Swiss-Prot;Acc:Q8GYK7]	4610567	4611658	42.67
AT5G02205			4611750	4612095	37.86
AT5G14290		Mitochondrial ribosomal protein L37 [Source:UniProtKB/TrEMBL;Acc:Q1PDW9]	4612097	4614401	42.13
AT5G14300	PHB5	Prohibitin-5, mitochondrial [Source:UniProtKB/Swiss-Prot;Acc:Q9LY99]	4613102	4614023	47.94
AT5G14310	CXE16	CXE16 [Source:UniProtKB/TrEMBL;Acc:A0A178UL11]	4615163	4617633	41.6
AT5G14320	RPS13	30S ribosomal protein S13, chloroplastic [Source:UniProtKB/Swiss-Prot;Acc:P42732]	4617218	4618981	38.78
AT5G14330		Transmembrane protein [Source:UniProtKB/TrEMBL;Acc:Q9LY96]	4619727	4620551	38.79
AT5G02215			4623058	4623308	34.66
AT5G14340	AtMYB40	myb domain protein 40 [Source:TAIR;Acc:AT5G14340]	4623198	4624960	33.64

AT5G14345	ENODL21	ENODL21 [Source:UniProtKB/TrEMBL;Acc:A0A178UD68]	4626175	4626782	39.64
AT5G14350		Pentatricopeptide repeat (PPR) superfamily protein [Source:UniProtKB/TrEMBL;Acc:F4K6R8]	4627140	4628703	42.46
AT5G14360		At5g14360 [Source:UniProtKB/TrEMBL;Acc:Q9LY93]	4630892	4631851	33.44
AT5G14370		CCT motif family protein [Source:UniProtKB/TrEMBL;Acc:Q949T9]	4631891	4633925	41.97
AT5G14380	AGP6	Classical arabinogalactan protein 6 [Source:UniProtKB/Swiss-Prot;Acc:Q9LY91]	4635644	4636676	39.21
AT5G14390		Alpha/beta-Hydrolases superfamily protein [Source:UniProtKB/TrEMBL;Acc:Q9LY90]	4637287	4639835	38.68
AT5G14400	CYP724A1	cytochrome P450, family 724, subfamily A, polypeptide 1 [Source:TAIR;Acc:AT5G14400]	4643510	4646530	31.74
AT5G14410		unknown protein; Ha. [Source:TAIR;Acc:AT5G14410]	4646702	4647503	33.67
AT5G14420	RGLG2	E3 ubiquitin-protein ligase RGLG2 [Source:UniProtKB/Swiss-Prot;Acc:Q9LY87]	4648081	4651442	38.67
AT5G14430		Probable methyltransferase PMT9 [Source:UniProtKB/Swiss-Prot;Acc:Q8VZV7]	4652915	4655973	42.69
AT5G14440		At5g14440 [Source:UniProtKB/TrEMBL;Acc:Q6NPE4]	4655903	4657907	37.46
AT5G02225			4656156	4656362	33.82
AT5G14450		GDSL esterase/lipase At5g14450 [Source:UniProtKB/Swiss-Prot;Acc:Q9LY84]	4658441	4660175	41.04
AT5G14460		Pseudouridine synthase family protein [Source:UniProtKB/TrEMBL;Acc:Q0WVR7]	4660059	4662668	39.66
AT5G14470	GLCAK2	Probable glucuronokinase 2 [Source:UniProtKB/Swiss-Prot;Acc:Q9LY82]	4662775	4664681	37.23
AT5G14480		At5g14480 [Source:UniProtKB/TrEMBL;Acc:Q9LY81]	4667774	4670213	38.61
AT5G14490	anac085	NAC domain containing protein 85 [Source:TAIR;Acc:AT5G14490]	4670289	4672958	36.4
AT5G14495		pre-tRNA [Source:TAIR;Acc:AT5G14495]	4673900	4673971	61.11
AT5G14500		aldose 1-epimerase family protein [Source:TAIR;Acc:AT5G14500]	4674213	4676872	38.95
AT5G14510		ARM repeat superfamily protein [Source:UniProtKB/TrEMBL;Acc:Q9LY78]	4677925	4679317	40.99
AT5G14515			4678005	4679595	39.53
AT5G14520	PES	Pescadillo homolog [Source:UniProtKB/Swiss-Prot;Acc:Q9LYK7]	4680540	4684472	37.81
AT5G14530	APRF1	Protein ANTHESIS POMOTING FACTOR 1 [Source:UniProtKB/Swiss-Prot;Acc:Q9LYK6]	4684597	4687023	39.23
AT5G14540		AT5G14540 protein [Source:UniProtKB/TrEMBL;Acc:B9DHK3]	4687069	4689923	44.03
AT5G02245			4690009	4690105	38.14
AT5G02255			4690371	4690412	38.1
AT5G14550		At5g14550 [Source:UniProtKB/TrEMBL;Acc:B6IDH4]	4691006	4694055	39.34
AT5G14545	MIR398B	MIR398B; miRNA [Source:TAIR;Acc:AT5G14545]	4691022	4691137	47.41
AT5G14560		At5g14560 [Source:UniProtKB/TrEMBL;Acc:Q9LYK3]	4693964	4694086	46.34
AT5G14565	MIR398C	MIR398C; miRNA [Source:TAIR;Acc:AT5G14565]	4694694	4694808	48.7
AT5G14570	NRT2.7	NRT2.7 [Source:UniProtKB/TrEMBL;Acc:A0A178UKX9]	4695084	4697078	47.67
AT5G14580	PNP2	Polyribonucleotide nucleotidyltransferase 2, mitochondrial [Source:UniProtKB/Swiss-Prot;Acc:Q9S7G6]	4697207	4703091	39.61
AT5G14590		Isocitrate dehydrogenase [NADP], chloroplastic/mitochondrial [Source:UniProtKB/Swiss-Prot;Acc:Q8LPJ5]	4703298	4706826	37.97
AT5G14600		tRNA (adenine(58)-N(1))-methyltransferase [Source:UniProtKB/TrEMBL;Acc:Q84K50]	4706891	4709163	37.53
AT5G14602		FUNCTIONS IN: molecular_function unknown; INVOLVED IN: biological_process unknown; LOCATED IN: endomembrane system; BEST Ar	4709262	4709871	37.54
AT5G14610		DEAD box RNA helicase family protein [Source:UniProtKB/TrEMBL;Acc:F4K6V1]	4710563	4715076	40.94
AT5G14620	DRM2	DNA (cytosine-5)-methyltransferase DRM2 [Source:UniProtKB/Swiss-Prot;Acc:Q9M548]	4715105	4718704	38.58
AT5G14640	ASK5	Shaggy-related protein kinase epsilon [Source:UniProtKB/Swiss-Prot;Acc:Q8VZD5]	4718994	4722371	37.86
AT5G02265			4723017	4723253	36.29
AT5G02275			4723632	4723983	33.24
AT5G14650		Pectin lyase-like superfamily protein [Source:UniProtKB/TrEMBL;Acc:Q9LYJ5]	4724407	4726675	39
AT5G14660	PDF1B	Peptide deformylase 1B, chloroplastic/mitochondrial [Source:UniProtKB/Swiss-Prot;Acc:Q9FUZ2]	4726641	4728746	38.22
AT5G14670	ATARFA1B	ADP-ribosylation factor A1B [Source:UniProtKB/TrEMBL;Acc:Q9LYJ3]	4728947	4730952	35.39
AT5G14680		Adenine nucleotide alpha hydrolases-like superfamily protein [Source:UniProtKB/TrEMBL;Acc:Q9LYJ2]	4730836	4733225	39.04
AT5G14690		At5g14690 [Source:UniProtKB/TrEMBL;Acc:Q9LYJ1]	4733658	4736645	35.37
AT5G14700		Cinnamoyl CoA reductase-like protein [Source:UniProtKB/TrEMBL;Acc:Q9LYJ0]	4740238	4743539	34.86
AT5G14710		Proteasome assembly chaperone-like protein [Source:UniProtKB/TrEMBL;Acc:Q9LER5]	4746264	4747934	34.53
AT5G14720		Protein kinase superfamily protein [Source:UniProtKB/TrEMBL;Acc:Q84WU5]	4747860	4753781	36.95
AT5G14730		At5g14730 [Source:UniProtKB/TrEMBL;Acc:Q9LER3]	4756057	4757744	41.29
AT5G14740	BCA2	Beta carbonic anhydrase 2, chloroplastic [Source:UniProtKB/Swiss-Prot;Acc:P42737]	4757856	4762666	31.45
AT5G14750	WER	WER1 [Source:UniProtKB/TrEMBL;Acc:A0A384KIP7]	4763372	4764834	31.78
AT5G14760	AO	L-aspartate oxidase, chloroplastic [Source:UniProtKB/Swiss-Prot;Acc:Q94AY1]	4768263	4772153	38.78
AT5G14770		Pentatricopeptide repeat-containing protein At5g14770, mitochondrial [Source:UniProtKB/Swiss-Prot;Acc:Q9LER0]	4772117	4775833	40.06
AT5G14780	FDH1	Formate dehydrogenase, mitochondrial [Source:UniProtKB/TrEMBL;Acc:A0A178UHK7]	4776733	4779526	40.66
AT5G14790		ARM repeat superfamily protein [Source:UniProtKB/TrEMBL;Acc:Q9LEQ9]	4782909	4785847	37.73
AT5G14800	PROC1	Pyrroline-5-carboxylate reductase [Source:UniProtKB/Swiss-Prot;Acc:P54904]	4785960	4787996	42.27
AT5G14820		Pentatricopeptide repeat-containing protein At5g14820, mitochondrial [Source:UniProtKB/Swiss-Prot;Acc:Q9LEQ7]	4792072	4794153	43.8
AT5G14850		Mannosyltransferase [Source:UniProtKB/TrEMBL;Acc:Q94A15]	4801870	4805036	38.93
AT5G14860		Glycosyltransferase [Source:UniProtKB/TrEMBL;Acc:Q1PDW8]	4805846	4807837	42.57
AT5G14870	CNGC18	Cyclic nucleotide-gated ion channel 18 [Source:UniProtKB/Swiss-Prot;Acc:Q9LEQ3]	4808091	4811093	42.09
AT5G14880	POT8	Potassium transporter [Source:UniProtKB/TrEMBL;Acc:A0A178UBD9]	4813931	4817881	39.26
AT5G14890		NHL domain-containing protein [Source:TAIR;Acc:AT5G14890]	4817997	4820601	38.66
AT5G14895		NHL domain protein [Source:UniProtKB/TrEMBL;Acc:A0A1P8BDM5]	4820830	4821859	41.55
AT5G14900		Helicase associated (HA2) domain-containing protein [Source:UniProtKB/TrEMBL;Acc:Q9LFR5]	4822676	4823581	42.6
AT5G14910		AT5g14910/F2G14_30 [Source:UniProtKB/TrEMBL;Acc:Q93VK7]	4823553	4825652	36.86
AT5G14920	GASA14	Gibberellin-regulated protein 14 [Source:UniProtKB/Swiss-Prot;Acc:Q9LFR3]	4826141	4828384	39.08
AT5G14930	SAG101	Senescence-associated carboxylesterase 101 [Source:UniProtKB/Swiss-Prot;Acc:Q4F883]	4828499	4830982	37.56
AT5G14940	NPF5.8	Protein NRT1/ PTR FAMILY 5.8 [Source:UniProtKB/Swiss-Prot;Acc:Q9LFR1]	4831583	4834532	36.64
AT5G14950	GMII	Alpha-mannosidase 2 [Source:UniProtKB/Swiss-Prot;Acc:Q9LFR0]	4837071	4842067	41.5
AT5G14960	E2FD	E2F transcription factor-like E2FD [Source:UniProtKB/Swiss-Prot;Acc:Q9LFQ9]	4843855	4846664	35.91
AT5G14970		At4g33110 [Source:UniProtKB/TrEMBL;Acc:Q9LFQ8]	4847247	4849053	40.51
AT5G14980		Alpha/beta-Hydrolases superfamily protein [Source:UniProtKB/TrEMBL;Acc:Q9LFQ7]	4849600	4850583	53.05
AT5G14990		WPP domain associated protein [Source:UniProtKB/TrEMBL;Acc:Q9LFQ6]	4850668	4853087	35.83
AT5G14995		ECA1 gametogenesis related family protein [Source:UniProtKB/TrEMBL;Acc:A8MS22]	4853413	4853718	43.14
AT5G15000		ECA1 gametogenesis family protein [Source:UniProtKB/TrEMBL;Acc:F4K8A7]	4854875	4855171	45.12
AT5G15008		unknown protein; Ha. [Source:TAIR;Acc:AT5G15008]	4856975	4857139	35.15
AT5G15010		Pentatricopeptide repeat-containing protein At5g15010, mitochondrial [Source:UniProtKB/Swiss-Prot;Acc:Q9LFQ4]	4857147	4859125	40.42
AT5G15020	SNL2	Paired amphipathic helix protein Sin3-like 2 [Source:UniProtKB/Swiss-Prot;Acc:Q9LFQ3]	4859127	4866034	38.43
AT5G15022		Potential natural antisense gene, locus overlaps with AT5G15030 [Source:TAIR;Acc:AT5G15022]	4866237	4866939	32.01
AT5G02295			4866316	4866393	32.05
AT5G02305			4866462	4866538	37.66
AT5G02315			4866592	4866655	40.62
AT5G02325			4866720	4866816	31.96
AT5G15025		Paired amphipathic helix Sin3-like protein [Source:UniProtKB/TrEMBL;Acc:A0A1P8BFD5]	4866954	4867558	38.51
AT5G15030		Paired amphipathic helix (PAH2) superfamily protein [Source:TAIR;Acc:AT5G15030]	4867971	4869079	34.54
AT5G15040		Paired amphipathic helix (PAH2) superfamily protein [Source:UniProtKB/TrEMBL;Acc:Q9LFQ1]	4869754	4870741	36.03
AT5G15050	GLCAT14B	Beta-glucuronosyltransferase GlcAT14B [Source:UniProtKB/Swiss-Prot;Acc:Q9LFQ0]	4871531	4873683	38.78
AT5G15060	LBD34	LOB domain-containing protein 34 [Source:UniProtKB/Swiss-Prot;Acc:P59469]	4874332	4875949	38.32
AT5G15070			4876515	4885836	37.94
AT5G15080	PIX7	Probable serine/threonine-protein kinase PIX7 [Source:UniProtKB/Swiss-Prot;Acc:Q9LFP7]	4886029	4888817	37.58
AT5G15090	VDAC3	Mitochondrial outer membrane protein porin 3 [Source:UniProtKB/Swiss-Prot;Acc:Q9SMX3]	4888964	4891603	38.64
AT5G15100	PIN8	Auxin efflux carrier component 8 [Source:UniProtKB/Swiss-Prot;Acc:Q9LFP6]	4892016	4894113	32.51
AT5G15110		Pectate lyase family protein [Source:TAIR;Acc:AT5G15110]	4895750	4897966	38.75
AT5G15120	PCO1	Plant cysteine oxidase 1 [Source:UniProtKB/Swiss-Prot;Acc:Q9LXG9]	4898641	4900734	36.68
AT5G15130	WRKY72	Probable WRKY transcription factor 72 [Source:UniProtKB/Swiss-Prot;Acc:Q9LXG8]	4904290	4907094	37.4
AT5G15140		Aldose 1-epimerase family protein [Source:UniProtKB/TrEMBL;Acc:Q9LXG7]	4908651	4910920	34.98
AT5G15150	HAT7	Homeobox-leucine zipper protein HAT7 [Source:UniProtKB/Swiss-Prot;Acc:Q00466]	4913699	4915976	34.55
AT5G02335			4917187	4917590	33.17
AT5G15160	PRE2	Transcription factor PRE2 [Source:UniProtKB/Swiss-Prot;Acc:Q9LXG5]	4921243	4922812	33.12
AT5G15170	TDP1	Tyrosyl-DNA phosphodiesterase 1 [Source:UniProtKB/Swiss-Prot;Acc:Q8H1D9]	4925850	4929502	38.38
AT5G15175		pre-tRNA [Source:TAIR;Acc:AT5G15175]	4929560	4929633	58.11
AT5G15180	PER56	Peroxidase 56 [Source:UniProtKB/Swiss-Prot;Acc:Q9LXG3]	4930425	4932406	34.96
AT5G15190		At5g15190 [Source:UniProtKB/TrEMBL;Acc:Q8GW49]	4932708	4933620	35.82
AT5G15200	RPS9B	40S ribosomal protein S9-1 [Source:UniProtKB/Swiss-Prot;Acc:Q9LXG1]	4934810	4936429	38.64
AT5G15210	ZHD8	Zinc-finger homeodomain protein 8 [Source:UniProtKB/Swiss-Prot;Acc:Q9LXG0]	4937465	4939207	39.7

AT5G15220		AT5g15220/F8M21_110 [Source:UniProtKB/TrEMBL;Acc:Q94AC6]	4941237	4942784	37.86
AT5G02345			4944177	4944415	32.64
AT5G15230	GASA4	Gibberellin-regulated protein 4 [Source:UniProtKB/Swiss-Prot;Acc:P46690]	4944816	4946299	32.14
AT5G15240	AVT1J	Amino acid transporter AVT1J [Source:UniProtKB/Swiss-Prot;Acc:Q9LXF8]	4947577	4950445	34.79
AT5G15250	FTSH6	ATP-dependent zinc metalloprotease FTSH 6, chloroplastic [Source:UniProtKB/Swiss-Prot;Acc:Q1PDW5]	4950359	4952803	43.44
AT5G15260		AT5g15260/F8M21_150 [Source:UniProtKB/TrEMBL;Acc:Q8VYH4]	4952881	4955027	36.56
AT5G15265		Transmembrane protein [Source:UniProtKB/TrEMBL;Acc:Q56YM4]	4956314	4957633	31.52
AT5G15270		RNA-binding KH domain-containing protein [Source:UniProtKB/TrEMBL;Acc:Q0WNX3]	4958303	4962102	39.58
AT5G15280		Pentatricopeptide repeat-containing protein At5g15280, mitochondrial [Source:UniProtKB/Swiss-Prot;Acc:Q9LXF4]	4962149	4966170	39.76
AT5G15290	CASP5	Casparian strip membrane protein 5 [Source:UniProtKB/Swiss-Prot;Acc:Q9LXF3]	4967011	4968092	38.35
AT5G15300	PCMP-E40	Pentatricopeptide repeat-containing protein At5g15300 [Source:UniProtKB/Swiss-Prot;Acc:Q9LXF2]	4968089	4970045	40.16
AT5G02355			4971886	4972090	37.56
AT5G02365			4971955	4972298	37.79
AT5G02375			4972814	4973058	39.18
AT5G02395			4973106	4973387	33.69
AT5G15310	ATMYB16	MYB16 [Source:UniProtKB/TrEMBL;Acc:A0A178UJ10]	4974671	4976470	37.67
AT5G15320		ATP synthase E chain [Source:UniProtKB/TrEMBL;Acc:Q8GXE7]	4977470	4979076	37.27
AT5G15330	ATSPX4	SPX domain gene 4 [Source:TAIR;Acc:AT5G15330]	4980374	4982231	39.4
AT5G15340	PCMP-H91	Pentatricopeptide repeat-containing protein At5g15340, mitochondrial [Source:UniProtKB/Swiss-Prot;Acc:Q9LXE8]	4982273	4984398	43.89
AT5G15350	ENODL17	Lamin-like protein [Source:UniProtKB/Swiss-Prot;Acc:Q39131]	4984919	4986377	37.56
AT5G15360		Transmembrane protein [Source:UniProtKB/TrEMBL;Acc:Q9LXE7]	4987437	4989182	46.68
AT5G02405			4990612	4991041	38.6
AT5G15380	DRM1	DNA (cytosine-5)-methyltransferase DRM1 [Source:UniProtKB/Swiss-Prot;Acc:Q9LXE5]	4991347	4994924	38.71
AT5G15390		rRNA methylase-like protein [Source:UniProtKB/TrEMBL;Acc:Q9LXE4]	4995138	4997489	41.41
AT5G15400	PUB1	Probable ubiquitin conjugation factor E4 [Source:UniProtKB/Swiss-Prot;Acc:Q9LF41]	4997489	5003253	38.89
AT5G15410	CNGC2	Cyclic nucleotide-gated ion channel 2 [Source:UniProtKB/Swiss-Prot;Acc:O65718]	5003226	5006882	42.63
AT5G15420		Uncharacterized protein T20K14_30 [Source:UniProtKB/TrEMBL;Acc:Q9LF40]	5007826	5008964	42.76
AT5G15430		At5g15430 [Source:UniProtKB/TrEMBL;Acc:Q9LF39]	5009687	5011856	37.51
AT5G15440	EDL1	EID1-like F-box protein 1 [Source:UniProtKB/Swiss-Prot;Acc:Q9LF38]	5012061	5014281	38.72
AT5G02415			5012367	5013135	36.02
AT5G15450	CLPB3	Chaperone protein ClpB3, chloroplastic [Source:UniProtKB/Swiss-Prot;Acc:Q9LF37]	5014212	5018347	40.45
AT5G15460	MUB2	Membrane-anchored ubiquitin-fold protein 2 [Source:UniProtKB/Swiss-Prot;Acc:Q8LCS8]	5018680	5020926	36.67
AT5G15470	GAUT14	GAUT14 [Source:UniProtKB/TrEMBL;Acc:A0A384LD84]	5020941	5024750	38.29
AT5G15480		C2H2-type zinc finger family protein [Source:UniProtKB/TrEMBL;Acc:Q9LF34]	5025952	5027160	41.11
AT5G15490	UGD3	UDP-glucose 6-dehydrogenase [Source:UniProtKB/TrEMBL;Acc:A0A178UJW9]	5027638	5030245	41.18
AT5G15500		Ankyrin repeat family protein [Source:UniProtKB/TrEMBL;Acc:Q9LF32]	5031648	5033540	40.99
AT5G15510		TPX2 (Targeting protein for Xklp2) protein family [Source:UniProtKB/TrEMBL;Acc:F4K9U0]	5033933	5036824	38.83
AT5G15520	RPS19B	40S ribosomal protein S19-2 [Source:UniProtKB/Swiss-Prot;Acc:Q9LF30]	5037021	5038754	37.49
AT5G15530	BCCP2	Biotin carboxyl carrier protein of acetyl-CoA carboxylase [Source:UniProtKB/TrEMBL;Acc:A0A178U9S1]	5038563	5040840	39.33
AT5G15533			5040657	5040836	35.56
AT5G15537		Transmembrane protein [Source:UniProtKB/TrEMBL;Acc:A0A1P8BHW0]	5040957	5045549	35.92
AT5G02425			5045301	5045978	38.94
AT5G15540	EMB2773	Sister chromatid cohesion protein SCC2 [Source:UniProtKB/Swiss-Prot;Acc:A5HEI1]	5047515	5057666	38.84
AT5G15550	WDR12	Ribosome biogenesis protein WDR12 homolog [Source:UniProtKB/TrEMBL;Acc:A0A178UL95]	5058672	5062144	38.47
AT5G15560		Uncharacterized protein T20K14_170 [Source:UniProtKB/TrEMBL;Acc:Q9LF26]	5064051	5064889	36.47
AT5G15570		Bromodomain transcription factor [Source:UniProtKB/TrEMBL;Acc:Q9LF25]	5066204	5068199	41.08
AT5G15580	LNG1	Protein LONGIFOLIA 1 [Source:UniProtKB/Swiss-Prot;Acc:Q9LF24]	5068237	5072492	41.21
AT5G15581		Putative uncharacterized protein T20K14_200 [Source:UniProtKB/TrEMBL;Acc:Q9LF23]	5071829	5072140	38.46
AT5G15600	SP1L4	Protein SPIRAL1-like 4 [Source:UniProtKB/Swiss-Prot;Acc:Q9LF22]	5078200	5079327	38.3
AT5G15610		Eukaryotic translation initiation factor 3 subunit M [Source:UniProtKB/TrEMBL;Acc:Q9LF21]	5079458	5082288	38.86
AT5G15620		Putative F-box/LRR-repeat protein At5g15620 [Source:UniProtKB/Swiss-Prot;Acc:Q9LF20]	5082465	5084136	38.82
AT5G15630	COBL4	COBRA-like protein 4 [Source:UniProtKB/Swiss-Prot;Acc:Q9LFW3]	5084524	5086811	38.59
AT5G15640		Mitochondrial substrate carrier family protein [Source:UniProtKB/TrEMBL;Acc:Q949U9]	5087219	5090047	39.31
AT5G15650	RGP2	UDP-arabinopyranose mutase 2 [Source:UniProtKB/Swiss-Prot;Acc:Q9LFW1]	5092140	5095302	39.61
AT5G15660		Putative F-box protein At5g15660 [Source:UniProtKB/Swiss-Prot;Acc:Q9LFW0]	5095652	5097041	40.07
AT5G15670		Putative F-box protein At5g15670 [Source:UniProtKB/Swiss-Prot;Acc:Q9LFV9]	5099595	5100407	41.45
AT5G15680		ARM repeat superfamily protein [Source:UniProtKB/TrEMBL;Acc:F4KB73]	5100916	5111105	41.11
AT5G15685		Mutator transposase MUDRA protein [Source:UniProtKB/TrEMBL;Acc:A0A1P8BBR6]	5111820	5113456	41.54
AT5G15690		Zinc ion binding protein [Source:UniProtKB/TrEMBL;Acc:Q9LFV7]	5113823	5114477	40.46
AT5G15700		DNA-directed RNA polymerase [Source:UniProtKB/TrEMBL;Acc:F4KB75]	5114761	5121841	38.95
AT5G15710		F-box/kelch-repeat protein At5g15710 [Source:UniProtKB/Swiss-Prot;Acc:Q9LFV5]	5122428	5124947	40.24
AT5G15720	GLIP7	GDSL esterase/lipase 7 [Source:UniProtKB/Swiss-Prot;Acc:Q8LFJ9]	5124525	5126486	37.51
AT5G15725		Transmembrane protein [Source:UniProtKB/TrEMBL;Acc:F4KB79]	5127375	5128418	31.7
AT5G15730	CRLK2	Calcium/calmodulin-regulated receptor-like kinase 2 [Source:UniProtKB/Swiss-Prot;Acc:Q9LFV3]	5130449	5133267	38.88
AT5G15740	OFUT34	O-fucosyltransferase 34 [Source:UniProtKB/Swiss-Prot;Acc:Q4V398]	5134507	5137707	38.77
AT5G15745			5137456	5137758	35.64
AT5G15750		Alpha-L RNA-binding motif/Ribosomal protein S4 family protein [Source:UniProtKB/TrEMBL;Acc:Q683D4]	5141035	5142890	36.64
AT5G15760		PSRP3/2 [Source:UniProtKB/TrEMBL;Acc:A0A178UH96]	5142990	5144606	40.88
AT5G15770	GNA1	Glucosamine 6-phosphate N-acetyltransferase [Source:UniProtKB/Swiss-Prot;Acc:Q9LFU9]	5144091	5144580	41.02
AT5G15780		At5g15780 [Source:UniProtKB/TrEMBL;Acc:Q9LFU8]	5144629	5146458	42.51
AT5G15790		RING/U-box superfamily protein [Source:TAIR;Acc:AT5G15790]	5148722	5151433	37.68
AT5G02445			5149165	5149358	32.99
AT5G15800	01-Sep	K-box region and MADS-box transcription factor family protein [Source:UniProtKB/TrEMBL;Acc:F4KB90]	5151334	5154253	34.14
AT5G02455			5153720	5154168	32.07
AT5G15802		Chaperone [Source:UniProtKB/TrEMBL;Acc:Q8GYN2]	5156244	5157545	36.71
AT5G15805		pre-tRNA [Source:TAIR;Acc:AT5G15805]	5157639	5157712	51.35
AT5G15810		Probable tRNA (guanine(26)-N(2))-dimethyltransferase 1 [Source:UniProtKB/Swiss-Prot;Acc:Q9LFU5]	5157841	5161363	38.97
AT5G15815		pre-tRNA [Source:TAIR;Acc:AT5G15815]	5161512	5161583	61.11
AT5G15820		AT5g15820/F14F8_200 [Source:UniProtKB/TrEMBL;Acc:Q9LFU4]	5161724	5163195	41.1
AT5G15830	AtbZIP3	At5g15830 [Source:UniProtKB/TrEMBL;Acc:Q9LFU3]	5168194	5169365	36.26
AT5G02465			5169699	5169907	43.54
AT5G15833	MIR865A	MIR865a; miRNA [Source:TAIR;Acc:AT5G15833]	5169990	5170131	29.58
AT5G15840	CO	Zinc finger protein CONSTANS [Source:UniProtKB/Swiss-Prot;Acc:Q39057]	5170162	5173085	40.77
AT5G15843		Transmembrane protein [Source:UniProtKB/TrEMBL;Acc:A0A1P8B9E1]	5170416	5172382	39.65
AT5G15845		other RNA [Source:TAIR;Acc:AT5G15845]	5173081	5178291	38.51
AT5G15850	COL1	Zinc finger protein CONSTANS-LIKE 1 [Source:UniProtKB/Swiss-Prot;Acc:O50055]	5176091	5177897	43.39
AT5G15853		unknown protein; LOCATED IN: endomembrane system; Ha. [Source:TAIR;Acc:AT5G15853]	5178107	5178395	40.48
AT5G15860	ICME	Isoprenylcysteine alpha-carbonyl methylesterase ICME [Source:UniProtKB/Swiss-Prot;Acc:Q94AS5]	5178157	5181761	39.17
AT5G15870		Glycosyl hydrolase family 81 protein [Source:UniProtKB/TrEMBL;Acc:Q9LFT3]	5182367	5184948	42.76
AT5G15880		At5g15880 [Source:UniProtKB/TrEMBL;Acc:Q9LFT2]	5185651	5187706	39.98
AT5G15890	TBL21	Protein trichome birefringence-like 21 [Source:UniProtKB/Swiss-Prot;Acc:Q9LFT1]	5187587	5189348	43.64
AT5G15900	TBL19	TRICHOME BIREFRINGENCE-LIKE 19 [Source:TAIR;Acc:AT5G15900]	5189376	5192176	36.42
AT5G15910		NAD(P)-binding Rossmann-fold superfamily protein [Source:UniProtKB/TrEMBL;Acc:Q949S7]	5192842	5195396	36.67
AT5G15920	SMC5	Structural maintenance of chromosomes protein 5 [Source:UniProtKB/Swiss-Prot;Acc:Q9LFS8]	5195593	5199697	40.58
AT5G15930	PAM1	Plant adhesion molecule 1 (PAM1) [Source:UniProtKB/TrEMBL;Acc:O81347]	5199711	5202452	36.69
AT5G15940		NAD(P)-binding Rossmann-fold superfamily protein [Source:TAIR;Acc:AT5G15940]	5202466	5204883	35.73
AT5G15950	SAMDC2	S-adenosylmethionine decarboxylase proenzyme 2 [Source:UniProtKB/Swiss-Prot;Acc:Q9S7T9]	5205869	5208036	40.04
AT5G15960	KIN1	Stress-induced protein KIN1 [Source:UniProtKB/Swiss-Prot;Acc:P18612]	5209717	5210826	35.32
AT5G15970	KIN2	KIN2 [Source:UniProtKB/TrEMBL;Acc:A0A384KRF8]	5211719	5212743	36.1
AT5G15980		Pentatricopeptide repeat-containing protein At5g15980, mitochondrial [Source:UniProtKB/Swiss-Prot;Acc:Q8LPF1]	5213062	5215674	40.26
AT5G02485			5216103	5216471	36.86
AT5G16000	NIK1	Protein NSP-INTERACTING KINASE 1 [Source:UniProtKB/Swiss-Prot;Acc:Q9LFS4]	5223934	5227288	36.18
AT5G16010		3-oxo-5-alpha-steroid 4-dehydrogenase family protein [Source:UniProtKB/TrEMBL;Acc:Q9LFS3]	5227747	5229117	38.51
AT5G16020	GEX3	Protein GAMETE EXPRESSED 3 [Source:UniProtKB/Swiss-Prot;Acc:Q9LFS2]	5229513	5232311	38.37
AT5G16023	RTFL18	DVL1 [Source:UniProtKB/TrEMBL;Acc:Q6X5V0]	5232532	5233036	35.84

AT5G02495			5236634	5236879	32.93
AT5G16030		unknown protein; BEST Arabidopsis thaliana protein match is: unknown protein (TAIR:AT3G02500.1); Ha. [Source:TAIR;Acc:AT5G16030]	5237970	5240748	32.85
AT5G02515			5239271	5240333	28.32
AT5G16040		At5g16040 [Source:UniProtKB/TrEMBL;Acc:Q9LFS0]	5240675	5243473	40.09
AT5G16050	GRF5	14-3-3-like protein GF14 upsilon [Source:UniProtKB/Swiss-Prot;Acc:P42645]	5243555	5245838	39.58
AT5G16060		COX assembly mitochondrial protein [Source:UniProtKB/TrEMBL;Acc:A0A178UR08]	5246080	5247442	37.12
AT5G16070	CCT6B	T-complex protein 1 subunit zeta 2 [Source:UniProtKB/Swiss-Prot;Acc:Q8L7N0]	5247354	5251179	38.11
AT5G16080	CXE17	Probable carboxylesterase 17 [Source:UniProtKB/Swiss-Prot;Acc:Q9LFR7]	5252251	5253790	43.12
AT5G16090		INVOLVED IN: nucleotide-excision repair; LOCATED IN: nucleus; CONTAINS InterPro DOMAIN/s: Ubiquitin-associated/translation elongati	5255797	5257351	38.33
AT5G16100		unknown protein; Ha. [Source:TAIR;Acc:AT5G16100]	5257609	5259924	38.04
AT5G16110		Uncharacterized protein T21H19_30 [Source:UniProtKB/TrEMBL;Acc:Q9LF17]	5260522	5262840	37.6
AT5G16120		alpha/beta-Hydrolases superfamily protein [Source:TAIR;Acc:AT5G16120]	5265473	5268078	35.99
AT5G16130	RPS7C	40S ribosomal protein S7-3 [Source:UniProtKB/Swiss-Prot;Acc:Q8LD03]	5268421	5270199	37.77
AT5G16140	CRS2B	Chloroplastic group IIB intron splicing facilitator CRS2-B, chloroplastic [Source:UniProtKB/Swiss-Prot;Acc:Q9LF14]	5270160	5271963	36.36
AT5G16150	GLT1	Plastidic glucose transporter 4 [Source:UniProtKB/Swiss-Prot;Acc:Q56ZZ7]	5272476	5275815	40.33
AT5G16160		At5g16160 [Source:UniProtKB/TrEMBL;Acc:Q67Z64]	5275695	5276643	36.78
AT5G16170		At5g16170 [Source:UniProtKB/TrEMBL;Acc:Q4V3A2]	5277787	5279821	36.46
AT5G16180	CRS1	Chloroplastic group IIA intron splicing facilitator CRS1, chloroplastic [Source:UniProtKB/Swiss-Prot;Acc:Q9LF10]	5279884	5283102	38.55
AT5G16190	ATCSLA11	cellulose synthase like A11 [Source:TAIR;Acc:AT5G16190]	5283447	5286842	34.07
AT5G16200		50S ribosomal protein-like protein [Source:UniProtKB/TrEMBL;Acc:Q9LF08]	5289401	5290506	41.23
AT5G16210		AT5g16210/T21H19_130 [Source:UniProtKB/TrEMBL;Acc:Q8VYW7]	5290696	5298169	38.98
AT5G16220		Octicosapeptide/Phox/Bem1p family protein [Source:UniProtKB/TrEMBL;Acc:Q9LF06]	5298112	5300642	41.68
AT5G16230	S-ACP-DES3	Stearoyl-[acyl-carrier-protein] 9-desaturase 3, chloroplastic [Source:UniProtKB/Swiss-Prot;Acc:Q9LF05]	5302905	5306088	35.08
AT5G16235		other RNA [Source:TAIR;Acc:AT5G16235]	5304494	5305945	40.15
AT5G16240	S-ACP-DES1	Stearoyl-[acyl-carrier-protein] 9-desaturase 1, chloroplastic [Source:UniProtKB/Swiss-Prot;Acc:Q9LF04]	5306740	5309961	36.13
AT5G16250		Transmembrane protein [Source:UniProtKB/TrEMBL;Acc:Q9LF03]	5310052	5310951	42.33
AT5G16260	ELF9	At5g16260 [Source:UniProtKB/TrEMBL;Acc:Q9LF02]	5311141	5315834	37.88
AT5G16270	SYN4	Sister chromatid cohesion 1 protein 4 [Source:UniProtKB/Swiss-Prot;Acc:Q8W1Y0]	5316197	5322700	39.01
AT5G16280		Tetratricopeptide repeat (TPR)-like superfamily protein [Source:TAIR;Acc:AT5G16280]	5323133	5331694	37.4
AT5G16285		Putative F-box protein At5g16285 [Source:UniProtKB/Swiss-Prot;Acc:Q3E9H1]	5332830	5333102	46.15
AT5G16290	VAT1	Acetolactate synthase small subunit 1, chloroplastic [Source:UniProtKB/Swiss-Prot;Acc:Q9FFF4]	5333750	5337784	37.84
AT5G16300		Low density lipoprotein B-like protein [Source:UniProtKB/TrEMBL;Acc:Q9FFF3]	5337895	5342432	40.7
AT5G16310	UCH1	Ubiquitin carboxyl-terminal hydrolase [Source:UniProtKB/Swiss-Prot;Acc:Q9FFF2]	5342391	5344285	38.58
AT5G02525			5343699	5344026	31.1
AT5G16320	FRL1	FRIGIDA-like protein 1 [Source:UniProtKB/Swiss-Prot;Acc:Q9FFF1]	5344495	5346106	41.94
AT5G16330		Gb [Source:UniProtKB/TrEMBL;Acc:Q9FFF0]	5346163	5346977	43.44
AT5G16340	AAE6	Probable acyl-activating enzyme 6 [Source:UniProtKB/Swiss-Prot;Acc:Q9FFE9]	5348993	5351095	45.08
AT5G16350		Emb [Source:UniProtKB/TrEMBL;Acc:Q9FFE8]	5352572	5355070	34.53
AT5G16360		Gb [Source:UniProtKB/TrEMBL;Acc:Q9FFE7]	5354876	5356353	36.67
AT5G16370	AAE5	Probable acyl-activating enzyme 5, peroxisomal [Source:UniProtKB/Swiss-Prot;Acc:Q9FFE6]	5356551	5358789	45.42
AT5G16375		pre-tRNA [Source:TAIR;Acc:AT5G16375]	5358916	5358997	58.54
AT5G16380		Uncharacterized protein At5g16380 [Source:UniProtKB/TrEMBL;Acc:Q8VZN3]	5359307	5360710	39.81
AT5G16390	BCCP1	Biotin carboxyl carrier protein of acetyl-CoA carboxylase 1, chloroplastic [Source:UniProtKB/Swiss-Prot;Acc:Q42533]	5360789	5363297	38.58
AT5G16400	TRXF2	TRXF2 [Source:UniProtKB/TrEMBL;Acc:A0A178UDV9]	5363420	5365391	37.32
AT5G16410		HXXXD-type acyl-transferase family protein [Source:UniProtKB/TrEMBL;Acc:Q9FFE4]	5365597	5367612	41.82
AT5G16420		Pentatricopeptide repeat-containing protein At5g16420, mitochondrial [Source:UniProtKB/Swiss-Prot;Acc:Q9FFE3]	5367971	5370242	41.51
AT5G16430		Cysteine/Histidine-rich C1 domain family protein [Source:UniProtKB/TrEMBL;Acc:Q9FFE2]	5370350	5370607	42.64
AT5G16440	IPP1	Isopentenyl-diphosphate Delta-isomerase I, chloroplastic [Source:UniProtKB/Swiss-Prot;Acc:Q38929]	5371627	5373809	37.93
AT5G16450		Putative 4-hydroxy-4-methyl-2-oxoglutarate aldolase 2 [Source:UniProtKB/Swiss-Prot;Acc:Q9FFE0]	5373820	5375459	36.16
AT5G02535			5374812	5374999	34.04
AT5G16453		Defensin-like protein 24 [Source:UniProtKB/Swiss-Prot;Acc:Q2V371]	5375455	5375999	36.7
AT5G16460	SEI1	Seipin-1 [Source:UniProtKB/Swiss-Prot;Acc:Q9FFD9]	5376961	5378533	38.72
AT5G16470	MBS2	Protein METHYLENE BLUE SENSITIVITY 2 [Source:UniProtKB/Swiss-Prot;Acc:Q9FFD8]	5379359	5380512	38.99
AT5G16480	DSP5	Tyrosine-protein phosphatase DSP5 [Source:UniProtKB/Swiss-Prot;Acc:Q9FFD7]	5381069	5382939	35.65
AT5G16486		RNA-directed DNA polymerase (Reverse transcriptase)-related family protein [Source:UniProtKB/TrEMBL;Acc:Q1G3F8]	5383597	5384226	44.92
AT5G16490	RIC4	CRIB domain-containing protein RIC4 [Source:UniProtKB/Swiss-Prot;Acc:Q9FFD5]	5384250	5385230	34.86
AT5G16500	LIP1	Receptor-like kinase LIP1 [Source:UniProtKB/Swiss-Prot;Acc:Q1PDW3]	5386524	5389311	39.31
AT5G16510	RGP5	Glycosyltransferase (Fragment) [Source:UniProtKB/TrEMBL;Acc:W8Q2T5]	5392576	5394573	40.74
AT5G16520		At5g16520 [Source:UniProtKB/TrEMBL;Acc:Q8GUJ0]	5394544	5397466	39.38
AT5G16530	PIN5	Auxin efflux carrier component [Source:UniProtKB/TrEMBL;Acc:A0A178UIR3]	5400600	5402845	35.53
AT5G16540	ZFN3	Zinc finger CCCH domain-containing protein 57 [Source:UniProtKB/Swiss-Prot;Acc:Q8L7N8]	5403069	5405173	40.76
AT5G16550		Voltage-dependent L-type calcium channel subunit [Source:UniProtKB/TrEMBL;Acc:Q9FFC9]	5405346	5406887	39.69
AT5G16560	KAN1	Transcription repressor KAN1 [Source:UniProtKB/Swiss-Prot;Acc:Q93WJ9]	5406863	5411766	33.05
AT5G02555			5411197	5411631	36.09
AT5G16570	GLN1-4	Glutamine synthetase [Source:UniProtKB/TrEMBL;Acc:A0A178UB24]	5421589	5424749	36.38
AT5G16580	BGLU2	Putative beta-glucosidase 2 [Source:UniProtKB/Swiss-Prot;Acc:Q9FMD8]	5425641	5427472	37.45
AT5G16590	LRR1	Probable inactive receptor kinase At5g16590 [Source:UniProtKB/Swiss-Prot;Acc:Q9FMD7]	5431584	5434186	42.03
AT5G16600	MYB43	MYB transcription factor [Source:UniProtKB/TrEMBL;Acc:Q9SPG7]	5438165	5440433	32.75
AT5G16610		unknown protein; Ha. [Source:TAIR;Acc:AT5G16610]	5444760	5448355	38.99
AT5G02565			5448918	5449170	38.74
AT5G02575			5449240	5449529	34.48
AT5G16620	TIC40	TIC40 [Source:UniProtKB/TrEMBL;Acc:A0A178UEV6]	5450620	5454577	36.31
AT5G16630	RAD4	DNA repair protein RAD4 [Source:UniProtKB/Swiss-Prot;Acc:Q8W489]	5454654	5459647	38.85
AT5G16640		Pentatricopeptide repeat-containing protein At5g16640, mitochondrial [Source:UniProtKB/Swiss-Prot;Acc:Q9FMD3]	5460921	5463017	39.1
AT5G16650		Chaperone DnaJ-domain superfamily protein [Source:TAIR;Acc:AT5G16650]	5463177	5465398	35.73
AT5G16660		Low-density receptor-like protein [Source:UniProtKB/TrEMBL;Acc:Q8H0X5]	5465475	5467319	35.5
AT5G16680		RING/FYVE/PHD zinc finger superfamily protein [Source:UniProtKB/TrEMBL;Acc:F4KE59]	5467228	5473592	39.78
AT5G16690	ORC3	ORC3 [Source:UniProtKB/TrEMBL;Acc:A0A178UNG9]	5474366	5480067	36.99
AT5G16700		Glycosyl hydrolase superfamily protein [Source:UniProtKB/TrEMBL;Acc:Q9LFE7]	5480763	5483045	40.78
AT5G16710	DHAR3	DHAR3 [Source:UniProtKB/TrEMBL;Acc:A0A178UIL9]	5483260	5485185	37.8
AT5G16715	EMB2247	Valine--tRNA ligase, chloroplastic/mitochondrial 2 [Source:UniProtKB/Swiss-Prot;Acc:F4KE63]	5485203	5493548	37.1
AT5G16720	MYOB3	Myosin-binding protein 3 [Source:UniProtKB/Swiss-Prot;Acc:Q0WNW4]	5494228	5496846	39.94
AT5G16730		WEB family protein At5g16730, chloroplastic [Source:UniProtKB/Swiss-Prot;Acc:Q9LFE4]	5497524	5500985	40.24
AT5G16740	AVT1H	Amino acid transporter AVT1H [Source:UniProtKB/Swiss-Prot;Acc:Q9LFE3]	5501044	5502856	41.53
AT5G16750	TOZ	Transducin family protein / WD-40 repeat family protein [Source:UniProtKB/TrEMBL;Acc:Q9LFE2]	5504242	5509361	38.79
AT5G16760	ITPK1	Inositol-tetrakisphosphate 1-kinase [Source:UniProtKB/TrEMBL;Acc:A0A178U6K3]	5509625	5511322	40.52
AT5G16770	AtMYB9	Myb domain protein 9 [Source:UniProtKB/TrEMBL;Acc:F4KE69]	5514717	5517153	34.1
AT5G16780	DOT2	MDF [Source:UniProtKB/TrEMBL;Acc:A0A178UP47]	5515707	5522261	37.48
AT5G16790	THO7A	THO7 [Source:UniProtKB/TrEMBL;Acc:A0A178UEM6]	5522417	5524011	37.87
AT5G16800		Acyl-CoA N-acyltransferases (NAT) superfamily protein [Source:UniProtKB/TrEMBL;Acc:Q9LFD8]	5524273	5526597	37.81
AT5G16810		Protein kinase superfamily protein [Source:UniProtKB/TrEMBL;Acc:Q8GWD0]	5526689	5529978	39.33
AT5G16820	HSFA1B	Heat stress transcription factor A-1b [Source:UniProtKB/Swiss-Prot;Acc:O81821]	5530195	5532889	39.96
AT5G16830	SYP21	Syntaxin-21 [Source:UniProtKB/Swiss-Prot;Acc:Q39233]	5532846	5535295	35.8
AT5G02595			5534569	5534966	31.91
AT5G16840	BPA1	binding partner of acd11 1 [Source:TAIR;Acc:AT5G16840]	5535845	5538250	37.95
AT5G02605			5536027	5536776	34.13
AT5G16850	TERT	TERT [Source:UniProtKB/TrEMBL;Acc:A0A178UJE7]	5538180	5543657	37.92
AT5G16860	PCMP-H92	Pentatricopeptide repeat-containing protein At5g16860 [Source:UniProtKB/Swiss-Prot;Acc:Q9LFL5]	5543834	5546537	42.83
AT5G16870		At5g16870 [Source:UniProtKB/TrEMBL;Acc:Q1H5E3]	5546811	5548777	37.26
AT5G16880	TOL1	TOM1-like protein 1 [Source:UniProtKB/Swiss-Prot;Acc:Q9LFL3]	5548914	5551509	38.52
AT5G16890		Exostosin family protein [Source:UniProtKB/TrEMBL;Acc:Q9LFL2]	5551627	5554919	38.93
AT5G16900		Probable LRR receptor-like serine/threonine-protein kinase At5g16900 [Source:UniProtKB/Swiss-Prot;Acc:C0LGT5]	5555057	5559997	37.12
AT5G16910	CSLD2	CSLD2 [Source:UniProtKB/TrEMBL;Acc:A0A178UAL6]	5560607	5565660	42.38
AT5G16920		At5g16920 [Source:UniProtKB/TrEMBL;Acc:Q1PDW1]	5567000	5568128	36.85
AT5G16930		AAA-type ATPase family protein [Source:UniProtKB/TrEMBL;Acc:Q8RXI0]	5568388	5571859	41.79

	AT5G16940		Carbon-sulfur lyase [Source:UniProtKB/TrEMBL;Acc:Q9LFK7]	5571550	5572405	38.32
	AT5G16950		AT5g16950/F2K13_100 [Source:UniProtKB/TrEMBL;Acc:Q9LFK6]	5572523	5573191	40.51
	AT5G02635			5573596	5573907	43.91
	AT5G16960		Quinone oxidoreductase-like protein [Source:UniProtKB/TrEMBL;Acc:Q9LFK5]	5573700	5575910	39.12
	AT5G16970	AER	NADPH-dependent oxidoreductase 2-alkenal reductase [Source:UniProtKB/Swiss-Prot;Acc:Q39172]	5575973	5578086	38.51
	AT5G16980		Zinc-binding dehydrogenase family protein [Source:UniProtKB/TrEMBL;Acc:F4KFK3]	5578846	5581311	38.52
	AT5G16990	P2	NADP-dependent alkenal double bond reductase P2 [Source:UniProtKB/Swiss-Prot;Acc:Q39173]	5581524	5584466	37.58
	AT5G17000		AT5g16970/F2K13_120 [Source:UniProtKB/TrEMBL;Acc:Q93Z72]	5584708	5587237	38.22
	AT5G17010		D-xylose-proton symporter-like 2 [Source:UniProtKB/Swiss-Prot;Acc:Q6AWX0]	5587344	5592534	37.64
	AT5G17020	XPO1	XPO1A [Source:UniProtKB/TrEMBL;Acc:A0A178U9W2]	5594632	5602969	37.08
	AT5G17030	UGT78D3	UGT78D3 [Source:UniProtKB/TrEMBL;Acc:A0A384LMC1]	5603133	5604893	46.56
	AT5G17040	UGT78D4	UDP-glycosyltransferase 78D4 [Source:UniProtKB/Swiss-Prot;Acc:Q9LFJ9]	5605201	5607128	41.34
	AT5G17050	UGT78D2	Glycosyltransferase (Fragment) [Source:UniProtKB/TrEMBL;Acc:W8PUE7]	5607788	5609589	44.12
	AT5G17060	ATARFB1B	ADP-ribosylation factor B1B [Source:UniProtKB/TrEMBL;Acc:Q9LFJ7]	5610741	5613074	36.29
	AT5G17070		Serine/threonine-protein phosphatase 4 regulatory subunit-like protein [Source:UniProtKB/TrEMBL;Acc:Q8VY58]	5613307	5616233	37.72
	AT5G17080		Cathepsin-related [Source:UniProtKB/TrEMBL;Acc:Q9LFJ5]	5619594	5620949	37.02
	AT5G17090		Cystatin/monellin superfamily protein [Source:UniProtKB/TrEMBL;Acc:Q9LFJ4]	5621249	5622542	40.34
	AT5G17100		Cystatin/monellin superfamily protein [Source:UniProtKB/TrEMBL;Acc:Q58FU2]	5623021	5624800	38.71
	AT5G17110		Cystatin/monellin superfamily protein [Source:UniProtKB/TrEMBL;Acc:Q9LFJ2]	5626563	5627474	39.47
	AT5G17120		Cystatin/monellin superfamily protein [Source:UniProtKB/TrEMBL;Acc:Q9LFJ1]	5628529	5629324	40.33
	AT5G17130		Cysteine-type peptidase [Source:UniProtKB/TrEMBL;Acc:Q9LFJ0]	5636969	5637727	33.47
	AT5G17140		Cysteine proteinases superfamily protein [Source:UniProtKB/TrEMBL;Acc:Q9LFI9]	5637830	5638273	34.23
	AT5G17150		Cystatin/monellin superfamily protein [Source:UniProtKB/TrEMBL;Acc:F4KFM6]	5638475	5639629	38.53
MQTL-3/Chr5	AT5G19750		Peroxisomal membrane 22 kDa (Mpv17/PMP22) family protein [Source:UniProtKB/TrEMBL;Acc:F4K2L8]	6677016	6679384	39.76
	AT5G19760	DTC	Mitochondrial dicarboxylate/tricarboxylate transporter DTC [Source:UniProtKB/Swiss-Prot;Acc:Q9C5M0]	6679306	6682054	38.52
	AT5G19770	TUBA3	Tubulin alpha chain [Source:UniProtKB/TrEMBL;Acc:A0A178UTQ9]	6682420	6684744	42.71
	AT5G19780	TUBA3	Tubulin alpha chain [Source:UniProtKB/TrEMBL;Acc:A0A178UTQ9]	6687030	6689171	43.28
	AT5G19790	RAP2-11	Ethylene-responsive transcription factor RAP2-11 [Source:UniProtKB/Swiss-Prot;Acc:Q6J9S1]	6689108	6690040	43.09
	AT5G19800		Hydroxyproline-rich glycoprotein family protein [Source:UniProtKB/TrEMBL;Acc:Q3E9B1]	6690520	6691176	37.6
	AT5G19810		Proline-rich extensin-like family protein [Source:UniProtKB/TrEMBL;Acc:Q4PSF3]	6692909	6694058	46.09
	AT5G19820	emb2734	ARM repeat superfamily protein [Source:UniProtKB/TrEMBL;Acc:Q93VS8]	6695174	6701355	40.12
	AT5G19830		Peptidyl-tRNA hydrolase, mitochondrial [Source:UniProtKB/Swiss-Prot;Acc:Q6NLS8]	6703082	6705249	38.98
	AT5G19840		2-oxoglutarate (2OG) and Fe(II)-dependent oxygenase superfamily protein [Source:UniProtKB/TrEMBL;Acc:F4K2M8]	6705325	6709382	37.93
	AT5G19850		Alpha/beta-Hydrolases superfamily protein [Source:UniProtKB/TrEMBL;Acc:F4K2M9]	6709734	6711723	41.36
	AT5G19855	RBCX2	RbcX2 [Source:UniProtKB/TrEMBL;Acc:A0A178UCQ2]	6711943	6713937	39.45
	AT5G19860		At5g19860 [Source:UniProtKB/TrEMBL;Acc:Q7XA63]	6713953	6715918	39.83
	AT5G19870		At5g19870 [Source:UniProtKB/TrEMBL;Acc:Q66GS4]	6715965	6717110	39.79
	AT5G19875		At5g19875 [Source:UniProtKB/TrEMBL;Acc:Q8L9M7]	6718206	6719303	37.8
	AT5G19880	PER58	Peroxidase 58 [Source:UniProtKB/Swiss-Prot;Acc:P59120]	6720384	6722477	37.34
	AT5G19890	PER59	Peroxidase 59 [Source:UniProtKB/Swiss-Prot;Acc:Q39034]	6724076	6725966	37.07
	AT5G19900		AT5G19900 protein [Source:UniProtKB/TrEMBL;Acc:B9DF92]	6727342	6730407	39.47
	AT5G19910	MED31	SOH1 family protein [Source:TAIR;Acc:AT5G19910]	6730978	6732990	39.15
	AT5G19920		At5g19920 [Source:UniProtKB/TrEMBL;Acc:Q8GX62]	6733211	6737496	40.2
	AT5G19930	PGR	Protein PGR [Source:UniProtKB/Swiss-Prot;Acc:Q0WP96]	6737512	6739573	39.77
	AT5G19940	PAP8	Probable plastid-lipid-associated protein 8, chloroplastic [Source:UniProtKB/Swiss-Prot;Acc:Q941D3]	6739624	6740957	42.95
	AT5G19950		AT5G19950 protein [Source:UniProtKB/TrEMBL;Acc:Q8L502]	6740777	6743850	39.75
	AT5G19960		Glycine-rich RNA-binding protein, putative [Source:UniProtKB/TrEMBL;Acc:Q8RWF8]	6743878	6746381	38.74
	AT5G19970		unknown protein; Ha. [Source:TAIR;Acc:AT5G19970]	6746814	6748982	37.71
	AT5G19980	GFT1	GONST4 [Source:UniProtKB/TrEMBL;Acc:A0A384LHD5]	6749613	6751152	41.3
	AT5G19990	RPT6A	26S proteasome regulatory subunit 8 homolog A [Source:UniProtKB/Swiss-Prot;Acc:Q9C5U3]	6751798	6755315	38.06
	AT5G20000	RPT6B	26S proteasome regulatory subunit 8 homolog B [Source:UniProtKB/Swiss-Prot;Acc:Q94BQ2]	6756633	6759800	38.32
	AT5G20010	RAN1	GTP-binding nuclear protein [Source:UniProtKB/TrEMBL;Acc:A0A178UC18]	6760094	6762330	40.68
	AT5G20020	RAN2	GTP-binding nuclear protein [Source:UniProtKB/TrEMBL;Acc:A0A178UQ77]	6762569	6764683	39.57
	AT5G20030		At5g20030 [Source:UniProtKB/TrEMBL;Acc:Q8LES5]	6764727	6766702	37.15
	AT5G20040	ATIPT9	Isopentenyltransferase 9 [Source:UniProtKB/TrEMBL;Acc:F4K2Q7]	6767701	6771190	38.85
	AT5G20045		Transmembrane protein [Source:UniProtKB/TrEMBL;Acc:Q8LB93]	6771472	6772252	35.72
	AT5G20050		Probable receptor-like protein kinase At5g20050 [Source:UniProtKB/Swiss-Prot;Acc:Q94C25]	6774102	6775935	39.64
	AT5G20060		Alpha/beta-Hydrolases superfamily protein [Source:UniProtKB/TrEMBL;Acc:Q8L9X1]	6776073	6779671	36.43
	AT5G20070	NUDT19	Nudix hydrolase 19, chloroplastic [Source:UniProtKB/Swiss-Prot;Acc:Q94A82]	6779841	6782501	40.21
	AT5G20080	CBR2	NADH-cytochrome b5 reductase-like protein [Source:UniProtKB/Swiss-Prot;Acc:P83291]	6782511	6786847	36.27
	AT5G20090	MPC1	Mitochondrial pyruvate carrier [Source:UniProtKB/TrEMBL;Acc:A0A178UR79]	6786834	6788680	37.85
	AT5G20100		unknown protein; BEST Arabidopsis thaliana protein match is: unknown protein (TAIR:AT1G80180.1); Ha. [Source:TAIR;Acc:AT5G20100]	6789935	6790841	35.39
	AT5G20110		At5g20110 [Source:UniProtKB/TrEMBL;Acc:Q6NM36]	6791360	6793419	33.4
	AT5G20120		At5g20120 [Source:UniProtKB/TrEMBL;Acc:Q84VV3]	6795314	6797249	38.64
	AT5G20130		unknown protein; Ha. [Source:TAIR;Acc:AT5G20130]	6797361	6798899	41.39
	AT5G20140		SOUL heme-binding family protein [Source:UniProtKB/TrEMBL;Acc:F4K452]	6798832	6800977	39.24
	AT5G20150	SPX1	SPX domain-containing protein 1 [Source:UniProtKB/Swiss-Prot;Acc:Q8LBH4]	6802282	6803655	36.1
	AT5G20160		Ribosomal protein L7Ae/L30e/S12e/Gadd45 family protein [Source:UniProtKB/TrEMBL;Acc:F4K455]	6803694	6805226	35.49
	AT5G20165		Protein kish [Source:UniProtKB/TrEMBL;Acc:F4K459]	6805542	6807345	36.25
	AT5G20170	MED17	Mediator of RNA polymerase II transcription subunit 17 [Source:UniProtKB/Swiss-Prot;Acc:F4K460]	6807375	6810919	39.94
	AT5G20180		Ribosomal protein [Source:UniProtKB/TrEMBL;Acc:Q8W464]	6810908	6812186	38.23
	AT5G20190		Tetratricopeptide repeat (TPR)-like superfamily protein [Source:UniProtKB/TrEMBL;Acc:F4K464]	6813762	6815873	40.67
	AT5G20200		Nucleoporin-like protein [Source:UniProtKB/TrEMBL;Acc:F4K465]	6816612	6821827	39.65
	AT5G20220		zinc knuckle (CCHC-type) family protein [Source:TAIR;Acc:AT5G20220]	6821881	6825981	38.55
	AT5G20225		other RNA [Source:TAIR;Acc:AT5G20225]	6824535	6826112	38.4
	AT5G20230	BCB	SAG14 [Source:UniProtKB/TrEMBL;Acc:A0A178UM84]	6826365	6827748	39.67
	AT5G03115			6827903	6828170	31.72
	AT5G20240	PI	Floral homeotic protein PISTILLATA [Source:UniProtKB/Swiss-Prot;Acc:P48007]	6828904	6831515	33.58
	AT5G20250	DIN10	Raffinose synthase family protein [Source:UniProtKB/TrEMBL;Acc:F4K470]	6833564	6836862	43.07
	AT5G20260		Probable glycosyltransferase At5g20260 [Source:UniProtKB/Swiss-Prot;Acc:Q3E9A4]	6836781	6839609	37.58
	AT5G20270	HHP1	Heptahelical transmembrane protein 1 [Source:UniProtKB/Swiss-Prot;Acc:Q93ZH9]	6840833	6842946	36.38
	AT5G20280	SPS1	Sucrose-phosphate synthase 1 [Source:UniProtKB/Swiss-Prot;Acc:Q94BT0]	6844712	6850237	40.52
	AT5G20290	RPS8A	40S ribosomal protein S8 [Source:UniProtKB/TrEMBL;Acc:A0A178UCD3]	6851341	6853106	39.52
	AT5G20300	TOC90	Translocase of chloroplast 90, chloroplastic [Source:UniProtKB/Swiss-Prot;Acc:Q6S5G3]	6853299	6856981	40.94
	AT5G20310		Adenine nucleotide alpha hydrolases-like superfamily protein [Source:UniProtKB/TrEMBL;Acc:Q1G363]	6857553	6859145	39.55
	AT5G20320	DCL4	Dicer-like protein 4 [Source:UniProtKB/Swiss-Prot;Acc:P84634]	6859212	6869307	37.98
	AT5G20330	BG4	Probable glucan endo-1,3-beta-glucosidase BG4 [Source:UniProtKB/Swiss-Prot;Acc:O49352]	6871511	6873112	42.7
	AT5G20340	BG5	Probable glucan endo-1,3-beta-glucosidase BG5 [Source:UniProtKB/Swiss-Prot;Acc:O49353]	6874787	6875926	45.09
	AT5G20350	PAT24	Protein S-acyltransferase 24 [Source:UniProtKB/Swiss-Prot;Acc:Q52T38]	6876441	6881506	38.26
	AT5G20360	Phox3	Protein PHOX3 [Source:UniProtKB/Swiss-Prot;Acc:F4K487]	6881332	6885093	38.14
	AT5G20370		Serine-rich protein-like protein [Source:UniProtKB/TrEMBL;Acc:Q3E9A1]	6886762	6887458	43.33
	AT5G20380	PHT4;5	phosphate transporter 4;5 [Source:TAIR;Acc:AT5G20380]	6887636	6892571	37.76
	AT5G20390		Glycosyl hydrolase superfamily protein [Source:UniProtKB/TrEMBL;Acc:Q8VZ16]	6892700	6894197	39.99
	AT5G20400		2-oxoglutarate (2OG) and Fe(II)-dependent oxygenase superfamily protein [Source:UniProtKB/TrEMBL;Acc:Q84K82]	6894726	6896350	40
	AT5G20410	MGD2	Monogalactosyldiacylglycerol synthase 2, chloroplastic [Source:UniProtKB/Swiss-Prot;Acc:O82730]	6896463	6898783	40.28
	AT5G20420	CLSY2	SNF2 domain-containing protein CLASSY 2 [Source:UniProtKB/Swiss-Prot;Acc:F4K493]	6898790	6904242	39.5
	AT5G20430	MOB2B	Putative MOB kinase activator-like 2B [Source:UniProtKB/Swiss-Prot;Acc:F4K494]	6904672	6905506	36.77
	AT5G20440	MOB2A	MOB kinase activator-like 2A [Source:UniProtKB/Swiss-Prot;Acc:F4K495]	6908606	6909996	36.3
	AT5G20447		unknown protein; Ha. [Source:TAIR;Acc:AT5G20447]	6910417	6910515	36.36
	AT5G20450		CONTAINS InterPro DOMAIN/s: Dilute (InterPro:IPR002710); BEST Arabidopsis thaliana protein match is: Myosin family protein with Dil dor	6910486	6913323	36.89
	AT5G20460		unknown protein; Ha. [Source:TAIR;Acc:AT5G20460]	6914303	6914855	34.9
	AT5G20470		Myosin [Source:UniProtKB/TrEMBL;Acc:F4K5I9]	6916181	6920120	37.64
	AT5G03125			6920199	6920402	33.33
	AT5G03135			6921234	6921613	33.42
	AT5G20480	EFR	LRR receptor-like serine/threonine-protein kinase EFR [Source:UniProtKB/Swiss-Prot;Acc:C0LGT6]	6921509	6925901	39.77

AT5G03145			6926005	6926446	34.62
AT5G20490	XI-K	Myosin-17 [Source:UniProtKB/Swiss-Prot;Acc:F4K5J1]	6926516	6937830	37.23
AT5G03155			6937702	6937917	36.57
AT5G20500	GRXC4	Glutaredoxin-C4 [Source:UniProtKB/Swiss-Prot;Acc:Q8LFQ6]	6938417	6939848	38.27
AT5G03165			6938816	6939201	33.68
AT5G20510	AL5	PHD finger protein ALFIN-LIKE 5 [Source:UniProtKB/Swiss-Prot;Acc:Q5XEM9]	6939813	6943011	36.82
AT5G03175			6940253	6942769	35.4
AT5G20520	WAV2	WAV2 [Source:UniProtKB/TrEMBL;Acc:A0A178UB23]	6942955	6946514	38.23
AT5G20540	BRXL4	Protein Brevis radix-like 4 [Source:UniProtKB/Swiss-Prot;Acc:Q8GZ92]	6947789	6952038	36.52
AT5G20550		2-oxoglutarate (2OG) and Fe(II)-dependent oxygenase superfamily protein [Source:UniProtKB/TrEMBL;Acc:Q8LF12]	6952397	6953971	39.94
AT5G20560		Glycosyl hydrolase superfamily protein [Source:UniProtKB/TrEMBL;Acc:Q4PSF2]	6955190	6956474	44.05
AT5G20570	ROC1	RING-box 1 [Source:UniProtKB/TrEMBL;Acc:F4K5K0]	6956573	6958338	36.81
AT5G20580		TMEM192 family protein [Source:UniProtKB/TrEMBL;Acc:Q0WP45]	6958597	6962916	35.62
AT5G20590	TBL5	Protein trichome birefringence-like 5 [Source:UniProtKB/Swiss-Prot;Acc:F4K5K4]	6963338	6966226	40.5
AT5G20600		AT5g20600/F7C8_190 [Source:UniProtKB/TrEMBL;Acc:Q8RY12]	6966178	6968033	41.11
AT5G20610	PMIR1	Protein PLASTID MOVEMENT IMPAIRED 1-RELATED 1 [Source:UniProtKB/Swiss-Prot;Acc:F4K5K6]	6968611	6972995	41.85
AT5G20620	UBQ4	Polyubiquitin 4 [Source:UniProtKB/Swiss-Prot;Acc:P0CH32]	6973053	6975009	41.54
AT5G20630	GER3	Germin-like protein subfamily 3 member 3 [Source:UniProtKB/Swiss-Prot;Acc:P94072]	6975029	6976036	40.67
AT5G20635	GG3	Guanine nucleotide-binding protein subunit gamma 3 [Source:UniProtKB/Swiss-Prot;Acc:Q6AWT8]	6979157	6982163	34.05
AT5G20640		Protein LURP-one-related 16 [Source:UniProtKB/Swiss-Prot;Acc:A0MFH4]	6984240	6985365	37.74
AT5G20650	COPT5	Copper transporter 5 [Source:UniProtKB/Swiss-Prot;Acc:Q93VM8]	6985298	6986037	43.24
AT5G03185			6985687	6985952	48.87
AT5G20660		24 kDa vacuolar protein-like [Source:UniProtKB/TrEMBL;Acc:Q0WVZ7]	6986180	6991139	38.69
AT5G20670		At5g20670 [Source:UniProtKB/TrEMBL;Acc:A6QR87]	6993084	6993948	42.66
AT5G20680	TBL16	TBL16 [Source:UniProtKB/TrEMBL;Acc:A0A178UN54]	6997990	7002033	38.9
AT5G20690	PRK6	Pollen receptor-like kinase 6 [Source:UniProtKB/Swiss-Prot;Acc:Q3E991]	7002453	7004775	45.11
AT5G20700	FLZ14	FCS-Like Zinc finger 14 [Source:UniProtKB/Swiss-Prot;Acc:Q8GYX2]	7005667	7007502	37.64
AT5G20710	BGAL7	Beta-galactosidase 7 [Source:UniProtKB/Swiss-Prot;Acc:Q9SCV5]	7010451	7014249	39.72
AT5G20720	CPN20	20 kDa chaperonin, chloroplastic [Source:UniProtKB/Swiss-Prot;Acc:O65282]	7014662	7016698	39.47
AT5G03195			7014727	7015030	38.49
AT5G20730	NPH4	Transcriptional factor B3 family protein / auxin-responsive factor AUX/IAA-related [Source:TAIR;Acc:AT5G20730]	7016445	7022179	42.3
AT5G03205			7023444	7023923	35.21
AT5G20740		Plant invertase/pectin methylesterase inhibitor superfamily protein [Source:TAIR;Acc:AT5G20740]	7025565	7026753	45.42
AT5G20790		unknown protein; FUNCTIONS IN: molecular_function unknown; INVOLVED IN: biological_process unknown; LOCATED IN: endomembrane	7039674	7040919	42.38
AT5G20810		SAUR-like auxin-responsive protein family [Source:UniProtKB/TrEMBL;Acc:Q2V359]	7043758	7045830	37
AT5G03215			7045917	7046151	42.13
AT5G20820	SAUR76	Auxin-responsive protein SAUR76 [Source:UniProtKB/Swiss-Prot;Acc:Q29PU2]	7046747	7047446	43.86
AT5G20830	SUS1	Sucrose synthase 1 [Source:UniProtKB/Swiss-Prot;Acc:P49040]	7050217	7055398	38.21
AT5G03225			7057157	7057231	42.67
AT5G20840	SAC4	Phosphoinositide phosphatase SAC4 [Source:UniProtKB/Swiss-Prot;Acc:Q7XZU1]	7060992	7069343	37.08
AT5G20850	RAD51	DNA repair protein RAD51 homolog 1 [Source:UniProtKB/Swiss-Prot;Acc:P94102]	7070491	7072968	40.56
AT5G20852		pre-tRNA [Source:TAIR;Acc:AT5G20852]	7073530	7073601	54.17
AT5G20854	ATC2	ATC2; pre-tRNA [Source:TAIR;Acc:AT5G20854]	7073943	7074014	54.17
AT5G20856		pre-tRNA [Source:TAIR;Acc:AT5G20856]	7074356	7074427	51.39
AT5G20858		pre-tRNA [Source:TAIR;Acc:AT5G20858]	7074772	7074843	52.78
AT5G20860		Plant invertase/pectin methylesterase inhibitor superfamily [Source:TAIR;Acc:AT5G20860]	7076735	7079149	39.75
AT5G20870		O-Glycosyl hydrolases family 17 protein [Source:UniProtKB/TrEMBL;Acc:F4K6W1]	7079799	7081789	44.25
AT5G20885		AT5g20880/F22D1_50 [Source:UniProtKB/TrEMBL;Acc:Q93ZD8]	7083408	7084859	40.01
AT5G20890	CCT2	T-complex protein 1 subunit beta [Source:UniProtKB/Swiss-Prot;Acc:Q940P8]	7086653	7090169	39.44
AT5G20900	TIFY3B	Protein TIFY 3B [Source:UniProtKB/Swiss-Prot;Acc:Q9C5K8]	7090704	7092539	37.64
AT5G03235			7091915	7092125	28.91
AT5G20910	AIP2	E3 ubiquitin-protein ligase AIP2 [Source:UniProtKB/Swiss-Prot;Acc:Q8RXD3]	7092512	7094503	38.4
AT5G03245			7094154	7094423	39.63
AT5G20920	EIF2B	Eukaryotic translation initiation factor 2 subunit beta [Source:UniProtKB/Swiss-Prot;Acc:Q41969]	7094649	7097145	36.68
AT5G20930	TOUSLED	Serine/threonine-protein kinase TOUSLED [Source:UniProtKB/Swiss-Prot;Acc:Q39238]	7097868	7103206	37.03
AT5G20935		unknown protein; CONTAINS InterPro DOMAIN/s: Protein of unknown function DUF3148 (InterPro:IPR021495); Ha. [Source:TAIR;Acc:AT5G	7103384	7103945	40.75
AT5G20940		Beta-glucosidase-like protein [Source:UniProtKB/TrEMBL;Acc:Q8RWM4]	7103927	7106834	43.05
AT5G03255			7104031	7106966	43.09
AT5G20950		Glycosyl hydrolase family protein [Source:TAIR;Acc:AT5G20950]	7107272	7111453	41.03
AT5G20960	AAO1	Indole-3-acetaldehyde oxidase [Source:UniProtKB/Swiss-Prot;Acc:Q7G193]	7116455	7122774	39.38
AT5G03265			7122566	7122688	22.76
AT5G20970		HSP20-like chaperones superfamily protein [Source:UniProtKB/TrEMBL;Acc:F4K6X6]	7122800	7124001	39.1
AT5G20980	MS3	5-methyltetrahydropteroyltriglutamate--homocysteine methyltransferase 3, chloroplastic [Source:UniProtKB/Swiss-Prot;Acc:Q0WNZ5]	7122800	7128412	40
AT5G20990	CNX1	Molybdopterin biosynthesis protein CNX1 [Source:UniProtKB/Swiss-Prot;Acc:Q39054]	7128475	7133512	38.94
AT5G20995			7134316	7135004	35.12
AT5G03275			7134754	7135261	36.42
AT5G21010	BPM5	BTB/POZ and MATH domain-containing protein 5 [Source:UniProtKB/Swiss-Prot;Acc:Q1EBV6]	7135961	7138711	39.48
AT5G21020		At5g21020 [Source:UniProtKB/TrEMBL;Acc:Q3E985]	7138762	7139661	41.67
AT5G21030		PAZ domain-containing protein / piwi domain-containing protein [Source:TAIR;Acc:AT5G21030]	7139817	7144272	36.56
AT5G21040	FBX2	FBX2 [Source:UniProtKB/TrEMBL;Acc:A0A178URR1]	7144829	7147277	42.02
AT5G21050		LOCATED IN: chloroplast; EXPRESSED IN: 14 plant structures; EXPRESSED DURING: 8 growth stages; CONTAINS InterPro DOMAIN/s: H	7147890	7148957	49.63
AT5G21060		Homoserine dehydrogenase [Source:UniProtKB/TrEMBL;Acc:F4K6Y8]	7148978	7152922	37.97
AT5G21070		AT5g21070/T10F18_100 [Source:UniProtKB/TrEMBL;Acc:Q940N6]	7156063	7157889	40.07
AT5G21080		Uncharacterized protein [Source:TAIR;Acc:AT5G21080]	7157803	7163188	38.15
AT5G03295			7163708	7163953	39.02
AT5G21090	LRR1	Leucine-rich repeat protein 1 [Source:UniProtKB/Swiss-Prot;Acc:Q9FPJ5]	7164560	7167351	36.39
AT5G21100		Ascorbate oxidase-like protein [Source:UniProtKB/TrEMBL;Acc:Q8LPL3]	7168106	7170961	39.81
AT5G03305			7168870	7168969	23
AT5G21105		Plant L-ascorbate oxidase [Source:UniProtKB/TrEMBL;Acc:F4K6Z5]	7172604	7177985	34.97
AT5G21120	EIL2	ETHYLENE-INSENSITIVE3-like 2 [Source:TAIR;Acc:AT5G21120]	7181926	7184365	39.14
AT5G21125		Putative uncharacterized protein [Source:UniProtKB/TrEMBL;Acc:Q84VP7]	7185048	7185428	41.47
AT5G21130		Late embryogenesis abundant (LEA) hydroxyproline-rich glycoprotein family [Source:UniProtKB/TrEMBL;Acc:Q84VP8]	7185897	7186919	44.09
AT5G21140	emb1379	At5g21140 [Source:UniProtKB/TrEMBL;Acc:Q84VP9]	7187096	7189696	36.76
AT5G21150	AGO9	Protein argonaute 9 [Source:UniProtKB/Swiss-Prot;Acc:Q84VQ0]	7192239	7198381	38.35
AT5G21160		LA RNA-binding protein [Source:UniProtKB/TrEMBL;Acc:F4K704]	7198633	7204097	38.87
AT5G21170	AKINBETA1	5'-AMP-activated protein kinase beta-2 subunit protein [Source:UniProtKB/TrEMBL;Acc:Q2V357]	7205477	7208613	39.46
AT5G21222	ATC401	Pentatricopeptide repeat-containing protein At5g21222 [Source:UniProtKB/Swiss-Prot;Acc:Q8S9D1]	7208643	7213915	39.14
AT5G21274	CAM6	CAM6 [Source:UniProtKB/TrEMBL;Acc:A0A178UHI2]	7214474	7216052	37.18
AT5G21326	CIPK26	Non-specific serine/threonine protein kinase [Source:UniProtKB/TrEMBL;Acc:A0A178URQ5]	7217244	7222021	36.58
AT5G21378		pre-tRNA [Source:TAIR;Acc:AT5G21378]	7222057	7222128	61.11
AT5G21430	ndhU	NdhU [Source:UniProtKB/TrEMBL;Acc:A0A178UN62]	7222179	7223639	38.06
AT5G21482	CKX7	Cytokinin dehydrogenase 7 [Source:UniProtKB/Swiss-Prot;Acc:Q9FUJ1]	7226606	7230304	39.55
AT5G21900		At5g21900 [Source:UniProtKB/TrEMBL;Acc:Q9C597]	7238174	7240535	37.93
AT5G21910		Putative uncharacterized protein [Source:UniProtKB/TrEMBL;Acc:Q5XV37]	7240659	7241746	40.72
AT5G21920	YLMG2	YlmG homolog protein 2, chloroplastic [Source:UniProtKB/Swiss-Prot;Acc:Q9C595]	7241201	7242880	40.24
AT5G03315			7241654	7241840	30.48
AT5G21930	PAA2	Copper-transporting ATPase PAA2, chloroplastic [Source:UniProtKB/Swiss-Prot;Acc:B9DFX7]	7243002	7249170	39
AT5G21940		At5g21940 [Source:UniProtKB/TrEMBL;Acc:Q9C593]	7249014	7250770	38.42
AT5G21950		alpha/beta-Hydrolases superfamily protein [Source:TAIR;Acc:AT5G21950]	7253849	7256156	36.18
AT5G21960	ERF016	Ethylene-responsive transcription factor ERF016 [Source:UniProtKB/Swiss-Prot;Acc:Q9C591]	7258037	7259568	39.49
AT5G21970		Expressed protein [Source:UniProtKB/TrEMBL;Acc:Q9C590]	7261523	7263644	40.29
AT5G21280		At5g21280 [Source:UniProtKB/TrEMBL;Acc:Q9C589]	7263828	7265874	37.76
AT5G03325			7270934	7271458	39.43
AT5G03335			7271223	7271528	47.39
AT5G21990	OEP61	TPR7 [Source:UniProtKB/TrEMBL;Acc:A0A178UNB9]	7273071	7276579	38.53

	AT5G22000	RHF2A	RHF2A [Source:UniProtKB/TrEMBL;Acc:A0A178UA98]	7277182	7280344	39.2
	AT5G22010	RFC1	Replication factor C subunit 1 [Source:UniProtKB/Swiss-Prot;Acc:Q9C587]	7280321	7287434	38.71
	AT5G22020		At5g22020 [Source:UniProtKB/TrEMBL;Acc:Q9C586]	7287628	7289646	40.42
	AT5G22030	UBP8	ubiquitin-specific protease 8 [Source:TAIR;Acc:AT5G22030]	7289703	7296344	38.09
	AT5G22040		At5g22040 [Source:UniProtKB/TrEMBL;Acc:Q9C584]	7296915	7299313	40.43
	AT5G22050		Protein kinase superfamily protein [Source:UniProtKB/TrEMBL;Acc:Q67YR4]	7301224	7303876	37.43
	AT5G22060	ATJ2	At5g22060 [Source:UniProtKB/TrEMBL;Acc:Q0V7U1]	7303506	7305870	40.85
	AT5G22070		Core-2/I-branching beta-1,6-N-acetylglucosaminyltransferase family protein [Source:UniProtKB/TrEMBL;Acc:Q9C581]	7307894	7310367	40.02
	AT5G03375			7308937	7309219	44.88
	AT5G22080		Chaperone DnaJ-domain superfamily protein [Source:UniProtKB/TrEMBL;Acc:Q9C580]	7310171	7313780	36.98
	AT5G22090		Protein FAF-like, chloroplastic [Source:UniProtKB/Swiss-Prot;Acc:Q0V865]	7314583	7321743	31.99
	AT5G03385			7321511	7322183	34.47
	AT5G22100		RNA 3'-terminal phosphate cyclase-like protein [Source:UniProtKB/TrEMBL;Acc:Q67XN9]	7328870	7330930	38.96
	AT5G22110	DPB2	DNA polymerase epsilon subunit B [Source:UniProtKB/Swiss-Prot;Acc:Q500V9]	7330825	7334282	38.2
	AT5G22120		At5g22100/At5g22100 [Source:UniProtKB/TrEMBL;Acc:Q9C576]	7334314	7337018	38.19
	AT5G22130	PNT1	Mannosyltransferase family protein [Source:UniProtKB/TrEMBL;Acc:F4K8F7]	7337158	7340052	38.17
	AT5G22140		FAD/NAD(P)-binding oxidoreductase family protein [Source:UniProtKB/TrEMBL;Acc:Q9C574]	7340055	7341959	38.01
	AT5G22145			7340426	7341028	40.3
	AT5G22150		Transmembrane protein [Source:UniProtKB/TrEMBL;Acc:Q9C573]	7342565	7346061	33.94
	AT5G22160		Transmembrane protein [Source:UniProtKB/TrEMBL;Acc:Q9C572]	7346944	7349255	37.41
	AT5G22170		Transmembrane protein [Source:UniProtKB/TrEMBL;Acc:Q9C571]	7350575	7351190	45.78
	AT5G22180		Uncharacterized protein At5g22180 [Source:UniProtKB/TrEMBL;Acc:Q9C570]	7353070	7353706	46.15
	AT5G22190		Uncharacterized protein At5g22190 [Source:UniProtKB/TrEMBL;Acc:Q9C569]	7354164	7354688	41.9
	AT5G22200		Late embryogenesis abundant (LEA) hydroxyproline-rich glycoprotein family [Source:UniProtKB/TrEMBL;Acc:Q9C568]	7355570	7357122	38.18
	AT5G22210		Aspartyl/glutamyl-tRNA (Asn/Gln) amidotransferase subunit B [Source:UniProtKB/TrEMBL;Acc:Q9C567]	7358778	7360051	35.09
	AT5G22220	E2FB	Transcription factor E2FB [Source:UniProtKB/Swiss-Prot;Acc:Q9FV71]	7360535	7364505	38.55
	AT5G22240	OFP10	Transcription repressor OFP10 [Source:UniProtKB/Swiss-Prot;Acc:Q9FMS7]	7364689	7365426	39.84
	AT5G22250	CAF1-11	Probable CCR4-associated factor 1 homolog 11 [Source:UniProtKB/Swiss-Prot;Acc:Q9FMS6]	7365477	7366799	42.55
	AT5G22260	MS1	PHD finger protein MALE STERILITY 1 [Source:UniProtKB/Swiss-Prot;Acc:Q9FMS5]	7367635	7370295	40.06
	AT5G22270		AT5G22270 protein [Source:UniProtKB/TrEMBL;Acc:Q9FMS4]	7372109	7373259	31.36
	AT5G22280		unknown protein; FUNCTIONS IN: molecular_function unknown; INVOLVED IN: biological_process unknown; LOCATED IN: endomembrane	7373963	7375794	35.75
	AT5G22290	NAC089	NAC domain-containing protein 89 [Source:UniProtKB/Swiss-Prot;Acc:Q94F58]	7375839	7377776	41.28
	AT5G22300	NIT4	Bifunctional nitrilase/nitrile hydratase NIT4 [Source:UniProtKB/Swiss-Prot;Acc:P46011]	7379187	7382025	39.94
	AT5G22310		AT5g22310/MWD9_9 [Source:UniProtKB/TrEMBL;Acc:Q93ZM2]	7383583	7385833	39.63
	AT5G22315		pre-tRNA [Source:TAIR;Acc:AT5G22315]	7387888	7387959	54.17
	AT5G22320		AT5g22320/MWD9_11 [Source:UniProtKB/TrEMBL;Acc:Q9FMS0]	7387985	7390613	37.35
	AT5G22330	RIN1	RuvB-like protein 1 [Source:UniProtKB/Swiss-Prot;Acc:Q9FMR9]	7390796	7394280	37.62
	AT5G22340		unknown protein; FUNCTIONS IN: molecular_function unknown; INVOLVED IN: biological_process unknown; LOCATED IN: chloroplast; EX	7394443	7397320	38.6
	AT5G22350	ELM1	Mitochondrial fission protein ELM1 [Source:UniProtKB/Swiss-Prot;Acc:Q93YN4]	7397386	7401290	38.31
	AT5G22355		Cysteine/Histidine-rich C1 domain family protein [Source:UniProtKB/TrEMBL;Acc:Q9FMR6]	7401707	7403972	41.09
	AT5G22360	VAMP714	Vesicle-associated membrane protein 714 [Source:UniProtKB/Swiss-Prot;Acc:Q9FMR5]	7404004	7405883	37.34
	AT5G22370	EMB1705	QQT1 [Source:UniProtKB/TrEMBL;Acc:A0A178UBM9]	7406030	7408754	36.55
	AT5G22380	NAC090	NAC-domain protein-like [Source:UniProtKB/TrEMBL;Acc:Q680R1]	7408759	7410102	38.54
	AT5G22390		FANTASTIC four-like protein (DUF3049) [Source:UniProtKB/TrEMBL;Acc:Q9FMR2]	7418095	7419335	39.89
	AT5G03395			7421048	7421568	34.74
	AT5G22400	ROPGAP1	Rho GTPase-activating protein 1 [Source:UniProtKB/Swiss-Prot;Acc:Q9FMR1]	7422670	7425574	39.31
	AT5G22410	PER60	Peroxidase 60 [Source:UniProtKB/Swiss-Prot;Acc:Q9FMR0]	7426310	7427964	35.95
	AT5G22420	FAR7	Putative fatty acyl-CoA reductase 7 [Source:UniProtKB/Swiss-Prot;Acc:Q9FMQ9]	7429769	7432825	33.2
	AT5G22430		Pollen Ole e 1 allergen and extensin family protein [Source:UniProtKB/TrEMBL;Acc:Q9FMQ8]	7433790	7434953	36.77
	AT5G22440	RPL10AC	60S ribosomal protein L10a-3 [Source:UniProtKB/Swiss-Prot;Acc:P59231]	7435065	7436769	36.77
	AT5G22450		unknown protein; LOCATED IN: chloroplast; EXPRESSED IN: 22 plant structures; EXPRESSED DURING: 13 growth stages; BEST Arabidops	7436905	7443463	39.29
	AT5G22460		Alpha/beta-Hydrolases superfamily protein [Source:UniProtKB/TrEMBL;Acc:Q9FMQ5]	7443460	7445639	35.28
	AT5G22470		NAD+ ADP-ribosyltransferases;NAD+ ADP-ribosyltransferases [Source:TAIR;Acc:AT5G22470]	7446962	7451049	39.65
	AT5G22480		ZPR1 zinc-finger domain protein [Source:UniProtKB/TrEMBL;Acc:F4K9W6]	7451503	7456865	37.16
	AT5G22490		Emb [Source:UniProtKB/TrEMBL;Acc:Q9FK89]	7458774	7461093	37.16
	AT5G03405			7462508	7462864	34.45
	AT5G22500	FAR1	Fatty acyl-CoA reductase 1 [Source:UniProtKB/Swiss-Prot;Acc:Q39152]	7470328	7474133	33.32
	AT5G22505			7472540	7473849	35.42
	AT5G22510	INVE	Alkaline/neutral invertase E, chloroplastic [Source:UniProtKB/Swiss-Prot;Acc:Q9FK88]	7474635	7478335	39.29
	AT5G03425			7478569	7478673	44.76
	AT5G22520		At5g22520 [Source:UniProtKB/TrEMBL;Acc:Q9FK87]	7478860	7479543	34.65
	AT5G22530		Uncharacterized protein At5g22530 [Source:UniProtKB/TrEMBL;Acc:Q9FK86]	7480413	7481206	38.16
	AT5G22540		At5g22540 [Source:UniProtKB/TrEMBL;Acc:Q9FK85]	7481397	7482953	37.51
	AT5G22545		Uncharacterized protein At5g22545 [Source:UniProtKB/TrEMBL;Acc:Q8GWC9]	7483159	7483861	35.14
	AT5G22550		Emb [Source:UniProtKB/TrEMBL;Acc:Q9FK84]	7483863	7485482	39.26
MQTL-4/Chr5	AT5G24770	VSP2	Vegetative storage protein 2 [Source:UniProtKB/Swiss-Prot;Acc:O82122]	8500468	8502224	37.91
	AT5G24780	VSP1	Vegetative storage protein 1 [Source:UniProtKB/Swiss-Prot;Acc:O49195]	8507590	8509201	39.58
	AT5G24790		Uncharacterized protein AT5g24790 [Source:UniProtKB/TrEMBL;Acc:Q9FT41]	8512042	8513218	39.93
	AT5G24800	BZIP9	Basic leucine zipper 9 [Source:UniProtKB/Swiss-Prot;Acc:Q9FUD3]	8515200	8516669	40.34
	AT5G24810		ABC1 family protein [Source:UniProtKB/TrEMBL;Acc:F4KIJ2]	8516635	8522800	38.11
	AT5G03825			8520898	8521247	36.29
	AT5G24820		Aspartyl protease family protein [Source:UniProtKB/TrEMBL;Acc:Q4PSE9]	8523390	8525356	38.64
	AT5G24825	MIR169B	MIR169B; miRNA [Source:TAIR;Acc:AT5G24825]	8527469	8527649	40.88
	AT5G24830		Pentatricopeptide repeat-containing protein At5g24830 [Source:UniProtKB/Swiss-Prot;Acc:Q8L6Y3]	8530915	8533867	38.91
	AT5G03835			8530939	8531228	38.97
	AT5G24840		tRNA (guanine-N(7)-)-methyltransferase [Source:UniProtKB/Swiss-Prot;Acc:Q8GXB7]	8533787	8535043	39.14
	AT5G24850	CRYD	Cryptochrome DASH, chloroplastic/mitochondrial [Source:UniProtKB/Swiss-Prot;Acc:Q84KJ5]	8535258	8538211	38.83
	AT5G24860	FPF1	flowering promoting factor 1 [Source:TAIR;Acc:AT5G24860]	8541558	8542473	38.54
	AT5G24870		At5g24870 [Source:UniProtKB/TrEMBL;Acc:Q8L775]	8544682	8547906	38.67
	AT5G24879		unknown protein; Ha. [Source:TAIR;Acc:AT5G24879]	8552491	8552604	35.96
	AT5G24880		Chromo domain cec-like protein [Source:UniProtKB/TrEMBL;Acc:Q3E966]	8552647	8554275	40.09
	AT5G24890		At5g24890 [Source:UniProtKB/TrEMBL;Acc:Q8L9W8]	8557737	8559488	34.36
	AT5G24900	CYP714A2	Cytochrome P450 714A2 [Source:UniProtKB/Swiss-Prot;Acc:Q6NKZ8]	8563800	8566812	36.74
	AT5G24910	CYP714A1	Cytochrome P450 714A1 [Source:UniProtKB/Swiss-Prot;Acc:Q93Z79]	8567504	8570367	38.79
	AT5G03845			8580763	8581113	31.34
	AT5G24920	GDU5	Protein GLUTAMINE DUMPER 5 [Source:UniProtKB/Swiss-Prot;Acc:Q3E965]	8584535	8585297	39.32
	AT5G24930	COL4	Zinc finger protein CONSTANS-LIKE 4 [Source:UniProtKB/Swiss-Prot;Acc:Q940T9]	8589457	8590949	44.14
	AT5G24940		Probable protein phosphatase 2C 71 [Source:UniProtKB/Swiss-Prot;Acc:Q4PSE8]	8591143	8593601	38.47
	AT5G24950	CYP71A15	Cytochrome P450 71A15 [Source:UniProtKB/Swiss-Prot;Acc:P58046]	8595209	8597761	35.33
	AT5G24960	CYP71A14	Cytochrome P450 71A14 [Source:UniProtKB/Swiss-Prot;Acc:P58045]	8599735	8603321	32.59
	AT5G24970		Protein kinase superfamily protein [Source:UniProtKB/TrEMBL;Acc:F4KIL1]	8604288	8608883	38.77
	AT5G24980		At5g24980 [Source:UniProtKB/TrEMBL;Acc:Q6NQ43]	8609023	8610005	37.13
	AT5G24990		Enhanced disease resistance-like protein (DUF1336) [Source:UniProtKB/TrEMBL;Acc:Q94AK1]	8610313	8611948	41.99
	AT5G25000		unknown protein; Ha. [Source:TAIR;Acc:AT5G25000]	8613966	8614331	37.16
	AT5G25010		Protein of unknown function (DUF1336) [Source:TAIR;Acc:AT5G25010]	8615778	8617209	40.01
	AT5G25020		Protein of unknown function (DUF1336) [Source:TAIR;Acc:AT5G25020]	8618404	8619465	44.35
	AT5G25030		Domain of unknown function (DUF2431) [Source:TAIR;Acc:AT5G25030]	8620083	8620991	33.88
	AT5G25040		Major facilitator superfamily protein [Source:TAIR;Acc:AT5G25040]	8621900	8624484	38.22
	AT5G25050		Probable folate-biopterin transporter 2 [Source:UniProtKB/Swiss-Prot;Acc:Q5FV41]	8631461	8634027	38.92
	AT5G25060	RRC1	Protein RRC1 [Source:UniProtKB/Swiss-Prot;Acc:Q9C5J3]	8633114	8640217	39.79
	AT5G25070		Neurofilament light protein [Source:UniProtKB/TrEMBL;Acc:Q8L4Q6]	8640754	8643516	42.38
	AT5G25080		AT5g25080/T11H3_90 [Source:UniProtKB/TrEMBL;Acc:Q93VA9]	8643662	8647058	36.47
	AT5G03875			8644914	8645262	34.67
	AT5G25090	ENODL13	ENODL13 [Source:UniProtKB/TrEMBL;Acc:A0A178U9R8]	8647018	8647817	41.25
	AT5G25100		Transmembrane 9 superfamily member [Source:UniProtKB/TrEMBL;Acc:F4KIM7]	8648006	8651176	40.71

	AT5G25110	CIPK25	CBL-interacting serine/threonine-protein kinase 25 [Source:UniProtKB/Swiss-Prot;Acc:Q8W1D5]	8657604	8659352	40.71
	AT5G03915			8660760	8661215	29.39
	AT5G25120	CYP71B11	Cytochrome P450 71B11 [Source:UniProtKB/Swiss-Prot;Acc:P58049]	8661970	8664681	34.11
	AT5G03925			8666822	8667260	48.29
	AT5G25130	CYP71B12	Cytochrome P450 71B12 [Source:UniProtKB/Swiss-Prot;Acc:Q9ZU07]	8668232	8670237	36.49
	AT5G25140	CYP71B13	Cytochrome P450 71B13 [Source:UniProtKB/Swiss-Prot;Acc:P58050]	8672316	8674749	35.41
	AT5G25150	TAF5	Transcription initiation factor TFIID subunit 5 [Source:UniProtKB/Swiss-Prot;Acc:Q6S7B0]	8677049	8682344	37.99
	AT5G25160	ZFP3	ZFP3 zinc finger protein [Source:UniProtKB/TrEMBL;Acc:Q0WT56]	8687188	8688415	38.68
	AT5G25170		At5g25170 [Source:UniProtKB/TrEMBL;Acc:Q6NQ19]	8693012	8695095	36.04
	AT5G25180	CYP71B14	Cytochrome P450 71B14 [Source:UniProtKB/Swiss-Prot;Acc:P58051]	8694630	8697108	34.57
	AT5G25190	ERF003	Ethylene-responsive transcription factor ERF003 [Source:UniProtKB/Swiss-Prot;Acc:Q94AW5]	8706745	8707885	40.75
	AT5G25195		F-box/associated interaction domain protein [Source:UniProtKB/TrEMBL;Acc:A0A1P8BAX2]	8716180	8716423	36.48
	AT5G25200		unknown protein; BEST Arabidopsis thaliana protein match is: unknown protein (TAIR:AT5G18636.1); Ha. [Source:TAIR;Acc:AT5G25200]	8716766	8717869	42.57
	AT5G25210		unknown protein; BEST Arabidopsis thaliana protein match is: unknown protein (TAIR:AT4G32030.1); Ha. [Source:TAIR;Acc:AT5G25210]	8723912	8725817	35.47
	AT5G03935			8726738	8727075	36.69
	AT5G25220	KNAT3	KNAT3 [Source:UniProtKB/TrEMBL;Acc:A0A178U9D1]	8735944	8738601	39.43
	AT5G25230	GFL	109 kDa U5 small nuclear ribonucleoprotein component GFL [Source:UniProtKB/Swiss-Prot;Acc:F4JWP9]	8739545	8743880	41.37
	AT5G25240		Stress induced protein [Source:UniProtKB/TrEMBL;Acc:Q8GYN8]	8746594	8747290	36.87
	AT5G03945			8748171	8748372	35.15
	AT5G25250	FLOT1	Flotillin-like protein 1 [Source:UniProtKB/Swiss-Prot;Acc:Q501E6]	8749680	8751636	41.13
	AT5G25260	FLOT2	Flotillin-like protein 2 [Source:UniProtKB/Swiss-Prot;Acc:Q4V3D6]	8752684	8754522	43.77
	AT5G25265	HPAT1	Hydroxyproline O-arabinosyltransferase 1 [Source:UniProtKB/Swiss-Prot;Acc:Q8W4E6]	8754181	8756948	37.97
	AT5G25270		Ubiquitin-like superfamily protein [Source:TAIR;Acc:AT5G25270]	8757434	8763005	38.85
	AT5G25280		AT5g25280/F18G18_20 [Source:UniProtKB/TrEMBL;Acc:Q93WB2]	8773734	8776594	36.95
	AT5G03955			8776926	8777236	35.37
	AT5G25290		F-box protein At5g25290 [Source:UniProtKB/Swiss-Prot;Acc:Q4PSE7]	8778592	8779839	40.38
	AT5G25300		Probable F-box protein At5g25300 [Source:UniProtKB/Swiss-Prot;Acc:Q3E960]	8780258	8783093	37.27
	AT5G25310		Probable glycosyltransferase At5g25310 [Source:UniProtKB/Swiss-Prot;Acc:Q3E7Q9]	8784747	8787366	35.76
	AT5G25320	ACR2	ACT domain-containing protein ACR2 [Source:UniProtKB/Swiss-Prot;Acc:F4JWR0]	8787196	8789846	40.1
MQTL-5/Chr5	AT5G34581		Hydroxyproline-rich glycoprotein family protein [Source:UniProtKB/TrEMBL;Acc:F4KIN3]	12920366	12921018	43.03
	AT5G34780	KPR	Putative 2-dehydropantoate 2-reductase [Source:UniProtKB/Swiss-Prot;Acc:F4KIN4]	12961682	12963892	37.27
	AT5G34830		unknown protein; Ha. [Source:TAIR;Acc:AT5G34830]	12981645	12982497	36.11
	AT5G34829		unknown protein; FUNCTIONS IN: molecular_function unknown; INVOLVED IN: biological_process unknown; LOCATED IN: cellular_compo	12982671	12982889	42.47
	AT5G34869		Putative uncharacterized protein [Source:UniProtKB/TrEMBL;Acc:Q1G374]	13061035	13062230	43.81
	AT5G34871		other RNA [Source:TAIR;Acc:AT5G34871]	13085037	13086565	35.64
	AT5G34828		Plant thionin family protein [Source:UniProtKB/TrEMBL;Acc:A8MRM7]	13094069	13094344	34.78
	AT5G05125			13099669	13099924	42.19
	AT5G05135			13099779	13100086	37.01
	AT5G34850	PAP26	Purple acid phosphatase [Source:UniProtKB/TrEMBL;Acc:A0A178UD94]	13107626	13111656	38.53
	AT5G34870		Zinc knuckle (CCHC-type) family protein [Source:UniProtKB/TrEMBL;Acc:F4JWU5]	13185521	13187946	45.01
	AT5G34882		Protein of unknown function (DUF784) [Source:TAIR;Acc:AT5G34882]	13195705	13196052	35.34
	AT5G34881		Protein of unknown function (DUF784) [Source:TAIR;Acc:AT5G34881]	13197604	13198096	33.06
	AT5G34883		Protein of unknown function (DUF784) [Source:TAIR;Acc:AT5G34883]	13199562	13199945	34.64
	AT5G34885		Protein of unknown function (DUF784) [Source:TAIR;Acc:AT5G34885]	13201475	13202185	28.83
	AT5G34887		Protein of unknown function (DUF784) [Source:TAIR;Acc:AT5G34887]	13203442	13203841	34.5
	AT5G34905		Protein of unknown function (DUF784) [Source:TAIR;Acc:AT5G34905]	13223069	13223493	32.71
	AT5G34908		Protein of unknown function (DUF784) [Source:TAIR;Acc:AT5G34908]	13225067	13225315	35.74
	AT5G34930		arogenate dehydrogenase [Source:TAIR;Acc:AT5G34930]	13233357	13235763	41.55
	AT5G34940	AtGUS3	Heparanase-like protein 3 [Source:UniProtKB/Swiss-Prot;Acc:Q9FZP1]	13235741	13238884	36.93
	AT5G05155			13242884	13243248	37.81
MQTL-6/Chr5	AT5G38610		At5g38610 [Source:UniProtKB/TrEMBL;Acc:Q9FFW0]	15460691	15461459	42.13
	AT5G38620		MADS-box protein AGL73 [Source:UniProtKB/TrEMBL;Acc:Q9FFV9]	15463858	15464907	42.57
	AT5G38630	CYB561B	Probable transmembrane ascorbate ferrireductase 2 [Source:UniProtKB/Swiss-Prot;Acc:Q9SWS1]	15465992	15468185	39.24
	AT5G38640		At5g38640 [Source:UniProtKB/TrEMBL;Acc:Q9FFV8]	15467679	15470724	42.51
	AT5G38660	APE1	Acclimation of photosynthesis to environment [Source:UniProtKB/TrEMBL;Acc:A0A219HZL6]	15470900	15476553	37.42
	AT5G38650		Gb [Source:UniProtKB/TrEMBL;Acc:Q9FFV7]	15470961	15472815	35.47
	AT5G38670		F-box/kelch-repeat protein At5g38670 [Source:UniProtKB/Swiss-Prot;Acc:Q9FFV5]	15475786	15476871	44.2
	AT5G38680		Putative F-box/kelch-repeat protein At5g38680 [Source:UniProtKB/Swiss-Prot;Acc:Q9FFV4]	15477324	15479040	41.29
	AT5G38690		AT5g38690/MBB18_24 [Source:UniProtKB/TrEMBL;Acc:Q8RW95]	15478701	15483250	35.63
	AT5G38700		Cotton fiber protein [Source:UniProtKB/TrEMBL;Acc:Q570M0]	15489593	15490745	38.25
	AT5G38710	POX2	Proline dehydrogenase 2, mitochondrial [Source:UniProtKB/Swiss-Prot;Acc:Q6NKX1]	15501126	15504269	37.12
	AT5G38720		unknown protein; Ha. [Source:TAIR;Acc:AT5G38720]	15508196	15510713	36.81
	AT5G38730		Pentatricopeptide repeat-containing protein At5g38730 [Source:UniProtKB/Swiss-Prot;Acc:Q9FKR3]	15510842	15512934	37.89
	AT5G38740	AGL77	AGAMOUS-like 77 [Source:UniProtKB/TrEMBL;Acc:Q9FKR2]	15512943	15514305	39.84
	AT5G05785			15515303	15515799	37.83
	AT5G38743		Uncharacterized protein (Fragment) [Source:UniProtKB/TrEMBL;Acc:A0A1P8BHB4]	15517299	15517796	35.34
	AT5G05805			15518146	15518499	31.92
	AT5G38747		Uncharacterized protein (Fragment) [Source:UniProtKB/TrEMBL;Acc:A0A1P8BHB2]	15518329	15521281	36.44
	AT5G05815			15520600	15520994	36.96
	AT5G38750		Asparaginyl-tRNA synthetase family [Source:UniProtKB/TrEMBL;Acc:F4KBM0]	15522650	15523180	46.14
	AT5G38760		At5g38760 [Source:UniProtKB/TrEMBL;Acc:Q9FKR1]	15524173	15525045	31.27
	AT5G38770	GDU7	GDU7 [Source:UniProtKB/TrEMBL;Acc:A0A178UB13]	15526065	15526575	36.99
	AT5G05835			15526742	15527147	38.67
	AT5G05825			15526742	15527147	38.67
	AT5G38780		Probable S-adenosylmethionine-dependent methyltransferase At5g38780 [Source:UniProtKB/Swiss-Prot;Acc:Q9FKR0]	15529778	15531524	35.89
	AT5G05845			15533514	15533753	30.42
	AT5G38790		Putative uncharacterized protein [Source:UniProtKB/TrEMBL;Acc:Q9FKQ9]	15533713	15534371	34.6
	AT5G05855			15534665	15534898	36.75
	AT5G38800	BZIP43	Basic leucine zipper 43 [Source:UniProtKB/Swiss-Prot;Acc:Q9FMC2]	15538141	15538889	40.19
	AT5G38810		Putative F-box protein At5g38810 [Source:UniProtKB/Swiss-Prot;Acc:Q9FMC1]	15540484	15541540	39.36
	AT5G38820			15543115	15545448	38.77
	AT5G38830		Cysteine--tRNA ligase 2, cytoplasmic [Source:UniProtKB/Swiss-Prot;Acc:B3LFA4]	15545519	15548293	38.59
	AT5G38840		Kanadaptin-like protein [Source:UniProtKB/TrEMBL;Acc:Q0WW94]	15551243	15555077	38.72
	AT5G38850		Disease resistance protein (TIR-NBS-LRR class) [Source:UniProtKB/TrEMBL;Acc:Q9FMB7]	15555156	15558882	39.39
	AT5G38860	BIM3	Transcription factor BIM3 [Source:UniProtKB/Swiss-Prot;Acc:Q9FMB6]	15558901	15561126	35.76
	AT5G38865		Uncharacterized protein (Fragment) [Source:UniProtKB/TrEMBL;Acc:A0A1P8BB09]	15561891	15562982	40.2
	AT5G38880	05-Aug	AUGMIN subunit 5 [Source:UniProtKB/Swiss-Prot;Acc:Q9FMB4]	15562939	15569250	37.63
	AT5G38890		AT5G38890 protein [Source:UniProtKB/TrEMBL;Acc:A2RVT9]	15569418	15571169	37.27
	AT5G38895		RING/U-box superfamily protein [Source:TAIR;Acc:AT5G38895]	15571254	15573526	35.5
	AT5G38900		Thioredoxin superfamily protein [Source:TAIR;Acc:AT5G38900]	15573505	15575712	34.51
	AT5G05865			15574935	15575123	30.69
	AT5G38905		pre-tRNA [Source:TAIR;Acc:AT5G38905]	15575857	15575930	55.41
	AT5G38910		Putative germin-like protein subfamily 1 member 9 [Source:UniProtKB/Swiss-Prot;Acc:Q9FMB0]	15578749	15579733	40.81
	AT5G38920		Polynucleotidyl transferase, ribonuclease H-like superfamily protein [Source:UniProtKB/TrEMBL;Acc:F4KCZ1]	15581719	15582439	44.94
	AT5G38930		Germin-like protein subfamily 1 member 10 [Source:UniProtKB/Swiss-Prot;Acc:Q9FMA9]	15585028	15586029	41.32
	AT5G38940		RmlC-like cupins superfamily protein [Source:TAIR;Acc:AT5G38940]	15588623	15589791	39.52
	AT5G38950		RmlC-like cupins superfamily protein [Source:UniProtKB/TrEMBL;Acc:F4KCZ4]	15590693	15591050	42.46
	AT5G38960		Putative germin-like protein subfamily 1 member 12 [Source:UniProtKB/Swiss-Prot;Acc:Q9FMA6]	15592922	15593963	37.52
	AT5G38970	CYP85A1	Cytochrome P450 85A1 [Source:UniProtKB/Swiss-Prot;Acc:Q9FMA5]	15594674	15598089	33.84
	AT5G05875			15603871	15604218	42.82
	AT5G38980		At5g38980 [Source:UniProtKB/TrEMBL;Acc:Q8GXY9]	15606683	15607409	37.41
	AT5G38990		Probable receptor-like protein kinase At5g38990 [Source:UniProtKB/Swiss-Prot;Acc:Q9FID9]	15608685	15611664	44.19
	AT5G39000		Putative receptor-like protein kinase At5g39000 [Source:UniProtKB/Swiss-Prot;Acc:Q9FID8]	15611812	15614664	43.39
	AT5G39010		Putative uncharacterized protein [Source:UniProtKB/TrEMBL;Acc:Q9FID7]	15615916	15616457	39.67
	AT5G39020		Probable receptor-like protein kinase At5g39020 [Source:UniProtKB/Swiss-Prot;Acc:Q9FID6]	15616770	15619446	41.99

AT5G39024		unknown protein; Ha. [Source:TAIR;Acc:AT5G39024]	15619691	15619786	32.29
AT5G39030		Probable receptor-like protein kinase At5g39030 [Source:UniProtKB/Swiss-Prot;Acc:Q9FID5]	15619811	15622620	40.85
AT5G39040	ABCB27	ABC transporter B family member 27 [Source:UniProtKB/Swiss-Prot;Acc:Q0WML0]	15625460	15629835	36.29
AT5G39050	PMAT1	Phenolic glucoside malonyltransferase 1 [Source:UniProtKB/Swiss-Prot;Acc:Q940Z5]	15634444	15636480	41.68
AT5G39080		Acyltransferase-like protein [Source:UniProtKB/TrEMBL;Acc:Q9FID2]	15641442	15643216	41.18
AT5G39090		Acyltransferase-like protein [Source:UniProtKB/TrEMBL;Acc:Q9FID1]	15643493	15645172	42.32
AT5G39100	GLP6	germin-like protein 6 [Source:TAIR;Acc:AT5G39100]	15653125	15654028	40.15
AT5G39110		Germin-like protein subfamily 1 member 14 [Source:UniProtKB/Swiss-Prot;Acc:Q9FID0]	15657697	15658632	38.89
AT5G39120		Germin-like protein subfamily 1 member 15 [Source:UniProtKB/Swiss-Prot;Acc:Q9FIC9]	15662582	15663527	38.69
AT5G39130		Germin-like protein subfamily 1 member 16 [Source:UniProtKB/Swiss-Prot;Acc:Q9FIC8]	15665502	15666472	39.34
AT5G39140		unknown protein; BEST Arabidopsis thaliana protein match is: unknown protein (TAIR:AT5G39200.1); Ha. [Source:TAIR;Acc:AT5G39140]	15667173	15667761	40.41
AT5G39150		Germin-like protein subfamily 1 member 17 [Source:UniProtKB/Swiss-Prot;Acc:Q9FIC6]	15669898	15670904	39.13
AT5G39160	GLP2A	Germin-like protein subfamily 1 member 18 [Source:UniProtKB/Swiss-Prot;Acc:P92999]	15679005	15680141	37.47
AT5G39170		unknown protein; BEST Arabidopsis thaliana protein match is: unknown protein (TAIR:AT5G39200.1); Ha. [Source:TAIR;Acc:AT5G39170]	15680731	15681325	40.5
AT5G39180		Germin-like protein subfamily 1 member 19 [Source:UniProtKB/Swiss-Prot;Acc:Q9FL89]	15683464	15684471	38.99
AT5G39190	GLP5A	Germin-like protein subfamily 1 member 20 [Source:UniProtKB/Swiss-Prot;Acc:P92996]	15692542	15693735	37.19
AT5G39200		unknown protein; BEST Arabidopsis thaliana protein match is: unknown protein (TAIR:AT5G39140.1); Ha. [Source:TAIR;Acc:AT5G39200]	15694238	15695042	40.99
AT5G39210	CRR7	Protein CHLORORESPIRATORY REDUCTION 7, chloroplastic [Source:UniProtKB/Swiss-Prot;Acc:Q9FL87]	15703078	15704272	34.23
AT5G39220		Alpha/beta-Hydrolases superfamily protein [Source:UniProtKB/TrEMBL;Acc:Q84JL7]	15705706	15708274	35.03
AT5G39230		TFIIB zinc-binding protein [Source:UniProtKB/TrEMBL;Acc:Q3E7U7]	15709474	15709925	36.95
AT5G05885			15710444	15710740	31.31
AT5G05895			15710479	15711244	34.73
AT5G39240		unknown protein; BEST Arabidopsis thaliana protein match is: unknown protein (TAIR:AT3G29370.1); Ha. [Source:TAIR;Acc:AT5G39240]	15712549	15713158	37.21
AT5G39250		F-box protein At5g39250 [Source:UniProtKB/Swiss-Prot;Acc:Q9FL82]	15724477	15727206	36.12
AT5G39260	EXPA21	Expansin-A21 [Source:UniProtKB/Swiss-Prot;Acc:Q9FL81]	15726243	15727458	37.58
AT5G39270	EXPA22	Expansin-A22 [Source:UniProtKB/Swiss-Prot;Acc:Q9FL80]	15729037	15730231	37.57
AT5G39280	EXPA23	Expansin-A23 [Source:UniProtKB/Swiss-Prot;Acc:Q9FL79]	15730653	15731790	37.61
AT5G39290	EXPA26	Putative expansin-A26 [Source:UniProtKB/Swiss-Prot;Acc:Q9FL78]	15735794	15736979	37.94
AT5G39300	EXPA25	Expansin-A25 [Source:UniProtKB/Swiss-Prot;Acc:Q9FL77]	15737366	15738479	37.52
AT5G39310	EXPA24	Expansin-A24 [Source:UniProtKB/Swiss-Prot;Acc:Q9FL76]	15739032	15740605	39.26
AT5G39320	UGD4	UDP-glucose 6-dehydrogenase 4 [Source:UniProtKB/Swiss-Prot;Acc:Q9FM01]	15742543	15744946	40.18
AT5G39330		Gb [Source:UniProtKB/TrEMBL;Acc:Q9FM00]	15746209	15748355	33.58
AT5G39340	AHP3	histidine-containing phosphotransmitter 3 [Source:TAIR;Acc:AT5G39340]	15748731	15750891	36.51
AT5G39350	PCMP-E16	Pentatricopeptide repeat-containing protein At5g39350 [Source:UniProtKB/Swiss-Prot;Acc:Q9FLZ9]	15750875	15752962	42.43
AT5G39360	EDL2	EDL2 [Source:UniProtKB/TrEMBL;Acc:A0A178UC92]	15753006	15754690	37.15
AT5G39365		Putative defensin-like protein 40 [Source:UniProtKB/Swiss-Prot;Acc:Q2V325]	15756106	15756911	30.77
AT5G05905			15757451	15757709	34.75
AT5G39370		Curculin-like (Mannose-binding) lectin family protein [Source:UniProtKB/TrEMBL;Acc:Q9FLZ7]	15757717	15758109	43
AT5G05915			15758105	15758192	35.23
AT5G39380		Emb [Source:UniProtKB/TrEMBL;Acc:Q9FLZ6]	15758717	15761747	37.68
AT5G39390		Leucine-rich repeat protein kinase family protein [Source:UniProtKB/TrEMBL;Acc:F4KEF0]	15763715	15765469	39.54
AT5G39400	PTEN1	Phosphatidylinositol 3,4,5-trisphosphate 3-phosphatase and protein-tyrosine-phosphatase PTEN1 [Source:UniProtKB/Swiss-Prot;Acc:Q9FLZ5]	15766037	15768354	38.31
AT5G39410		Probable mitochondrial saccharopine dehydrogenase-like oxidoreductase At5g39410 [Source:UniProtKB/Swiss-Prot;Acc:Q8LGI2]	15767898	15770456	39.51
AT5G05925			15770687	15771129	33.41
AT5G39420	cdc2cAt	At5g39420 [Source:UniProtKB/TrEMBL;Acc:B5X564]	15771931	15775251	38
AT5G39430		DUF1336 family protein, putative (DUF1336) [Source:UniProtKB/TrEMBL;Acc:Q9ZR99]	15775302	15777599	36.34
AT5G39440	SnRK1.3	SNF1-related protein kinase catalytic subunit alpha KIN12 [Source:UniProtKB/Swiss-Prot;Acc:Q9FLZ3]	15781907	15784699	30.4
AT5G39450		F-box protein At5g39450 [Source:UniProtKB/Swiss-Prot;Acc:Q8RWD6]	15785717	15788150	38.91
AT5G39460		Putative F-box protein At5g39460 [Source:UniProtKB/Swiss-Prot;Acc:Q9FLZ1]	15788289	15790004	42.31
AT5G05935			15790573	15790987	38.07
AT5G05955			15790645	15790949	40
AT5G39470		Putative F-box protein At5g39470 [Source:UniProtKB/Swiss-Prot;Acc:Q3E8K6]	15791952	15793062	35.82
AT5G39471		Cysteine/Histidine-rich C1 domain family protein [Source:UniProtKB/TrEMBL;Acc:Q9FLZ0]	15793789	15795743	35.29
AT5G39480		Putative F-box protein At5g39480 [Source:UniProtKB/Swiss-Prot;Acc:Q9FLY8]	15801635	15803341	42.3
AT5G39490		Probable F-box protein At5g39490 [Source:UniProtKB/Swiss-Prot;Acc:Q9FLY7]	15805808	15807508	42.03
AT5G39493	SPH15	S-protein homolog 15 [Source:UniProtKB/Swiss-Prot;Acc:Q9FLY6]	15809955	15810693	31.12
AT5G39500	GNL1	ARF guanine-nucleotide exchange factor GNL1 [Source:UniProtKB/Swiss-Prot;Acc:Q9FLY5]	15814559	15820151	41.1
AT5G39510	VTI11	Vesicle transport v-SNARE 11 [Source:UniProtKB/Swiss-Prot;Acc:Q9SEL6]	15821293	15823840	37.76
AT5G05975			15822219	15822437	35.62
AT5G39520		Uncharacterized protein At5g39520 [Source:UniProtKB/TrEMBL;Acc:Q0WW43]	15823735	15825566	35.59
AT5G39530		Uncharacterized protein At5g39530/MUL8_210 [Source:UniProtKB/TrEMBL;Acc:Q8GWM5]	15826017	15828035	36.7
AT5G39535		pre-tRNA [Source:TAIR;Acc:AT5G39535]	15827026	15827097	54.17
AT5G39540		F-box associated ubiquitination effector family protein [Source:UniProtKB/TrEMBL;Acc:F4KEG7]	15833743	15834420	39.97
AT5G39550	ORTH1	E3 ubiquitin-protein ligase ORTHRUS 1 [Source:UniProtKB/Swiss-Prot;Acc:Q9FKA7]	15837178	15840678	39.67
AT5G39560		F-box/kelch-repeat protein At5g39560 [Source:UniProtKB/Swiss-Prot;Acc:Q6NL02]	15841692	15843169	42.83
AT5G39570		Transmembrane protein [Source:UniProtKB/TrEMBL;Acc:Q8L7C5]	15843881	15846467	40.66
AT5G39580	PER62	Peroxidase 62 [Source:UniProtKB/Swiss-Prot;Acc:Q9FKA4]	15847065	15849141	36.4
AT5G39590		TLD-domain containing nucleolar protein [Source:UniProtKB/TrEMBL;Acc:Q9FKA3]	15851389	15853990	38.62
AT5G39600		39S ribosomal protein [Source:UniProtKB/TrEMBL;Acc:Q940G3]	15853929	15855192	38.84
AT5G39610	NAC92	NAC domain-containing protein 92 [Source:UniProtKB/Swiss-Prot;Acc:Q9FKA0]	15858398	15859911	36.99
AT5G39620	RABG1	Ras-related protein RABG1 [Source:UniProtKB/Swiss-Prot;Acc:Q948K6]	15864166	15865782	35.56
AT5G39630		Vesicle transport v-SNARE family protein [Source:UniProtKB/TrEMBL;Acc:Q1PDQ2]	15868262	15869714	32.21
AT5G39635	MIR169C	MIR169C; miRNA [Source:TAIR;Acc:AT5G39635]	15870590	15871000	33.82
AT5G39640		Putative endonuclease or glycosyl hydrolase [Source:UniProtKB/TrEMBL;Acc:F4KEH9]	15873039	15873269	46.32
AT5G39645		Defensin-like (DEFL) family protein [Source:UniProtKB/TrEMBL;Acc:A8MR15]	15873392	15873680	32.18
AT5G39650	DMP9	Protein DMP9 [Source:UniProtKB/Swiss-Prot;Acc:Q9FK96]	15875125	15876116	42.04
AT5G39660	CDF2	Cyclic dof factor 2 [Source:UniProtKB/Swiss-Prot;Acc:Q93ZL5]	15878698	15881537	39.4
AT5G39670	CML46	Probable calcium-binding protein CML46 [Source:UniProtKB/Swiss-Prot;Acc:Q93Z27]	15883179	15884067	37.23
AT5G05995			15883195	15884130	36.75
AT5G39680	EMB2744	Pentatricopeptide repeat-containing protein At5g39680 [Source:UniProtKB/Swiss-Prot;Acc:Q9FK93]	15884001	15886449	39.65
AT5G39690	ANAC093	NAC domain containing protein 93 [Source:UniProtKB/TrEMBL;Acc:F4KEI6]	15888171	15890187	35.4
AT5G06005			15890980	15891187	36.06
AT5G39693	MIR869A	MIR869a; miRNA [Source:TAIR;Acc:AT5G39693]	15891579	15891873	40
AT5G39700	MYB89	Myb domain protein 89 [Source:UniProtKB/TrEMBL;Acc:Q9SPG4]	15893761	15894424	36.14
AT5G39710	EMB2745	Pentatricopeptide repeat-containing protein At5g39710 [Source:UniProtKB/Swiss-Prot;Acc:Q9FIX3]	15895682	15898219	39.36
AT5G39715		Lysine histidine transporter-like protein [Source:UniProtKB/TrEMBL;Acc:A0A1P8BGM4]	15898233	15899136	34.73
AT5G39720	AIG2LA	At5g39720 [Source:UniProtKB/TrEMBL;Acc:A2RVP6]	15899618	15900667	36
AT5G39730	AIG2LB	AIG2-like protein B [Source:UniProtKB/Swiss-Prot;Acc:Q9FIX1]	15901569	15902958	35.54
AT5G39740	RPL5B	RPL5B [Source:UniProtKB/TrEMBL;Acc:A0A178UJU5]	15903279	15905532	38.15
AT5G39750	AGL81	Agamous-like MADS-box protein AGL81 [Source:UniProtKB/Swiss-Prot;Acc:Q9FIX0]	15906875	15907942	41.67
AT5G39760	ZHD10	Zinc-finger homeodomain protein 10 [Source:UniProtKB/Swiss-Prot;Acc:Q9FIW9]	15911350	15912860	42.55
AT5G39770		Restriction endonuclease, type II-like superfamily protein [Source:UniProtKB/TrEMBL;Acc:F4KFU8]	15919241	15927549	43.56
AT5G39775		Gag-Pol polyprotein/retrotransposon [Source:UniProtKB/TrEMBL;Acc:A0A1P8BH06]	15923803	15924404	45.18
AT5G39785		Protein of unknown function (DUF1666) [Source:TAIR;Acc:AT5G39785]	15928765	15932347	34.08
AT5G39790		5'-AMP-activated protein kinase-related [Source:TAIR;Acc:AT5G39790]	15932505	15935270	34.27
AT5G39800		Mitochondrial ribosomal protein L27 [Source:UniProtKB/TrEMBL;Acc:Q8GXS6]	15935453	15937201	33.5
AT5G39810	AGL98	AGAMOUS-like 98 [Source:UniProtKB/TrEMBL;Acc:Q9FIW6]	15937278	15938458	40.56
AT5G39820	ANAC094	Putative NAC domain-containing protein 94 [Source:UniProtKB/Swiss-Prot;Acc:Q9FIW5]	15939110	15940590	40.04
AT5G39830	DEGP8	Protease Do-like 8, chloroplastic [Source:UniProtKB/Swiss-Prot;Acc:Q9LU10]	15942764	15945897	36.06
AT5G39840		DExH-box ATP-dependent RNA helicase DExH18, mitochondrial [Source:UniProtKB/Swiss-Prot;Acc:F4KFV7]	15946566	15949422	39.94
AT5G39850	RPS9C	40S ribosomal protein S9-2 [Source:UniProtKB/Swiss-Prot;Acc:Q9FLF0]	15949910	15951475	36.97
AT5G39860	PRE1	Transcription factor PRE1 [Source:UniProtKB/Swiss-Prot;Acc:Q9FLE9]	15957368	15958559	34.48
AT5G39865		Uncharacterized protein At5g39865 [Source:UniProtKB/Swiss-Prot;Acc:Q9FLE8]	15965301	15967186	38.81
AT5G39870		Protein of unknown function (DUF1216) [Source:TAIR;Acc:AT5G39870]	15968589	15970046	32.37
AT5G39880		Transmembrane protein [Source:UniProtKB/TrEMBL;Acc:Q9FLE6]	15970887	15972344	39.3

AT5G39890	PCO2	Plant cysteine oxidase 2 [Source:UniProtKB/Swiss-Prot;Acc:Q8LGJ5]	15974270	15976270	37.18
AT5G39895		pre-tRNA [Source:TAIR;Acc:AT5G39895]	15976287	15976359	58.9
AT5G39900		Translation factor GUF1 homolog, mitochondrial [Source:UniProtKB/Swiss-Prot;Acc:Q9FLE4]	15976507	15979001	39.8
AT5G39910		Pectin lyase-like superfamily protein [Source:UniProtKB/TrEMBL;Acc:F4KFW5]	15979412	15981671	31.9
AT5G39920		CONTAINS InterPro DOMAIN/s: Pre-mRNA cleavage complex II Clp1 (InterPro:IPR010655); BEST Arabidopsis thaliana protein match is: CLP	15982173	15983136	34.44
AT5G39930	CLPS5	Protein CLP1 homolog 5 [Source:UniProtKB/Swiss-Prot;Acc:Q9FLE2]	15985025	15987339	35.16
AT5G39940		At5g39940 [Source:UniProtKB/TrEMBL;Acc:Q8GUI2]	15987461	15990701	36.22
AT5G39950	TRX2	Thioredoxin H2 [Source:UniProtKB/Swiss-Prot;Acc:Q38879]	15990687	15991978	37.46
AT5G39960		GTP-binding protein [Source:UniProtKB/TrEMBL;Acc:F4KFX1]	15993953	15997058	39.09
AT5G39970		catalytics [Source:TAIR;Acc:AT5G39970]	15997970	16000813	39.45
AT5G39980		Pentatricopeptide repeat-containing protein At5g39980, chloroplastic [Source:UniProtKB/Swiss-Prot;Acc:Q9FLD8]	16000712	16003072	40.58
AT5G39990	GLCAT14A	Beta-glucuronosyltransferase GlcAT14A [Source:UniProtKB/Swiss-Prot;Acc:Q9FLD7]	16004253	16006823	38.35
AT5G40000		AAA-ATPase At5g40000 [Source:UniProtKB/Swiss-Prot;Acc:F4KFX5]	16010869	16012559	38.5
AT5G40010	AATP1	AAA-ATPase ASD, mitochondrial [Source:UniProtKB/Swiss-Prot;Acc:Q9FLD5]	16020058	16021916	38.25
AT5G40020		At5g40020 [Source:UniProtKB/TrEMBL;Acc:Q9FLD4]	16022579	16024316	34.7
AT5G40030		Protein kinase superfamily protein [Source:UniProtKB/TrEMBL;Acc:Q9LUK3]	16026047	16028483	37.67
AT5G40040	RPP2E	60S acidic ribosomal protein P2-5 [Source:UniProtKB/Swiss-Prot;Acc:Q9LUK2]	16029703	16030335	41.71
AT5G40050		Putative F-box protein At5g40050 [Source:UniProtKB/Swiss-Prot;Acc:Q9LUK1]	16032083	16033551	39.14
AT5G40060		Disease resistance protein (NBS-LRR class) family [Source:UniProtKB/TrEMBL;Acc:F4KFY1]	16034441	16039096	38.42
AT5G40070		MADS-box family protein [Source:UniProtKB/TrEMBL;Acc:F4KFY2]	16039179	16039747	39.89
AT5G40080		At5g40080 [Source:UniProtKB/TrEMBL;Acc:Q9LUJ9]	16039927	16041714	34.23
AT5G40090	CHL1	CHL1 [Source:UniProtKB/TrEMBL;Acc:A0A178UIM8]	16041919	16043733	39.28
AT5G40100		Disease resistance protein (TIR-NBS-LRR class) family [Source:UniProtKB/TrEMBL;Acc:F4KFY5]	16043727	16047572	41.55
AT5G40120	AGL76	AGAMOUS-like 76 [Source:UniProtKB/TrEMBL;Acc:Q9FL19]	16051879	16053036	39.72
AT5G40140	PUB40	U-box domain-containing protein 40 [Source:UniProtKB/Swiss-Prot;Acc:Q9FL17]	16057079	16059303	40.99
AT5G40150	PER63	Peroxidase 63 [Source:UniProtKB/Swiss-Prot;Acc:Q9FL16]	16059504	16061043	44.22
AT5G40153		Peroxidase [Source:UniProtKB/TrEMBL;Acc:Q0WR27]	16060342	16060629	52.78
AT5G40155		Defensin-like protein 313 [Source:UniProtKB/Swiss-Prot;Acc:Q3E8K0]	16061891	16062548	33.28
AT5G40160	EMB506	Ankyrin repeat domain-containing protein EMB506, chloroplastic [Source:UniProtKB/Swiss-Prot;Acc:Q9SQK3]	16062479	16064689	34.1
AT5G40170	AtRLP54	Receptor-like protein 54 [Source:UniProtKB/Swiss-Prot;Acc:F4KHA2]	16064859	16067628	39.64
AT5G40180		BEST Arabidopsis thaliana protein match is: TRICHOME BIREFRINGENCE-LIKE 22 (TAIR:AT3G28150.1); Ha. [Source:TAIR;Acc:AT5G401	16067868	16068751	37.78
AT5G40190		At5g40190 [Source:UniProtKB/TrEMBL;Acc:Q9FL13]	16069043	16070044	40.32
AT5G40200	DEGP9	Protease Do-like 9 [Source:UniProtKB/Swiss-Prot;Acc:Q9FL12]	16070146	16073387	37.88
AT5G40210		WAT1-related protein At5g40210 [Source:UniProtKB/Swiss-Prot;Acc:Q945L4]	16073534	16076221	32.89
AT5G40220	AGL43	AGAMOUS-like 43 [Source:UniProtKB/TrEMBL;Acc:Q9FL10]	16078390	16079364	41.95
AT5G40230		WAT1-related protein At5g40230 [Source:UniProtKB/Swiss-Prot;Acc:F4KHA8]	16079648	16081893	38.29
AT5G40240		WAT1-related protein [Source:UniProtKB/TrEMBL;Acc:F4KHA9]	16081988	16085213	38.44
AT5G40250	ATL46	RING-H2 finger protein ATL46 [Source:UniProtKB/Swiss-Prot;Acc:Q9FL07]	16085635	16087543	38.82
AT5G40260	SWEET8	Bidirectional sugar transporter SWEET8 [Source:UniProtKB/Swiss-Prot;Acc:Q8LFH5]	16089678	16091797	33.96
AT5G40270		AT5g40270/MSN9_170 [Source:UniProtKB/TrEMBL;Acc:Q9FL05]	16093035	16097245	36.59
AT5G40275		other RNA [Source:TAIR;Acc:AT5G40275]	16098190	16100051	36.09
AT5G40280	ERA1	Prenyltransferase family protein [Source:TAIR;Acc:AT5G40280]	16101343	16104962	38.26
AT5G40290		HD domain-containing metal-dependent phosphohydrolase family protein [Source:UniProtKB/TrEMBL;Acc:F4KHB6]	16105259	16108501	34.29
AT5G40300		CASP-like protein 4A1 [Source:UniProtKB/Swiss-Prot;Acc:Q9FNE8]	16110634	16113059	35.66
AT5G40310		Exonuclease family protein [Source:TAIR;Acc:AT5G40310]	16113079	16116171	32.69
AT5G40315			16119247	16119258	25
AT5G40316		other RNA [Source:TAIR;Acc:AT5G40316]	16119296	16120018	42.46
AT5G40320		CHP-rich zinc finger protein-like [Source:UniProtKB/TrEMBL;Acc:Q9FNE5]	16121708	16123556	41.1
AT5G40330	MYB23	Transcription factor MYB23 [Source:UniProtKB/Swiss-Prot;Acc:Q96276]	16127725	16129661	33.04
AT5G40340		Tudor/PWWP/MBT superfamily protein [Source:UniProtKB/TrEMBL;Acc:Q9FNE4]	16130881	16135393	38.82
AT5G40348		other RNA [Source:TAIR;Acc:AT5G40348]	16136594	16141245	31.32
AT5G40350	MYB24	Transcription factor MYB24 [Source:UniProtKB/Swiss-Prot;Acc:Q9SPG9]	16138544	16141134	32.42
AT5G06015			16141472	16141678	36.23
AT5G06025			16143399	16143688	38.97
AT5G40360	MYB115	Transcription factor MYB115 [Source:UniProtKB/Swiss-Prot;Acc:Q1PDP9]	16144792	16146671	32.23
AT5G40370		Glutaredoxin family protein [Source:UniProtKB/TrEMBL;Acc:B3H604]	16147347	16149256	35.92
AT5G40380	CRK42	Cysteine-rich receptor-like protein kinase 42 [Source:UniProtKB/Swiss-Prot;Acc:Q9FNE1]	16151522	16155215	36.9
AT5G40382		Putative cytochrome c oxidase subunit 5C-4 [Source:UniProtKB/Swiss-Prot;Acc:Q9FNE0]	16155086	16156221	32.13
AT5G40384	MIR866A	MIR866a; miRNA [Source:TAIR;Acc:AT5G40384]	16157823	16157948	33.33
AT5G06035			16158958	16159355	36.68
AT5G40390	RFS5	Probable galactinol--sucrose galactosyltransferase 5 [Source:UniProtKB/Swiss-Prot;Acc:Q9FND9]	16161449	16165453	40.57
AT5G40395	U6acat	U6acat; snRNA [Source:TAIR;Acc:AT5G40395]	16166180	16166387	41.83
AT5G40400		Pentatricopeptide repeat (PPR) superfamily protein [Source:TAIR;Acc:AT5G40400]	16166231	16168870	39.28
AT5G40405	PCMP-H14	Putative pentatricopeptide repeat-containing protein At5g40405 [Source:UniProtKB/Swiss-Prot;Acc:Q9FND7]	16168949	16171317	41.62
AT5G40410	PCMP-H15	Pentatricopeptide repeat-containing protein At5g40410, mitochondrial [Source:UniProtKB/Swiss-Prot;Acc:Q9FND6]	16171374	16173354	39.02
AT5G40420	OLEO2	Oleosin 21.2 kDa [Source:UniProtKB/Swiss-Prot;Acc:Q39165]	16173340	16174783	37.19
AT5G40430	MYB22	At5g40430 [Source:UniProtKB/TrEMBL;Acc:Q9SPH0]	16176427	16177731	33.87
AT5G06045			16179598	16179902	46.56
AT5G40440	MKK3	Mitogen-activated protein kinase kinase 3 [Source:UniProtKB/Swiss-Prot;Acc:O80396]	16181628	16184827	34.81
AT5G06055			16183414	16183534	33.88
AT5G40450		unknown protein; FUNCTIONS IN: molecular_function unknown; INVOLVED IN: biological_process unknown; LOCATED IN: chloroplast, pla	16184650	16195888	38.05
AT5G06065			16201725	16202096	39.52
AT5G40460	SMR6	Cyclin-dependent protein kinase inhibitor SMR6 [Source:UniProtKB/Swiss-Prot;Acc:Q29Q81]	16202142	16203358	34.35
AT5G06075			16206042	16206453	32.04
AT5G40470		AT5g40470/K21I16_20 [Source:UniProtKB/TrEMBL;Acc:Q9FI63]	16208009	16209927	37.94
AT5G40480	GB210	Nuclear pore complex protein GP210 [Source:UniProtKB/Swiss-Prot;Acc:F4KHD8]	16213036	16224177	38.19
AT5G40490		RNA-binding (RRM/RBD/RNP motifs) family protein [Source:UniProtKB/TrEMBL;Acc:Q9FM47]	16225326	16227757	43.01
AT5G40500		Uncharacterized protein At5g40500 [Source:UniProtKB/TrEMBL;Acc:Q8VZ30]	16227771	16228693	37.7
AT5G40510		Sucrase/ferredoxin-like family protein [Source:TAIR;Acc:AT5G40510]	16228960	16231142	37.52
AT5G40520		unknown protein; Ha. [Source:TAIR;Acc:AT5G40520]	16231143	16234348	38.62
AT5G40530		S-adenosyl-L-methionine-dependent methyltransferases superfamily protein [Source:TAIR;Acc:AT5G40530]	16234461	16236311	35.82
AT5G40540		Protein kinase ATN1 [Source:UniProtKB/TrEMBL;Acc:Q9FM43]	16236956	16239601	35.26
AT5G40545		pre-tRNA [Source:TAIR;Acc:AT5G40545]	16239638	16239709	61.11
AT5G40550		SGF29 tudor-like domain [Source:TAIR;Acc:AT5G40550]	16241009	16243410	35.26
AT5G40560	DEGP13	Putative protease Do-like 13 [Source:UniProtKB/Swiss-Prot;Acc:Q9FM41]	16243537	16246519	33.83
AT5G40570		Surfeit locus protein 2 (SURF2) [Source:UniProtKB/TrEMBL;Acc:A8MRG2]	16246760	16248390	36.66
AT5G40580	PBB2	Proteasome subunit beta type-7-B [Source:UniProtKB/Swiss-Prot;Acc:Q7DLS1]	16248286	16250925	37.73
AT5G40590		At5g40590 [Source:UniProtKB/TrEMBL;Acc:Q9FM39]	16251318	16252434	37.87
AT5G40600		unknown protein; FUNCTIONS IN: molecular_function unknown; LOCATED IN: endomembrane system; EXPRESSED IN: 22 plant structures;	16253469	16256829	37.82
AT5G40595		unknown protein; Ha. [Source:TAIR;Acc:AT5G40595]	16253510	16254079	44.04
AT5G40610	DHAPRD	Glycerol-3-phosphate dehydrogenase [NAD(+)] 1, chloroplastic [Source:UniProtKB/Swiss-Prot;Acc:Q9SCX9]	16264829	16267406	38.87
AT5G40620		Dbj [Source:UniProtKB/TrEMBL;Acc:Q9FM36]	16269157	16269833	37.52
AT5G40630		AT5G40630 protein [Source:UniProtKB/TrEMBL;Acc:Q9FM35]	16271047	16272513	30.67
AT5G40640		Transmembrane protein [Source:UniProtKB/TrEMBL;Acc:Q84K38]	16277042	16280424	37.63
AT5G40645		At5g40645 [Source:UniProtKB/TrEMBL;Acc:Q8LGF0]	16280283	16280993	34.04
AT5G40650	SDH2-2	Succinate dehydrogenase [ubiquinone] iron-sulfur subunit 2, mitochondrial [Source:UniProtKB/Swiss-Prot;Acc:Q8LB02]	16281394	16283527	38.57
AT5G40660		AT5g40660/MNF13_180 [Source:UniProtKB/TrEMBL;Acc:Q94BY8]	16283146	16285344	39.43
AT5G40670		Cystinosin homolog [Source:UniProtKB/Swiss-Prot;Acc:P57758]	16285711	16287947	36.16
AT5G40680		Galactose oxidase/kelch repeat superfamily protein [Source:UniProtKB/TrEMBL;Acc:Q9FM30]	16288754	16290001	42.87
AT5G40690		At5g40690 [Source:UniProtKB/TrEMBL;Acc:Q9FM29]	16290386	16291713	36.75
AT5G40700		At5g40700 [Source:UniProtKB/TrEMBL;Acc:Q5PP69]	16291755	16293649	36.46
AT5G40710		At5g40710 [Source:UniProtKB/TrEMBL;Acc:Q9FM27]	16294967	16297058	35.13
AT5G40720		C3H4 type zinc finger protein (DUF23) [Source:UniProtKB/TrEMBL;Acc:Q9FM26]	16297300	16300037	40.8
AT5G40730	AGP24	Arabinogalactan peptide 24 [Source:UniProtKB/Swiss-Prot;Acc:Q5PP12]	16301072	16301748	37.08
AT5G40740	06-Aug	AUG6 [Source:UniProtKB/TrEMBL;Acc:A0A178UQ56]	16301846	16306479	39.51

AT5G40750		FBD / Leucine Rich Repeat domains containing protein [Source:UniProtKB/TrEMBL;Acc:F4KIR0]	16308356	16309819	37.36
AT5G40760	G6PD6	G6PD6 [Source:UniProtKB/TrEMBL;Acc:A0A384LPG4]	16310663	16314811	37.12
AT5G40770	PHB3	Prohibitin-3, mitochondrial [Source:UniProtKB/Swiss-Prot;Acc:O04331]	16314850	16317291	39.31
AT5G40780	LHT1	Lysine histidine transporter 1 [Source:UniProtKB/Swiss-Prot;Acc:Q9FKS8]	16323567	16327382	34.85
AT5G40790		Emb [Source:UniProtKB/TrEMBL;Acc:Q9FKS7]	16330041	16331276	44.42
AT5G40800		Emb [Source:UniProtKB/TrEMBL;Acc:Q9FKS6]	16334581	16336017	41.54
AT5G40810	CYC12	Cytochrome c1 2, heme protein, mitochondrial [Source:UniProtKB/Swiss-Prot;Acc:Q9FKS5]	16339863	16342927	38.43
AT5G40820	ATRAD3	Ataxia telangiectasia-mutated and RAD3-related [Source:TAIR;Acc:AT5G40820]	16342728	16353898	40.94
AT5G40830		At5g40830 [Source:UniProtKB/TrEMBL;Acc:Q9FKS3]	16354362	16356341	38.13
AT5G40840	SYN2	Sister chromatid cohesion 1 protein 2 [Source:UniProtKB/Swiss-Prot;Acc:Q9FQ20]	16359331	16363878	38.57
AT5G40850	UPM1	AT5g40850/MHK7_8 [Source:UniProtKB/TrEMBL;Acc:Q42606]	16365900	16369316	40.24
AT5G40855		unknown protein; Ha. [Source:TAIR;Acc:AT5G40855]	16369114	16369350	40.93
AT5G40860		Transmembrane protein [Source:UniProtKB/TrEMBL;Acc:Q1PDP3]	16370368	16372294	33.47
AT5G40870	UKL1	Uridine kinase [Source:UniProtKB/TrEMBL;Acc:A0A178UHR6]	16374799	16378652	38.19
AT5G40880	ZFWD3	Uncharacterized protein At5g40880 (Fragment) [Source:UniProtKB/TrEMBL;Acc:C0SVR8]	16378893	16381352	40.53
AT5G40890	CLC-A	Chloride channel protein CLC-a [Source:UniProtKB/Swiss-Prot;Acc:P92941]	16381314	16385424	37.29
AT5G06085			16388635	16388841	39.13
AT5G40900		Glycosyltransferase [Source:UniProtKB/TrEMBL;Acc:F4KIT4]	16391836	16393427	34.23
AT5G06095			16394060	16394258	41.21
AT5G40910		Disease resistance protein (TIR-NBS-LRR class) family [Source:UniProtKB/TrEMBL;Acc:Q9FKR7]	16394462	16400489	37.86
AT5G40930	TOM20-4	Mitochondrial import receptor subunit TOM20-4 [Source:UniProtKB/Swiss-Prot;Acc:P82805]	16404457	16406079	36.6
AT5G40940	FLA20	Putative fasciclin-like arabinogalactan protein 20 [Source:UniProtKB/Swiss-Prot;Acc:Q9FGW0]	16406631	16407930	41.69
AT5G06105			16409804	16409862	37.29
AT5G40945		pre-tRNA [Source:TAIR;Acc:AT5G40945]	16410585	16410665	51.85
AT5G40950	RPL27	50S ribosomal protein L27, chloroplastic [Source:UniProtKB/Swiss-Prot;Acc:Q9FLN4]	16410782	16412114	39.76
AT5G40960		At5g40960 [Source:UniProtKB/TrEMBL;Acc:Q9FLN3]	16412404	16413202	36.05
AT5G40970		Protein of unknown function (DUF 3339) [Source:TAIR;Acc:AT5G40970]	16413671	16415897	34.93
AT5G40980		AT.I.24-6 protein, putative (DUF 3339) [Source:UniProtKB/TrEMBL;Acc:Q9FLN1]	16414510	16415265	36.64
AT5G40981		unknown protein; Ha. [Source:TAIR;Acc:AT5G40981]	16415791	16416044	38.58
AT5G40990	GLIP1	GDSL esterase/lipase 1 [Source:UniProtKB/Swiss-Prot;Acc:Q9FLN0]	16418842	16420616	37.24
AT5G41000	YSL4	Probable metal-nicotianamine transporter YSL4 [Source:UniProtKB/Swiss-Prot;Acc:Q6R3K8]	16420755	16423830	39.24
AT5G41010	NRPB12	DNA-directed RNA polymerases II, IV and V subunit 12 [Source:UniProtKB/Swiss-Prot;Acc:Q9FLM8]	16423919	16425324	36.2
AT5G06115			16424246	16424389	38.19
AT5G41020		Gb [Source:UniProtKB/TrEMBL;Acc:Q9FLM7]	16425883	16427797	40.94
AT5G41025		Transmembrane protein [Source:UniProtKB/TrEMBL;Acc:A0A1P8BCS2]	16426679	16427510	39.06
AT5G41030	TCP6	Transcription factor TCP6 [Source:UniProtKB/Swiss-Prot;Acc:Q9FLM6]	16428706	16429679	39.53
AT5G41040	HHT1	Omega-hydroxypalmitate O-feruloyl transferase [Source:UniProtKB/Swiss-Prot;Acc:Q94CD1]	16431304	16433305	39.91
AT5G41050		At5g41050 [Source:UniProtKB/TrEMBL;Acc:Q9FLM4]	16433243	16434458	35.28
AT5G41060	PAT06	Probable protein S-acyltransferase 6 [Source:UniProtKB/Swiss-Prot;Acc:Q9FLM3]	16435153	16437805	37.17
AT5G41070	DRB5	Double-stranded RNA-binding protein 5 [Source:UniProtKB/Swiss-Prot;Acc:Q8GY79]	16438265	16440055	38.36
AT5G41071		unknown protein; Ha. [Source:TAIR;Acc:AT5G41071]	16440069	16440251	39.34
AT5G41080	GDPD2	Glycerophosphodiester phosphodiesterase GDPD2 [Source:UniProtKB/Swiss-Prot;Acc:Q9FLM1]	16441655	16444023	37.44
AT5G41090	anac095	At5g41090 [Source:UniProtKB/TrEMBL;Acc:Q9FLM0]	16445655	16446673	38.57
AT5G41100		At5g41100 [Source:UniProtKB/TrEMBL;Acc:A4FVS2]	16447185	16450909	39.44
AT5G41110		Meiosis chromosome segregation family protein [Source:UniProtKB/TrEMBL;Acc:Q9FLL8]	16451470	16455364	37.82
AT5G41109		unknown protein; LOCATED IN: endomembrane system; Ha. [Source:TAIR;Acc:AT5G41109]	16452453	16452593	39.01
AT5G41120		Esterase/lipase/thioesterase family protein [Source:UniProtKB/TrEMBL;Acc:Q9FLL7]	16455094	16459253	38
AT5G41130		Esterase/lipase/thioesterase family protein [Source:TAIR;Acc:AT5G41130]	16459474	16463432	39.15
AT5G41140		Myosin heavy chain-related protein [Source:UniProtKB/TrEMBL;Acc:Q9FLL5]	16468327	16472810	39.03
AT5G41150	UVH1	Ultraviolet hypersensitive 1 [Source:UniProtKB/TrEMBL;Acc:Q2F6I1]	16472764	16477417	39.47
AT5G41160	ATPUP12	purine permease 12 [Source:TAIR;Acc:AT5G41160]	16477119	16478436	39.45
AT5G41170		Pentatricopeptide repeat-containing protein At5g41170, mitochondrial [Source:UniProtKB/Swiss-Prot;Acc:Q9FLL3]	16478486	16480584	38.02
AT5G41180		Leucine-rich repeat receptor-like protein kinase [Source:UniProtKB/TrEMBL;Acc:Q9FLL2]	16483760	16487427	37.68
AT5G41190		RNA-binding NOB1-like protein [Source:UniProtKB/TrEMBL;Acc:Q9FLL1]	16487466	16490101	41.2
AT5G41200	AGL75	Agamous-like MADS-box protein AGL75 [Source:UniProtKB/Swiss-Prot;Acc:Q9FLL0]	16490544	16491536	40.58
AT5G41210	GSTT1	Glutathione S-transferase T1 [Source:UniProtKB/Swiss-Prot;Acc:Q9ZRT5]	16492275	16494059	37.48
AT5G41220	GSTT3	Glutathione S-transferase T3 [Source:UniProtKB/Swiss-Prot;Acc:Q9FHE1]	16494325	16497163	38.39
AT5G41240	GSTT2	Glutathione S-transferase T2 [Source:UniProtKB/Swiss-Prot;Acc:Q8L727]	16497959	16500865	39.11
AT5G41250	GT20	Probable xyloglucan galactosyltransferase GT20 [Source:UniProtKB/Swiss-Prot;Acc:Q9FHD8]	16501217	16502902	40.81
AT5G41260	BSK8	Serine/threonine-protein kinase BSK8 [Source:UniProtKB/Swiss-Prot;Acc:Q9FHD7]	16503562	16507475	37.05
AT5G41265		pre-tRNA [Source:TAIR;Acc:AT5G41265]	16507533	16507604	59.72
AT5G41270		At5g41270 [Source:UniProtKB/TrEMBL;Acc:Q84VV1]	16507662	16509034	41.44
AT5G41280	CRRSP57	Cysteine-rich repeat secretory protein 57 [Source:UniProtKB/Swiss-Prot;Acc:Q9FHD5]	16509507	16511078	33.65
AT5G41290	CRRSP58	Cysteine-rich repeat secretory protein 58 [Source:UniProtKB/Swiss-Prot;Acc:Q9FHD4]	16512154	16513671	34.65
AT5G41300	CRRSP59	Cysteine-rich repeat secretory protein 59 [Source:UniProtKB/Swiss-Prot;Acc:Q9FHD3]	16514929	16516331	36.42
AT5G41310		P-loop nucleoside triphosphate hydrolases superfamily protein with CH (Calponin Homology) domain [Source:TAIR;Acc:AT5G41310]	16516470	16522392	32.96
AT5G06145			16528588	16529125	30.67
AT5G41315	GL3	MYC6.2 [Source:UniProtKB/TrEMBL;Acc:A0A178UFB0]	16529090	16533287	36.78
AT5G41320		Stress response NST1-like protein [Source:UniProtKB/TrEMBL;Acc:Q9FN68]	16533931	16535989	40.41
AT5G06155			16534231	16535281	41.67
AT5G41330		BTB/POZ domain-containing protein At5g41330 [Source:UniProtKB/Swiss-Prot;Acc:Q9FN67]	16535339	16538016	42.27
AT5G41340	UBC4	Ubiquitin-conjugating enzyme E2 4 [Source:UniProtKB/Swiss-Prot;Acc:P42748]	16538063	16540189	36.06
AT5G41350		RING/U-box superfamily protein [Source:TAIR;Acc:AT5G41350]	16541462	16543985	35.54
AT5G41360	XPB2	XPB2 [Source:UniProtKB/TrEMBL;Acc:A0A178UFA0]	16544333	16549327	37.26
AT5G41370	XPB1	General transcription and DNA repair factor IIH helicase subunit XPB1 [Source:UniProtKB/Swiss-Prot;Acc:Q38861]	16551119	16556038	37.97
AT5G41380		CCT motif family protein [Source:UniProtKB/TrEMBL;Acc:Q5Q0A4]	16561278	16563732	33.08
AT5G41390		PLAC8 family protein [Source:UniProtKB/TrEMBL;Acc:Q45GG6]	16565303	16567483	36.5
AT5G41400		Putative RING zinc finger protein [Source:UniProtKB/TrEMBL;Acc:Q9FN60]	16569375	16570275	40.73
AT5G41401		unknown protein; Ha. [Source:TAIR;Acc:AT5G41401]	16572025	16572189	38.79
AT5G41410	BEL1	Homeobox protein BEL1 homolog [Source:UniProtKB/Swiss-Prot;Acc:Q38897]	16579936	16584077	34.45
AT5G41420		unknown protein; Ha. [Source:TAIR;Acc:AT5G41420]	16585119	16585363	42.04
AT5G41430		RING/U-box superfamily protein [Source:UniProtKB/TrEMBL;Acc:Q9FN58]	16586119	16586604	41.56
AT5G41440		Emb [Source:UniProtKB/TrEMBL;Acc:Q9FN57]	16587235	16587609	42.67
AT5G41450		RING/U-box superfamily protein [Source:UniProtKB/TrEMBL;Acc:Q9FN56]	16588600	16589094	36.97
AT5G41460		At5g41460 [Source:UniProtKB/TrEMBL;Acc:Q9FN55]	16589390	16592381	38.74
AT5G41470		Nuclear transport factor 2 (NTF2) family protein [Source:UniProtKB/TrEMBL;Acc:F4JYE8]	16593841	16595568	31.02
AT5G41480	DHFS	Dihydrofolate synthetase [Source:UniProtKB/Swiss-Prot;Acc:F4JYE9]	16595863	16598682	37.27
AT5G41471	SNOR108	SNOR108; snoRNA [Source:TAIR;Acc:AT5G41471]	16598796	16598905	41.82
AT5G41490		F-box protein At5g41490 [Source:UniProtKB/Swiss-Prot;Acc:Q9FH73]	16600445	16601608	38.92
AT5G41500		Putative F-box protein At5g41500 [Source:UniProtKB/Swiss-Prot;Acc:Q9FFT0]	16604002	16605213	40.1
AT5G41510		Putative F-box protein At5g41510 [Source:UniProtKB/Swiss-Prot;Acc:Q9FFS9]	16607124	16608326	39.15
AT5G41520	RPS10B	40S ribosomal protein S10-2 [Source:UniProtKB/Swiss-Prot;Acc:Q9FFS8]	16609021	16610692	38.28
AT5G41530		Transmembrane protein [Source:UniProtKB/TrEMBL;Acc:Q9FFS7]	16611660	16612088	34.73
AT5G41540		Disease resistance protein (TIR-NBS-LRR class) family [Source:UniProtKB/TrEMBL;Acc:Q9FFS6]	16612659	16616063	39.56
AT5G41550		Disease resistance protein (TIR-NBS-LRR class) family [Source:UniProtKB/TrEMBL;Acc:Q9FFS5]	16617232	16620824	40.19
AT5G41560		At5g41560 [Source:UniProtKB/TrEMBL;Acc:Q9FFS4]	16621197	16622757	35.81
AT5G41570	WRKY24	Probable WRKY transcription factor 24 [Source:UniProtKB/Swiss-Prot;Acc:Q9FFS3]	16624157	16625977	33.06
AT5G41580	PIAL2	E4 SUMO-protein ligase PIAL2 [Source:UniProtKB/Swiss-Prot;Acc:F4JYG0]	16626185	16630820	39.5
AT5G41590		Protein LURP-one-related 17 [Source:UniProtKB/Swiss-Prot;Acc:A0MFL4]	16632086	16634012	33.89
AT5G41600	RTNLB4	Reticulon-like protein B4 [Source:UniProtKB/Swiss-Prot;Acc:Q9FFS0]	16636029	16637908	38.51
AT5G41605		pre-tRNA [Source:TAIR;Acc:AT5G41605]	16638166	16638249	57.14
AT5G41610	CHX18	Cation/H(+) antiporter 18 [Source:UniProtKB/Swiss-Prot;Acc:Q9FFR9]	16638302	16641933	41.41
AT5G41612		other RNA [Source:TAIR;Acc:AT5G41612]	16638352	16641307	43.34
AT5G41620		Uncharacterized protein At5g41620 [Source:UniProtKB/Swiss-Prot;Acc:Q66GQ2]	16646157	16649026	41.22
AT5G06175			16649994	16650138	41.38

AT5G41630		Putative F-box/LRR-repeat protein At5g41630 [Source:UniProtKB/Swiss-Prot;Acc:Q9FFR7]	16650282	16651835	37.58
AT5G41640		UPF0725 protein At5g41640 [Source:UniProtKB/Swiss-Prot;Acc:Q9FFR6]	16653337	16654635	36.03
AT5G41650		Gb [Source:UniProtKB/TrEMBL;Acc:Q9FFR5]	16654913	16655860	37.55
AT5G41660		unknown protein; FUNCTIONS IN: molecular_function unknown; INVOLVED IN: biological_process unknown; LOCATED IN: mitochondrion;	16656708	16658405	42.05
AT5G41663	MIR319B	MIR319/MIR319B; miRNA [Source:TAIR;Acc:AT5G41663]	16660469	16660640	38.95
AT5G41670	PGD3	6-phosphogluconate dehydrogenase, decarboxylating [Source:UniProtKB/TrEMBL;Acc:A0A178UQL8]	16664674	16667390	41.92
AT5G41675		pre-tRNA [Source:TAIR;Acc:AT5G41675]	16667436	16667507	62.5
AT5G41680		Probably inactive receptor-like protein kinase At5g41680 [Source:UniProtKB/Swiss-Prot;Acc:Q3E8J4]	16667769	16669489	42.01
AT5G41685	TOM7-1	Mitochondrial import receptor subunit TOM7-1 [Source:UniProtKB/Swiss-Prot;Acc:Q9ASY8]	16669149	16669723	38.78
AT5G41690		RNA-binding (RRM/RBD/RNP motifs) family protein [Source:UniProtKB/TrEMBL;Acc:Q56XC6]	16669981	16675166	36.41
AT5G41700	UBC8	ubiquitin conjugating enzyme 8 [Source:TAIR;Acc:AT5G41700]	16675582	16677525	34.72
AT5G41720		F-box protein At5g41720 [Source:UniProtKB/Swiss-Prot;Acc:Q6NLE0]	16683165	16683942	39.2
AT5G41730		Protein kinase family protein [Source:UniProtKB/TrEMBL;Acc:Q9FGG5]	16684606	16687706	37.76
AT5G06185			16687519	16687892	32.35
AT5G41740		Disease resistance protein (TIR-NBS-LRR class) family [Source:UniProtKB/TrEMBL;Acc:F4JYI4]	16688626	16693119	40.12
AT5G41750		Disease resistance protein (TIR-NBS-LRR class) family [Source:UniProtKB/TrEMBL;Acc:Q9LSX5]	16693832	16699028	39.47
AT5G41760		CMP-sialic acid transporter 1 [Source:UniProtKB/Swiss-Prot;Acc:Q8LGE9]	16706850	16709909	36.99
AT5G41761		Putative uncharacterized protein [Source:UniProtKB/TrEMBL;Acc:Q1G3G9]	16711042	16712069	32.98
AT5G41763		Uncharacterized protein (Fragment) [Source:UniProtKB/TrEMBL;Acc:A0A1P8BEK1]	16714509	16715882	37.92
AT5G41765		Probable transcription factor At5g41765 [Source:UniProtKB/Swiss-Prot;Acc:F4JYJ1]	16716552	16717558	34.06
AT5G41770		Crooked neck protein, putative / cell cycle protein [Source:UniProtKB/TrEMBL;Acc:F4JZX8]	16717946	16721222	38.91
AT5G41780		Myosin heavy chain-like [Source:UniProtKB/TrEMBL;Acc:Q9FJ36]	16722239	16725040	36.44
AT5G06205			16725542	16726024	36.44
AT5G41790	CIP1	COP1-interactive protein 1 [Source:UniProtKB/Swiss-Prot;Acc:F4JZY1]	16725753	16732847	39.18
AT5G41800		Probable GABA transporter 2 [Source:UniProtKB/Swiss-Prot;Acc:Q8L4X4]	16733663	16736093	39.53
AT5G41810		AT5g41810/K16L22_9 [Source:UniProtKB/TrEMBL;Acc:Q9FJ33]	16738062	16739790	36.38
AT5G41820	RGTA2	Geranylgeranyl transferase type-2 subunit alpha 2 [Source:UniProtKB/Swiss-Prot;Acc:Q9FJ32]	16740593	16743527	39.97
AT5G41830		RNI-like superfamily protein [Source:TAIR;Acc:AT5G41830]	16743636	16745519	37.9
AT5G41840		Putative F-box/LRR-repeat protein At5g41840 [Source:UniProtKB/Swiss-Prot;Acc:Q9FJ30]	16754436	16756263	39.06
AT5G41850		Alpha/beta-Hydrolases superfamily protein [Source:UniProtKB/TrEMBL;Acc:Q9FJ29]	16756698	16758220	40.12
AT5G41860		Transmembrane protein [Source:UniProtKB/TrEMBL;Acc:Q9FJ28]	16757951	16758718	42.84
AT5G06235			16757977	16758829	41.74
AT5G41870		At5g41870 [Source:UniProtKB/TrEMBL;Acc:Q9FJ27]	16758709	16760576	42.51
AT5G06245			16758986	16759343	47.77
AT5G41880	POLA3	DNA primase [Source:UniProtKB/TrEMBL;Acc:F4JZZ1]	16760846	16764276	35.85
AT5G41890		GDSL-like Lipase/Acylhydrolase superfamily protein [Source:TAIR;Acc:AT5G41890]	16764211	16766702	33.95
AT5G41900	BDG2	Probable lysophospholipase BODYGUARD 2 [Source:UniProtKB/Swiss-Prot;Acc:Q9FJ24]	16768935	16771899	36.05
AT5G06255			16773757	16774029	36.26
AT5G06275			16773900	16774225	33.13
AT5G41905	MIR166E	MIR166/MIR166E; miRNA [Source:TAIR;Acc:AT5G41905]	16775520	16775662	37.06
AT5G41908		unknown protein; LOCATED IN: endomembrane system; Ha. [Source:TAIR;Acc:AT5G41908]	16777055	16777525	42.04
AT5G41910	MED10A	Mediator of RNA polymerase II transcription subunit 10a [Source:UniProtKB/Swiss-Prot;Acc:Q9FHZ2]	16777691	16779295	37.13
AT5G41920	SCL23	Scarecrow-like protein 23 [Source:UniProtKB/Swiss-Prot;Acc:Q9FHZ1]	16779643	16781424	42.93
AT5G41940		GTPase activator protein of Rab-like small GTPases-like protein [Source:UniProtKB/TrEMBL;Acc:Q67YN2]	16781858	16785609	40.01
AT5G41950	HLB1	Protein HLB1 [Source:UniProtKB/Swiss-Prot;Acc:Q9FHY8]	16785680	16789676	38.93
AT5G41960		At5g41960 [Source:UniProtKB/TrEMBL;Acc:Q9FHY7]	16789624	16791042	36.43
AT5G41970		Metal-dependent protein hydrolase [Source:UniProtKB/TrEMBL;Acc:F4K000]	16791151	16793261	39.79
AT5G41980		At5g41980 [Source:UniProtKB/TrEMBL;Acc:Q9FHY5]	16793614	16795006	39.99
AT5G41990	WNK8	Serine/threonine-protein kinase WNK8 [Source:UniProtKB/Swiss-Prot;Acc:Q944Q0]	16794967	16798006	39.84
AT5G42000		At5g42000 [Source:UniProtKB/TrEMBL;Acc:Q9FHY3]	16799218	16801102	36.39
AT5G42010		Similarity to unknown protein [Source:UniProtKB/TrEMBL;Acc:Q9FHY2]	16801707	16805242	40.24
AT5G42020	BIP	Heat shock protein 70 (Hsp 70) family protein [Source:TAIR;Acc:AT5G42020]	16807342	16810693	41.83
AT5G42030	ABIL4	ABL interactor-like protein 4 [Source:TAIR;Acc:AT5G42030]	16811307	16813295	35.8
AT5G42040	RPN12B	Putative 26S proteasome non-ATPase regulatory subunit 8 homolog B [Source:UniProtKB/Swiss-Prot;Acc:Q9FHY0]	16813565	16814749	38.99
AT5G42050		DCD (Development and Cell Death) domain protein [Source:UniProtKB/TrEMBL;Acc:Q8RXN8]	16815310	16817346	38.24
AT5G42053		unknown protein; Ha. [Source:TAIR;Acc:AT5G42053]	16817678	16817984	37.79
AT5G42060		Mediator-associated protein 3 [Source:UniProtKB/Swiss-Prot;Acc:Q9FHX8]	16818388	16818955	37.85
AT5G42070		Uncharacterized protein At5g42070 [Source:UniProtKB/TrEMBL;Acc:Q8RWR9]	16818984	16820195	39.6
AT5G42080	DRP1A	RSW9 [Source:UniProtKB/TrEMBL;Acc:A0A178UEJ4]	16820284	16825016	39.26
AT5G42090		Dbj [Source:UniProtKB/TrEMBL;Acc:Q9FHX6]	16826542	16829101	42.89
AT5G42092		Potential natural antisense gene, locus overlaps with AT5G42090 [Source:TAIR;Acc:AT5G42092]	16826639	16828529	42.25
AT5G42100	ATBG_PPAP	Glucan endo-1,3-beta-glucosidase 10 [Source:UniProtKB/Swiss-Prot;Acc:Q9FHX5]	16829113	16831618	44.81
AT5G42110		Putative uncharacterized protein [Source:UniProtKB/TrEMBL;Acc:Q8GW66]	16831696	16832357	40.63
AT5G42120	LECRKS6	L-type lectin-domain containing receptor kinase S.6 [Source:UniProtKB/Swiss-Prot;Acc:Q9FHX3]	16833052	16835169	47.4
AT5G42130	MFL1	Protein MITOFERRINLIKE 1, chloroplastic [Source:UniProtKB/Swiss-Prot;Acc:Q9FHX2]	16835233	16836895	42.99
AT5G42140		Regulator of chromosome condensation (RCC1) family with FYVE zinc finger domain [Source:TAIR;Acc:AT5G42140]	16837332	16842493	40.74
AT5G06285			16841930	16842396	44.54
AT5G42146		At5g42146 [Source:UniProtKB/TrEMBL;Acc:Q2V323]	16844336	16845015	37.35
AT5G42150		Glutathione S-transferase family protein [Source:UniProtKB/TrEMBL;Acc:Q9FHX0]	16846150	16848568	38.24
AT5G42170		GDSL esterase/lipase At5g42170 [Source:UniProtKB/Swiss-Prot;Acc:Q9FHW9]	16848624	16852221	28.02
AT5G42180	PER64	Peroxidase [Source:UniProtKB/TrEMBL;Acc:A0A178UJR4]	16852628	16854224	39.07
AT5G42190	SKP1B	SKP1-like protein 1B [Source:UniProtKB/Swiss-Prot;Acc:Q9FHW7]	16854191	16855741	37.46
AT5G42200	ATL23	E3 ubiquitin-protein ligase ATL23 [Source:UniProtKB/Swiss-Prot;Acc:Q8L9W3]	16860286	16861290	40.3
AT5G42210		Major facilitator superfamily protein [Source:TAIR;Acc:AT5G42210]	16865770	16870887	30.97
AT5G42220		AT5g42220/K5J14_2 [Source:UniProtKB/TrEMBL;Acc:Q8L745]	16872044	16878383	38.44
AT5G42223		Putative defensin-like protein 114 [Source:UniProtKB/Swiss-Prot;Acc:Q2V322]	16878562	16878956	31.65
AT5G42230	SCPL41	Serine carboxypeptidase-like 41 [Source:UniProtKB/Swiss-Prot;Acc:Q9FH06]	16880592	16884462	37.77
AT5G42232		Defensin-like protein 115 [Source:UniProtKB/Swiss-Prot;Acc:Q2V321]	16884940	16885439	30.2
AT5G42235		Defensin-like protein 116 [Source:UniProtKB/Swiss-Prot;Acc:Q3E8I8]	16886903	16887570	30.39
AT5G42240	SCPL42	Serine carboxypeptidase-like 42 [Source:UniProtKB/Swiss-Prot;Acc:Q9FH05]	16887626	16891192	38.07
AT5G42242	LCR57	Putative defensin-like protein 179 [Source:UniProtKB/Swiss-Prot;Acc:P82771]	16891188	16891909	34.63
AT5G42250		Alcohol dehydrogenase-like 7 [Source:UniProtKB/Swiss-Prot;Acc:Q9FH04]	16893849	16897690	33.52
AT5G42260	BGLU12	Beta-glucosidase 12 [Source:UniProtKB/Swiss-Prot;Acc:Q9FH03]	16898712	16900317	40.29
AT5G42270	FTSH5	VAR1 [Source:UniProtKB/TrEMBL;Acc:A0A178U8J6]	16902475	16905294	41.81
AT5G42280		Cysteine/Histidine-rich C1 domain family protein [Source:UniProtKB/TrEMBL;Acc:F4K1I0]	16905202	16908059	40.69
AT5G42300	UBL5	UBL5 [Source:UniProtKB/TrEMBL;Acc:A0A384KQX3]	16911579	16913991	38
AT5G42290		At5g42290 [Source:UniProtKB/TrEMBL;Acc:Q9FH00]	16911580	16912198	44.43
AT5G42310	CRP1	Pentatricopeptide repeat-containing protein At5g42310, chloroplastic [Source:UniProtKB/Swiss-Prot;Acc:Q8L844]	16915728	16918569	41.17
AT5G42320		Zn-dependent exopeptidases superfamily protein [Source:TAIR;Acc:AT5G42320]	16918489	16921596	38.06
AT5G06295			16920835	16921229	35.95
AT5G42325		Transcription factor IIS protein [Source:UniProtKB/TrEMBL;Acc:F4K1I5]	16924102	16924851	43.33
AT5G42330		At5g42330 [Source:UniProtKB/TrEMBL;Acc:Q6GKX1]	16926608	16927580	40.29
AT5G42340	PUB15	U-box domain-containing protein 15 [Source:UniProtKB/Swiss-Prot;Acc:Q681N2]	16927893	16930532	37.54
AT5G42350		F-box/kelch-repeat protein At5g42350 [Source:UniProtKB/Swiss-Prot;Acc:Q9FII2]	16931531	16934031	40.58
AT5G42360		F-box/kelch-repeat protein At5g42360 [Source:UniProtKB/Swiss-Prot;Acc:Q9FII1]	16936000	16938525	40.82
AT5G42370		Calcineurin-like metallo-phosphoesterase superfamily protein [Source:UniProtKB/TrEMBL;Acc:F4K1J1]	16938981	16941754	37.56
AT5G42380	CML37	Calcium-binding protein CML37 [Source:UniProtKB/Swiss-Prot;Acc:Q9FIH9]	16942447	16943820	40.1
AT5G42390	SPP	Stromal processing peptidase, chloroplastic [Source:UniProtKB/Swiss-Prot;Acc:Q9FIH8]	16945165	16953353	38.86
AT5G42400	ATXR7	SET domain protein 25 [Source:TAIR;Acc:AT5G42400]	16953515	16961245	37.29
AT5G42410		SAUR-like auxin-responsive protein family [Source:UniProtKB/TrEMBL;Acc:Q9FIH6]	16964650	16965261	33.5
AT5G42420	URGT3	UDP-rhamnose/UDP-galactose transporter 3 [Source:UniProtKB/Swiss-Prot;Acc:Q9FIH5]	16968655	16970527	38.71
AT5G42430		Putative F-box protein At5g42430 [Source:UniProtKB/Swiss-Prot;Acc:Q9FIH4]	16971312	16972499	38.55
AT5G42440		At5g42440 [Source:UniProtKB/TrEMBL;Acc:Q9FIH3]	16973202	16974877	42.06
AT5G06305			16975699	16975782	42.86
AT5G42450	PCMP-E102	Pentatricopeptide repeat-containing protein At5g42450, mitochondrial [Source:UniProtKB/Swiss-Prot;Acc:Q9FIH2]	16977207	16979073	40.01

AT5G42460		F-box protein At5g42460 [Source:UniProtKB/Swiss-Prot;Acc:Q9FIH1]	16979773	16981195	39.35
AT5G42470		At5g42470 [Source:UniProtKB/TrEMBL;Acc:Q5XF81]	16982149	16984933	36.91
AT5G42480	ARC6	Protein ACCUMULATION AND REPLICATION OF CHLOROPLASTS 6, chloroplastic [Source:UniProtKB/Swiss-Prot;Acc:Q9FIG9]	16985096	16988796	41.77
AT5G42490		ATP binding microtubule motor family protein [Source:TAIR;Acc:AT5G42490]	16988280	16992754	40.27
AT5G42500	DIR2	Dirigent protein 2 [Source:UniProtKB/Swiss-Prot;Acc:Q9FIG7]	16994106	16995062	38.66
AT5G42504		unknown protein; Ha. [Source:TAIR;Acc:AT5G42504]	16996900	16997127	47.37
AT5G42510	DIR1	Dirigent protein 1 [Source:UniProtKB/Swiss-Prot;Acc:Q9FIG6]	16997979	16998925	40.02
AT5G42520	BPC6	BPC6 [Source:UniProtKB/TrEMBL;Acc:A0A178UIB6]	17000259	17002607	40.44
AT5G42530		Uncharacterized protein At5g42530; MDH9.23 [Source:UniProtKB/TrEMBL;Acc:Q8W483]	17004738	17005510	37.26
AT5G42540	XRN2	5'-3' exoribonuclease [Source:UniProtKB/TrEMBL;Acc:F4K1L3]	17007366	17014846	36.57
AT5G42560	HVA22I	HVA22-like protein i [Source:UniProtKB/Swiss-Prot;Acc:Q8LE10]	17014847	17017252	38.03
AT5G42567		Protein of unknown function (DUF784) [Source:TAIR;Acc:AT5G42567]	17018034	17018466	34.18
AT5G42570		At5g42570 [Source:UniProtKB/TrEMBL;Acc:Q93XZ7]	17021218	17022723	37.25
AT5G42580	CYP705A12	Cytochrome P450 705A12 [Source:UniProtKB/Swiss-Prot;Acc:Q9FH67]	17023502	17025361	40.54
AT5G42590	CYP71A16	Cytochrome P450 71A16 [Source:UniProtKB/Swiss-Prot;Acc:Q9FH66]	17031024	17033800	35.76
AT5G06325			17049733	17050099	41.96
AT5G06335			17049733	17050241	38.9
AT5G42600	MRN1	Marneral synthase [Source:UniProtKB/Swiss-Prot;Acc:Q9FJV8]	17053453	17058245	35.41
AT5G42610		Calcium uniporter protein 4, mitochondrial [Source:UniProtKB/Swiss-Prot;Acc:Q9FJV7]	17062455	17064047	34.84
AT5G42620		Metalloendopeptidase / zinc ion binding protein [Source:UniProtKB/TrEMBL;Acc:F4K306]	17066197	17071963	38.32
AT5G42630	KAN4	Probable transcription factor KAN4 [Source:UniProtKB/Swiss-Prot;Acc:Q9FJV5]	17073673	17075904	33.6
AT5G42635		Glycine-rich protein [Source:UniProtKB/TrEMBL;Acc:F4K310]	17076227	17076725	65.93
AT5G06345			17084174	17084754	33.22
AT5G06365			17084449	17084687	33.89
AT5G42640		C2H2 and C2HC zinc fingers superfamily protein [Source:UniProtKB/TrEMBL;Acc:Q9FJV4]	17088695	17089597	34.66
AT5G42650	CYP74A	Allene oxide synthase, chloroplastic [Source:UniProtKB/Swiss-Prot;Acc:Q96242]	17097564	17099595	43.6
AT5G42655		Disease resistance-responsive (dirigent-like protein) family protein [Source:TAIR;Acc:AT5G42655]	17102027	17102896	41.84
AT5G42660		AT5g42660/MJB21_3 [Source:UniProtKB/TrEMBL;Acc:Q93Z91]	17102924	17106103	37.2
AT5G42670		Agenet domain-containing protein [Source:TAIR;Acc:AT5G42670]	17107855	17110761	35.57
AT5G42680		At5g42680 [Source:UniProtKB/TrEMBL;Acc:Q9FMZ6]	17114098	17115571	37.25
AT5G42690		Expressed protein [Source:UniProtKB/TrEMBL;Acc:Q45GG2]	17116376	17119892	34.92
AT5G42700		AP2/B3-like transcriptional factor family protein [Source:TAIR;Acc:AT5G42700]	17122102	17124441	35.26
AT5G42710		Putative uncharacterized protein [Source:UniProtKB/TrEMBL;Acc:Q9FMZ3]	17125455	17128934	37.27
AT5G42720		Glycosyl hydrolase family 17 protein [Source:UniProtKB/TrEMBL;Acc:Q8VY12]	17130186	17132909	38.51
AT5G42740	PGIC	Glucose-6-phosphate isomerase [Source:UniProtKB/TrEMBL;Acc:A0A178UPP5]	17135661	17141173	37.4
AT5G42750	BKI1	BRI1 kinase inhibitor 1 [Source:UniProtKB/Swiss-Prot;Acc:Q9FMZ0]	17142816	17144672	39.42
AT5G42760		Leucine carboxyl methyltransferase [Source:UniProtKB/TrEMBL;Acc:F4K328]	17148808	17150451	40.75
AT5G06385			17149832	17150219	45.1
AT5G42765		INVOLVED IN: biological_process unknown; LOCATED IN: thylakoid, chloroplast thylakoid membrane, chloroplast; EXPRESSED IN: 22 plan	17150238	17151681	37.12
AT5G42770		Maf-like protein [Source:UniProtKB/TrEMBL;Acc:F4K330]	17151988	17154618	36.72
AT5G42780	ZHD13	Zinc-finger homeodomain protein 13 [Source:UniProtKB/Swiss-Prot;Acc:Q9FMY7]	17154718	17155757	42.69
AT5G42785		Transmembrane protein [Source:UniProtKB/TrEMBL;Acc:Q1PDN7]	17156853	17157857	36.02
AT5G42790	PAF1	Proteasome subunit alpha type [Source:UniProtKB/TrEMBL;Acc:A0A178UFX8]	17159044	17161156	40.09
AT5G42797	LCR28	Defensin-like protein 130 [Source:UniProtKB/Swiss-Prot;Acc:P82743]	17163196	17163574	31.13
AT5G42800	DFRA	Dihydroflavonol reductase [Source:UniProtKB/TrEMBL;Acc:B1GV15]	17164093	17166032	38.04
AT5G42810	IPK1	Inositol-pentakisphosphate 2-kinase [Source:UniProtKB/Swiss-Prot;Acc:Q93YN9]	17166702	17169459	36.48
AT5G42820	U2AF35B	U2AF35B [Source:UniProtKB/TrEMBL;Acc:A0A178UM74]	17169283	17172787	39.86
AT5G06395			17170305	17171188	46.72
AT5G42825		At5g42825 [Source:UniProtKB/TrEMBL;Acc:Q94AK0]	17171807	17172800	36.82
AT5G42830		HXXXD-type acyl-transferase family protein [Source:UniProtKB/TrEMBL;Acc:Q9FMN6]	17176293	17178130	39.45
AT5G42840		CHP-rich zinc finger protein-like [Source:UniProtKB/TrEMBL;Acc:Q9FMN5]	17179073	17181365	39.9
AT5G42850		Thioredoxin-like protein Clot [Source:UniProtKB/Swiss-Prot;Acc:Q9FMN4]	17181412	17182743	38.89
AT5G42860		Emb [Source:UniProtKB/TrEMBL;Acc:Q9FMN3]	17183061	17185032	38.13
AT5G42870	PAH2	Phosphatidate phosphatase PAH2 [Source:UniProtKB/Swiss-Prot;Acc:Q9FMN2]	17185440	17189978	38.8
AT5G06405			17190459	17190698	33.75
AT5G06415			17191309	17191517	37.8
AT5G42880	WEL3	Protein WEAK CHLOROPLAST MOVEMENT UNDER BLUE LIGHT-like 3 [Source:UniProtKB/Swiss-Prot;Acc:Q9FMN1]	17191499	17194131	42.01
AT5G42890	ATSCP2	SCP2 [Source:UniProtKB/TrEMBL;Acc:A0A178UHV6]	17194170	17196093	35.14
AT5G42895		ECA1 gametogenesis related family protein [Source:UniProtKB/TrEMBL;Acc:A8MQ80]	17196762	17197163	38.06
AT5G42900	COR27	Cold regulated protein 27 [Source:UniProtKB/TrEMBL;Acc:Q8L8T7]	17197641	17199910	35.11
AT5G42905		Polynucleotidyl transferase, ribonuclease H-like superfamily protein [Source:UniProtKB/TrEMBL;Acc:F4K355]	17201414	17202323	46.59
AT5G42910	BZIP15	ABSCISIC ACID-INSENSITIVE 5-like protein 8 [Source:UniProtKB/Swiss-Prot;Acc:Q9FMM7]	17203742	17205380	36.97
AT5G42920	THO5B	THO complex subunit 5B [Source:UniProtKB/Swiss-Prot;Acc:F4K4J0]	17205971	17209650	40.43
AT5G42930		Alpha/beta-Hydrolases superfamily protein [Source:UniProtKB/TrEMBL;Acc:F4K4J2]	17210533	17214220	31.97
AT5G42940	RHG1A	Probable E3 ubiquitin-protein ligase RHG1A [Source:UniProtKB/Swiss-Prot;Acc:Q9FMM4]	17216329	17220672	39.53
AT5G06425			17220757	17220975	37.44
AT5G42950		GYF domain-containing protein [Source:UniProtKB/TrEMBL;Acc:Q9FMM3]	17224270	17231384	43.51
AT5G42955		Protein of unknown function (DUF784) [Source:TAIR;Acc:AT5G42955]	17231842	17232324	33.13
AT5G42957		Protein of unknown function (DUF784) [Source:TAIR;Acc:AT5G42957]	17233599	17234132	32.02
AT5G42960	OEP24B	Outer envelope pore protein 24B, chloroplastic [Source:UniProtKB/Swiss-Prot;Acc:Q8H0Y1]	17235092	17236679	38.85
AT5G42965		Polynucleotidyl transferase, ribonuclease H-like superfamily protein [Source:UniProtKB/TrEMBL;Acc:Q3E8I3]	17236736	17237139	39.36
AT5G42970	CSN4	At5g42970 [Source:UniProtKB/TrEMBL;Acc:Q1H5B6]	17237153	17240816	36.71
AT5G42980	TRX3	Thioredoxin H3 [Source:UniProtKB/Swiss-Prot;Acc:Q42403]	17242607	17244231	36.18
AT5G42990	UBC18	Probable ubiquitin-conjugating enzyme E2 18 [Source:UniProtKB/Swiss-Prot;Acc:Q9FMM0]	17243987	17245988	35.31
AT5G43000		Putative uncharacterized protein [Source:UniProtKB/TrEMBL;Acc:Q9FML9]	17246354	17247202	42.87
AT5G43010	RPT4A	26S proteasome regulatory subunit 10B homolog A [Source:UniProtKB/Swiss-Prot;Acc:Q9SEI3]	17248290	17251200	38.92
AT5G43020		Leucine-rich repeat protein kinase family protein [Source:UniProtKB/TrEMBL;Acc:Q9FMI2]	17255205	17258077	41.18
AT5G43030		CHP-rich zinc finger protein-like [Source:UniProtKB/TrEMBL;Acc:Q9FMI1]	17258927	17260784	41.39
AT5G06435			17261765	17262121	38.66
AT5G43040		CHP-rich zinc finger protein-like [Source:UniProtKB/TrEMBL;Acc:Q9FMI0]	17263685	17265777	41.52
AT5G43050	NPQ6	At5g43050 [Source:UniProtKB/TrEMBL;Acc:Q9FMH9]	17267838	17269172	37.83
AT5G43060	RD21B	Probable cysteine protease RD21B [Source:UniProtKB/Swiss-Prot;Acc:Q9FMH8]	17269310	17272298	37.4
AT5G43064		Transmembrane protein [Source:UniProtKB/TrEMBL;Acc:A0A1P8BER4]	17282272	17282490	48.86
AT5G06445			17282739	17283132	34.52
AT5G43066		unknown protein; LOCATED IN: endomembrane system; Ha. [Source:TAIR;Acc:AT5G43066]	17284583	17284807	47.56
AT5G06455			17285168	17285423	43.75
AT5G06465			17286563	17287137	37.74
AT5G43068		Transmembrane protein [Source:UniProtKB/TrEMBL;Acc:A0A1P8BEV7]	17286835	17287354	37.12
AT5G43070	WPP1	WPP domain-containing protein 1 [Source:UniProtKB/Swiss-Prot;Acc:Q9FMH6]	17288965	17289958	39.24
AT5G43080	CYCA3-1	Putative cyclin-A3-1 [Source:UniProtKB/Swiss-Prot;Acc:Q9FMH5]	17292952	17295024	36.18
AT5G43090	APUM13	Putative pumilio homolog 13 [Source:UniProtKB/Swiss-Prot;Acc:Q9FMH4]	17295933	17298214	37.99
AT5G43100		Eukaryotic aspartyl protease family protein [Source:UniProtKB/TrEMBL;Acc:F4K4L3]	17298814	17303058	39.01
AT5G43110	APUM14	Pumilio homolog 14 [Source:UniProtKB/Swiss-Prot;Acc:Q4PSD1]	17309842	17311937	38.84
AT5G43120		ARM-repeat/Tetratricopeptide repeat (TPR)-like protein [Source:TAIR;Acc:AT5G43120]	17312543	17314579	40.7
AT5G43130	TAF4B	Transcription initiation factor TFIID subunit 4b [Source:UniProtKB/Swiss-Prot;Acc:F4K4L7]	17315326	17321231	38.25
AT5G43140		At5g43140 [Source:UniProtKB/TrEMBL;Acc:A0JQ86]	17321395	17323484	36.84
AT5G43150		At5g43150 [Source:UniProtKB/TrEMBL;Acc:Q9FMG8]	17324909	17325987	36.98
AT5G06475			17326369	17326864	34.88
AT5G43155		QWRF motif protein [Source:UniProtKB/TrEMBL;Acc:A0A1P8BC48]	17328460	17329240	45.71
AT5G43160	QWRF9	Family of unknown function (DUF566) [Source:TAIR;Acc:AT5G43160]	17329275	17331352	40.33
AT5G43170	AZF3	Zinc finger protein AZF3 [Source:UniProtKB/Swiss-Prot;Acc:Q9SSW0]	17330945	17331646	47.15
AT5G43175	BHLH139	Transcription factor bHLH139 [Source:UniProtKB/Swiss-Prot;Acc:Q3E7L7]	17334121	17335234	36.71
AT5G43180		At5g43180 [Source:UniProtKB/TrEMBL;Acc:Q9LUH6]	17336291	17337884	33.75
AT5G43185		At5g43185 [Source:UniProtKB/TrEMBL;Acc:Q3E7J3]	17338299	17338895	30.99
AT5G43190		F-box/kelch-repeat protein At5g43190 [Source:UniProtKB/Swiss-Prot;Acc:Q9FHS6]	17340071	17341814	41.28

	AT5G06495			17345360	17345672	39.3
	AT5G43200		Gb [Source:UniProtKB/TrEMBL;Acc:Q9FHS5]	17346141	17346829	42.67
	AT5G43210		Emb [Source:UniProtKB/TrEMBL;Acc:Q9FHS4]	17347565	17348746	38.32
	AT5G43211		unknown protein; LOCATED IN: endomembrane system; Ha. [Source:TAIR;Acc:AT5G43211]	17348722	17348886	37.58
	AT5G43230		unknown protein; BEST Arabidopsis thaliana protein match is: unknown protein (TAIR:AT3G01810.3); Ha. [Source:TAIR;Acc:AT5G43230]	17348876	17352933	38.66
MQTL-7/Chr5	AT5G46020		28 kDa heat/acid-stable phosphoprotein-like protein [Source:UniProtKB/TrEMBL;Acc:Q9FNM0]	18662805	18664908	35.98
	AT5G46025		Ras-related small GTP-binding family protein [Source:UniProtKB/TrEMBL;Acc:Q3E8G1]	18665255	18665596	40.64
	AT5G46030		Transcription elongation factor 1 homolog [Source:UniProtKB/Swiss-Prot;Acc:Q8LEF3]	18670051	18671461	35.51
	AT5G46040	NPF5.3	Protein NRT1/ PTR FAMILY 5.3 [Source:UniProtKB/Swiss-Prot;Acc:Q9FNL8]	18671397	18673551	39.86
	AT5G46050	NPF5.2	Protein NRT1/ PTR FAMILY 5.2 [Source:UniProtKB/Swiss-Prot;Acc:Q9FNL7]	18674968	18679191	33.55
	AT5G46060		Emb [Source:UniProtKB/TrEMBL;Acc:Q9FNL6]	18681326	18682708	35.72
	AT5G46070		Guanylate-binding family protein [Source:UniProtKB/TrEMBL;Acc:F4KG14]	18683319	18688822	39.14
	AT5G46080		Protein kinase superfamily protein [Source:UniProtKB/TrEMBL;Acc:Q9FNL4]	18689361	18690857	37.27
	AT5G46085			18693316	18693567	34.92
	AT5G46090	DMP6	Protein DMP6 [Source:UniProtKB/Swiss-Prot;Acc:Q9FNL3]	18693485	18694129	41.86
	AT5G46100		Pentatricopeptide repeat-containing protein At5g46100 [Source:UniProtKB/Swiss-Prot;Acc:Q9FNL2]	18694316	18695856	39.97
	AT5G46110	APE2	Glucose-6-phosphate/phosphate translocator-like protein [Source:UniProtKB/TrEMBL;Acc:A0A219I0W9]	18697235	18700900	36.85
	AT5G46105		pre-tRNA [Source:TAIR;Acc:AT5G46105]	18698662	18698733	58.33
	AT5G46115		Putative uncharacterized protein [Source:UniProtKB/TrEMBL;Acc:Q3E8G0]	18701246	18701852	34.27
	AT5G46130		Putative uncharacterized protein [Source:UniProtKB/TrEMBL;Acc:Q9FNK9]	18702914	18704484	37.56
	AT5G46140		Putative uncharacterized protein [Source:UniProtKB/TrEMBL;Acc:Q5XV21]	18704997	18706708	36.62
	AT5G46150	ALIS2	Putative ALA-interacting subunit 2 [Source:UniProtKB/Swiss-Prot;Acc:Q67YS6]	18707546	18710722	36.39
	AT5G46160	HLP	50S ribosomal protein HLP, mitochondrial [Source:UniProtKB/Swiss-Prot;Acc:Q93Z17]	18710851	18712559	37.8
	AT5G46170		F-box protein At5g46170 [Source:UniProtKB/Swiss-Prot;Acc:Q9FNK5]	18714864	18717031	39.53
	AT5G46180	DELTA-OAT	Ornithine aminotransferase, mitochondrial [Source:UniProtKB/Swiss-Prot;Acc:Q9FNK4]	18718417	18721744	36.69
	AT5G46190		RNA-binding KH domain-containing protein [Source:TAIR;Acc:AT5G46190]	18721752	18726249	38.08
	AT5G46200		Carboxyl-terminal proteinase-like protein (DUF239) [Source:UniProtKB/TrEMBL;Acc:Q9FGP1]	18727802	18730331	31.46
	AT5G46210	CUL4	Cullin-4 [Source:UniProtKB/Swiss-Prot;Acc:Q8LGH4]	18731371	18736815	37.83
	AT5G46220		At5g46220 [Source:UniProtKB/TrEMBL;Acc:Q6DBD7]	18738697	18742126	32.22
	AT5G46230		At5g46230 [Source:UniProtKB/TrEMBL;Acc:Q9FL37]	18742272	18743077	35.24
	AT5G46240	KAT1	At5g46240 [Source:UniProtKB/TrEMBL;Acc:Q67YG3]	18743510	18747406	37.03
	AT5G06785			18747608	18747881	46.35
	AT5G46250	LARP6A	La-related protein 6A [Source:UniProtKB/Swiss-Prot;Acc:Q94A38]	18755280	18758333	37.75
	AT5G46260		Disease resistance protein (TIR-NBS-LRR class) family [Source:UniProtKB/TrEMBL;Acc:Q9FL35]	18758699	18763503	37.67
	AT5G46270		Disease resistance protein (TIR-NBS-LRR class) family [Source:TAIR;Acc:AT5G46270]	18763972	18769090	38.05
	AT5G06795			18769157	18769416	32.31
	AT5G46280	MCM3	DNA replication licensing factor MCM3 [Source:UniProtKB/Swiss-Prot;Acc:Q9FL33]	18769649	18773706	38.47
	AT5G46290	KASI	3-ketoacyl-acyl carrier protein synthase I [Source:UniProtKB/TrEMBL;Acc:F4KHF4]	18774056	18776839	40.34
	AT5G46295		Transmembrane protein [Source:UniProtKB/TrEMBL;Acc:Q3E8F8]	18779790	18780205	32.21
	AT5G46297			18781172	18781838	31.48
	AT5G46300		Putative uncharacterized protein [Source:UniProtKB/TrEMBL;Acc:Q9FL31]	18783585	18784786	30.45
	AT5G46310		WRKY family transcription factor [Source:UniProtKB/TrEMBL;Acc:Q9FL30]	18785672	18786956	39.3
	AT5G46315	U6-29	U6-29; snRNA [Source:TAIR;Acc:AT5G46315]	18787079	18787990	38.05
	AT5G46320		MADS-box family protein [Source:UniProtKB/TrEMBL;Acc:Q9FL29]	18788086	18788910	32.85
	AT5G46325		pre-tRNA [Source:TAIR;Acc:AT5G46325]	18790308	18790387	56.25
	AT5G46330	FLS2	Leucine-rich repeat receptor-like protein kinase (Fragment) [Source:UniProtKB/TrEMBL;Acc:C0LGU8]	18791575	18795908	41.07
	AT5G46340	RWA1	RWA1 [Source:UniProtKB/TrEMBL;Acc:A0A178UNJ7]	18795737	18800122	37.94
	AT5G46350	WRKY8	WRKY transcription factor 8 [Source:UniProtKB/Swiss-Prot;Acc:Q9FL26]	18801164	18804056	31.01
	AT5G06815			18804617	18804717	43.56
	AT5G46360	KCO3	Ca2+ activated outward rectifying K+ channel 3 [Source:TAIR;Acc:AT5G46360]	18806563	18808224	36.34
	AT5G46370	TPK2	KCO2 [Source:UniProtKB/TrEMBL;Acc:A0A178UQZ6]	18809496	18811870	38.11
	AT5G46380		Kinase-related protein of unknown function (DUF1296) [Source:TAIR;Acc:AT5G46380]	18813088	18816431	39.38
	AT5G46390	CTPA1	Carboxyl-terminal-processing peptidase 1, chloroplastic [Source:UniProtKB/Swiss-Prot;Acc:F4KHG6]	18816585	18819462	37.18
	AT5G46395		Transmembrane protein [Source:UniProtKB/TrEMBL;Acc:A0A1P8BAC6]	18819464	18819755	53.42
	AT5G46400	AT PRP39-2	Tetratricopeptide repeat (TPR)-like superfamily protein [Source:UniProtKB/TrEMBL;Acc:F4KHG8]	18819933	18824782	38.54
	AT5G46410	SSP4	SCP1-like small phosphatase 4 [Source:UniProtKB/TrEMBL;Acc:F4KHG9]	18825100	18829624	36.73
	AT5G46420		16S rRNA processing protein RimM family [Source:UniProtKB/TrEMBL;Acc:Q9FHG3]	18829436	18833060	38.9
	AT5G46430	RPL32B	60S ribosomal protein L32-2 [Source:UniProtKB/Swiss-Prot;Acc:Q9FHG2]	18833240	18834632	38.62
	AT5G06825			18833820	18833896	37.66
	AT5G46440		At5g46440 [Source:UniProtKB/TrEMBL;Acc:Q9FHG1]	18834643	18835162	41.35
	AT5G46450		Disease resistance protein (TIR-NBS-LRR class) family [Source:UniProtKB/TrEMBL;Acc:Q9FHG0]	18835605	18840058	38.84
	AT5G46460	PCMP-H49	Pentatricopeptide repeat-containing protein At5g46460, mitochondrial [Source:UniProtKB/Swiss-Prot;Acc:Q9FHF9]	18840196	18842479	41.42
	AT5G46470	RPS6	Disease resistance protein RPS6 [Source:UniProtKB/Swiss-Prot;Acc:F4KHH8]	18842553	18849741	37.85
	AT5G46490		Disease resistance protein (TIR-NBS-LRR class) family [Source:UniProtKB/TrEMBL;Acc:Q9FHF6]	18850645	18853896	37.12
	AT5G46500		Protein VARIATION IN COMPOUND TRIGGERED ROOT growth protein [Source:UniProtKB/TrEMBL;Acc:Q9FHF5]	18855955	18858552	35.22
	AT5G46510		Disease resistance protein (TIR-NBS-LRR class) family [Source:UniProtKB/TrEMBL;Acc:Q9FHF4]	18860234	18867015	38.53
	AT5G06835			18866218	18866362	37.93
	AT5G46520		Disease resistance protein (TIR-NBS-LRR class) family [Source:TAIR;Acc:AT5G46520]	18867616	18872494	38.9
	AT5G46530		AWPM-19-like family protein [Source:UniProtKB/TrEMBL;Acc:F4KHI4]	18875382	18876928	32.26
	AT5G46540	ABCB7	ABC transporter B family member 7 [Source:UniProtKB/Swiss-Prot;Acc:Q9FHF1]	18877090	18882462	39.33
	AT5G46550	GTE12	Transcription factor GTE12 [Source:UniProtKB/Swiss-Prot;Acc:Q9LS28]	18884218	18886960	36.6
	AT5G46560		CONTAINS InterPro DOMAIN/s: Inner nuclear membrane protein MAN1 (InterPro:IPR018996); Ha. [Source:TAIR;Acc:AT5G46560]	18887987	18890517	35.72
	AT5G06845			18891003	18891437	35.4
	AT5G06855			18891988	18892294	33.88
	AT5G46570	BSK2	Serine/threonine-protein kinase BSK2 [Source:UniProtKB/Swiss-Prot;Acc:Q9LS26]	18894378	18897406	38.3
	AT5G46580		Pentatricopeptide repeat-containing protein At5g46580, chloroplastic [Source:UniProtKB/Swiss-Prot;Acc:Q9LS25]	18897445	18899646	40.96
	AT5G06875			18903952	18904338	35.14
	AT5G46590	anac096	NAC domain-containing protein 96 [Source:UniProtKB/Swiss-Prot;Acc:Q9LS24]	18905258	18907052	33.93
	AT5G46595		pre-tRNA [Source:TAIR;Acc:AT5G46595]	18907523	18907594	55.56
	AT5G46600	ALMT13	Aluminum-activated malate transporter 13 [Source:UniProtKB/Swiss-Prot;Acc:Q9LS23]	18907639	18910838	35.25
	AT5G46610	ALMT14	Aluminum-activated malate transporter 14 [Source:UniProtKB/Swiss-Prot;Acc:Q9LS22]	18912667	18915779	35.3
	AT5G46620		At5g46620 [Source:UniProtKB/TrEMBL;Acc:Q8GYV3]	18917570	18919442	36.73
	AT5G46630		Clathrin adaptor complexes medium subunit family protein [Source:UniProtKB/TrEMBL;Acc:F4KHJ7]	18920334	18923551	37.51
	AT5G46640	AHL8	AT-hook motif nuclear-localized protein 8 [Source:UniProtKB/Swiss-Prot;Acc:Q9FIR1]	18924429	18926984	38.85
	AT5G46650	ATL30	RING-H2 finger protein ATL30 [Source:UniProtKB/Swiss-Prot;Acc:Q9FIR0]	18930246	18931312	39.55
	AT5G46660		CHP-rich zinc finger protein [Source:UniProtKB/TrEMBL;Acc:Q9FIQ9]	18932181	18933391	39.97
	AT5G46670		Cysteine/Histidine-rich C1 domain family protein [Source:UniProtKB/TrEMBL;Acc:Q9FIQ8]	18938847	18940166	40
	AT5G46680		Pentatricopeptide repeat-containing protein At5g46680 [Source:UniProtKB/Swiss-Prot;Acc:Q56XR6]	18940903	18942672	38.87
	AT5G46690	BHLH71	Transcription factor bHLH71 [Source:UniProtKB/Swiss-Prot;Acc:Q56XR0]	18945543	18947637	34.89
	AT5G46700	TRN2	TRN2 [Source:UniProtKB/TrEMBL;Acc:A0A178ULL6]	18950843	18953127	35.4
	AT5G46710		At5g46710 [Source:UniProtKB/TrEMBL;Acc:Q9FIQ4]	18952603	18954692	35.22
	AT5G46720		AIG2-like (Avirulence induced gene) family protein [Source:UniProtKB/TrEMBL;Acc:Q9FIQ3]	18959094	18959852	39.26
	AT5G46730		Glycine-rich protein [Source:UniProtKB/TrEMBL;Acc:Q7Y218]	18963977	18965222	51.52
	AT5G46740	UBP21	Ubiquitin carboxyl-terminal hydrolase 21 [Source:UniProtKB/Swiss-Prot;Acc:Q9FIQ1]	18965271	18968798	38.63
	AT5G46750	AGD9	AGD9 [Source:UniProtKB/TrEMBL;Acc:A0A178UAW4]	18969736	18971981	37.58
	AT5G46760	MYC3	Transcription factor MYC3 [Source:UniProtKB/Swiss-Prot;Acc:Q9FIP9]	18974022	18976590	40.76
	AT5G46770		AT5g46770/MZA15_19 [Source:UniProtKB/TrEMBL;Acc:Q9FIP8]	18977399	18978246	36.2
	AT5G46780		At5g46780 [Source:UniProtKB/TrEMBL;Acc:Q9FIP7]	18978953	18980183	38.59
	AT5G06880			18982793	18983022	34.35
	AT5G46790	PYL1	Abscisic acid receptor PYL1 [Source:UniProtKB/Swiss-Prot;Acc:Q8VZS8]	18983540	18984923	35.19
	AT5G46795	MSP2	Microspore-specific promoter 2 [Source:UniProtKB/TrEMBL;Acc:Q1PDM1]	18986936	18988197	32.49
	AT5G46800	BOU	Mitochondrial carnitine/acylcarnitine carrier-like protein [Source:UniProtKB/Swiss-Prot;Acc:Q93XM7]	18988588	18990867	38.68
	AT5G46810		Protein of Unknown Function (DUF239) [Source:TAIR;Acc:AT5G46810]	18991164	18993376	33.76
	AT5G46820		Protein of Unknown Function (DUF239) [Source:TAIR;Acc:AT5G46820]	18993633	18996672	32.4
	AT5G46825			19002045	19002499	37.58

AT5G46830	BHLH28	Transcription factor bHLH28 [Source:UniProtKB/Swiss-Prot;Acc:Q9LUK7]	19002564	19004384	43.99
AT5G46840		At5g46840 [Source:UniProtKB/TrEMBL;Acc:Q9LUK6]	19005547	19007770	39.16
AT5G46845	MIR160C	MIR160/MIR160C (MICRORNA160); miRNA [Source:TAIR;Acc:AT5G46845]	19009094	19009182	51.69
AT5G46850		unknown protein; Ha. [Source:TAIR;Acc:AT5G46850]	19010608	19012038	40.74
AT5G46860	SYP22	VAM3 [Source:UniProtKB/TrEMBL;Acc:A0A178UDT6]	19012021	19014110	36.75
AT5G46870		At5g46870 [Source:UniProtKB/TrEMBL;Acc:Q6NM69]	19015233	19017247	36.38
AT5G46871		Defensin-like protein 308 [Source:UniProtKB/Swiss-Prot;Acc:Q4VP04]	19017942	19018710	31.73
AT5G46873		Putative defensin-like protein 303 [Source:UniProtKB/Swiss-Prot;Acc:Q2V311]	19021183	19021594	31.8
AT5G46874		Putative defensin-like protein 309 [Source:UniProtKB/Swiss-Prot;Acc:Q2V310]	19022383	19022699	34.38
AT5G46877		Putative defensin-like protein 304 [Source:UniProtKB/Swiss-Prot;Acc:Q2V309]	19025860	19026294	32.18
AT5G46880	HDG5	Homeobox-leucine zipper protein HDG5 [Source:UniProtKB/Swiss-Prot;Acc:Q9FJS2]	19030844	19035959	36.81
AT5G46890		Bifunctional inhibitor/lipid-transfer protein/seed storage 2S albumin superfamily protein [Source:UniProtKB/TrEMBL;Acc:Q9FJS1]	19036296	19036938	38.41
AT5G46900		Bifunctional inhibitor/lipid-transfer protein/seed storage 2S albumin superfamily protein [Source:UniProtKB/TrEMBL;Acc:Q39100]	19039701	19040456	36.9
AT5G46910		Transcription factor jumonji (Jmj) family protein / zinc finger (C5HC2 type) family protein [Source:UniProtKB/TrEMBL;Acc:F4KIX0]	19046339	19051379	38.15
AT5G06885			19046652	19046919	45.52
AT5G46915		Putative B3 domain-containing protein At5g46915 [Source:UniProtKB/Swiss-Prot;Acc:F4KIX1]	19051417	19053200	38.34
AT5G46920	NMAT2	Nuclear intron maturase 2, mitochondrial [Source:UniProtKB/Swiss-Prot;Acc:Q9FJR9]	19053472	19056073	40.16
AT5G46930		Plant invertase/pectin methylesterase inhibitor superfamily protein [Source:UniProtKB/TrEMBL;Acc:Q9FJR8]	19056505	19057243	37.08
AT5G46940		Plant invertase/pectin methylesterase inhibitor superfamily protein [Source:UniProtKB/TrEMBL;Acc:Q9FJR7]	19058031	19058814	38.27
AT5G46950		At5g46950 [Source:UniProtKB/TrEMBL;Acc:Q9FJR6]	19059472	19060121	37.54
AT5G46960	PMEI12	Pectinesterase inhibitor 12 [Source:UniProtKB/Swiss-Prot;Acc:Q9FJR5]	19062146	19062814	37.37
AT5G46970		Plant invertase/pectin methylesterase inhibitor superfamily protein [Source:UniProtKB/TrEMBL;Acc:Q9FJR4]	19063755	19064418	35.39
AT5G06915			19065143	19065345	33.5
AT5G46980		Plant invertase/pectin methylesterase inhibitor superfamily protein [Source:UniProtKB/TrEMBL;Acc:Q9FJR3]	19065311	19066007	34.86
AT5G46990		Plant invertase/pectin methylesterase inhibitor superfamily protein [Source:UniProtKB/TrEMBL;Acc:Q9FJR2]	19067455	19068292	36.28
AT5G47000	PER65	Peroxidase 65 [Source:UniProtKB/Swiss-Prot;Acc:Q9FJR1]	19068803	19070333	41.28
AT5G47010	UPF1	Regulator of nonsense transcripts 1 homolog [Source:UniProtKB/Swiss-Prot;Acc:Q9FJR0]	19071824	19079453	40.21
AT5G47020		unknown protein; FUNCTIONS IN: molecular_function unknown; INVOLVED IN: biological_process unknown; LOCATED IN: endomembrane	19081458	19090104	39.48
AT5G47030		ATP synthase subunit delta', mitochondrial [Source:UniProtKB/Swiss-Prot;Acc:Q96252]	19090150	19092416	38.6
AT5G47040	LON2	Lon protease homolog 2, peroxisomal [Source:UniProtKB/Swiss-Prot;Acc:O64948]	19092858	19098772	39.93
AT5G06925			19100802	19101205	35.64
AT5G47050		SBP (S-ribonuclease binding protein) family protein [Source:UniProtKB/TrEMBL;Acc:Q9FJQ8]	19106297	19108348	36.79
AT5G06935			19115322	19115651	37.88
AT5G06945			19115358	19115909	38.04
AT5G47060		Putative uncharacterized protein [Source:UniProtKB/TrEMBL;Acc:Q9FGQ9]	19116719	19118744	30.45
AT5G47070	PBL19	Probable serine/threonine-protein kinase PBL19 [Source:UniProtKB/Swiss-Prot;Acc:Q9LTC0]	19118465	19121103	35.81
AT5G47075	LCR20	Defensin-like protein 127 [Source:UniProtKB/Swiss-Prot;Acc:P82734]	19122503	19122866	31.87
AT5G47077	LCR6	Putative defensin-like protein 126 [Source:UniProtKB/Swiss-Prot;Acc:P82721]	19123506	19123779	35.04
AT5G47080	CKB1	Casein kinase II subunit beta-1 [Source:UniProtKB/Swiss-Prot;Acc:P40228]	19124563	19126694	37.05
AT5G47090		AT5g47090/K14A3_4 [Source:UniProtKB/TrEMBL;Acc:Q9LTB9]	19127187	19129605	38.94
AT5G47100	CBL9	Calcineurin B-like protein 9 [Source:UniProtKB/Swiss-Prot;Acc:Q9LTB8]	19129640	19132304	35.76
AT5G47110	LIL3.2	Light-harvesting complex-like protein 3 isotype 2, chloroplastic [Source:UniProtKB/Swiss-Prot;Acc:Q6NKS4]	19133986	19135414	38.91
AT5G47120	BI-1	Bax inhibitor 1 [Source:UniProtKB/Swiss-Prot;Acc:Q9LD45]	19135952	19138025	37.8
AT5G47130		Bax inhibitor-1 family protein [Source:TAIR;Acc:AT5G47130]	19138840	19142284	32.31
AT5G06955			19140798	19140996	34.17
AT5G47140	GATA27	GATA transcription factor 27 [Source:UniProtKB/Swiss-Prot;Acc:Q5PP38]	19144601	19147657	35.92
AT5G47150		YDG domain-containing protein At5g47150 [Source:UniProtKB/Swiss-Prot;Acc:Q9FHI0]	19149938	19151913	38.66
AT5G47160		YDG domain-containing protein At5g47160 [Source:UniProtKB/Swiss-Prot;Acc:Q9LVU3]	19155976	19158249	37.25
AT5G47170		Gb [Source:UniProtKB/TrEMBL;Acc:Q9LVU2]	19158351	19159091	39.81
AT5G47175	LCR3	Defensin-like protein 141 [Source:UniProtKB/Swiss-Prot;Acc:P82718]	19160013	19160456	29.05
AT5G47180	PVA21	Vesicle-associated protein 2-1 [Source:UniProtKB/Swiss-Prot;Acc:Q9LVU1]	19161009	19163980	37.25
AT5G47190		50S ribosomal protein L19-2, chloroplastic [Source:UniProtKB/Swiss-Prot;Acc:Q8RXX5]	19164184	19166135	36.99
AT5G47200	RABD2B	Ras-related protein RABD2b [Source:UniProtKB/Swiss-Prot;Acc:Q9FPJ4]	19166859	19169093	36.87
AT5G47210	RGGC	RGG repeats nuclear RNA binding protein C [Source:UniProtKB/Swiss-Prot;Acc:Q9LVT8]	19168941	19171263	41.41
AT5G47220	ERF2	ERF2 [Source:UniProtKB/TrEMBL;Acc:A0A178UCP3]	19171643	19172956	40.87
AT5G47229		unknown protein; Ha. [Source:TAIR;Acc:AT5G47229]	19179694	19179849	39.1
AT5G47230	ERF5	ERF5 [Source:UniProtKB/TrEMBL;Acc:A0A178UKK9]	19179881	19181182	40.32
AT5G06965			19182632	19182954	38.08
AT5G47240	atnudt8	nudix hydrolase homolog 8 [Source:TAIR;Acc:AT5G47240]	19183523	19185644	34.92
AT5G47250		Probable disease resistance protein At5g47250 [Source:UniProtKB/Swiss-Prot;Acc:Q9LVT4]	19185698	19188885	40.18
AT5G47260		Probable disease resistance protein At5g47260 [Source:UniProtKB/Swiss-Prot;Acc:Q9LVT3]	19189411	19192516	40.86
AT5G47280	ADR1-L3	Putative disease resistance protein At5g47280 [Source:UniProtKB/Swiss-Prot;Acc:Q9LVT1]	19193157	19195559	41.66
AT5G47290		Myb family transcription factor [Source:UniProtKB/TrEMBL;Acc:Q9LVT0]	19197040	19197431	45.41
AT5G47300		Probable F-box protein At5g47300 [Source:UniProtKB/Swiss-Prot;Acc:Q9LVS9]	19198125	19199375	39.49
AT5G47310		At5g47310 [Source:UniProtKB/TrEMBL;Acc:Q9LVS8]	19200731	19202972	37.47
AT5G47320	RPS19	40S ribosomal protein S19, mitochondrial [Source:UniProtKB/Swiss-Prot;Acc:P39697]	19203052	19205285	38.27
AT5G47330		Alpha/beta-Hydrolases superfamily protein [Source:UniProtKB/TrEMBL;Acc:Q9LVS6]	19206829	19208998	36.31
AT5G47340		Alpha/beta-Hydrolases superfamily protein [Source:UniProtKB/TrEMBL;Acc:F4JX42]	19209507	19211134	36.55
AT5G06975			19212044	19212249	41.26
AT5G47350		Alpha/beta-Hydrolases superfamily protein [Source:UniProtKB/TrEMBL;Acc:Q9LVS4]	19212665	19214453	37.73
AT5G47360		Pentatricopeptide repeat-containing protein At5g47360 [Source:UniProtKB/Swiss-Prot;Acc:Q9LVS3]	19214445	19215878	41.14
AT5G47370	HAT2	Homeobox-leucine zipper protein HAT2 [Source:UniProtKB/Swiss-Prot;Acc:P46601]	19216271	19218007	39.15
AT5G06985			19219391	19219647	43.58
AT5G06995			19219758	19220028	38.01
AT5G47380		Protein of unknown function, DUF547 [Source:TAIR;Acc:AT5G47380]	19221274	19224652	37.7
AT5G07005			19225524	19225825	29.8
AT5G47390	KUA1	Transcription factor KUA1 [Source:UniProtKB/Swiss-Prot;Acc:Q9LVS0]	19226769	19228895	39.92
AT5G47400		unknown protein; FUNCTIONS IN: molecular_function unknown; LOCATED IN: endomembrane system; EXPRESSED IN: 23 plant structures;	19229162	19232743	41.32
AT5G47420		Tryptophan RNA-binding attenuator protein-like protein [Source:UniProtKB/TrEMBL;Acc:Q8RXM5]	19232784	19235066	39.68
AT5G47430		DWNN domain, a CCHC-type zinc finger [Source:TAIR;Acc:AT5G47430]	19235555	19241263	40.29
AT5G47435	PURU1	Formyltetrahydrofolate deformylase 1, mitochondrial [Source:UniProtKB/Swiss-Prot;Acc:Q93YQ3]	19241672	19243988	39.06
AT5G47440		At5g47440 [Source:UniProtKB/TrEMBL;Acc:Q9FGL4]	19243531	19245648	41.97
AT5G47450	TIP2-3	Aquaporin TIP2-3 [Source:UniProtKB/Swiss-Prot;Acc:Q9FGL2]	19248249	19249622	42.29
AT5G47455		unknown protein; BEST Arabidopsis thaliana protein match is: unknown protein (TAIR:AT4G17310.2); Ha. [Source:TAIR;Acc:AT5G47455]	19250659	19252491	37.64
AT5G47460	PCMP-E103	Putative pentatricopeptide repeat-containing protein At5g47460 [Source:UniProtKB/Swiss-Prot;Acc:Q9FGL1]	19252463	19254336	43.38
AT5G47470		Nodulin MtN21 /EamA-like transporter family protein [Source:TAIR;Acc:AT5G47470]	19254403	19256810	36
AT5G47480	MAG5	Protein transport protein SEC16A homolog [Source:UniProtKB/Swiss-Prot;Acc:Q9FGK9]	19257027	19263425	41.85
AT5G47490	SEC16B	Protein transport protein SEC16B homolog [Source:UniProtKB/Swiss-Prot;Acc:Q9FGK8]	19263826	19270899	41.16
AT5G47500	PME68	Probable pectinesterase 68 [Source:UniProtKB/Swiss-Prot;Acc:Q8LPF3]	19270944	19272892	39.97
AT5G47510		Sec14p-like phosphatidylinositol transfer family protein [Source:TAIR;Acc:AT5G47510]	19274853	19277383	37.5
AT5G47520	RABA5A	Ras-related protein RABA5a [Source:UniProtKB/Swiss-Prot;Acc:Q9FGK5]	19277230	19279747	38.68
AT5G47530		Cytochrome b561 and DOMON domain-containing protein [Source:UniProtKB/TrEMBL;Acc:A0A178USN6]	19281374	19283061	42.65
AT5G47540		At5g47540 [Source:UniProtKB/TrEMBL;Acc:B3LFB8]	19283029	19285758	39.45
AT5G47550	CYS5	Cysteine proteinase inhibitor 5 [Source:UniProtKB/Swiss-Prot;Acc:Q41916]	19286314	19287179	39.95
AT5G47560	TDT	Tonoplast dicarboxylate transporter [Source:UniProtKB/Swiss-Prot;Acc:Q8LG88]	19287369	19291103	39.38
AT5G47570		NADH dehydrogenase [ubiquinone] 1 beta subcomplex subunit 8, mitochondrial [Source:UniProtKB/Swiss-Prot;Acc:Q9FGK0]	19292587	19294800	39.43
AT5G47580		AT5g47580/MNJ7_17 [Source:UniProtKB/TrEMBL;Acc:Q9FGJ9]	19294813	19297424	43.99
AT5G47590		Heat shock protein HSP20/alpha crystallin family [Source:UniProtKB/TrEMBL;Acc:Q9FGJ8]	19297614	19299174	42.09
AT5G47600	HSP14.7	14.7 kDa heat shock protein [Source:UniProtKB/Swiss-Prot;Acc:Q6NLV0]	19299717	19300503	41.04
AT5G47610	ATL79	At5g47610 [Source:UniProtKB/TrEMBL;Acc:Q1H5E6]	19301130	19302117	41.9
AT5G47620		RNA-binding (RRM/RBD/RNP motifs) family protein [Source:UniProtKB/TrEMBL;Acc:F4JYK6]	19302190	19304950	39.51
AT5G47630	MTACP2	Acyl carrier protein [Source:UniProtKB/TrEMBL;Acc:A0A178UB54]	19306139	19307094	38.28
AT5G47635		At5g47635 [Source:UniProtKB/TrEMBL;Acc:Q29PT1]	19307048	19308009	42.31
AT5G07015			19309153	19309593	44.67
AT5G47640	NFYB2	Nuclear transcription factor Y subunit B-2 [Source:UniProtKB/Swiss-Prot;Acc:Q9FGJ3]	19309227	19310272	44.36

AT5G47650	ATNUDT2	nudix hydrolase homolog 2 [Source:TAIR;Acc:AT5G47650]	19310192	19312747	39.4
AT5G47660		Homeodomain-like superfamily protein [Source:UniProtKB/TrEMBL;Acc:Q9FGJ1]	19312747	19314893	43.22
AT5G47670	NFYB6	Nuclear transcription factor Y subunit B-6 [Source:UniProtKB/Swiss-Prot;Acc:Q84W66]	19314778	19316169	44.54
AT5G47680	TRM10	At5g47680 [Source:UniProtKB/TrEMBL;Acc:Q9FGI9]	19316046	19317539	41.37
AT5G47690		Binding protein [Source:UniProtKB/TrEMBL;Acc:B3H5K3]	19317668	19327431	37.66
AT5G47700	RPP1C	60S acidic ribosomal protein P1-3 [Source:UniProtKB/Swiss-Prot;Acc:Q8LEQ0]	19327566	19328986	38.49
AT5G47710	CAR11	Protein C2-DOMAIN ABA-RELATED 11 [Source:UniProtKB/Swiss-Prot;Acc:Q9FIK8]	19329544	19331477	38.62
AT5G47720		AACT1 [Source:UniProtKB/TrEMBL;Acc:A0A384KT84]	19331547	19334427	42.14
AT5G47730		At5g47730 [Source:UniProtKB/TrEMBL;Acc:Q9FIK6]	19334250	19337148	38.08
AT5G07025			19336704	19337115	37.14
AT5G47740		Adenine nucleotide alpha hydrolases-like superfamily protein [Source:UniProtKB/TrEMBL;Acc:F4JYN2]	19338511	19339775	42.45
AT5G47750	D6PKL2	Serine/threonine-protein kinase D6PKL2 [Source:UniProtKB/Swiss-Prot;Acc:Q39183]	19339703	19342410	45.46
AT5G47760	PGLP2	Phosphoglycolate phosphatase 2 [Source:UniProtKB/Swiss-Prot;Acc:Q8GWU0]	19342405	19345127	39.66
AT5G47770	FPS1	Farnesyl pyrophosphate synthase 1, mitochondrial [Source:UniProtKB/Swiss-Prot;Acc:Q09152]	19345110	19347677	39.56
AT5G47780	GAUT4	Hexosyltransferase (Fragment) [Source:UniProtKB/TrEMBL;Acc:W8PUB8]	19347874	19350976	41.35
AT5G47790		Protein phosphatase 1 regulatory inhibitor subunit PPP1R8 homolog [Source:UniProtKB/Swiss-Prot;Acc:Q9FIK2]	19351292	19353495	43.15
AT5G47800		Phototropic-responsive NPH3 family protein [Source:TAIR;Acc:AT5G47800]	19353532	19356448	41.65
AT5G47810	PFK2	ATP-dependent 6-phosphofructokinase 2 [Source:UniProtKB/Swiss-Prot;Acc:Q9FIK0]	19356292	19358125	48.15
AT5G47820	KIN4A	Kinesin-like protein KIN-4A [Source:UniProtKB/Swiss-Prot;Acc:Q8GS71]	19366133	19372815	38.19
AT5G47830		unknown protein; Ha. [Source:TAIR;Acc:AT5G47830]	19372821	19374693	36.57
AT5G47840	AMK2	Adenylate kinase 2, chloroplastic [Source:UniProtKB/Swiss-Prot;Acc:Q9FIJ7]	19375317	19378465	37.82
AT5G47850	CCR4	Serine/threonine-protein kinase-like protein CCR4 [Source:UniProtKB/Swiss-Prot;Acc:Q9FIJ6]	19378669	19381190	45.16
AT5G47860		AT5g47860/MCA23_20 [Source:UniProtKB/TrEMBL;Acc:Q9FIJ5]	19381519	19384422	38.74
AT5G47870	RAD52-2	DNA repair RAD52-like protein 2, chloroplastic [Source:UniProtKB/Swiss-Prot;Acc:Q9FIJ4]	19384368	19385869	37.88
AT5G47880	ERF1-1	ERF1-1 [Source:UniProtKB/TrEMBL;Acc:A0A178UFY7]	19386045	19388587	38.3
AT5G47890		NADH dehydrogenase [ubiquinone] 1 alpha subcomplex subunit 2 [Source:UniProtKB/Swiss-Prot;Acc:Q9FIJ2]	19388704	19390937	35.05
AT5G47900		Protein of unknown function (DUF1624) [Source:TAIR;Acc:AT5G47900]	19392166	19395287	35.75
AT5G47910	RBOHD	Respiratory burst oxidase homolog protein D [Source:UniProtKB/Swiss-Prot;Acc:Q9FIJ0]	19397041	19402061	39.41
AT5G47920		Emb [Source:UniProtKB/TrEMBL;Acc:Q9FII9]	19403171	19404147	40.12
AT5G47930	RPS27D	40S ribosomal protein S27-3 [Source:UniProtKB/Swiss-Prot;Acc:Q8L953]	19406080	19407449	34.74
AT5G47940		40S ribosomal protein S27 [Source:UniProtKB/TrEMBL;Acc:Q9FGV5]	19409242	19412077	38.93
AT5G47950		Acetyl-CoA:benzylalcohol acetyltranferase-like protein [Source:UniProtKB/TrEMBL;Acc:Q9FGV4]	19417020	19418657	42.31
AT5G47960	RABA4C	SMG1 [Source:UniProtKB/TrEMBL;Acc:A0A178UQI5]	19421340	19422533	37.02
AT5G47970		tRNA-dihydrouridine synthase [Source:UniProtKB/TrEMBL;Acc:Q9FI41]	19422203	19425872	37.71
AT5G47980	BAHD1	BAHD acyltransferase At5g47980 [Source:UniProtKB/Swiss-Prot;Acc:Q9FI40]	19428888	19430517	43.87
AT5G07035			19431998	19432892	36.98
AT5G47990	CYP705A5	THAD1 [Source:UniProtKB/TrEMBL;Acc:A0A178UGC2]	19434758	19436749	41.62
AT5G48000	CYP708A2	Cytochrome P450, family 708, subfamily A, polypeptide 2 [Source:UniProtKB/TrEMBL;Acc:F4K051]	19444103	19447866	33.69
AT5G48010	THAS1	Thalianol synthase [Source:UniProtKB/Swiss-Prot;Acc:Q9FI37]	19456837	19461706	34.17
AT5G48020		2-oxoglutarate (2OG) and Fe(II)-dependent oxygenase superfamily protein [Source:UniProtKB/TrEMBL;Acc:Q9FI36]	19462516	19464971	39.13
AT5G48030	GFA2	GFA2 [Source:UniProtKB/TrEMBL;Acc:A0A178UJR3]	19465788	19469939	36.68
AT5G48040		Emb [Source:UniProtKB/TrEMBL;Acc:Q9FI34]	19469992	19472445	39.61
AT5G48050		Copia-like polyprotein/retrotransposon [Source:UniProtKB/TrEMBL;Acc:Q9FI33]	19472446	19473838	41.78
AT5G48060		C2 calcium/lipid-binding plant phosphoribosyltransferase family protein [Source:UniProtKB/TrEMBL;Acc:Q9FI32]	19475296	19479066	41.42
AT5G48070	XTH20	Xyloglucan endotransglucosylase/hydrolase protein 20 [Source:UniProtKB/Swiss-Prot;Acc:Q9FI31]	19481897	19483527	35.32
AT5G48090	ELP1	EDM2-like protein1 [Source:UniProtKB/TrEMBL;Acc:F4K064]	19484015	19488657	37.05
AT5G48100	TT10	Laccase-15 [Source:UniProtKB/Swiss-Prot;Acc:Q84J37]	19489327	19492744	34.06
AT5G07055			19499055	19499533	27.77
AT5G48110		Terpenoid cyclases/Protein prenyltransferases superfamily protein [Source:TAIR;Acc:AT5G48110]	19500988	19504534	34.34
AT5G48120	MMS19	MMS19 nucleotide excision repair protein homolog [Source:UniProtKB/Swiss-Prot;Acc:Q0WVF8]	19507397	19514895	37
AT5G48130		BTB/POZ domain-containing protein At5g48130 [Source:UniProtKB/Swiss-Prot;Acc:Q9LUB9]	19516102	19518513	39.3
AT5G48140		At5g48140 [Source:UniProtKB/TrEMBL;Acc:Q9LUB8]	19518666	19520528	39.35
AT5G48150	PAT1	Scarecrow-like transcription factor PAT1 [Source:UniProtKB/Swiss-Prot;Acc:Q9LDL7]	19522238	19524788	40.18
AT5G48160	OBE2	Protein OBERON 2 [Source:UniProtKB/Swiss-Prot;Acc:Q9LUB7]	19527767	19530430	40.65
AT5G48170	SNE	SNE [Source:UniProtKB/TrEMBL;Acc:A0A178UIU3]	19532270	19533390	40.68
AT5G48175		At5g48175 [Source:UniProtKB/TrEMBL;Acc:Q8L9E4]	19539145	19540623	32.52
AT5G48180	NSP5	Nitrile-specifier protein 5 [Source:UniProtKB/Swiss-Prot;Acc:Q93XW5]	19540937	19542760	39.53
AT5G48190		Gb [Source:UniProtKB/TrEMBL;Acc:Q9LDS4]	19542778	19544429	34.44
AT5G48200		Putative uncharacterized protein [Source:UniProtKB/TrEMBL;Acc:Q9LUB4]	19545255	19547270	34.08
AT5G48205		Zinc ion binding protein [Source:UniProtKB/TrEMBL;Acc:F4K080]	19546007	19547787	37.06
AT5G48210		Prolamin-like protein (DUF1278) [Source:UniProtKB/TrEMBL;Acc:Q9LUB3]	19548414	19549141	36.13
AT5G48220		AT5g48220/MIF21_11 [Source:UniProtKB/TrEMBL;Acc:Q9LUB2]	19549676	19552267	39.24
AT5G48230	AAT1	AT5G48230 protein [Source:UniProtKB/TrEMBL;Acc:B9DGQ1]	19552142	19555531	38.11
AT5G48240		unknown protein; FUNCTIONS IN: molecular_function unknown; INVOLVED IN: biological_process unknown; LOCATED IN: nucleolus; EXP	19559201	19561374	36.66
AT5G48250	COL10	Zinc finger protein CONSTANS-LIKE 10 [Source:UniProtKB/Swiss-Prot;Acc:Q9LUA9]	19561319	19563832	38.58
AT5G48270		DUF868 family protein (DUF868) [Source:UniProtKB/TrEMBL;Acc:Q9LUA7]	19564422	19565968	38.33
AT5G07065			19566960	19567084	40.8
AT5G48280		Transmembrane protein [Source:UniProtKB/TrEMBL;Acc:Q9FGW2]	19567064	19567688	31.2
AT5G07075			19567208	19567386	33.52
AT5G48290		Heavy metal transport/detoxification superfamily protein [Source:TAIR;Acc:AT5G48290]	19568760	19569866	39.3
AT5G48300	APS1	Glucose-1-phosphate adenylyltransferase small subunit, chloroplastic [Source:UniProtKB/Swiss-Prot;Acc:P55228]	19570238	19572804	40.79
AT5G48310		Portal protein [Source:UniProtKB/TrEMBL;Acc:Q9LK83]	19574780	19580469	37.64
AT5G48320		Cysteine/Histidine-rich C1 domain family protein [Source:UniProtKB/TrEMBL;Acc:Q9LK82]	19581697	19584772	41.58
AT5G48330		Putative regulator of chromosome condensation (Cell cycle regulatory protein) [Source:UniProtKB/TrEMBL;Acc:Q8GYL6]	19585924	19587857	42.92
AT5G48335		At5g48335 [Source:UniProtKB/TrEMBL;Acc:Q6AWV7]	19587872	19589606	37.98
AT5G48340		Uncharacterized protein At5g48340 [Source:UniProtKB/TrEMBL;Acc:Q9LK80]	19590309	19593075	38.56
AT5G48350		Emb [Source:UniProtKB/TrEMBL;Acc:Q9LK79]	19593217	19594441	38.37
AT5G48360	FH9	Formin-like protein 9 [Source:UniProtKB/Swiss-Prot;Acc:Q8GX37]	19595536	19598492	41.87
AT5G48370		At5g48370 [Source:UniProtKB/TrEMBL;Acc:Q9LK77]	19598486	19600708	40.13
AT5G48375	TGG3	Putative myrosinase 3 [Source:UniProtKB/Swiss-Prot;Acc:Q3E8E5]	19601303	19603883	37.12
AT5G48380	BIR1	Probably inactive leucine-rich repeat receptor-like protein kinase At5g48380 [Source:UniProtKB/Swiss-Prot;Acc:Q9ASS4]	19604089	19607147	39.65
AT5G48385	FRL3	FRIGIDA-like protein 3 [Source:UniProtKB/Swiss-Prot;Acc:Q67ZB3]	19608490	19612007	40.11
AT5G48390	ZIP4	TPR repeat-containing protein ZIP4 [Source:UniProtKB/Swiss-Prot;Acc:B0M1H3]	19612020	19615383	44.02
AT5G48400	GLR1.2	Glutamate receptor 1.2 [Source:UniProtKB/Swiss-Prot;Acc:Q9LV72]	19616066	19619508	40.4
AT5G48410	GLR1.3	Glutamate receptor 1.3 [Source:UniProtKB/Swiss-Prot;Acc:Q9FH75]	19620267	19623425	39.51
AT5G48412			19623516	19624232	39.75
AT5G07095			19624548	19624687	37.14
AT5G07105			19624720	19624869	39.33
AT5G07115			19625247	19625636	38.72
AT5G07125			19625384	19625721	39.64
AT5G48420		Putative uncharacterized protein [Source:UniProtKB/TrEMBL;Acc:Q9LV71]	19626362	19626790	43.36
AT5G48430		At5g48430 [Source:UniProtKB/TrEMBL;Acc:Q9LV70]	19627634	19629167	41.92
AT5G48440		At5g48440 [Source:UniProtKB/TrEMBL;Acc:Q9LV69]	19629658	19632775	39.38
AT5G07145			19632695	19633228	39.33
AT5G48450	sks3	SKU5 similar 3 [Source:UniProtKB/TrEMBL;Acc:F4K1P9]	19632703	19635679	39.64
AT5G48460	FIM2	Fimbrin-2 [Source:UniProtKB/Swiss-Prot;Acc:O50064]	19636045	19639529	35.93
AT5G48465		pre-tRNA [Source:TAIR;Acc:AT5G48465]	19641603	19641674	59.72
AT5G48470	PRDA1	Protein PEP-RELATED DEVELOPMENT ARRESTED 1, chloroplastic [Source:UniProtKB/Swiss-Prot;Acc:Q1H5E9]	19641744	19644362	37.46
AT5G48480		Uncharacterized protein At5g48480 [Source:UniProtKB/Swiss-Prot;Acc:Q9LV66]	19644539	19646840	39.36
AT5G48485	DIR1	Putative lipid-transfer protein DIR1 [Source:UniProtKB/Swiss-Prot;Acc:Q8W453]	19646028	19646840	41.33
AT5G48490		At5g48490 [Source:UniProtKB/TrEMBL;Acc:Q9LV65]	19647784	19648362	38
AT5G48500		Pathogenic type III effector avirulence factor Avr AvrRpt-cleavage: cleavage site protein [Source:UniProtKB/TrEMBL;Acc:Q8LEL0]	19652980	19654355	38.88
AT5G48510		BTB/POZ domain-containing protein At5g48510 [Source:UniProtKB/Swiss-Prot;Acc:Q9LV63]	19658080	19658938	38.3
AT5G07155			19659330	19659608	37.28
AT5G48515		Putative defensin-like protein 221 [Source:UniProtKB/Swiss-Prot;Acc:Q2V306]	19659857	19660201	27.25

AT5G48520		unknown protein; Ha. [Source:TAIR;Acc:AT5G48520]	19661203	19666436	37.43
AT5G48530		unknown protein; FUNCTIONS IN: molecular_function unknown; INVOLVED IN: biological_process unknown; LOCATED IN: mitochondrion;	19666790	19668830	38.9
AT5G48540	CRRSP55	Cysteine-rich repeat secretory protein 55 [Source:UniProtKB/Swiss-Prot;Acc:Q9LV60]	19668964	19669920	40.23
AT5G07185			19673375	19673763	33.93
AT5G48543	LCR1	Defensin-like protein 147 [Source:UniProtKB/Swiss-Prot;Acc:P82716]	19674358	19675465	27.62
AT5G48545	HINT3	HINT3 [Source:UniProtKB/TrEMBL;Acc:A0A178UAY8]	19675452	19678194	35.44
AT5G48550		F-box associated ubiquitination effector family protein [Source:UniProtKB/TrEMBL;Acc:F4K1R3]	19678825	19680560	41.07
AT5G48560	BHLH78	Transcription factor bHLH78 [Source:UniProtKB/Swiss-Prot;Acc:Q9FJL4]	19683809	19687187	34.24
AT5G48570	FKBP65	Peptidyl-prolyl cis-trans isomerase FKBP65 [Source:UniProtKB/Swiss-Prot;Acc:Q9FJL3]	19690461	19693845	38.82
AT5G48575		Mediator of RNA polymerase II transcription subunit-like protein, putative (DUF1216) [Source:UniProtKB/TrEMBL;Acc:Q3E8E3]	19694150	19695065	38.76
AT5G48580	FKBP15-2	Peptidylprolyl isomerase [Source:UniProtKB/TrEMBL;Acc:A0A178UPA7]	19695767	19698712	34.69
AT5G48590		Gb [Source:UniProtKB/TrEMBL;Acc:Q9FJL1]	19698280	19699992	39.87
AT5G48595		Defensin-like protein 219 [Source:UniProtKB/Swiss-Prot;Acc:Q2V305]	19700640	19701022	26.63
AT5G48600	ATSMC3	Structural maintenance of chromosomes protein [Source:UniProtKB/TrEMBL;Acc:F4K1S1]	19701728	19709342	37.52
AT5G48605		Defensin-like protein 220 [Source:UniProtKB/Swiss-Prot;Acc:Q2V304]	19710163	19710599	26.09
AT5G48610		unknown protein; Ha. [Source:TAIR;Acc:AT5G48610]	19711541	19716059	38.42
AT5G48620	RPP8L4	Probable disease resistance RPP8-like protein 4 [Source:UniProtKB/Swiss-Prot;Acc:Q9FJK8]	19716104	19721477	36.75
AT5G48630		Cyclin family protein [Source:UniProtKB/TrEMBL;Acc:F4K359]	19721465	19723307	36.63
AT5G48640	CYCC1-1	Cyclin-C1-1 [Source:UniProtKB/Swiss-Prot;Acc:Q9FJK6]	19723388	19725748	35.45
AT5G48650		Nuclear transport factor 2 (NTF2) family protein with RNA binding (RRM-RBD-RNP motifs) domain-containing protein [Source:UniProtKB/Tr	19726672	19729764	38.64
AT5G48655		Putative RING zinc finger [Source:UniProtKB/TrEMBL;Acc:Q8GS41]	19730380	19733156	38.28
AT5G48657		defense protein-related [Source:TAIR;Acc:AT5G48657]	19733882	19735726	35.61
AT5G48660		B-cell receptor-associated protein 31-like protein [Source:UniProtKB/TrEMBL;Acc:Q9FJK4]	19736540	19738538	35.87
AT5G48670	AGL80	Agamous-like MADS-box protein AGL80 [Source:UniProtKB/Swiss-Prot;Acc:Q9FJK3]	19738646	19739841	37.21
AT5G48675		pre-tRNA [Source:TAIR;Acc:AT5G48675]	19744175	19744246	62.5
AT5G48680		At5g48680 [Source:UniProtKB/TrEMBL;Acc:Q52K85]	19744404	19746192	37.28
AT5G48690		Ubiquitin-associated (UBA)/TS-N domain protein [Source:UniProtKB/TrEMBL;Acc:Q5XV09]	19747026	19749061	36.98
AT5G48700	SUMO6	Putative small ubiquitin-related modifier 6 [Source:UniProtKB/Swiss-Prot;Acc:Q9FKC6]	19749494	19750013	36.35
AT5G07195			19753327	19753620	32.99
AT5G48710	SUMO4	Putative small ubiquitin-related modifier 4 [Source:UniProtKB/Swiss-Prot;Acc:Q9FKC5]	19755065	19756051	40.32
AT5G07205			19755915	19756320	39.41
AT5G48720	XRI1	XRI1 [Source:UniProtKB/TrEMBL;Acc:A0A178UAQ4]	19758881	19762015	37.58
AT5G07255			19762359	19762647	28.03
AT5G48730		Pentatricopeptide repeat-containing protein At5g48730, chloroplastic [Source:UniProtKB/Swiss-Prot;Acc:Q9FKC3]	19763110	19765272	39.39
AT5G07265			19764422	19764740	42.95
AT5G48740		Probable LRR receptor-like serine/threonine-protein kinase At5g48740 [Source:UniProtKB/Swiss-Prot;Acc:C0LGV0]	19765195	19769369	39.31
AT5G48750		Cytochrome b561 and DOMON domain-containing protein At5g48750 [Source:UniProtKB/Swiss-Prot;Acc:Q9FKC1]	19770052	19770819	43.88
AT5G48760	RPL13AD	60S ribosomal protein L13a-4 [Source:UniProtKB/Swiss-Prot;Acc:Q9FKC0]	19771099	19772789	39.86
AT5G48775		other RNA [Source:TAIR;Acc:AT5G48775]	19772844	19777231	41.57
AT5G48770		Disease resistance protein (TIR-NBS-LRR class) family [Source:UniProtKB/TrEMBL;Acc:Q9FKB9]	19773117	19777242	41.54
AT5G48780		Disease resistance protein (TIR-NBS class) [Source:UniProtKB/TrEMBL;Acc:F4K383]	19777453	19779815	40.96
AT5G48790		AT5g48790/K24G6_12 [Source:UniProtKB/TrEMBL;Acc:Q94F50]	19779860	19782066	35.66
AT5G48800		BTB/POZ domain-containing protein At5g48800 [Source:UniProtKB/Swiss-Prot;Acc:Q9FKB6]	19786465	19789129	41.05
AT5G07275			19787994	19788229	46.19
AT5G48810	CYTB5-D	Cytochrome B5 isoform D [Source:UniProtKB/Swiss-Prot;Acc:Q9ZWT2]	19789067	19790372	38.9
AT5G07285			19789614	19790115	35.06
AT5G48820	KRP3	Inhibitor/interactor with cyclin-dependent kinase [Source:UniProtKB/TrEMBL;Acc:F4K388]	19792270	19794480	37.09
AT5G48830		unknown protein; FUNCTIONS IN: molecular_function unknown; INVOLVED IN: biological_process unknown; LOCATED IN: chloroplast; EX	19799953	19803098	38.43
AT5G48835		pre-tRNA [Source:TAIR;Acc:AT5G48835]	19803135	19803220	58.14
AT5G48840	PANC	Pantoate--beta-alanine ligase [Source:UniProtKB/Swiss-Prot;Acc:Q9FKB3]	19803502	19805356	40.32
AT5G48850	SDI1	ATSDI1 [Source:UniProtKB/TrEMBL;Acc:A0A178UKA2]	19805437	19807853	34.55
AT5G48860		Putative uncharacterized protein [Source:UniProtKB/TrEMBL;Acc:Q9FKB1]	19811651	19812865	37.28
AT5G48870	LSM5	SAD1 [Source:UniProtKB/TrEMBL;Acc:A0A178USD1]	19813346	19814554	36.15
AT5G48880	KAT5	PKT2 [Source:UniProtKB/TrEMBL;Acc:A0A178UEC0]	19814395	19817955	37.77
AT5G48890	LATE	LATE [Source:UniProtKB/TrEMBL;Acc:A0A178UK76]	19819948	19821007	41.42
AT5G48900		Probable pectate lyase 20 [Source:UniProtKB/Swiss-Prot;Acc:Q93WF1]	19825078	19829140	34.21
AT5G07295			19829614	19829971	43.58
AT5G48905	LCR12	Putative defensin-like protein 165 [Source:UniProtKB/Swiss-Prot;Acc:P82727]	19831255	19831917	28.05
AT5G48910	PCMP-H38	Pentatricopeptide repeat-containing protein At5g48910 [Source:UniProtKB/Swiss-Prot;Acc:Q9FI80]	19832785	19835148	40.91
AT5G48920	TED7	At5g48920 [Source:UniProtKB/TrEMBL;Acc:Q9FI79]	19835337	19836258	42.19
AT5G48930	HST	Shikimate O-hydroxycinnamoyltransferase [Source:UniProtKB/Swiss-Prot;Acc:Q9FI78]	19836299	19838235	42.95
AT5G48940	RCH1	LRR receptor-like serine/threonine-protein kinase RCH1 [Source:UniProtKB/Swiss-Prot;Acc:C0LGV1]	19839663	19843859	40.41
AT5G48945	LCR46	LCR46 [Source:UniProtKB/TrEMBL;Acc:A0A178UR05]	19844683	19845548	28.64
AT5G48950	DHNAT2	1,4-dihydroxy-2-naphthoyl-CoA thioesterase 2 [Source:UniProtKB/Swiss-Prot;Acc:Q9FI76]	19845999	19847435	33.89
AT5G48953	LCR86	Defensin-like protein 76 [Source:UniProtKB/Swiss-Prot;Acc:P82795]	19847642	19848284	31.1
AT5G48960		HAD-superfamily hydrolase, subfamily IG, 5'-nucleotidase [Source:UniProtKB/TrEMBL;Acc:F4K3A9]	19849437	19853598	38.01
AT5G48970		Mitochondrial thiamine diphosphate carrier 2 [Source:UniProtKB/Swiss-Prot;Acc:Q9FI73]	19856830	19859847	37.34
AT5G48980		F-box/kelch-repeat protein At5g48980 [Source:UniProtKB/Swiss-Prot;Acc:Q9FI72]	19860708	19862052	40.97
AT5G48990		F-box/kelch-repeat protein At5g48990 [Source:UniProtKB/Swiss-Prot;Acc:Q9FI71]	19862235	19863975	41.24
AT5G49000		F-box/kelch-repeat protein At5g49000 [Source:UniProtKB/Swiss-Prot;Acc:Q9FI70]	19864410	19865825	41.53
AT5G49010	SLD5	DNA replication complex GINS protein SLD5 [Source:UniProtKB/TrEMBL;Acc:A0A178UAX9]	19866231	19868877	37.36
AT5G49015		Expressed protein [Source:TAIR;Acc:AT5G49015]	19868926	19870410	35.02
AT5G49020	PRMT14	Probable histone-arginine methyltransferase 1.4 [Source:UniProtKB/Swiss-Prot;Acc:A3KPF2]	19871191	19874974	38.69
AT5G49030	OVA2	tRNA synthetase class I (I, L, M and V) family protein [Source:UniProtKB/TrEMBL;Acc:F4K4Q2]	19874925	19883297	38.56
AT5G49040	DIR3	Dirigent protein 3 [Source:UniProtKB/Swiss-Prot;Acc:Q9FI66]	19882676	19883251	47.22
AT5G49050		Universal stress A-like protein [Source:UniProtKB/TrEMBL;Acc:Q9FI65]	19883929	19884381	47.46
AT5G07325			19885473	19886179	44.41
AT5G49060	ATJ49	Chaperone protein dnaJ 49 [Source:UniProtKB/Swiss-Prot;Acc:Q9FH28]	19886308	19888571	38.03
AT5G49070	KCS21	Probable 3-ketoacyl-CoA synthase 21 [Source:UniProtKB/Swiss-Prot;Acc:Q9FH27]	19888751	19890174	42.06
AT5G49100		Vitellogenin-like protein [Source:UniProtKB/TrEMBL;Acc:Q9FH24]	19897370	19899301	37.84
AT5G49110		unknown protein; INVOLVED IN: biological_process unknown; LOCATED IN: cellular_component unknown; EXPRESSED IN: cultured cell; F	19900150	19906171	39.59
AT5G07335			19902818	19902970	35.95
AT5G07345			19903159	19903323	43.03
AT5G49120	FLZ15	FCS-Like Zinc finger 15 [Source:UniProtKB/Swiss-Prot;Acc:Q9FH22]	19908614	19909452	37.31
AT5G49138		other RNA [Source:TAIR;Acc:AT5G49138]	19915806	19917787	44.5
AT5G49130	DTX55	Protein DETOXIFICATION 55 [Source:UniProtKB/Swiss-Prot;Acc:Q9FH21]	19915872	19917967	44.75
AT5G49140		Disease resistance protein (TIR-NBS-LRR class) family [Source:UniProtKB/TrEMBL;Acc:Q9FH20]	19918971	19923415	36.06
AT5G49152		other RNA [Source:TAIR;Acc:AT5G49152]	19924322	19929377	38.69
AT5G49150	ATGEX2	Gamete expressed 2 [Source:UniProtKB/TrEMBL;Acc:W0TY53]	19924354	19929256	38.77
AT5G49160	DMT1	DNA (cytosine-5)-methyltransferase 1 [Source:UniProtKB/Swiss-Prot;Acc:P34881]	19932114	19938470	41.1
AT5G49170		Uncharacterized protein At5g49170 [Source:UniProtKB/TrEMBL;Acc:Q0WQJ1]	19938540	19939797	36.88
AT5G49180	PME58	Pectinesterase [Source:UniProtKB/TrEMBL;Acc:A0A178UKT8]	19940462	19943015	40.64
AT5G49190	SUS2	Sucrose synthase 2 [Source:UniProtKB/Swiss-Prot;Acc:Q00917]	19943181	19947588	40.27
AT5G49200	ZFWD4	Zinc finger CCCH domain-containing protein 62 [Source:UniProtKB/Swiss-Prot;Acc:Q9FE91]	19947689	19949175	42.5
AT5G49210		AT5g49210/K21P3_8 [Source:UniProtKB/TrEMBL;Acc:Q945P2]	19949691	19951713	38.75
AT5G49215		Pectin lyase-like superfamily protein [Source:UniProtKB/TrEMBL;Acc:Q8RX04]	19953459	19956145	38.44
AT5G49220		Uncharacterized protein At5g49220 [Source:UniProtKB/TrEMBL;Acc:Q8VZK6]	19956218	19958861	39.56
AT5G49230	DI19-7	Protein DEHYDRATION-INDUCED 19 homolog 7 [Source:UniProtKB/Swiss-Prot;Acc:Q9FJ17]	19958872	19960681	35.86
AT5G49240	APRR4	Putative two-component response regulator-like APRR4 [Source:UniProtKB/Swiss-Prot;Acc:Q9FJ16]	19962934	19964351	36.53
AT5G49250		Beta-galactosidase related protein [Source:UniProtKB/TrEMBL;Acc:Q9FJ15]	19965343	19965945	42.62
AT5G49260		Putative uncharacterized protein [Source:UniProtKB/TrEMBL;Acc:Q9FJ14]	19966799	19967527	38.82
AT5G49270	COBL9	COBRA-like protein 9 [Source:UniProtKB/Swiss-Prot;Acc:Q9FJ13]	19971884	19974530	42.65
AT5G49280		At5g49280 [Source:UniProtKB/TrEMBL;Acc:Q9FJ12]	19976222	19977620	36.81
AT5G07355			19979318	19979668	34.76
AT5G49290	ATRLP56	receptor like protein 56 [Source:TAIR;Acc:AT5G49290]	19979559	19984131	36.67

AT5G49300	GATA16	Uncharacterized protein At5g49300 (Fragment) [Source:UniProtKB/TrEMBL;Acc:C0SVS9]	19984626	19985788	38.78
AT5G07365			19987573	19987785	36.15
AT5G49305		Importin subunit alpha [Source:UniProtKB/TrEMBL;Acc:A0A1P8BDF2]	19988464	19991580	37.92
AT5G07385			19988554	19989030	39.41
AT5G49310	IMPA5	Importin subunit alpha-5 [Source:UniProtKB/Swiss-Prot;Acc:Q9FJ09]	19991946	19994672	38.5
AT5G49320		At5g49320 [Source:UniProtKB/TrEMBL;Acc:Q9FJ08]	19994994	19996357	37.17
AT5G49330	MYB111	Transcription factor MYB111 [Source:UniProtKB/Swiss-Prot;Acc:Q9FJ07]	19998823	20001572	35.82
AT5G49340	TBL4	Protein trichome birefringence-like 4 [Source:UniProtKB/Swiss-Prot;Acc:Q9FJ06]	20007313	20009038	40.96
AT5G49350		At5g49350 [Source:UniProtKB/TrEMBL;Acc:Q84Q60]	20009520	20011099	43.42
AT5G49360	BXL1	Beta-D-xylosidase 1 [Source:UniProtKB/Swiss-Prot;Acc:Q9FGY1]	20011812	20016857	37.34
AT5G49390		LOCATED IN: endomembrane system; EXPRESSED IN: 17 plant structures; EXPRESSED DURING: 11 growth stages; BEST Arabidopsis thal	20023442	20030387	37.06
AT5G49400		At5g49400 [Source:UniProtKB/TrEMBL;Acc:Q6NMK2]	20030652	20032283	40.2
AT5G49410		At5g49410 [Source:UniProtKB/TrEMBL;Acc:Q9FGX6]	20032287	20034020	35.52
AT5G49420		MADS-box protein AGL84 [Source:UniProtKB/TrEMBL;Acc:Q7X9H5]	20034672	20036205	41.26
AT5G49430		WD40/YVTN repeat and Bromo-WDR9-I-like domain-containing protein [Source:UniProtKB/TrEMBL;Acc:F4K639]	20036988	20046702	39.14
AT5G49435		pre-tRNA [Source:TAIR;Acc:AT5G49435]	20046823	20046894	55.56
AT5G49440		AT5g49440/K7J8_12 [Source:UniProtKB/TrEMBL;Acc:Q9FGX3]	20048239	20049776	43.37
AT5G49450	BZIP1	Basic leucine zipper 1 [Source:UniProtKB/Swiss-Prot;Acc:Q9FGX2]	20051829	20052735	36.93
AT5G49460	ACLB-2	ATP-citrate synthase beta chain protein 2 [Source:UniProtKB/Swiss-Prot;Acc:Q9FGX1]	20054501	20058555	38.96
AT5G49470		PAS domain-containing protein tyrosine kinase family protein [Source:UniProtKB/TrEMBL;Acc:F4K645]	20063331	20068793	38.82
AT5G49480	ATCP1	Calcium-binding protein CP1 [Source:UniProtKB/Swiss-Prot;Acc:Q9FDX6]	20070489	20071682	37.19
AT5G49490	AGL83	AGAMOUS-like 83 [Source:UniProtKB/TrEMBL;Acc:Q9FGZ5]	20075328	20076185	41.38
AT5G49500	SRP-54B	Signal recognition particle 54 kDa protein 2 [Source:UniProtKB/Swiss-Prot;Acc:P49966]	20077304	20080329	39.09
AT5G49510	PFD3	Probable prefoldin subunit 3 [Source:UniProtKB/Swiss-Prot;Acc:P57741]	20080600	20083042	37.04
AT5G49520	WRKY48	Probable WRKY transcription factor 48 [Source:UniProtKB/Swiss-Prot;Acc:Q9FGZ4]	20090648	20093346	35.64
AT5G07395			20095589	20096207	36.35
AT5G49525		At5g49525 [Source:UniProtKB/TrEMBL;Acc:Q8L7F0]	20096932	20097551	35.65
AT5G49530		SIN-like family protein [Source:UniProtKB/TrEMBL;Acc:Q9FGZ3]	20100545	20104605	38.24
AT5G49540		AT5g49540/K6M13_9 [Source:UniProtKB/TrEMBL;Acc:Q9AST5]	20104481	20106278	34.54
AT5G49550	BLOS2	Biogenesis of lysosome-related organelles complex 1 subunit 2 [Source:UniProtKB/Swiss-Prot;Acc:F4K657]	20106532	20107208	37.81
AT5G49555		FAD/NAD(P)-binding oxidoreductase family protein [Source:UniProtKB/TrEMBL;Acc:F4K658]	20107229	20110638	40.15
AT5G49560		Putative methyltransferase family protein [Source:UniProtKB/TrEMBL;Acc:Q9FGZ0]	20110832	20111806	47.28
AT5G49570	PNG1	Transglutaminase [Source:UniProtKB/TrEMBL;Acc:A5PHD1]	20111859	20116269	38.68
AT5G07405			20112031	20112072	26.19
AT5G07415			20112276	20112431	40.38
AT5G07425			20120207	20120724	44.4
AT5G07435			20120670	20120891	41.44
AT5G49580		Chaperone DnaJ-domain superfamily protein [Source:UniProtKB/TrEMBL;Acc:Q9FGY8]	20123563	20127509	38.48
AT5G49590		Putative uncharacterized protein [Source:UniProtKB/TrEMBL;Acc:Q9FGY6]	20128736	20129554	40.54
AT5G49600		Similarity to unknown protein [Source:UniProtKB/TrEMBL;Acc:Q9FGY5]	20130752	20131295	40.44
AT5G49610		F-box protein At5g49610 [Source:UniProtKB/Swiss-Prot;Acc:Q9FGY4]	20131274	20132744	37.73
AT5G49615	TAS3b	TAS3b (trans-acting siRNA 3b); other RNA [Source:TAIR;Acc:AT5G49615]	20134200	20134786	33.22
AT5G49620	AtMYB78	myb domain protein 78 [Source:TAIR;Acc:AT5G49620]	20137214	20139609	34.18
AT5G49630	AAP6	AAP6 [Source:UniProtKB/TrEMBL;Acc:A0A178UHU7]	20142430	20146690	34.73
AT5G49640		At5g49640 [Source:UniProtKB/TrEMBL;Acc:Q9LT52]	20151163	20151842	44.85
AT5G49645			20151163	20151882	45
AT5G49650	XK-2	Xylulose kinase 2 [Source:UniProtKB/Swiss-Prot;Acc:Q949W8]	20152731	20156613	39.71
AT5G49660	CEPR1	Receptor protein-tyrosine kinase CEPR1 [Source:UniProtKB/Swiss-Prot;Acc:Q9FGL5]	20161140	20164729	41.73
AT5G49665	WAV3	E3 ubiquitin-protein ligase WAV3 [Source:UniProtKB/Swiss-Prot;Acc:Q9LTA6]	20166891	20169923	45.27
AT5G07445			20176062	20176345	48.59
AT5G49680	KIP	Protein KINKY POLLEN [Source:UniProtKB/Swiss-Prot;Acc:Q6IMT0]	20176080	20188567	39.37
AT5G49690	UGT91C1	UDP-glycosyltransferase 91C1 [Source:UniProtKB/Swiss-Prot;Acc:Q9LTA3]	20189655	20191604	40.97
AT5G49700	AHL17	AT-hook motif nuclear-localized protein 17 [Source:UniProtKB/Swiss-Prot;Acc:Q9LTA2]	20192159	20193800	41.29
AT5G49710		RING finger protein [Source:UniProtKB/TrEMBL;Acc:Q0WP38]	20195303	20197419	38.12
AT5G49720	KOR	Endoglucanase 25 [Source:UniProtKB/Swiss-Prot;Acc:Q38890]	20197391	20200468	40.29
AT5G49730	FRO6	Ferric reduction oxidase 6 [Source:UniProtKB/Swiss-Prot;Acc:Q8RWS6]	20201037	20204595	38.44
AT5G49740	FRO7	Ferric reduction oxidase 7, chloroplastic [Source:UniProtKB/Swiss-Prot;Acc:Q3KTM0]	20205302	20208777	39.04
AT5G49750		Leucine-rich repeat (LRR) family protein [Source:UniProtKB/TrEMBL;Acc:F4K682]	20210878	20213734	34.27
AT5G07455			20214323	20214952	36.03
AT5G49760		At5g49760 [Source:UniProtKB/TrEMBL;Acc:Q8GZ99]	20216332	20221382	37.18
AT5G49770		Probable leucine-rich repeat receptor-like protein kinase At5g49770 [Source:UniProtKB/Swiss-Prot;Acc:Q9LT96]	20222669	20227466	36.79
AT5G07465			20228268	20228656	29.31
AT5G49780		Leucine-rich repeat protein kinase family protein [Source:TAIR;Acc:AT5G49780]	20228645	20233095	36.62
AT5G49800		At5g49800 [Source:UniProtKB/TrEMBL;Acc:Q5HZ48]	20234509	20236329	37.07
AT5G49810	MMT1	Methionine S-methyltransferase [Source:UniProtKB/Swiss-Prot;Acc:Q9LTB2]	20239293	20246398	38.12
AT5G49820	RUS6	Protein root UVB sensitive 6 [Source:UniProtKB/Swiss-Prot;Acc:Q93YU2]	20246391	20249622	38.95
AT5G49830	EXO84B	Exocyst complex component 84B [Source:UniProtKB/TrEMBL;Acc:F4K7F5]	20250419	20255089	40.61
AT5G49840		ATP-dependent Clp protease [Source:TAIR;Acc:AT5G49840]	20255173	20259982	38.07
AT5G49850	JAL46	Jacalin-related lectin 46 [Source:UniProtKB/Swiss-Prot;Acc:Q9LTA8]	20259440	20262585	38.75
AT5G49860	JAL47	Jacalin-related lectin 47 [Source:UniProtKB/Swiss-Prot;Acc:Q9LTA7]	20264567	20265334	40.62
AT5G49870	JAL48	Jacalin-related lectin 48 [Source:UniProtKB/Swiss-Prot;Acc:Q9LTY2]	20270101	20272895	37.89
AT5G07485			20275697	20276186	34.69
AT5G07495			20276669	20277046	38.36
AT5G49880	MAD1	Mitotic spindle checkpoint protein MAD1 [Source:UniProtKB/Swiss-Prot;Acc:Q9LTY1]	20282111	20288084	36.39
AT5G49890	CLC-C	Chloride channel protein CLC-c [Source:UniProtKB/Swiss-Prot;Acc:Q96282]	20288055	20292432	39.29
AT5G49900		Non-lysosomal glucosylceramidase [Source:UniProtKB/TrEMBL;Acc:Q8GUI9]	20296518	20302575	37.74
AT5G49910	HSP70-7	Heat shock 70 kDa protein 7, chloroplastic [Source:UniProtKB/Swiss-Prot;Acc:Q9LTX9]	20303334	20306469	41.9
AT5G49920		At5g49920 [Source:UniProtKB/TrEMBL;Acc:Q9LTX8]	20306409	20307943	42.28
AT5G07515			20306885	20307120	49.58
AT5G49930	emb1441	Gb [Source:UniProtKB/TrEMBL;Acc:Q9LTX7]	20308033	20312892	38.91
AT5G49940	NIFU2	NifU-like protein 2, chloroplastic [Source:UniProtKB/Swiss-Prot;Acc:Q93W20]	20315390	20317401	38.57
AT5G49945		Uncharacterized protein At5g49945 [Source:UniProtKB/Swiss-Prot;Acc:Q94CC0]	20317405	20319830	40.85
AT5G07525			20318418	20318925	36.81
AT5G49950		Alpha/beta-Hydrolases superfamily protein [Source:UniProtKB/TrEMBL;Acc:Q9LTX5]	20319733	20323649	37.81
AT5G49960		Probable ion channel POLLUX [Source:UniProtKB/Swiss-Prot;Acc:Q9LTX4]	20323785	20327881	38.05
AT5G49970	PPOX1	Pyridoxine/pyridoxamine 5'-phosphate oxidase 1, chloroplastic [Source:UniProtKB/Swiss-Prot;Acc:Q9LTX3]	20329103	20333172	38.18
AT5G49980	AFB5	AFB5 [Source:UniProtKB/TrEMBL;Acc:A0A178UB83]	20333897	20336671	41.69
AT5G49990	NAT5	Nucleobase-ascorbate transporter 5 [Source:UniProtKB/Swiss-Prot;Acc:Q8RWE9]	20337586	20342035	37.87
AT5G50000		Protein kinase superfamily protein [Source:UniProtKB/TrEMBL;Acc:Q9FGB1]	20342505	20345302	37.42
AT5G50010	BHLH145	Transcription factor bHLH145 [Source:UniProtKB/Swiss-Prot;Acc:Q9FGB0]	20348615	20350830	39.03
AT5G50020		DHHC-type zinc finger family protein [Source:TAIR;Acc:AT5G50020]	20351157	20354436	37.56
AT5G50030		At5g50030 [Source:UniProtKB/TrEMBL;Acc:Q9FGA8]	20354589	20355706	39.98
AT5G50040		Plant invertase/pectin methylesterase inhibitor superfamily protein [Source:UniProtKB/TrEMBL;Acc:Q9FGA7]	20356876	20358381	34.06
AT5G50050		At5g50050 [Source:UniProtKB/TrEMBL;Acc:Q9FGA6]	20360015	20361031	35.59
AT5G50060		Plant invertase/pectin methylesterase inhibitor superfamily protein [Source:UniProtKB/TrEMBL;Acc:Q9FGA5]	20362486	20363597	36.42
AT5G50070		Plant invertase/pectin methylesterase inhibitor superfamily protein [Source:UniProtKB/TrEMBL;Acc:Q9FGA4]	20364516	20365481	35.82
AT5G50080	ERF110	Ethylene response factor 110 [Source:UniProtKB/TrEMBL;Acc:G8IQH2]	20365722	20367093	40.96
AT5G50090		At5g50090 [Source:UniProtKB/TrEMBL;Acc:Q9FGA2]	20369657	20371391	35.68
AT5G50100		Uncharacterized protein At5g50100, chloroplastic [Source:UniProtKB/Swiss-Prot;Acc:Q8W485]	20371798	20373441	33.88
AT5G50110		S-adenosyl-L-methionine-dependent methyltransferases superfamily protein [Source:UniProtKB/TrEMBL;Acc:F4K7J0]	20373491	20376252	35.12
AT5G07535			20377777	20377984	40.87
AT5G50115		CONTAINS InterPro DOMAIN/s: Molecular chaperone, heat shock protein, Hsp40, DnaJ (InterPro:IPR015609); BEST Arabidopsis thaliana prot	20379489	20381036	41.67
AT5G50120		Similarity to GTP-binding regulatory protein and WD-repeat protein [Source:UniProtKB/TrEMBL;Acc:Q9FG99]	20382310	20384025	39.1
AT5G07555			20384901	20385459	37.03
AT5G50130		NAD(P)-binding Rossmann-fold superfamily protein [Source:UniProtKB/TrEMBL;Acc:Q8RWK2]	20389915	20393306	32.49

	AT5G07565			20393330	20393648	30.41
	AT5G07575			20394269	20394760	38.62
	AT5G50140		Ankyrin repeat family protein [Source:UniProtKB/TrEMBL;Acc:Q9FG97]	20395856	20398197	38.73
	AT5G50150		AT5g50150/MPF21_17 [Source:UniProtKB/TrEMBL;Acc:Q9FG96]	20402217	20406731	32.58
	AT5G07595			20412424	20412662	35.15
	AT5G07585			20412424	20412701	35.97
	AT5G50160	FRO8	Ferric reduction oxidase 8, mitochondrial [Source:UniProtKB/Swiss-Prot;Acc:Q8VY13]	20415764	20418847	36.45
	AT5G50170		C2 and GRAM domain-containing protein At5g50170 [Source:UniProtKB/Swiss-Prot;Acc:Q9FGS8]	20420951	20425604	39.15
	AT5G50175		Transmembrane protein [Source:UniProtKB/TrEMBL;Acc:Q2V301]	20427749	20428719	38
	AT5G50180		At5g50180 [Source:UniProtKB/TrEMBL;Acc:Q9FGS7]	20430453	20433146	36.71
	AT5G50190		Unknown gene [Source:TAIR;Acc:AT5G50190]	20433507	20434543	34.52
	AT5G50200	NRT3.1	High-affinity nitrate transporter 3.1 [Source:UniProtKB/Swiss-Prot;Acc:Q9FGS5]	20436180	20437815	38.02
	AT5G50210	QS	SUFE3 [Source:UniProtKB/TrEMBL;Acc:A0A178UIT6]	20442613	20446050	39.35
	AT5G50220		Putative F-box protein At5g50220 [Source:UniProtKB/Swiss-Prot;Acc:Q9FGS3]	20446068	20447200	41.31
	AT5G50230	ATG16	Autophagy-related protein 16 [Source:UniProtKB/Swiss-Prot;Acc:Q6NNP0]	20448310	20451062	40.07
	AT5G50240	PIMT2	Protein-L-isoaspartate O-methyltransferase 2 [Source:UniProtKB/Swiss-Prot;Acc:Q64J17]	20451228	20452569	41.88
	AT5G50250	CP31B	RNA-binding protein CP31B, chloroplastic [Source:UniProtKB/Swiss-Prot;Acc:Q9FGS0]	20452487	20454041	38.59
	AT5G50260	CEP1	KDEL-tailed cysteine endopeptidase CEP1 [Source:UniProtKB/Swiss-Prot;Acc:Q9FGR9]	20455317	20457068	37.56
	AT5G50270		Putative FBD-associated F-box protein At5g50270 [Source:UniProtKB/Swiss-Prot;Acc:Q9FGR8]	20457487	20458996	38.74
	AT5G50280	EMB1006	Pentatricopeptide repeat-containing protein At5g50280, chloroplastic [Source:UniProtKB/Swiss-Prot;Acc:Q9FGR7]	20458939	20461710	40.3
	AT5G50290		Wall-associated receptor kinase galacturonan-binding protein [Source:UniProtKB/TrEMBL;Acc:Q5XV04]	20461777	20464616	35.35
	AT5G50300	AZG2	Xanthine/uracil permease family protein [Source:TAIR;Acc:AT5G50300]	20466068	20468217	44.79
	AT5G07605			20473294	20473536	34.57
	AT5G50310		AT5g50310/MXI22_1 [Source:UniProtKB/TrEMBL;Acc:Q945N1]	20474457	20479478	36.68
	AT5G50320	HAG3	Elongator complex protein 3 [Source:UniProtKB/TrEMBL;Acc:A0A178UGS1]	20480626	20484945	39.1
	AT5G50330		At5g50330 [Source:UniProtKB/TrEMBL;Acc:Q0WSU9]	20485134	20488702	36.87
	AT5G50335		At5g50335 [Source:UniProtKB/TrEMBL;Acc:Q8LEB7]	20489033	20489727	31.8
	AT5G50340		ATP-dependent peptidases;nucleotide binding;serine-type endopeptidases;DNA helicases;ATP binding;damaged DNA binding;nucleoside-tripho	20491393	20496589	37.87
	AT5G50345	ESFL11	EMBRYO SURROUNDING FACTOR 1-like protein 11 [Source:UniProtKB/Swiss-Prot;Acc:A8MRV5]	20497401	20497868	31.2
	AT5G50350		Uncharacterized protein At5g50350 [Source:UniProtKB/TrEMBL;Acc:Q9FK37]	20498476	20501786	40.38
	AT5G50360		von willebrand factor A domain protein [Source:UniProtKB/TrEMBL;Acc:Q9FK36]	20505089	20506354	43.13
	AT5G50361		unknown protein; LOCATED IN: chloroplast; Ha. [Source:TAIR;Acc:AT5G50361]	20506381	20506578	50
	AT5G50365		Transmembrane protein [Source:UniProtKB/TrEMBL;Acc:A0A1R7T3F0]	20508126	20508776	33.79
	AT5G07615			20508179	20508541	43.53
	AT5G50370	ADK2	Adenylate kinase 3 [Source:UniProtKB/Swiss-Prot;Acc:Q9FK35]	20509115	20510810	39.62
	AT5G50375	CPI1	Cyclopropyl isomerase [Source:UniProtKB/TrEMBL;Acc:F4K8Y4]	20511642	20514346	38.3
	AT5G50380	ATEXO70F1	Exocyst subunit Exo70 family protein [Source:UniProtKB/TrEMBL;Acc:F4K8Y6]	20516055	20518815	41.36
	AT5G50390	PCMP-H58	Pentatricopeptide repeat-containing protein At5g50390, chloroplastic [Source:UniProtKB/Swiss-Prot;Acc:Q9FK33]	20520600	20523212	40.57
	AT5G50400	PAP27	Probable inactive purple acid phosphatase 27 [Source:UniProtKB/Swiss-Prot;Acc:Q5MAU8]	20523366	20526521	39.96
	AT5G50410		At5g50410 [Source:UniProtKB/TrEMBL;Acc:Q9FK31]	20528068	20529885	37.95
	AT5G50420	OFUT36	O-fucosyltransferase 36 [Source:UniProtKB/Swiss-Prot;Acc:Q9FK30]	20530200	20532856	40.01
	AT5G50423		Defensin-like protein 222 [Source:UniProtKB/Swiss-Prot;Acc:Q2V300]	20532860	20533471	27.94
	AT5G50430	UBC33	Probable ubiquitin-conjugating enzyme E2 33 [Source:UniProtKB/Swiss-Prot;Acc:Q9FK29]	20534058	20537138	35.61
	AT5G50440	MEMB12	Membrin-12 [Source:UniProtKB/Swiss-Prot;Acc:Q9FK28]	20537426	20538996	38.26
	AT5G50443			20540335	20541211	33.52
	AT5G07635			20540612	20540859	37.1
	AT5G50450		F-box protein At5g50450 [Source:UniProtKB/Swiss-Prot;Acc:Q9FK27]	20543225	20544857	42.99
	AT5G50460	SEC61G2	Protein transport protein Sec61 subunit gamma-1 [Source:UniProtKB/Swiss-Prot;Acc:P0DI74]	20551132	20552670	36.45
	AT5G07655			20554881	20555053	32.95
	AT5G50470	NFYC7	Uncharacterized protein At5g50470 (Fragment) [Source:UniProtKB/TrEMBL;Acc:C0SVT1]	20555120	20555758	52.9
	AT5G50480	NFYC6	Nuclear transcription factor Y subunit C-6 [Source:UniProtKB/Swiss-Prot;Acc:Q9FGP7]	20557574	20558487	47.26
	AT5G50490	NFYC5	Nuclear transcription factor Y subunit C-5 [Source:UniProtKB/Swiss-Prot;Acc:Q9FGP6]	20560434	20561228	43.14
	AT5G50500		Putative uncharacterized protein [Source:UniProtKB/TrEMBL;Acc:Q9FGP5]	20562150	20562557	38.73
	AT5G07665			20563579	20563784	39.81
	AT5G07695			20563618	20564100	37.89
	AT5G50510		Molecular chaperone Hsp40/DnaJ family protein [Source:UniProtKB/TrEMBL;Acc:Q9LUF9]	20567386	20568075	42.32
	AT5G50520		Major facilitator superfamily protein [Source:UniProtKB/TrEMBL;Acc:Q9LUF8]	20569533	20571656	42
	AT5G50530	CBSCBSPB4	CBS domain-containing protein CBSCBSPB5 [Source:UniProtKB/Swiss-Prot;Acc:P0DH79]	20571681	20575250	36.13
	AT5G50540		At5g50645 [Source:UniProtKB/TrEMBL;Acc:Q9FGP4]	20574577	20575538	37.63
	AT5G50550		At5g50550 [Source:UniProtKB/TrEMBL;Acc:Q9FGP3]	20576733	20579103	39.43
	AT5G50560		At5g50660 [Source:UniProtKB/TrEMBL;Acc:Q9LUF5]	20579027	20579633	39.54
	AT5G50565		unknown protein; BEST Arabidopsis thaliana protein match is: unknown protein (TAIR:AT5G50665.2); Ha. [Source:TAIR;Acc:AT5G50565]	20580113	20582178	33.3
	AT5G50562		unknown protein; Ha. [Source:TAIR;Acc:AT5G50562]	20580269	20580442	43.1
	AT5G50570	SPL13A	Squamosa promoter-binding-like protein 13B [Source:UniProtKB/Swiss-Prot;Acc:P0DI11]	20582083	20584721	35.13
	AT5G50580	SAE1B-2	SUMO-activating enzyme subunit 1B-1 [Source:UniProtKB/Swiss-Prot;Acc:P0DI12]	20585349	20587761	37.67
	AT5G50590	HSD4	11-beta-hydroxysteroid dehydrogenase-like 4A [Source:UniProtKB/Swiss-Prot;Acc:P0DKC7]	20587936	20589490	37.88
	AT5G50600	HSD1	11-beta-hydroxysteroid dehydrogenase 1A [Source:UniProtKB/Swiss-Prot;Acc:P0DKC5]	20589702	20591937	37.25
	AT5G50610		Transmembrane protein [Source:UniProtKB/TrEMBL;Acc:Q9LUF0]	20593600	20594379	37.69
	AT5G50620		Molecular chaperone Hsp40/DnaJ family protein [Source:UniProtKB/TrEMBL;Acc:Q9LUF9]	20600727	20601416	42.32
	AT5G50630		Major facilitator superfamily protein [Source:UniProtKB/TrEMBL;Acc:Q9LUF8]	20602874	20604997	42
	AT5G50640	CBSCBSPB4	CBS domain-containing protein CBSCBSPB5 [Source:UniProtKB/Swiss-Prot;Acc:P0DH79]	20605218	20608594	36.54
	AT5G50650		At5g50550 [Source:UniProtKB/TrEMBL;Acc:Q9FGP3]	20610075	20612445	39.43
	AT5G50660		At5g50660 [Source:UniProtKB/TrEMBL;Acc:Q9LUF5]	20612369	20612975	39.54
	AT5G50665		unknown protein; BEST Arabidopsis thaliana protein match is: unknown protein (TAIR:AT5G50565.2); Ha. [Source:TAIR;Acc:AT5G50665]	20613455	20615520	33.3
	AT5G50670	SPL13A	Squamosa promoter-binding-like protein 13B [Source:UniProtKB/Swiss-Prot;Acc:P0DI11]	20615565	20618063	35.49
	AT5G50680	SAE1B-2	SUMO-activating enzyme subunit 1B-1 [Source:UniProtKB/Swiss-Prot;Acc:P0DI12]	20618733	20621103	37.87
	AT5G50690	HSD4	11-beta-hydroxysteroid dehydrogenase-like 4B [Source:UniProtKB/Swiss-Prot;Acc:Q9LUF2]	20621278	20622832	37.88
	AT5G50700	HSD1	11-beta-hydroxysteroid dehydrogenase 1A [Source:UniProtKB/Swiss-Prot;Acc:P0DKC5]	20623024	20625279	37.15
	AT5G50710		Transmembrane protein [Source:UniProtKB/TrEMBL;Acc:Q9LUF0]	20626942	20627720	37.74
	AT5G50717	MIR405B	MIR405B; miRNA [Source:TAIR;Acc:AT5G50717]	20632514	20632637	30.65
	AT5G50720	HVA22E	HVA22-like protein [Source:UniProtKB/TrEMBL;Acc:Q0IGM2]	20633193	20634618	33.8
	AT5G50740		Heavy metal transport/detoxification superfamily protein [Source:UniProtKB/TrEMBL;Acc:B3H4U8]	20635826	20638104	33.52
	AT5G50750	RGP4	Probable UDP-arabinopyranose mutase 4 [Source:UniProtKB/Swiss-Prot;Acc:Q9LUE6]	20640992	20642787	39.37
	AT5G07705			20643104	20643339	36.86
	AT5G50760		At5g50760 [Source:UniProtKB/TrEMBL;Acc:Q9LUE5]	20644525	20645698	36.12
	AT5G50770	HSD6	11-beta-hydroxysteroid dehydrogenase-like 6 [Source:UniProtKB/Swiss-Prot;Acc:Q9LUE4]	20646680	20648725	37.73
	AT5G50780		Histidine kinase-, DNA gyrase B-, and HSP90-like ATPase family protein [Source:TAIR;Acc:AT5G50780]	20650086	20655401	37.81
	AT5G50790	SWEET10	Bidirectional sugar transporter SWEET10 [Source:UniProtKB/Swiss-Prot;Acc:Q9LUE3]	20656193	20658001	35.38
MQTL-8/Chr5	AT5G55180		O-Glycosyl hydrolases family 17 protein [Source:UniProtKB/TrEMBL;Acc:F4K3D8]	22388521	22390763	43.16
	AT5G55190	RAN3	GTP-binding nuclear protein [Source:UniProtKB/TrEMBL;Acc:A0A178UD71]	22392063	22394185	39.24
	AT5G55200	Mge1	GrpE protein homolog 1, mitochondrial [Source:UniProtKB/Swiss-Prot;Acc:Q9FLP3]	22394550	22396572	38.36
	AT5G55210		At5g55210 [Source:UniProtKB/TrEMBL;Acc:Q9FLP2]	22396426	22397446	41.23
	AT5G55220	TIG	Trigger factor-like protein TIG, Chloroplastic [Source:UniProtKB/Swiss-Prot;Acc:Q8S9L5]	22397457	22400856	36.71
	AT5G55230	ATMAP65-1	Microtubule-associated proteins 65-1 [Source:UniProtKB/TrEMBL;Acc:F4K3E4]	22401927	22405568	39.35
	AT5G55240	PXG2	Peroxygenase 2 [Source:UniProtKB/Swiss-Prot;Acc:Q9FLN9]	22405896	22407351	39.49
	AT5G55250	IAMT1	Indole-3-acetate O-methyltransferase 1 [Source:UniProtKB/Swiss-Prot;Acc:Q9FLN8]	22407427	22410974	36.16
	AT5G55260	PPX2	protein phosphatase X 2 [Source:TAIR;Acc:AT5G55260]	22416578	22418990	38
	AT5G55270		Putative uncharacterized protein [Source:UniProtKB/TrEMBL;Acc:Q9FLN7]	22419147	22420518	42.27
	AT5G55280	FTSZ1	Cell division protein FtsZ homolog 1, chloroplastic [Source:UniProtKB/Swiss-Prot;Acc:Q42545]	22420600	22422649	40.78
	AT5G55290	VHA-E1	V-type proton ATPase subunit e1 [Source:UniProtKB/Swiss-Prot;Acc:Q9FLN5]	22422673	22424380	36.59
	AT5G55300	TOP1ALPHA	DNA topoisomerase I alpha [Source:UniProtKB/TrEMBL;Acc:F4K3F4]	22424502	22429514	37.98
	AT5G55310	TOP1B	DNA topoisomerase 1 beta [Source:UniProtKB/Swiss-Prot;Acc:Q9FJ79]	22430105	22435134	37.5
	AT5G55320	AT7	Probable long-chain-alcohol O-fatty-acyltransferase 7 [Source:UniProtKB/Swiss-Prot;Acc:Q9FJ78]	22435607	22436943	39.57
	AT5G55330	AT6	Probable long-chain-alcohol O-fatty-acyltransferase 6 [Source:UniProtKB/Swiss-Prot;Acc:Q9FJ77]	22437471	22438884	39.46
	AT5G55340	AT5	Probable long-chain-alcohol O-fatty-acyltransferase 5 [Source:UniProtKB/Swiss-Prot;Acc:Q9FJ76]	22439714	22441115	41.73

AT5G55350	AT4	Probable long-chain-alcohol O-fatty-acyltransferase 4 [Source:UniProtKB/Swiss-Prot;Acc:Q9FJ75]	22442269	22443437	43.28
AT5G55360	AT3	Probable long-chain-alcohol O-fatty-acyltransferase 3 [Source:UniProtKB/Swiss-Prot;Acc:Q9FJ74]	22443684	22444895	42.41
AT5G55370	AT2	Probable long-chain-alcohol O-fatty-acyltransferase 2 [Source:UniProtKB/Swiss-Prot;Acc:Q9FJ73]	22445069	22446133	42.07
AT5G55380	AT1	Probable long-chain-alcohol O-fatty-acyltransferase 1 [Source:UniProtKB/Swiss-Prot;Acc:Q9FJ72]	22446624	22447953	42.18
AT5G55390	EDM2	Protein ENHANCED DOWNY MILDEW 2 [Source:UniProtKB/Swiss-Prot;Acc:F4K3G5]	22447937	22454805	38.96
AT5G55400	FIM3	Fimbrin-3 [Source:UniProtKB/Swiss-Prot;Acc:Q9FJ70]	22455369	22459598	37.78
AT5G55410		Bifunctional inhibitor/lipid-transfer protein/seed storage 2S albumin superfamily protein [Source:UniProtKB/TrEMBL;Acc:Q9FJ69]	22460550	22461385	36.72
AT5G55430		Putative uncharacterized protein [Source:UniProtKB/TrEMBL;Acc:Q9FJ67]	22464589	22465227	43.97
AT5G55440		F-box protein, putative (DUF295) [Source:UniProtKB/TrEMBL;Acc:Q9FJ66]	22465693	22467209	43.11
AT5G55450		Bifunctional inhibitor/lipid-transfer protein/seed storage 2S albumin superfamily protein [Source:UniProtKB/TrEMBL;Acc:Q9FJ65]	22467337	22468306	39.07
AT5G08275			22467509	22468191	41.73
AT5G55460		At5g55460 [Source:UniProtKB/TrEMBL;Acc:Q8GWA4]	22468579	22469304	40.36
AT5G08285			22468929	22469238	41.61
AT5G55470	NHX4	Sodium/hydrogen exchanger 4 [Source:UniProtKB/Swiss-Prot;Acc:Q8S397]	22469245	22472706	37.12
AT5G55480	GDPDL4	Glycerophosphodiester phosphodiesterase GDPDL4 [Source:UniProtKB/Swiss-Prot;Acc:Q9FJ62]	22474142	22478216	39.95
AT5G55490	GEX1	Protein GAMETE EXPRESSED 1 [Source:UniProtKB/Swiss-Prot;Acc:Q681K7]	22478712	22481937	35.31
AT5G55500	XYLT	Glycosyltransferase (Fragment) [Source:UniProtKB/TrEMBL;Acc:W8PUA9]	22482125	22484631	42.32
AT5G55505		pre-tRNA [Source:TAIR;Acc:AT5G55505]	22484770	22484842	58.9
AT5G55507		At5g55507 [Source:UniProtKB/TrEMBL;Acc:Q84VG8]	22485099	22485712	39.09
AT5G55508		unknown protein; Ha. [Source:TAIR;Acc:AT5G55508]	22485872	22486015	33.33
AT5G55510	TIM22-3	Mitochondrial import inner membrane translocase subunit TIM22-3 [Source:UniProtKB/Swiss-Prot;Acc:Q6NKU9]	22486297	22488015	37.35
AT5G55520		CONTAINS InterPro DOMAIN/s: Kinesin-related protein (InterPro:IPR010544); BEST Arabidopsis thaliana protein match is: unknown protein (	22488054	22491300	39.51
AT5G55530		At5g55530 [Source:UniProtKB/TrEMBL;Acc:Q5HZ03]	22492892	22496064	38.51
AT5G55540	TRN1	Protein TORNADO 1 [Source:UniProtKB/Swiss-Prot;Acc:Q9FJ57]	22496196	22500714	42.77
AT5G55550		RNA-binding (RRM/RBD/RNP motifs) family protein [Source:TAIR;Acc:AT5G55550]	22501535	22504144	37.62
AT5G55560	WNK11	Probable serine/threonine-protein kinase WNK11 [Source:UniProtKB/Swiss-Prot;Acc:Q6ICW6]	22506216	22507879	41.41
AT5G55565		Defensin-like protein 41 [Source:UniProtKB/Swiss-Prot;Acc:Q3E8B0]	22510017	22513588	29.7
AT5G55570		unknown protein; LOCATED IN: chloroplast; Ha. [Source:TAIR;Acc:AT5G55570]	22514214	22515458	36.79
AT5G55580	MTERF9	Transcription termination factor MTERF9, chloroplastic [Source:UniProtKB/Swiss-Prot;Acc:Q9FM80]	22515391	22517964	38.31
AT5G55590	QRT1	Pectinesterase QRT1 [Source:UniProtKB/Swiss-Prot;Acc:Q9FM79]	22519654	22521858	38.82
AT5G55600		AT5g55600/MDF20_4 [Source:UniProtKB/TrEMBL;Acc:Q8W109]	22521843	22525685	39.99
AT5G55610		Isopentenyl-diphosphate delta-isomerase [Source:UniProtKB/TrEMBL;Acc:Q9FM77]	22525771	22528143	38.94
AT5G55620		At5g55620 [Source:UniProtKB/TrEMBL;Acc:Q8LC55]	22530007	22530987	33.23
AT5G55630	TPK1	Two-pore potassium channel 1 [Source:UniProtKB/Swiss-Prot;Acc:Q8LBL1]	22531358	22533122	40.4
AT5G55640		At5g55640 [Source:UniProtKB/TrEMBL;Acc:Q9FM74]	22533085	22534155	38.1
AT5G55650		Transmembrane protein [Source:UniProtKB/TrEMBL;Acc:Q9FM73]	22536072	22536976	34.48
AT5G55660		DEK domain-containing chromatin associated protein [Source:UniProtKB/TrEMBL;Acc:F4K4Y5]	22538556	22543474	38.28
AT5G55670		RNA-binding (RRM/RBD/RNP motifs) family protein [Source:UniProtKB/TrEMBL;Acc:Q9FM71]	22543728	22547062	43.63
AT5G55680		Glycine-rich protein [Source:UniProtKB/TrEMBL;Acc:Q9FM70]	22547445	22548679	45.59
AT5G55690		At5g55690 [Source:UniProtKB/TrEMBL;Acc:Q9FM69]	22548707	22550094	37.68
AT5G55700	BAM4	Inactive beta-amylase 4, chloroplastic [Source:UniProtKB/Swiss-Prot;Acc:Q9FM68]	22551734	22554964	38.75
AT5G55710	TIC20-V	Tic20-V [Source:UniProtKB/TrEMBL;Acc:A0A178URN8]	22554878	22555890	40.38
AT5G55720		Putative pectate lyase 21 [Source:UniProtKB/Swiss-Prot;Acc:Q9FM66]	22555995	22557725	37.72
AT5G55730	FLA1	Fasciclin-like arabinogalactan protein 1 [Source:UniProtKB/Swiss-Prot;Acc:Q9FM65]	22558044	22560541	38.55
AT5G55740	CRR21	Pentatricopeptide repeat-containing protein At5g55740, chloroplastic [Source:UniProtKB/Swiss-Prot;Acc:Q9FM64]	22561849	22564587	39.5
AT5G55750		Hydroxyproline-rich glycoprotein family protein [Source:UniProtKB/TrEMBL;Acc:Q9FM63]	22564801	22566021	41.28
AT5G55760	SRT1	SRT1 [Source:UniProtKB/TrEMBL;Acc:A0A178UR49]	22567222	22571008	37.18
AT5G55770		Cysteine/Histidine-rich C1 domain family protein [Source:UniProtKB/TrEMBL;Acc:Q9FM62]	22571645	22573732	41.04
AT5G55780		Cysteine/Histidine-rich C1 domain family protein [Source:UniProtKB/TrEMBL;Acc:Q9FM61]	22576493	22578682	40.59
AT5G55790		At5g55790 [Source:UniProtKB/TrEMBL;Acc:Q9FM60]	22578715	22581335	33.08
AT5G55800		Cysteine/Histidine-rich C1 domain family protein [Source:UniProtKB/TrEMBL;Acc:Q9FM59]	22581694	22583430	40.88
AT5G55810	AtNMNAT	Nicotinate/nicotinamide mononucleotide adenyltransferase [Source:UniProtKB/TrEMBL;Acc:F4K688]	22583972	22586405	35.54
AT5G55820		Inner centromere protein, ARK-binding region protein [Source:UniProtKB/TrEMBL;Acc:Q9FM57]	22586287	22594135	38.99
AT5G55830	LECRKS7	Probable L-type lectin-domain containing receptor kinase S.7 [Source:UniProtKB/Swiss-Prot;Acc:Q9FHG4]	22594617	22596843	41.22
AT5G55835	MIR156H	MIR156H; miRNA [Source:TAIR;Acc:AT5G55835]	22597012	22597117	44.34
AT5G55840		Pentatricopeptide repeat (PPR) superfamily protein [Source:TAIR;Acc:AT5G55840]	22597993	22602760	39.2
AT5G55850	NOI	RPM1-interacting protein 4 (RIN4) family protein [Source:UniProtKB/TrEMBL;Acc:F4K692]	22603302	22605300	33.92
AT5G55855	SUMO7	Putative small ubiquitin-related modifier 7 [Source:UniProtKB/Swiss-Prot;Acc:Q3E8A8]	22605805	22606634	30.96
AT5G55856	SUMO8	Putative small ubiquitin-related modifier 8 [Source:UniProtKB/Swiss-Prot;Acc:B3H5R8]	22607927	22608640	31.79
AT5G55860		WEB family protein At5g55860 [Source:UniProtKB/Swiss-Prot;Acc:Q9LVQ4]	22609433	22612486	39.29
AT5G55870		Putative uncharacterized protein [Source:UniProtKB/TrEMBL;Acc:Q9LVQ3]	22612637	22613311	37.78
AT5G55880		Putative uncharacterized protein [Source:UniProtKB/TrEMBL;Acc:Q9LDH8]	22619104	22620204	45.41
AT5G08375			22622311	22622579	38.66
AT5G55890		Putative uncharacterized protein [Source:UniProtKB/TrEMBL;Acc:Q9LDH8]	22622737	22623837	45.41
AT5G55893		unknown protein; Ha. [Source:TAIR;Acc:AT5G55893]	22624738	22626570	45.83
AT5G08385			22634685	22635601	37.3
AT5G08395			22634942	22635453	38.67
AT5G55900		At5g55900 [Source:UniProtKB/TrEMBL;Acc:Q9FG75]	22637510	22639828	39.24
AT5G55910	D6PK	Serine/threonine-protein kinase D6PK [Source:UniProtKB/Swiss-Prot;Acc:Q9FG74]	22639813	22642414	40.01
AT5G55920	OLI2	Nucleolar protein-like [Source:UniProtKB/TrEMBL;Acc:Q9FG73]	22645448	22649517	37.99
AT5G55930	OPT1	Oligopeptide transporter 1 [Source:UniProtKB/Swiss-Prot;Acc:Q9FG72]	22652715	22656106	41.1
AT5G08425			22655538	22655817	49.29
AT5G55940	EMB2731	ER membrane protein complex subunit 8/9 homolog [Source:UniProtKB/Swiss-Prot;Acc:Q9FG71]	22655998	22658084	36.85
AT5G55950		Nucleotide-sugar uncharacterized transporter 2 [Source:UniProtKB/Swiss-Prot;Acc:Q9FG70]	22658112	22660505	36.84
AT5G55960		Transmembrane protein C9orf5 protein [Source:UniProtKB/TrEMBL;Acc:Q93XX1]	22662532	22665099	40.23
AT5G55970		RING/U-box superfamily protein [Source:UniProtKB/TrEMBL;Acc:Q8LES9]	22667220	22669631	37.02
AT5G55980		Serine-rich protein-like protein [Source:UniProtKB/TrEMBL;Acc:Q9FKU6]	22670186	22670893	37.71
AT5G55990	CBL2	Calcineurin B-like protein 2 [Source:UniProtKB/Swiss-Prot;Acc:Q8LAS7]	22671113	22674100	33.97
AT5G56000	HSP90-4	Hsp81.4 [Source:UniProtKB/TrEMBL;Acc:A0A178UQ52]	22677338	22680152	42.56
AT5G56010	HSP90-3	Heat shock protein 90-3 [Source:UniProtKB/Swiss-Prot;Acc:P51818]	22681238	22684252	42.02
AT5G56020		Vesicle transport protein [Source:UniProtKB/TrEMBL;Acc:Q9FKU4]	22684386	22686466	38.25
AT5G56030	HSP81-2	Heat shock protein 81-2 [Source:UniProtKB/TrEMBL;Acc:F4K6B6]	22686473	22689671	41.89
AT5G56040		Leucine-rich receptor-like protein kinase family protein [Source:UniProtKB/TrEMBL;Acc:F4K6B8]	22694823	22698645	43.42
AT5G56050		Late embryogenesis abundant (LEA) hydroxyproline-rich glycoprotein family protein [Source:UniProtKB/TrEMBL;Acc:Q9FKU2]	22700919	22702200	39.39
AT5G56060		Domain of unknown function (DUF2431) [Source:TAIR;Acc:AT5G56060]	22704223	22704892	38.06
AT5G56070		Putative uncharacterized protein [Source:UniProtKB/TrEMBL;Acc:Q9FKU0]	22708679	22709204	38.02
AT5G56075		Domain of unknown function (DUF2431) [Source:TAIR;Acc:AT5G56075]	22709756	22710809	37.1
AT5G56080	NAS2	NAS2 [Source:UniProtKB/TrEMBL;Acc:A0A178ULG9]	22711141	22712441	40.58
AT5G56090	COX15	Cytochrome c oxidase assembly protein COX15 [Source:UniProtKB/Swiss-Prot;Acc:Q9FKT8]	22714471	22716997	39.89
AT5G56100		Glycine-rich protein / oleosin [Source:UniProtKB/TrEMBL;Acc:Q9FKT7]	22716917	22717849	40.62
AT5G56110	MYB80	MYB80 [Source:UniProtKB/TrEMBL;Acc:A0A178UM75]	22719191	22720664	39.21
AT5G56120		At5g56120 [Source:UniProtKB/TrEMBL;Acc:Q6NPP2]	22721373	22722613	38.76
AT5G56130	THO3	THO complex subunit 3 [Source:UniProtKB/Swiss-Prot;Acc:Q9FKT5]	22722482	22725099	36.97
AT5G56140		KH domain-containing protein At5g56140 [Source:UniProtKB/Swiss-Prot;Acc:Q9FKT4]	22725238	22728473	36.84
AT5G56150	UBC30	UBC30 [Source:UniProtKB/TrEMBL;Acc:A0A178UMN8]	22729015	22731315	37.16
AT5G56160	SFH14	Phosphatidylinositol/phosphatidylcholine transfer protein SFH14 [Source:UniProtKB/Swiss-Prot;Acc:F4K6D3]	22731949	22735612	36.57
AT5G56170	LLG1	LLG1 [Source:UniProtKB/TrEMBL;Acc:A0A178USL6]	22735899	22737339	37.68
AT5G56180	ARP8	Actin-related protein 8 [Source:UniProtKB/Swiss-Prot;Acc:Q9FKT0]	22737325	22741100	38.35
AT5G56190		Transducin/WD40 repeat-like superfamily protein [Source:TAIR;Acc:AT5G56190]	22741965	22745191	37.47
AT5G56200		C2H2 type zinc finger transcription factor family [Source:UniProtKB/TrEMBL;Acc:Q9FH19]	22747478	22749427	39.13
AT5G56210	WIP2	WPP domain-interacting protein 2 [Source:UniProtKB/Swiss-Prot;Acc:Q9FH18]	22750462	22752817	38.92
AT5G08435			22752876	22753197	35.71
AT5G56220		P-loop containing nucleoside triphosphate hydrolases superfamily protein [Source:UniProtKB/TrEMBL;Acc:Q9FH17]	22753888	22758057	40.55
AT5G08445			22753926	22754558	34.6
AT5G56230	PRA1G2	PRA1 family protein G2 [Source:UniProtKB/Swiss-Prot;Acc:Q9FH16]	22758544	22759556	40.18

AT5G56240		BEST Arabidopsis thaliana protein match is: hapless 8 (TAIR:AT5G56250.1); Ha. [Source:TAIR;Acc:AT5G56240]	22759663	22764587	37.89
AT5G56250	HAP8	Hapless 8 [Source:UniProtKB/TrEMBL;Acc:Q9FH14]	22768453	22773021	36.29
AT5G08455			22773003	22773204	34.16
AT5G56260		Ribonuclease E inhibitor RraA/Dimethylmenaquinone methyltransferase [Source:TAIR;Acc:AT5G56260]	22775189	22777355	37.06
AT5G56270	WRKY2	Probable WRKY transcription factor 2 [Source:UniProtKB/Swiss-Prot;Acc:Q9FG77]	22779614	22783426	39.52
AT5G56280	CSN6A	COP9 signalosome complex subunit 6a [Source:UniProtKB/Swiss-Prot;Acc:Q8W206]	22783384	22785605	37.13
AT5G56290	PEX5	Peroxisome biogenesis protein 5 [Source:UniProtKB/Swiss-Prot;Acc:Q9FMA3]	22786496	22792306	38.58
AT5G08465			22792730	22793272	34.62
AT5G08475			22792840	22793079	37.92
AT5G56300	GAMT2	Gibberellic acid methyltransferase 2 [Source:UniProtKB/Swiss-Prot;Acc:Q5XF78]	22799507	22801692	36.14
AT5G56310	PCMP-E13	Pentatricopeptide repeat-containing protein At5g56310 [Source:UniProtKB/Swiss-Prot;Acc:Q9FMA1]	22802299	22804377	41.17
AT5G56320	ATEXPA14	expansin A14 [Source:TAIR;Acc:AT5G56320]	22808641	22810747	36.97
AT5G08485			22811476	22811970	38.38
AT5G56325		FBD-like domain family protein [Source:UniProtKB/TrEMBL;Acc:Q1PDI7]	22812213	22813263	36.73
AT5G56330	ATACA8	alpha carbonic anhydrase 8 [Source:TAIR;Acc:AT5G56330]	22813750	22816256	34.58
AT5G56340	ATCRT1	RING/U-box superfamily protein [Source:UniProtKB/TrEMBL;Acc:Q9FM98]	22817480	22819730	36.92
AT5G56350		Pyruvate kinase [Source:UniProtKB/TrEMBL;Acc:Q9FM97]	22819936	22822904	39.81
AT5G56360	PSL4	Glucosidase 2 subunit beta [Source:UniProtKB/Swiss-Prot;Acc:Q9FM96]	22823189	22828062	37.03
AT5G56365		pre-tRNA [Source:TAIR;Acc:AT5G56365]	22828131	22828204	55.41
AT5G08495			22828549	22828703	41.94
AT5G08515			22828809	22828897	37.08
AT5G08525			22828963	22829045	37.35
AT5G56368		Putative defensin-like protein 282 [Source:UniProtKB/Swiss-Prot;Acc:Q2V2Y0]	22833964	22834645	35.19
AT5G56369		Putative defensin-like protein 283 [Source:UniProtKB/Swiss-Prot;Acc:Q2L6T1]	22835392	22835930	37.48
AT5G56370		FBD-associated F-box protein At5g56370 [Source:UniProtKB/Swiss-Prot;Acc:Q9FM94]	22835931	22837972	38.54
AT5G56380		FBD-associated F-box protein At5g56380 [Source:UniProtKB/Swiss-Prot;Acc:Q9FM93]	22838633	22841117	38.87
AT5G56390		Putative FBD-associated F-box protein At5g56390 [Source:UniProtKB/Swiss-Prot;Acc:Q9FM92]	22842790	22844273	38.48
AT5G56400		Putative FBD-associated F-box protein At5g56400 [Source:UniProtKB/Swiss-Prot;Acc:Q9FM91]	22846069	22847615	38.4
AT5G56410		Putative FBD-associated F-box protein At5g56410 [Source:UniProtKB/Swiss-Prot;Acc:Q9FM90]	22848508	22850331	37.45
AT5G56420		F-box/FBD/LRR-repeat protein At5g56420 [Source:UniProtKB/Swiss-Prot;Acc:Q9FM89]	22850584	22852753	37.65
AT5G56430		Putative FBD-associated F-box protein At5g56430 [Source:UniProtKB/Swiss-Prot;Acc:Q9FM88]	22853323	22854801	38.54
AT5G56440		Putative FBD-associated F-box protein At5g56440 [Source:UniProtKB/Swiss-Prot;Acc:Q9FM87]	22855742	22857117	40.84
AT5G56450		Probable ADP,ATP carrier protein At5g56450 [Source:UniProtKB/Swiss-Prot;Acc:Q9FM86]	22858509	22860071	39.48
AT5G56452		FBD-like domain family protein [Source:UniProtKB/TrEMBL;Acc:Q1G371]	22860450	22861818	37.4
AT5G56460	PBL16	Probable serine/threonine-protein kinase PBL16 [Source:UniProtKB/Swiss-Prot;Acc:Q9FM85]	22865074	22868036	36.52
AT5G56470	GULLO7	Probable truncated L-gulonolactone oxidase 7, mitochondrial [Source:UniProtKB/Swiss-Prot;Acc:Q9FM84]	22869060	22869958	41.49
AT5G56480		Bifunctional inhibitor/lipid-transfer protein/seed storage 2S albumin superfamily protein [Source:UniProtKB/TrEMBL;Acc:Q9FM83]	22870259	22870817	38.64
AT5G56490	GULLO4	Probable L-gulonolactone oxidase 4 [Source:UniProtKB/Swiss-Prot;Acc:Q9FM82]	22871463	22873501	41.59
AT5G56500	CPN60B3	Chaperonin 60 subunit beta 3, chloroplastic [Source:UniProtKB/Swiss-Prot;Acc:C0Z361]	22873666	22877407	37.81
AT5G56510	APUM12	Pumilio homolog 12 [Source:UniProtKB/Swiss-Prot;Acc:Q9LVC3]	22880687	22884087	36.4
AT5G08575			22880926	22881124	37.69
AT5G56520		At5g56520 [Source:UniProtKB/TrEMBL;Acc:Q8L9N8]	22885143	22886600	35.46
AT5G08585			22887447	22887656	42.86
AT5G56530		Similarity to carboxyl-terminal proteinase [Source:UniProtKB/TrEMBL;Acc:Q9LVC1]	22889073	22891866	38.05
AT5G56540	AGP14	Arabinogalactan peptide 14 [Source:UniProtKB/Swiss-Prot;Acc:Q9LVC0]	22893151	22893651	35.33
AT5G56550	OXS3	Emb [Source:UniProtKB/TrEMBL;Acc:Q9LVB9]	22895616	22896734	33.78
AT5G08595			22898420	22898747	32.93
AT5G56555			22898714	22899064	34.47
AT5G56560		FBD, F-box and Leucine Rich Repeat domains containing protein [Source:TAIR;Acc:AT5G56560]	22899724	22901985	37.8
AT5G56570		F-box/FBD/LRR-repeat protein At5g56570 [Source:UniProtKB/Swiss-Prot;Acc:Q9FJV1]	22902736	22904596	37.24
AT5G56580	MKK6	Mitogen-activated protein kinase kinase 6 [Source:UniProtKB/Swiss-Prot;Acc:Q9FJV0]	22904622	22906764	36.72
AT5G56590		Glucan endo-1,3-beta-glucosidase 13 [Source:UniProtKB/Swiss-Prot;Acc:Q9FJU9]	22907264	22909707	39.77
AT5G56600	PRO5	Profilin-5 [Source:UniProtKB/Swiss-Prot;Acc:Q9FE63]	22909882	22910710	39.81
AT5G56610		At5g56610 [Source:UniProtKB/TrEMBL;Acc:Q6NKR2]	22916692	22918468	37.65
AT5G56620	anac099	NAC domain containing protein 99 [Source:TAIR;Acc:AT5G56620]	22917934	22921499	32.87
AT5G08605			22921496	22921738	28.81
AT5G56630	PFK7	ATP-dependent 6-phosphofructokinase 7 [Source:UniProtKB/Swiss-Prot;Acc:Q9C5J7]	22923804	22926963	37.47
AT5G56640	MIOX5	Inositol oxygenase 5 [Source:UniProtKB/Swiss-Prot;Acc:Q9FJU4]	22927230	22929717	31.59
AT5G56650	ILL1	At5g56650 [Source:UniProtKB/TrEMBL;Acc:Q67YZ8]	22930775	22932822	40.92
AT5G56660	ILL2	At5g56660 [Source:UniProtKB/TrEMBL;Acc:Q5HZ30]	22933267	22935316	41.66
AT5G08615			22933439	22933634	48.47
AT5G56670	RPS30A	40S ribosomal protein S30 [Source:UniProtKB/Swiss-Prot;Acc:P49689]	22935211	22936084	36.27
AT5G56680	SYNC1	Asparagine--tRNA ligase, cytoplasmic 1 [Source:UniProtKB/Swiss-Prot;Acc:Q9SW96]	22936534	22939060	40.96
AT5G56690		Putative FBD-associated F-box protein At5g56690 [Source:UniProtKB/Swiss-Prot;Acc:Q9FJU3]	22939723	22941099	37.91
AT5G56700		FBD / Leucine Rich Repeat domains containing protein [Source:TAIR;Acc:AT5G56700]	22941750	22943587	38.68
AT5G56710	RPL31C	60S ribosomal protein L31-3 [Source:UniProtKB/Swiss-Prot;Acc:P51420]	22943707	22944993	36.29
AT5G56720		Malate dehydrogenase [Source:UniProtKB/TrEMBL;Acc:A0A178UAN5]	22945419	22946807	39.52
AT5G56730		Insulinase (Peptidase family M16) protein [Source:TAIR;Acc:AT5G56730]	22946906	22952599	38.95
AT5G56740	HAG2	Histone acetyltransferase type B catalytic subunit [Source:UniProtKB/TrEMBL;Acc:Q1JPN3]	22952767	22955739	39.22
AT5G56745		pre-tRNA [Source:TAIR;Acc:AT5G56745]	22955841	22955912	58.33
AT5G56750	NDL1	Protein NDL1 [Source:UniProtKB/Swiss-Prot;Acc:Q9FJT7]	22957629	22960916	36.19
AT5G56760	SAT5	Serine acetyltransferase 5 [Source:UniProtKB/Swiss-Prot;Acc:Q42538]	22961229	22962713	42.69
AT5G56770	ET3	Protein EFFECTOR OF TRANSCRIPTION 3 [Source:UniProtKB/Swiss-Prot;Acc:Q9FJT6]	22962994	22963989	40.46
AT5G56780	ET2	ET2 [Source:UniProtKB/TrEMBL;Acc:A0A178URY3]	22964233	22966398	40.35
AT5G56790		Protein kinase superfamily protein [Source:UniProtKB/TrEMBL;Acc:Q8H0Z8]	22967891	22971689	37.69
AT5G56795	MT1B	MT1B [Source:UniProtKB/TrEMBL;Acc:A0A178UJC4]	22971713	22972690	35.28
AT5G56800		Emb [Source:UniProtKB/TrEMBL;Acc:Q9FJT3]	22972989	22974308	36.44
AT5G56810		Putative F-box/FBD/LRR-repeat protein At5g56810 [Source:UniProtKB/Swiss-Prot;Acc:Q9FJT2]	22974363	22976112	36.91
AT5G56820		Putative FBD-associated F-box protein At5g56820 [Source:UniProtKB/Swiss-Prot;Acc:Q9FJT1]	22976334	22977838	39
AT5G56840		At5g56840 [Source:UniProtKB/TrEMBL;Acc:Q9FJS9]	22980638	22982425	35.57
AT5G56850		unknown protein; EXPRESSED IN: 21 plant structures; EXPRESSED DURING: 13 growth stages; Ha. [Source:TAIR;Acc:AT5G56850]	22985115	22988110	35.88
AT5G56860	GATA21	GATA transcription factor 21 [Source:UniProtKB/Swiss-Prot;Acc:Q5HZ36]	22989216	22991530	35.94
AT5G08645			22992866	22993188	39.01
AT5G08635			22992866	22993341	33.82
AT5G08655			22994445	22994649	40.98
AT5G08665			22994477	22994725	39.76
AT5G56870	BGAL4	Beta-galactosidase 4 [Source:UniProtKB/Swiss-Prot;Acc:Q9SCV8]	23003909	23009672	35.91
AT5G56880		At5g56880 [Source:UniProtKB/TrEMBL;Acc:Q9LV38]	23008764	23009709	40.38
AT5G56890		Protein kinase superfamily protein [Source:UniProtKB/TrEMBL;Acc:F4K946]	23010423	23016015	41.61
AT5G56900		Zinc finger CCCH domain-containing protein 64 [Source:UniProtKB/Swiss-Prot;Acc:Q84WU9]	23019245	23022765	38.48
AT5G56910		Proteinase inhibitor I25, cystatin, conserved region [Source:UniProtKB/TrEMBL;Acc:Q9LTS9]	23022862	23024257	38.32
AT5G56920		Cystatin/monellin superfamily protein [Source:UniProtKB/TrEMBL;Acc:Q9LTS8]	23024896	23025915	41.37
AT5G56930	EMB1789	EMB1789 [Source:UniProtKB/TrEMBL;Acc:A0A178UTA2]	23027658	23030639	39.37
AT5G56940	RPS16-2	30S ribosomal protein S16-2, chloroplastic/mitochondrial [Source:UniProtKB/Swiss-Prot;Acc:Q9LTS6]	23030747	23032411	37.36
AT5G56950	NAP1;3	Nucleosome assembly protein 1;3 [Source:UniProtKB/Swiss-Prot;Acc:Q94K07]	23032449	23035693	35.69
AT5G56960		basic helix-loop-helix (bHLH) DNA-binding family protein [Source:TAIR;Acc:AT5G56960]	23038389	23041617	35.8
AT5G08675			23041533	23041839	30.62
AT5G56970	CKX3	Cytokinin dehydrogenase 3 [Source:UniProtKB/Swiss-Prot;Acc:Q9LTS3]	23044839	23048292	36.19
AT5G08685			23054466	23054677	41.51
AT5G56975		pre-tRNA [Source:TAIR;Acc:AT5G56975]	23054771	23054844	58.11
AT5G56980	A70	unknown protein; FUNCTIONS IN: molecular_function unknown; INVOLVED IN: biological_process unknown; LOCATED IN: endomembrane	23056080	23057742	43.6
AT5G56985			23056476	23056904	49.65
AT5G56990		BEST Arabidopsis thaliana protein match is: Cystatin/monellin superfamily protein (TAIR:AT5G56920.1); Ha. [Source:TAIR;Acc:AT5G56990]	23060833	23062400	39.48
AT5G57000		At5g57000 [Source:UniProtKB/TrEMBL;Acc:Q9LTS1]	23063576	23065350	35.61
AT5G57010	IQM5	IQ domain-containing protein IQM5 [Source:UniProtKB/Swiss-Prot;Acc:Q058N0]	23067828	23070431	36.87

	AT5G57015	CKL12	Ckl12 [Source:UniProtKB/TrEMBL;Acc:A0A178UEU7]	23071028	23074843	36.48
	AT5G57020	NMT1	Glycylpeptide N-tetradecanoyltransferase 1 [Source:UniProtKB/Swiss-Prot;Acc:Q9LTR9]	23074867	23077193	42.11
	AT5G57030	LUT2	Lycopene epsilon cyclase, chloroplastic [Source:UniProtKB/Swiss-Prot;Acc:Q38932]	23077120	23080096	38.43
	AT5G57035		U-box domain-containing protein kinase family protein [Source:UniProtKB/TrEMBL;Acc:F4K964]	23080647	23083988	39.68
	AT5G57040		At5g57040 [Source:UniProtKB/TrEMBL;Acc:Q9LTR8]	23083898	23085246	36.62
	AT5G57050	ABI2	AtABI2 [Source:UniProtKB/TrEMBL;Acc:A0A178UGB7]	23087122	23089631	38.33
	AT5G57060		60S ribosomal L18a-like protein [Source:UniProtKB/TrEMBL;Acc:Q9LTR7]	23089909	23092224	34.46
	AT5G57070		Hydroxyproline-rich glycoprotein family protein [Source:UniProtKB/TrEMBL;Acc:F4KAI4]	23096024	23098602	44.67
	AT5G57080		Putative uncharacterized protein [Source:UniProtKB/TrEMBL;Acc:Q9LU78]	23098872	23099587	37.71
	AT5G57090	PIN2	WAV6 [Source:UniProtKB/TrEMBL;Acc:A0A384LBE9]	23100516	23105228	35.07
	AT5G57100		Nucleotide-sugar uncharacterized transporter 1 [Source:UniProtKB/Swiss-Prot;Acc:Q9LU76]	23106298	23108883	37.55
	AT5G57110	ACA8	Calcium-transporting ATPase [Source:UniProtKB/TrEMBL;Acc:Q0WV19]	23109438	23117686	37.69
	AT5G57120		AT5g57120/MUL3_6 [Source:UniProtKB/TrEMBL;Acc:Q9LU74]	23122488	23124594	38.73
	AT5G57123		At5g57123 [Source:UniProtKB/TrEMBL;Acc:Q8LCB5]	23126874	23127724	33.49
	AT5G08705			23133765	23133997	36.48
	AT5G57130	SMXL5	SMXL5 [Source:UniProtKB/TrEMBL;Acc:A0A178UNM9]	23144947	23149615	40.01
	AT5G57140	PAP28	Probable inactive purple acid phosphatase 28 [Source:UniProtKB/Swiss-Prot;Acc:Q9LU72]	23149880	23151677	38.26
	AT5G57150		Basic helix-loop-helix (BHLH) DNA-binding superfamily protein [Source:UniProtKB/TrEMBL;Acc:F4KAJ5]	23152174	23155066	32.22
	AT5G57160	LIG4	DNA ligase 4 [Source:UniProtKB/Swiss-Prot;Acc:Q9LL84]	23154965	23161826	39.27
	AT5G57170		Tautomerase/MIF superfamily protein [Source:UniProtKB/TrEMBL;Acc:F4KAK0]	23161877	23163290	35.64
	AT5G57180	CIA2	CIA2 [Source:UniProtKB/TrEMBL;Acc:A0A178UDY1]	23167774	23171025	35.89
	AT5G57190	PSD2	Phosphatidylserine decarboxylase proenzyme 2 [Source:UniProtKB/TrEMBL;Acc:A0A178UED8]	23171049	23175495	36.86
	AT5G57200		Putative clathrin assembly protein At5g57200 [Source:UniProtKB/Swiss-Prot;Acc:Q9LVD8]	23177525	23180853	36.98
	AT5G57210		AT5g57210/MJB24_2 [Source:UniProtKB/TrEMBL;Acc:Q9LVD7]	23180833	23184053	40.02
	AT5G57220	CYP81F2	Cytochrome P450 81F2 [Source:UniProtKB/Swiss-Prot;Acc:Q9LVD6]	23187840	23189902	39.65
	AT5G57230		Thioredoxin superfamily protein [Source:TAIR;Acc:AT5G57230]	23189938	23191714	35.96
	AT5G57240	ORP4C	ORP4C [Source:UniProtKB/TrEMBL;Acc:A0A384LHK8]	23192872	23195476	36.89
	AT5G57250		Pentatricopeptide repeat-containing protein At5g57250, mitochondrial [Source:UniProtKB/Swiss-Prot;Acc:Q9LVD3]	23194833	23198609	40.51
	AT5G57260	CYP71B10	CYP71B10 [Source:UniProtKB/TrEMBL;Acc:A0A178UG51]	23198589	23200318	39.36
	AT5G57270		At5g57270 [Source:UniProtKB/TrEMBL;Acc:Q8W4D1]	23200591	23203908	36.86
	AT5G57280	RID2	RID2 [Source:UniProtKB/TrEMBL;Acc:A0A178UNY0]	23204377	23206663	37.17
	AT5G57290	RPP3B	60S acidic ribosomal protein P3-2 [Source:UniProtKB/Swiss-Prot;Acc:Q9LVC9]	23206696	23207961	36.81
	AT5G57300	COQ5	2-methoxy-6-polyprenyl-1,4-benzoquinol methylase, mitochondrial [Source:UniProtKB/TrEMBL;Acc:A0A178U865]	23208463	23211139	37.17
	AT5G57320	VLN5	Villin-5 [Source:UniProtKB/Swiss-Prot;Acc:Q9LVC6]	23211952	23218041	38.01
	AT5G57330		Glucose-6-phosphate 1-epimerase [Source:UniProtKB/TrEMBL;Acc:Q9LVC5]	23218086	23221089	36.75
	AT5G57340		Ras guanine nucleotide exchange factor Q-like protein [Source:UniProtKB/TrEMBL;Acc:Q9LVC4]	23225951	23229298	31.51
	AT5G57345		At5g57345 [Source:UniProtKB/TrEMBL;Acc:Q8LAD4]	23229291	23230749	37.56
	AT5G57350	AHA3	Plasma membrane ATPase [Source:UniProtKB/TrEMBL;Acc:A0A178UHY0]	23230874	23236682	37.11
	AT5G57360	ZTL	Galactose oxidase/kelch repeat superfamily protein [Source:UniProtKB/TrEMBL;Acc:F4KAN2]	23241320	23244590	43.81
	AT5G57370		U4/U6.U5 small nuclear ribonucleoprotein [Source:UniProtKB/TrEMBL;Acc:Q9FIE4]	23244610	23246420	42.19
	AT5G57380	VIN3	VIN3 [Source:UniProtKB/TrEMBL;Acc:A0A178UF19]	23246395	23249504	38.78
	AT5G57390	AIL5	AP2-like ethylene-responsive transcription factor AIL5 [Source:UniProtKB/Swiss-Prot;Acc:Q6PQQ3]	23252989	23256703	37.2
	AT5G57400		Transmembrane protein [Source:UniProtKB/TrEMBL;Acc:Q5BPG1]	23261985	23263479	33.71
	AT5G57410		Afadin/alpha-actinin-binding protein [Source:UniProtKB/TrEMBL;Acc:F4KAN9]	23264314	23267905	36.89
	AT5G57420	IAA33	Auxin-responsive protein IAA33 [Source:UniProtKB/Swiss-Prot;Acc:Q9FKM7]	23269914	23271092	38.51
	AT5G57440	GPP2, GS1	(DL)-glycerol-3-phosphatase 2 [Source:UniProtKB/Swiss-Prot;Acc:Q8VZP1]	23271114	23273021	38.89
	AT5G57450	XRCC3	DNA repair protein XRCC3 homolog [Source:UniProtKB/Swiss-Prot;Acc:Q9FKM5]	23273067	23274976	39.16
	AT5G57460		At5g57460 [Source:UniProtKB/TrEMBL;Acc:Q9FKM4]	23275110	23277488	43.09
	AT5G57480		AAA-ATPase At5g57480 [Source:UniProtKB/Swiss-Prot;Acc:Q9FKM3]	23279139	23281271	39.33
	AT5G57490	VDAC4	Mitochondrial outer membrane protein porin 4 [Source:UniProtKB/Swiss-Prot;Acc:Q9FKM2]	23283617	23286005	38.93
	AT5G57500		Hexosyltransferase [Source:UniProtKB/TrEMBL;Acc:Q9FKM1]	23286197	23287739	36.29
	AT5G57510		Cotton fiber protein [Source:UniProtKB/TrEMBL;Acc:Q9FKM0]	23288885	23289635	34.89
	AT5G57520	ZFP2	Zinc finger protein 2 [Source:UniProtKB/Swiss-Prot;Acc:Q39261]	23295652	23296776	38.04
	AT5G57530	XTH12	Probable xyloglucan endotransglucosylase/hydrolase protein 12 [Source:UniProtKB/Swiss-Prot;Acc:Q9FKL9]	23300321	23301689	40.69
	AT5G57535		unknown protein; Ha. [Source:TAIR;Acc:AT5G57535]	23302415	23302549	40
	AT5G57540	XTH13	Putative xyloglucan endotransglucosylase/hydrolase protein 13 [Source:UniProtKB/Swiss-Prot;Acc:Q9FKL8]	23302787	23304095	39.42
MQTL-9/Chr5	AT5G63145		pre-tRNA [Source:TAIR;Acc:AT5G63145]	25330036	25330107	54.17
	AT5G64505		pre-tRNA [Source:TAIR;Acc:AT5G64505]	25784115	25784188	54.05
	AT5G64735		pre-tRNA [Source:TAIR;Acc:AT5G64735]	25875394	25875465	55.56
	AT5G62848	MIR420	MIR420; miRNA [Source:TAIR;Acc:AT5G62848]	25230568	25230667	28
	AT5G62162	MIR399C	MIR399C; miRNA [Source:TAIR;Acc:AT5G62162]	24962485	24962598	45.61
	AT5G63715	MIR166G	MIR166/MIR166G; miRNA [Source:TAIR;Acc:AT5G63715]	25504798	25504919	43.44
	AT5G09130			25290992	25291043	26.92
	AT5G09020			24782700	24782799	45
	AT5G09000			24709074	24709141	32.35
	AT5G09005			24709217	24709297	38.27
	AT5G09010			24730635	24730829	28.21
	AT5G09140			25296826	25296926	33.66
	AT5G61455	U2.7	U2.7; snRNA [Source:TAIR;Acc:AT5G61455]	24713264	24714094	37.91
	AT5G09080			25083791	25083985	28.21
	AT5G61345		pre-tRNA [Source:TAIR;Acc:AT5G61345]	24667282	24667353	62.5
	AT5G60966		pre-tRNA [Source:TAIR;Acc:AT5G60966]	24531667	24531738	61.11
	AT5G61445		pre-tRNA [Source:TAIR;Acc:AT5G61445]	24709666	24709738	58.9
	AT5G61835		pre-tRNA [Source:TAIR;Acc:AT5G61835]	24838980	24839064	50.59
	AT5G09150			25370434	25370870	42.79
	AT5G08990			24624933	24625189	36.96
	AT5G09175			25616392	25618826	38.93
	AT5G09135			25293451	25293694	34.84
	AT5G09275			25913347	25913937	39.42
	AT5G09160			25420555	25420756	42.57
	AT5G09120			25284023	25284489	40.04
	AT5G09155			25374353	25375247	41.9
	AT5G09030			24901362	24903100	40.25
	AT5G64572		other RNA [Source:TAIR;Acc:AT5G64572]	25810298	25811733	46.94
	AT5G63195		other RNA [Source:TAIR;Acc:AT5G63195]	25346729	25348386	43.24
	AT5G09185			25670435	25670699	50.57
	AT5G09190			25771560	25771845	37.06
	AT5G09205			25794445	25794743	35.79
	AT5G09025			24801000	24801346	29.68
	AT5G09235			25858101	25858537	39.36
	AT5G09215			25857698	25858537	37.26
	AT5G09070			25066296	25066564	37.92
	AT5G09015			24747856	24748067	33.49
	AT5G09035			24905537	24905816	32.14
	AT5G09050			24993776	24994011	42.37
	AT5G09110			25274466	25274705	48.75
	AT5G09145			25303491	25303728	43.7
	AT5G09090			25239514	25240235	36.01
	AT5G09105			25274429	25274946	39.38
	AT5G09165			25422276	25422517	35.95
	AT5G09100			25273909	25274156	34.68
	AT5G09045			24909440	24909720	40.21
	AT5G09265			25906524	25907000	28.51
	AT5G09065			25066226	25067028	38.98
	AT5G09255			25890519	25890874	42.13

AT5G09125			25290913	25291912	34.1
AT5G09085			25158096	25158546	31.26
AT5G09200			25794376	25794789	33.57
AT5G09095			25266183	25266460	38.85
AT5G09040			24908742	24909077	36.31
AT5G09245			25866998	25867205	33.65
AT5G09060			25046174	25046741	40.67
AT5G09055			25035668	25036126	36.6
AT5G08985			24541981	24542224	35.66
AT5G08995			24699414	24699617	31.37
AT5G09195			25786692	25787325	37.85
AT5G09170			25436917	25437296	47.63
AT5G61800	PCMP-E8	Pentatricopeptide repeat-containing protein At5g61800 [Source:UniProtKB/Swiss-Prot;Acc:Q9FLS9]	24829987	24831615	41.07
AT5G64170	LNK1	Protein LNK1 [Source:UniProtKB/Swiss-Prot;Acc:A8MQN2]	25672466	25676823	37.72
AT5G64310	AGP1	Classical arabinogalactan protein 1 [Source:UniProtKB/Swiss-Prot;Acc:Q8LCN5]	25721733	25722678	41.33
AT5G64667	IDL2	IDL2 [Source:UniProtKB/TrEMBL;Acc:A0A178U8Z6]	25850509	25851375	34.37
AT5G63900		Acyl-CoA N-acyltransferase with RING/FYVE/PHD-type zinc finger domain-containing protein [Source:UniProtKB/TrEMBL;Acc:Q9FMZ9]	25568644	25570402	44.12
AT5G62165	AGL42	MADS-box protein AGL42 [Source:UniProtKB/Swiss-Prot;Acc:Q9FIS1]	24964646	24968607	31.15
AT5G62140		ATP-dependent Clp protease ATP-binding subunit [Source:UniProtKB/TrEMBL;Acc:Q9FIS4]	24954216	24955503	37.73
AT5G64610	HAM1	Histone acetyltransferase of the MYST family 1 [Source:UniProtKB/Swiss-Prot;Acc:Q9FLF7]	25828160	25830669	39.24
AT5G61560		U-box domain-containing protein kinase family protein [Source:TAIR;Acc:AT5G61560]	24752783	24756768	38.61
AT5G63630	RH31	DEAD-box ATP-dependent RNA helicase 31 [Source:UniProtKB/Swiss-Prot;Acc:Q9FFQ1]	25472445	25475927	39.53
AT5G64630	FAS2	Chromatin assembly factor 1 subunit FAS2 [Source:UniProtKB/Swiss-Prot;Acc:Q9SXY1]	25833165	25836400	38.91
AT5G63790	ANAC102	NAC domain containing protein 102 [Source:TAIR;Acc:AT5G63790]	25526509	25528281	41.79
AT5G63140	PAP29	Probable inactive purple acid phosphatase 29 [Source:UniProtKB/Swiss-Prot;Acc:Q9FMK9]	25328119	25329882	39.29
AT5G63740		RING/U-box superfamily protein [Source:UniProtKB/TrEMBL;Acc:Q9FFP0]	25510776	25511456	41.12
AT5G61970		Signal recognition particle subunit SRP68 [Source:UniProtKB/TrEMBL;Acc:Q9FH46]	24888596	24893366	37.64
AT5G64120	PER71	Peroxidase [Source:UniProtKB/TrEMBL;Acc:A0A178UAR8]	25659257	25661007	39.98
AT5G63560	FACT	Fatty alcohol:caffeoyl-CoA acyltransferase [Source:UniProtKB/Swiss-Prot;Acc:Q9FFQ7]	25449311	25451419	40.78
AT5G61997		unknown protein; Ha. [Source:TAIR;Acc:AT5G61997]	24908265	24908384	45
AT5G63030	GRXC1	Glutaredoxin-C1 [Source:UniProtKB/Swiss-Prot;Acc:Q8L8T2]	25286177	25287694	38.34
AT5G62190	RH7	DEAD-box ATP-dependent RNA helicase 7 [Source:UniProtKB/Swiss-Prot;Acc:Q39189]	24980268	24984044	38.71
AT5G62360		Plant invertase/pectin methylesterase inhibitor superfamily protein [Source:UniProtKB/TrEMBL;Acc:Q9LVA3]	25040540	25041504	35.85
AT5G62490	HVA22B	HVA22-like protein b [Source:UniProtKB/Swiss-Prot;Acc:Q9SYX7]	25090091	25091559	36.76
AT5G62560	PUB41	U-box domain-containing protein 41 [Source:UniProtKB/Swiss-Prot;Acc:Q0WUF6]	25109840	25112001	45.65
AT5G62690	TUBB3	Tubulin beta-2 chain [Source:UniProtKB/Swiss-Prot;Acc:Q56YW9]	25181296	25183811	42.17
AT5G64300	RIBA1	RIBA1 [Source:UniProtKB/TrEMBL;Acc:A0A178UCT4]	25718008	25721166	39.38
AT5G63595	FLS4	Probable flavonol synthase 4 [Source:UniProtKB/Swiss-Prot;Acc:F4KAS1]	25459087	25460436	40.22
AT5G64480		Putative uncharacterized protein [Source:UniProtKB/TrEMBL;Acc:Q9FGE8]	25778030	25778992	37.69
AT5G62030		Diphthamide synthesis DPH2 family protein [Source:UniProtKB/TrEMBL;Acc:Q8RWW3]	24918479	24920955	40.33
AT5G62200	ATS3B	Embryo-specific protein ATS3B [Source:UniProtKB/Swiss-Prot;Acc:Q6NPM5]	24984048	24985895	37.45
AT5G61412		Putative uncharacterized protein [Source:UniProtKB/TrEMBL;Acc:Q9FLJ3]	24686610	24687366	38.44
AT5G62998		unknown protein; Ha. [Source:TAIR;Acc:AT5G62998]	25279882	25279995	36.84
AT5G63500		At5g63500 [Source:UniProtKB/TrEMBL;Acc:Q9FMV2]	25423370	25423880	40.7
AT5G63930		Non-specific serine/threonine protein kinase [Source:UniProtKB/TrEMBL;Acc:A0A178UJL1]	25581696	25586630	42.05
AT5G61260		Emb [Source:UniProtKB/TrEMBL;Acc:Q9FLK7]	24636296	24638808	37.29
AT5G62440	EMB514	Protein EMBRYO DEFECTIVE 514 [Source:UniProtKB/Swiss-Prot;Acc:Q8L557]	25072117	25074069	37.28
AT5G62850	SWEET5	Bidirectional sugar transporter SWEET5 [Source:UniProtKB/Swiss-Prot;Acc:Q9FM10]	25229938	25231844	33.56
AT5G61610		Oleosin family protein [Source:UniProtKB/TrEMBL;Acc:F4K3K4]	24770046	24771401	40.19
AT5G61050		Histone deacetylase-related / HD-like protein [Source:UniProtKB/TrEMBL;Acc:Q9FNQ9]	24565482	24566615	39.95
AT5G63085		Plant thionin family protein [Source:UniProtKB/TrEMBL;Acc:A8MR43]	25302814	25303355	34.32
AT5G62940	DOF5.6	HCA2 [Source:UniProtKB/TrEMBL;Acc:A0A178UF22]	25256852	25258704	41.18
AT5G61340		Transmembrane protein [Source:UniProtKB/TrEMBL;Acc:Q9FLJ9]	24661679	24663217	37.95
AT5G61070	HDA18	Histone deacetylase 18 [Source:UniProtKB/Swiss-Prot;Acc:Q8LRK8]	24571095	24574387	41.72
AT5G62550		Microtubule-associated futsch-like protein [Source:UniProtKB/TrEMBL;Acc:Q9FJJ1]	25105576	25108013	37.41
AT5G62730	NPF4.7	Protein NRT1/ PTR FAMILY 4.7 [Source:UniProtKB/Swiss-Prot;Acc:Q9FM20]	25197461	25200386	39.34
AT5G64520	XRCC2	homolog of X-ray repair cross complementing 2 (XRCC2) [Source:TAIR;Acc:AT5G64520]	25787969	25791197	36.51
AT5G62310	IRE	Probable serine/threonine protein kinase IRE [Source:UniProtKB/Swiss-Prot;Acc:Q9LE81]	25023155	25028686	39.26
AT5G64000	SAL2	SAL2 phosphatase [Source:UniProtKB/Swiss-Prot;Acc:O49623]	25616625	25618631	39.81
AT5G61030	RBG3	GR-RBP3 [Source:UniProtKB/TrEMBL;Acc:A0A178UBT5]	24560309	24562527	40.78
AT5G64260	EXL2	Protein EXORDIUM-like 2 [Source:UniProtKB/Swiss-Prot;Acc:Q9FE06]	25703649	25705339	44.35
AT5G64813	LIP1	Small GTPase LIP1 [Source:UniProtKB/Swiss-Prot;Acc:Q9C5J9]	25910015	25912900	39.5
AT5G64430		At5g64430 [Source:UniProtKB/TrEMBL;Acc:Q9FGF3]	25762160	25764420	44.18
AT5G64720	EC1.5	EC1.5 [Source:UniProtKB/TrEMBL;Acc:A0A178U9F2]	25872305	25872833	43.86
AT5G64080		Non-specific lipid-transfer protein-like protein At5g64080 [Source:UniProtKB/Swiss-Prot;Acc:Q8VYI9]	25645209	25646845	37.08
AT5G61940		Ubiquitin carboxyl-terminal hydrolase-related protein [Source:TAIR;Acc:AT5G61940]	24867945	24872954	38.8
AT5G64040	PSAN	Photosystem I reaction center subunit PSI-N, chloroplast, putative / PSI-N, putative (PSAN) [Source:UniProtKB/TrEMBL;Acc:F4KC80]	25628521	25629708	37.79
AT5G62573			25113965	25114210	43.9
AT5G62500	EB1B	Microtubule-associated protein RP/EB family member 1B [Source:UniProtKB/Swiss-Prot;Acc:Q9FJJ5]	25092641	25095310	36.78
AT5G61230	PIA2	Phytochrome-interacting ankyrin-repeat protein 2 [Source:UniProtKB/Swiss-Prot;Acc:Q9FNP4]	24627187	24628968	41.64
AT5G61830		At5g61830 [Source:UniProtKB/TrEMBL;Acc:Q501A2]	24836898	24838970	38.11
AT5G61980	AGD1	ADP-ribosylation factor GTPase-activating protein AGD1 [Source:UniProtKB/Swiss-Prot;Acc:Q9FIT8]	24894053	24899973	38.19
AT5G63040		Emb [Source:UniProtKB/TrEMBL;Acc:Q9FM48]	25288146	25290420	40.18
AT5G63520		F-box/LRR-repeat protein At5g63520 [Source:UniProtKB/Swiss-Prot;Acc:Q9FMV0]	25425950	25429255	36.66
AT5G63010		Transducin/WD40 repeat-like superfamily protein [Source:UniProtKB/TrEMBL;Acc:Q9FM51]	25281676	25283176	41.57
AT5G62623		Putative defensin-like protein 264 [Source:UniProtKB/Swiss-Prot;Acc:Q2V2W3]	25140727	25141351	30.4
AT5G62660		Putative F-box protein At5g62660 [Source:UniProtKB/Swiss-Prot;Acc:Q9LV12]	25156326	25157465	42.72
AT5G62130		Per1-like family protein [Source:UniProtKB/TrEMBL;Acc:F4K556]	24950264	24953362	39.21
AT5G63490	CBSCBSPB1	CBS domain-containing protein CBSCBSPB1 [Source:UniProtKB/Swiss-Prot;Acc:Q9FMV3]	25418594	25422466	37.85
AT5G61100		BEST Arabidopsis thaliana protein match is: zinc ion binding (TAIR:AT5G61110.1); Ha. [Source:TAIR;Acc:AT5G61100]	24579090	24580481	35.92
AT5G61865		At5g61865 [Source:UniProtKB/TrEMBL;Acc:Q501B3]	24847310	24850118	38.91
AT5G61250	AtGUS1	GUS1 [Source:UniProtKB/TrEMBL;Acc:A0A384LHC0]	24632154	24635312	38.02
AT5G61450		P-loop containing nucleoside triphosphate hydrolases superfamily protein [Source:UniProtKB/TrEMBL;Acc:Q9FII8]	24709862	24713010	35.76
AT5G61950		Ubiquitin carboxyl-terminal hydrolase-related protein [Source:UniProtKB/TrEMBL;Acc:F4K529]	24873053	24878309	38.88
AT5G63070	RPS15F	40S ribosomal protein S15-6 [Source:UniProtKB/Swiss-Prot;Acc:Q9FML6]	25299178	25299673	43.75
AT5G63550		DEK domain-containing chromatin associated protein [Source:UniProtKB/TrEMBL;Acc:Q9FFQ8]	25444575	25448255	37.54
AT5G64450		Emb [Source:UniProtKB/TrEMBL;Acc:Q9FGF1]	25771949	25772761	39.24
AT5G61020	ECT3	ECT3 [Source:UniProtKB/TrEMBL;Acc:A0A178UM32]	24556994	24559936	37.99
AT5G62065	LTP14	Putative non-specific lipid-transfer protein 14 [Source:UniProtKB/Swiss-Prot;Acc:Q9FIT2]	24929207	24929778	42.83
AT5G62230	ERL1	LRR receptor-like serine/threonine-protein kinase ERL1 [Source:UniProtKB/Swiss-Prot;Acc:C0LGW6]	24996129	25002439	36.43
AT5G62865		Uncharacterized protein At5g62860/MQB2_160 [Source:UniProtKB/TrEMBL;Acc:Q9FM08]	25233881	25234884	42.53
AT5G62530	ALDH12A1	Delta-1-pyrroline-5-carboxylate dehydrogenase 12A1, mitochondrial [Source:UniProtKB/Swiss-Prot;Acc:Q8VZC3]	25099003	25103312	37.84
AT5G62820		CASP-like protein [Source:UniProtKB/TrEMBL;Acc:A0A178U8F8]	25223727	25225052	42.61
AT5G63480	MED30	Mediator of RNA polymerase II transcription subunit 30 [Source:UniProtKB/Swiss-Prot;Acc:Q9FMV4]	25416833	25418635	38.38
AT5G62420		Aldo/keto reductase family protein [Source:UniProtKB/TrEMBL;Acc:Q9FJK0]	25064793	25066336	39.18
AT5G62520	SRO5	Probable inactive poly [ADP-ribose] polymerase SRO5 [Source:UniProtKB/Swiss-Prot;Acc:Q9FJJ3]	25097944	25099907	37.93
AT5G61660		At5g61660 [Source:UniProtKB/TrEMBL;Acc:Q9FKF5]	24778653	24779945	38.28
AT5G64240	AMC3	Metacaspase-3 [Source:UniProtKB/Swiss-Prot;Acc:Q9FMG1]	25695620	25697416	42.35
AT5G62300	RPS20C	40S ribosomal protein S20-1 [Source:UniProtKB/Swiss-Prot;Acc:P49200]	25021200	25022564	36.7
AT5G62370		Pentatricopeptide repeat-containing protein At5g62370 [Source:UniProtKB/Swiss-Prot;Acc:Q9LVA2]	25041819	25045047	39.33
AT5G63230		Carbohydrate-binding X8 domain superfamily protein [Source:UniProtKB/TrEMBL;Acc:A8MRV8]	25357360	25357822	38.88
AT5G63170		GDSL esterase/lipase At5g63170 [Source:UniProtKB/Swiss-Prot;Acc:Q9FMK6]	25338699	25340096	35.84
AT5G63250		At5g63250 [Source:UniProtKB/TrEMBL;Acc:Q9FMJ8]	25360121	25360695	37.04
AT5G64100	PER69	Peroxidase [Source:UniProtKB/TrEMBL;Acc:A0A178U798]	25650567	25652247	41.58
AT5G61680		Pectin lyase-like superfamily protein [Source:TAIR;Acc:AT5G61680]	24786162	24788060	38.39

AT5G63990	SAL3	Probable SAL3 phosphatase [Source:UniProtKB/Swiss-Prot;Acc:Q8GY63]	25613096	25615901	36.78
AT5G63530	HIPP07	Heavy metal-associated isoprenylated plant protein 7 [Source:UniProtKB/Swiss-Prot;Acc:Q9C5D3]	25432890	25435834	34.67
AT5G60980		AT5g60980/MSL3_100 [Source:UniProtKB/TrEMBL;Acc:Q9FME2]	24543136	24546352	39.45
AT5G61810		Mitochondrial substrate carrier family protein [Source:UniProtKB/TrEMBL;Acc:Q9FLS8]	24831574	24833984	39.82
AT5G63800	BGAL6	Beta-galactosidase 6 [Source:UniProtKB/Swiss-Prot;Acc:Q9FFN4]	25530055	25535889	33.86
AT5G63970	RGLG3	E3 ubiquitin-protein ligase RGLG3 [Source:UniProtKB/Swiss-Prot;Acc:Q8RX26]	25606771	25609306	36.91
AT5G63100		S-adenosyl-L-methionine-dependent methyltransferases superfamily protein [Source:UniProtKB/TrEMBL;Acc:Q9FML3]	25314104	25315533	40.91
AT5G62740	HIR1	HIR4 [Source:UniProtKB/TrEMBL;Acc:A0A178U8N7]	25200838	25202839	37.36
AT5G64030		Probable methyltransferase PMT26 [Source:UniProtKB/Swiss-Prot;Acc:Q8L7V3]	25623952	25628559	40.15
AT5G63130		At5g63130 [Source:UniProtKB/TrEMBL;Acc:Q9FML0]	25322975	25324739	39.72
AT5G63160	BT1	BTB/POZ and TAZ domain-containing protein 1 [Source:UniProtKB/Swiss-Prot;Acc:Q9FMK7]	25333172	25335570	37.31
AT5G62280		At5g62280 [Source:UniProtKB/TrEMBL;Acc:Q9LVA8]	25016860	25018265	37.48
AT5G62290		Chloride conductance regulatory protein ICln [Source:UniProtKB/Swiss-Prot;Acc:Q9LVA7]	25019175	25021131	37.05
AT5G64060	anac103	NAC domain containing protein 103 [Source:UniProtKB/TrEMBL;Acc:Q9FMJ1]	25633455	25635789	40.64
AT5G61240		Leucine-rich repeat (LRR) family protein [Source:UniProtKB/TrEMBL;Acc:F4K217]	24629256	24632211	37.48
AT5G64160		AT5g64160/MHJ24_14 [Source:UniProtKB/TrEMBL;Acc:Q94C20]	25670930	25672710	38.01
AT5G62180	CXE20	Probable carboxylesterase 120 [Source:UniProtKB/Swiss-Prot;Acc:Q9LVB8]	24978718	24980075	42.64
AT5G61750		Germin-like protein subfamily 3 member 4 [Source:UniProtKB/Swiss-Prot;Acc:Q9FLT3]	24812804	24813436	46.45
AT5G63270		RPM1-interacting protein 4 (RIN4) family protein [Source:UniProtKB/TrEMBL;Acc:Q9FMJ6]	25365230	25366012	39.21
AT5G61580	PFK4	ATP-dependent 6-phosphofructokinase [Source:UniProtKB/TrEMBL;Acc:A0A178UCI3]	24761041	24764168	37.53
AT5G64460		Phosphoglycerate mutase-like protein 1 [Source:UniProtKB/Swiss-Prot;Acc:Q9FGF0]	25772848	25775635	36.08
AT5G63870	PP7	Serine/threonine-protein phosphatase 7 [Source:UniProtKB/Swiss-Prot;Acc:Q9FN02]	25561070	25563316	39.88
AT5G63370	CDKG1	Cyclin-dependent kinase G1 [Source:UniProtKB/Swiss-Prot;Acc:Q9FGW5]	25383810	25387270	40.74
AT5G61420	MYB28	PMG1 [Source:UniProtKB/TrEMBL;Acc:A0A178UN88]	24689069	24691187	38.08
AT5G64750	ABR1	Ethylene-responsive transcription factor ABR1 [Source:UniProtKB/Swiss-Prot;Acc:Q9FGF8]	25891449	25893888	37.34
AT5G63200		Tetratricopeptide repeat (TPR)-containing protein [Source:UniProtKB/TrEMBL;Acc:Q67YJ9]	25348903	25353039	40.2
AT5G64250		2-nitropropane dioxygenase-like protein [Source:UniProtKB/TrEMBL;Acc:Q9FMG0]	25697354	25699255	39.59
AT5G63440		UPF0235 protein At5g63440 [Source:UniProtKB/Swiss-Prot;Acc:Q8GWQ6]	25406252	25408456	37.96
AT5G64110	PER70	Peroxidase [Source:UniProtKB/TrEMBL;Acc:A0A178UNC2]	25654074	25656067	37.71
AT5G63680		Pyruvate kinase [Source:UniProtKB/TrEMBL;Acc:Q9FFP6]	25490065	25492763	40.2
AT5G64670		Ribosomal protein L18e/L15 superfamily protein [Source:UniProtKB/TrEMBL;Acc:Q9FLF3]	25852241	25854067	40.39
AT5G62710		Leucine-rich repeat protein kinase family protein [Source:UniProtKB/TrEMBL;Acc:Q8GX94]	25187221	25190722	38.18
AT5G62050	OXA1	Mitochondrial inner membrane protein OXA1 [Source:UniProtKB/Swiss-Prot;Acc:Q42191]	24923465	24927314	37.51
AT5G64850		At5g64850 [Source:UniProtKB/TrEMBL;Acc:Q0WN19]	25921402	25923418	32.33
AT5G61330		rRNA processing protein-related [Source:TAIR;Acc:AT5G61330]	24657097	24661241	36.96
AT5G62900		Basic-leucine zipper transcription factor K [Source:UniProtKB/TrEMBL;Acc:Q9FM06]	25248690	25250032	34.55
AT5G64340	SAC51	Transcription factor SAC51 [Source:UniProtKB/Swiss-Prot;Acc:Q9FMF4]	25730335	25733313	38.7
AT5G61530		Uncharacterized Rho GTPase-activating protein At5g61530 [Source:UniProtKB/Swiss-Prot;Acc:Q3E875]	24741976	24744823	38.52
AT5G63880	VPS20.1	SNF7 family protein [Source:UniProtKB/TrEMBL;Acc:F4KC57]	25563724	25565498	36.28
AT5G64740	CESA6	Cellulose synthase A catalytic subunit 6 [UDP-forming] [Source:UniProtKB/Swiss-Prot;Acc:Q94JQ6]	25881033	25886728	39.48
AT5G64700		WAT1-related protein At5g64700 [Source:UniProtKB/Swiss-Prot;Acc:Q9FGG3]	25864937	25867025	33.99
AT5G61720		Dbj [Source:UniProtKB/TrEMBL;Acc:Q9FLT6]	24801505	24803395	38.5
AT5G61920	FLXL4	FLX4 [Source:UniProtKB/TrEMBL;Acc:A0A178USJ6]	24864290	24866019	39.88
AT5G62460		RING/FYVE/PHD zinc finger superfamily protein [Source:TAIR;Acc:AT5G62460]	25074782	25077398	37.52
AT5G61190		putative endonuclease or glycosyl hydrolase with C2H2-type zinc finger domain [Source:TAIR;Acc:AT5G61190]	24615253	24620053	39.87
AT5G63640	TOL5	TOM1-like protein 5 [Source:UniProtKB/Swiss-Prot;Acc:Q9FFQ0]	25477541	25481715	38.11
AT5G64680		Mediator-associated protein 2 [Source:UniProtKB/Swiss-Prot;Acc:F4KF27]	25854077	25855484	37.57
AT5G63730	ARI14	Probable E3 ubiquitin-protein ligase ARI14 [Source:UniProtKB/Swiss-Prot;Acc:Q9FFP1]	25508100	25509706	40.63
AT5G61590	ERF107	Ethylene-responsive transcription factor ERF107 [Source:UniProtKB/Swiss-Prot;Acc:Q9FKG2]	24764256	24765511	39.25
AT5G63375		Putative uncharacterized protein [Source:UniProtKB/TrEMBL;Acc:Q9FGW6]	25384040	25384487	37.5
AT5G61310		Cytochrome c oxidase subunit 5C [Source:UniProtKB/TrEMBL;Acc:A0A178UCG7]	24653261	24654940	36.55
AT5G63750	ARI13	Probable E3 ubiquitin-protein ligase ARI13 [Source:UniProtKB/Swiss-Prot;Acc:Q9FFN9]	25512994	25514941	41.84
AT5G62970		Putative F-box/FBD/LRR-repeat protein At5g62970 [Source:UniProtKB/Swiss-Prot;Acc:Q9FM55]	25272033	25273537	38.94
AT5G63610	CDKE-1	Cyclin-dependent kinase E-1 [Source:UniProtKB/Swiss-Prot;Acc:Q84TI6]	25463362	25465922	42.41
AT5G64530	NAC104	NAC domain-containing protein 104 [Source:UniProtKB/Swiss-Prot;Acc:Q8GWK6]	25794957	25797031	35.18
AT5G60990	RH10	DEAD-box ATP-dependent RNA helicase 10 [Source:UniProtKB/Swiss-Prot;Acc:Q8GY84]	24546329	24549339	37.66
AT5G62120	ARR23	Response regulator 23 [Source:UniProtKB/TrEMBL;Acc:F4K554]	24946955	24948225	31.24
AT5G62000	ARF2	Auxin response factor 2 [Source:UniProtKB/Swiss-Prot;Acc:Q94JM3]	24910191	24915395	41.15
AT5G62627		Putative defensin-like protein 265 [Source:UniProtKB/Swiss-Prot;Acc:Q2V2W2]	25142290	25142960	31.15
AT5G64560	MRS2-2	Magnesium transporter MRS2-2 [Source:UniProtKB/Swiss-Prot;Acc:Q9FLG2]	25806698	25809711	38.65
AT5G63410		Leucine-rich repeat protein kinase family protein [Source:UniProtKB/TrEMBL;Acc:Q84JF8]	25394938	25398282	36.41
AT5G63860	UVR8	Ultraviolet-B receptor UVR8 [Source:UniProtKB/Swiss-Prot;Acc:Q9FN03]	25554416	25559499	36.88
AT5G62220	XLT2	Xyloglucan galactosyltransferase XLT2 [Source:UniProtKB/Swiss-Prot;Acc:F4K6F1]	24988214	24990331	43.3
AT5G62330		BEST Arabidopsis thaliana protein match is: Plant invertase/pectin methylesterase inhibitor superfamily protein (TAIR:AT5G62340.1); Ha. [Sou	25031563	25032276	46.36
AT5G61130	PDCB1	PDCB1 [Source:UniProtKB/TrEMBL;Acc:A0A178URD1]	24587173	24589580	36.54
AT5G63320	GTE10	NPX1 [Source:UniProtKB/TrEMBL;Acc:A0A178UIE5]	25373955	25379297	39.29
AT5G61010	ATEXO70E2	Exocyst complex component EXO70E2 [Source:UniProtKB/Swiss-Prot;Acc:Q9FNR3]	24553744	24557043	40.45
AT5G63670	SPT42	Transcription elongation factor SPT4 homolog 2 [Source:UniProtKB/Swiss-Prot;Acc:Q94C60]	25487927	25489600	36.32
AT5G64290	DIT2-1	Dicarboxylate transporter 2.1, chloroplastic [Source:UniProtKB/Swiss-Prot;Acc:Q9FMF7]	25714178	25716901	42.95
AT5G64770	RGF9	RGF9 [Source:UniProtKB/TrEMBL;Acc:A0A178UIJ2]	25897321	25898171	31.49
AT5G64550		Emb [Source:UniProtKB/TrEMBL;Acc:Q9FLG3]	25801528	25804980	41.3
AT5G61890	ERF114	Ethylene-responsive transcription factor ERF114 [Source:UniProtKB/Swiss-Prot;Acc:Q9FH54]	24852322	24853941	36.67
AT5G63225		At5g63230 [Source:UniProtKB/TrEMBL;Acc:Q8GX54]	25356133	25356767	35.75
AT5G64180		Dbj [Source:UniProtKB/TrEMBL;Acc:Q9FMG6]	25677072	25678702	39.55
AT5G62980	FOLB2	Dihydroneopterin aldolase 2 [Source:UniProtKB/Swiss-Prot;Acc:Q9FM54]	25276017	25277293	35.4
AT5G62110		Homeodomain-like superfamily protein [Source:TAIR;Acc:AT5G62110]	24942169	24946087	30.06
AT5G61790	CNX1	Calnexin homolog 1 [Source:UniProtKB/Swiss-Prot;Acc:P29402]	24827063	24829900	41.51
AT5G62510		F-box protein At5g62510 [Source:UniProtKB/Swiss-Prot;Acc:Q9FJJ4]	25095373	25096635	42.12
AT5G62060		Putative F-box protein At5g62060 [Source:UniProtKB/Swiss-Prot;Acc:Q9FIT3]	24927688	24928698	41.05
AT5G63720	KPL	Protein KOKOPELLI [Source:UniProtKB/Swiss-Prot;Acc:Q9FFP2]	25506279	25508017	40.54
AT5G62670	AHA11	ATPase 11, plasma membrane-type [Source:UniProtKB/Swiss-Prot;Acc:Q9LV11]	25159208	25165209	39.37
AT5G63350		Emb [Source:UniProtKB/TrEMBL;Acc:Q9FGW7]	25379931	25381317	44.12
AT5G61110		Gb [Source:UniProtKB/TrEMBL;Acc:Q9FNQ4]	24581078	24582130	37.51
AT5G64540		Mucin-like protein [Source:UniProtKB/TrEMBL;Acc:Q5BPE9]	25798697	25801302	41.67
AT5G63087		Plant thionin family protein [Source:UniProtKB/TrEMBL;Acc:A8MSA5]	25304750	25305371	33.6
AT5G62070	IQD23	IQD23 [Source:UniProtKB/TrEMBL;Acc:A0A178UJA3]	24929859	24932686	38.68
AT5G63600	FLS5	Flavonol synthase 5 [Source:UniProtKB/TrEMBL;Acc:F4KAS3]	25460591	25462568	38.93
AT5G61170	RPS19C	40S ribosomal protein S19-3 [Source:UniProtKB/Swiss-Prot;Acc:Q9FNP8]	24611032	24612564	39.2
AT5G62990	emb1692	Ubiquitin carboxyl-terminal hydrolase family protein [Source:UniProtKB/TrEMBL;Acc:Q9FM53]	25277404	25279474	39.69
AT5G64790		Beta-1,3-glucanase [Source:UniProtKB/TrEMBL;Acc:Q9LV98]	25902533	25904804	38.64
AT5G62580	TOR1L3	TORTIFOLIA1-like protein 3 [Source:UniProtKB/Swiss-Prot;Acc:Q6NPR6]	25119511	25122578	40.74
AT5G61460	SMC6B	Structural maintenance of chromosomes protein 6B [Source:UniProtKB/Swiss-Prot;Acc:Q9FII7]	24714076	24721970	37.23
AT5G63780	SHA1	At5g63780 [Source:UniProtKB/TrEMBL;Acc:Q8GUG6]	25523827	25526593	38.67
AT5G64280	DIT2-2	Dicarboxylate transporter 2.2, chloroplastic [Source:UniProtKB/Swiss-Prot;Acc:Q9FMF8]	25710999	25713574	43.01
AT5G63150		At5g63150 [Source:UniProtKB/TrEMBL;Acc:Q9FMK8]	25330125	25330907	38.95
AT5G62270		Gb [Source:UniProtKB/TrEMBL;Acc:Q9LVA9]	25011957	25014812	39.43
AT5G62640	ELF5	Proline-rich family protein [Source:UniProtKB/TrEMBL;Acc:F4K7R6]	25149329	25152563	40.28
AT5G63660	PDF2.5	Defensin-like protein 6 [Source:UniProtKB/Swiss-Prot;Acc:Q9FFP8]	25485486	25486324	34.21
AT5G63620		AT5g63620/MBK5_9 [Source:UniProtKB/TrEMBL;Acc:Q93ZM6]	25466128	25468550	40.28
AT5G61210	SNAP33	SNP33 [Source:UniProtKB/TrEMBL;Acc:A0A178UGR9]	24623248	24625631	38.05
AT5G61550		U-box domain-containing protein kinase family protein [Source:UniProtKB/TrEMBL;Acc:F4K3J3]	24748023	24751871	42.19
AT5G61740	ABCA10	ABC transporter A family member 10 [Source:UniProtKB/Swiss-Prot;Acc:Q9FLT4]	24808406	24812739	38.99
AT5G62040	BFT	Protein BROTHER of FT and TFL 1 [Source:UniProtKB/Swiss-Prot;Acc:Q9FIT4]	24922802	24923797	39.06
AT5G61630		Transmembrane protein [Source:UniProtKB/TrEMBL;Acc:Q1PDG7]	24773229	24774578	38.07
AT5G61760	IPK2B	Inositol polyphosphate multikinase beta [Source:UniProtKB/Swiss-Prot;Acc:Q9FLT2]	24813660	24815458	39.02

AT5G63340		Putative uncharacterized protein [Source:UniProtKB/TrEMBL;Acc:Q9FGW8]	25378894	25379674	38.92
AT5G61180		Putative endonuclease or glycosyl hydrolase [Source:TAIR;Acc:AT5G61180]	24612867	24614471	42.31
AT5G63260		Zinc finger C-x8-C-x5-C-x3-H type family protein [Source:UniProtKB/TrEMBL;Acc:F4K9A6]	25361713	25364882	39.05
AT5G62830		F-box associated ubiquitination effector family protein [Source:UniProtKB/TrEMBL;Acc:F4K7U0]	25226168	25227131	39.94
AT5G61900	BON1	Protein BONZAI 1 [Source:UniProtKB/Swiss-Prot;Acc:Q941L3]	24855661	24859341	38.11
AT5G62720		At5g62720 [Source:UniProtKB/TrEMBL;Acc:Q8GZ51]	25191402	25195071	32.23
AT5G62890	NAT6	Nucleobase-ascorbate transporter 6 [Source:UniProtKB/Swiss-Prot;Acc:Q27GI3]	25243148	25247377	38.25
AT5G63060		Sec14p-like phosphatidylinositol transfer family protein [Source:UniProtKB/TrEMBL;Acc:F4K975]	25294954	25296896	36.8
AT5G62880	ARAC10	ROP11 [Source:UniProtKB/TrEMBL;Acc:A0A384LM06]	25236945	25239262	34.47
AT5G61290		Flavin-containing monooxygenase FMO GS-OX-like 8 [Source:UniProtKB/Swiss-Prot;Acc:Q9FLK4]	24648528	24650815	36.36
AT5G64800	CLE21	CLAVATA3/ESR (CLE)-related protein 21 [Source:UniProtKB/Swiss-Prot;Acc:Q9LV97]	25905956	25906713	33.77
AT5G64410	OPT4	Isp4-like protein [Source:UniProtKB/TrEMBL;Acc:Q0WUW4]	25750845	25755501	36.8
AT5G64150		At5g64156 [Source:UniProtKB/TrEMBL;Acc:Q9FMI5]	25668755	25671218	40.75
AT5G63063		Plant thionin family protein [Source:TAIR;Acc:AT5G63063]	25297420	25298206	36.98
AT5G63220		At5g63220 [Source:UniProtKB/TrEMBL;Acc:Q6GKV1]	25353018	25355535	36.5
AT5G61730	ABCA9	ABC transporter A family member 9 [Source:UniProtKB/Swiss-Prot;Acc:Q9FLT5]	24803427	24807920	38.34
AT5G62910		At5g62910 [Source:UniProtKB/TrEMBL;Acc:Q9FM05]	25250256	25252354	38.78
AT5G64070	PI4KB1	Phosphatidylinositol 4-kinase beta 1 [Source:UniProtKB/Swiss-Prot;Acc:Q9FMJ0]	25636882	25644478	38.61
AT5G64320		Pentatricopeptide repeat-containing protein At5g64320, mitochondrial [Source:UniProtKB/Swiss-Prot;Acc:Q9FMF6]	25723049	25725534	40.63
AT5G64687		Ubiquinol oxidase [Source:UniProtKB/TrEMBL;Acc:A0A1P8BB03]	25858584	25861909	31.21
AT5G63300		At5g63300 [Source:UniProtKB/TrEMBL;Acc:Q9FMJ3]	25371091	25372017	44.12
AT5G61370		Pentatricopeptide repeat-containing protein At5g61370, mitochondrial [Source:UniProtKB/Swiss-Prot;Acc:Q9FLJ6]	24671770	24673657	40.1
AT5G63180		Probable pectate lyase 22 [Source:UniProtKB/Swiss-Prot;Acc:Q93Z25]	25340914	25343369	37.34
AT5G61990		Pentatricopeptide repeat-containing protein At5g61990, mitochondrial [Source:UniProtKB/Swiss-Prot;Acc:Q9FIT7]	24899606	24903131	39.56
AT5G61500	ATG3	Autophagy-related protein 3 [Source:UniProtKB/TrEMBL;Acc:A0A178UM04]	24733785	24736876	37.23
AT5G64420		DNA polymerase V family [Source:UniProtKB/TrEMBL;Acc:Q9FGF4]	25756272	25761516	39.89
AT5G61480	TDR	Leucine-rich repeat receptor-like protein kinase TDR [Source:UniProtKB/Swiss-Prot;Acc:Q9FII5]	24724337	24728244	41.79
AT5G62770		At5g62770 [Source:UniProtKB/TrEMBL;Acc:Q9FM16]	25210301	25211665	42.27
AT5G62700	TUBB3	Tubulin beta-2 chain [Source:UniProtKB/Swiss-Prot;Acc:Q56YW9]	25184258	25186777	42.02
AT5G63540	ATRMI1	Domain of unknown function (DUF1767) [Source:TAIR;Acc:AT5G63540]	25440783	25444471	38.66
AT5G64330	RPT3	Root phototropism protein 3 [Source:UniProtKB/Swiss-Prot;Acc:Q9FMF5]	25727126	25730358	42.25
AT5G62080		Bifunctional inhibitor/lipid-transfer protein/seed storage 2S albumin superfamily protein [Source:UniProtKB/TrEMBL;Acc:Q8GT78]	24933471	24934484	34.42
AT5G62150		At5g62150 [Source:UniProtKB/TrEMBL;Acc:Q9FIS3]	24958125	24958813	36.72
AT5G63120	RH30	DEAD-box ATP-dependent RNA helicase 30 [Source:UniProtKB/Swiss-Prot;Acc:Q8W4R3]	25318642	25322284	40.13
AT5G64190		unknown protein; BEST Arabidopsis thaliana protein match is: unknown protein (TAIR:AT2G40390.1); Ha. [Source:TAIR;Acc:AT5G64190]	25679425	25681265	40.74
AT5G63090	LOB	LOB [Source:UniProtKB/TrEMBL;Acc:A0A178UKE6]	25308516	25311019	35.06
AT5G63240		At5g63240 [Source:UniProtKB/TrEMBL;Acc:Q9FMJ9]	25358968	25359565	40.13
AT5G63390	OFUT37	O-fucosyltransferase 37 [Source:UniProtKB/Swiss-Prot;Acc:Q9FMW3]	25390249	25392684	41.71
AT5G61380	APRR1	Two-component response regulator-like APRR1 [Source:UniProtKB/Swiss-Prot;Acc:Q9LKL2]	24674963	24678550	40.27
AT5G61160	ACT	Agmatine coumaroyltransferase [Source:UniProtKB/Swiss-Prot;Acc:Q9FNP9]	24608605	24610329	44.41
AT5G62020	HSFB2A	HSFB2A [Source:UniProtKB/TrEMBL;Acc:A0A384KDZ5]	24915807	24917643	38.6
AT5G61510		At5g61510 [Source:UniProtKB/TrEMBL;Acc:A1L4Y4]	24736871	24739002	41.14
AT5G62575	SDH7B	SDH7B [Source:UniProtKB/TrEMBL;Acc:A0A178UBI4]	25117414	25118892	35.43
AT5G62960		At5g62960 [Source:UniProtKB/TrEMBL;Acc:Q6NPD1]	25268950	25271823	34.41
AT5G61620		Probable transcription factor At5g61620 [Source:UniProtKB/Swiss-Prot;Acc:Q9FKF9]	24772326	24773507	40.78
AT5G64730		Transducin/WD40 repeat-like superfamily protein [Source:UniProtKB/TrEMBL;Acc:Q94AH2]	25873101	25875293	37.3
AT5G63470	NFYC4	NF-YC4 [Source:UniProtKB/TrEMBL;Acc:A0A384KX14]	25415600	25417199	39.94
AT5G64710		Emb [Source:UniProtKB/TrEMBL;Acc:Q9FGG2]	25869222	25872269	40.52
AT5G64220	CAMTA2	Calmodulin-binding transcription activator 2 [Source:UniProtKB/Swiss-Prot;Acc:Q6NPP4]	25686193	25692339	39.22
AT5G64470	TBL12	Protein trichome birefringence-like 12 [Source:UniProtKB/Swiss-Prot;Acc:Q9FGE9]	25775975	25777963	40.62
AT5G61220		At5g61220 [Source:UniProtKB/TrEMBL;Acc:Q8L9E3]	24625573	24626965	37.04
AT5G63290		At5g63290 [Source:UniProtKB/TrEMBL;Acc:Q9FMJ4]	25369245	25370940	40.51
AT5G63135		At5g63135 [Source:UniProtKB/TrEMBL;Acc:Q8LC33]	25324754	25326479	35.34
AT5G63840	RSW3	Glycosyl hydrolases family 31 protein [Source:TAIR;Acc:AT5G63840]	25544907	25549199	41.58
AT5G64580	FTSHI4	Probable inactive ATP-dependent zinc metalloprotease FTSHI 4, chloroplastic [Source:UniProtKB/Swiss-Prot;Acc:F4KF14]	25816918	25821567	37.16
AT5G63830		HIT-type Zinc finger family protein [Source:UniProtKB/TrEMBL;Acc:Q9FN06]	25543331	25544898	40.75
AT5G63760	ARI15	Probable E3 ubiquitin-protein ligase ARI15 [Source:UniProtKB/Swiss-Prot;Acc:Q84RQ8]	25515662	25517891	40.81
AT5G64090		At5g64090 [Source:UniProtKB/TrEMBL;Acc:Q9FMI8]	25647913	25649768	44.34
AT5G62240		Cell cycle regulated microtubule associated protein [Source:UniProtKB/TrEMBL;Acc:Q5XUX8]	25002358	25004783	36.56
AT5G62950		At5g62950 [Source:UniProtKB/TrEMBL;Acc:Q7XJ61]	25262162	25264053	35.68
AT5G61140	BRR2C	DExH-box ATP-dependent RNA helicase DExH14 [Source:UniProtKB/Swiss-Prot;Acc:Q9FNQ1]	24589906	24603522	37.19
AT5G60964		ECA1 gametogenesis related family protein [Source:UniProtKB/TrEMBL;Acc:A8MSD1]	24530572	24530924	32.01
AT5G62650		AT5g62650/MRG21_7 [Source:UniProtKB/TrEMBL;Acc:Q940R3]	25152625	25155983	38.61
AT5G62160	ZIP12	ZIP12 [Source:UniProtKB/TrEMBL;Acc:A0A178UGP0]	24960107	24961864	39.82
AT5G64620	C/VIF2	Cell wall / vacuolar inhibitor of fructosidase 2 [Source:UniProtKB/Swiss-Prot;Acc:O49603]	25831635	25832731	38.38
AT5G63400	ADK1	ADK1 [Source:UniProtKB/TrEMBL;Acc:A0A178UAM7]	25392920	25394907	39.24
AT5G61710		Cotton fiber protein [Source:UniProtKB/TrEMBL;Acc:Q9FLT7]	24799836	24800783	34.7
AT5G61690	ABCA11	ABC transporter A family member 11 [Source:UniProtKB/Swiss-Prot;Acc:Q9FKF2]	24789237	24793528	38.68
AT5G63950	CHR24	Protein CHROMATIN REMODELING 24 [Source:UniProtKB/Swiss-Prot;Acc:Q8W103]	25591956	25598671	38.16
AT5G63110	HDA6	SIL1 [Source:UniProtKB/TrEMBL;Acc:A0A384KCB8]	25315506	25318347	41.73
AT5G62800		Protein with RING/U-box and TRAF-like domains [Source:TAIR;Acc:AT5G62800]	25218634	25219967	39.06
AT5G63280		C2H2-like zinc finger protein [Source:UniProtKB/TrEMBL;Acc:Q8VZP2]	25367031	25369297	34.63
AT5G61000	RPA1D	Replication protein A subunit [Source:UniProtKB/TrEMBL;Acc:A0A178UJ91]	24549479	24552855	37.64
AT5G64440	FAAH	Fatty acid amide hydrolase [Source:UniProtKB/Swiss-Prot;Acc:Q7XJJ7]	25765712	25770618	37.38
AT5G62380	NAC101	NAC domain-containing protein 101 [Source:UniProtKB/Swiss-Prot;Acc:Q9LVA1]	25050152	25052054	35.37
AT5G62680	NPF2.11	GTR2 [Source:UniProtKB/TrEMBL;Acc:A0A178UEQ7]	25165137	25168060	40.39
AT5G61910		DCD (Development and Cell Death) domain protein [Source:UniProtKB/TrEMBL;Acc:F4K518]	24855621	24864172	38.8
AT5G61650	CYCU4-3	Cyclin-U4-3 [Source:UniProtKB/Swiss-Prot;Acc:Q9FKF6]	24778191	24779989	38.85
AT5G62250	MAP65-9	65-kDa microtubule-associated protein 9 [Source:UniProtKB/Swiss-Prot;Acc:Q4PSA3]	25005434	25008361	33.71
AT5G63570	GSA1	Glutamate-1-semialdehyde 2,1-aminomutase 1, chloroplastic [Source:UniProtKB/Swiss-Prot;Acc:P42799]	25451800	25453845	43.94
AT5G63850	AAP4	AAP4 [Source:UniProtKB/TrEMBL;Acc:A0A178UDW1]	25550937	25553656	39.56
AT5G64640	PME64	Probable pectinesterase/pectinesterase inhibitor 64 [Source:UniProtKB/Swiss-Prot;Acc:Q8L7Q7]	25836514	25839220	41.97
AT5G64270		Nuclear protein-like [Source:UniProtKB/TrEMBL;Acc:Q9FMF9]	25706608	25711355	44.38
AT5G61320	CYP89A3	Cytochrome P450, family 89, subfamily A, polypeptide 3 [Source:UniProtKB/TrEMBL;Acc:F4K231]	24655098	24656638	45.1
AT5G61270	BHLH72	Transcription factor PIF7 [Source:UniProtKB/Swiss-Prot;Acc:Q570R7]	24638491	24640765	40.97
AT5G62410	SMC2-1	Structural maintenance of chromosomes protein 2-1 [Source:UniProtKB/Swiss-Prot;Acc:Q9C5Y4]	25056025	25062670	37.93
AT5G64210	AOX2	Ubiquinol oxidase 2, mitochondrial [Source:UniProtKB/Swiss-Prot;Acc:O22049]	25683770	25685731	37.82
AT5G61200		BEST Arabidopsis thaliana protein match is: myosin heavy chain-related (TAIR:AT5G07890.2); Ha. [Source:TAIR;Acc:AT5G61200]	24620144	24622666	37.38
AT5G61120		BEST Arabidopsis thaliana protein match is: Polynucleotidyl transferase, ribonuclease H-like superfamily protein (TAIR:AT5G61090.1); Ha. [Sc	24582225	24585818	36.78
AT5G62400		Transmembrane protein [Source:UniProtKB/TrEMBL;Acc:Q9LV99]	25054614	25055664	38.53
AT5G62470	MYB96	Transcription factor MYB96 [Source:UniProtKB/Swiss-Prot;Acc:Q24JK1]	25078991	25081141	36.12
AT5G62430	CDF1	Cyclic dof factor 1 [Source:UniProtKB/Swiss-Prot;Acc:Q8W1E3]	25069093	25071111	36.26
AT5G62630	HIPL2	HIPL2 protein [Source:UniProtKB/Swiss-Prot;Acc:Q94F08]	25143518	25146587	39.41
AT5G61400		Pentatricopeptide repeat-containing protein At5g61400 [Source:UniProtKB/Swiss-Prot;Acc:Q9FLJ4]	24681550	24683514	40.71
AT5G63890	HISN8	Histidinol dehydrogenase, chloroplastic [Source:UniProtKB/TrEMBL;Acc:A0A178UP37]	25565431	25568206	39.05
AT5G63980	SAL1	SAL1 phosphatase [Source:UniProtKB/Swiss-Prot;Acc:Q42546]	25610002	25611802	39.59
AT5G63580	FLS2	Putative inactive flavonol synthase 2 [Source:UniProtKB/Swiss-Prot;Acc:B2GVM7]	25454578	25456476	35.97
AT5G64660	PUB27	U-box domain-containing protein 27 [Source:UniProtKB/Swiss-Prot;Acc:Q9FLF4]	25841913	25843555	41.87
AT5G61600	ERF104	Ethylene-responsive transcription factor ERF104 [Source:UniProtKB/Swiss-Prot;Acc:Q9FKG1]	24766344	24767732	39.88
AT5G60970	TCP5	Transcription factor TCP5 [Source:UniProtKB/Swiss-Prot;Acc:Q9FME3]	24535337	24537244	36.37
AT5G63420	emb2746	Ribonuclease J [Source:UniProtKB/Swiss-Prot;Acc:Q84W56]	25400317	25406250	38.96
AT5G63310	NDPK2	NDPK2 [Source:UniProtKB/TrEMBL;Acc:A0A384KNJ7]	25371904	25373904	37.88
AT5G64510	TIN1	Tunicamycin induced protein [Source:UniProtKB/TrEMBL;Acc:Q84JN2]	25784404	25786385	37.24
AT5G63590	FLS3	Flavonol synthase 3 [Source:UniProtKB/Swiss-Prot;Acc:Q9FFQ5]	25456910	25458639	39.08
AT5G64370	PYD3	Beta-ureidopropionase [Source:UniProtKB/Swiss-Prot;Acc:Q8H183]	25739131	25741222	39.67

AT5G64360		AT5G64360 protein [Source:UniProtKB/TrEMBL;Acc:Q9FMF3]	25736171	25738676	43.02
AT5G62840		Phosphoglycerate mutase family protein [Source:UniProtKB/TrEMBL;Acc:Q9FM11]	25228139	25229738	40.12
AT5G64230		unknown protein; FUNCTIONS IN: molecular_function unknown; INVOLVED IN: biological_process unknown; LOCATED IN: endomembrane	25692425	25694137	41.1
AT5G64390	HEN4	RNA-binding KH domain-containing protein [Source:UniProtKB/TrEMBL;Acc:F4KDM9]	25743344	25748575	39.91
AT5G62780		Chaperone DnaJ-domain superfamily protein [Source:UniProtKB/TrEMBL;Acc:F4K7T4]	25213386	25214137	42.82
AT5G63700		Zinc ion binding / DNA binding protein [Source:UniProtKB/TrEMBL;Acc:F4KAT5]	25493707	25497764	36.57
AT5G63920	TOP3A	DNA topoisomerase 3-alpha [Source:UniProtKB/Swiss-Prot;Acc:Q9LVP1]	25574419	25581433	38.89
AT5G64130		AT5G64130 protein [Source:UniProtKB/TrEMBL;Acc:A8MS32]	25664290	25666393	37.88
AT5G63690		Nucleic acid-binding, OB-fold-like protein [Source:UniProtKB/TrEMBL;Acc:Q9FFP5]	25492772	25493699	41.06
AT5G64140	RPS28C	RPS28 [Source:UniProtKB/TrEMBL;Acc:A0A178UA91]	25667324	25668777	38.1
AT5G64400		CONTAINS InterPro DOMAIN/s: CHCH (InterPro:IPR010625); BEST Arabidopsis thaliana protein match is: Cox19-like CHCH family protein (	25747856	25750830	36.71
AT5G64010		unknown protein; FUNCTIONS IN: molecular_function unknown; INVOLVED IN: biological_process unknown; LOCATED IN: cellular_compo	25618606	25620223	40.05
AT5G64690		At5g64690 [Source:UniProtKB/TrEMBL;Acc:Q9FGG4]	25862715	25864792	33.88
AT5G61605	ESFL10	EMBRYO SURROUNDING FACTOR 1-like protein 10 [Source:UniProtKB/Swiss-Prot;Acc:Q1PDG8]	24768382	24769010	33.23
AT5G62810	PEX14	Peroxisomal membrane protein PEX14 [Source:UniProtKB/Swiss-Prot;Acc:Q9FXT6]	25220147	25223789	38.54
AT5G62350		Plant invertase/pectin methylesterase inhibitor superfamily protein [Source:UniProtKB/TrEMBL;Acc:Q9LVA4]	25037191	25038424	40.6
AT5G64650		At5g64650 [Source:UniProtKB/TrEMBL;Acc:Q1ECK2]	25839244	25841023	36.69
AT5G61300		Gb [Source:UniProtKB/TrEMBL;Acc:Q9FLK3]	24650704	24653269	40.1
AT5G64816		Uncharacterized protein At5g64816 [Source:UniProtKB/Swiss-Prot;Acc:Q8L8Q8]	25912969	25914027	37.49
AT5G62090	SLK2	Probable transcriptional regulator SLK2 [Source:UniProtKB/Swiss-Prot;Acc:Q94BP0]	24934962	24939239	40.3
AT5G61090		Gb [Source:UniProtKB/TrEMBL;Acc:Q9FNQ6]	24574652	24578200	41.81
AT5G64020	TBL14	Protein trichome birefringence-like 14 [Source:UniProtKB/Swiss-Prot;Acc:Q0WPS0]	25620246	25622646	38.44
AT5G62920	ARR6	Response regulator 6 [Source:UniProtKB/TrEMBL;Acc:Q0WSS6]	25252007	25254318	34.13
AT5G62790	DXR	1-deoxy-D-xylulose 5-phosphate reductoisomerase [Source:UniProtKB/TrEMBL;Acc:F4K7T6]	25214125	25217469	37.7
AT5G61060	HDA05	Histone deacetylase [Source:UniProtKB/TrEMBL;Acc:F4K1Z5]	24566783	24571072	39.3
AT5G63000		Mitochondrial import inner membrane translocase subunit Tim17/Tim22/Tim23 family protein [Source:UniProtKB/TrEMBL;Acc:Q84K84]	25280012	25281652	39.79
AT5G62860		F-box associated ubiquitination effector family protein [Source:UniProtKB/TrEMBL;Acc:F4K7U3]	25232632	25233541	40.66
AT5G63380	4CLL9	4-coumarate--CoA ligase-like 9 [Source:UniProtKB/Swiss-Prot;Acc:Q84P23]	25387411	25390227	41.78
AT5G64830		AT5g64830/MXK3_5 [Source:UniProtKB/TrEMBL;Acc:Q9LV94]	25914567	25916652	38.64
AT5G61840	IRX10L	GUT1 [Source:UniProtKB/TrEMBL;Acc:A0A178UAL2]	24839177	24842143	40.14
AT5G62540	UBC3	UBC3 [Source:UniProtKB/TrEMBL;Acc:A0A178ULQ4]	25103934	25105619	34.46
AT5G61640	PMSR1	peptidemethionine sulfoxide reductase 1 [Source:TAIR;Acc:AT5G61640]	24774980	24776398	39.75
AT5G61390	NEN2	Protein NEN2 [Source:UniProtKB/Swiss-Prot;Acc:Q0V842]	24678555	24681078	40.53
AT5G61410	RPE	RPE [Source:UniProtKB/TrEMBL;Acc:A0A178UPG9]	24683606	24686217	38.25
AT5G63770	DGK2	Diacylglycerol kinase 2 [Source:UniProtKB/Swiss-Prot;Acc:Q9FFN7]	25518404	25523621	38
AT5G63710		Leucine-rich repeat protein kinase family protein [Source:TAIR;Acc:AT5G63710]	25499378	25503023	36.67
AT5G61780	TSN2	Ribonuclease TUDOR 2 [Source:UniProtKB/Swiss-Prot;Acc:Q9FLT0]	24821203	24826969	40.54
AT5G63020		Probable disease resistance protein At5g63020 [Source:UniProtKB/Swiss-Prot;Acc:Q8RXS5]	25283063	25286121	40.93
AT5G61520	STP3	AT5G61520 protein [Source:UniProtKB/TrEMBL;Acc:B9DGV5]	24739026	24741401	40.53
AT5G61430	NAC100	NAC domain-containing protein 100 [Source:UniProtKB/Swiss-Prot;Acc:Q9FLJ2]	24701062	24702787	37.78
AT5G61670	OR	Protein ORANGE, chloroplastic [Source:UniProtKB/Swiss-Prot;Acc:Q9FKF4]	24783429	24786265	38.03
AT5G62100	ATBAG2	BCL-2-associated athanogene 2 [Source:TAIR;Acc:AT5G62100]	24940203	24942003	34.65
AT5G62930		SGNH hydrolase-type esterase superfamily protein [Source:TAIR;Acc:AT5G62930]	25254821	25256619	40.41
AT5G61495		unknown protein; FUNCTIONS IN: molecular_function unknown; INVOLVED IN: biological_process unknown; LOCATED IN: cellular_compo	24732768	24733485	40.95
AT5G64600	OFUT38	O-fucosyltransferase 38 [Source:UniProtKB/Swiss-Prot;Acc:F4KF16]	25824997	25828185	37.88
AT5G61280		Gb [Source:UniProtKB/TrEMBL;Acc:Q9FLK5]	24643556	24644990	30.94
AT5G62600	MOS14	Transportin MOS14 [Source:UniProtKB/Swiss-Prot;Acc:Q8GUL2]	25122803	25132716	36.35
AT5G62570		Calmodulin binding protein-like [Source:TAIR;Acc:AT5G62570]	25113948	25117314	38.02
AT5G64050	OVA3	Glutamate--tRNA ligase, chloroplastic/mitochondrial [Source:UniProtKB/Swiss-Prot;Acc:Q9FEA2]	25629832	25633209	39.08
AT5G61540		Probable isoaspartyl peptidase/L-asparaginase 3 [Source:UniProtKB/Swiss-Prot;Acc:Q56W64]	24744853	24747542	39.03
AT5G64780		Holocarboxylase synthetase [Source:UniProtKB/TrEMBL;Acc:Q9FGF5]	25900412	25902127	38.23
AT5G61470		C2H2-like zinc finger protein [Source:UniProtKB/TrEMBL;Acc:Q9FII6]	24722870	24723983	36.89
AT5G63650	SRK2H	Serine/threonine-protein kinase SRK2H [Source:UniProtKB/Swiss-Prot;Acc:Q9FFP9]	25481334	25483799	38.73
AT5G61440	ACHT5	Thioredoxin-like 1-2, chloroplastic [Source:UniProtKB/Swiss-Prot;Acc:Q9XFI1]	24707445	24709396	34.02
AT5G62610	BHLH79	Transcription factor bHLH79 [Source:UniProtKB/Swiss-Prot;Acc:Q9LV17]	25132841	25136065	34.57
AT5G64810	WRKY51	Probable WRKY transcription factor 51 [Source:UniProtKB/Swiss-Prot;Acc:Q93WU9]	25908247	25909881	30.64
AT5G61360		At5g61360 [Source:UniProtKB/TrEMBL;Acc:Q9FLJ7]	24670792	24671868	39.37
AT5G64350	FKBP12	Peptidyl-prolyl cis-trans isomerase FKBP12 [Source:UniProtKB/Swiss-Prot;Acc:Q8LGG0]	25734590	25736145	36.83
AT5G63510	GAMMA CAL1	Gamma carbonic anhydrase like 1 [Source:UniProtKB/TrEMBL;Acc:F4KAQ8]	25423967	25425871	41.31
AT5G64395		unknown protein; Ha. [Source:TAIR;Acc:AT5G64395]	25748202	25748297	39.58
AT5G61880	PAM16L1	Mitochondrial import inner membrane translocase subunit PAM16 like 1 [Source:UniProtKB/Swiss-Prot;Acc:Q93W66]	24850211	24851803	37.85
AT5G61350		Probable receptor-like protein kinase At5g61350 [Source:UniProtKB/Swiss-Prot;Acc:Q9FLJ8]	24667873	24670749	43.83
AT5G61040		Emb [Source:UniProtKB/TrEMBL;Acc:Q9FNR0]	24562333	24564979	41.25
AT5G64200	SC35	Serine/arginine-rich splicing factor SC35 [Source:UniProtKB/Swiss-Prot;Acc:Q9FMG4]	25681202	25683641	42.38
AT5G61850	LFY	floral meristem identity control protein LEAFY (LFY) [Source:TAIR;Acc:AT5G61850]	24844248	24846989	39.42
AT5G61150	VIP4	Protein LEO1 homolog [Source:UniProtKB/Swiss-Prot;Acc:Q9FNQ0]	24603634	24607725	40.03
AT5G62170		unknown protein; BEST Arabidopsis thaliana protein match is: unknown protein (TAIR:AT5G51850.1); Ha. [Source:TAIR;Acc:AT5G62170]	24972886	24975708	40.98
AT5G61490		Transmembrane protein [Source:UniProtKB/TrEMBL;Acc:Q9FKH0]	24732597	24733824	41.94
AT5G61820		Stress up-regulated Nod 19 protein [Source:UniProtKB/TrEMBL;Acc:Q8VZ21]	24834300	24836933	40.17
AT5G63905		unknown protein; FUNCTIONS IN: molecular_function unknown; INVOLVED IN: N-terminal protein myristoylation; LOCATED IN: cellular_c	25570793	25571699	34.73
AT5G62620	GALT6	Hydroxyproline O-galactosyltransferase GALT6 [Source:UniProtKB/Swiss-Prot;Acc:Q9LV16]	25136700	25140024	40.45
AT5G62210		Embryo-specific protein 3, (ATS3) [Source:UniProtKB/TrEMBL;Acc:Q9LVB5]	24986294	24987811	38.54
AT5G63625			25470218	25471984	30.79
AT5G63450	CYP94B1	cytochrome P450, family 94, subfamily B, polypeptide 1 [Source:TAIR;Acc:AT5G63450]	25408821	25410725	43.73
AT5G64760	RPN5B	26S proteasome non-ATPase regulatory subunit 12 homolog B [Source:UniProtKB/Swiss-Prot;Acc:Q8VWK0]	25893780	25896586	37.34
AT5G62340		At5g62340 [Source:UniProtKB/TrEMBL;Acc:Q9LVA5]	25033471	25034404	43.15
AT5G63050	EMB2759	At5g63050 [Source:UniProtKB/TrEMBL;Acc:Q6NM17]	25292404	25295020	38.14
AT5G62480	GSTU9	Glutathione S-transferase U9 [Source:UniProtKB/Swiss-Prot;Acc:Q9FUT0]	25088527	25089626	39.73
AT5G64500		Probable sphingolipid transporter spinster homolog 2 [Source:UniProtKB/Swiss-Prot;Acc:Q9FLG8]	25780382	25783753	37.66
AT5G64590		Emb [Source:UniProtKB/TrEMBL;Acc:Q9FLF9]	25822332	25823201	43.56
AT5G62390	BAG7	BAG family molecular chaperone regulator 7 [Source:UniProtKB/Swiss-Prot;Acc:Q9LVA0]	25051978	25054444	39.4
AT5G63940		At5g63940 [Source:UniProtKB/TrEMBL;Acc:Q9LVN9]	25587998	25591482	40.29
AT5G64820		Transmembrane protein [Source:UniProtKB/TrEMBL;Acc:Q9LV95]	25913999	25914523	39.81
AT5G63460		SAP domain-containing protein [Source:UniProtKB/TrEMBL;Acc:F4KAQ2]	25413286	25415592	37.02
AT5G64380		AT5g64380/MSJ1_22 [Source:UniProtKB/TrEMBL;Acc:Q9FMF1]	25741238	25743198	41.1
AT5G64401		unknown protein; LOCATED IN: mitochondrion; Ha. [Source:TAIR;Acc:AT5G64401]	25750441	25750602	41.36
AT5G64840	ABCF5	GCN5 [Source:UniProtKB/TrEMBL;Acc:A0A178UAA3]	25916722	25920254	38.61
AT5G64490	APUM26	Pumilio homolog 26 [Source:UniProtKB/Swiss-Prot;Acc:Q9FGE7]	25779225	25780308	40.77
AT5G63960		DNA polymerase [Source:UniProtKB/TrEMBL;Acc:F4KC69]	25599514	25606841	38.11
AT5G61570		Protein kinase superfamily protein [Source:UniProtKB/TrEMBL;Acc:F4K3J7]	24758189	24760789	36.99
AT5G63080		2-oxoglutarate (2OG) and Fe(II)-dependent oxygenase superfamily protein [Source:UniProtKB/TrEMBL;Acc:Q67ZB6]	25299933	25302476	36.56
AT5G62320	MYB99	MYB transcription factor [Source:UniProtKB/TrEMBL;Acc:Q9SNW9]	25028716	25029933	38.42
AT5G64570	BXL4	Beta-D-xylosidase 4 [Source:UniProtKB/Swiss-Prot;Acc:Q9FLG1]	25809975	25813648	41.05
AT5G63190		MA3 domain-containing protein [Source:UniProtKB/TrEMBL;Acc:Q94BR1]	25345542	25348800	40.38
AT5G62760		P-loop containing nucleoside triphosphate hydrolases superfamily protein [Source:UniProtKB/TrEMBL;Acc:F4K7T1]	25204472	25209583	37.77
AT5G61700	ABCA12	ABC transporter A family member 12 [Source:UniProtKB/Swiss-Prot;Acc:Q9FLT8]	24793779	24798745	39.12
AT5G63910	FLCY	Farnesylcysteine lyase [Source:UniProtKB/Swiss-Prot;Acc:P57681]	25571779	25574178	40.54
AT5G61770	PPAN	PETER PAN-like protein [Source:UniProtKB/TrEMBL;Acc:F4K3M1]	24815541	24817728	38.21
AT5G63810	BGAL10	Beta-galactosidase 10 [Source:UniProtKB/Swiss-Prot;Acc:Q9FN08]	25537074	25541529	39.79
AT5G61930	APO3	APO protein 3, mitochondrial [Source:UniProtKB/Swiss-Prot;Acc:Q9FH50]	24865086	24868812	40.84
AT5G63820		UPF0725 protein At5g63820 [Source:UniProtKB/Swiss-Prot;Acc:Q9FN07]	25541576	25542893	39.23
AT5G62750		At5g62750 [Source:UniProtKB/TrEMBL;Acc:Q9FM18]	25203425	25204320	32.92
AT5G61960	ML1	Protein MEI2-like 1 [Source:UniProtKB/Swiss-Prot;Acc:Q8W4I9]	24878507	24884852	38.35
AT5G62260	AHL6	AT-hook motif nuclear-localized protein 6 [Source:UniProtKB/Swiss-Prot;Acc:Q9LVB0]	25008843	25011666	37.92

Table S2. The list of common candidate genes (CGs) between current MQTL analysis and Arabidopsis GWAS result.

A. thaliana MQTL	Ch. no.	Gene ID	Gene name	Description	Genomic Position in bp	
MQTL-3/Chr1	1	*AT1G19850*	*ARF5*	Auxin response factor [Source:UniProtKB/TrEMBL;Acc:A0A178W993]	6886669	6891404
	1	*AT1G19860*	-	Zinc finger C-x8-C-x5-C-x3-H type family protein [Source:TAIR;Acc:AT1G19860]	6891382	6894752
	1	*AT1G19870*	*IQD32*	Protein IQ-DOMAIN 32 [Source:UniProtKB/Swiss-Prot;Acc:Q9FXI5]	6895009	6898818
	1	*AT1G19880*	-	Regulator of chromosome condensation (RCC1) family protein [Source:UniProtKB/TrEMBL;Acc:F4HQZ1]	6900337	6904155
	1	*AT1G19890*	*MGH3*	MGH3 [Source:UniProtKB/TrEMBL;Acc:A0A178WKL2]	6904963	6906028
	1	*AT1G19900*	-	At1g19900/F6F9_4 [Source:UniProtKB/TrEMBL;Acc:Q93Z02]	6906899	6908805
	1	*AT1G19910*	*VHA-C2*	V-type proton ATPase proteolipid subunit [Source:UniProtKB/TrEMBL;Acc:Q24JM2]	6913001	6914634
	1	*AT1G19930*	-	Putative F-box/kelch-repeat protein At1g19930 [Source:UniProtKB/Swiss-Prot;Acc:Q3ED92]	6917020	6918138
	1	*AT1G19940*	*AtGH9B5*	Endoglucanase 2 [Source:UniProtKB/Swiss-Prot;Acc:Q9FXI9]	6918159	6921979
	1	*AT1G19950*	*HVA22H*	HVA22-like protein h [Source:UniProtKB/Swiss-Prot;Acc:Q8LEM6]	6924091	6926741
	1	*AT1G19960*	-	Putative uncharacterized protein At1g19960 [Source:UniProtKB/TrEMBL;Acc:Q9LNS5]	6927736	6928656
MQTL-3/Chr3	3	*AT3G15640*	*COX5B-1*	Cytochrome c oxidase subunit 5b-1, mitochondrial [Source:UniProtKB/Swiss-Prot;Acc:Q9LW15]	5298991	5301745
	3	*AT3G15650*	-	Alpha/beta-Hydrolases superfamily protein [Source:UniProtKB/TrEMBL;Acc:F4J010]	5305549	5307979
	3	*AT3G15660*	*GRXS15*	GRX4 [Source:UniProtKB/TrEMBL;Acc:A0A178VHP8]	5307694	5309624
	3	*AT3G15670*	*LEA29*	Late embryogenesis abundant protein 29 [Source:UniProtKB/Swiss-Prot;Acc:Q9LW12]	5309886	5310988
	3	*AT3G15680*	-	At3g15680 [Source:UniProtKB/TrEMBL;Acc:Q9LW11]	5314817	5316617
	3	*AT3G15690*	-	Putative acetyl-CoA carboxylase biotin-containing subunit [Source:UniProtKB/TrEMBL;Acc:Q8GRT9]	5316916	5320121
	3	*AT3G15700*	-	P-loop containing nucleoside triphosphate hydrolases superfamily protein [Source:TAIR;Acc:AT3G15700]	5320644	5322432
	3	*AT3G15710*	-	Signal peptidase I [Source:UniProtKB/TrEMBL;Acc:Q9LW08]	5323262	5324966
	3	*AT3G15720*	-	Probable polygalacturonase At3g15720 [Source:UniProtKB/Swiss-Prot;Acc:Q9LW07]	5325127	5327672
	3	*AT3G15730*	*PLDALPHA1*	Phospholipase D alpha 1 [Source:UniProtKB/Swiss-Prot;Acc:Q38882]	5330194	5333745
	3	*AT3G15740*	-	RING/U-box superfamily protein [Source:UniProtKB/TrEMBL;Acc:Q9LW05]	5333769	5334553
	3	*AT3G15750*	-	Essential protein Yae1, N-terminal [Source:UniProtKB/TrEMBL;Acc:Q9LW04]	5334844	5336485
	3	*AT3G15760*	-	At3g15760 [Source:UniProtKB/TrEMBL;Acc:Q9LW03]	5337475	5338562
	3	*AT3G15770*	-	AT3G15770 protein [Source:UniProtKB/TrEMBL;Acc:B9DGF6]	5339992	5341339
	3	*AT3G15780*	-	Transmembrane protein [Source:UniProtKB/TrEMBL;Acc:Q9LW01]	5341356	5342557
	3	*AT3G15790*	*MBD11*	Methyl-CpG-binding domain-containing protein 11 [Source:UniProtKB/Swiss-Prot;Acc:Q9LW00]	5342712	5344715
MQTL-4/Chr3	3	*AT3G23570*	-	Alpha/beta-Hydrolases superfamily protein [Source:UniProtKB/TrEMBL;Acc:Q94JY3]	8457767	8459990
	3	*AT3G23580*	*RNR2A*	Ribonucleoside-diphosphate reductase small chain A [Source:UniProtKB/Swiss-Prot;Acc:P50651]	8459838	8462815
	3	*AT3G23590*	*MED33A*	Mediator of RNA polymerase II transcription subunit 33A [Source:UniProtKB/Swiss-Prot;Acc:Q9LUG9]	8467398	8473627
	3	*AT3G23600*	-	AT3g23600/MDB19_9 [Source:UniProtKB/TrEMBL;Acc:Q9LUG8]	8473627	8476024
	3	*AT3G23605*	*PUX12*	Plant UBX domain-containing protein 12 [Source:UniProtKB/Swiss-Prot;Acc:Q9LUG7]	8476482	8477392
	3	*AT3G23610*	*DSPTP1*	dual specificity protein phosphatase 1 [Source:TAIR;Acc:AT3G23610]	8477800	8479877
	3	*AT3G23620*	-	Ribosome production factor 2 homolog [Source:UniProtKB/Swiss-Prot;Acc:Q9LUG5]	8480025	8482222
	3	*AT3G23630*	*IPT7*	Adenylate isopentenyltransferase 7, mitochondrial [Source:UniProtKB/Swiss-Prot;Acc:Q94ID1]	8488695	8490266
	3	*AT3G23633*	-	F-box associated ubiquitination effector family protein [Source:UniProtKB/Swiss-Prot;Acc:Q9LUG3]	8496440	8497281
	3	*AT3G23635*	*RTFL13*	ROTUNDIFOLIA like 13 [Source:UniProtKB/TrEMBL;Acc:A8MRE9]	8497679	8498390
MQTL-5/Chr3	3	*AT3G42473*	*LCR47*	Defensin-like protein 91 [Source:UniProtKB/Swiss-Prot;Acc:P82762]	14607080	14607971
	3	*AT3G42550*	-	Eukaryotic aspartyl protease family protein [Source:UniProtKB/TrEMBL;Acc:F4JF07]	14665728	14669135
MQTL-2/Chr4	4	*AT4G05040*	-	AT4G05040 protein [Source:UniProtKB/TrEMBL;Acc:Q94A85]	2578594	2582290
	4	*AT4G05050*	*UBQ11*	Polyubiquitin [Source:UniProtKB/TrEMBL;Acc:Q8H0Y0]	2588004	2589437
	4	*AT4G05060*	*PVA43*	Vesicle-associated protein 4-3 [Source:UniProtKB/Swiss-Prot;Acc:Q8LPQ7]	2590065	2592541
	4	*AT4G05070*	-	AT4g05070 protein [Source:UniProtKB/TrEMBL;Acc:Q9S9T1]	2593595	2594125
	4	*AT4G05080*	-	F-box/kelch-repeat protein At4g05080 [Source:UniProtKB/Swiss-Prot;Acc:Q9S9T0]	2603001	2604176
	4	*AT4G05090*	-	3'(2'),5'-bisphosphate nucleotidase-like protein [Source:UniProtKB/TrEMBL;Acc:Q682R6]	2609066	2611762
	4	*AT4G05091*	-	unknown protein; Ha. [Source:TAIR;Acc:AT4G05091]	2611781	2612195
	4	*AT4G05095*	-	sis thaliana protein match is: RNA-directed DNA polymerase (reverse transcriptase)-related family protein (TAIR:AT4G04650.1); Ha. [Source:TAIR;A	2613798	2614214
	4	*AT4G05100*	*AtMYB74*	Transcription factor MYB74 [Source:UniProtKB/Swiss-Prot;Acc:Q9M0Y5]	2618372	2619947
	4	*AT4G08140*	-	26S proteasome non-ATPase regulatory subunit-like protein [Source:UniProtKB/TrEMBL;Acc:Q9SYE4]	5135673	5136140
	4	*AT4G08150*	*KNAT1*	Homeobox protein knotted-1-like 1 [Source:UniProtKB/Swiss-Prot;Acc:P46639]	5147699	5151159
	4	*AT4G08160*	*XYN3*	Endo-1,4-beta-xylanase 3 [Source:UniProtKB/Swiss-Prot;Acc:F4JG10]	5159033	5162867
	4	*AT4G08170*	*ITPK3*	Inositol-tetrakisphosphate 1-kinase 3 [Source:UniProtKB/Swiss-Prot;Acc:Q9SUG3]	5163220	5167192
	4	*AT4G08180*	*ORP1C*	Oxysterol-binding protein-related protein 1C [Source:UniProtKB/Swiss-Prot;Acc:Q8L751]	5169459	5174107
MQTL-3/Chr4	4	*AT4G10420*	-	FBD / Leucine Rich Repeat domains containing protein [Source:UniProtKB/TrEMBL;Acc:F4JLN7]	6452811	6454209
	4	*AT4G10430*	-	TMPIT-like protein [Source:UniProtKB/TrEMBL;Acc:Q93XX0]	6454703	6458146
	4	*AT4G10440*	-	Probable methyltransferase PMT17 [Source:UniProtKB/Swiss-Prot;Acc:Q9SZX8]	6459539	6462092
	4	*AT4G10450*	*RPL9D*	60S ribosomal protein L9-2 [Source:UniProtKB/Swiss-Prot;Acc:Q9SZX9]	6462918	6464558
	4	*AT4G10457*	*SCRL1*	Putative defensin-like protein 225 [Source:UniProtKB/Swiss-Prot;Acc:P82620]	6466900	6467270
	4	*AT4G10465*	*HIPP44*	Heavy metal-associated isoprenylated plant protein 44 [Source:UniProtKB/Swiss-Prot;Acc:F4JMB8]	6473547	6475434
	4	*AT4G10470*	-	AT4g10470/F7L13_50 [Source:UniProtKB/TrEMBL;Acc:Q944H1]	6475984	6477883
	4	*AT4G10480*	-	Putative alpha NAC [Source:UniProtKB/TrEMBL;Acc:Q0WWN5]	6477876	6479171
	4	*AT4G10490*	*DLO2*	Protein DMR6-LIKE OXYGENASE 2 [Source:UniProtKB/Swiss-Prot;Acc:Q9ZSA7]	6483867	6485360
	4	*AT4G10500*	*DLO1*	Protein DMR6-LIKE OXYGENASE 1 [Source:UniProtKB/Swiss-Prot;Acc:Q9ZSA8]	6491017	6492578
	4	*AT4G10590*	*UBP9*	AT4G10590 protein [Source:UniProtKB/TrEMBL;Acc:B9DFI6]	6538406	6544915
	4	*AT4G10595*	*LCR2*	low-molecular-weight cysteine-rich 2 [Source:TAIR;Acc:AT4G10595]	6545340	6546616
	4	*AT4G10600*	-	RING/FYVE/PHD zinc finger superfamily protein [Source:UniProtKB/TrEMBL;Acc:Q9T0B9]	6547235	6548878
	4	*AT4G10603*	-	Putative defensin-like protein 169 [Source:UniProtKB/Swiss-Prot;Acc:Q2V3K0]	6550922	6551975
	4	*AT4G10610*	*CID12*	RBP37 [Source:UniProtKB/TrEMBL;Acc:A0A178UWM0]	6556982	6559602
	4	*AT4G10613*	-	RNA-directed DNA polymerase (Reverse transcriptase)-related family protein [Source:UniProtKB/TrEMBL;Acc:Q2V3J8]	6560363	6561726
	4	*AT4G10620*	-	F3H7.11 protein [Source:UniProtKB/TrEMBL;Acc:Q9ZSB8]	6564183	6566504
	4	*AT4G10630*	-	F3H7.9 protein [Source:UniProtKB/TrEMBL;Acc:Q9ZSB9]	6566625	6567740
	4	*AT4G10640*	*IQD16*	At4g10640 [Source:UniProtKB/TrEMBL;Acc:Q7XA83]	6571894	6574586
MQTL-4/Chr4	4	*AT4G22580*	*GT19*	Probable xyloglucan galactosyltransferase GT19 [Source:UniProtKB/Swiss-Prot;Acc:Q9SUW1]	11889098	11890808
	4	*AT4G22590*	*TPPG*	Trehalose 6-phosphate phosphatase [Source:UniProtKB/TrEMBL;Acc:A0A178UWC3]	11893499	11896480
	4	*AT4G22610*	-	AT4g22610/F7K2_190 [Source:UniProtKB/TrEMBL;Acc:Q9SUV8]	11901703	11902642
	4	*AT4G22620*	-	Auxin-responsive family protein [Source:UniProtKB/TrEMBL;Acc:O49643]	11907355	11908357
	4	*AT4G22630*	-	Bifunctional inhibitor/lipid-transfer protein/seed storage 2S albumin superfamily protein [Source:UniProtKB/TrEMBL;Acc:O49644]	11908673	11910013
	4	*AT4G22650*	-	Lipid transfer protein [Source:UniProtKB/TrEMBL;Acc:Q9SUV6]	11913557	11914027
	4	*AT4G22660*	-	Putative F-box protein At4g22660 [Source:UniProtKB/Swiss-Prot;Acc:O49647]	11914981	11916171
	4	*AT4G22666*	-	Bifunctional inhibitor/lipid-transfer protein/seed storage 2S albumin superfamily protein [Source:UniProtKB/TrEMBL;Acc:Q1G3I0]	11917057	11917959
	4	*AT4G22680*	*MYB85*	MYB transcription factor [Source:UniProtKB/TrEMBL;Acc:Q94GA6]	11922340	11924230
MQTL-2/Chr5	5	*AT5G15420*	-	Uncharacterized protein T20K14_30 [Source:UniProtKB/TrEMBL;Acc:Q9LF40]	5007826	5008964
	5	*AT5G15430*	-	At5g15430 [Source:UniProtKB/TrEMBL;Acc:Q9LF39]	5009687	5011856
	5	*AT5G15440*	*EDL1*	EID1-like F-box protein 1 [Source:UniProtKB/Swiss-Prot;Acc:Q9LF38]	5012061	5014281
	5	*AT5G15450*	*CLPB3*	Chaperone protein ClpB3, chloroplastic [Source:UniProtKB/Swiss-Prot;Acc:Q9LF37]	5014212	5018347
	5	*AT5G15460*	*MUB2*	Membrane-anchored ubiquitin-fold protein 2 [Source:UniProtKB/Swiss-Prot;Acc:Q8LCS8]	5018680	5020926
	5	*AT5G15470*	*GAUT14*	GAUT14 [Source:UniProtKB/TrEMBL;Acc:A0A384LD84]	5020941	5024750
	5	*AT5G15480*	-	C2H2-type zinc finger family protein [Source:UniProtKB/TrEMBL;Acc:Q9LF34]	5025952	5027160
	5	*AT5G15490*	*UGD3*	UDP-glucose 6-dehydrogenase [Source:UniProtKB/TrEMBL;Acc:A0A178UJW9]	5027638	5030245
	5	*AT5G15500*	-	Ankyrin repeat family protein [Source:UniProtKB/TrEMBL;Acc:Q9LF32]	5031648	5033540
	5	*AT5G15510*	-	TPX2 (Targeting protein for Xklp2) protein family [Source:UniProtKB/TrEMBL;Acc:F4K9U0]	5033933	5036824
	5	*AT5G15520*	*RPS19B*	40S ribosomal protein S19-2 [Source:UniProtKB/Swiss-Prot;Acc:Q9LF30]	5037021	5038754
	5	*AT5G15530*	*BCCP2*	Biotin carboxyl carrier protein of acetyl-CoA carboxylase [Source:UniProtKB/TrEMBL;Acc:A0A178U9S1]	5038563	5040840
MQTL-7/Chr5	5	*AT5G46270*	-	Disease resistance protein (TIR-NBS-LRR class) family [Source:TAIR;Acc:AT5G46270]	18763972	18769090
	5	*AT5G46280*	*MCM3*	DNA replication licensing factor MCM3 [Source:UniProtKB/Swiss-Prot;Acc:Q9FL33]	18769649	18773706
	5	*AT5G46290*	*KASI*	3-ketoacyl-acyl carrier protein synthase I [Source:UniProtKB/TrEMBL;Acc:F4KHF4]	18774056	18776839
	5	*AT5G46295*	-	Transmembrane protein [Source:UniProtKB/TrEMBL;Acc:Q3E8F8]	18779790	18780205
	5	*AT5G46300*	-	Putative uncharacterized protein [Source:UniProtKB/TrEMBL;Acc:Q9FL31]	18783585	18784786
	5	*AT5G46310*	-	WRKY family transcription factor [Source:UniProtKB/TrEMBL;Acc:Q9FL30]	18785672	18786956
	5	*AT5G46320*	-	MADS-box family protein [Source:UniProtKB/TrEMBL;Acc:Q9FL29]	18788086	18788910
	5	*AT5G46330*	*FLS2*	Leucine-rich repeat receptor-like protein kinase (Fragment) [Source:UniProtKB/TrEMBL;Acc:C0LGU8]	18791575	18795908
	5	*AT5G46340*	*RWA1*	RWA1 [Source:UniProtKB/TrEMBL;Acc:A0A178UNJ7]	18795737	18800122
	5	*AT5G46350*	*WRKY8*	WRKY transcription factor 8 [Source:UniProtKB/Swiss-Prot;Acc:Q9FL26]	18801164	18804056
	5	*AT5G46360*	*KCO3*	Ca2+ activated outward rectifying K+ channel 3 [Source:TAIR;Acc:AT5G46360]	18806563	18808224
